# Evolutionary history of DNA methylation related genes in chordates: new insights from multiple whole genome duplications

**DOI:** 10.1038/s41598-020-57753-w

**Published:** 2020-01-22

**Authors:** Jingwei Liu, Huihua Hu, Stéphane Panserat, Lucie Marandel

**Affiliations:** 1 INRAE, Univ Pau & Pays de l′Adour, E2S-UPPA, UMR1419 Nutrition Metabolism and Aquaculture, Aquapôle, F-64310 Saint-Pée-sur-Nivelle, France; 2 State Key Laboratory of Freshwater Ecology and Biotechnology, Institute of Hydrobiology, Chinese Academy of Sciences, Wuhan, Hubei 430072 China; 3 University of Chinese Academy of Sciences, Beijing, 100049 China

**Keywords:** Phylogeny, Phylogeny, Molecular evolution, Molecular evolution, Gene expression

## Abstract

DNA methylation is an important epigenetic mechanism involved in many biological processes, *i*.*e*. gametogenesis and embryonic development. However, increased copy numbers of DNA methylation related genes (*dnmt*, *tet* and *tdg*) have been found during chordate evolution due to successive whole genome duplication (WGD) events. Their evolutionary history and phylogenetic relationships remain unclear. The present study is the first to clarify the evolutionary history of DNA methylation genes in chordates. In particular, our results highlight the fixation of several *dnmt3*-related genes following successive WGD throughout evolution. The rainbow trout genome offered a unique opportunity to study the early evolutionary fates of duplicated genes due to a recent round of WGD at the radiation of salmonids. Differences highlighted in transcriptional patterns of these genes during gametogenesis and ontogenesis in trout indicated that they might be subjected to sub- or neo-functionalisation after WDG. The fixation of multiple *dnmt3* genes in genomes after WGD could contribute to the diversification and plastic adaptation of the teleost.

## Introduction

DNA methylation is an important epigenetic mechanism involving the covalent binding of a methyl group to the 5^th^ carbon position of cytosine in CpG dinucleotides in vertebrates^1^. This mechanism is generally considered as a repressive epigenetic mark that inhibits gene expression^2^. DNA methylation plays a critical role in several biological processes such as embryonic development and gametogenesis^1,3,4^. The DNA methylation process is mediated by DNA methyltransferases (Dnmts): maintenance methyltransferase Dnmt1^5^ and *de novo* methyltransferase Dnmt3^6^. The erasure of methylation marks can be achieved either passively through the inhibition of Dnmt1 during DNA replication and cell division^7^, or actively through the action of ten-eleven translocation (Tet) dioxygenase family mediated iterative oxidation of 5-methylcytosine (5mC) and thymine DNA glycosylase (Tdg)-dependent base excision repair (BER)^8–10^.

The DNA methylation/demethylation machinery has been well described in mammals. While *dnmt1* is generally identified as single-copy gene during evolution^11^, multiple *dnmt3* genes were found in vertebrates with different gains and losses among tetrapod lineages^12^. The mammalian *dnmt3* family consists of four members: *dnmt3a*, *dnmt3b*, *dnmt3c* and *dnmt3l*^13,14^. *dnmt3l* serves as a catalytically inactive cofactor for *de novo* methylation, and is found to exist only in eutherian mammals and in some marsupials^15^, whilst *dnmt3c*, previously annotated as a pseudogene, was recently identified in rodent genomes^14^. In contrast, the discovery of active demethylation-related genes occurred fairly late, with three *tet* paralogs (*tet1*, *tet2* and *tet3*) and a single *tdg* gene found in mammalian genomes^9,10,16^. The identification of well-conserved DNA methylation genes in vertebrates, including teleosts, suggests that these regulatory pathways may be conserved across vertebrates^17,18^. However, due to the additional round of whole genome duplication (WGD) event that occurred before the radiation of the teleost lineage [TGD, teleost-specific genome duplication, 320 Mya (million years ago)], an increase in the number of copies of these genes has been found in teleost species^19^. For instance, the *de novo* methyltransferase *dnmt3* was shown to be more divergent in teleosts compared with mammals: despite the absence of *dnmt3l* in zebrafish (*Danio rerio*) genome, up to 6 *dnmt* genes were identified as orthologous to mammalian *dnmt3a* and *dnmt3b* genes^20–22^. Similarly, 4 to 9 different *dnmt3* paralogs were reported to exist in the genome of other teleost species^23–28^.

It is generally accepted that a WGD event can provide additional genetic material for selection, and are thus associated with phenotypic diversity and evolutionary innovations^29^. Alternatively, duplicated genes can also originate from small scale duplications (SSD) which can produce different kinds of adaptations compared to WGD^30^. Following WGD or SSD events, duplicated genes can either be lost or retained with three distinct outcomes: conservation of the ancestral gene functions, sub-functionalisation, or neo-functionalisation^31^. Through these processes, the fixation of extra copies of DNA methylation genes in teleost genomes may contribute to the diversification and plastic adaptation of teleosts. However, to characterise these adaptation, it is essential to first understand the evolutionary origin of these additional copies.

The increasing availability of sequenced genomes of teleost species facilitates to establish a comprehensive comparative study of DNA methylation genes among different taxa. However, few studies have been done to clarify the evolutionary history of *dnmt*, *tet* and *tdg* genes in vertebrates^22–24,32^. Hence, the evolutionary history and the orthologous relationship of DNA methylation genes remain incomplete and unclear, especially when considering genomes with higher complexity, *i*.*e*. salmonid species, which experienced a fourth round of WGD (Salmonid specific genome duplication, SaGD, 100 Mya).

The present study aimed to refine the current knowledge concerning the evolutionary history of *dnmt* genes in vertebrates, and to update the existing story with all DNA methylation genes (*dnmt*, *tet* and *tdg*) for extended taxa within the chordate phylum. To conduct the present study, we selected representative species with sequenced genomes of different taxa from a WGD point of view. Rainbow trout (*Oncorhynchus mykiss*), a salmonid fish, was included as a model species, which is supposed to have the maximum copies of DNA methylation genes among chordates due to SaGD. To explore if WGD events lead to sub- or neo-functionalisation of DNA methylation genes, estimations of the expression patterns of DNA methylation genes were done during gametogenesis and early development in trout.

## Results

### Evolutionary history of dnmt genes in chordates

#### Dnmt1

Through analysing the genomes of representative species in Ensembl (Release 91, December 2017) and in NCBI, we found that the *dnmt1* gene was generally identified as a single copy in the chordate phylum (Table 1), except that 2 *dnmt1* genes were identified in trout and Atlantic salmon (*Salmo salar*). Phylogenetic analyses showed that both *dnmt1* genes identified in trout and salmon grouped together with other vertebrate *dnmt1* orthologs, whereas the *dnmt1* genes of amphioxus (*Branchiostoma*. *floridae*), ciona (*Ciona intestinalis*) and lamprey (*Petromyzon marinus*) rooted as outgroups in the tree (Supplemental Fig. S1A, dnmt1 protein aliment in Supplemental Fig. S2). The subsequent syntenic analysis showed that the 2 *dnmt1* genes in trout were included in the same syntenic group conserved among vertebrates (*angptl6-eif3g-****dnmt1****-s1pr2-cdc37-tyk2-raver1*, Supplemental Fig. S1B). We thus annotated them as *dnmt1a* and *dnmt1b* according to ZFIN Nomenclature. Further analysis of conserved domain search showed that both the two *dnmt1* encoding protein sequences in trout have all the conserved domain features as their mammalian DNMT1 orthologs (DMAP1-binding domain, DNMT1-RFD domain, zf-CXXC domain, BAH domain, and Dcm domain). They also share identical conserved motifs in C-terminal catalytic domain (Supplemental Fig. S2).

**Table 1:** Copy numbers of DNA methylation genes in target species in chordates.

	dnmt1	dnmt3	tet	tdg
Amphioxus	1	1	1	1
Ciona	1	2 (?)	1	1
Lamprey	1	3 (A/A/A)	3 (1/2/3?)	1
Shark	1	2 (A/B)	3 (1/2/3)	1
Lizard	1	2 (A/B)	3 (1/2/3)	1
Chicken	1	2 (A/B)	3 (1/2/3)	1
Mouse	1	3 (A/B/C)	3 (1/2/3)	1
Spotted gar	1	3 (a/ba/bb)	3 (1/2/3)	1
Zebrafish	1	6 (aa/ab/ba/bba/bbb1/bbb2)	3 (1/2/3)	2 (a/b)
Medaka	1	4 (aa/ba/bba/bbb)	3 (1/2/3)	2 (a/b)
Stickleback	1	5 (aa/ab/ba/bba/bbb)	3 (1/2/3)	2 (a/b)
Fugu	1	5 (aa/ab/ba/bba/bbb)	3 (1/2/3)	2 (a/b)
Tetraodon	1	5 (aa/ab/ba/bba/bbb)	3 (1/2/3)	2 (a/b)
**Trout**	**2**	**8 (aa/ab1/ab2/ba1/ba2/bba1/bba2/bbb)**	**7 (1a/1b/2a/2b/2c/3a/3b)**	**4 (aa/ab/ba/bb)**

#### Dnmt3

**Figure 1:**
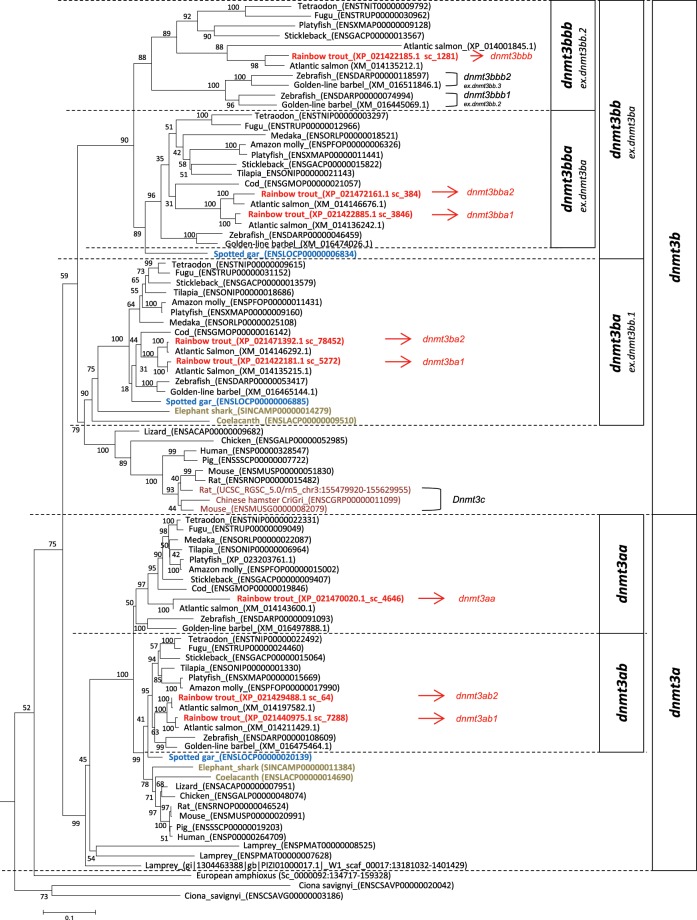
Phylogeny of dnmt3 in chordates. The phylogenetic tree was built by the Neighbor-Joining (NJ) method. The reliability of the inferred trees was estimated by the bootstrap method with 1,000 replications. All accession numbers are specified in parentheses.

We first evaluated the evolutionary history of *dnmt3* in non-vertebrate chordate species. Through a BLAST search against the amphioxus genome, we found that both *B*. *floridae* and *B*. *lanceolatum* had only one *dnmt3* gene located on BRAFLscaffold_185, and sc_0000092, respectively. In ciona (*C*. *savignyi*), two putative *dnmt3* homologs were identified (Table 1), with 45.5% of their identity shared in their protein sequences. Further analysis of conserved domain search showed that the *dnmt3*-related protein sequence in *B*. *lanceolatum* and one of the *dnmt3* in *C*. *savignyi* (ENSCSAVP00000020042) have all the domain features specific for *dnmt3*: PWWP domain, ADD domain and Dcm domain (Supplemental Fig. S3). But the other *dnmt3* in *C*. *savignyi* (ENSCSAVP00000020042) has only the Dcm domain in its protein sequence. The conserved dnmt3 amino acid sequences (sequences alignment including PWWP, ADD and Dcm domain) of representative species in chordates were used to perform phylogenetic analysis (alignment in Supplemental Fig. S3), results showed that amphioxus and the ciona dnmt3 rooted as outgroup in the tree, whereas vertebrate dnmt3a and dnmt3b related sequences formed two separate groups (Fig. 1). Percentage identity matrix was calculated between this two dnmt3 related sequences in ciona and tetrapods, which showed that ciona sequences seemed no more related to dnmt3a than to dnmt3b (Supplemental Table S1). In order to clarify the specific timing for when *dnmt3a* and *dnmt3b* genes arose, we did a dnmt3 syntenic analysis in chordates using Genomicus software v01.01 (www.genomicus.biologie.ens.fr, Supplemental Fig. S4). We showed that the synteny around *dnmt3* genes in amphioxus and ciona was not well conserved except for *col21a1* and *akap7*, which were found on reftig_1 and reftig_44 in ciona, respectively. In vertebrates, *dnmt3a* and *dnmt3b* genes were included in two distinct syntenic groups: a*k7-abhd1-col21a1-akap7-epb41l2-sl3a1-zbtb24* and *pcsk2-dusp15-pofut1-b4galt5-zbtb46*, respectively.

We then investigate the evolutionary history of *dnmt3* in vertebrates. In jawless vertebrate, lamprey, two *dnmt3* genes annotated as *dnmt3a* (ENSPMAG00000007710 and ENSPMAG00000006895) were identified in the Ensembl database. However, we identified a 3^rd^ sequence in the lamprey genome assembly in NCBI (NCBI, GCA_002833325.1). We identified this sequence (W1 scaf_00017) as a novel *dnmt3* in lampreys. This new *dnmt3* gene and ENSPMAG00000007710 in lamprey have all the conserved domain features for *dnmt3*, whereas the ENSPMAG00000006895 has no PWWP domain (Supplemental Fig. S3). Percentage identity matrix (Supplemental Table S1) and phylogenetic analysis (Fig. 1) confirmed that all these three *dnmt3* related genes in lamprey shared higher conservation with *dnmt3a* gnathostoma genes than with *dnmt3b*. Syntenic analysis results also showed a partial syntenic conservation of the first group cited above around *dnmt3* genes found in lampreys, confirming that these genes were related to the *dnmt3a* gene in other vertebrates (Supplemental Fig. S4). We also verified this result in SIMRBASE. Noteworthy, there is another gene PMZ_0040501-RA annotated as *dnmt3b* in this database. However, we found that this latter sequence contains only a FYVE domain, and clustered with the mammalian *DNMT3L* orthologs (Data not shown).

**Figure 2:**
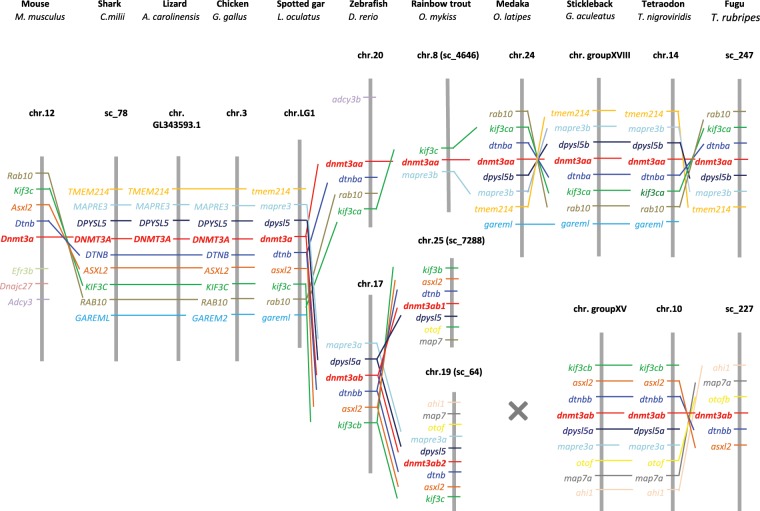
Syntenic analysis of *dnmt3a* in jawed vertebrates. Data were collected with Genomicus software version 01.01 and NCBI. chr., chromosome; sc., scaffold.

In jawed vertebrates, only one *dnmt3a* gene was identified in tetrapods, shark, coelacanth and spotted gar, but *dnmt3a* pseudogenes were identified in humans (ENSG00000224071, chr.2) and mice (ENSMUST00000192011.1, chr.3, Supplemental Fig. S4). By contrast, two *dnmt3a* genes were found in most of the teleost species (Table 1), which were well grouped as two independent clusters, *dnmt3aa* and *dnmt3ab*, in phylogenetic tree (Fig. 1). Both duplicated *dnmt3a* genes in teleost shared conserved synteny with other vertebrates (Fig. 2). Exceptionally, one *dnmt3aa* gene and two *dnmt3ab* genes were found in trout and salmon, whereas *dnmt3ab* was not found in medaka. Conserved domain analysis showed that no PWWP domain was identified in Dnmt3aa of salmon. Besides, rainbow trout Dnmt3aa lost one of the 6 conserved motifs (I, IV, VI, VIII, IX and X), motif IX, in its catalytic region, and its ADD domain sequence also showed less conserved compared to other DNMT3 related sequences (Supplemental Fig. S3).

Concerning *dnmt3b*, only one *dnmt3b* related gene was found in non-teleost vertebrates except for the spotted gar, whose genome contained 2 *dnmt3b* related genes in Ensembl annotated as *dnmt3ba* and *dnmt3bb*.*1*. The murine specific *Dnmt3c* genes were grouped together, and rooted with the *Dnmt3b* genes of rodents in phylogenetic tree (Fig. 1). In most of the teleost species, there were three *dnmt3b* genes identified in Ensembl, namely *dnmt3ba*, *dnmt3bb*.*1* and *dnmt3bb*.*2*. Phylogenetic tree showed that the *dnmt3bb*.*1* in spotted gar clustered with the teleost *dnmt3bb*.*1* genes, the elephant shark and the coelacanth *dnmt3b* orthologs and then rooted together with the tetrapod *dnmt3b* genes, whereas the gar *dnmt3ba* rooted together with teleost *dnmt3ba* and *dnmt3bb*.*2* genes as a separate branch (Fig. 1). We thus proposed to rename *dnmt3bb*.*1* and *dnmt3ba* in the spotted gar as *dnmt3ba* and *dnmt3bb* and the previous *dnmt3bb*.*1*, *dnmt3ba* and *dnmt3bb*.*2* in teleosts as *dnmt3ba*, *dnmt3bba* and *dnmt3bbb*, respectively according to ZFIN Nomenclature guidelines (Table 2). These genes will be referred by their new nomenclature henceforth in this paper.

**Table 2:** Proposed nomenclature for *dnmt3, tet* and *tdg* genes.

Species	Name proposed in Ensembl	Name proposed by Simoda *et al*.(2005)	Name proposed by Campos *et al*. (2012)	Proposed new nomenclature
Spotted gar	*dnmt3aa*			*dnmt3a*
	*dnmt3ba*			*dnmt3bb*
	*dnmt3bb*.*1*			*dnmt3ba*
	*tdg*.*1*			*tdg*
Zebrafish	*dnmt3aa*	*dnmt8*	*dnmt3a2*	*dnmt3aa*
	*dnmt3ab*	*dnmt6*	*dnmt3a1*	*dnmt3ab*
	*dnmt3bb*.*1*	*dnmt4*	*dnmt3b1*	*dnmt3ba*
	*dnmt3ba*	*dnmt7*	*dnmt3b2*	*dnmt3bba*
	*dnmt3bb*.*2*	*dnmt3*	*dnmt3b3*	*dnmt3bbb1*
	*dnmt3bb*.*3*	*dnmt5*	*dnmt3b4*	*dnmt3bbb2*
	*tdg1*			*tdga*
	*tdg2*			*tdgb*
	**Gene loci in Genomicus**	**Gene loci in NCBI**		**Proposed new nomenclature**
Trout	scaffold_644	chr.12		*dnmt1a*
	scaffold_1433	chr.13		*dnmt1b*
	scaffold_4646	chr.8		*dnmt3aa*
	scaffold_7288	chr.25		*dnmt3ab1*
	scaffold_64	chr.19		*dnmt3ab2*
	scaffold_5272	chr.16		*dnmt3ba1*
	scaffold_78452	chr.9		*dnmt3ba2*
	scaffold_3846	chr.16		*dnmt3bba1*
	scaffold_384	chr.9		*dnmt3bba2*
	scaffold_1281	chr.16		*dnmt3bbb*
	scaffold_18888 & 2097	chr.1		*tet1a*
	scaffold_189 & 12473	chr.23		*tet1b*
	scaffold_1163	chr.19: 4604994..4635364		*tet2a*
	/	chr.19: 4726729..4738210		*tet2b*
	/	chr.10		*tet2c*
	scaffold_21498 & 36080	chr.6		*tet3a*
	scaffold_605	chr.11		*tet3b*
	scaffold_2682	chr.4		*tdgaa*
	scaffold_82	chr.2		*tdgab*
	scaffold_2682	chr.4		*tdgba*
	scaffold_82	chr.2		*tdgbb*

**Figure 3:**
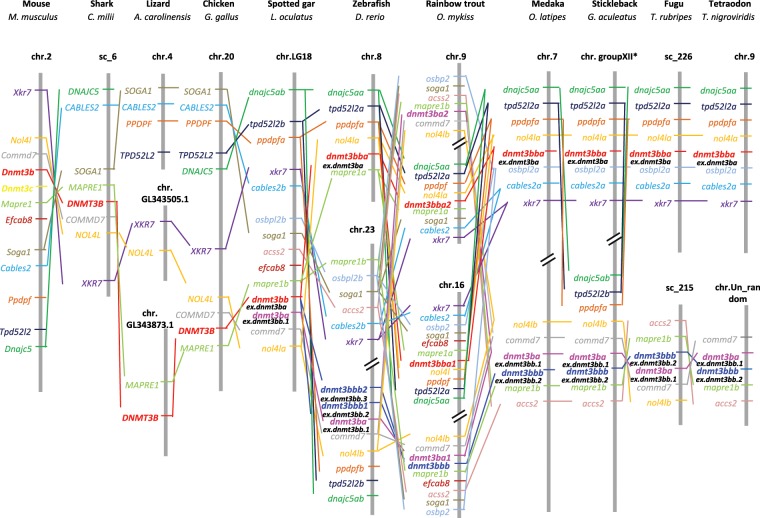
Syntenic analysis of dnmt3b in jawed vertebrates. Data were collected with Genomicus software version 01.01 and NCBI. chr., chromosome; sc., scaffold. *The synteny of stickleback is in reverse direction.

Regarding the dnmt3ba sub-tree, for most of non-salmonid teleost species, only one gene was found grouped together with non-teleost dnmt3b sequences. In salmonids, we identified 2 sequences related to dnmt3ba in our phylogenetic analysis which were located in the conserved and duplicated syntenic group *osbp2–soga1-acss2–mapre1b–****dnmt3ba****-commd –nol4lb* (Fig. 3).

For dnmt3bb sub-tree, the gar dnmt3bb sequence rooted at the basis of the dnmt3bba (ex. dnmt3ba) cluster, whereas dnmt3bbb (ex. dnmt3bb.2) sequences formed a separate cluster (Fig. 1). However, the conserved syntenic group of *dnmt3bb* in the spotted gar was identified on two distinct chromosomes in teleost species including *dnmt3bba* and *dnmt3bbb*. In salmonid species, 3 *dnmt3bb* paralogs were found in the trout genome, with two of them clustering with dnmt3bba, and the last one clustering with dnmt3bbb sequences (Fig. 1) in the phylogenetic tree. Both *dnmt3bba* related genes in trout were included in two well-conserved duplicated syntenic groups (*dnajc5aa–tpd5212a–ppdpfa-nol4la–****dnmt3bba****–osbpl2a–cables2a*, Fig. 3) across teleosts. *dnmt3bbb* was also included in the *nol4lb–commd7–****dnmt3ba****–****dnmt3bbb****–mapre1b* syntenic group conserved among teleosts. Compared with trout, salmon were found to have an additional *dnmt3bbb* paralog (XP_014001845.1) but it is very short in length (150aa). Besides, we found two *dnmt3bbb* (ex. *dnmt3bb*.*2*) paralogs (ex. *dnmt3/dnmt3bb*.*2* and ex. *dnmt5/dnmt3bb*.*3*) in zebrafish and another cyprinid fish, golden-line barbel. Further analysis of conserved domain search showed that the protein sequences of Dnmt3 orthologs are highly conserved across chordate phylum. Interestingly, all the Dnmt3bba and Dnmt3bbb related protein sequences have an extra CH domain in their N-terminal (Supplemental Fig. S3).

### Evolutionary history of DNA demethylation genes: tet and tdg genes

#### Tet

We identified one *tet* related sequence in each genome of amphioxus (BRAFLscaffold_344 in *B*. *floridae*; sc_00000095 in *B*. *lanceolatum ill*) and ciona (chromosome 12 in *C*. *intestinalis*; reftig_54 in *C*. *savignyi*) through a BLAST search (Table 1). Our results also showed that, most of the jawed vertebrates have three *tet* genes, namely *tet1*, *tet2* and *tet3* (Table 1) which were included in three distinct syntenic groups conserved among vertebrates (Supplemental Fig. S5). Interestingly, in amphioxus, the only *tet* gene was included in the *wnt8b-scd-****tet****-rassf-tmem2-cisd2-fam13-cdh-hnrnpd* syntenic group. Genes from this syntenic group were also found syntenic with *tet1*, *tet2* and to a lesser extent with *tet3* vertebrate genes, suggesting that these 3 genes shared a common ancestor. The phylogenetic analysis showed that tet1/2/3 formed three separate groups in the tree, whereas the amphioxus and ciona tet sequences rooted as an outgroup (Supplemental Fig. S6; protein alignment in Supplemental Fig. S7).

In lamprey, 3 hits were found on chromosomes scaf_00044, scaf_03335 and scaf_00172, respectively, in the Genbank assembly GCA_002833325.1.Further research in SIMRBASE confirmed this result. However, because the *tet* sequences on scaf_00172 and scaf_03335 were aligned to different region of putative *tet* gene on scaf_00044, thus it was impossible to include 3 of them together in phylogenetic tree. Our phylogenetic analysis showed that, the putative *tet* gene in lampreys on scaf_03335 clustered with *tet3*, whereas the other putative tet gene on scaf_00044 rooted together with ciona and amphioxus as an outgroup in the tree but the bootstrap value for this outgroup was very low (*i*.*e*. 18, Supplemental Fig. S6). Interestingly, the conserved syntenic group of both *tet1* and *tet2* in vertebrates were found on scaf_00044.

In trout, a total of seven *tet* paralogs were identified, with two orthologs of *tet1*, three orthologs of *tet2* and two orthologs of *tet3* (Table 1). *tet1* and *tet3* most probably duplicated before or around the salmonid radiation following the SaGD, giving rise respectively to *tet1a* and *tet1b*, and *tet3a* and *tet3b*. In the case of *tet2*, as previously mentioned, 3 duplicates (annotated as *tet2a*, *tet2b* and *tet2c*, respectively; Table 2) were identified in trout genomes. In trout, *tet2a* (XM_021573348.1) and *tet2b* (XM_021573351.1) were located close to one another on chromosome 19 (Supplemental Fig. S5) and shared extremely high identity in the cDNA sequence (96.5%), but *tet2b* was found to be only half the length of *tet2a*. The *tet2b* sequence corresponded to exons 6 to 12 of *tet2a*. Similarly, *tet2c* (XM_021617281.1) was found to be closely related to *tet2a* (89.6% identity), but again, the *tet2c* sequence was shorter than *tet2a* and corresponded to exons 1 to 5 of *tet2a*. There was thus no shared region between *tet2b* and *tet2c*, making it impossible to include the three *tet2* paralogs of trout together in the phylogenetic analysis (Supplemental Fig. S6). However, the high sequence identity shared by these 3 sequences and the conserved synteny around these 3 loci strongly confirmed that these genes were paralogs. Analysis in salmon also highlighted the 3 tet2 related sequences (XP_014050290.1, XP_014065146.1 and XP_014042431.1) within its genome. Further study of conserved domain search confirmed that that Tet2b and Tet2c share only part of the sequence with Tet2a sequence in trout, with Tet2b lacking the cysteine-rich domain whereas Tet2c lacking the DBSH domain in the C-terminal catalytic domain (Supplemental Fig. S7). Moreover, in most of the studied species, all the conserved motifs in catalytic domain of Tet families (*i*.*e*. iron and 2-OG binding sites) can be found (Supplemental Fig. S7), except that the Fe binding, 2-OG binding and 5mC binding sites at the end of C-terminal catalytic domain were not found in amphioxus, two putative tet (located on scaf_00044 and scaf_03335) in lamprey and Tet1a of salmon.

#### Tdg

Finally, with regards to *tdg* locus, our results showed that amphioxus, ciona, lamprey, shark, tetrapods and spotted gar had one copy of *tdg* in their genome (Table 1). In most of teleost species, there were two *tdg* paralogs (Table 1). Our phylogenetic analysis demonstrated that these two genes were co-orthologous to non-teleost *tdg* genes (Supplemental Fig. S8A, tdg protein aliment in Supplemental Fig. S9). The syntenic analysis (Supplemental Fig. S8B)showed that these 2 genes were side-by-side on the same chromosome and included in a syntenic group (*appl2-nuak1b-rps13-pik3c2a-****tdgb****-****tdga****-samm50-api5-nt5dc3-hsp90b1*) in teleosts, which is similar to the syntenic block of *tdg* in non-teleost species (*i*.*e*. *nuak1*, *appl2*, *nfyb*, *hsp90b1*, *nt5dc3*, and *samm50*). On the other hand, salmonids retained duplicates of *tdga* and *tdgb* genes (Table 1), which were therefore annotated as *tdgaa*, *tdgab*, *tdgba* and *tdgbb* (Table 2). Phylogenetic analysis showed that the duplicated *tdga* and *tdgb* genes in salmon and trout were well clustered with their respective orthologs in other teleosts. The syntenic region around *tdga*/*tdgb* in trout was similar to those in other teleost species, with *tdgaa*/*tdgba* and *tdgab*/*tdgbb* arranged in tandem on chromosome 4 and 2, respectively (Supplemental Fig. S8B). Conserved domain search demonstrated that the functional sites were highly conserved among all the analysed tdg-related sequences (Supplementary Fig. S9).

### Expression pattern of DNA methylation genes during gametogenesis and embryogenesis in trout

**Figure 4:**
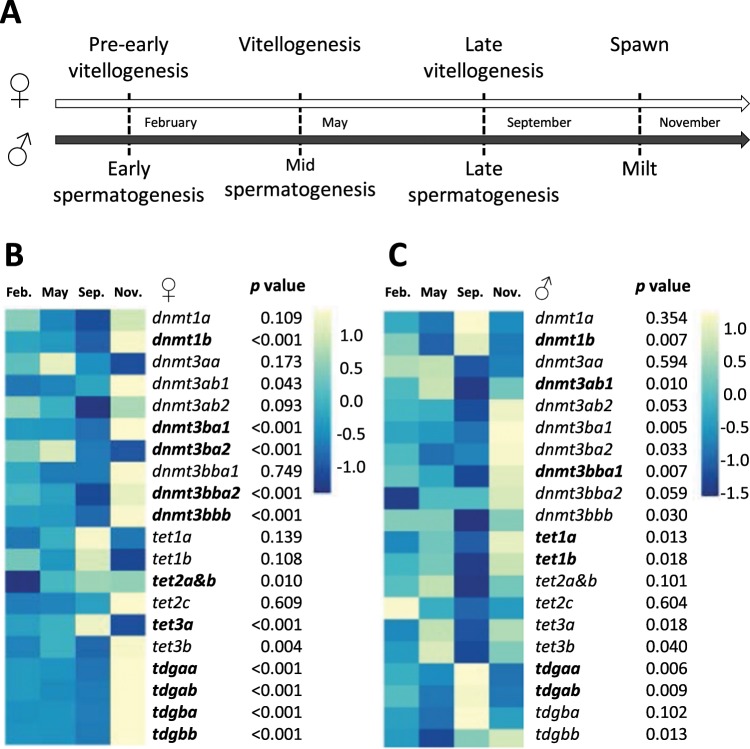
Expression patterns of DNA methylation genes during gametogenesis in trout. Experimental design. (**A**) Heatmaps were used to depict the mRNA levels of DNA methylation genes during oogenesis (**B**) and spermatogenesis (**C**) with the mean value of each stage (n = 6). When there is a significantly difference among groups (*p* < 0.05), the gene’s name is in bold.

**Figure 5:**
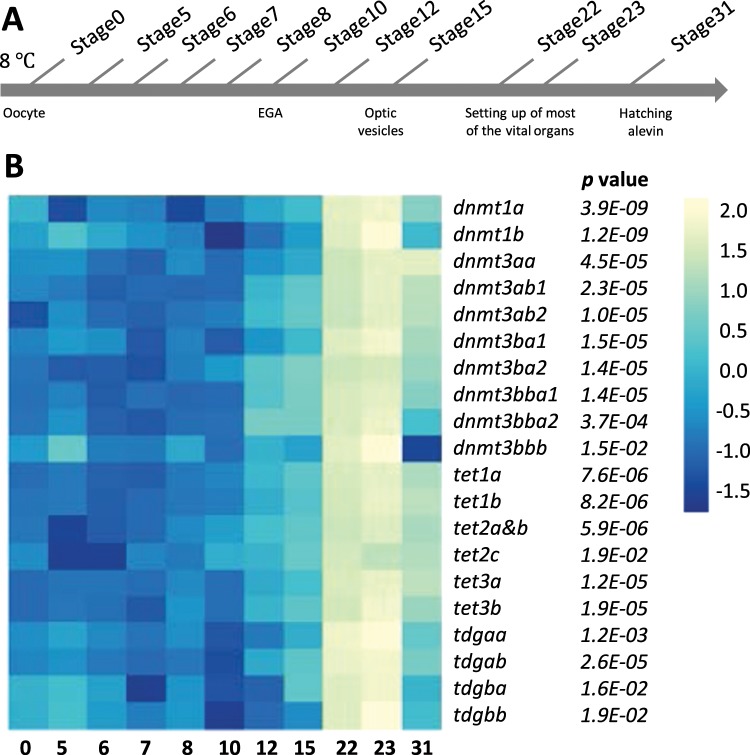
Expression patterns of DNA methylation genes during ontogenesis in trout. (**A**) Experimental design: Stages numbers referred to the Vernier developmental table 46. (**B**) The mRNA levels of DNA methylation genes during ontogenesis were shown in heatmap with the mean value of each stage. Data were subjected to log transformation. EGA, embryonic genome activation.

In the Phylofish database^33^ we found that the high expression of DNA methylation genes in reproduction and development-related tissues seems to have been conserved throughout chordate evolution (data not shown). Therefore, in order to explore the potential different function of duplicated DNA methylation genes after WGD, we investigated their expression patterns during gametogenesis (Fig. 4A) and embryogenesis (Fig. 5A) in trout, which has the maximum DNA methylation genes fixed in its genome.

During oogenesis, we observed that *dnmt1b*, *dnmt3ba1*, *dnmt3bba2*, *dnmt3bbb* exhibited a similar expression pattern (Fig. 4B, mean ± SD data in Supplemental Table S4), which remained steady from February to May (pre-early vitellogenesis and vitellogenesis stages), was halved in September (late vitellogenesis stage), and increased several fold in November (spawn stage). In contrast, the mRNA level of *dnmt3ba2* was significantly lower in September and November compared to February and May in trout ovaries (Fig. 4B). No significant difference was observed in the mRNA levels of *dnmt1a*, all *dnmt3a* paralogs and *dnmt3bba1* during oogenesis (Fig. 4B). Regarding *tet* and *tdg* genes, mRNA levels of both *tet1* ohnologs, *tet2c* and *tet3b* were not significantly different among different stages. As for *tet2a&b* (we were not able to design discriminating primers to separately amplify *tet2a* and *tet2b*), the mRNA level increased slightly but significantly during oogenesis (Fig. 4B). The same pattern was observed for *tet3a* except in spawned oocytes (November) in which *tet3a* mRNA level was lower than the other stages. All *tdg* paralogs displayed a similar pattern with a strong increase in mRNA levels at the spawn stage (November) (Fig. 4B).

In males, we observed that the expression profile of DNA methylation genes fluctuated during spermatogenesis (Fig. 4C, mean ± SD data in Supplemental Table S5). Increased mRNA level of *dnmt1b* was found in testes sampled in September (late spermatogenesis stage), whereas the expression of *dnmt3ab1* and *dnmt3bba1* decreased to its lowest level in September, and increased again to a high level in November (milt stage, Fig. 4C). No significant difference was found in mRNA levels for other *dnmt* genes during spermatogenesis (Fig. 4C). There was no significant difference in all *tet* genes in testes whatever the gonadal developmental stages, except for *tet1 ohnologs*, which displayed a significantly lower mRNA abundance in September than in earlier stages, but increased to a higher level in November (Fig. 4C). For tdg genes, *tdga* ohnologs displayed similar expression patterns and remarkably reached a maximum in September and then decreased to a low level in November. No significant difference was found in the mRNA level of *tdgb ohnologs* across all stages (Fig. 4C).

During trout ontogenesis (Fig. 5A), our results showed that mRNA levels for most DNA methylation genes displayed a similar expression pattern, with no or low mRNA levels from stages 0 to 15, followed by a remarkable increase to reach the maximum expression at stage 22/23, and then a decrease after hatching (Fig. 5B, mean ± SD data in Table S6). Exceptionally, relatively high mRNA abundances of *dnmt1b*, *dnmt3bbb* and *tdgb* ohnologs were observed at the beginning of ontogenesis. Besides, significantly high mRNA level of *dnmt3aa* and low mRNA level of *dnmt3bbb* were found at stage 31.

## Discussion

*In silico* analysis of DNA methylation genes in the present study showed that only one *dnmt1* gene was fixed in the genomes of most species in chordates, which was in accordance with previous studies^34,35^. A recent study concerning the molecular evolution of *dnmt1* in vertebrates demonstrated that an additional SSD event occurred before the radiation of major marsupial groups, giving rise to the fixation of two *dnmt1* copies in marsupials^11^. Here, we also identified two *dnmt1* genes in trout and salmon, which were supposed to arise from the SaGD thus being ohnologous genes.

Similar results were found for *tet1*, *tet2*, and *tet3*, which represent as single-copy gene across chordate evolution, except that amphioxus and ciona have only one *tet* related gene, whereas trout has 7 *tet* related genes. Previous studies did by Zhang *et al*.^36^ failed to identify any *tet* related genes in lamprey genome, probably due to the fact that their analyses were done with lamprey somatic genome, which contains only a part of the germline genome due to the programmed genome rearrangement event that happened specifically in lamprey^37^. In the present study, we identified 3 putative *tet* genes in lamprey, 1 of them are likely to be ortholog of *tet3*, but with a remaining question about the identity of the other two genes. So it still remains elusive to identify the origin of *tet1/2/3* during evolution. In terms of the fixation of multiple *tet* genes, especially the 3 *tet2* genes in salmonid species, it could be hypothesised that a lineage-specific duplication of *tet2* and synthenic genes may have occurred following the SaGD event.

Concerning *tdg* genes, our results showed an increase in the copy number of this gene during chordate evolution. Results in this study suggested that, a single copy of *tdg* was present at least in the gnathostomata ancestor, and a duplication of the *tdg* gene occurred before or around the teleost radiation, which resulted in the presence of the duplicated genes *tdga* and *tdgb* in the ancestral teleost. These results were in contrast with the previous hypothesis proposed by Best *et al*. (2018), which stated that the two *tdg* paralogs derived from a lineage-specific duplication event^28^. This is the reason why in the present study we renamed *tdg1* and *tdg2* as *tdga* and *tdgb*, respectively according to ZFIN Nomenclature guidelines. On the other hand, salmonids possess duplicates of *tdga* and *tdgb* genes, which most probably duplicated before or around the salmonid radiation and were retained following the SaGD.

Finally, the most important work in the present study should be the clarification of the evolutionary history of *dnmt3* genes in chordates. First, our investigation of the origin timing of *dnmt3a* and *dnmt3b* takes advantage of the inclusion of cephalochordate, tunicate and agnatha data. Campos *et al*. suggested that the ancestral *dnmt3a* and *dnmt3b* genes seem to have arisen during the VGD2 (second vertebrate whole genome duplication) event^22^, but the identification of multiple *dnmt3* genes in ciona and more specifically the confirmation of several *dnmt3a* genes in lamprey in the present study challenged this view. Several authors are now in support of the idea that one round of genome duplication (VDG1) occurred during the evolution of jawless vertebrates from chordate invertebrates, but whether the VGD2 occurred before or after the cyclostome-gnathostome split remains a controversy^38,39^. It can be thus hypothesised that *dnmt3a* and *dnmt3b* arose before the radiation of the lamprey ancestor, probably during VGD1 event. The *dnmt3b* seems to be lost in lamprey, whereas the 3 *dnmt3a* exist in its genome suggested that lamprey *dnmt3a* experienced several duplication events either through VGD2 followed by another SSD event, or through other possible duplication scenarios *i*.*e*. triplication, chromosome-scale duplications^37^.

On the other hand, we refined the evolutionary history of *dnmt3* in vertebrates, especially for *dnmt3b*. A previous study proposed that *dnmt3bba* (ex. *dnmt3b2/dnmt3ba*) was derived from TDG, whereas *dnmt3ba* (ex. *dnmt3b1/dnmt3bb*.*1*) and *dnmt3bbb* (ex. *dnmt3b3/dnmt3bb*.*2*) arose from a common ancestor through lineage-specific duplication after TDG^22^. This conclusion, however, took only tetrapod and teleost sequences into account. Thus through the inclusion of the missing link which was previously described as the bridge from tetrapods to teleosts^40^, namely the spotted gar, as well as shark and coelacanth, we clarified and reviewed the phylogenetic relationship between *dnmt3b* paralogs in teleosts. Our results showed that 2 *dnmt3b* genes exist in the genome of gar; one of them (*ex*. *dnmt3bb*.*1*) clustered together on one hand with *dnmt3b* orthologs in shark, coelacanth and tetrapods and one the other hand with teleost orthologs, while the other *dnmt3b* gene in gar clustered with ex- *dnmt3ba* and ex- *dnmt3bb*.*2* in teleosts. We thus hypothesised that the ancestral *dnmt3b* duplicated at VGD2, both duplicates were fixed at least in holeostei, whereas one copy was lost in gnathostomes. Although we cannot exclude the possibility that the *dnmt3ba/bb* might arise from a punctual duplication occurred in ancestral holeostei, we are much in favour of the former hypothesis. In addition, we demonstrated a closer phylogenetic relationship between ex- *dnmt3ba* and ex- *dnmt3bb*.*2* than between these genes and ex- *dnmt3bb*.*1*, which is in accordance with the previous studies^22–24,41^. This is the reason why we proposed new nomenclatures for these genes. Besides, we found two *dnmt3bbb* (ex. *dnmt3bb*.*2*) paralogs (ex. *dnmt3/dnmt3bb*.*2* and ex. *dnmt5/dnmt3bb*.*3*) in zebrafish and another cyprinid fish, indicating that these two genes may arose from a lineage-specific duplication in cyprinid ancestor, as was reported before^22^.

**Figure 6:**
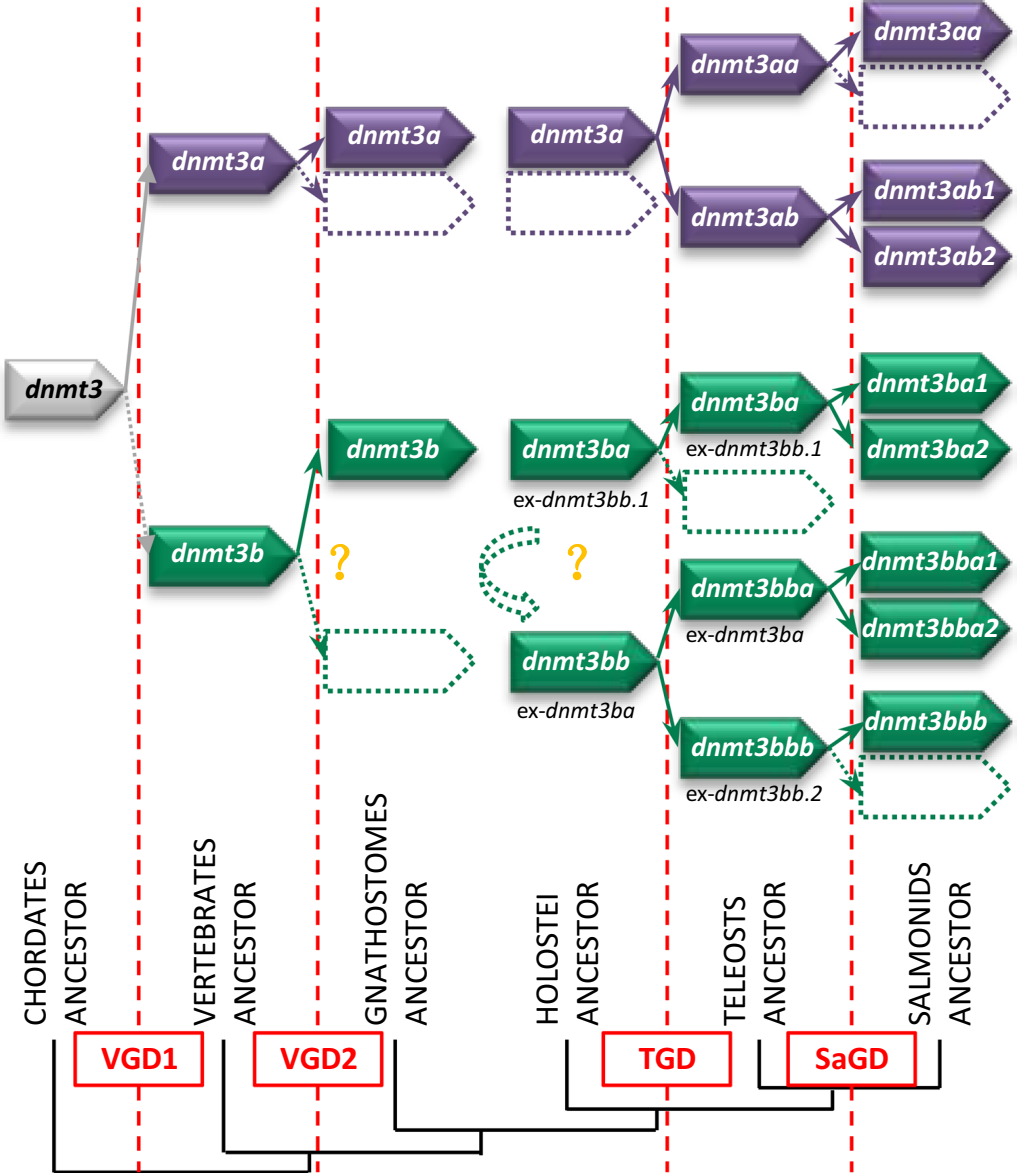
Proposed evolutionary history model of *dnmt3* in chordates.

In summary, we shed new light onto the evolutionary history of complex *dnmt3* among chordates. Based on our findings, we proposed a model for the evolutionary history of these genes in chordates depicted in Fig. 6.

Our findings prove that DNA methylation genes seemed to have conserved their importance during gametogenesis in teleosts as in mammals. However, the increasing number of DNA methylation related genes in teleosts raises the question of their divergence in term of expressional territories and functions.

In the present study, we demonstrated that most of the identified ohnologous/paralogous genes displayed divergent expression pattern during oogenesis or spermatogenesis (*i*.*e* among the dnmt3b encoding genes), suggesting that after duplication these genes encountered a sub- or neo-functionalisation. This hypothesis might be supported by our analyses of the branch test of dN/dS ratio for duplicated *dnmt*, *tet*, and *tdg* genes in trout (Supplemental Fig. S10), which demonstrated that these genes evolved differently. For instance, among the 6 *dnmt3* paralogs in trout, the *dnmt3aa* and *dnmt3bba2* exhibited higher dN/dS ratio compared to the other paralogs, indicating that these genes accumulated more non-synonymous substitutions and evolved faster (Supplemental Fig. S10B).

In addition, results obtained from the present study showed that these duplications also led to new expression patterns compared to what was previously described in other teleosts or mammals. During oogenesis, *dnmt1b* was found maternally expressed in trout oocytes whereas its zebrafish ortholog *dnmt1* was not found in mature oocytes^42^. Moreover trout *tet3a* expression pattern strongly differed from those previously described for the mammalian *tet3*^43^. To date, little is known concerning methylome modification during gametogenesis in fish. Nonetheless, low mRNA levels of *dnmt1b*, *dnmt3ba1*, *dnmt3bba2*, *dnmt3bbb* together with the high mRNA level of *tet3a* in September (late vitellogenesis stage) may possibly coincide with a demethylation event. Additionally, our results highlighted a significant increase in mRNA levels for genes involved in both methylation and demethylation (all *tdg* paralogs) pathways of mature oocytes, indicating that they may be important for early embryonic development before zygotic genome activation.

In terms of spermatogenesis, a previous study in zebrafish reported that the testicular transcription of *dnmt1* and *dnmt3* was correlated with global DNA methylation level^44^. In trout, the higher mRNA levels of *dnmt3ab1* and *dnmt3bba1* observed at the end of spermatogenesis may indicate that a *de novo* methylation event occurred before the release of sperm. Moreover, *tet1* ohnologs were the only tet encoding genes that differentially regulated during spermatogenesis in trout, suggesting an important role of *tet1* genes as was previously proposed in mammals^45^. Overall, our results suggest that late spermatogenesis stage may be as a possible “active phase” for the transcriptional regulation of DNA methylation genes, as most of the affected DNA methylation genes exhibited either their highest (*dnmt1b*, *tdgaa*, and *tdgab*) or lowest (*dnmt3ab1*, *dnmt3bba1* and *tet1b*) mRNA level at this stage.

Concerning early development, we demonstrated that all DNA methylation genes have similar expression pattern with an increase of mRNA levels after the embryonic genome activation (stage 10) and strongly increased from stage 15, which corresponds to the developmental stage of visible optic vesicles^46^. This observation could possibly be explained by the indispensable function of DNA methylation in lens development as was previously described in zebrafish^47^. The peak of expression was observed occurred during the setting up of vital organs (stage 22, 23). This result is in accordance with previous studies, which showed that the expression of DNA methylation genes was largely increased during the organogenesis period in teleosts^18,48–50^. The high expression of DNA methylation related genes at these specific stages maybe explained by the phylotic stage related widespread DNA demethylation of enhancers of regulatory genes during embryogenesis^50^. However this latter publication was conducted on zebrafish, and comparative embryology of 2 different ectotherm species bred at different temperature remained elusive without performing histological analysis.

Looking at the results in detail we showed that some ohnologous/paralogous genes displayed divergent expression patterns at specific stages, suggesting a neo- or sub-functionalisation after duplication. For some of these genes, expression patterns diverged from what was previously described in other species, which is in accordance with previous studies, indicating that DNA methylation related genes could be differentially regulated from species to species^22–24,48^. Indeed, in contrast to what was previously described in zebrafish and sole, we did not observe a peak in the expression of *dnmt3b* paralogs during the blastula or gastrula stages^22,23,51^, which were in favour of neo-functionalisation of these genes in trout during development.

On the other hand, early embryonic expression of *dnmt1* was in accordance with previous studies in other vertebrate species^23,52–54^, suggesting that the vital function of methylation maintenance during this period was conserved during evolution. Besides, the high mRNA level of *dnmt3aa* in hatched alevins as was previously showed in several teleosts^22,23,48^ may indicate a special role of this gene in alevins compared to the other DNA methylation genes.

## Conclusion

The present study is the first to analyse the evolutionary history of DNA methylation genes (*dnmt*, *tet* and *tdg*) in chordates. Results showed that while *dnmt1*, *tet1*, *tet2*, and *tet3* remain as a single copy gene in the genomes of most species of chordates, *dnmt3* and *tdg* duplicates were preferentially fixed in genomes after successive WGD events, increasing their copy numbers during evolution. The case study for trout revealed that these duplicated genes were regulated independently during gametogenesis and embryogenesis, suggesting that they may have suffered sub- or neo-functionalisation after WDG.

## Methods

### Ethical issues and approval

Investigations were conducted according to the guiding principles for the use and care of laboratory animals and in compliance with French and European regulations on animal welfare (Decree 2001-464, 29 May 2001 and Directive 2010/63/EU, respectively). This protocol and the project as a whole were approved by the French National Consultative Ethics Committee (201610061056842).

### *In silico* analysis

Paralogs for DNA methylation genes (*dnmt*, *tet* and *tdg*) in trout were identified in in NCBI assembly database (GCF_002163495.1) using the BLAST tool. Orthologous genes of DNA methylation genes and deduced amino acid sequences for other species were identified from the Ensembl genome browser (Ensembl release 91, December 2017, http://www.ensembl.org) or NCBI database with BLAST tool. The corresponding genes in amphioxus, elephant shark were identified in their respective genome database (https://genome.ucsc.edu/) or NCBI database through BLAST searches. SIMRBASE (https://genomes.stowers.org/search/loc), a database which provides preliminary annotations to characterise the latest lamprey assembly (GCA_002833325.1) was also used to verify the results in lamprey. All the BLAST searches were performed using TBLASTN method with default algorithm parameters: expect threshold was set as 10; word size was set as 6; and low complexity regions were filtered. All the identified sequences were subsequently subjected to conserved domain search in NCBI (https://www.ncbi.nlm.nih.gov/Structure/cdd/wrpsb.cgi) to further confirm their identities and check if there is any alteration in conserved functional sites.

Phylogenetic analyses were carried out using MEGA package version 7 software^55^ with the deduced amino acid sequences of DNA methylation genes. All the protein sequences were aligned with the MUSCLE method in MEGA software before constructing phylogenetic tree. Phylogenetic trees of Dnmt1, Tet and Tdg were built using Maximum Likelihood method and the whole protein sequences, whereas the phylogenetic tree of Dnmt3 was constructed using Neighbour-Joining (NJ) method with the protein alignment containing only the conserved functional domains (PWWP, ADD and Dcm). The reliability of the inferred trees was estimated using the bootstrap method with 500 or 1000 replications in Maximum likelihood method and NJ method, respectively. New gene annotations were allocated according to ZFIN Nomenclature guidelines (http://zfin.org/).

Syntenic analyses were performed with the Genomicus software, version 01.01 (www.genomicus.biologie.ens.fr) to confirm identities of the target genes in various vertebrates. The gene loci of DNA methylation genes in lampreys, elephant sharks and trout and their syntenic genes were identified using the BLAST tool against their corresponding assembly databases in NCBI.

In order to have a preliminary idea about the prediction of sub- or neo-functionalisation among multiple paralogues, the ratios between non-synonymous and synonymous differences (dN/dS) were also calculated with the codeml program in the PAML package version 4.9i^56^. The aligned amino acids sequences of *dnmt1*, *dnmt3*, *tet1*, *tet2*, *tet3* and *tdg genes* in selected representative species were used to guide the alignment of the nucleotides sequences by PAL2NAL to get codon alignments of these genes^57^. We further built phylogenetic trees with their respective codon alignment of these genes using Maximum likelihood method. The codon alignments and phylogenetic trees were used as the input file for codeml program. Branch tests with the free-ratios model (Alternative hypothesis: heterogeneity of dN/dS among branches) and model 0 (Null hypothesis: homogeneous dN/dS across all sites and branches) were performed to calculate the dN/dS for each of the branches of the gene tree. The comparison between two models was conducted using a likelihood ratio test (LRT) followed by a chi-squared test of significance^56^.

### Fish and experimental design

For gametogenesis experiment: two year old genitors were obtained and reared at 8 °C in INRA experimental facilities. Ovaries and testes were sampled (6 individuals per time point) during gametogenesis process in February, May, September and November, corresponding to pre-early vitellogenesis/early spermatogenesis, vitellogenesis/mid spermatogenesis, late vitellogenesis/late spermatogenesis, and spawn stage, respectively (Fig. 4A). For ontogenesis experiment: embryos were collected on triplicate spawns intermittently before the fertilisation stage (oocyte) and during embryonic development: stages 5, 6, 7, 8, 10, 12, 15, 22, 23 and 31 (hatching alevins, 384°D (degree days)) according to Vernier^46^. Embryos were directly snap-frozen whereas alevins and fish were sacrificed by terminal anaesthetisation with a benzocaine bath (60 mg·L^−1^) prior to sampling and subsequently frozen in liquid nitrogen. Samples were stored at −80 °C until further analysis (Fig. 5A).

### Total RNA extraction, cDNA synthesis and RT-qPCR analysis

Total RNA, cDNA synthesis and RT-qPCR analysis on whole embryos (30 embryos extracted together per spawn) and whole alevins (6 alevins extracted individually per spawn) was performed as previously described^58^. Data were normalised to the exogenous luciferase transcript abundance in samples diluted at 1:25^59^.

For gonads, total RNA and cDNA synthesis were performed as previously described^59^. However, samples were diluted at 1:40, and the geometric mean of several reference genes: *β-actin*, elongation factor 1α (*ef1α*), 18S ribosomal RNA (*18S rRNA*), 40 S ribosomal protein S6 (*rps16*), and 60 S Ribosomal Protein L27 (*rpl27*) was used as a normalisation for qPCR data during gametogenesis using the E method (Light Cycler software) as previously described^60–62^. The primer sets used for analysis are listed in Supplemental Table S2 (target genes) and Supplemental Table S3 (reference genes).

### Statistical analysis

The qPCR data were presented as heatmap with mean value of each condition using ClustVis (https://biit.cs.ut.ee/clustvis/). Normality of distributions was assessed using the Shapiro-Wilk test using R (v3.4.0)/Rcmdr Package. Data were analysed by a Kruskal-Wallis non-parametric test followed by a Tukey test as *post hoc* analysis.

## Supplementary information

Supplemental information.
