# Supplementary material for: Evolutionary history of DNA methylation related genes in chordates: new insights from multiple whole genome duplications
Source: Sci Rep. 2020 Jan 22;10:970. doi: 10.1038/s41598-020-57753-w (PMC6976628; doi:10.1038/s41598-020-57753-w)
Supplement: Supplementary file 1 — Supplemental information. [file 41598_2020_57753_MOESM1_ESM.pdf]

Evolutionary history of DNA methylation related genes in chordates: new insights from multiple whole genome duplications

Jingwei Liu, Huihua Hu, Stéphane Panserat, Lucie Marandel[§]

Supplementary Information

Supplemental Table S1. Percentage Identity Matrix between tetrapod and lamprey/ciona dnmt3 proteins

	ciona savignyi (ENSCSAVP000000200)	ciona savignyi (ENSCSAVG00000031)	chicken dnmt3b	human dnmt3b	mouse dnmt3b	lamprey (ENSPMAT00000008525)	lamprey (ENSPMAT00000007628)	lamprey (gi|1304463388|)	chicken dnmt3a	human dnmt3a	mouse dnmt3a
ciona savignyi (ENSCSAVP000000200)	100.00										
ciona savignyi (ENSCSAVG00000031)	48.89	100.00									
chicken dnmt3b	41.19	52.43	100.00								
human dnmt3b	41.48	52.06	62.77	100.00							
mouse dnmt3b	42.36	50.94	60.88	84.07	100.00						
lamprey (ENSPMAT00000008525)	40.07	51.24	55.22	55.94	55.25	100.00					
lamprey (ENSPMAT00000007628)	45.91	56.10	61.39	63.61	61.93	67.20	100.00				
lamprey (gi|1304463388|)	49.50	53.56	66.14	70.58	69.58	68.56	72.06	100.00			
chicken dnmt3a	42.58	52.26	47.66	51.71	51.28	64.27	71.94	75.15	100.00		
human dnmt3a	42.83	53.56	48.01	49.82	49.52	64.69	72.49	75.75	83.37	100.00	
mouse dnmt3a	42.67	53.56	47.94	50.12	49.94	64.69	72.49	75.75	84.06	96.15	100.00

Supplemental Table S2. Annotation, location, primer sequences and accession number of DNA methylation toolkit in rainbow trout

Gene	Scaffold No. in Genomicus	Chromosome No. in NCBI	Forward Primer(5'-3')	Reverse Primer (5'-3')	NCBI Accession No.
dnmt1a	scaffold_644	chr.12	AACCGTTTCACAGAGGACACG	GCAGGGGGTGACGATGATC	XM_021557911.1
dnmt1b	scaffold_1433	chr.13	ATACCTCCTTGTCTCTGCCG	CTGCTGAGCTGGTCCTGG	XM_021625236.1
dnmt3aa	scaffold_4646	chr.8	AATTTGAGGCAGCCAGGTTG	CGATCCCCACGGTGATAGAA	XM_021614345.1
dnmt3ab1	scaffold_7288	chr.25	TGGCACCAGAAGAGAAATCCT	CATGACTCCATTCTGCACCTG	XM_021585300.1
dnmt3ab2	scaffold_64	chr.19	GTGTGCGAGGACTCCGTC	CCTAGCCGGGTTGACAATAGAG	XM_021573815.1
dnmt3ba1	scaffold_5272	chr.16	CAAGGGTTTTGGCATTGGAGA	ACCTCAGAGAACTTGCCATCA	XM_021566506.1
dnmt3ba2	scaffold_78452	chr.9	ACAAGGGTTTTGGTATTGGGGA	AGCAGACACCTCAGAGAACTT	XM_021615717.1
dnmt3bba1	scaffold_3846	chr.16	GGCGATGGAACCTTTGACAAG	TGGGCAGGAATGGAGGGATA	XM_021567210.1
dnmt3bba2	scaffold_384	chr.9	GGCGATGGAACCTTTAAGCAA	CGAGGGCACTGTTGTTGATG	XM_021616487.1
dnmt3bbb	scaffold_1281	chr.16	AGGACCATCACCACCAACC	TCTGCTGGCGATTCATGTTCT	XM_021566510.1
tet1a	scaffold_18888 & 2097	chr.1	CAGTGGCCAGACAAGCTCA	GCCTTCGCCCCTGTTATGT	XM_021614130.1
tet1b	scaffold_189 & 12473	chr.23	GGCTGACAAACAAGCTGCAA	CACCCACATTTCCTGGTCTGT	XM_021580846.1
tet2a/b-glob	scaffold_1163	chr.19	GCTCCATCCTCATCGAGTGT	TCCTTCTCCCGTTGCTTCTC	XM_021573348.1 &XM_021573351.1
tet2c	/	chr.10	AAGAAGCTGAAATCCCCTGGG	CTGCTGCTGTGTCTCCATGAT	XM_021617281.1
tet3a	scaffold_21498 & 36080	chr.6	CAGCACTGCCCTATAGCTACT	GAGTAGGAGTGAGCCAGCC	XM_021606330.1
tet3b	scaffold_605	chr.11	GGCAGGTACACAGTCTCCAA	GGGCACACTCGATCAGGATA	XM_021621924.1
tdgaa	scaffold2682	chr.4	GCAGCCACACAAGATACCTG	ACTCCTGAATGTCGTCCGTT	XM_021598712.1
tdgab	scaffold_82	chr.2	GTCAAGAAGAAAAGGGACCGC	GAATCCGGACAAGAACAGGC	XM_021566210.1
tdgba	scaffold_2682	chr.4	CGTTTGCAGACCAGATACCG	ATGGGCCATTTCTCCTCCAT	XM_021598711.1
tdgbb	scaffold_82	chr.2	CATAACACAGGTCATATGGGC	CTCTCTTTCTCTTCCCTGGCA	XM_021566172.1

Supplemental Table S3. Primer sequences of reference genes

Reference gene	Forward Primer(5'-3')	Reverse Primer (5'-3')
luciferase	CATTCTTCGCCAAAAGCACTCTG	AGCCCATATCCTTGTCGTATCCC
β-actin	GATGGGCCAGAAAGACAGCTA	TCGTCCCAGTTGGTGACGAT
ef1α	TCCTCTTGGTCGTTTCGCTG	ACCCGAGGGACATCCTGTG
18s rRNA	CGGAGGTTCGAAGACGATCA	TCGCTAGTTGGCATCGTTTAT
rps16	TTTCAGGTGGCGAAACATGC	GGGGTCTGCCATTCACCTTG
rpl27	CACAACCATGGGCAAGAAGA	TCAGGGCAGGGTCTCTGAAG

Supplemental Table S4. The mRNA levels of DNA methylation genes during oogenesis in trout

Gene	Oogenesis								p value
	Feb.		May		Sep.		Nov.		
	Mean	SD	Mean	SD	Mean	SD	Mean	SD	
dnmt1a	2.81	2.04	1.19	1.42	0.35	0.43	3.54	4.70	0.109
dnmt1b	1.86[b]	0.19	1.65[b]	0.35	0.79[a]	0.23	4.21[c]	0.75	< 0.001
dnmt3aa	1.24	0.77	1.51	0.47	1.05	0.29	0.88	0.48	0.173
dnmt3ab1	0.55	0.18	0.62	0.22	0.88	0.25	1.97	4.46	0.043
dnmt3ab2	1.27	0.39	1.12	0.23	0.83	0.15	1.30	0.93	0.093
dnmt3ba1	1.42[b]	0.33	1.38[b]	0.13	0.63[a]	0.16	4.60[c]	1.48	< 0.001
dnmt3ba2	0.76[b]	0.14	0.96[b]	0.16	0.40[a]	0.09	0.29[a]	0.14	< 0.001
dnmt3bba1	0.60	0.31	0.52	0.33	0.52	0.33	0.84	0.71	0.749
dnmt3bba2	2.09[b]	0.06	1.72[b]	0.30	0.74[a]	0.24	3.39[c]	0.50	< 0.001
dnmt3bbb	1.87[b]	0.32	1.77[b]	0.43	0.67[a]	0.20	5.54[b]	1.78	< 0.001
tet1a	0.56	0.14	0.61	0.13	0.72	0.11	0.56	0.21	0.139
tet1b	0.85	0.27	0.70	0.20	0.98	0.27	0.54	0.55	0.108
tet2a&b	0.83[a]	0.14	1.47[b]	0.44	1.74[b]	0.28	1.70[ab]	2.87	0.010
tet2c	0.40	0.47	0.55	0.62	1.12	1.32	4.38	6.69	0.609
tet3a	0.70[ab]	0.14	0.82[bc]	0.17	1.52[c]	0.38	0.27[a]	0.18	< 0.001
tet3b	0.92	0.15	1.16	0.24	0.81	0.12	1.77	0.72	0.004
tdgaa	1.69[b]	0.26	1.46[b]	0.28	0.65[a]	0.12	6.55[c]	0.90	< 0.001
tdgab	1.51[ab]	0.51	1.22[b]	0.21	0.77[a]	0.13	5.75[c]	2.00	< 0.001
tdgba	1.24[b]	0.16	1.43[b]	0.43	0.66[a]	0.17	7.16[c]	2.33	< 0.001
tdgbb	1.88[b]	0.44	1.76[b]	0.21	0.74[a]	0.15	10.65[c]	2.98	< 0.001

Supplemental Table S5. The mRNA levels of DNA methylation genes during spermatogenesis in trout

Gene	Feb.		May		Sep.		Nov.		*p* value
	Mean	SD	Mean	SD	Mean	SD	Mean	SD	
dnmt1a	0.39	0.61	0.07	0.10	1.23	1.46	0.18	0.19	0.354
dnmt1b	0.76[ab]	0.86	0.16[a]	0.03	1.04[b]	0.39	0.26[a]	0.07	0.007
dnmt3aa	0.68	0.29	0.69	0.48	0.50	0.22	0.54	0.22	0.594
dnmt3ab1	1.87[ab]	1.47	2.71[b]	1.17	0.50[a]	0.16	2.13[b]	0.95	0.010
dnmt3ab2	0.60	0.26	0.58	0.34	0.42	0.13	0.80	0.23	0.053
dnmt3ba1	0.69	0.52	0.61	0.24	0.41	0.35	1.53	0.73	0.005
dnmt3ba2	1.46	1.26	0.71	0.42	0.87	0.71	2.54	1.17	0.033
dnmt3bba1	1.86[ab]	1.61	1.36[ab]	1.15	0.28[a]	0.25	2.81[b]	0.59	0.007
dnmt3bba2	0.15	0.07	0.24	0.08	0.24	0.04	0.30	0.14	0.059
dnmt3bbb	0.01	0.01	0.01	0.00	0.00	0.00	0.01	0.00	0.030
tet1a	1.04[ab]	0.46	1.63[ab]	0.99	0.75[a]	0.10	2.20[b]	0.72	0.013
tet1b	1.12[ab]	0.79	1.35[ab]	0.66	0.37[a]	0.20	1.67[b]	0.46	0.018
tet2a&b	0.76	0.64	0.98	0.69	0.28	0.13	0.82	0.27	0.101
tet2c	1.92	3.30	0.82	1.15	0.26	0.20	0.67	0.58	0.604
tet3a	0.64	0.33	1.48	1.21	0.34	0.15	1.42	0.59	0.018
tet3b	0.24	0.13	0.49	0.39	0.10	0.06	0.38	0.20	0.040
tdgaa	0.16[ab]	0.11	0.10[ab]	0.10	0.38[b]	0.13	0.06[a]	0.02	0.006
tdgab	0.81[ab]	0.64	0.31a	0.14	1.48[b]	0.41	0.31[a]	0.08	0.009
tdgba	0.20	0.11	0.16	0.04	0.33	0.12	0.20	0.09	0.102
tdgbb	0.12	0.03	0.09	0.03	0.15	0.05	0.17	0.04	0.013

Supplemental Table S6. The mRNA levels of DNA methylation genes during ontogenesis in trout

Gene	oocyte		stage5		stage6		stage7		stage8		stage10		stage12		stage15		stage22		stage23		stage31		*p* value
	Mean	SD	Mean	SD	Mean	SD	Mean	SD	Mean	SD	Mean	SD	Mean	SD	Mean	SD	Mean	SD	Mean	SD	Mean	SD	
dnmt1a	1.28^{abc}	2.22	0.15^{a}	0.16	0.46^{ab}	0.50	0.40^{ab}	0.59	0.13^{a}	0.12	0.38^{ab}	0.48	0.89^{bc}	0.58	1.59^{c}	0.31	17.82^{de}	5.50	23.12^{e}	4.97	4.56^{abcd}	5.68	3.9E-09
dnmt1b	2.00^{b}	0.80	3.51^{c}	0.22	2.24^{bc}	1.42	1.58^{ab}	0.93	1.31^{ab}	1.24	0.49^{a}	0.25	1.10^{ab}	1.06	1.83^{ab}	0.92	12.34^{de}	7.57	19.02^{e}	4.84	2.85^{bcd}	2.19	1.2E-09
dnmt3aa	0.04^{ab}	0.02	0.04^{ab}	0.04	0.02^{ab}	0.02	0.01^{a}	0.01	0.03^{ab}	0.05	0.02^{a}	0.01	0.04^{ab}	0.05	0.08^{b}	0.05	1.70^{c}	1.11	2.84^{c}	0.77	2.81^{c}	2.08	4.5E-05
dnmt3ab1	0.31^{a}	0.04	0.27^{a}	0.09	0.14^{a}	0.05	0.17^{a}	0.08	0.17^{b}	0.15	0.18^{b}	0.07	1.06^{c}	0.99	2.00^{c}	0.97	10.49^{d}	7.12	16.41^{d}	4.92	7.77^{cd}	6.40	2.3E-05
dnmt3ab2	0.03^{ab}	0.01	0.15^{b}	0.07	0.06^{ab}	0.08	0.04^{a}	0.03	0.08^{ab}	0.09	0.10^{ab}	0.02	0.75^{bc}	0.72	1.55^{c}	0.68	12.52^{d}	8.61	22.46^{d}	5.83	8.55^{d}	6.91	1.0E-05
dnmt3ba1	0.18^{b}	0.03	0.28^{c}	0.05	0.22^{abcd}	0.18	0.08^{a}	0.05	0.18^{abcd}	0.20	0.09^{ab}	0.05	0.25^{bc}	0.13	0.61^{de}	0.22	6.11^{f}	4.69	8.90^{f}	1.97	2.68^{ef}	2.29	1.5E-05
dnmt3ba2	0.01^{a}	0.01	0.00^{a}	0.00	0.00^{a}	0.01	0.00^{a}	0.00	0.02^{a}	0.01	0.04^{b}	0.01	0.51^{c}	0.48	1.80^{c}	1.10	12.73^{de}	9.44	18.62^{e}	1.55	2.96^{cd}	2.49	1.4E-05
dnmt3bba1	0.03^{ab}	0.02	0.06^{b}	0.01	0.02^{a}	0.02	0.03^{ab}	0.03	0.02^{a}	0.01	0.03^{a}	0.01	0.92^{c}	0.78	2.46^{c}	1.38	20.49^{d}	15.13	26.99^{d}	7.90	3.12^{c}	2.23	1.4E-05
dnmt3bba2	0.84^{ab}	0.38	1.25^{b}	0.26	0.63^{ab}	0.45	0.55^{a}	0.35	0.77^{ab}	0.73	0.78^{ab}	0.33	5.46^{bc}	5.18	5.60^{c}	2.92	16.00^{cd}	10.87	20.80^{d}	3.96	3.15^{bc}	2.39	3.7E-04
dnmt3bbb	4.08^{ab}	2.87	7.28^{bc}	1.83	3.29^{ab}	2.52	3.20^{ab}	2.44	4.76^{abc}	4.83	2.90^{a}	1.52	5.17^{abc}	5.51	4.40^{ab}	2.59	14.60^{cd}	10.13	19.31^{d}	4.01	2.15^{a}	1.59	1.5E-02
tet1a	0.02^{a}	0.01	0.02^{a}	0.01	0.01^{a}	0.01	0.01^{a}	0.01	0.02^{a}	0.02	0.03^{a}	0.01	0.16^{b}	0.13	0.37^{b}	0.14	5.34^{cd}	3.31	7.98^{d}	1.80	2.26^{c}	1.74	7.6E-06
tet1b	0.02^{ab}	0.01	0.02^{ab}	0.01	0.01^{a}	0.01	0.01^{ab}	0.01	0.02^{ab}	0.01	0.02^{b}	0.00	0.10^{c}	0.09	0.30^{c}	0.17	5.07^{d}	3.10	8.40^{d}	1.96	2.66^{d}	2.05	8.2E-06
tet2a&b	0.01^{ab}	0.02	0.00^{a}	0.00	0.00^{a}	0.01	0.01^{ab}	0.01	0.02^{b}	0.00	0.05^{c}	0.01	0.24^{d}	0.09	0.48^{d}	0.15	6.59^{ef}	4.88	11.28^{f}	2.75	2.88^{e}	2.21	5.9E-06
tet2c	0.02^{abc}	0.03	0.00^{a}	0.00	0.00^{a}	0.00	0.01^{ab}	0.02	0.01^{ab}	0.02	0.08^{abc}	0.05	0.11^{abc}	0.12	0.54^{c}	0.57	10.14^{d}	9.69	5.97^{d}	6.05	4.08^{bcd}	6.08	1.9E-02
tet3a	0.01^{a}	0.00	0.01^{a}	0.01	0.01^{a}	0.01	0.01^{a}	0.01	0.02^{ab}	0.03	0.01^{a}	0.01	0.08^{b}	0.03	0.20^{c}	0.08	6.63^{d}	4.73	10.00^{d}	2.68	3.02^{d}	2.40	1.2E-05
tet3b	0.17^{a}	0.09	0.21^{a}	0.04	0.19^{a}	0.15	0.11^{a}	0.09	0.38^{ab}	0.30	0.17^{a}	0.10	0.83^{bc}	0.27	1.64^{d}	0.57	9.68e^{ef}	6.34	16.48^{f}	6.22	3.99^{cde}	3.17	1.9E-05
tdgaa	0.79^{bc}	0.23	1.13^{c}	0.14	0.71^{abc}	0.67	0.54^{ab}	0.32	0.80^{abc}	0.91	0.35^{a}	0.07	0.61^{abc}	0.64	1.32^{bc}	0.72	11.03^{de}	7.63	15.22^{e}	2.71	2.58^{bcd}	2.00	1.2E-03
tdgab	0.32^{bc}	0.13	0.41^{c}	0.02	0.27^{abc}	0.24	0.20^{ab}	0.12	0.25^{abc}	0.24	0.11^{a}	0.01	0.74^{bcd}	0.66	1.83^{d}	1.04	14.45^{ef}	8.98	18.14^{f}	2.59	2.71^{de}	2.18	2.6E-05
tdgba	1.12^{b}	0.56	1.31^{b}	0.51	0.90^{ab}	0.68	0.31^{a}	0.15	0.77^{ab}	0.86	0.39^{a}	0.02	0.45^{ab}	0.34	1.65^{bc}	1.04	4.50^{cd}	3.10	5.39^{d}	2.83	1.15^{abc}	0.90	1.6E-02
tdgbb	1.82^{bc}	0.70	2.27^{c}	0.37	1.43^{abc}	1.14	1.07^{ab}	0.63	1.38^{abc}	1.40	0.57^{a}	0.02	0.89^{ab}	0.80	1.21^{abc}	0.71	6.73^{de}	4.57	10.19^{e}	2.58	2.19^{abcd}	1.72	1.9E-02

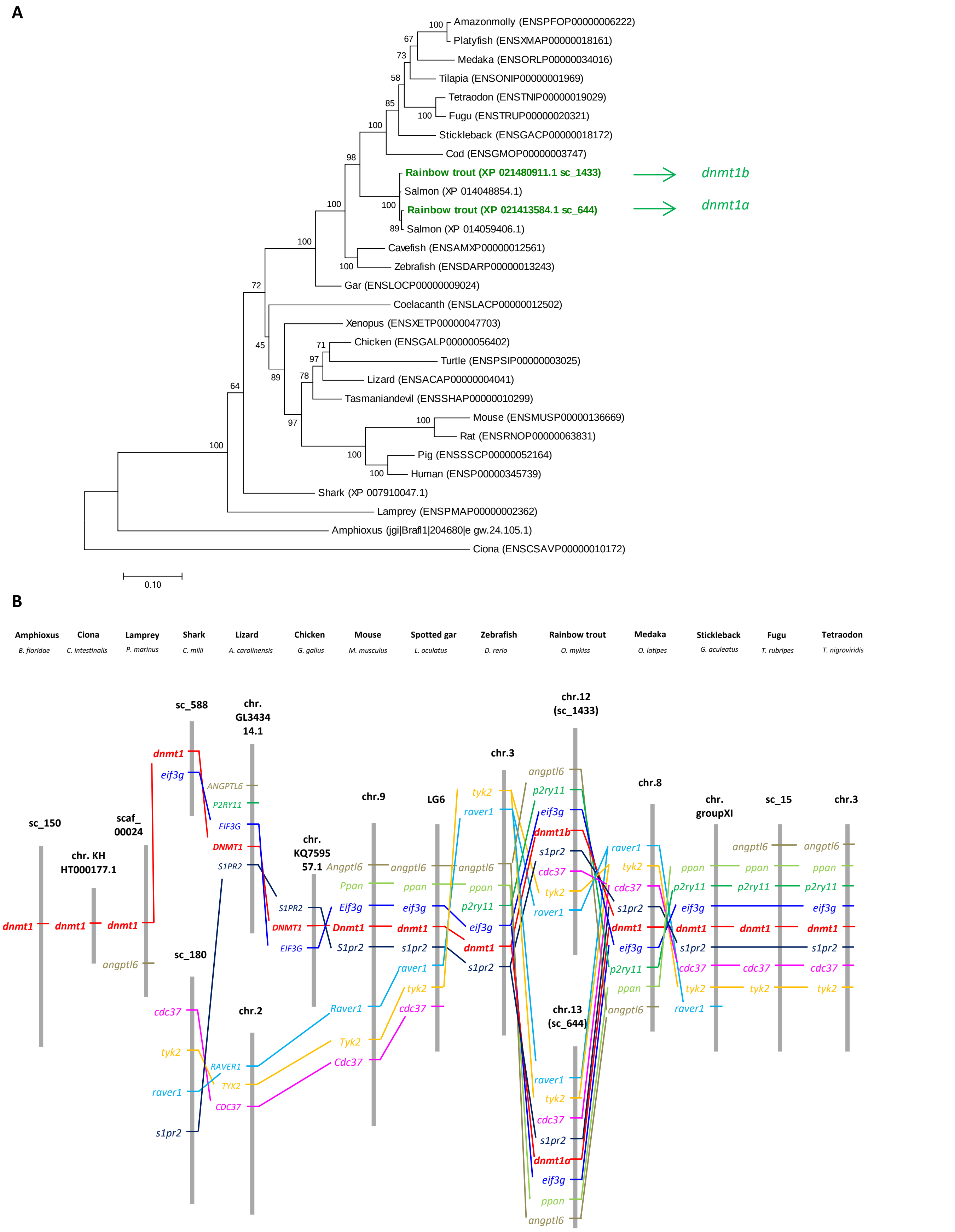

Supplementary Fig. S1 ***In silico*** **analysis of dnmt1 in chrodates.** (**A**) Pylogenetic of *dnmt1* genes. The phylogenetic trees were built using the Maximum Likelihood method based on the JTT matrix-based model in MEGA7. The tree with the highest log likelihood (-17912.17) is shown. A discrete Gamma distribution was used to model evolutionary rate differences among sites (5 categories (+G, parameter = 0.6612)). The reliability of the inferred trees was estimated by the bootstrap method with 500 replications. All accession numbers are specified in parentheses. (**B**) Syntenic analysis of *dnmt1* genes. Conserved synteny around the *dnmt1* loci in chrodates. Data were collected with Genomicus software version 01.01, and *dnmt1* genes in rainbow trout were annotated by ourselves according to our phylogenetic analysis following ZFIN nomenclature guidelines. chr., chromosome; sc., scaffold.

```
                                      10        20        30        40        50        60        70        80
                                ....|....|....|....|....|....|....|....|....|....|....|....|....|....|....|....|
Tetraodon(ENSTNIP00000019029)   --------------------------------------------------------------------------------
Fugu(ENSTRUP00000020321)        --------------------------------------------------------------MPTRTSLSL---------
Tilapia(ENSONIP00000001969)     --------------------------------------------------------------MPTRTSLPL---------
Medaka(ENSORLP00000034016)      --------------------------------------------------------------MPSRTSLSL---------
Amazon molly(ENSPFOP0000000622  --------------------------------------------------------------MPSKTSLSL---------
Platyfish(ENSXMAP00000018161 )  --------------------------------------------------------------MPSKTSLSL---------
Stickleback(ENSGACP00000018172  -----------------------------------------------------------PFVMPSKTSLSL---------
Cod(ENSGMOP00000003747)         -----------------------------------------------------------LFTMPTKTSLSL---------
Salmon1b (XP_014048854.1)       --------------------------------------------------------------MPANTSLSL---------
Salmon1a(XP_014059406.1)        --------------------------------------------------------------MPANTSLSL---------
Trout1b(XP_021480911.1)         --------------------------------------------------------------MPANTSLSL---------
Trout1a(XP_021413584.1)         --------------------------------------------------------------MPANTSLFL---------
Cavefish(ENSAMXP00000012561)    --------------------------------------------------------------MPAKTSLSL---------
Zebrafish(ENSDARP00000013243)   --------------------------------------------------------------MPTKTSLSL---------
Gar(ENSLOCP00000009024)         --------------------------------------------------------------MPAKTSLSL---------
Shark(XP_007910047.1)           MGDEVKKMPAQTVVKNMEWSGPEGKTLIQPTPCSPQHVPASSHSPSTTNPYLHHSPTSAPPTCPYRSPFALYLTHITLPT
Coelacanth(ENSLACP00000012502)  --------------------------------------------------------------------------------
Xenopus(ENSXETP00000047703)     -------------------------------------------------LTESVSPLLYTAMPAQSASLAL---------
Chicken(ENSGALP00000056402)     --------------------------------------------------------------------------------
Turtle(ENSPSIP00000003025)      --------------------------------------------------------------LPAXMAGGS---------
Lizard(ENSACAP00000004041)      ------------------MVPSSAGPAPPSSPSPRCARNCSRPPSRSLPTAASLPVCLAGPEMPARAAPLT---------
Tasmanian devil(ENSSHAP0000001  ------------------------------------------------------LPATRPQLNPYFTRFSL---------
Mouse(ENSMUSP00000136669)       ------------------------------------------------------------MADSNRSPRSR---------
Rat(ENSRNOP00000063831)         --------------------------------------------------------------MPARTAPARVPALASPAG
Pig(ENSSSCP00000052164)         -----------------------------------------------------------MAASRDRRRRLG---------
Human(ENSP00000345739)          --------------------------------------------------------------MPARTAPARVPTLAVPAI
Lamprey(ENSPMAP00000002362)     ---------------------------------------------------------------GHSTWVKL---------
Ciona(ENSCSAVP00000010172)      --------------------------------------------------------------------------------
Amphioxus(jgi|Brafl1|204680|e_  --------------------------------------------------------------------------------

                                      90       100       110       120       130       140       150       160
                                ....|....|....|....|....|....|....|....|....|....|....|....|....|....|....|....|
Tetraodon(ENSTNIP00000019029)   --------------------------------------------------------------------------------
Fugu(ENSTRUP00000020321)        --------------------------------------------------------------------------------
Tilapia(ENSONIP00000001969)     --------------------------------------------------------------------------------
Medaka(ENSORLP00000034016)      --------------------------------------------------------------------------------
Amazon molly(ENSPFOP0000000622  --------------------------------------------------------------------------------
Platyfish(ENSXMAP00000018161 )  --------------------------------------------------------------------------------
Stickleback(ENSGACP00000018172  --------------------------------------------------------------------------------
Cod(ENSGMOP00000003747)         --------------------------------------------------------------------------------
Salmon1b (XP_014048854.1)       --------------------------------------------------------------------------------
Salmon1a(XP_014059406.1)        --------------------------------------------------------------------------------
Trout1b(XP_021480911.1)         --------------------------------------------------------------------------------
Trout1a(XP_021413584.1)         --------------------------------------------------------------------------------
Cavefish(ENSAMXP00000012561)    --------------------------------------------------------------------------------
Zebrafish(ENSDARP00000013243)   --------------------------------------------------------------------------------
Gar(ENSLOCP00000009024)         --------------------------------------------------------------------------------
Shark(XP_007910047.1)           LVPHCSSTSPPASPSPHSFPPAPPPTQLVLLLYLNPPFATPVCPLPPVPACSVLSPHTHLPPPTLPLQPTCTALPHPLPQ
Coelacanth(ENSLACP00000012502)  --------------------------------------------------------------------------------
Xenopus(ENSXETP00000047703)     --------------------------------------------------------------------------------
Chicken(ENSGALP00000056402)     --------------------------------------------------------------------------------
Turtle(ENSPSIP00000003025)      --------------------------------------------------------------------------------
Lizard(ENSACAP00000004041)      --------------------------------------------------------------------------------
Tasmanian devil(ENSSHAP0000001  --------------------------------------------------------------------------------
Mouse(ENSMUSP00000136669)       --------------------------------------------------------------------------------
Rat(ENSRNOP00000063831)         SL------------------------------------------------------------------------------
Pig(ENSSSCP00000052164)         --------------------------------------------------------------------------------
Human(ENSP00000345739)          SL------------------------------------------------------------------------------
Lamprey(ENSPMAP00000002362)     --------------------------------------------------------------------------------
Ciona(ENSCSAVP00000010172)      --------------------------------------------------------------------------------
Amphioxus(jgi|Brafl1|204680|e_  --------------------------------------------------------------------------------

                                                                        DMAP domain
                                                                    <-------------------------------------------->
                                     170       180       190       200       210       220       230       240
                                ....|....|....|....|....|....|....|....|....|....|....|....|....|....|....|....|
Tetraodon(ENSTNIP00000019029)   --------------------------------------------------------------------------------
Fugu(ENSTRUP00000020321)        ------------------------------------PED-----------------------------------------
Tilapia(ENSONIP00000001969)     ------------------------------------PDD-----------------------------------------
Medaka(ENSORLP00000034016)      ------------------------------------PDD-----------------------------------------
Amazon molly(ENSPFOP0000000622  ------------------------------------PDD-----------------------------------------
Platyfish(ENSXMAP00000018161 )  ------------------------------------PDD-----------------------------------------
Stickleback(ENSGACP00000018172  ------------------------------------PED-----------------------------------------
Cod(ENSGMOP00000003747)         ------------------------------------PED-----------------------------------------
Salmon1b (XP_014048854.1)       ------------------------------------PVD-----------------------------------------
Salmon1a(XP_014059406.1)        ------------------------------------PVD-----------------------------------------
Trout1b(XP_021480911.1)         ------------------------------------PVD-----------------------------------------
Trout1a(XP_021413584.1)         ------------------------------------PVD-----------------------------------------
Cavefish(ENSAMXP00000012561)    ------------------------------------PED-----------------------------------------
Zebrafish(ENSDARP00000013243)   ------------------------------------PED-----------------------------------------
Gar(ENSLOCP00000009024)         ------------------------------------PED-----------------------------------------
Shark(XP_007910047.1)           LYPRECLSPASTCSHSQLNFLSWGKGGSQSAIVSGAPLQSGLFKDRVWKVSLIFLVAYSGTLCLCALYVKCLFQTTFYTV
Coelacanth(ENSLACP00000012502)  --------------------------------------------------------------------------------
Xenopus(ENSXETP00000047703)     ------------------------------------PAD-----------------------------------------
Chicken(ENSGALP00000056402)     --------------------------------------------------------------------------------
Turtle(ENSPSIP00000003025)      ------------------------------------SADKPRFS------------------------------------
Lizard(ENSACAP00000004041)      ------------------------------------PEE-----------------------------------------
Tasmanian devil(ENSSHAP0000001  ------------------------------------NLDLN---------------------------------------
```

```
Mouse(ENSMUSP00000136669)        ------------------------------------PKP-----------------------------------------
Rat(ENSRNOP00000063831)          ------------------------------------PDH-----------------------------------------
Pig(ENSSSCP00000052164)          ------------------------------------PGR-----------------------------------------
Human(ENSP00000345739)           ------------------------------------PDD-----------------------------------------
Lamprey(ENSPMAP00000002362)      ------------------------------------PLT-----------------------------------------
Ciona(ENSCSAVP00000010172)       --------------------------------------------------------------------------------
Amphioxus(jgi|Brafl1|204680|e_   --------------------------------------------------------------------------------
```

DMAP domain

```
                                          250       260       270       280       290       300       310       320
                                 ....|....|....|....|....|....|....|....|....|....|....|....|....|....|....|....|
Tetraodon(ENSTNIP00000019029)    --------------------------------------------------------------------------------
Fugu(ENSTRUP00000020321)         ---VKKRLQVL----DEDGSTEEDHVKEKLKLVQKFLHIDAQDQLTSLEESMKNSEISMEVYISKVKA------LLGKEL
Tilapia(ENSONIP00000001969)      ---VRKSLQRL----DEEGSAEEEHVKEKLKLVQDFLHADAQDQLTSLEEKMKSSEISKEVYISKVKA------LLGKEL
Medaka(ENSORLP00000034016)       ---VRKRLQML----EEDGSSEEDQVKEKLKLVQDFLHDDTQTQLTSLEKEMKSSEISKEVYISKVKA------LLGKEL
Amazon molly(ENSPFOP0000000622   ---VRKRLQML----DEDGSPEEDQVKEKLRLVQDFLHDDAQDQLTNLEEKMKNSEISKEVYISKVKV------LLGKEL
Platyfish(ENSXMAP00000018161 )   ---VRKRLQKL----DEDGSPEEDQVKEKLRLVQDFLHDDAQDQLTNLEEKMKNSEISKEVYISKVKV------LLGKEL
Stickleback(ENSGACP00000018172   ---VRKRLQVL----DEDGSSGEDHVKEKLKLVQNFLHVDAQDQLNSLEEKMKSSEMSMDVYLSKVKA------LLGKEL
Cod(ENSGMOP00000003747)          ---VRK-----------------DRVKEKLKLVQEFLHDDAQDQLSSLKEKMKSSEISVEGYLSKVKA------LLVREL
Salmon1b (XP_014048854.1)        ---VRKRLAVLEK--DEDGISDEDLVKEKVKLVQDFLHADAQDQLSSLKAKMKSSEISMDGYLSKVKS------LLGREL
Salmon1a(XP_014059406.1)         ---VRKRLAVLEK--DEDGISDEDRVKEKVKLVQDFLHADAQDQLSSLKAKMKSSEISMDGYLSKVKA------LLGREL
Trout1b(XP_021480911.1)          ---VRKRLEVLEK--DEDGISDEDLVKEKVELLQDFLHADAQDQLSSLKAKMKSSEISMDGYLSKVKA------LLGREL
Trout1a(XP_021413584.1)          ---VRKRLEVLEK--DEDGISDEDRVKEKVKLVQDFLHPDAQDQLSSLKAKMKSSEISMDGYLSKVKA------LLGREL
Cavefish(ENSAMXP00000012561)     -----------------------ECVKEKLRLLQDFLRDETQDQLKNLEAKLKSSELSTEVYTSEVKA------VLKKAL
Zebrafish(ENSDARP00000013243)    ---VKERLQVLDE--GGDSLSDEECVKEKLRLLQEFLLADTQDQLKNLEDKLKSSELSTEVYMSEVKA------VLKKAL
Gar(ENSLOCP00000009024)          ---VKKRLQVLDT--DGDGLTDEERVRETLSLVLGYLKADAQNQLNNLESKLKKEELSVEGYLSKVKA------LLGREL
Shark(XP_007910047.1)            ALISSHRLQALED--KVDGLTEKECIKEKLSLLHGFLQADVQNNLNELETKFQKDELSEEGYFAKVKV------LLSQEP
Coelacanth(ENSLACP00000012502)   --------------------------------------------------------------------------------
Xenopus(ENSXETP00000047703)      ---VRKRLKDLER--DQDGMTEKECVQQKLSLVLGFLEADARNKLSDLESKLSSEELSEEGYLTKVKA------LLRKQL
Chicken(ENSGALP00000056402)      --------------------------------------MEVEEAAASSSSSSSSSSSSSSSSSSSSSL------------
Turtle(ENSPSIP00000003025)       -----CRLKDLEK--DEEGLSEKEWEKEKLSLVHGFLQADAQNQLSDLETKLRREELAGGGDLPSSVC------LPSVPL
Lizard(ENSACAP00000004041)       ---LRWRLKDLER--NEDGLGERECVKEKLSLMHGFLQADVQNQLKDLETKFHKEELSEERYLAKVKA------LLHKEL
Tasmanian devil(ENSSHAP0000001   ---INIRLKDLEK--DEDILTEKEFVKKKLSLLHEFLQAEVQHQLFDLEIKLHKEELSEEGYLSKVKF------LLNKEL
Mouse(ENSMUSP00000136669)        -----RGPRRS----KSDSDTLFETSPSSVATRRTTRQTTITAHFTKGPTKRKPKEESEEGNSAESAAEERDQDKKRRVV
Rat(ENSRNOP00000063831)          ---VRRRLKDL----ERDGLTEKECVKEKLNLLHEFLQTEIKSQLCDLETKLHKEELSEEGYLAKVKT------LLNKDL
Pig(ENSSSCP00000052164)          ---GRLRLKDL----ERHSLTEKECVKEKLNLLHEFLQTEIKNQLCDLETKLHKEELSEEGYLAKVKS------LLNKDL
Human(ENSP00000345739)           ---VRRRLKDL----ERDSLTEKECVKEKLNLLHEFLQTEIKNQLCDLETKLRKEELSEEGYLAKVKS------LLNKDL
Lamprey(ENSPMAP00000002362)      ---VRKKLAELEERFRTECLTEKGYMKKKLGLVQPFLSGEVKTQLETLQEELKEEDITEKGYLNKVKT------LLLREL
Ciona(ENSCSAVP00000010172)       --------------------------------------------------------------------------------
Amphioxus(jgi|Brafl1|204680|e_   --------------------------------------------------------------------------------
```

DMAP domain

```
                                          330       340       350       360       370       380       390       400
                                 ....|....|....|....|....|....|....|....|....|....|....|....|....|....|....|....|
Tetraodon(ENSTNIP00000019029)    --------------------------------------------------------------------------------
Fugu(ENSTRUP00000020321)         HLENGN--------------SNGYT-NGSH------------KDEDGEEASMDVQ-EGEETMKS------PTAS--KGKG
Tilapia(ENSONIP00000001969)      HLENGSHVDDAEK----NGKTNGFS-NGSH------------KDEHDEDVTMSVQEEEEESFKS------PTAS--KGKG
Medaka(ENSORLP00000034016)       HLENDLEV----------GKTNGFS-NGSH------------EDEQDEEA------MGTDDVKS------PPPS--KGKA
Amazon molly(ENSPFOP0000000622   RLENGSHEDGVEQ----NGKANGFA-NGSH------------KDKEDEDVNMVTEREETET-KS------PAAL--KGKG
Platyfish(ENSXMAP00000018161 )   RLENGSHEDGVEQ----NGKANGFV-NGSH------------KDKEDEDVNMATEQEETET-KS------PPAL--KGKG
Stickleback(ENSGACP00000018172   NHENGSHVDSVEH----NGKT--VLSNGSH------------KDEHSEDVTME---EEEEAVKS------PTAS--KGKG
Cod(ENSGMOP00000003747)          S-TNGEGVNG--Q----SGKTNGLATNGSH------------KGEEDAEVTMDTQ-EEAAPLKS------PTAA--KGKN
Salmon1b (XP_014048854.1)        CVENGSHGDVVEQNCK-NGKTNGSAANGDS------------HKEEDEDGIMDT--KEADAMKS------PSAP--KGKG
Salmon1a(XP_014059406.1)         CVENGSHGDVVEQNCK-NGKTNGSAANGDY------------HKEEDEEGIMDT--KEADVMKS------PSAP--KGKG
Trout1b(XP_021480911.1)          CVENGSHGDVVEQNCK-NGKTNGSAANGDS------------HKEED-DGIMDT--KEADAMKS------PSAP--KGKG
Trout1a(XP_021413584.1)          CVENGSHGDVVAQNCK-NGKTNGSAANGDS------------HKKEDEEVIMDT--KEADAMKS------PSAP--KGKG
Cavefish(ENSAMXP00000012561)     ALGKDVQSDGVEQ----NGHSNGISENGSH------------KGNGEKEGDMDAE-EEGDAIKS------PSTT--KGKG
Zebrafish(ENSDARP00000013243)    GVVKEGDGVEQ------NGHSNGFSENGSH------------KDNGEQEGAMDTQ-EEGDAIKS------PSAP--KGRG
Gar(ENSLOCP00000009024)          CLENGSSEL--------NGQANGCAENGAH---GASDEDCGPRNGQGAEGTVEA--REEEGLKS------PSTG--KPRG
Shark(XP_007910047.1)            PVENGDSEL--------GPNANGCAENGAR------SGKKDDDRDKANGVAGERNGQEQNLMETGGEDPTPQAA--KSRG
Coelacanth(ENSLACP00000012502)   --------------------------------------------------------------------------------
Xenopus(ENSXETP00000047703)      SCENGDLSL--------NGETNGCSTNGTC---------------GSDEEDVEMSESNTAGVKN--------------RK
Chicken(ENSGALP00000056402)      ----------------------------------------------------------------------LPAP--RARK
Turtle(ENSPSIP00000003025)       NAAGTSGAG--------ALAAEGCSAPGTGAVWGVGGGQEQRREASGTASFLTSARAQGQARRATVSAVPPPSR--STLL
Lizard(ENSACAP00000004041)       SFENGDNLEL-------GQKTNGCAENCAN-------GSDEEPERMGNKTEEDSGMEIEEAASSSSSTPLASPSVLKARK
Tasmanian devil(ENSSHAP0000001   FLENGDHPL--------TSRVNGCLENGIY----------TSDEDFEKSVEEDSTVEMEEATGS------STSLS-KTRK
Mouse(ENSMUSP00000136669)        DTESGAAAAVEKLEEVTAGTQLGPEEPCEQ---------------EDDNRSLRRHTRELSLRRK------SKED--PDRE
Rat(ENSRNOP00000063831)          CLENGTLSL--------TQKANGCPANGSR---------------------PTWKAEMADSNRS------PRSRP-KPRG
Pig(ENSSSCP00000052164)          SLENGAHAF--------SREVNGYLENGSQ------------------TSGEDRRVEMAEENKS------PKPVS-KLGP
Human(ENSP00000345739)           SLENGAHAY--------NREVNGRLENGNQ-------------------ARSEARRVGMADANS------PPKPLSKPRT
Lamprey(ENSPMAP00000002362)      PKLEEEGSE--------KPHQNGAAKNGKA------NGDEAATENGDAHPAMANGSPECSELAS------P-----RGRG
Ciona(ENSCSAVP00000010172)       --------------------------------------------------------------------------------
Amphioxus(jgi|Brafl1|204680|e_   --------------------------------------------------------------------------------
```

```
                                      410       420       430       440       450       460       470       480
                               ....|....|....|....|....|....|....|....|....|....|....|....|....|....|....|....|
Tetraodon(ENSTNIP00000019029)  ----------------------------------TLFQKL-IKRKSEDLNGDS--TN-----------------------
Fugu(ENSTRUP00000020321)       GRRSKANSDTK--KSPASTRVTRN--SGKQPTIMSMFSKV-QKRKSEDLNGDS--TN-----------------------
Tilapia(ENSONIP00000001969)    GRRSKANSDTK--KSPASTRVTRN--SGKQPTILSMFTKV-QKRKSEDLNGEA--VN-----------------------
Medaka(ENSORLP00000034016)     GRRSKSNGDSK--KSPASARVTRN--SGKQPTILSMFSKV-QKRKSEEMSEES--VN-----------------------
Amazon molly(ENSPFOP0000000622 GRRSKANSETK--KSPTSTRVTRN--SGKQPTILSLFSKV-QKRKSEDLNGEA--VN-----------------------
Platyfish(ENSXMAP00000018161 ) GRRSKANSETK--KSPTSTRVTRN--SGKQPTILSMFSKV-QKRKSEDLNGEA--IN-----------------------
Stickleback(ENSGACP00000018172 GRKSKTNSDNT--KSPASTRVTRN--AGKQPTIMSMFSKVSQKRKSEDLNGDA--AN-----------------------
Cod(ENSGMOP00000003747)        GRKSKSDSD-----SPASARVTRN--SGKQPTIMSMFPKVRQKRKSDEVNGEDSIAN-----------------------
Salmon1b (XP_014048854.1)      GRKSKSDFEPK--KSPGS-RVTRNSVSGKQPTILSMFSKV-QKRKSDEVNGEV--TN-----------------------
Salmon1a(XP_014059406.1)       GRKSKSDSEPK--KSPGS-RVTRNSVSGKQPTILSMFSNV-QKRKSDEVNGEV--TN-----------------------
Trout1b(XP_021480911.1)        GRKIKSDSEPK--KSPGS-RVTRNSVSGKQPTILSMFSKV-QKCKSDEVNGEV--TN-----------------------
Trout1a(XP_021413584.1)        GRKSKSDSEPK--KSPGS-RVTRNSVSGKQPTILSMFSKV-QKRKSDEVNGEV--TN-----------------------
Cavefish(ENSAMXP00000012561)   GRRSKADSEPK--KSPGSSRVTRN--SGKQPTILSMFSRV-PKRKSDEVNGET--TN-----------------------
Zebrafish(ENSDARP00000013243)  GRRSKADSEPK--KSPASSRVTRN--TGKQQTIVSMFSRV-PKRKSDELNGEP--AN-----------------------
Gar(ENSLOCP00000009024)        GRRSKTEGDTK--KSFSSSRVTRN--SVKQPTILSMFAKSSSKRKSDEVNGEE--VA-----------------------
Shark(XP_007910047.1)          KRQSKSNGDNR--RTSGSPRITRNS-MKQQATITSMFAKAANKRKSEELNDEE---------------------------
Coelacanth(ENSLACP00000012502) --------------------------------------------------------------------------------
Xenopus(ENSXETP00000047703)    TRKSKTNGENKT-ESPARARSSRST-TGKQATILSMFSKGSSDLAMHQHSCNITFDNLITSDGIRRRCSIPFQHIMLEYQ
Chicken(ENSGALP00000056402)    ARRSRSNGESK--KSPASSRVTRS--SGRQPTILSVFSKGSTKRKSEEVNGAVKPEV-----------------------
Turtle(ENSPSIP00000003025)     ERRARHAGPRS--QXPASSRVTRS--SGRQPTILALFAKGSNKRKSDEVNGEV--KQ-----------------------
Lizard(ENSACAP00000004041)     GRKSKSNSESR--KTPSSSRVTRS--SGKQPTIVAMFSKGSNKRKSEEVNGKLKQET-----------------------
Tasmanian devil(ENSSHAP0000001 SRRSKSDGEAKT-ESSASSRVTRS--SGKQPTIVSLFSKGSGKRKSEGATGEI--KQ-----------------------
Mouse(ENSMUSP00000136669)      ARPETHLDEDE--DGKKDKRSSRPRSQPRDPAA---------KRRPKEAEPEQVAPE-----------------------
Rat(ENSRNOP00000063831)        PRRSKSDSETMI-EASSSSVATRR--TTRQTTITSHF-KGPAKRKPKEDSEKG--NA-----------------------
Pig(ENSSSCP00000052164)        PRRSKSDGEAKSAEVSSSPRITRQ--TTRQTTITSHFTRGPGKRKPEEDTAKAKPDS-----------------------
Human(ENSP00000345739)         PRRSKSDGEAKP-EPSPSPRITRK--STRQTTITSHFAKGPAKRKPQEESERA--KS-----------------------
Lamprey(ENSPMAP00000002362)    GQRGRKSVGAA---------------GGSQQTIASMFGKGSVKRKSEEANGKV---------------------------
Ciona(ENSCSAVP00000010172)     --------------------------------------------------------------------------------
Amphioxus(jgi|Brafl1|204680|e_ --------------------------------------------------------------------------------

                                      490       500       510       520       530       540       550       560
                               ....|....|....|....|....|....|....|....|....|....|....|....|....|....|....|....|
Tetraodon(ENSTNIP00000019029)  -------------------------------------------G-------QNEDKKEEDNTEE----------------
Fugu(ENSTRUP00000020321)       -------------------------------------------G-------QNEDKKEDDNVEE----------------
Tilapia(ENSONIP00000001969)    -------------------------------------------G-------QNEPKKDDD-VEE----------------
Medaka(ENSORLP00000034016)     -------------------------------------------G-------KDEVKKEED-VEE----------------
Amazon molly(ENSPFOP0000000622 -------------------------------------------G-------INEAQKEDD-AEE----------------
Platyfish(ENSXMAP00000018161 ) -------------------------------------------G-------INEAQKEDY-TEE----------------
Stickleback(ENSGACP00000018172 -------------------------------------------G-------ENEGKKEEDDAEA----------------
Cod(ENSGMOP00000003747)        -------------------------------------------G-------EKEVKKEEEEEED----------------
Salmon1b (XP_014048854.1)      -------------------------------------------GNV-----EEKGEVERDTKEE----------------
Salmon1a(XP_014059406.1)       -------------------------------------------GEVTNR--EVEEKVERDTEEE----------------
Trout1b(XP_021480911.1)        -------------------------------------------GKV-----EEQEKIERDMEEE----------------
Trout1a(XP_021413584.1)        -------------------------------------------GEVTNREVEEKEKVDRDT-EE----------------
Cavefish(ENSAMXP00000012561)   -------------------------------------------GET-----AMETKTEEADVEE----------------
Zebrafish(ENSDARP00000013243)  -------------------------------------------G-------DTEIKTEETITEE----------------
Gar(ENSLOCP00000009024)        -------------------------------------------P-------VTEEGVTDD-EEK----------------
Shark(XP_007910047.1)          ---------------------------------------------------QVPVKKEEESEEQ----------------
Coelacanth(ENSLACP00000012502) --------------------------------------------------------------------------------
Xenopus(ENSXETP00000047703)    FQVDKKRPTSSVKFQWSLALTSNSTESSVLSILKASPFYTITSGLLRSNKRKSSDEEKDTDVSA----------------
Chicken(ENSGALP00000056402)    -------------------------------------------------SAEKDEEEEEELEEK----------------
Turtle(ENSPSIP00000003025)     -------------------------------------------E-------TNSEKEEEELEEK----------------
Lizard(ENSACAP00000004041)     ------------------------------------------------NLENEEEEEEEQFGEK----------------
Tasmanian devil(ENSSHAP0000001 -------------------------------------------E-------MNIEKEEEKIIEASTHQNTLWLKPASEGL
Mouse(ENSMUSP00000136669)      ---------------------------------------------------TPEDRDEDEREEK----------------
Rat(ENSRNOP00000063831)        -------------------------------------------NESAAEERDQDKKRRVAGTESRASRAGESVEKPERVR
Pig(ENSSSCP00000052164)        -------------------------------------------PV------EEEEKDQEEKRRKVTSRDSVAGLLPTEEP
Human(ENSP00000345739)         -------------------------------------------DESIKEEDKDQDEKRRRVTSRERVARPLPAEEPERAK
Lamprey(ENSPMAP00000002362)    ---------------------------------------------------KEEDIQDSQTKEE----------------
Ciona(ENSCSAVP00000010172)     --------------------------------------------------------------------------------
Amphioxus(jgi|Brafl1|204680|e_ --------------------------------------------------------------------------------

                                      570       580       590       600       610       620       630       640
                               ....|....|....|....|....|....|....|....|....|....|....|....|....|....|....|....|
Tetraodon(ENSTNIP00000019029)  ------------EREEKRL-----------------------------------------------------KVESN---
Fugu(ENSTRUP00000020321)       ------------AREEKRL-----------------------------------------------------KVESN---
Tilapia(ENSONIP00000001969)    ------------SREEKRL-----------------------------------------------------KVESD---
Medaka(ENSORLP00000034016)     ------------SQEKKRL-----------------------------------------------------KVESD---
Amazon molly(ENSPFOP0000000622 ------------TQEEKRL-----------------------------------------------------KADSD---
Platyfish(ENSXMAP00000018161 ) ------------TQVEKRL-----------------------------------------------------RVDSD---
Stickleback(ENSGACP00000018172 ------------TREEKRL-----------------------------------------------------KVESD---
Cod(ENSGMOP00000003747)        ------------SREEKRL-----------------------------------------------------KVDSD---
Salmon1b (XP_014048854.1)      ------------TQEEKRF-----------------------------------------------------KVEPD---
Salmon1a(XP_014059406.1)       ------------TQKEKRF-----------------------------------------------------KVEPD---
Trout1b(XP_021480911.1)        ------------TPEEKRF-----------------------------------------------------KVEPD---
Trout1a(XP_021413584.1)        ------------TQEEKRF-----------------------------------------------------KVEPD---
Cavefish(ENSAMXP00000012561)   --------------ASAHM-----------------------------------------------------LVPDI---
Zebrafish(ENSDARP00000013243)  ------------VREEKRL-----------------------------------------------------KTEDE---
Gar(ENSLOCP00000009024)        ------------AQDEKRL-----------------------------------------------------KVESE---
Shark(XP_007910047.1)          -----------EEQDEKRI-----------------------------------------------------KVES----
Coelacanth(ENSLACP00000012502) --------------------------------------------------------------------------------
Xenopus(ENSXETP00000047703)    ----DADQPEEKEKEEKRI-----------------------------------------------------KIEVN---
Chicken(ENSGALP00000056402)    ------------EQDEKRI-----------------------------------------------------KIETK---
Turtle(ENSPSIP00000003025)     ------------EHDEKRM-----------------------------------------------------KIETK---
Lizard(ENSACAP00000004041)     ------------EPDVKRF-----------------------------------------------------KTETK---
Tasmanian devil(ENSSHAP0000001 KCGCTIRRKLVNEQGEKKM-----------------------------------------------------KFELKEGS
Mouse(ENSMUSP00000136669)      ------------RRKTTRK-----------------------------------------------------KLESH---
Rat(ENSRNOP00000063831)        PGTQLCQEEQGEQEDDRRPRRQTRELASRRKSREDPDREARPGTHLDVDDDDEKDKRSSRPRSQPRDLATKRRPKEE-VE
Pig(ENSSSCP00000052164)        ERVRPGTHMEEDDKEEKRLRSQTKELTPKQKIKEELDRSTRPG--GAQPETNEEDKDEKRHRSQPKDLAGKRRPEEKEPE
```

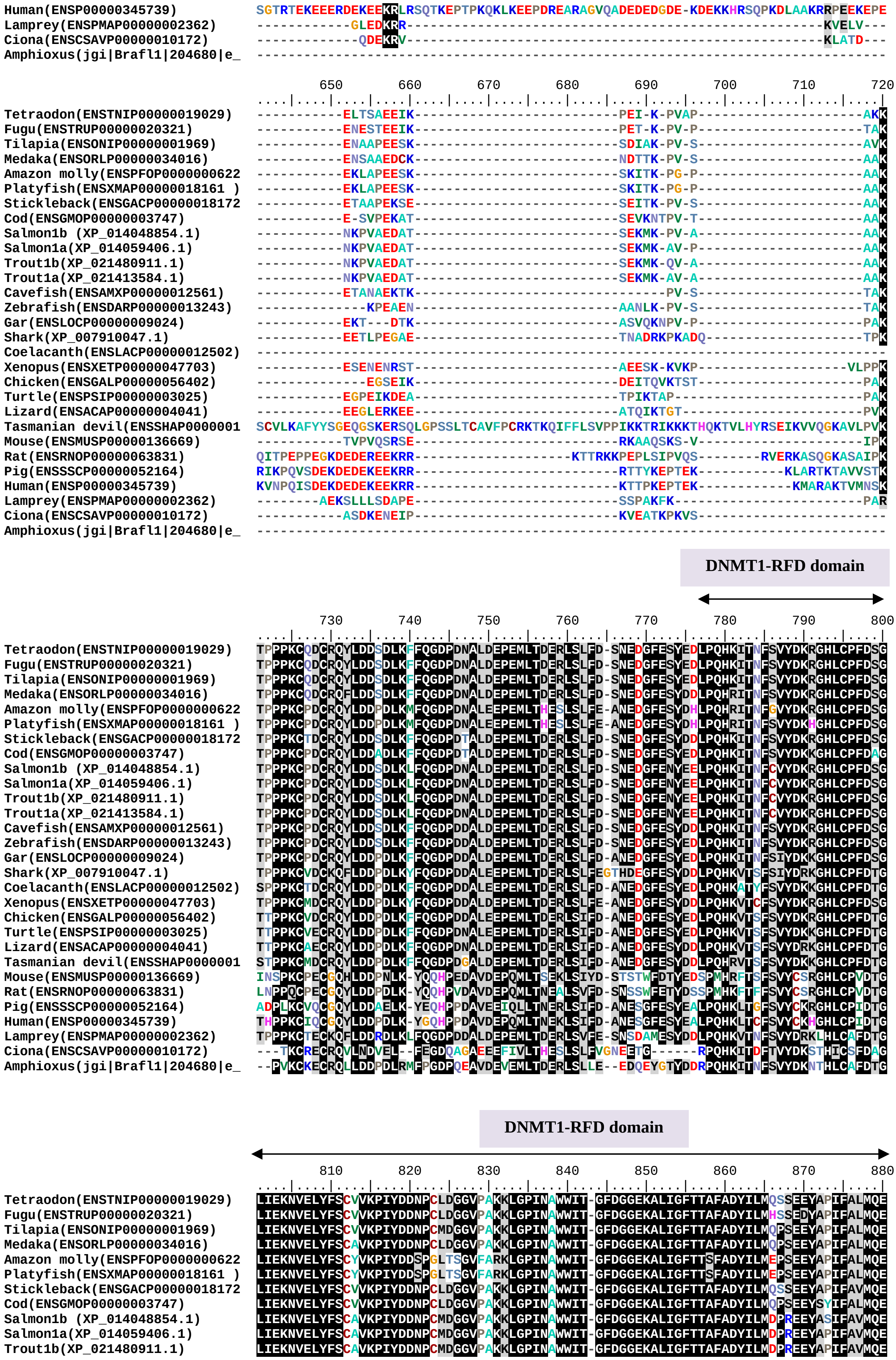

Human(ENSP00000345739)
Lamprey(ENSPMAP00000002362)
Ciona(ENSCSAVP00000010172)
Amphioxus(jgi|Brafl1|204680|e_
650
660
670
680
690
700
710
720
Tetraodon(ENSTNIP00000019029)
Fugu(ENSTRUP00000020321)
Tilapia(ENSONIP00000001969)
Medaka(ENSORLP00000034016)
Amazon molly(ENSPFOP0000000622
Platyfish(ENSXMAP00000018161)
Stickleback(ENSGACP00000018172
Cod(ENSGMOP00000003747)
Salmon1b (XP_014048854.1)
Salmon1a(XP_014059406.1)
Trout1b(XP_021480911.1)
Trout1a(XP_021413584.1)
Cavefish(ENSAMXP00000012561)
Zebrafish(ENSDARP00000013243)
Gar(ENSLOCP00000009024)
Shark(XP_007910047.1)
Coelacanth(ENSLACP00000012502)
Xenopus(ENSXETP00000047703)
Chicken(ENSGALP00000056402)
Turtle(ENSPSIP00000003025)
Lizard(ENSACAP00000004041)
Tasmanian devil(ENSSHAP0000001
Mouse(ENSMUSP00000136669)
Rat(ENSRNOP00000063831)
Pig(ENSSSCP00000052164)
Human(ENSP00000345739)
Lamprey(ENSPMAP00000002362)
Ciona(ENSCSAVP00000010172)
Amphioxus(jgi|Brafl1|204680|e_
DNMT1-RFD domain
730
740
750
760
770
780
790
800
DNMT1-RFD domain
810
820
830
840
850
860
870
880
Trout1b(XP_021480911.1)

```
Trout1a(XP_021413584.1)             LIEKNVELYFSCAVKPIYDDNPCMDGGVPAKKLGPINAWWIT-GFDGGEKALIGFTTAFADYILMDPREEYAPIFAVMQE
Cavefish(ENSAMXP00000012561)        LIEKNVELYFSCAVKPIYDDNPCMDGGVPAKKLGPINAWWIT-GFDGGEKALIGFTTAFADYILMEPSEEYSPIFALMQE
Zebrafish(ENSDARP00000013243)       LIEKNVELYFSCAVKPIYDDNPCMDGGVPAKKLGPINAWWIT-GFDGGEKALIGFTTAFADYILMDPSEEYSAIFALMQE
Gar(ENSLOCP00000009024)             LIEKNVELYFSCAVKPIYDDNPCMDGGVPAKKLGPINAWWIT-GFDGGEKALIGFTTAFADYILMDPSEEYAPIFALMQE
Shark(XP_007910047.1)               LIERNVELYFSGVVKPIYDDNPCLDGGVKAKKLGPINAWWIT-GFDGGEKALIGFTTAFADYILMDPREEYAAIFAVMQE
Coelacanth(ENSLACP00000012502)      LIEKNVELYFSGAVKPIYDDNPCLDGNIRVFKKKS-NIVYIK-GFPKSQQLGNALTLAFADYILMEPSEEYSAIFAVMQE
Xenopus(ENSXETP00000047703)         LIEKNVELYFSGYVKPIYDDNPSLDGGVRAKKLGPINAWWIT-GFDGGEKALIGFTTAFADYILMDPSEEYSSIFALMQE
Chicken(ENSGALP00000056402)         LIERNIELYFSGAVKPIYDDNPCLDGGVRAKKLGPINAWWIT-GFDGGEKALIGFTTAFADYILMEPSEEYAPIFALMQE
Turtle(ENSPSIP00000003025)          LVERNIELYFSGTVKPIYDDNPCLDGGVRAKKLGPINAWWIT-GFDGGERALIGFTTAFADYILMEPSEEYAPTFALMQE
Lizard(ENSACAP00000004041)          LIERNIELYFSGVVKPIYDDNPCLDGGVRARKMGPINAWWIT-GFDGGEKALIGFTTAFADYILMEPSEEYAPIFALMQE
Tasmanian devil(ENSSHAP0000001      LIEKNIELYFSGTVKPIYDDNPCLDGGVRAKKLGPINAWWIT-GFDGGEKALIGFTTAFADYILMAPSEEYAPIFALMQE
Mouse(ENSMUSP00000136669)           LIEKNVELYFSGCAKAIHDENPSMEGGINGKNLGPINQWWLS-GFDGGEKVLIGFSTAFAEYILMEPSKEYEPIFGLMQE
Rat(ENSRNOP00000063831)             LIEKNVELYFSGVAKAIHEENPSVEGGVNGKNLGPINQWWIS-GFDGGEKALIGFSTAFAEYFLMEPSPEYAPIFGLMQE
Pig(ENSSSCP00000052164)             LIEKDVELFFSGSAKPIYEDDPSLEGGVNGKNLGPINEWWIT-GFDGGEKALIGFSTSFAEYILMDPNPEYAPLFSVMQE
Human(ENSP00000345739)              LIEKNIELFFSGSAKPIYDDDPSLEGGVNGKNLGPINEWWIT-GFDGGEKALIGFSTSFAEYILMDPSPEYAPIFGLMQE
Lamprey(ENSPMAP00000002362)         LVEKNVELYLSGVVKPIYDENPCNDGGVSAKKLGPINSWWTT-GFDGGEKALVGITTAFADYILMNPSEEYASTFAVMQE
Ciona(ENSCSAVP00000010172)          LIEKNVELYFSGLVKPIYDEDPSTDTGVPGKRLGPINEWWTAGGFDGGERALVGFSTGFADYYLMEPSLSYSPQWSSIQE
Amphioxus(jgi|Brafl1|204680|e_      LIEKNVELYFSGAVKPIYDENPSADGGVLAKNVGPINEWWTA-GFDGGENALIGFTTAFAEYIVMQPSEAYAPFMDTMWE
```

DNMT1-RFD domain

```
                                            890       900       910       920       930       940       950       960
                                    ....|....|....|....|....|....|....|....|....|....|....|....|....|....|....|....|
Tetraodon(ENSTNIP00000019029)       KIYMSKIVVEFLQKNREATYEDLLNKIETTVPPAGLNFNCFTEDTLLRHAQFVVEQVESYDEAGDSDEERIIVTPCMRDL
Fugu(ENSTRUP00000020321)            KIYMSKIVVEFLQKNREATYEDLLNKIETTVPPAGLNFNCFTEDTLLRHAQFVVEQVESYDEAGDSDEERIIVTPCMRDL
Tilapia(ENSONIP00000001969)         KIYMSKIVVEFLQKNPDATYEDLLNKIETTVPPAGLNFNCFTEDTLLRHAQFVVEQVESYDEAGDSDEQPIIVTPCMRDL
Medaka(ENSORLP00000034016)          KIYMSKIVVEFLQKNPDVTYEDLLNKIETTVPPAGLNFNCFTEDTLLRHAQFVVEQVESYDEAGDSDEQPIIVTPCMRDL
Amazon molly(ENSPFOP0000000622      KIYMSKIVVEFLQKNPDATYEDLLNKIETTVPPAGLNFNCFTEDTLLRHAQFVVEQVESYDEAGDSDEQPIIVTPCMRDL
Platyfish(ENSXMAP00000018161 )      KIYMSKIVVEFLQKNPDATYEDLLNKIETTVPPAGLNFNCFTEDTLLRHAQFVVEQVESYDEAGDSDEQPIIVTPCMRDL
Stickleback(ENSGACP00000018172      KIYMSKIVVEFLQKNPDASYEDLLNKIETTVPPAGLNFNCFTEDTLLRHAQFVVEQVESYDEAGDSDEQPIIVTPCMRDL
Cod(ENSGMOP00000003747)             KIYMSKIVVEFLQKNPDVSYEDLLNKIETTVPPAGLNFNSFTEDTLLRHAQFVVEQVESYDEAGDQDEQPIIVTPCMRDL
Salmon1b (XP_014048854.1)           KIYMSKMVVEFLQKNPDVSYEDLLNKIETTVPPAGLNFNRFTEDTLLRHAQFVVEQVESYDEAGDSDEQPIIVTPCMRDL
Salmon1a(XP_014059406.1)            KIYMSKMVVEFLQKNPDVSYEDLLNKIETTVPPAGLNFNRFTEDTLLRHAQFVVEQVESYDEAGDSDEQPIIVTPCMRDL
Trout1b(XP_021480911.1)             KIYMSKMVVEFLQKNPDVSYEDLLNKIETTVPPAGLNFNRFTEDTLLRHAQFVVEQVESYDEAGDSDEQPIIVTPCMRDL
Trout1a(XP_021413584.1)             KIYMSKMVVEFLQKNPDVSYEDLLNKIETTVPPAGLNFNRFTEDTLLRHAQFVVEQVESYDEAGDSDEQPIIVTPCMRDL
Cavefish(ENSAMXP00000012561)        KIYMSKIVVEFLQKNPDVTYEDLLNKIETTVPPAGLNFNRFTEDTLLRHAQFVVEQVESYDEAGDSDEQPIIVTPCMRDL
Zebrafish(ENSDARP00000013243)       KIYMSKIVVEFLQKNQDATYEDLLNKIETTVPPAGLNFNRFTEDTLLRHAQFVVEQVESYDEAGDSDEQPIIITPCMRDL
Gar(ENSLOCP00000009024)             KIYMSKIVVEFLQKNPEATYEDLLNKIETTVPPAGLNFNRFTEDTLLRHAQFVVEQVESYDEAGDSDEQPIIVTPCMRDL
Shark(XP_007910047.1)               KIHMSKIVIEFVQNNPDSTYEDLLNKIETTVPPAGLSFSRFTEDSLLRHAQFVVEQVESYDEAGDVDEQPIIIIPCMRDL
Coelacanth(ENSLACP00000012502)      KIYMSKIVVEFLQKNPDATYEDLLNKIETTVPPAGLNFNRFTEDSLLRHAQFVVEQVESYDEAGDIDEQPIIITPCMRDL
Xenopus(ENSXETP00000047703)         KIYMSKIVVEFLQNNPDVSYEDLLNKIETTVPPSGLNFNRFTEDSLLRHAQFVVEQVESYDEAGDSDEQPVIVTPCMRDL
Chicken(ENSGALP00000056402)         KIYMSKIVVEFLQNNRDVSYEDLLNKIETTVPPVGLNFNRFTEDSLLRHAQFVVEQVESYDEAGDSDEPPVLITPCMRDL
Turtle(ENSPSIP00000003025)          KIYMSKIVVEFLQNNPHVSYEDLLNKIETTVPPAGLNFNRFTEDSLLRHAQFVVEQVESYDEAGDSDEPPVLITPCMRDL
Lizard(ENSACAP00000004041)          KIYMSKIVVEFLQNNPDVSYEDLLNKIETTVPPAGLNFNRFTEDSLLRHAQFVVEQVESYDEAGDSDEPPVLITPCMRDL
Tasmanian devil(ENSSHAP0000001      KIYMSKIVVEFLQNNPDVSYEDLINKIETTVPPAGLNFNRFTEDSLLRHAQFVVEQVESYDEAGDSDEQPVIITPCMRDL
Mouse(ENSMUSP00000136669)           KIYISKIVVEFLQNNPDAVYEDLINKIETTVPPSTINVNRFTEDSLLRHAQFVVSQVESYDEAKDDDETPIFLSPCMRAL
Rat(ENSRNOP00000063831)             KIYISKIVVEFLQSNPDAVYEDLINKIETTVPPSAINVNRFTEDSLLRHAQFVVSQVESYDDAKDDDETPIFLSPCMRSL
Pig(ENSSSCP00000052164)             KIYISKIVVEFLQNNPDSTYEDLINKIETTVPPSVLNLNRFTEDSLLRHAQFVVEQVESYDQAGDSDEQPIFLTPCMRDL
Human(ENSP00000345739)              KIYISKIVVEFLQSNSDSTYEDLINKIETTVPPSGLNLNRFTEDSLLRHAQFVVEQVESYDEAGDSDEQPIFLTPCMRDL
Lamprey(ENSPMAP00000002362)         KIYMFKIVIEFLQHNRDATYEDLLNKIETTVPPCGLSFTRFTEDSLLRHAQFIVEQVESYDEAGDEDEPPIIITPCMRDL
Ciona(ENSCSAVP00000010172)          KIYMSKNVIEFLSETPDADYEDLVNRIQTSVPPPTLGIAKFTEDSLLRHAQFVVEQVESYDNARSEEDSLLGTPCMRDL
Amphioxus(jgi|Brafl1|204680|e_      KIHMSKLVIEFLVNNQEAAYEDLLNKIQTTVPPQSLRVTSFSEESLLRHAQFVVEQVENFDLAGDADEAPLLTTPCMRAL
```

zf-CXXC domain

```
                                            970       980       990      1000      1010      1020      1030      1040
                                    ....|....|....|....|....|....|....|....|....|....|....|....|....|....|....|....|
Tetraodon(ENSTNIP00000019029)       IKLAGVTLGKR-----R-AARRQAIRHPTKIEKDSKG-PTKATTTKLVCQIFDTFFSDQIEQN----DKEVVGVKRHRCG
Fugu(ENSTRUP00000020321)            IKLAGVTLGKR-----R-AARRQAIRHPTKIEKDSKG-PTKATTTKLVCQIFDTFFSDQIEQN----DKEVVGVKRHRCG
Tilapia(ENSONIP00000001969)         IKLAGVTLGKR-----R-AARRQAIRHPTKIEKDSKG-PTKATTTKLVYQIFDAFFSDQIEQN----DKE-SAMKRQRCG
Medaka(ENSORLP00000034016)          IKLAGVTLGKSTLLYWR-AARRQAIRHPTKIEKDSKG-PTKATTTKLVYQIFDAFFSDQIEQN----DKE-SALKRQRCG
Amazon molly(ENSPFOP0000000622      IKLAGVTLGKR-----R-AARRQTIRHPTKIEKDSKG-PTKATTTKMVYQIFDAFFADQIEQN----DKE-GGVKRQRCG
Platyfish(ENSXMAP00000018161 )      IKLAGVTLGKR-----R-AARRQTIRHPTKIEKDSKG-PTKATTTKMVYQIFDAFFADQIEQN----DKE-GGVKRQRCG
Stickleback(ENSGACP00000018172      IKLAGVTLGKR-----R-AARRQAIRHPTKIEKDNKG-PTKATTTTLVYQIFDTFFSEQIEQN----DKDSAGAKRQRCG
Cod(ENSGMOP00000003747)             IKLAGVTLGKR-----R-AARRQAIRHPTKIEKDLKG-PTKATTTKLVYTIFDTFFSDQIDQI----DKD-GGIKRQRCG
Salmon1b (XP_014048854.1)           IKLAGVTLGKR-----R-AARRQAIRHPTKIEKDSKGGPTRATTTKLVYKIFDTFFSDQIDQN----EKD-GGIKRQRCG
Salmon1a(XP_014059406.1)            IKLAGVTLGKSMLLYWR-AARRQAIRHPTKIEKDSKGGPTRATTTKLVYKIFDTFFSDQIDQN----EKD-GGTKRQRCG
Trout1b(XP_021480911.1)             IKLAGVTLGKR-----R-AARRQAIRHPTKIEKDSKGGPTRATTTKLVYKIFDTFFSDQIDQN----EKD-GGIKRQRCG
Trout1a(XP_021413584.1)             IKLAGVTLGKSMLLYWR-AARRQAIRHPTKIERDSKGGPTRATTTKLVYKIFDTFFSDQIDQN----EKD-GGIKRQRCG
Cavefish(ENSAMXP00000012561)        IKLAGVTLGKR-----R-AARRQAIRHPTKIEKDLKG-PTKATTTKLVYQIFDTFFSDQIDQN----NKD-GGIKRQRCG
Zebrafish(ENSDARP00000013243)       IKLAGVTLGKR-----R-AARRQAVRHPTKIEKDNKG-PTKATTTKLVYLIFDTFFSDQIDQN----NKD-GGVKRQRCG
Gar(ENSLOCP00000009024)             IKLAGVTLGKSMLLYWR-AARRQAIRHPTKIEKDNKG-PTKATTTKLVYQIFDTFFSDQIEQN----DKE-SSAKRRRCG
Shark(XP_007910047.1)               IKLAGVTLGKR-----R-AARRQAIRHPTKIDKD-KG-PTKATTTSLVYQIFDTFFSEQIDQN----DKE-NGLKRQRCG
Coelacanth(ENSLACP00000012502)      IKLAGVTLGKR-----R-AARRQAIRHPTKIEKD-KG-PTKATTTKLVYQIFDIFFSEQIEQN----NKE-NIFKRRRCG
Xenopus(ENSXETP00000047703)         IKLAGVTLGKR-----R-AARRQAIRHPTKIEKDNKG-PTKATTTKLVYQIFDTFFSEQIEKDA---DKE-NGIKRRRCG
Chicken(ENSGALP00000056402)         IKLAGVTLGKR-----R-AVRRQAIRHPTRIDKD-KG-PTKATTTKLVYLIFDTFFSEQIEKDEREDDKE-NAMKRRRCG
Turtle(ENSPSIP00000003025)          IKLAGVTLGKS-----RLAARRQAIRHPTKIDKD-KG-PTKATTTKLVYLIFDTFFSEQIEKNEREDDKE-NAMKRRRCG
Lizard(ENSACAP00000004041)          IKLAGVTLGKR-----R-AARRQAIRHPTKIDKD-KG-PTKATTTKLVYLIFDTFFSEQIEKTEKEEDNE-NVSKRRRCG
Tasmanian devil(ENSSHAP0000001      IKLAGVTLGKR-----R-AARRQAIRHPTKINKE-KG-PTKATTTKLVYLIFDTFFSEQIEKNEREDDKE-NVTKRRRCG
Mouse(ENSMUSP00000136669)           IHLAGVSLGQR-----R-ATRR--VMGATK-EKD-KA-PTKATTTKLVYQIFDTFFSEQIEKYDKE-DKE-NAMKRRRCG
Rat(ENSRNOP00000063831)             IHLAGVSLGQR-----R-ATRRTVI-NSAKVKR--KG-PTKATTTKLVYQIFDTFFSEQIEKDDKE-DKE-NTMKRRRCG
Pig(ENSSSCP00000052164)             IKLAGVTLGKR-----R-AERRRTIGHSTK-EKD-KG-PTKATTTKLVYQIFDTFFAEQIEKDDKE-DKE-NAFKRRRCG
Human(ENSP00000345739)              IKLAGVTLGQR-----RAQARRQTIRHSTR-EKD-RG-PTKATTTKLVYQIFDTFFAEQIEKDDRE-DKE-NAFKRRRCG
Lamprey(ENSPMAP00000002362)         IKLAGVTLGKR-----R-AQRRQTIKHPTKIAKDAKG-PSKATTTLLVRQIFDEFFDEQIEQR----DKE-NVIKRRRCG
Ciona(ENSCSAVP00000010172)          IHLAGVTLGKR-----R-VQRRTAS-------------STKATTTRLVAEVMEKFFGGTVDA-----TTG-NKIRRRRCG
Amphioxus(jgi|Brafl1|204680|e_      IKLAGVTLGKR-----R--AGRSGIKQPRKVKAKPMR-QTKATTTNLVCSIFDTFFKDQIQD-----DKA-GGPRRRRCG
```

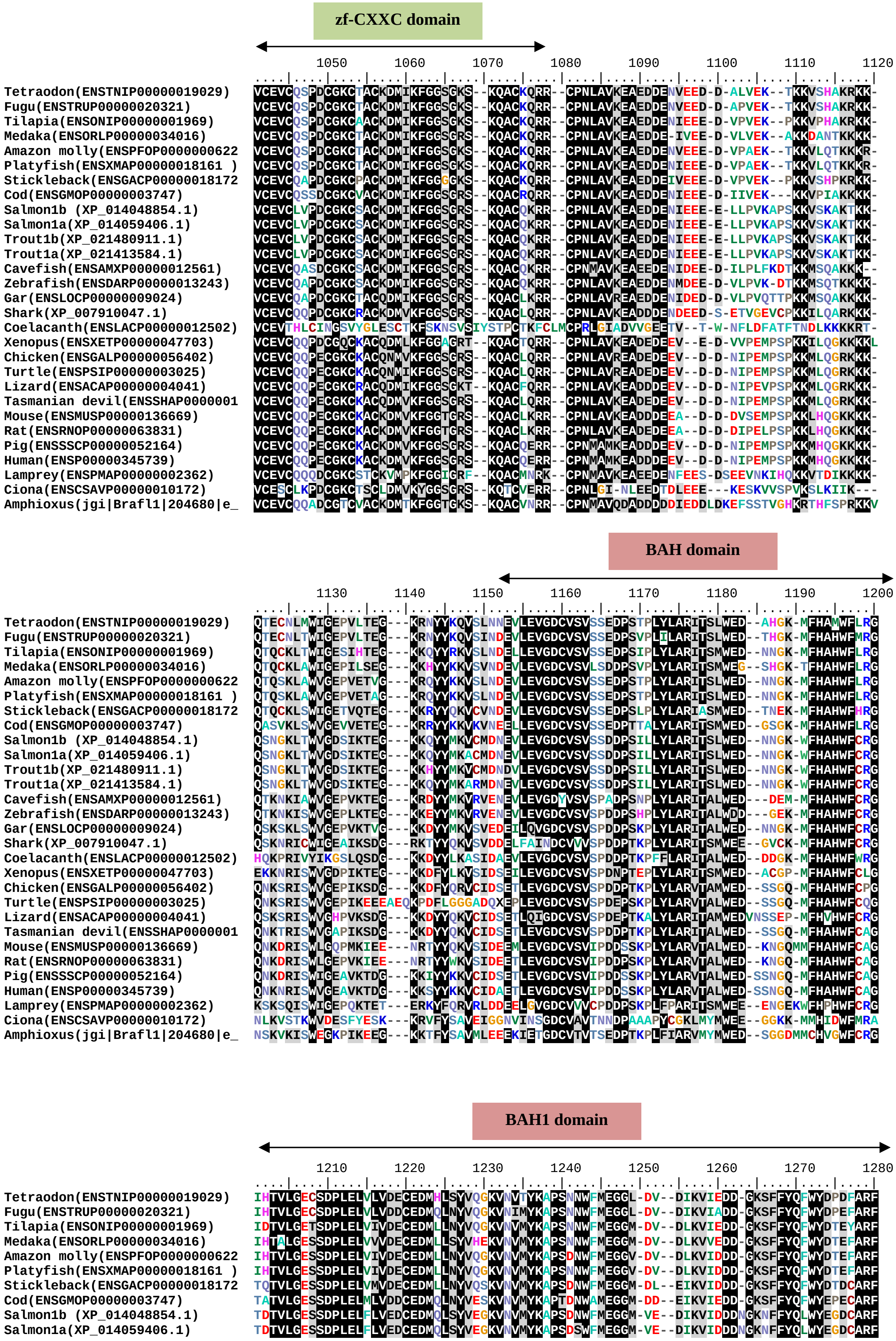
zf-CXXC domain
BAH domain
BAH1 domain
Tetraodon(ENSTNIP00000019029)
Fugu(ENSTRUP00000020321)
Tilapia(ENSONIP00000001969)
Medaka(ENSORLP00000034016)
Amazon molly(ENSPFOP0000000622
Platyfish(ENSXMAP00000018161)
Stickleback(ENSGACP00000018172
Cod(ENSGMOP00000003747)
Salmon1b (XP_014048854.1)
Salmon1a(XP_014059406.1)
Trout1b(XP_021480911.1)
Trout1a(XP_021413584.1)
Cavefish(ENSAMXP00000012561)
Zebrafish(ENSDARP00000013243)
Gar(ENSLOCP00000009024)
Shark(XP_007910047.1)
Coelacanth(ENSLACP00000012502)
Xenopus(ENSXETP00000047703)
Chicken(ENSGALP00000056402)
Turtle(ENSPSIP00000003025)
Lizard(ENSACAP00000004041)
Tasmanian devil(ENSSHAP0000001
Mouse(ENSMUSP00000136669)
Rat(ENSRNOP00000063831)
Pig(ENSSSCP00000052164)
Human(ENSP00000345739)
Lamprey(ENSPMAP00000002362)
Ciona(ENSCSAVP00000010172)
Amphioxus(jgi|Brafl1|204680|e_

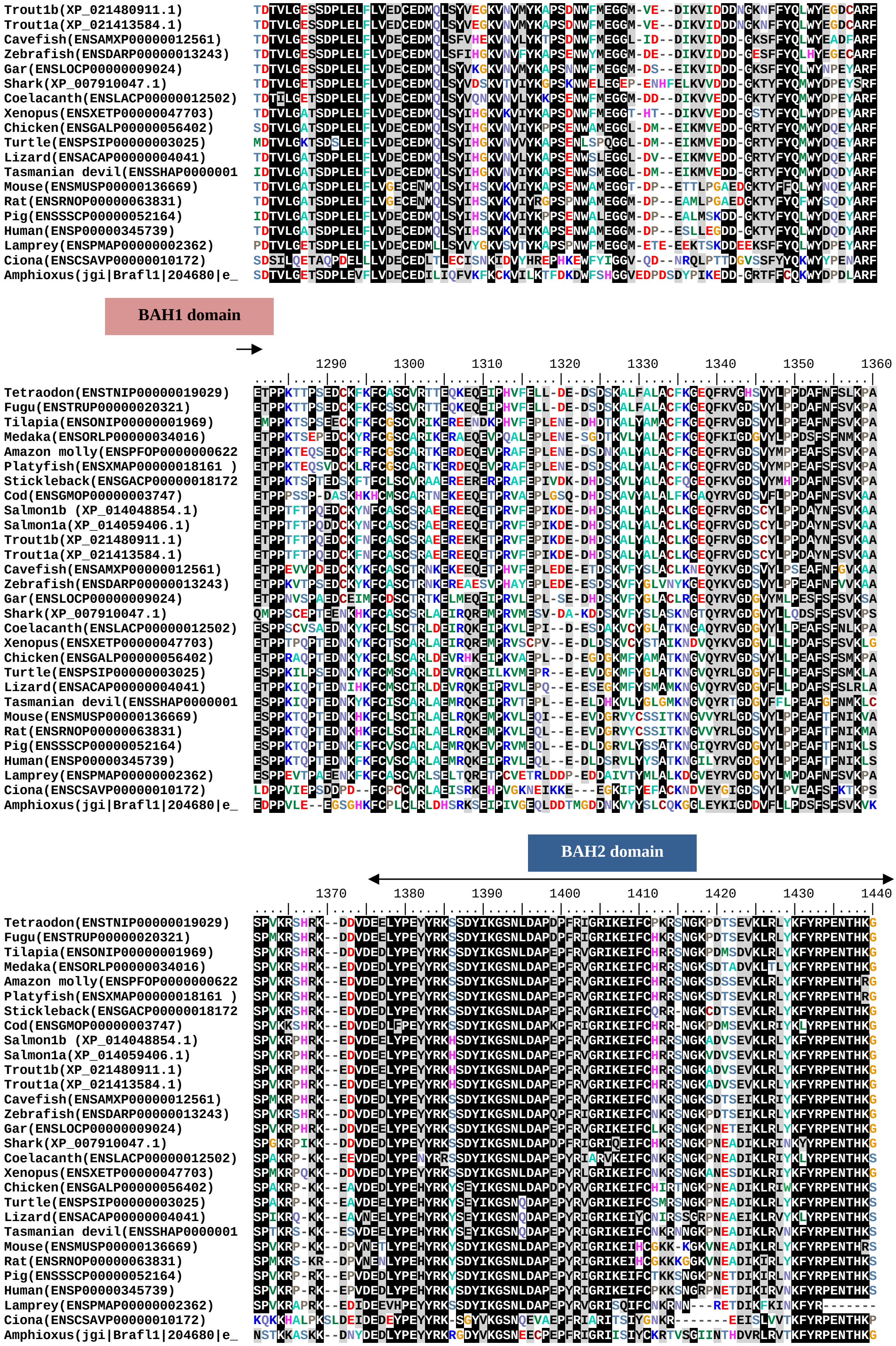
BAH1 domain
BAH2 domain

BAH2 domain

```
                                     1450      1460      1470      1480      1490      1500      1510      1520
                                 ....|....|....|....|....|....|....|....|....|....|....|....|....|....|....|....|
Tetraodon(ENSTNIP00000019029)    VKATYHADINQLYWSDEEVTVDMAQVLGRCQVEYGEDL-NEPVQEYSSGGPDRFYFL-EAYNAKTKSFEDPPNHARSVV-
Fugu(ENSTRUP00000020321)         VKATYHTDINQLYWSDEEVTVDMAQVLGRCQVEYGEDL-NEPVQEYSSGGPDRFYFL-EAYNAKTKSFEDPPNHARSAA-
Tilapia(ENSONIP00000001969)      VKASYHTDINQLYWSDEEVTVNMGDVLGRCQVEYAEDL-NESIQDYSSVGPDRFYFL-EAYSAKAKSFEDPPNHARSTV-
Medaka(ENSORLP00000034016)       VKASYHADINQLYWSDEEVTVRMSEVLGRCQIEYAEDL-IESVQDYSSGGPDRFYFL-EAYNARSKSFEDPPNHARSAV-
Amazon molly(ENSPFOP0000000622   VKASYHTDINQLYWSDEEVTVSMSEVLGRCQVEYAEDL-NESVQDYSSGGPDRFYFL-EAYNAKSKSFEDPPNHARSCV-
Platyfish(ENSXMAP00000018161 )   VKASYHTDINQLYWSDEEVTVSMSEVLGRCQVEYAEDL-NESVQDYSSGGPDRFYFL-EAYNAKSKSFEDPPNHARSCV-
Stickleback(ENSGACP00000018172   VKASYHADINQLYWSDEEVTVNMAELLGRCQVEYAEDL-NESVQDYSSGGTDRFYFL-EAYNAKSKSFEDPPNHARSAV-
Cod(ENSGMOP00000003747)          VKASYHTDINQLYWSEEEGTVGMAEVLGRCQVEYAEDL-NESVQDFSSGGPDRFYFL-EAYNAKTKGFEDPPNHARSSV-
Salmon1b (XP_014048854.1)        AKAGYHKDINQLYWSDEEVTVNMSEVLGRCRVEYGEDL-NETVQDFSSGGPDRFYFL-EAYNAKSKSFEDPPNHARSAV-
Salmon1a(XP_014059406.1)         AKAGYHTDINQLYWSDEEVTVNMSEVLGRCRVEYGEDL-NETVQDFSSGGPDRFYFL-EAYNAKSKSFEDPPNHARSAV-
Trout1b(XP_021480911.1)          AKAGYHTDINQLYWSDEEVTVNMSEVLGRCRVEYGEDL-NETVQDFSSGGPDRFYFL-EAYNAKSKSFEDPPNHARSAV-
Trout1a(XP_021413584.1)          AKAGYHTDINQLYWSDEEVTVNMSEVLGRCRVEYGEDL-NETVQDFSSGGPDRFYFL-EAYNAKSKSFEDPPNHARSAV-
Cavefish(ENSAMXP00000012561)     PKAGYHTDINQLYWSDEEATVNMADVLGRCRVEYGEDL-VESVQEYSTGGPDRFYFL-EAYNSKSKSFEDPPNHARSVV-
Zebrafish(ENSDARP00000013243)    PKGAYHSDINQLYWSDEEATVSMTEVLTRCRVEYAEDL-VESVQDYSNKGPDRFYFL-EAYNAKTKSFEDPPNHARSAV-
Gar(ENSLOCP00000009024)          PKAGYHTDINMLYWSDEEVTVDCKDVQGRCTVEYGEDL-IESVQEYSSAGPDRFYFL-EAYNSKTKSFEDPPNHARSAS-
Shark(XP_007910047.1)            LKASYHTDINLLYWSDEEVVVDFKDVQGRCRVEYGEDLTTENIHEYSAGGTDRFYFL-EAYNAKTKSYEDPPNRARSSA-
Coelacanth(ENSLACP00000012502)   LKASYHADINLLYWSDEEVTVDFKDVQGRCSVEYGEDL-TESIQEYSAGGLDRFYFL-EAYSAKSKTFEDPPNHARSAG-
Xenopus(ENSXETP00000047703)      VKASYHSDVNMVYWSDEEAVVDFKAVQGHCTVEYGEDL-TETIQEYSAGGSDRFYFL-EAYSAKTKSFEDPPNHARSAV-
Chicken(ENSGALP00000056402)      MKATYHADINLLYWSDEETTVDFCAVQGRCTVVYGEDL-TESIQDYSAGGLDRFYFL-EAYNAKTKSFEDPPNHARSSG-
Turtle(ENSPSIP00000003025)       VKASYHSDINLLYWSDEEVTVDFKAVQGRCTVEYGEDL-TAVASPTVPPGLGRSLVGAGAWRE-----GDPPPAVNGGR-
Lizard(ENSACAP00000004041)       VKASYHADINLLYWSDEEASVEFKDVQGRCTVVYGEDL-TENIQDYSASSSDRFYFL-EAYNAKTKTFEDPPYHARSSG-
Tasmanian devil(ENSSHAP0000001   MKASHHADINLLYWSDEEAIVDFKAVQGRCIIEYGEDL-TECIQDYSAGGSDRTYFLFEAYNAKTKSFEDPPNHARSPR-
Mouse(ENSMUSP00000136669)        YNGSYHTDINMLYWSDEEAVVNFSDVQGRCTVEYGEDL-LESIQDYSQGGPDRFYFL-EAYNSKTKNFEDPPNHARSPG-
Rat(ENSRNOP00000063831)          IQATYHADINLLYWSDEEAVVDFSDVQGRCTVEYGEDL-LESIQDYSQGGPDRFYFL-EAYNSKTKSFEDPPNHARSPG-
Pig(ENSSSCP00000052164)          TPASYHADINLLYWSDEEAVVDFKSVQGRCTVEYGEDL-PECLQDFSAGGPDRFYFL-EAYNAKSKSFEDPPNHARSPG-
Human(ENSP00000345739)           TPASYHADINLLYWSDEEAVVDFKAVQGRCTVEYGEDL-PECVQVYSMGGPNRFYFL-EAYNAKSKSFEDPPNHARSPG-
Lamprey(ENSPMAP00000002362)      --------------------------------------------------------------------------------
Ciona(ENSCSAVP00000010172)       LKSTYFCDLNMLYWTDEEVTVDFKLVQGKCRVECEFDL-NVSPAEYSKKGANRFYFV-EAYNAKEEAFYDAPVRSRRAGV
Amphioxus(jgi|Brafl1|204680|e_   KAGSHHTDLNMLYWSDEEAVVDFMSVQGKCQVVYGEDL-DVSVEDWSSKGLHRFYFN-EAYNTESKQFEEPPNKARSGG-
```

Dcm domain

```
                                     1530      1540      1550      1560      1570      1580      1590      1600
                                 ....|....|....|....|....|....|....|....|....|....|....|....|....|....|....|....|
Tetraodon(ENSTNIP00000019029)    ----HKGKGK-GKGKGK-----GKGK-AASE-SQDTPNEPQ-TLKVPKYRTLDVFSGCGGLSEGFHQA---GISETLWA
Fugu(ENSTRUP00000020321)         ----HKGKGK-GKGKGK-----GKGK-AAQD-SHDTPNEPQ-MPKVPKYRTLDVFSGCGGLSEGFHQA---GISESLWA
Tilapia(ENSONIP00000001969)      ----HKGKGK-GKGKGK-----GKGK-ASAA-QEQLDSQPQPKPKVPKYRTLDVFSGCGGLSEGFHQA---GISETLWA
Medaka(ENSORLP00000034016)       ----HKGKGK-GKGKGK-----GKGK-ASQE-QQDSNVGPQ-MPKVPKYRTLDVFSGCGGLSEGFHQA---GISESLWA
Amazon molly(ENSPFOP0000000622   ----HKGKGK-GKGKGK-----GKGK-ASQE-PQESRTEPQ-TLKVPKYRTLDVFSGCGGLSEGFHQA---GISETLWA
Platyfish(ENSXMAP00000018161 )   ----HKGKGK-GKGKGK-----GKGK-ASQE-PQESRTEPQ-ALKVPKYRTLDVFSGCGGLSEGFHQA---GISETLWA
Stickleback(ENSGACP00000018172   ----HKGKGK-GKGKGK-----GKGKAAAQE-QQDSQNEPQ-AVKVPKYRALDVFSGCGGLSEGFHQA---GMTETLWA
Cod(ENSGMOP00000003747)          ----QKGKGK-GKGKGK-----GKGK-SSAP--QEDVQEPQ-VPKVPKYRTLDVFSGCGGLSEGFPQV---NISETLWA
Salmon1b (XP_014048854.1)        ----NKGKGK-GKGKGK-----GKGK-SSSA-QEPPDQEPQ-DPKVPKLRTLDVFSGCGGLSEGFHQA---GIAETLWA
Salmon1a(XP_014059406.1)         ----NKGKGK-GKGKGK-----GKGK-SSSA-QEPPDQEPQ-DPKVPKLRTLDVFSGCGGLSEGFHQA---GIAETLWA
Trout1b(XP_021480911.1)          ----NKGKGK-GKGKGK-----GKGK-SSSA-QEPPDQEPQ-DPKVPKLRTLDVFSGCGGLSEGFHQA---GIAETLWA
Trout1a(XP_021413584.1)          ----NKGKGK-GKGKGK-----GKGK-SSSV-QEPPDQEPQ-DPKVPKLRTLDVFSGCGGLSEGFHQA---GIAETLWA
Cavefish(ENSAMXP00000012561)     ----HKGKGK-GKGKGK----------ASSQ--DVPEQEAQ-ELKVPKLRTLDVFSGCGGLSEGFHQA---GISETNWA
Zebrafish(ENSDARP00000013243)    ----NKGKGK-GKGKGK-----GKGK--AAP-QEPQDQEPQ-EPVVPKLRTLDVFSGCGGLSEGFHQA---GISETHWA
Gar(ENSLOCP00000009024)          ----HKGKGK-GKGKGK-----GKGKSSSSTVQEQLDQEPQ-DLKPHKLRTLDVFSGCGGLSEGFHQA---GVAETLWA
Shark(XP_007910047.1)            ----NKGKGK-GKGKGK-----GKSK-ATAE-PEQTEQENA-ESKFQKLRSLDVFSGCGGLSEGFHQA---GISETLWA
Coelacanth(ENSLACP00000012502)   ----NKGKGK-GKGKGK-----GKGK-ASVP-SEQTEQETA-ESKVLKLRTLDVFSGCGGLSEGFHQA---EIAETLWA
Xenopus(ENSXETP00000047703)      ----NKGKGK-GKGKGRTQTNSSKGK-TASK-SENEQQNCG-D-KLPKLRTLDVFSGCGGLSEGFHQA---GISETNWA
Chicken(ENSGALP00000056402)      ----NKGKGK-GKGKGK-----GKGK-SSTT-CEQSEPEPT-ELKLPKLRTLDVFSGCGGLSEGFHQA---GVSETLWA
Turtle(ENSPSIP00000003025)       ----RLSLGLWGKEPVS-----HAGV-GSTR-LPSALSRPT-ALG--QERALPRVSSTAGRSPGLTRSAPAPGIAETLWA
Lizard(ENSACAP00000004041)       ----NKGKGK-GKGKGK-----SKGK-SSTA-SEHSEHEAA-KMKPPKLRSLDVFSGCGGLSEGFHQA---EVSETLWA
Tasmanian devil(ENSSHAP0000001   ----NKGKGK-GKGKGK-----SRGK--SVA-VEPSEQETI-EVKLPKLRTLDVFSGCGGLSEGFHQA---GISETLWA
Mouse(ENSMUSP00000136669)        ----NKGKGK-GKGKGK-----GKHQ-VSEP------KEPEAAIKLPKLRTLDVFSGCGGLSEGFHQA---GISETLWA
Rat(ENSRNOP00000063831)          ----NKGKGK-GKGKGK-----GKPQ-VSEP------KEPEAAIKLPKLRTLDVFSGCGGLTEGFHQA---GISETLWA
Pig(ENSSSCP00000052164)          ----NKGKGK-GKGKSR-----TKSQ-TCEP------SELETEIKLPKLRTLDVFSGCGGLSEGFHQA---GISETLWA
Human(ENSP00000345739)           ----NKGKGK-GKGKGK-----PKSQ-ACEP------SEPEIEIKLPKLRTLDVFSGCGGLSEGFHQA---GISDTLWA
Lamprey(ENSPMAP00000002362)      --------------------------------------------------------------------------------
Ciona(ENSCSAVP00000010172)       QKLINEAKGI-KPKLTKLTKVTKVTKATKEESKLVSDDEVD-KNAFRKLHTLDVFSGCGGLSEGFHQA---GIAEPAYA
Amphioxus(jgi|Brafl1|204680|e_   ----KKAKGK-GKGKGK-----GKGK-SSEA--ESKEQQPP-SPAFTKLRMLDVFAGCGGLSEGFHQA---GIADSKWA
                                                                                  *  *             *
```

Motif I

Dcm domain

```
                                     1610      1620      1630      1640      1650      1660      1670      1680
                                 ....|....|....|....|....|....|....|....|....|....|....|....|....|....|....|....|
Tetraodon(ENSTNIP00000019029)    IEMWEPAAQAFRLNNPGATVFTEDCNILLKLVMSGEKTNSLGQKLPQKGDVEMLCGGPPCQGFSGMNRFNSRTYSAFKNS
Fugu(ENSTRUP00000020321)         IEMWEPAAQAFRLNNPGATVFTEDCNILLKLVMSGEKTNSLGQKLPQKGDVEMLCGGPPCQGFSGMNRFNSRTYSAFKNS
Tilapia(ENSONIP00000001969)      IEMWEPAAQAFRLNNPGTTVFTEDCNVLLKLVMSGEKTNSLGQKLPQKGDVEMLCGGPPCQGFSGMNRFNSRTYSKFKNS
Medaka(ENSORLP00000034016)       IEMWEPAAQAFRLNNPGTTVFTEDCNVLLKLVMSGEKTNSLGQKLPQKGDVEMLCGGPPCQGFSGMNRFNSRTYSKFKNS
Amazon molly(ENSPFOP0000000622   IEMWEPAAQAFRLNNPGTTVFTEDCNVLLKLVMSGEKTNSLGQKLPQKGDVEMLCGGPPCQGFSGMNRFNSRTYSKFKNS
Platyfish(ENSXMAP00000018161 )   IEMWEPAAQAFRLNNPGTTVFTEDCNVLLKLVMSGEKTNSLGQKLPQKGDVEMLCGGPPCQGFSGMNRFNSRTYSKFKNS
Stickleback(ENSGACP00000018172   IEMWEPAAQAFRLNNPGTTVFTEDCNVLLKLVMSGEKTNSLGQKLPQKGDVEMLCGGPPCQGFSGMNRFNSRTYSKFKNS
Cod(ENSGMOP00000003747)          IEMWEPAAQAFRLNNPGTTVFTEDCNVLLKLVMSGEKTNSLGQKLPQKGDVEMLCGGPPCQGFSGMNRFNSRTYSKFKNS
```

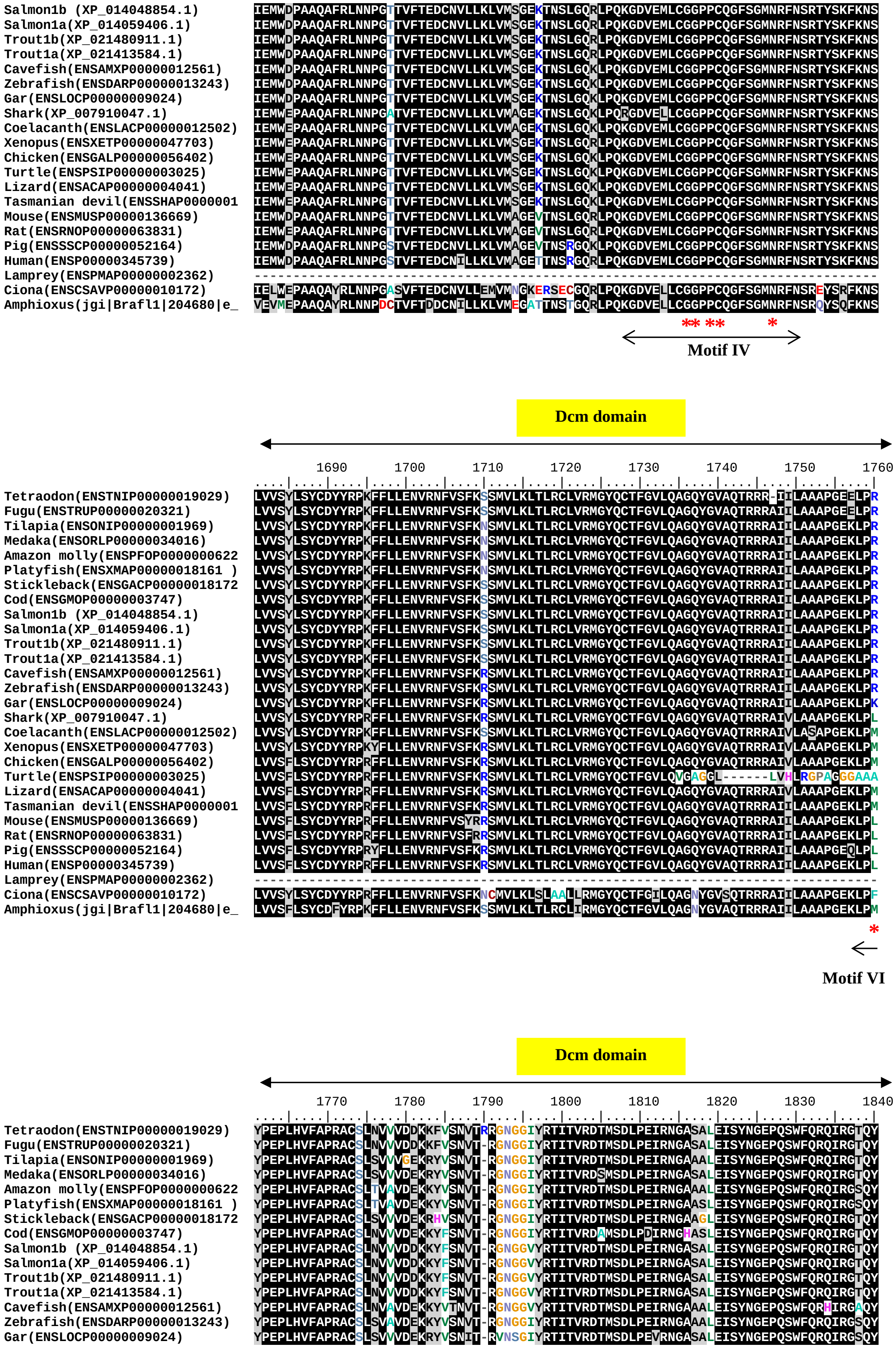

Salmon1b (XP_014048854.1)
Salmon1a(XP_014059406.1)
Trout1b(XP_021480911.1)
Trout1a(XP_021413584.1)
Cavefish(ENSAMXP00000012561)
Zebrafish(ENSDARP00000013243)
Gar(ENSLOCP00000009024)
Shark(XP_007910047.1)
Coelacanth(ENSLACP00000012502)
Xenopus(ENSXETP00000047703)
Chicken(ENSGALP00000056402)
Turtle(ENSPSIP00000003025)
Lizard(ENSACAP00000004041)
Tasmanian devil(ENSSHAP0000001
Mouse(ENSMUSP00000136669)
Rat(ENSRNOP00000063831)
Pig(ENSSSCP00000052164)
Human(ENSP00000345739)
Lamprey(ENSPMAP00000002362)
Ciona(ENSCSAVP00000010172)
Amphioxus(jgi|Brafl1|204680|e_
Motif IV
Dcm domain
1690
1700
1710
1720
1730
1740
1750
1760
Tetraodon(ENSTNIP00000019029)
Fugu(ENSTRUP00000020321)
Tilapia(ENSONIP00000001969)
Medaka(ENSORLP00000034016)
Amazon molly(ENSPFOP0000000622
Platyfish(ENSXMAP00000018161)
Stickleback(ENSGACP00000018172
Cod(ENSGMOP00000003747)
Motif VI
Dcm domain
1770
1780
1790
1800
1810
1820
1830
1840

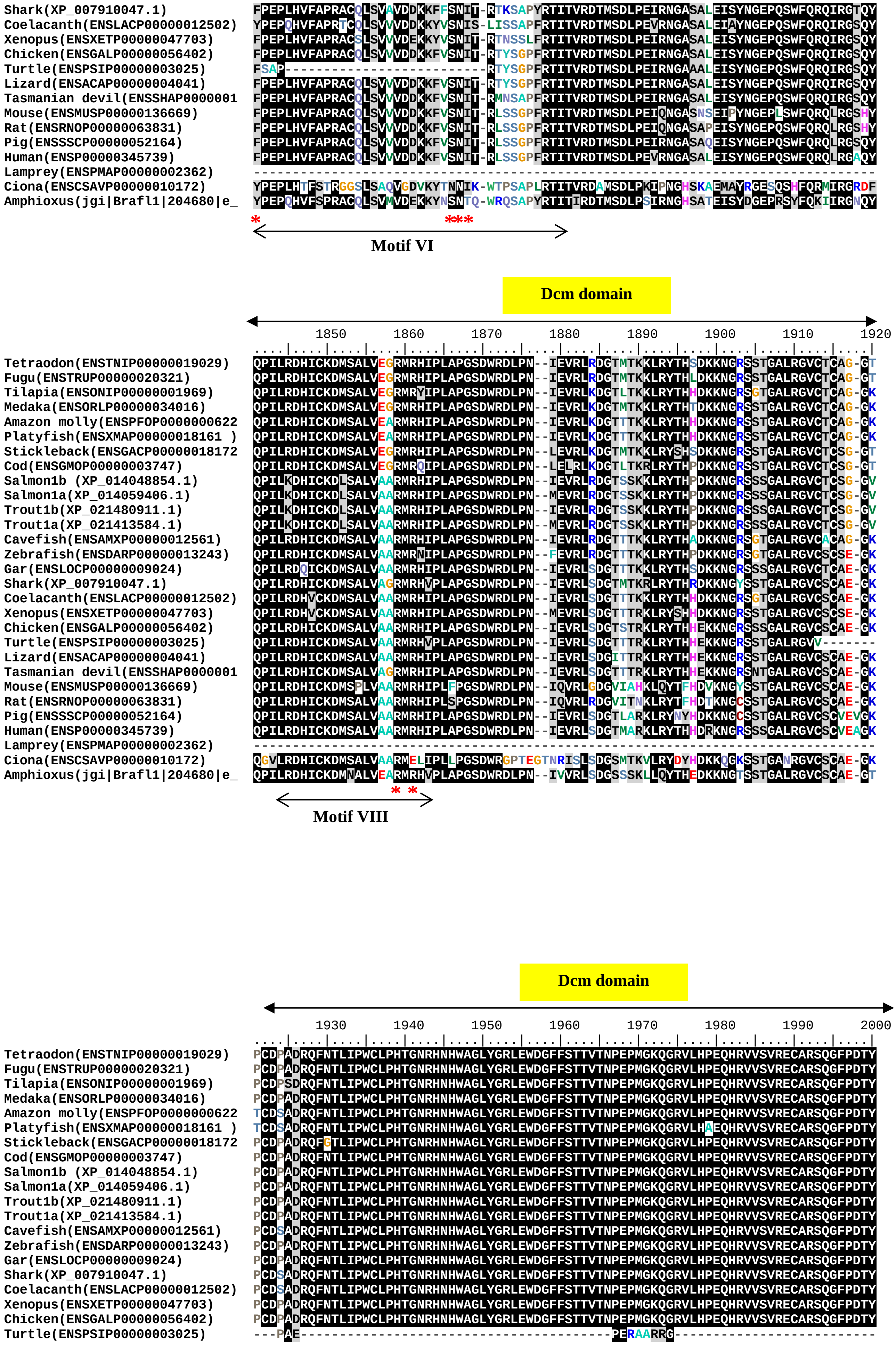
Shark(XP_007910047.1)
Coelacanth(ENSLACP00000012502)
Xenopus(ENSXETP00000047703)
Chicken(ENSGALP00000056402)
Turtle(ENSPSIP00000003025)
Lizard(ENSACAP00000004041)
Tasmanian devil(ENSSHAP0000001
Mouse(ENSMUSP00000136669)
Rat(ENSRNOP00000063831)
Pig(ENSSSCP00000052164)
Human(ENSP00000345739)
Lamprey(ENSPMAP00000002362)
Ciona(ENSCSAVP00000010172)
Amphioxus(jgi|Brafl1|204680|e_
Motif VI
Dcm domain
1850 1860 1870 1880 1890 1900 1910 1920
Tetraodon(ENSTNIP00000019029)
Fugu(ENSTRUP00000020321)
Tilapia(ENSONIP00000001969)
Medaka(ENSORLP00000034016)
Amazon molly(ENSPFOP0000000622
Platyfish(ENSXMAP00000018161)
Stickleback(ENSGACP00000018172
Cod(ENSGMOP00000003747)
Salmon1b (XP_014048854.1)
Salmon1a(XP_014059406.1)
Trout1b(XP_021480911.1)
Trout1a(XP_021413584.1)
Cavefish(ENSAMXP00000012561)
Zebrafish(ENSDARP00000013243)
Gar(ENSLOCP00000009024)
Motif VIII
Dcm domain
1930 1940 1950 1960 1970 1980 1990 2000

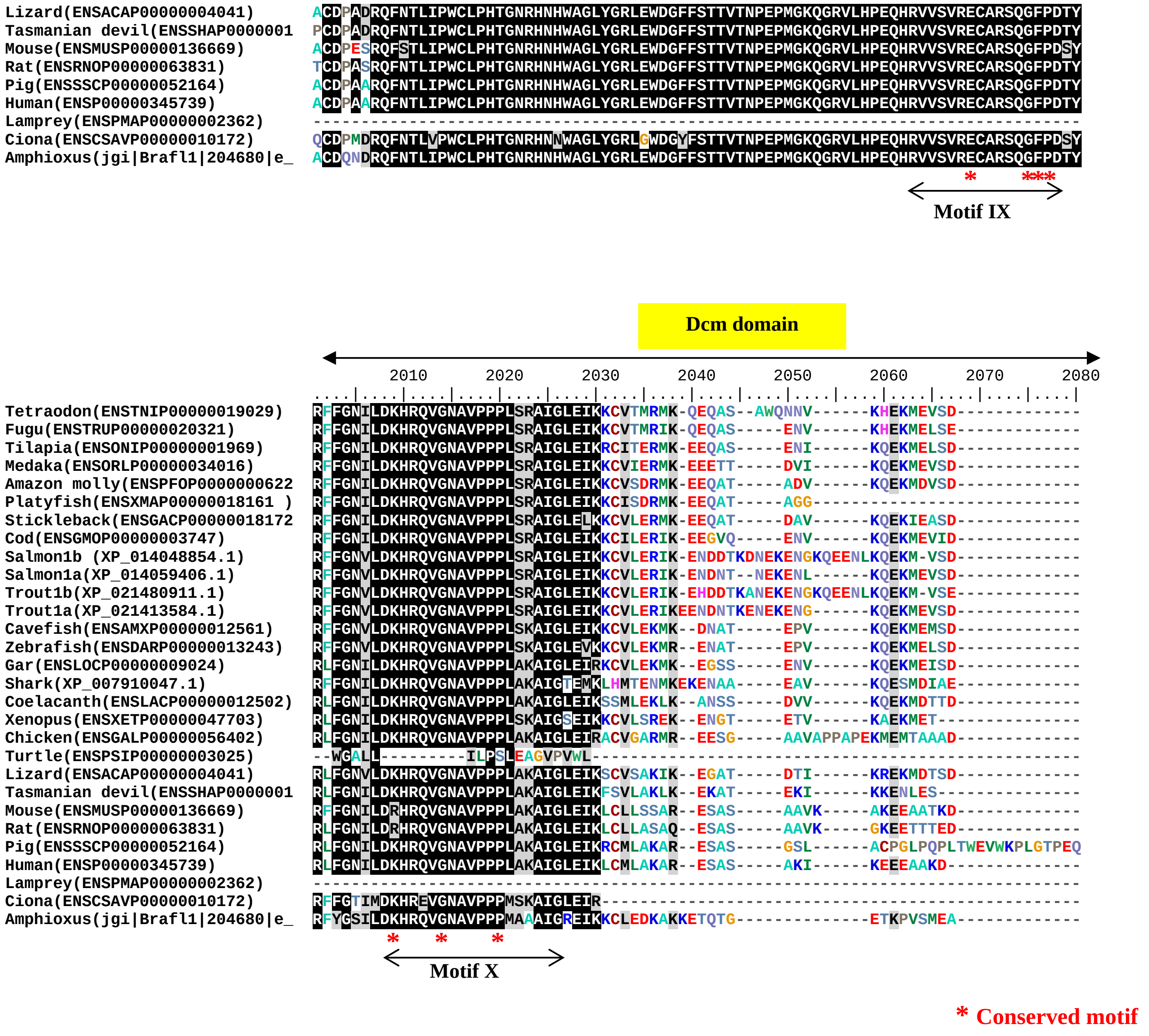

Supplemental Fig. S2 Dnmt1protein sequences alignment. All the protein sequences were aligned with the MUSCLE method in MEGA software. Conserved domains were noted above. Conserved motifs were marked with an asterisk (*).

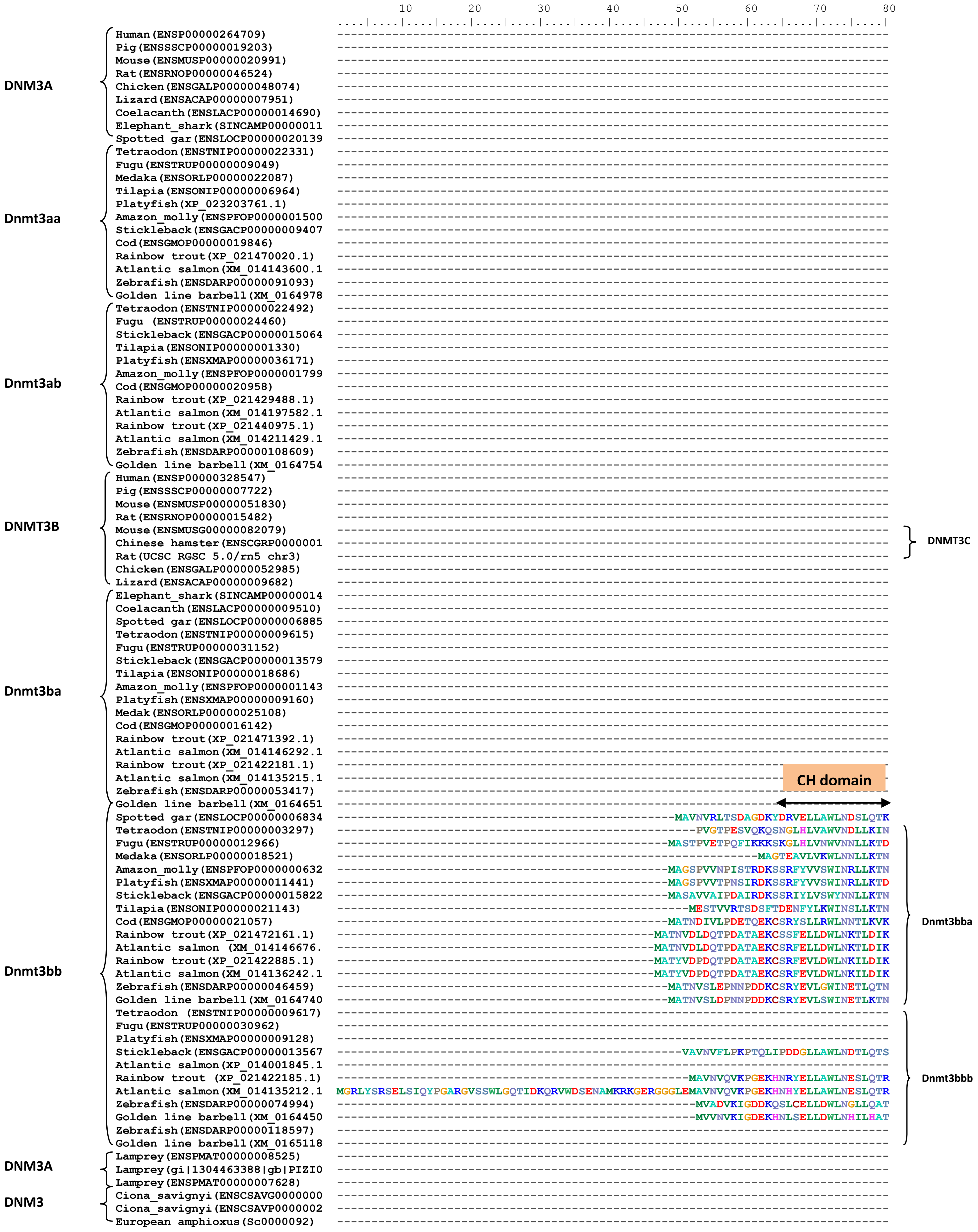

10 20 30 40 50 60 70 80
DNM3A
Human(ENSP00000264709)
Pig(ENSSSCP00000019203)
Mouse(ENSMUSP00000020991)
Rat(ENSRNOP00000046524)
Chicken(ENSGALP00000048074)
Lizard(ENSACAP00000007951)
Coelacanth(ENSLACP00000014690)
Elephant_shark(SINCAMP00000011
Spotted gar(ENSLOCP00000020139
Dnmt3aa
Tetraodon(ENSTNIP00000022331)
Fugu(ENSTRUP00000009049)
Medaka(ENSORLP00000022087)
Tilapia(ENSONIP00000006964)
Platyfish(XP_023203761.1)
Amazon_molly(ENSPFOP0000001500
Stickleback(ENSGACP00000009407
Cod(ENSGMOP00000019846)
Rainbow trout(XP_021470020.1)
Atlantic salmon(XM_014143600.1
Zebrafish(ENSDARP00000091093)
Golden line barbell(XM_0164978
Dnmt3ab
Tetraodon(ENSTNIP00000022492)
Fugu (ENSTRUP00000024460)
Stickleback(ENSGACP00000015064
Tilapia(ENSONIP00000001330)
Platyfish(ENSXMAP00000036171)
Amazon_molly(ENSPFOP0000001799
Cod(ENSGMOP00000020958)
Rainbow trout(XP_021429488.1)
Atlantic salmon(XM_014197582.1
Rainbow trout(XP_021440975.1)
Atlantic salmon(XM_014211429.1
Zebrafish(ENSDARP00000108609)
Golden line barbell(XM_0164754
DNMT3B
Human(ENSP00000328547)
Pig(ENSSSCP00000007722)
Mouse(ENSMUSP00000051830)
Rat(ENSRNOP00000015482)
Mouse(ENSMUSG00000082079)
Chinese hamster(ENSCGRP0000001
Rat(UCSC RGSC 5.0/rn5 chr3)
Chicken(ENSGALP00000052985)
Lizard(ENSACAP00000009682)
DNMT3C
Dnmt3ba
Elephant_shark(SINCAMP00000014
Coelacanth(ENSLACP00000009510)
Spotted gar(ENSLOCP00000006885
Tetraodon(ENSTNIP00000009615)
Fugu(ENSTRUP00000031152)
Stickleback(ENSGACP00000013579
Tilapia(ENSONIP00000018686)
Amazon_molly(ENSPFOP0000001143
Platyfish(ENSXMAP00000009160)
Medak(ENSORLP00000025108)
Cod(ENSGMOP00000016142)
Rainbow trout(XP_021471392.1)
Atlantic salmon(XM_014146292.1
Rainbow trout(XP_021422181.1)
Atlantic salmon(XM_014135215.1
Zebrafish(ENSDARP00000053417)
Golden line barbell(XM_0164651
CH domain
Dnmt3bb
Spotted gar(ENSLOCP00000006834 MAVNVRLTSDAGDKYDRVELLAWLNDSLQTK
Dnmt3bba
Tetraodon(ENSTNIP00000003297) PVGTPESVQKQSNGLHLVAWVNDLLKIN
Fugu(ENSTRUP00000012966) MASTPVETPQFIKKKSKGLHLVNWVNNLLKTD
Medaka(ENSORLP00000018521) MAGTEAVLVKWLNNLLKTN
Amazon_molly(ENSPFOP0000000632 MAGSPVVNPISTRDKSSRFYVVSWINRLLKTN
Platyfish(ENSXMAP00000011441) MAGSPVVTPNSIRDKSSRFYVVSWINRLLKTD
Stickleback(ENSGACP00000015822 MASAVVAIPDAIRDKSSRIYLVSWYNNLLKTN
Tilapia(ENSONIP00000021143) MESTVVRTSDSFTDENFYLKWINSLLKTN
Cod(ENSGMOP00000021057) MATNDIVLPDETQEKCSRYSLLRWLNNTLKVK
Rainbow trout(XP_021472161.1) MATNVDLDQTPDATAEKCSSFELLDWLNKTLDIK
Atlantic salmon (XM_014146676. MATNVDLDQTPDATAEKCSRFELLDWLNKTLDIK
Rainbow trout(XP_021422885.1) MATYVDPDQTPDATAEKCSRFEVLDWLNKILDIK
Atlantic salmon(XM_014136242.1 MATYVDPDQTPDATAEKCSRFEVLDWLNKILDIK
Zebrafish(ENSDARP00000046459) MATNVSLEPNNPDDKCSRYEVLGWINETLQTN
Golden line barbell(XM_0164740 MATNVSLDPNNPDDKCSRYEVLSWINETLKTN
Dnmt3bbb
Tetraodon (ENSTNIP00000009617)
Fugu(ENSTRUP00000030962)
Platyfish(ENSXMAP00000009128)
Stickleback(ENSGACP00000013567 VAVNVFLPKPTQLIPDDGLLAWLNDTLQTS
Atlantic salmon(XP_014001845.1
Rainbow trout (XP_021422185.1) MAVNVQVKPGEKHNRYELLAWLNESLQTR
Atlantic salmon(XM_014135212.1 MGRLYSRSELSIQYPGARGVSSWLGQTIDKQRVWDSENAMKRKGERGGGLEMAVNVQVKPGEKHNHYELLAWLNESLQTR
Zebrafish(ENSDARP00000074994) MVADVKIGDDKQSLCELLDWLNGLLQAT
Golden line barbell(XM_0164450 MVVNVKIGDEKHNLSELLDWLNHILHAT
Zebrafish(ENSDARP00000118597)
Golden line barbell(XM_0165118
DNM3A
Lamprey(ENSPMAT00000008525)
Lamprey(gi|1304463388|gb|PIZI0
Lamprey(ENSPMAT00000007628)
DNM3
Ciona_savignyi(ENSCSAVG0000000
Ciona_savignyi(ENSCSAVP0000002
European amphioxus(Sc0000092)

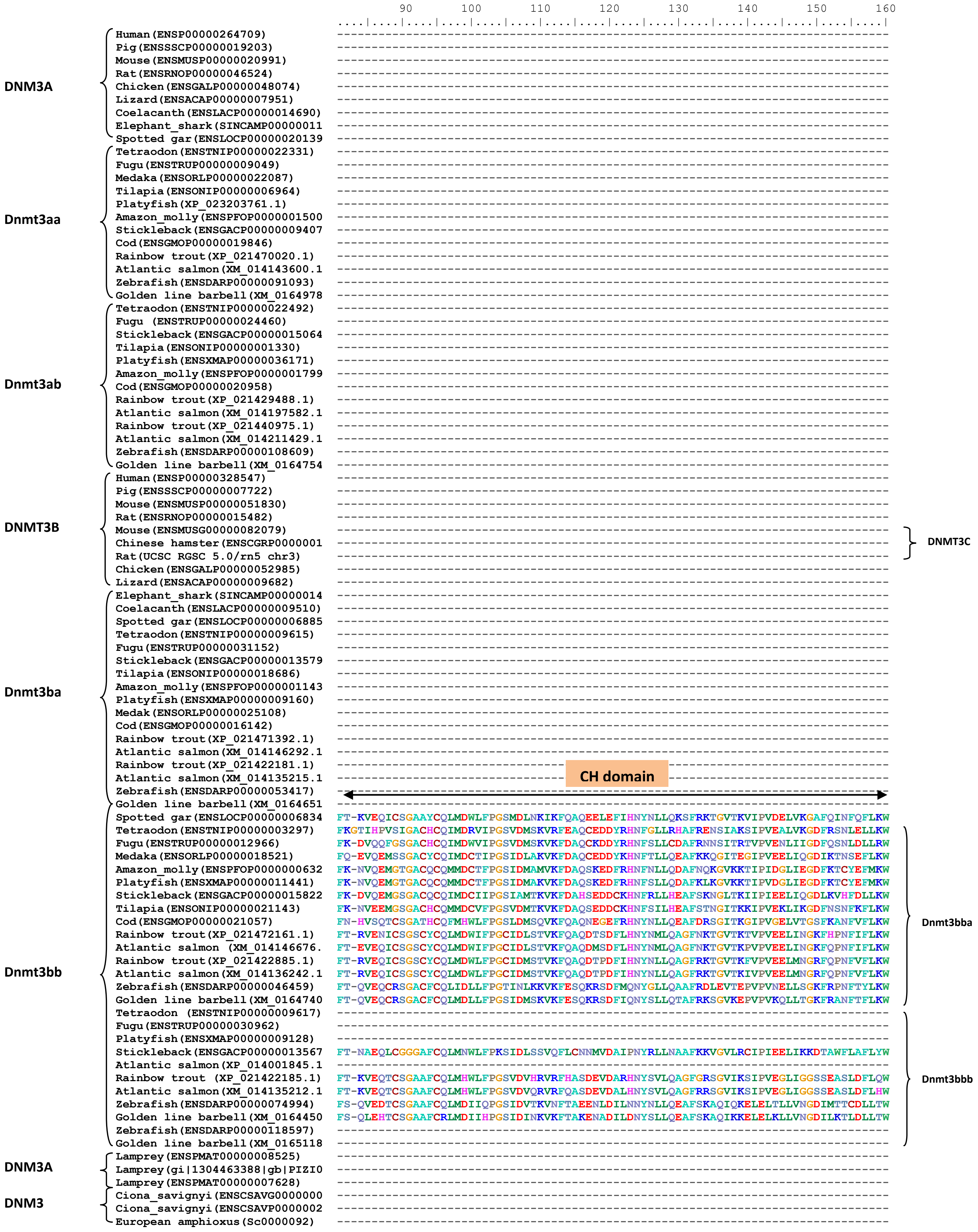

90 100 110 120 130 140 150 160
DNM3A
Human (ENSP00000264709)
Pig (ENSSSCP00000019203)
Mouse (ENSMUSP00000020991)
Rat (ENSRNOP00000046524)
Chicken (ENSGALP00000048074)
Lizard (ENSACAP00000007951)
Coelacanth (ENSLACP00000014690)
Elephant_shark (SINCAMP00000011
Spotted gar (ENSLOCP00000020139
Dnmt3aa
Tetraodon (ENSTNIP00000022331)
Fugu (ENSTRUP00000009049)
Medaka (ENSORLP00000022087)
Tilapia (ENSONIP00000006964)
Platyfish (XP_023203761.1)
Amazon_molly (ENSPFOP0000001500
Stickleback (ENSGACP00000009407
Cod (ENSGMOP00000019846)
Rainbow trout (XP_021470020.1)
Atlantic salmon (XM_014143600.1
Zebrafish (ENSDARP00000091093)
Golden line barbell (XM_0164978
Dnmt3ab
Tetraodon (ENSTNIP00000022492)
Fugu (ENSTRUP00000024460)
Stickleback (ENSGACP00000015064
Tilapia (ENSONIP00000001330)
Platyfish (ENSXMAP00000036171)
Amazon_molly (ENSPFOP0000001799
Cod (ENSGMOP00000020958)
Rainbow trout (XP_021429488.1)
Atlantic salmon (XM_014197582.1
Rainbow trout (XP_021440975.1)
Atlantic salmon (XM_014211429.1
Zebrafish (ENSDARP00000108609)
Golden line barbell (XM_0164754
DNMT3B
Human (ENSP00000328547)
Pig (ENSSSCP00000007722)
Mouse (ENSMUSP00000051830)
Rat (ENSRNOP00000015482)
Mouse (ENSMUSG00000082079)
Chinese hamster (ENSCGRP0000001
Rat (UCSC RGSC 5.0/rn5 chr3)
Chicken (ENSGALP00000052985)
Lizard (ENSACAP00000009682)
DNMT3C
Dnmt3ba
Elephant_shark (SINCAMP00000014
Coelacanth (ENSLACP00000009510)
Spotted gar (ENSLOCP00000006885
Tetraodon (ENSTNIP00000009615)
Fugu (ENSTRUP00000031152)
Stickleback (ENSGACP00000013579
Tilapia (ENSONIP00000018686)
Amazon_molly (ENSPFOP0000001143
Platyfish (ENSXMAP00000009160)
Medak (ENSORLP00000025108)
Cod (ENSGMOP00000016142)
Rainbow trout (XP_021471392.1)
Atlantic salmon (XM_014146292.1
Rainbow trout (XP_021422181.1)
Atlantic salmon (XM_014135215.1
Zebrafish (ENSDARP00000053417)
Golden line barbell (XM_0164651
CH domain
Dnmt3bb
Spotted gar (ENSLOCP00000006834 FT-KVEQICSGAAYCQLMDWLFPGSMDLNKIKFQAQEELEFIHNYNLLQKSFRKTGVTKVIPVDELVKGAFQINFQFLKW
Dnmt3bba
Tetraodon (ENSTNIP00000003297) FKGTIHPVSIGACHCQIMDRVIPGSVDMSKVRFEAQCEDDYRHNFGLLRHAFRENSIAKSIPVEALVKGDFRSNLELLKW
Fugu (ENSTRUP00000012966) FK-DVQQFGSGACHCQIMDWVIPGSVDMSKVKFDAQCKDDYRHNFSLLCDAFRNNSITRTVPVENLIIGDFQSNLDLLRW
Medaka (ENSORLP00000018521) FQ-EVQEMSSGACYCQIMDCTIPGSIDLAKVKFDAQCEDDYKHNFTLLQEAFKKQGITEGIPVEELIQGDIKTNSEFLKW
Amazon_molly (ENSPFOP0000000632 FK-NVQEMGTGACQCQMMDCTFPGSIDMAMVKFDAQSKEDFRHNFNLLQDAFNQKGVKKTIPIDGLIEGDFKTCYEFMKW
Platyfish (ENSXMAP00000011441) FK-NVQEMGTGACQCQMMDCTFPGSIDMAKVKFDAQSKEDFRHNFSLLQDAFKLKGVKKTIPVDGLIEGDFKTCYEFMKW
Stickleback (ENSGACP00000015822 FK-DVQEMGSGACQCQIMDCIIPGSIAMTKVKFDAHSEDDCKHNFRLLHEAFSKNGLTKIIPIEELIQGDLKVHFDLLKW
Tilapia (ENSONIP00000021143) FK-NVEEMGSGACHCQMMDCVFPGSVDMTKVKFDAQSEDDCKHNFSILHEAFSTNGITKKIPVEKLIKGDFNSNFKFLKW
Cod (ENSGMOP00000021057) FN-HVSQTCSGATHCQFMHWLFPGSLDMSQVKFQAQNEGEFRHNYNLLQEAFDRSGITKGIPVGELVTGSFKANFVFLKW
Rainbow trout (XP_021472161.1) FT-RVENICSGSCYCQLMDWIFPGCIDLSTVKFQAQDTSDFLHNYNMLQAGFNKTGVTKTVPVEELINGKFHPNFIFLKW
Atlantic salmon (XM_014146676. FT-EVEQICSGSCYCQLMDWIFPGCIDLSTVKFQAQDMSDFLHNYNMLQAGFNKTGVTKPVPVEELINGKFQPNFIFLKW
Rainbow trout (XP_021422885.1) FT-RVEQICSGSCYCQLMDWLFPGCIDMSTVKFQAQDTPDFIHNYNLLQAGFRKTGVTKFVPVEELMNGRFQPNFVFLKW
Atlantic salmon (XM_014136242.1 FT-RVEQICSGSCYCQLMDWLFPGCIDMSTVKFQAQDTPDFIHNYNLLQAGFRKTGVTKIVPVEELMNGRFQPNFVFLKW
Zebrafish (ENSDARP00000046459) FT-QVEQCRSGACFCQLIDLLFPGTINLKKVKFESQKRSDFMQNYGLLQAAFRDLEVTEPVPVNELLSGKFRPNFTYLKW
Golden line barbell (XM_0164740 FT-QVEQCRSGACFCQLMDLLFPGSIDMSKVKFESQKRSDFIQNYSLLQTAFRKSGVKEPVPVKQLLTGKFRANFTFLKW
Dnmt3bbb
Tetraodon (ENSTNIP00000009617)
Fugu (ENSTRUP00000030962)
Platyfish (ENSXMAP00000009128)
Stickleback (ENSGACP00000013567 FT-NAEQLCGGGAFCQLMNWLFPKSIDLSSVQFLCNNMVDAIPNYRLLNAAFKKVGVLRCIPIEELIKKDTAWFLAFLYW
Atlantic salmon (XP_014001845.1
Rainbow trout (XP_021422185.1) FT-KVEQTCSGAAFCQLMHWLFPGSVDVHRVRFHASDEVDARHNYSVLQAGFGRSGVIKSIPVEGLIGGSSEASLDFLQW
Atlantic salmon (XM_014135212.1 FT-KVEQTCSGAAFCQLMHWLFPGSVDVQRVRFQASDEVDALHNYSVLQAGFRRSGVIKSIPVEGLIGGSSEASLDFLHW
Zebrafish (ENSDARP00000074994) FS-QVEDTCSGAAFCQLMDIIQPGSIDVTKVNFTAEENLDILNNYNLLQEAFSKAQIQKELELTLLVNGDIMTTCDLLTW
Golden line barbell (XM_0164450 FS-QLEHTCSGAAFCRLMDIIHPGSIDINKVKFTAKENADILDNYSLLQEAFSKAQIKKELELKLLVNGDILKTLDLLTW
Zebrafish (ENSDARP00000118597)
Golden line barbell (XM_0165118
DNM3A
Lamprey (ENSPMAT00000008525)
Lamprey (gi|1304463388|gb|PIZI0
Lamprey (ENSPMAT00000007628)
DNM3
Ciona_savignyi (ENSCSAVG0000000
Ciona_savignyi (ENSCSAVP0000002
European amphioxus (Sc0000092)

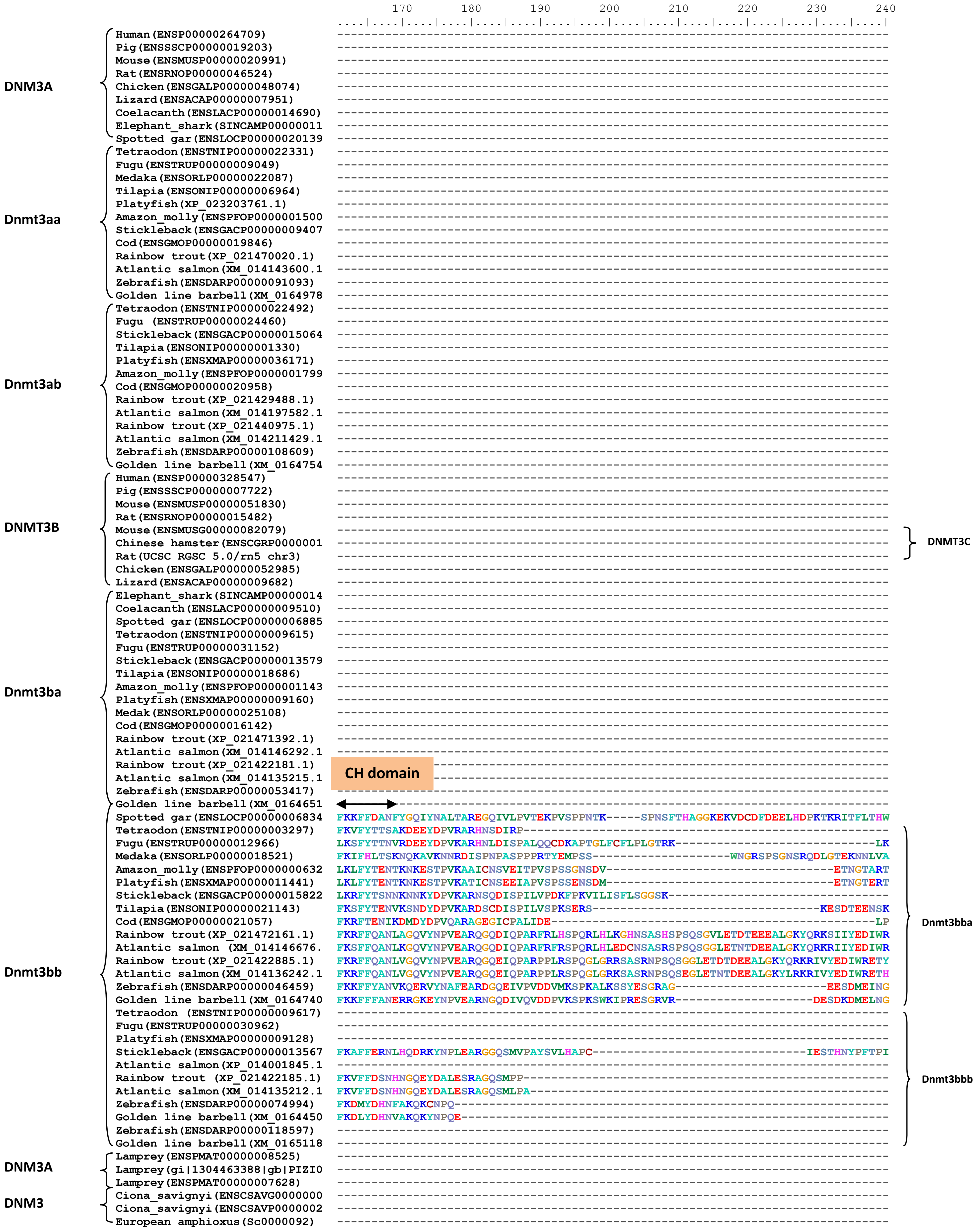
170 180 190 200 210 220 230 240
DNM3A
Human (ENSP00000264709)
Pig (ENSSSCP00000019203)
Mouse (ENSMUSP00000020991)
Rat (ENSRNOP00000046524)
Chicken (ENSGALP00000048074)
Lizard (ENSACAP00000007951)
Coelacanth (ENSLACP00000014690)
Elephant_shark (SINCAMP00000011
Spotted gar (ENSLOCP00000020139
Dnmt3aa
Tetraodon (ENSTNIP00000022331)
Fugu (ENSTRUP00000009049)
Medaka (ENSORLP00000022087)
Tilapia (ENSONIP00000006964)
Platyfish (XP_023203761.1)
Amazon_molly (ENSPFOP0000001500
Stickleback (ENSGACP00000009407
Cod (ENSGMOP00000019846)
Rainbow trout (XP_021470020.1)
Atlantic salmon (XM_014143600.1
Zebrafish (ENSDARP00000091093)
Golden line barbell (XM_0164978
Dnmt3ab
Tetraodon (ENSTNIP00000022492)
Fugu (ENSTRUP00000024460)
Stickleback (ENSGACP00000015064
Tilapia (ENSONIP00000001330)
Platyfish (ENSXMAP00000036171)
Amazon_molly (ENSPFOP0000001799
Cod (ENSGMOP00000020958)
Rainbow trout (XP_021429488.1)
Atlantic salmon (XM_014197582.1
Rainbow trout (XP_021440975.1)
Atlantic salmon (XM_014211429.1
Zebrafish (ENSDARP00000108609)
Golden line barbell (XM_0164754
DNMT3B
Human (ENSP00000328547)
Pig (ENSSSCP00000007722)
Mouse (ENSMUSP00000051830)
Rat (ENSRNOP00000015482)
Mouse (ENSMUSG00000082079)
Chinese hamster (ENSCGRP0000001
Rat (UCSC RGSC 5.0/rn5 chr3)
Chicken (ENSGALP00000052985)
Lizard (ENSACAP00000009682)
DNMT3C
Dnmt3ba
Elephant_shark (SINCAMP00000014
Coelacanth (ENSLACP00000009510)
Spotted gar (ENSLOCP00000006885
Tetraodon (ENSTNIP00000009615)
Fugu (ENSTRUP00000031152)
Stickleback (ENSGACP00000013579
Tilapia (ENSONIP00000018686)
Amazon_molly (ENSPFOP0000001143
Platyfish (ENSXMAP00000009160)
Medak (ENSORLP00000025108)
Cod (ENSGMOP00000016142)
Rainbow trout (XP_021471392.1)
Atlantic salmon (XM_014146292.1
Rainbow trout (XP_021422181.1)
Atlantic salmon (XM_014135215.1
Zebrafish (ENSDARP00000053417)
Golden line barbell (XM_0164651
CH domain
Dnmt3bb
Spotted gar (ENSLOCP00000006834 FKKFFDANFYGQIYNALTAREGQIVLPVTEKPVSPPNTK-----SPNSFTHAGGKEKVDCDFDEELHDPKTKRITFLTHW
Dnmt3bba
Tetraodon (ENSTNIP00000003297) FKVFYTTSAKDEEYDPVRARHNSDIRP
Fugu (ENSTRUP00000012966) LKSFYTTNVRDEEYDPVKARHNLDISPALQQCDKAPTGLFCFLPLGTRK-----LK
Medaka (ENSORLP00000018521) FKIFHLTSKNQKAVKNNRDISPNPASPPPRTYEMPSS-----WNGRSPSGNSRQDLGTEKNNLVA
Amazon_molly (ENSPFOP0000000632 LKLFYTENTKNKESTPVKAAICNSVEITPVSPSSGNSDV-----ETNGTART
Platyfish (ENSXMAP00000011441) LKLFYTENTKNKESTPVKATICNSEEIAPVSPSSENSDM-----ETNGTERT
Stickleback (ENSGACP00000015822 LKRFYTSNNKNNKYDPVKARNSQDISPILVPDKFPKVILISFLSGGSK
Tilapia (ENSONIP00000021143) FKSFYTENVKSNDYDPVKARDSCDISPILVSPKSERS-----KESDTEENSK
Cod (ENSGMOP00000021057) FKRFTENIKDMDYDPVQARAGEGICPALIDE-----LP
Rainbow trout (XP_021472161.1) FKRFFQANLAGQVYNPVEARQGQDIQPARFRLHSPQRLHLKGHNSASHSPSQSGVLETDTEEEALGKYQRKSIIYEDIWR
Atlantic salmon (XM_014146676. FKSFFQANLKGQVYNPVEARQGQDIQPARFRFRSPQRLHLEDCNSASRSPSQSGGLETNTDEEALGKYQRKRIIYEDIWR
Rainbow trout (XP_021422885.1) FKRFFQANLVGQVYNPVEARQGQEIQPARPPLRSPQGLGRRSASRNPSQSGGLETDTDEEALGKYQRKRIVYEDIWRETY
Atlantic salmon (XM_014136242.1 FKRFFQANLVGQVYNPVEARQGQEIQPARPPLRSPQGLGRKSASRNPSQSEGLETNTDEEALGKYLRKRIVYEDIWRETH
Zebrafish (ENSDARP00000046459) FKKFFYANVKQERVYNAFEARDGQEIVPVDDVMKSPKALKSSYESGRAG-----EESDMEING
Golden line barbell (XM_0164740 FKKFFFANERRGKEYNPVEARNGQDIVQVDDPVKSPKSWKIPRESGRVR-----DESDKDMELNG
Dnmt3bbb
Tetraodon (ENSTNIP00000009617)
Fugu (ENSTRUP00000030962)
Platyfish (ENSXMAP00000009128)
Stickleback (ENSGACP00000013567 FKAFFERNLHQDRKYNPLEARGGQSMVPAYSVLHAPC-----IESTHNYPFTPI
Atlantic salmon (XP_014001845.1
Rainbow trout (XP_021422185.1) FKVFFDSNHNGQEYDALESRAGQSMPP
Atlantic salmon (XM_014135212.1 FKVFFDSNHNGQEYDALESRAGQSMLPA
Zebrafish (ENSDARP00000074994) FKDMYDHNFAKQKCNPQ
Golden line barbell (XM_0164450 FKDLYDHNVAKQKYNPQE
Zebrafish (ENSDARP00000118597)
Golden line barbell (XM_0165118
DNM3A
Lamprey (ENSPMAT00000008525)
Lamprey (gi|1304463388|gb|PIZI0
Lamprey (ENSPMAT00000007628)
DNM3
Ciona_savignyi (ENSCSAVG0000000
Ciona_savignyi (ENSCSAVP0000002
European amphioxus (Sc0000092)

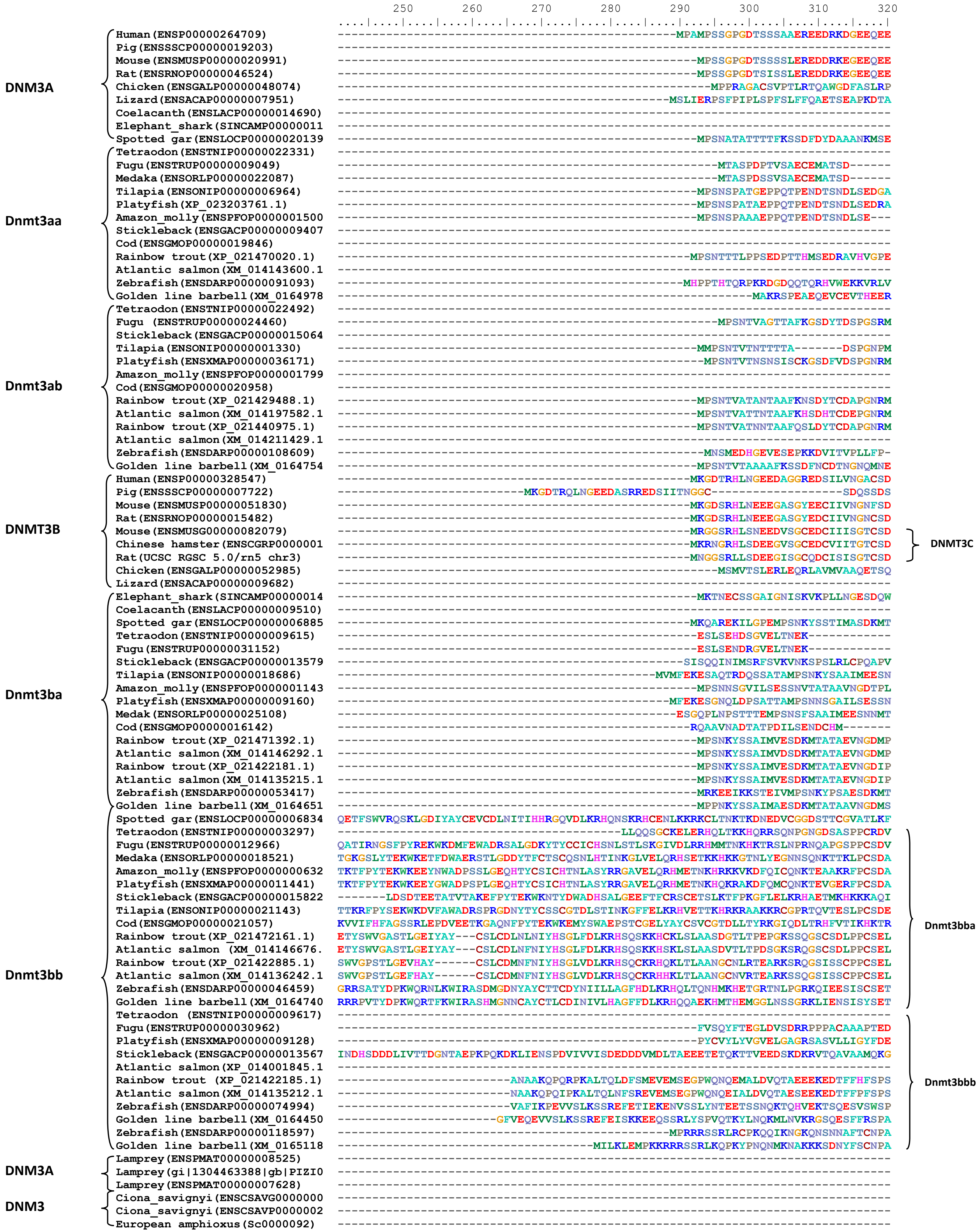

250
260
270
280
290
300
310
320
DNM3A
Human (ENSP00000264709)
Pig (ENSSSCP00000019203)
Mouse (ENSMUSP00000020991)
Rat (ENSRNOP00000046524)
Chicken (ENSGALP00000048074)
Lizard (ENSACAP00000007951)
Coelacanth (ENSLACP00000014690)
Elephant_shark (SINCAMP0000011
Spotted gar (ENSLOCP00000020139
Dnmt3aa
Tetraodon (ENSTNIP00000022331)
Fugu (ENSTRUP00000009049)
Medaka (ENSORLP00000022087)
Tilapia (ENSONIP00000006964)
Platyfish (XP_023203761.1)
Amazon_molly (ENSPFOP0000001500
Stickleback (ENSGACP00000009407
Cod (ENSGMOP00000019846)
Rainbow trout (XP_021470020.1)
Atlantic salmon (XM_014143600.1
Zebrafish (ENSDARP00000091093)
Golden line barbell (XM_0164978
Dnmt3ab
Tetraodon (ENSTNIP00000022492)
Fugu (ENSTRUP00000024460)
Stickleback (ENSGACP00000015064
Tilapia (ENSONIP00000001330)
Platyfish (ENSXMAP00000036171)
Amazon_molly (ENSPFOP0000001799
Cod (ENSGMOP00000020958)
Rainbow trout (XP_021429488.1)
Atlantic salmon (XM_014197582.1
Rainbow trout (XP_021440975.1)
Atlantic salmon (XM_014211429.1
Zebrafish (ENSDARP00000108609)
Golden line barbell (XM_0164754
DNMT3B
Human (ENSP00000328547)
Pig (ENSSSCP00000007722)
Mouse (ENSMUSP00000051830)
Rat (ENSRNOP00000015482)
Mouse (ENSMUSG00000082079)
Chinese hamster (ENSCGRP0000001
Rat (UCSC RGSC 5.0/rn5 chr3)
DNMT3C
Chicken (ENSGALP00000052985)
Lizard (ENSACAP00000009682)
Dnmt3ba
Elephant_shark (SINCAMP00000014
Coelacanth (ENSLACP00000009510)
Spotted gar (ENSLOCP00000006885
Tetraodon (ENSTNIP00000009615)
Fugu (ENSTRUP00000031152)
Stickleback (ENSGACP00000013579
Tilapia (ENSONIP00000018686)
Amazon_molly (ENSPFOP0000001143
Platyfish (ENSXMAP00000009160)
Medak (ENSORLP00000025108)
Cod (ENSGMOP00000016142)
Rainbow trout (XP_021471392.1)
Atlantic salmon (XM_014146292.1
Rainbow trout (XP_021422181.1)
Atlantic salmon (XM_014135215.1
Zebrafish (ENSDARP00000053417)
Golden line barbell (XM_0164651
Dnmt3bb
Spotted gar (ENSLOCP00000006834
Dnmt3bba
Tetraodon (ENSTNIP00000003297)
Fugu (ENSTRUP00000012966)
Medaka (ENSORLP00000018521)
Amazon_molly (ENSPFOP0000000632
Platyfish (ENSXMAP00000011441)
Stickleback (ENSGACP00000015822
Tilapia (ENSONIP00000021143)
Cod (ENSGMOP00000021057)
Rainbow trout (XP_021472161.1)
Atlantic salmon (XM_014146676.
Rainbow trout (XP_021422885.1)
Atlantic salmon (XM_014136242.1
Zebrafish (ENSDARP00000046459)
Golden line barbell (XM_0164740
Dnmt3bbb
Tetraodon (ENSTNIP00000009617)
Fugu (ENSTRUP00000030962)
Platyfish (ENSXMAP00000009128)
Stickleback (ENSGACP00000013567
Atlantic salmon (XP_014001845.1
Rainbow trout (XP_021422185.1)
Atlantic salmon (XP_014135212.1
Zebrafish (ENSDARP00000074994)
Golden line barbell (XM_0164450
Zebrafish (ENSDARP00000118597)
Golden line barbell (XM_0165118
DNM3A
Lamprey (ENSPMAT00000008525)
Lamprey (gi|1304463388|gb|PIZI0
Lamprey (ENSPMAT00000007628)
DNM3
Ciona_savignyi (ENSCSAVG0000000
Ciona_savignyi (ENSCSAVP0000002
European amphioxus (Sc0000092)

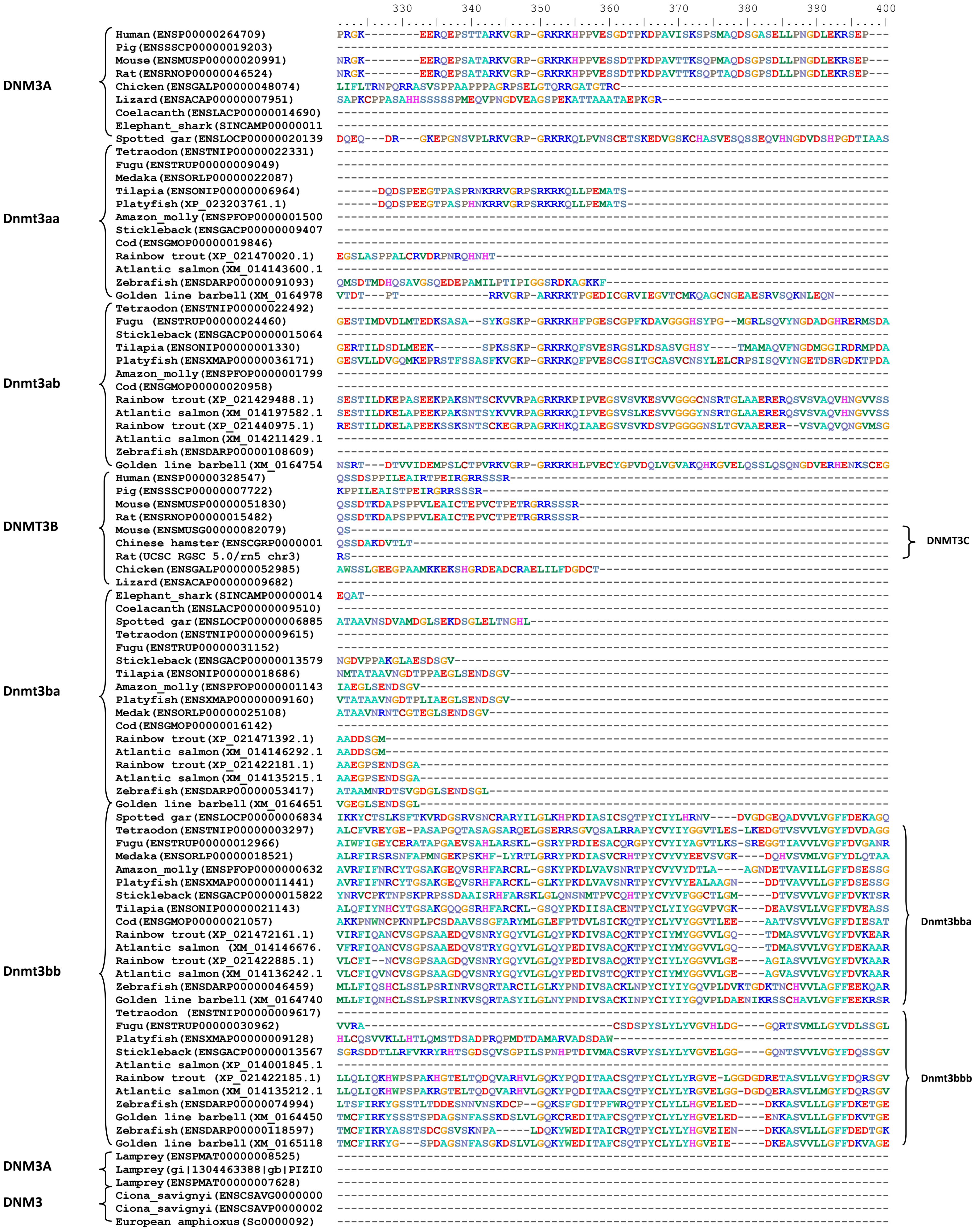

330 340 350 360 370 380 390 400
DNM3A
Human(ENSP00000264709)
Pig(ENSSSCP00000019203)
Mouse(ENSMUSP00000020991)
Rat(ENSRNOP00000046524)
Chicken(ENSGALP00000048074)
Lizard(ENSACAP00000007951)
Coelacanth(ENSLACP00000014690)
Elephant_shark(SINCAMP00000011
Spotted gar(ENSLOCP00000020139
Dnmt3aa
Tetraodon(ENSTNIP00000022331)
Fugu(ENSTRUP00000009049)
Medaka(ENSORLP00000022087)
Tilapia(ENSONIP00000006964)
Platyfish(XP_023203761.1)
Amazon_molly(ENSPFOP0000001500
Stickleback(ENSGACP00000009407
Cod(ENSGMOP00000019846)
Rainbow trout(XP_021470020.1)
Atlantic salmon(XM_014143600.1
Zebrafish(ENSDARP00000091093)
Golden line barbell(XM_0164978
Dnmt3ab
Tetraodon(ENSTNIP00000022492)
Fugu (ENSTRUP00000024460)
Stickleback(ENSGACP00000015064
Tilapia(ENSONIP00000001330)
Platyfish(ENSXMAP00000036171)
Amazon_molly(ENSPFOP0000001799
Cod(ENSGMOP00000020958)
Rainbow trout(XP_021429488.1)
Atlantic salmon(XM_014197582.1
Rainbow trout(XP_021440975.1)
Atlantic salmon(XM_014211429.1
Zebrafish(ENSDARP00000108609)
Golden line barbell(XM_0164754
DNMT3B
Human(ENSP00000328547)
Pig(ENSSSCP00000007722)
Mouse(ENSMUSP00000051830)
Rat(ENSRNOP00000015482)
Mouse(ENSMUSG00000082079)
Chinese hamster(ENSCGRP0000001
Rat(UCSC RGSC 5.0/rn5 chr3)
Chicken(ENSGALP00000052985)
Lizard(ENSACAP00000009682)
DNMT3C
Dnmt3ba
Elephant_shark(SINCAMP00000014
Coelacanth(ENSLACP00000009510)
Spotted gar(ENSLOCP00000006885
Tetraodon(ENSTNIP00000009615)
Fugu(ENSTRUP00000031152)
Stickleback(ENSGACP00000013579
Tilapia(ENSONIP00000018686)
Amazon_molly(ENSPFOP0000001143
Platyfish(ENSXMAP00000009160)
Medak(ENSORLP00000025108)
Cod(ENSGMOP00000016142)
Rainbow trout(XP_021471392.1)
Atlantic salmon(XM_014146292.1
Rainbow trout(XP_021422181.1)
Atlantic salmon(XM_014135215.1
Zebrafish(ENSDARP00000053417)
Golden line barbell(XM_0164651
Dnmt3bb
Spotted gar(ENSLOCP00000006834
Tetraodon(ENSTNIP00000003297)
Fugu(ENSTRUP00000012966)
Medaka(ENSORLP00000018521)
Amazon_molly(ENSPFOP0000000632
Platyfish(ENSXMAP00000011441)
Stickleback(ENSGACP00000015822
Tilapia(ENSONIP00000021143)
Cod(ENSGMOP00000021057)
Rainbow trout(XP_021472161.1)
Atlantic salmon (XM_014146676.
Rainbow trout(XP_021422885.1)
Atlantic salmon(XM_014136242.1
Zebrafish(ENSDARP00000046459)
Golden line barbell(XM_0164740
Dnmt3bba
Tetraodon (ENSTNIP00000009617)
Fugu(ENSTRUP00000030962)
Platyfish(ENSXMAP00000009128)
Stickleback(ENSGACP00000013567
Atlantic salmon(XP_014001845.1
Rainbow trout (XP_021422185.1)
Atlantic salmon(XM_014135212.1
Zebrafish(ENSDARP00000074994)
Golden line barbell(XM_0164450
Zebrafish(ENSDARP00000118597)
Golden line barbell(XM_0165118
Dnmt3bbb
DNM3A
Lamprey(ENSPMAT00000008525)
Lamprey(gi|1304463388|gb|PIZI0
Lamprey(ENSPMAT00000007628)
DNM3
Ciona_savignyi(ENSCSAVG0000000
Ciona_savignyi(ENSCSAVP0000002
European amphioxus(Sc0000092)

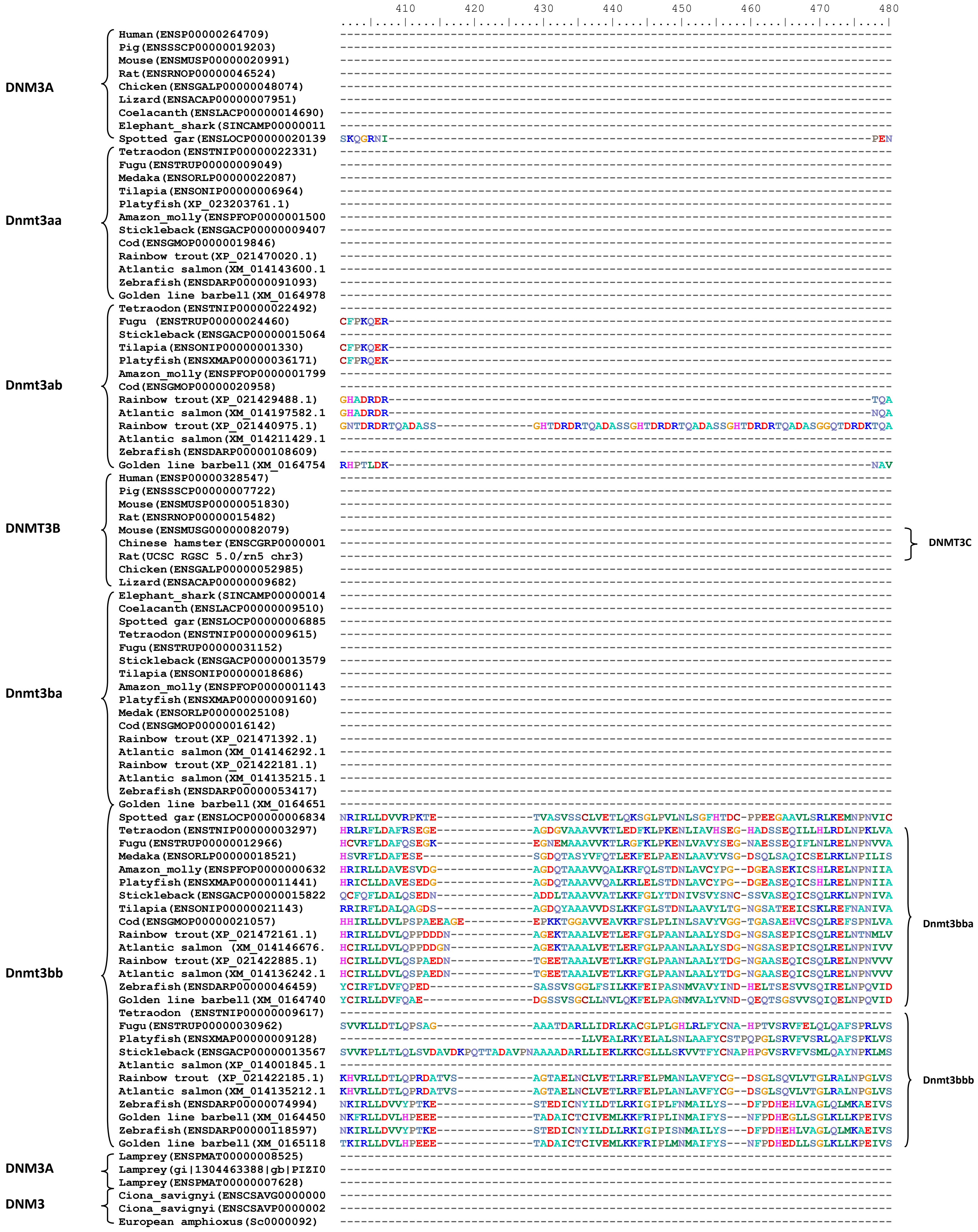

DNM3A
Human(ENSP00000264709)
Pig(ENSSSCP00000019203)
Mouse(ENSMUSP00000020991)
Rat(ENSRNOP00000046524)
Chicken(ENSGALP00000048074)
Lizard(ENSACAP00000007951)
Coelacanth(ENSLACP00000014690)
Elephant_shark(SINCAMP00000011
Spotted gar(ENSLOCP00000020139
Dnmt3aa
Tetraodon(ENSTNIP00000022331)
Fugu(ENSTRUP00000009049)
Medaka(ENSORLP00000022087)
Tilapia(ENSONIP00000006964)
Platyfish(XP_023203761.1)
Amazon_molly(ENSPFOP0000001500
Stickleback(ENSGACP00000009407
Cod(ENSGMOP00000019846)
Rainbow trout(XP_021470020.1)
Atlantic salmon(XM_014143600.1
Zebrafish(ENSDARP00000091093)
Golden line barbell(XM_0164978
Dnmt3ab
Tetraodon(ENSTNIP00000022492)
Fugu (ENSTRUP00000024460)
Stickleback(ENSGACP00000015064
Tilapia(ENSONIP00000001330)
Platyfish(ENSXMAP00000036171)
Amazon_molly(ENSPFOP0000001799
Cod(ENSGMOP00000020958)
Rainbow trout(XP_021429488.1)
Atlantic salmon(XM_014197582.1
Rainbow trout(XP_021440975.1)
Atlantic salmon(XM_014211429.1
Zebrafish(ENSDARP00000108609)
Golden line barbell(XM_0164754
DNMT3B
Human(ENSP00000328547)
Pig(ENSSSCP00000007722)
Mouse(ENSMUSP00000051830)
Rat(ENSRNOP00000015482)
Mouse(ENSMUSG00000082079)
Chinese hamster(ENSCGRP0000001
Rat(UCSC RGSC 5.0/rn5 chr3)
Chicken(ENSGALP00000052985)
Lizard(ENSACAP00000009682)
DNMT3C
Dnmt3ba
Elephant_shark(SINCAMP00000014
Coelacanth(ENSLACP00000009510)
Spotted gar(ENSLOCP00000006885
Tetraodon(ENSTNIP00000009615)
Fugu(ENSTRUP00000031152)
Stickleback(ENSGACP00000013579
Tilapia(ENSONIP00000018686)
Amazon_molly(ENSPFOP0000001143
Platyfish(ENSXMAP00000009160)
Medak(ENSORLP00000025108)
Cod(ENSGMOP00000016142)
Rainbow trout(XP_021471392.1)
Atlantic salmon(XM_014146292.1
Rainbow trout(XP_021422181.1)
Atlantic salmon(XM_014135215.1
Zebrafish(ENSDARP00000053417)
Golden line barbell(XM_0164651
Dnmt3bb
Spotted gar(ENSLOCP00000006834
Tetraodon(ENSTNIP00000003297)
Fugu(ENSTRUP00000012966)
Medaka(ENSORLP00000018521)
Amazon_molly(ENSPFOP0000000632
Platyfish(ENSXMAP00000011441)
Stickleback(ENSGACP00000015822
Tilapia(ENSONIP00000021143)
Cod(ENSGMOP00000021057)
Rainbow trout(XP_021472161.1)
Atlantic salmon (XM_014146676.
Rainbow trout(XP_021422885.1)
Atlantic salmon(XM_014136242.1
Zebrafish(ENSDARP00000046459)
Golden line barbell(XM_0164740
Dnmt3bba
Tetraodon (ENSTNIP00000009617)
Fugu(ENSTRUP00000030962)
Platyfish(ENSXMAP00000009128)
Stickleback(ENSGACP00000013567
Atlantic salmon(XP_014001845.1
Rainbow trout (XP_021422185.1)
Atlantic salmon(XM_014135212.1
Zebrafish(ENSDARP00000074994)
Golden line barbell(XM_0164450
Zebrafish(ENSDARP00000118597)
Golden line barbell(XM_0165118
Dnmt3bbb
DNM3A
Lamprey(ENSPMAT00000008525)
Lamprey(gi|1304463388|gb|PIZI0
Lamprey(ENSPMAT00000007628)
DNM3
Ciona_savignyi(ENSCSAVG0000000
Ciona_savignyi(ENSCSAVP0000002
European amphioxus(Sc0000092)
410 420 430 440 450 460 470 480

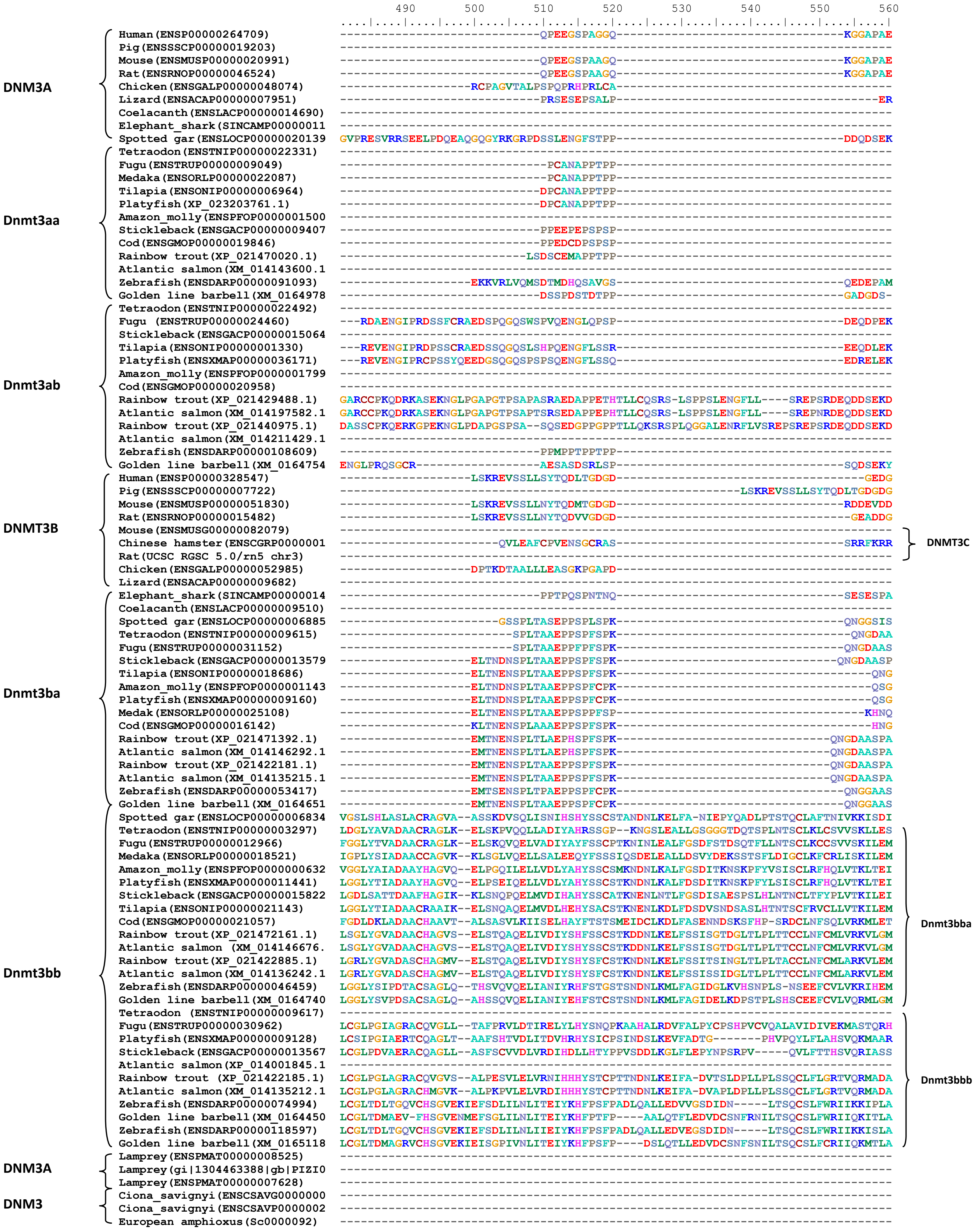
490
500
510
520
530
540
550
560
DNM3A
Human(ENSP00000264709)
Pig(ENSSSCP00000019203)
Mouse(ENSMUSP00000020991)
Rat(ENSRNOP00000046524)
Chicken(ENSGALP00000048074)
Lizard(ENSACAP00000007951)
Coelacanth(ENSLACP00000014690)
Elephant_shark(SINCAMP00000011
Spotted gar(ENSLOCP00000020139
Dnmt3aa
Tetraodon(ENSTNIP00000022331)
Fugu(ENSTRUP00000009049)
Medaka(ENSORLP00000022087)
Tilapia(ENSONIP00000006964)
Platyfish(XP_023203761.1)
Amazon_molly(ENSPFOP0000001500
Stickleback(ENSGACP00000009407
Cod(ENSGMOP00000019846)
Rainbow trout(XP_021470020.1)
Atlantic salmon(XM_014143600.1
Zebrafish(ENSDARP00000091093)
Golden line barbell(XM_0164978
Dnmt3ab
Tetraodon(ENSTNIP00000022492)
Fugu (ENSTRUP00000024460)
Stickleback(ENSGACP00000015064
Tilapia(ENSONIP00000001330)
Platyfish(ENSXMAP00000036171)
Amazon_molly(ENSPFOP0000001799
Cod(ENSGMOP00000020958)
Rainbow trout(XP_021429488.1)
Atlantic salmon(XM_014197582.1
Rainbow trout(XP_021440975.1)
Atlantic salmon(XM_014211429.1
Zebrafish(ENSDARP00000108609)
Golden line barbell(XM_0164754
DNMT3B
Human(ENSP00000328547)
Pig(ENSSSCP00000007722)
Mouse(ENSMUSP00000051830)
Rat(ENSRNOP00000015482)
Mouse(ENSMUSG00000082079)
Chinese hamster(ENSCGRP0000001
Rat(UCSC RGSC 5.0/rn5 chr3)
DNMT3C
Chicken(ENSGALP00000052985)
Lizard(ENSACAP00000009682)
Dnmt3ba
Elephant_shark(SINCAMP00000014
Coelacanth(ENSLACP00000009510)
Spotted gar(ENSLOCP00000006885
Tetraodon(ENSTNIP00000009615)
Fugu(ENSTRUP00000031152)
Stickleback(ENSGACP00000013579
Tilapia(ENSONIP00000018686)
Amazon_molly(ENSPFOP0000001143
Platyfish(ENSXMAP00000009160)
Medak(ENSORLP00000025108)
Cod(ENSGMOP00000016142)
Rainbow trout(XP_021471392.1)
Atlantic salmon(XM_014146292.1
Rainbow trout(XP_021422181.1)
Atlantic salmon(XM_014135215.1
Zebrafish(ENSDARP00000053417)
Golden line barbell(XM_0164651
Dnmt3bb
Spotted gar(ENSLOCP00000006834
Dnmt3bba
Tetraodon(ENSTNIP00000003297)
Fugu(ENSTRUP00000012966)
Medaka(ENSORLP00000018521)
Amazon_molly(ENSPFOP0000000632
Platyfish(ENSXMAP00000011441)
Stickleback(ENSGACP00000015822
Tilapia(ENSONIP00000021143)
Cod(ENSGMOP00000021057)
Rainbow trout(XP_021472161.1)
Atlantic salmon (XM_014146676.
Rainbow trout(XP_021422885.1)
Atlantic salmon(XM_014136242.1
Zebrafish(ENSDARP00000046459)
Golden line barbell(XM_0164740
Dnmt3bbb
Tetraodon (ENSTNIP00000009617)
Fugu(ENSTRUP00000030962)
Platyfish(ENSXMAP00000009128)
Stickleback(ENSGACP00000013567
Atlantic salmon(XP_014001845.1
Rainbow trout (XP_021422185.1)
Atlantic salmon(XM_014135212.1
Zebrafish(ENSDARP00000074994)
Golden line barbell(XM_0164450
Zebrafish(ENSDARP00000118597)
Golden line barbell(XM_0165118
DNM3A
Lamprey(ENSPMAT00000008525)
Lamprey(gi|1304463388|gb|PIZI0
Lamprey(ENSPMAT00000007628)
DNM3
Ciona_savignyi(ENSCSAVG0000000
Ciona_savignyi(ENSCSAVP0000002
European amphioxus(Sc0000092)

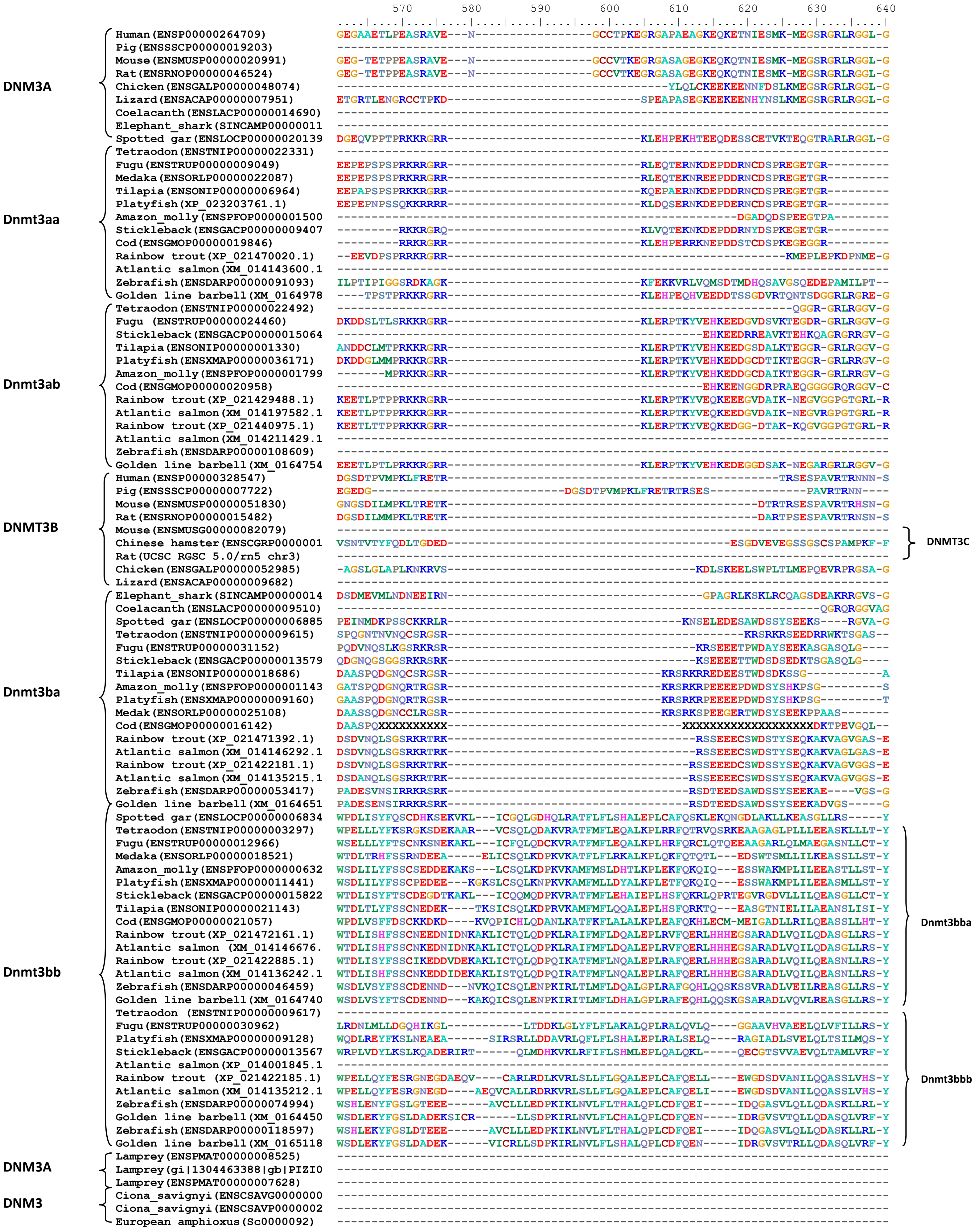
570 580 590 600 610 620 630 640
DNM3A
Human(ENSP00000264709)
Pig(ENSSSCP00000019203)
Mouse(ENSMUSP00000020991)
Rat(ENSRNOP00000046524)
Chicken(ENSGALP00000048074)
Lizard(ENSACAP00000007951)
Coelacanth(ENSLACP00000014690)
Elephant_shark(SINCAMP0000011
Spotted gar(ENSLOCP00000020139
Dnmt3aa
Tetraodon(ENSTNIP00000022331)
Fugu(ENSTRUP00000009049)
Medaka(ENSORLP00000022087)
Tilapia(ENSONIP00000006964)
Platyfish(XP_023203761.1)
Amazon_molly(ENSPFOP0000001500
Stickleback(ENSGACP00000009407
Cod(ENSGMOP00000019846)
Rainbow trout(XP_021470020.1)
Atlantic salmon(XM_014143600.1
Zebrafish(ENSDARP00000091093)
Golden line barbell(XM_0164978
Dnmt3ab
Tetraodon(ENSTNIP00000022492)
Fugu (ENSTRUP00000024460)
Stickleback(ENSGACP00000015064
Tilapia(ENSONIP00000001330)
Platyfish(ENSXMAP00000036171)
Amazon_molly(ENSPFOP0000001799
Cod(ENSGMOP00000020958)
Rainbow trout(XP_021429488.1)
Atlantic salmon(XM_014197582.1
Rainbow trout(XP_021440975.1)
Atlantic salmon(XM_014211429.1
Zebrafish(ENSDARP00000108609)
Golden line barbell(XM_0164754
DNMT3B
Human(ENSP00000328547)
Pig(ENSSSCP00000007722)
Mouse(ENSMUSP00000051830)
Rat(ENSRNOP00000015482)
Mouse(ENSMUSG00000082079)
Chinese hamster(ENSCGRP0000001
Rat(UCSC RGSC 5.0/rn5 chr3)
DNMT3C
Chicken(ENSGALP00000052985)
Lizard(ENSACAP00000009682)
Dnmt3ba
Elephant_shark(SINCAMP00000014
Coelacanth(ENSLACP00000009510)
Spotted gar(ENSLOCP00000006885
Tetraodon(ENSTNIP00000009615)
Fugu(ENSTRUP00000031152)
Stickleback(ENSGACP00000013579
Tilapia(ENSONIP00000018686)
Amazon_molly(ENSPFOP0000001143
Platyfish(ENSXMAP00000009160)
Medak(ENSORLP00000025108)
Cod(ENSGMOP00000016142)
Rainbow trout(XP_021471392.1)
Atlantic salmon(XM_014146292.1
Rainbow trout(XP_021422181.1)
Atlantic salmon(XM_014135215.1
Zebrafish(ENSDARP00000053417)
Golden line barbell(XM_0164651
Dnmt3bb
Spotted gar(ENSLOCP00000006834
Dnmt3bba
Tetraodon(ENSTNIP00000003297)
Fugu(ENSTRUP00000012966)
Medaka(ENSORLP00000018521)
Amazon_molly(ENSPFOP0000000632
Platyfish(ENSXMAP00000011441)
Stickleback(ENSGACP00000015822
Tilapia(ENSONIP00000021143)
Cod(ENSGMOP00000021057)
Rainbow trout(XP_021472161.1)
Atlantic salmon (XM_014146676.
Rainbow trout(XP_021422885.1)
Atlantic salmon(XM_014136242.1
Zebrafish(ENSDARP00000046459)
Golden line barbell(XM_0164740
Dnmt3bbb
Tetraodon (ENSTNIP00000009617)
Fugu(ENSTRUP00000030962)
Platyfish(ENSXMAP00000009128)
Stickleback(ENSGACP00000013567
Atlantic salmon(XP_014001845.1
Rainbow trout (XP_021422185.1)
Atlantic salmon(XM_014135212.1
Zebrafish(ENSDARP00000074994)
Golden line barbell(XM_0164450
Zebrafish(ENSDARP00000118597)
Golden line barbell(XM_0165118
DNM3A
Lamprey(ENSPMAT00000008525)
Lamprey(gi|1304463388|gb|PIZI0
Lamprey(ENSPMAT00000007628)
DNM3
Ciona_savignyi(ENSCSAVG0000000
Ciona_savignyi(ENSCSAVP0000002
European amphioxus(Sc0000092)

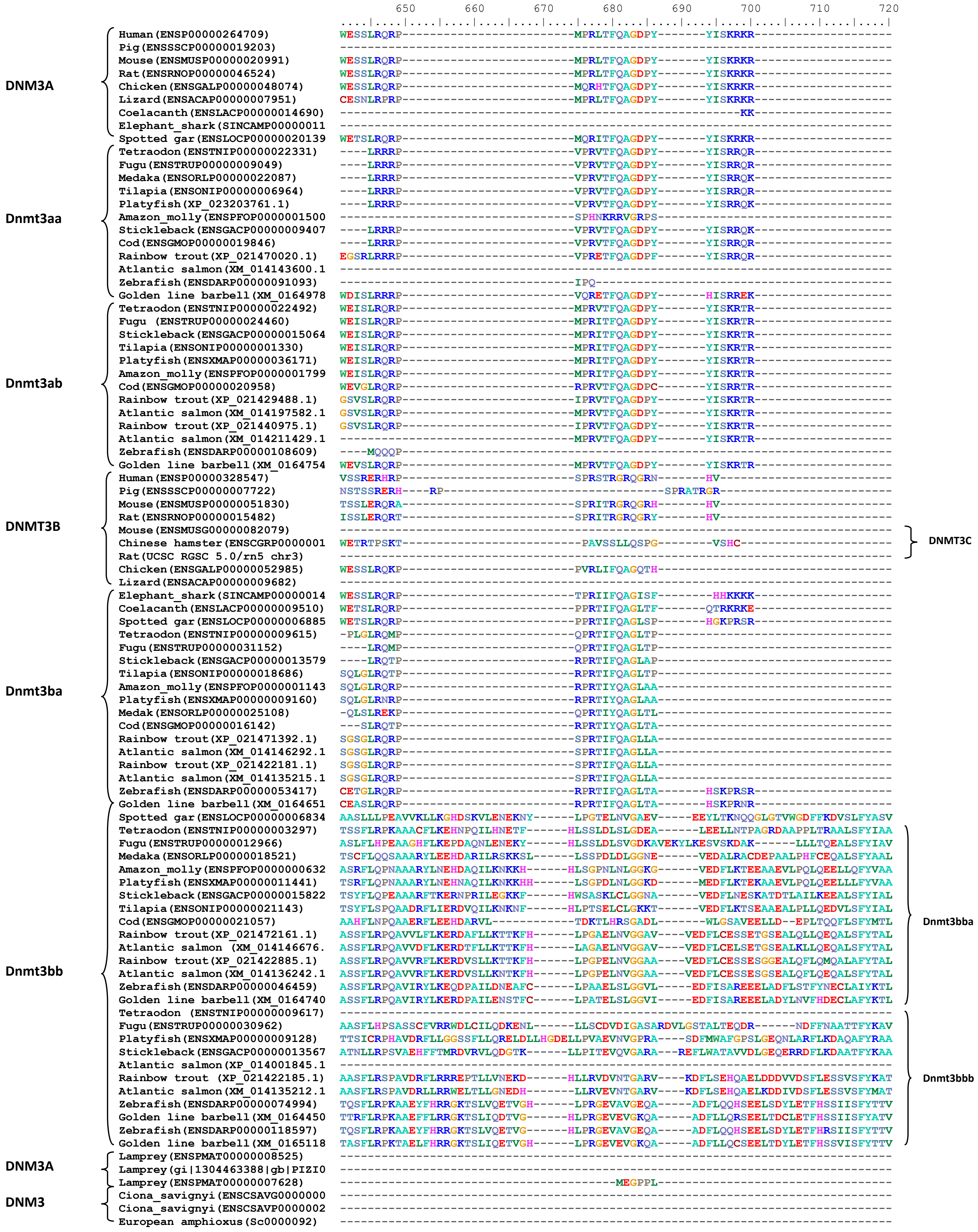

650 660 670 680 690 700 710 720
DNM3A
Human(ENSP00000264709) WESSLRQRP-------------------------MPRLTFQAGDPY-------YISKRKR--------------------
Pig(ENSSSCP00000019203) --
Mouse(ENSMUSP00000020991) WESSLRQRP-------------------------MPRLTFQAGDPY-------YISKRKR--------------------
Rat(ENSRNOP00000046524) WESSLRQRP-------------------------MPRLTFQAGDPY-------YISKRKR--------------------
Chicken(ENSGALP00000048074) WESSLRQRP-------------------------MQRHTFQAGDPY-------YISKRKR--------------------
Lizard(ENSACAP00000007951) CESNLRPRP-------------------------MPRLTFQAGDPY-------YISKRKR--------------------
Coelacanth(ENSLACP00000014690) --KK----------------------
Elephant_shark(SINCAMP00000011 --
Spotted gar(ENSLOCP00000020139 WETSLRQRP-------------------------MQRITFQAGDPY-------YISKRKR--------------------
Dnmt3aa
Tetraodon(ENSTNIP00000022331) ----LRRRP-------------------------VPRVTFQAGDPY-------YISRRQR--------------------
Fugu(ENSTRUP00000009049) ----LRRRP-------------------------VPRVTFQAGDPY-------YISRRQR--------------------
Medaka(ENSORLP00000022087) ----LRRRP-------------------------VPRVTFQAGDPY-------YISRRQK--------------------
Tilapia(ENSONIP00000006964) ----LRRRP-------------------------VPRVTFQAGDPY-------YISRRQR--------------------
Platyfish(XP_023203761.1) ----LRRRP-------------------------VPRVTFQAGDPY-------YISRRQK--------------------
Amazon_molly(ENSPFOP0000001500 ----------------------------------SPHNKRRVGRPS--------------------------------------
Stickleback(ENSGACP00000009407 ----LRRRP-------------------------VPRVTFQAGDPY-------YISRRQK--------------------
Cod(ENSGMOP00000019846) ----LRRRP-------------------------VPRVTFQAGDPY-------YISRRQR--------------------
Rainbow trout(XP_021470020.1) EGSRLRRRP-------------------------VPRETFQAGDPF-------YISRRQR--------------------
Atlantic salmon(XM_014143600.1 --
Zebrafish(ENSDARP00000091093) ----------------------------------IPQ---
Golden line barbell(XM_0164978 WDISLRRRP-------------------------VQRETFQAGDPY-------HISRREK--------------------
Dnmt3ab
Tetraodon(ENSTNIP00000022492) WEISLRQRP-------------------------MPRVTFQAGDPY-------YISKRTR--------------------
Fugu (ENSTRUP00000024460) WEISLRQRP-------------------------MPRITFQAGDPY-------YISKRTR--------------------
Stickleback(ENSGACP00000015064 WEISLRQRP-------------------------MPRITFQAGDPY-------YISKRTR--------------------
Tilapia(ENSONIP00000001330) WEISLRQRP-------------------------MPRITFQAGDPY-------YISKRTR--------------------
Platyfish(ENSXMAP00000036171) WEISLRQRP-------------------------MPRITFQAGDPY-------YISKRTR--------------------
Amazon_molly(ENSPFOP0000001799 WEISLRQRP-------------------------MPRITFQAGDPY-------YISKRTR--------------------
Cod(ENSGMOP00000020958) WEVGLRQRP-------------------------RPRVTFQAGDPC-------YISRRTR--------------------
Rainbow trout(XP_021429488.1) GSVSLRQRP-------------------------IPRVTFQAGDPY-------YISKRTR--------------------
Atlantic salmon(XM_014197582.1 GSVSLRQRP-------------------------MPRVTFQAGDPY-------YISKRTR--------------------
Rainbow trout(XP_021440975.1) GSVSLRQRP-------------------------IPRVTFQAGDPY-------YISKRTR--------------------
Atlantic salmon(XM_014211429.1 ----------------------------------MPRVTFQAGDPY-------YISKRTR--------------------
Zebrafish(ENSDARP00000108609) ----MQQQP---
Golden line barbell(XM_0164754 WEVSLRQRP-------------------------MPRVTFQAGDPY-------YISKRTR--------------------
DNMT3B
Human(ENSP00000328547) VSSRERHRP-------------------------SPRSTRGRQGRN-------HV-------------------------
Pig(ENSSSCP00000007722) NSTSSRERH----RP-------------------------------SPRATRGR--------------------------
Mouse(ENSMUSP00000051830) TSSLERQRA-------------------------SPRITRGRQGRH-------HV-------------------------
Rat(ENSRNOP00000015482) ISSLERQRT-------------------------SPRITRGRQGRY-------HV-------------------------
Mouse(ENSMUSG00000082079) --
Chinese hamster(ENSCGRP0000001 WETRTPSKT--------------------------PAVSSLLQSPG--------VSHC-----------------------
Rat(UCSC RGSC 5.0/rn5 chr3) --
DNMT3C
Chicken(ENSGALP00000052985) WESSLRQKP-------------------------PVRLIFQAGQTH--------------------------------------
Lizard(ENSACAP00000009682) --
Dnmt3ba
Elephant_shark(SINCAMP00000014 WESSLRQRP-------------------------TPRIIFQAGISF--------HHKKKK--------------------
Coelacanth(ENSLACP00000009510) WETSLRQRP-------------------------PPRTIFQAGLTF-------QTRKRKE--------------------
Spotted gar(ENSLOCP00000006885 WETSLRQRP-------------------------PPRTIFQAGLSP-------HGKPRSR--------------------
Tetraodon(ENSTNIP00000009615) -PLGLRQMP-------------------------QPRTIFQAGLTP--------------------------------------
Fugu(ENSTRUP00000031152) ----LRQMP-------------------------QPRTIFQAGLTP--------------------------------------
Stickleback(ENSGACP00000013579 ----LRQTP-------------------------RPRTIFQAGLAP--------------------------------------
Tilapia(ENSONIP00000018686) SQLGLRQTP-------------------------RPRTIFQAGLTP--------------------------------------
Amazon_molly(ENSPFOP0000001143 SQLGLRQRP-------------------------RPRTIYQAGLAA--------------------------------------
Platyfish(ENSXMAP00000009160) SQLGLRNRP-------------------------RPRTIYQAGLAA--------------------------------------
Medak(ENSORLP00000025108) -QLSLREKP-------------------------QPRTIYQAGLTL--------------------------------------
Cod(ENSGMOP00000016142) ---SLRQTP-------------------------RPRTIYQAGLTA--------------------------------------
Rainbow trout(XP_021471392.1) SGSGLRQRP-------------------------SPRTIFQAGLLA--------------------------------------
Atlantic salmon(XM_014146292.1 SGSGLRQRP-------------------------SPRTIFQAGLLA--------------------------------------
Rainbow trout(XP_021422181.1) SGSGLRQRP-------------------------SPRTIFQAGLLA--------------------------------------
Atlantic salmon(XM_014135215.1 SGSGLRQRP-------------------------SPRTIFQAGLLA--------------------------------------
Zebrafish(ENSDARP00000053417) CETGLRQRP-------------------------RPRTIFQAGLTA-------HSKPRSR--------------------
Golden line barbell(XM_0164651 CEASLRQRP-------------------------RPRTIFQAGLTA-------HSKPRNR--------------------
Dnmt3bb
Spotted gar(ENSLOCP00000006834 AASLLLPEAVVKLLKGHDSKVLENEKNY------LPGTELNVGAEV-----EEYLTKNQQGLGTVWGDFFKDVSLFYASV
Dnmt3bba
Tetraodon(ENSTNIP00000003297) TSSFLRPKAAACFLKEHNPQILHNETF------HLSSLDLSLGDEA------LEELLNTPAGRDAAPPLTRAALSFYIAA
Fugu(ENSTRUP00000012966) ASLFLHPEAAGHFLKEPDAQNLENEKY------HLSSLDLSVGDKAVEKYLKESVSKDAK------LLLTQEALSFYIAV
Medaka(ENSORLP00000018521) TSCFLQQSAAARYLEEHDARILRSKKSL------LSSPDLDLGGNE------VEDALRACDEPAALPHFCEQALSFYAAL
Amazon_molly(ENSPFOP0000000632 ASRFLQPNAAARYLNEHDAQILKNKKH------HLSGPNLNLGGKG------VEDFLKTEEAAEVLPQLQEELLQFYVAA
Platyfish(ENSXMAP00000011441) TSRFLQPNAAARYLNEHNAQILKNKKHH------LSGPDLNLGGKD------MEDFLKTEKAAEVLPQLQEELLLFYVAA
Stickleback(ENSGACP00000015822 TSYFLQPEAAARFTKERNPRILEGKKF------HWSASKLCLGGNA------VEDFLNESKATDTLAILKEEALSFYIAL
Tilapia(ENSONIP00000021143) TSYFLSPQAADRFLIERDVQILKNKNF------HLPTSELCLGKKT------VEDFLKTSEAAEALPLLQEDVLSFYIAL
Cod(ENSGMOP00000021057) AAHFLNPQAAERFLEEHDARVL------------TDKTLHRSGADL-------WLGSAVEELLD--EPLTQQFLSFYMTL
Rainbow trout(XP_021472161.1) ASSFLRPQAVVLFLKERDAFLLKTTKFH------LPGAELNVGGAV----VEDFLCESSETGSEALQLLQEQALSFYTAL
Atlantic salmon (XM_014146676. ASSFLRPQAVVDFLKERDTFLLKTTKFH------LAGAELNVGGAV----VEDFLCELSETGSEALKLLQEQALSFYTAL
Rainbow trout(XP_021422885.1) ASSFLRPQAVVRFLKERDVSLLKTTKFH------LPGPELNVGGAA----VEDFLCESSESGGEALQFLQMQALAFYTAL
Atlantic salmon(XM_014136242.1 ASSFLRPQAVVRFLKERDVSLLKNTKFH------LPGPELNVGGAA----VEDFLCESSESGSEALQFLQEQALSFYTAL
Zebrafish(ENSDARP00000046459) ASSFLRPQAVIRYLKEQDPAILDNEAFC------LPAAELSLGGVL-----EDFISAREEELADFLSTFYNECLAIYKTL
Golden line barbell(XM_0164740 ASSFLRPQAVIRYLKERDPAILENSTFC------LPATELSLGGVI-----EDFISAREEELADYLNVFHDECLAFYKTL
Dnmt3bbb
Tetraodon (ENSTNIP00000009617)
Fugu(ENSTRUP00000030962) AASFLHPSASSCFVRRWDLCILQDKENL------LLSCDVDIGASARDVLGSTALTEQDR------NDFFNAATTFYKAV
Platyfish(ENSXMAP00000009128) TTSICRPHAVDRFLLGGSSFLLQRELDLLHGDELLPVAEVNVGPRA----SDFMWAFGPSLGEQNLARFLKDAQAFYRAA
Stickleback(ENSGACP00000013567 ATNLLRPSVAEHFFTMRDVRVLQDGTK------LLPITEVQVGARA---REFLWATAVVDLGEQERRDFLKDAATFYKAA
Atlantic salmon(XP_014001845.1
Rainbow trout (XP_021422185.1) AASFLRSPAVDRFLRRREPTLLVNEKD------HLLRVDVNTGARV----KDFLSEHQAELDDDVVDSFLESSVSFYKAT
Atlantic salmon(XM_014135212.1 AASFLRSPAVDRLLRRWELTLLGNEDH-------LLRVEVNTGARV----KDFLSEHQAELKDDIVDSFLESSVSFYMAT
Zebrafish(ENSDARP00000074994) TQSFLRPKAAEYFHRRGKTSLVQETVGH------LPRGEVAVGEQA-----ADFLQQHSEELSDYLETFHSSIISFYTTV
Golden line barbell(XM_0164450 TTRFLRPKAAEFFLRRGKTSLIQDTVG------HLPRGEVEVGKQA-----ADFLLQRSEELTDCLETFHSSIISFYATV
Zebrafish(ENSDARP00000118597) TQSFLRPKAAEYFHRRGKTSLVQETVG------HLPRGEVAVGEQA-----ADFLQQHSEELSDYLETFHRSIISFYTTV
Golden line barbell(XM_0165118 TASFLRPKTAELFHRRGKTSLIQETVGH------LPRGEVEVGKQA-----ADFLLQCSEELTDYLETFHSSVISFYTTV
DNM3A
Lamprey(ENSPMAT00000008525) --
Lamprey(gi|1304463388|gb|PIZI0 --
Lamprey(ENSPMAT00000007628) --MEGPPL--------------------------------------
DNM3
Ciona_savignyi(ENSCSAVG0000000 --
Ciona_savignyi(ENSCSAVP0000002 --
European amphioxus(Sc0000092) --

```
                                                     730       740       750       760       770       780       790       800
                                                ....|....|....|....|....|....|....|....|....|....|....|....|....|....|....|....|
          Human(ENSP00000264709)                --------------------------------------------------------------------------------
          Pig(ENSSSCP00000019203)               ------------MNAMEENQGSTESQKVEE--------------------------------------------------
          Mouse(ENSMUSP00000020991)             --------------------------------------------------------------------------------
          Rat(ENSRNOP00000046524)               --------------------------------------------------------------------------------
DNM3A     Chicken(ENSGALP00000048074)           --------------------------------------------------------------------------------
          Lizard(ENSACAP00000007951)            --------------------------------------------------------------------------------
          Coelacanth(ENSLACP00000014690)        --------------------------------------------------------------------------------
          Elephant_shark(SINCAMP00000011        --------------------------------------------------------------------------------
          Spotted gar(ENSLOCP00000020139        --------------------------------------------------------------------------------
          Tetraodon(ENSTNIP00000022331)         --------------------------------------------------------------------------------
          Fugu(ENSTRUP00000009049)              --------------------------------------------------------------------------------
          Medaka(ENSORLP00000022087)            --------------------------------------------------------------------------------
          Tilapia(ENSONIP00000006964)           --------------------------------------------------------------------------------
          Platyfish(XP_023203761.1)             --------------------------------------------------------------------------------
Dnmt3aa   Amazon_molly(ENSPFOP0000001500        --------------------------------------------------------------------------------
          Stickleback(ENSGACP00000009407        --------------------------------------------------------------------------------
          Cod(ENSGMOP00000019846)               --------------------------------------------------------------------------------
          Rainbow trout(XP_021470020.1)         --------------------------------------------------------------------------------
          Atlantic salmon(XM_014143600.1        --------------------------------------------------------------------------------
          Zebrafish(ENSDARP00000091093)         --------------------------------------------------------------------------------
          Golden line barbell(XM_0164978        --------------------------------------------------------------------------------
          Tetraodon(ENSTNIP00000022492)         --------------------------------------------------------------------------------
          Fugu (ENSTRUP00000024460)             --------------------------------------------------------------------------------
          Stickleback(ENSGACP00000015064        --------------------------------------------------------------------------------
          Tilapia(ENSONIP00000001330)           --------------------------------------------------------------------------------
          Platyfish(ENSXMAP00000036171)         --------------------------------------------------------------------------------
          Amazon_molly(ENSPFOP0000001799        --------------------------------------------------------------------------------
Dnmt3ab   Cod(ENSGMOP00000020958)               --------------------------------------------------------------------------------
          Rainbow trout(XP_021429488.1)         --------------------------------------------------------------------------------
          Atlantic salmon(XM_014197582.1        --------------------------------------------------------------------------------
          Rainbow trout(XP_021440975.1)         --------------------------------------------------------------------------------
          Atlantic salmon(XM_014211429.1        --------------------------------------------------------------------------------
          Zebrafish(ENSDARP00000108609)         --------------------------------------------------------------------------------
          Golden line barbell(XM_0164754        --------------------------------------------------------------------------------
          Human(ENSP00000328547)                --------------------------------------------------------------------------------
          Pig(ENSSSCP00000007722)               ----------QGRHHVDESPVEFSATRSLR--------------------------------------------------
          Mouse(ENSMUSP00000051830)             --------------------------------------------------------------------------------
          Rat(ENSRNOP00000015482)               --------------------------------------------------------------------------------
DNMT3B    Mouse(ENSMUSG00000082079)             --------------------------------------------------------------------------------
          Chinese hamster(ENSCGRP0000001        --------------------------------------------------------------------------------  DNMT3C
          Rat(UCSC RGSC 5.0/rn5 chr3)           --------------------------------------------------------------------------------
          Chicken(ENSGALP00000052985)           --------------------------------------------------------------------------------
          Lizard(ENSACAP00000009682)            --------------------------------------------------------------------------------
          Elephant_shark(SINCAMP00000014        --------------------------------------------------------------------------------
          Coelacanth(ENSLACP00000009510)        --------------------------------------------------------------------------------
          Spotted gar(ENSLOCP00000006885        --------------------------------------------------------------------------------
          Tetraodon(ENSTNIP00000009615)         --------------------------------------------------------------------------------
          Fugu(ENSTRUP00000031152)              --------------------------------------------------------------------------------
          Stickleback(ENSGACP00000013579        --------------------------------------------------------------------------------
          Tilapia(ENSONIP00000018686)           --------------------------------------------------------------------------------
Dnmt3ba   Amazon_molly(ENSPFOP0000001143        --------------------------------------------------------------------------------
          Platyfish(ENSXMAP00000009160)         --------------------------------------------------------------------------------
          Medak(ENSORLP00000025108)             --------------------------------------------------------------------------------
          Cod(ENSGMOP00000016142)               --------------------------------------------------------------------------------
          Rainbow trout(XP_021471392.1)         --------------------------------------------------------------------------------
          Atlantic salmon(XM_014146292.1        --------------------------------------------------------------------------------
          Rainbow trout(XP_021422181.1)         --------------------------------------------------------------------------------
          Atlantic salmon(XM_014135215.1        --------------------------------------------------------------------------------
          Zebrafish(ENSDARP00000053417)         --------------------------------------------------------------------------------
          Golden line barbell(XM_0164651        --------------------------------------------------------------------------------
          Spotted gar(ENSLOCP00000006834        TAQIMQNLP--LNDTLLRNISILLNPSCKLK---ITGKVVADLATHLGLCKRPEETTQLIDEFLEYQLLENDEAQVLP--
          Tetraodon(ENSTNIP00000003297)         TRCVAETLP--LSERTRQSVAHLLNPRGWLH---LAGKAVGRLGGELGVCSSAEDAARLTQEFLQHRGEGEEVEG-----
          Fugu(ENSTRUP00000012966)              TRCITEKLP--LGEGVLKSIAQLLSPHRWLK---VAGKAVGELGTQLGICSSAEDATQLTDEFLQHRLLE----------
          Medaka(ENSORLP00000018521)            TGCIAEELP--LSVKALRSIAQLLSPQSWLK---VTSAQVKELGTQLGVCNSTDEIRQLTEEFLECKPTREVGPHAGTTV
          Amazon_molly(ENSPFOP0000000632        TCCIAEELP--LSDTVLRNVAQLLNPESSLK---VTDVAVEDLGRKLGVCSS-PEEVRQLKQEFSEYQQAEEERSDQGEN
          Platyfish(ENSXMAP00000011441)         TCCIAEELP--LSDTVLRNIAQLLNPESSLK---VTDVAMEELGRKLEVCSSPEEVRQLKQEFREYQQAEEERSNQGVNG
          Stickleback(ENSGACP00000015822        TGCIAKELP--LSDQVLSGIAQLLNPDSRGS---LSGKEVEKLGTSLKVCSS-PEEVEQLTREFLEYQQAERGDKEPSGV
          Tilapia(ENSONIP00000021143)           TGCIAEELP--LNDSVLKSIAQLLNPQSRLK---VTEETVGELGTKLGICNSPEEVKQLTNEFLEYQKAQEKEAE-----
          Cod(ENSGMOP00000021057)               TGLIAKEVP--LSDRILKSMAQLLNPETKLK---VTDTAVEELGKRLGVCSTEGEVNQLTKELLEYQQREEEEEEEDGEH  Dnmt3bba
          Rainbow trout(XP_021472161.1)         TARVAEGLP--LSDGVLRSMAQLLSPQGRLK---VTGKAVGELGAKLGLCSS-PEEISQLNKEFLEYQLAEEGEGDDGEN
          Atlantic salmon (XM_014146676.        TASVAEGLP--LSDGVLRSMAQLLSPQGRLK---VTGKAVGELGAKLGLCSS-PEEISQLNKEFLEYQLAEEGEGDEGEN
          Rainbow trout(XP_021422885.1)         TASVAEGLP--LSDGVLRSMAQLLSPQGRLT---VTGKVVGELGAKLGLCSS-PEEIRQLNKEFLEYQLAEEGEWEEGEN
Dnmt3bb   Atlantic salmon(XM_014136242.1        TASVAEGLP--LSDGVLRSMAQLLSPQGRLK---VTRKVVGELGAKLGLCSS-PEEISQLNKEFLEYQLAEEGEWEEGEN
          Zebrafish(ENSDARP00000046459)         TTSIAASLP--LSDSVLRAISQLLSPAGRLK---VTGKNIVDLAVRFGFCSKPEDSAKLNDEFLEYQLAEEENLSST---
          Golden line barbell(XM_0164740        TTSIADSLP--LSDSVLRGISQLLSPAGRLK---VTGKSIVDLAVQFGICSKPEDSAKLTDEFLEYQLVEDEEDTSST--
          Tetraodon (ENSTNIP00000009617)        --------------------------------------------------------------------------------
          Fugu(ENSTRUP00000030962)              LETLVQNIPKRLGNAALMTVTKVLKHPEDLCVINVLIKSGVQLGVCEKDSAESRQMASDYLSALQAIKEEQL--------
          Platyfish(ENSXMAP00000009128)         LQSLVECIPGPLGDVTLRNIGLMLKHPESITEKHFPWEVLSVMAVQLGLCAAGPPEVELERSCCDFRKLIQEQSS-----
          Stickleback(ENSGACP00000013567        LECLVESLPRHLGEVVLTNIYTVLKHSGSIRGNTLSRNVLYELGFQLGLFKCGLSEKHQLAYDYHKLIKT----------
          Atlantic salmon(XP_014001845.1        --------------------------------------------------------------------------------
          Rainbow trout (XP_021422185.1)        TWSLMKSLP--LPNVALKNIPILLRPAGRLE---VTSRVVAELGSLMGLCGV-PGDMALLTDEFLEYMISEGEKPTADSP  Dnmt3bbb
          Atlantic salmon(XM_014135212.1        TWSLMESLP--LPNVALKNIPILLRPAGRLE---VTGRVVAELGSLMGLCGVPGDMALLTDEFLEYMISEGEKPTADSPS
          Zebrafish(ENSDARP00000074994)         TVNIVKRLP--LPDSTLRNLSLVLSPGKKLE---VTGKMVQDLGVGFGVCIRPDNVSLLTDEFLEYQLIDGGDTGSVD--
          Golden line barbell(XM_0164450        TVNIIERLP--FSDSILRNLSLVLSPGKKLE---VTGKIVQDLGVCFGVCSG-PENVSLLTDEFLEYQFIDGGDTQLAD-
          Zebrafish(ENSDARP00000118597)         TVNIVKRLP--LPDSTLRNLSLVLSPGKKLE---VTGKMVQDLGIGFGVC---SENVSLLKDEFLEYQFIDESDAQPAD-
          Golden line barbell(XM_0165118        TVNIVECLP--FSDSTMRNLSLVLCPGKKLE---VTGKIVQDLGACFGVCSS-PENVSLLTDEFLEYQFIDGGDTHPAD-
          Lamprey(ENSPMAT00000008525)           --------------------------------------------------------------------------------
DNM3A     Lamprey(gi|1304463388|gb|PIZI0        --------------------------------------------------------------------------------
          Lamprey(ENSPMAT00000007628)           --------------------------------------------------------------------------------
          Ciona_savignyi(ENSCSAVG0000000        --------------------------------------------------------------------------------
DNM3      Ciona_savignyi(ENSCSAVP0000002        --------------------------------------------------------------------------------
          European amphioxus(Sc0000092)         --------------------------------------------------------------------------------
```

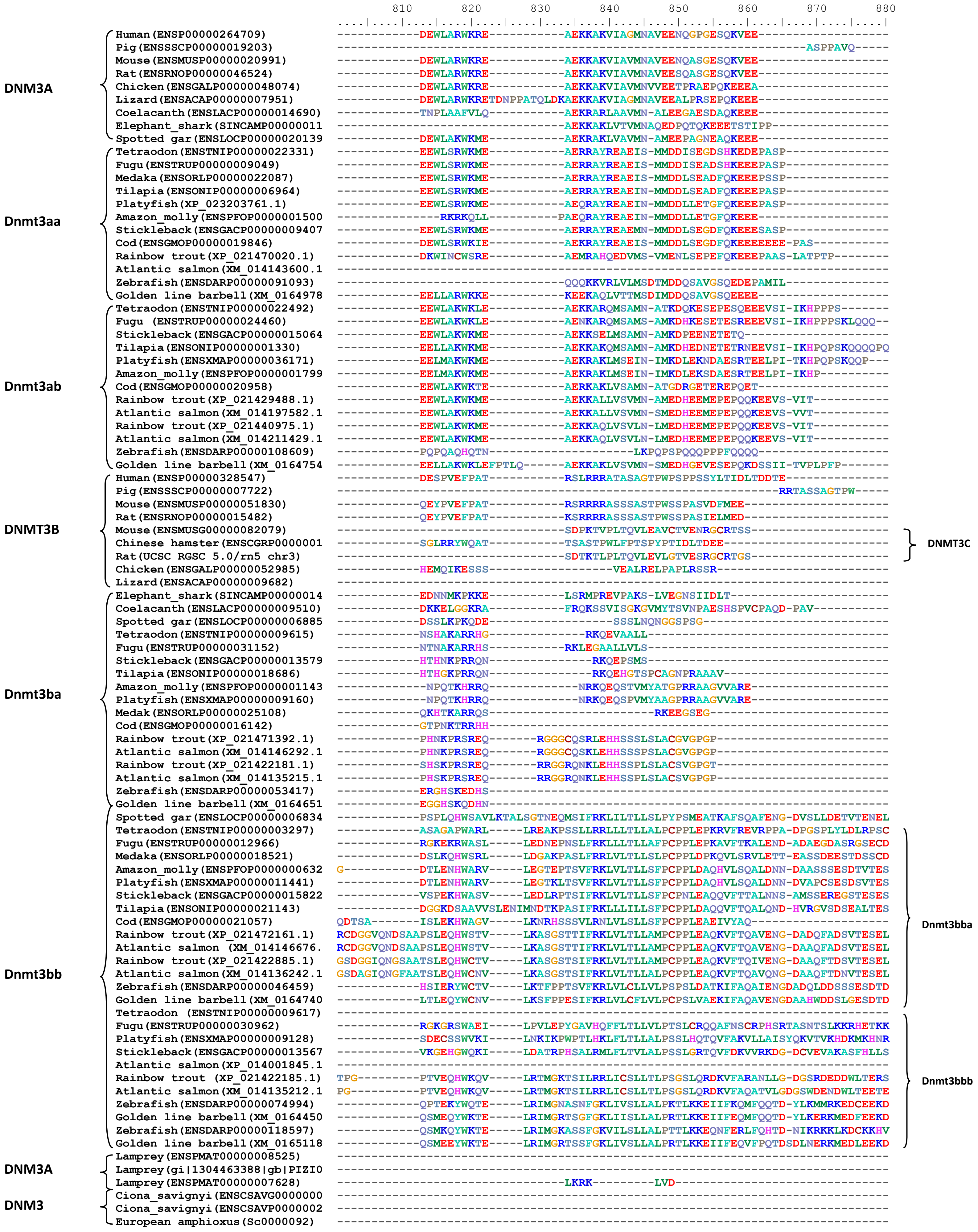

810
820
830
840
850
860
870
880
DNM3A
Dnmt3aa
Dnmt3ab
DNMT3B
DNMT3C
Dnmt3ba
Dnmt3bb
Dnmt3bba
Dnmt3bbb
DNM3A
DNM3

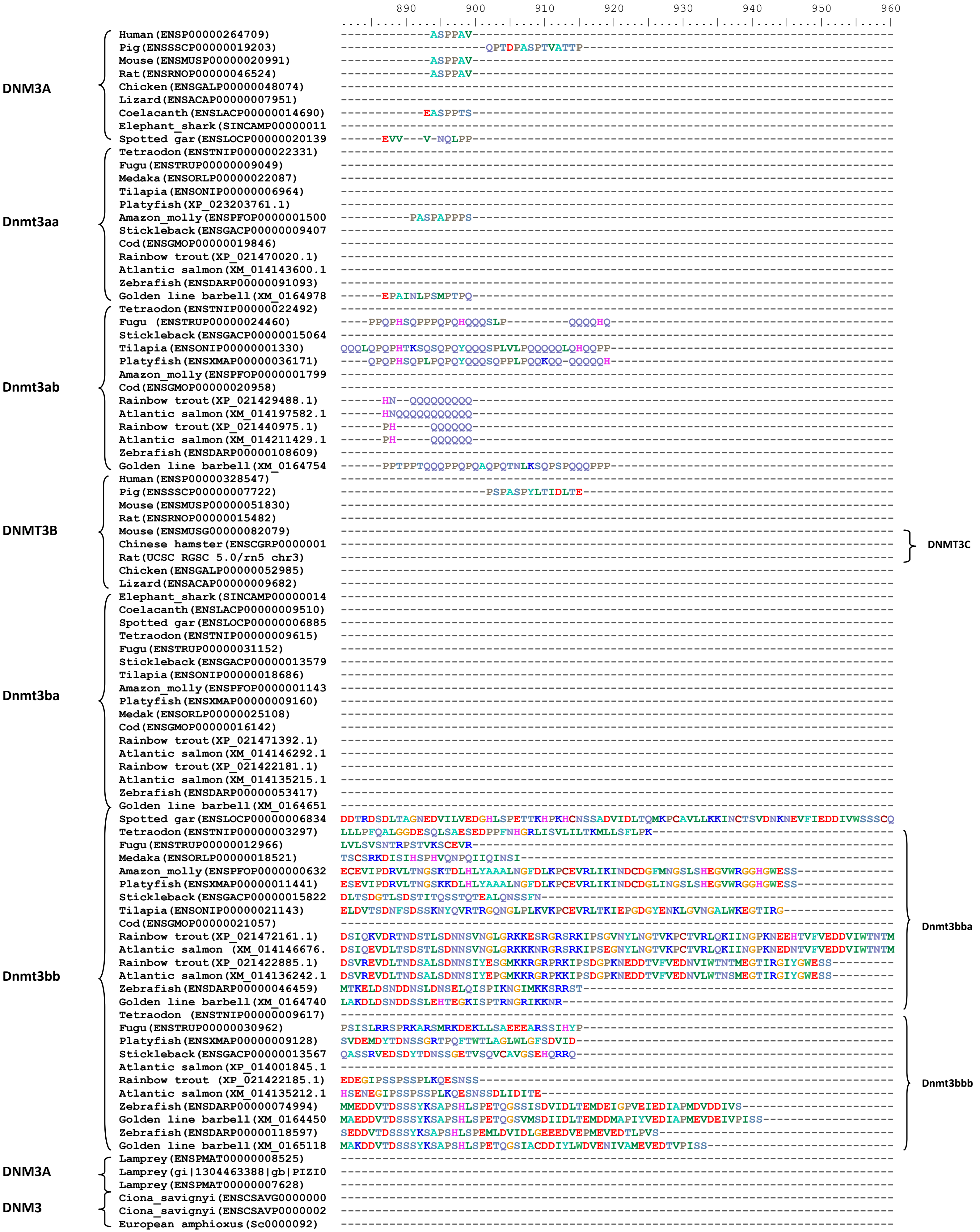

890
900
910
920
930
940
950
960
DNM3A
Human (ENSP00000264709)
Pig (ENSSSCP00000019203)
Mouse (ENSMUSP00000020991)
Rat (ENSRNOP00000046524)
Chicken (ENSGALP00000048074)
Lizard (ENSACAP00000007951)
Coelacanth (ENSLACP00000014690)
Elephant_shark (SINCAMP00000011
Spotted gar (ENSLOCP00000020139
Dnmt3aa
Tetraodon (ENSTNIP00000022331)
Fugu (ENSTRUP00000009049)
Medaka (ENSORLP00000022087)
Tilapia (ENSONIP00000006964)
Platyfish (XP_023203761.1)
Amazon_molly (ENSPFOP0000001500
Stickleback (ENSGACP00000009407
Cod (ENSGMOP00000019846)
Rainbow trout (XP_021470020.1)
Atlantic salmon (XM_014143600.1
Zebrafish (ENSDARP00000091093)
Golden line barbell (XM_0164978
Dnmt3ab
Tetraodon (ENSTNIP00000022492)
Fugu (ENSTRUP00000024460)
Stickleback (ENSGACP00000015064
Tilapia (ENSONIP00000001330)
Platyfish (ENSXMAP00000036171)
Amazon_molly (ENSPFOP0000001799
Cod (ENSGMOP00000020958)
Rainbow trout (XP_021429488.1)
Atlantic salmon (XM_014197582.1
Rainbow trout (XP_021440975.1)
Atlantic salmon (XM_014211429.1
Zebrafish (ENSDARP00000108609)
Golden line barbell (XM_0164754
DNMT3B
Human (ENSP00000328547)
Pig (ENSSSCP00000007722)
Mouse (ENSMUSP00000051830)
Rat (ENSRNOP00000015482)
Mouse (ENSMUSG00000082079)
Chinese hamster (ENSCGRP0000001
Rat (UCSC RGSC 5.0/rn5 chr3)
DNMT3C
Chicken (ENSGALP00000052985)
Lizard (ENSACAP00000009682)
Dnmt3ba
Elephant_shark (SINCAMP00000014
Coelacanth (ENSLACP00000009510)
Spotted gar (ENSLOCP00000006885
Tetraodon (ENSTNIP00000009615)
Fugu (ENSTRUP00000031152)
Stickleback (ENSGACP00000013579
Tilapia (ENSONIP00000018686)
Amazon_molly (ENSPFOP0000001143
Platyfish (ENSXMAP00000009160)
Medak (ENSORLP00000025108)
Cod (ENSGMOP00000016142)
Rainbow trout (XP_021471392.1)
Atlantic salmon (XM_014146292.1
Rainbow trout (XP_021422181.1)
Atlantic salmon (XM_014135215.1
Zebrafish (ENSDARP00000053417)
Golden line barbell (XM_0164651
Dnmt3bb
Spotted gar (ENSLOCP00000006834
Tetraodon (ENSTNIP00000003297)
Fugu (ENSTRUP00000012966)
Medaka (ENSORLP00000018521)
Amazon_molly (ENSPFOP0000000632
Platyfish (ENSXMAP00000011441)
Stickleback (ENSGACP00000015822
Tilapia (ENSONIP00000021143)
Cod (ENSGMOP00000021057)
Rainbow trout (XP_021472161.1)
Atlantic salmon (XM_014146676.
Rainbow trout (XP_021422885.1)
Atlantic salmon (XM_014136242.1
Zebrafish (ENSDARP00000046459)
Golden line barbell (XM_0164740
Dnmt3bba
Tetraodon (ENSTNIP00000009617)
Fugu (ENSTRUP00000030962)
Platyfish (ENSXMAP00000009128)
Stickleback (ENSGACP00000013567
Atlantic salmon (XP_014001845.1
Rainbow trout (XP_021422185.1)
Atlantic salmon (XM_014135212.1
Zebrafish (ENSDARP00000074994)
Golden line barbell (XM_0164450
Zebrafish (ENSDARP00000118597)
Golden line barbell (XM_0165118
Dnmt3bbb
DNM3A
Lamprey (ENSPMAT00000008525)
Lamprey (gi|1304463388|gb|PIZI0
Lamprey (ENSPMAT00000007628)
DNM3
Ciona_savignyi (ENSCSAVG0000000
Ciona_savignyi (ENSCSAVP0000002
European amphioxus (Sc0000092)

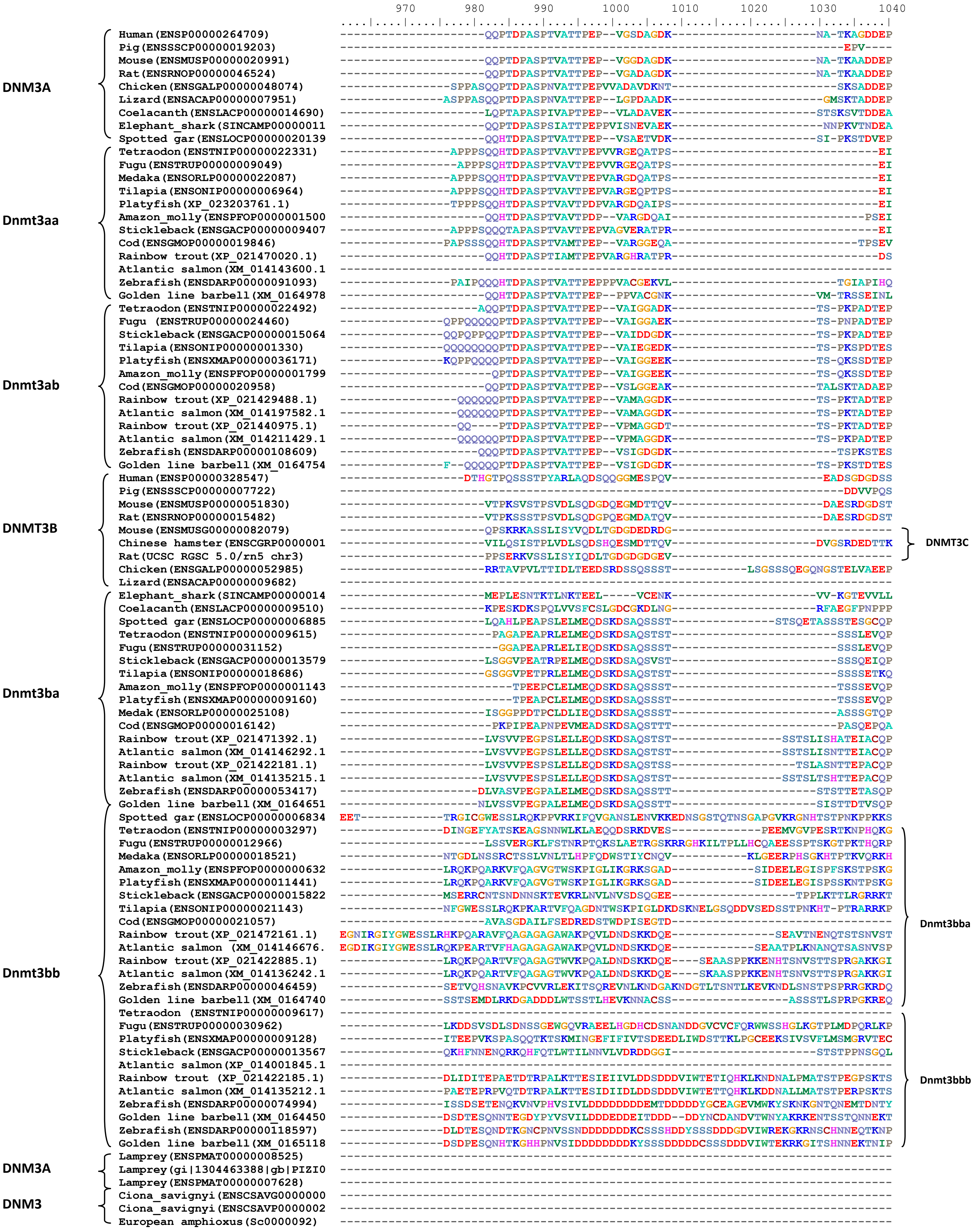

970 980 990 1000 1010 1020 1030 1040
DNM3A
Human(ENSP00000264709)
Pig(ENSSSCP00000019203)
Mouse(ENSMUSP00000020991)
Rat(ENSRNOP00000046524)
Chicken(ENSGALP00000048074)
Lizard(ENSACAP00000007951)
Coelacanth(ENSLACP00000014690)
Elephant_shark(SINCAMP00000011
Spotted gar(ENSLOCP00000020139
Dnmt3aa
Tetraodon(ENSTNIP00000022331)
Fugu(ENSTRUP00000009049)
Medaka(ENSORLP00000022087)
Tilapia(ENSONIP00000006964)
Platyfish(XP_023203761.1)
Amazon_molly(ENSPFOP0000001500
Stickleback(ENSGACP00000009407
Cod(ENSGMOP00000019846)
Rainbow trout(XP_021470020.1)
Atlantic salmon(XM_014143600.1
Zebrafish(ENSDARP00000091093)
Golden line barbell(XM_0164978
Dnmt3ab
Tetraodon(ENSTNIP00000022492)
Fugu (ENSTRUP00000024460)
Stickleback(ENSGACP00000015064
Tilapia(ENSONIP00000001330)
Platyfish(ENSXMAP00000036171)
Amazon_molly(ENSPFOP0000001799
Cod(ENSGMOP00000020958)
Rainbow trout(XP_021429488.1)
Atlantic salmon(XM_014197582.1
Rainbow trout(XP_021440975.1)
Atlantic salmon(XM_014211429.1
Zebrafish(ENSDARP00000108609)
Golden line barbell(XM_0164754
DNMT3B
Human(ENSP00000328547)
Pig(ENSSSCP00000007722)
Mouse(ENSMUSP00000051830)
Rat(ENSRNOP00000015482)
Mouse(ENSMUSG00000082079)
Chinese hamster(ENSCGRP0000001
Rat(UCSC RGSC 5.0/rn5 chr3)
Chicken(ENSGALP00000052985)
Lizard(ENSACAP00000009682)
DNMT3C
Dnmt3ba
Elephant_shark(SINCAMP00000014
Coelacanth(ENSLACP00000009510)
Spotted gar(ENSLOCP00000006885
Tetraodon(ENSTNIP00000009615)
Fugu(ENSTRUP00000031152)
Stickleback(ENSGACP00000013579
Tilapia(ENSONIP00000018686)
Amazon_molly(ENSPFOP0000001143
Platyfish(ENSXMAP00000009160)
Medak(ENSORLP00000025108)
Cod(ENSGMOP00000016142)
Rainbow trout(XP_021471392.1)
Atlantic salmon(XM_014146292.1
Rainbow trout(XP_021422181.1)
Atlantic salmon(XM_014135215.1
Zebrafish(ENSDARP00000053417)
Golden line barbell(XM_0164651
Dnmt3bb
Spotted gar(ENSLOCP00000006834
Tetraodon(ENSTNIP00000003297)
Fugu(ENSTRUP00000012966)
Medaka(ENSORLP00000018521)
Amazon_molly(ENSPFOP0000000632
Platyfish(ENSXMAP00000011441)
Stickleback(ENSGACP00000015822
Tilapia(ENSONIP00000021143)
Cod(ENSGMOP00000021057)
Rainbow trout(XP_021472161.1)
Atlantic salmon (XM_014146676.
Rainbow trout(XP_021422885.1)
Atlantic salmon(XM_014136242.1
Zebrafish(ENSDARP00000046459)
Golden line barbell(XM_0164740
Dnmt3bba
Tetraodon (ENSTNIP00000009617)
Fugu(ENSTRUP00000030962)
Platyfish(ENSXMAP00000009128)
Stickleback(ENSGACP00000013567
Atlantic salmon(XP_014001845.1
Rainbow trout (XP_021422185.1)
Atlantic salmon(XM_014135212.1
Zebrafish(ENSDARP00000074994)
Golden line barbell(XM_0164450
Zebrafish(ENSDARP00000118597)
Golden line barbell(XM_0165118
Dnmt3bbb
DNM3A
Lamprey(ENSPMAT00000008525)
Lamprey(gi|1304463388|gb|PIZI0
Lamprey(ENSPMAT00000007628)
DNM3
Ciona_savignyi(ENSCSAVG0000000
Ciona_savignyi(ENSCSAVP0000002
European amphioxus(Sc0000092)

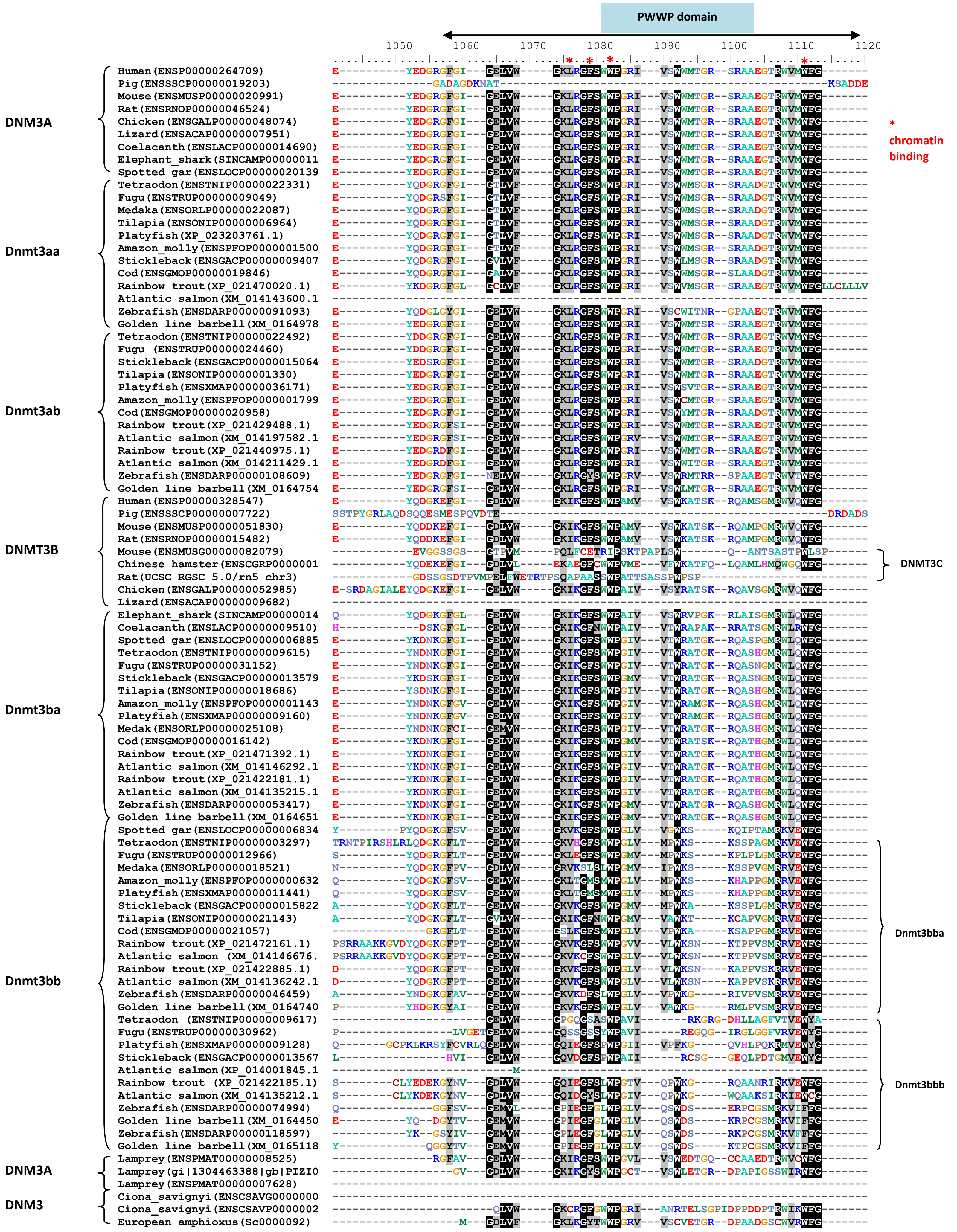

PWWP domain
1050
1060
1070
1080
1090
1100
1110
1120
* chromatin binding
DNM3A
Human (ENSP00000264709)
Pig (ENSSSCP00000019203)
Mouse (ENSMUSP00000020991)
Rat (ENSRNOP00000046524)
Chicken (ENSGALP00000048074)
Lizard (ENSACAP00000007951)
Coelacanth (ENSLACP00000014690)
Elephant_shark (SINCAMP00000011
Spotted gar (ENSLOCP00000020139
Dnmt3aa
Tetraodon (ENSTNIP00000022331)
Fugu (ENSTRUP00000009049)
Medaka (ENSORLP00000022087)
Tilapia (ENSONIP00000006964)
Platyfish (XP_023203761.1)
Amazon_molly (ENSPFOP0000001500
Stickleback (ENSGACP00000009407
Cod (ENSGMOP00000019846)
Rainbow trout (XP_021470020.1)
Atlantic salmon (XM_014143600.1
Zebrafish (ENSDARP00000091093)
Golden line barbell (XM_0164978
Dnmt3ab
Tetraodon (ENSTNIP00000022492)
Fugu (ENSTRUP00000024460)
Stickleback (ENSGACP00000015064
Tilapia (ENSONIP00000001330)
Platyfish (ENSXMAP00000036171)
Amazon_molly (ENSPFOP0000001799
Cod (ENSGMOP00000020958)
Rainbow trout (XP_021429488.1)
Atlantic salmon (XM_014197582.1
Rainbow trout (XP_021440975.1)
Atlantic salmon (XM_014211429.1
Zebrafish (ENSDARP00000108609)
Golden line barbell (XM_0164754
DNMT3B
Human (ENSP00000328547)
Pig (ENSSSCP00000007722)
Mouse (ENSMUSP00000051830)
Rat (ENSRNOP00000015482)
Mouse (ENSMUSG00000082079)
Chinese hamster (ENSCGRP0000001
Rat (UCSC RGSC 5.0/rn5 chr3)
Chicken (ENSGALP00000052985)
Lizard (ENSACAP00000009682)
DNMT3C
Dnmt3ba
Elephant_shark (SINCAMP00000014
Coelacanth (ENSLACP00000009510)
Spotted gar (ENSLOCP00000006885
Tetraodon (ENSTNIP00000009615)
Fugu (ENSTRUP00000031152)
Stickleback (ENSGACP00000013579
Tilapia (ENSONIP00000018686)
Amazon_molly (ENSPFOP0000001143
Platyfish (ENSXMAP00000009160)
Medak (ENSORLP00000025108)
Cod (ENSGMOP00000016142)
Rainbow trout (XP_021471392.1)
Atlantic salmon (XM_014146292.1
Rainbow trout (XP_021422181.1)
Atlantic salmon (XM_014135215.1
Zebrafish (ENSDARP00000053417)
Golden line barbell (XM_0164651
Dnmt3bb
Spotted gar (ENSLOCP00000006834
Tetraodon (ENSTNIP00000003297)
Fugu (ENSTRUP00000012966)
Medaka (ENSORLP00000018521)
Amazon_molly (ENSPFOP0000000632
Platyfish (ENSXMAP00000011441)
Stickleback (ENSGACP00000015822
Tilapia (ENSONIP00000021143)
Cod (ENSGMOP00000021057)
Rainbow trout (XP_021472161.1)
Atlantic salmon (XM_014146676.
Rainbow trout (XP_021422885.1)
Atlantic salmon (XM_014136242.1
Zebrafish (ENSDARP00000046459)
Golden line barbell (XM_0164740
Dnmt3bba
Tetraodon (ENSTNIP00000009617)
Fugu (ENSTRUP00000030962)
Platyfish (ENSXMAP00000009128)
Stickleback (ENSGACP00000013567
Atlantic salmon (XP_014001845.1
Rainbow trout (XP_021422185.1)
Atlantic salmon (XM_014135212.1
Zebrafish (ENSDARP00000074994)
Golden line barbell (XM_0164450
Zebrafish (ENSDARP00000118597)
Golden line barbell (XM_0165118
Dnmt3bbb
DNM3A
Lamprey (ENSPMAT00000008525)
Lamprey (gi|1304463388|gb|PIZI0
Lamprey (ENSPMAT00000007628)
DNM3
Ciona_savignyi (ENSCSAVG0000000
Ciona_savignyi (ENSCSAVP0000002
European amphioxus (Sc0000092)

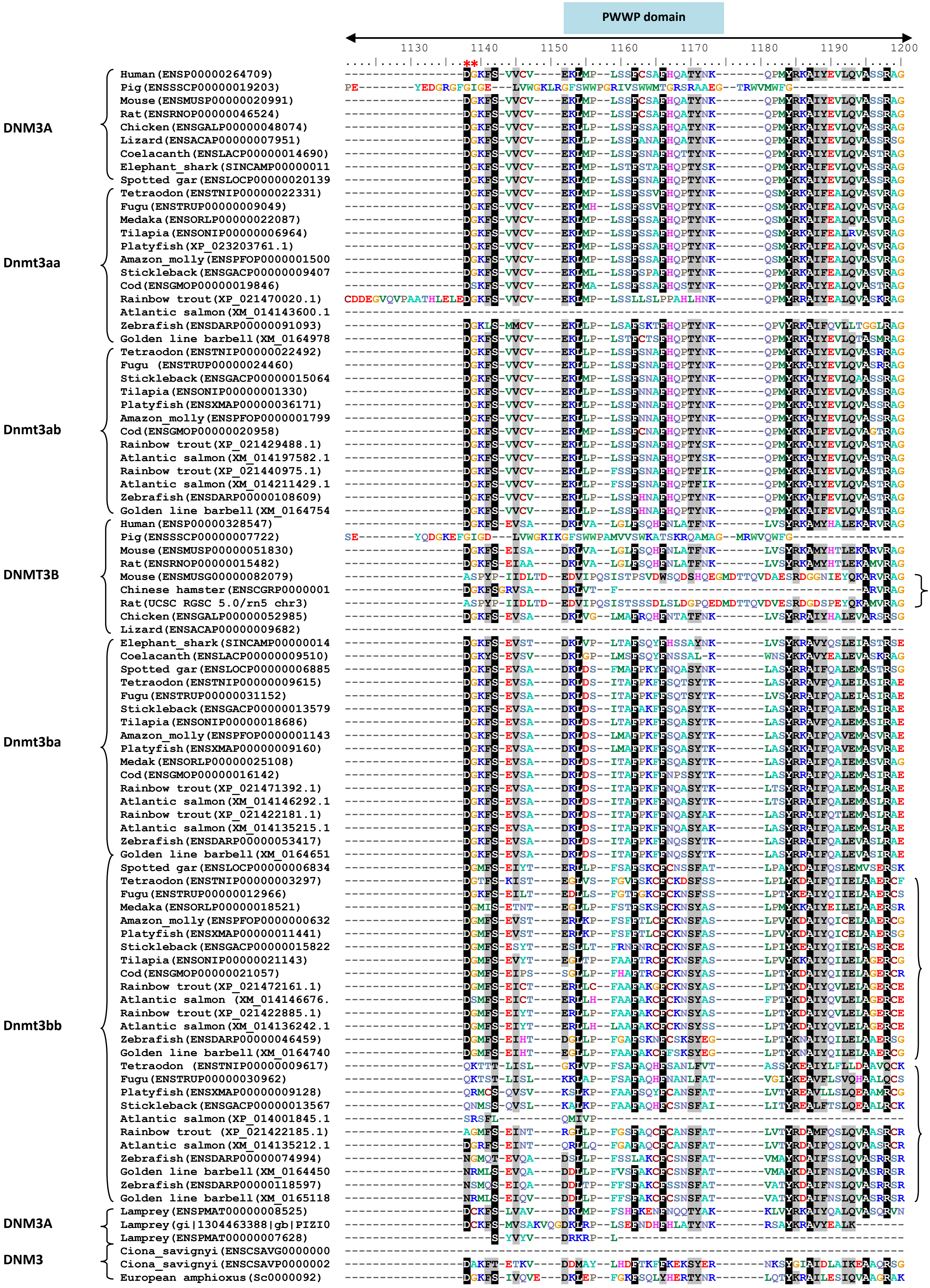

PWWP domain
1130
1140
1150
1160
1170
1180
1190
1200
DNM3A
Human (ENSP00000264709)
Pig (ENSSSCP00000019203)
Mouse (ENSMUSP00000020991)
Rat (ENSRNOP00000046524)
Chicken (ENSGALP00000048074)
Lizard (ENSACAP00000007951)
Coelacanth (ENSLACP00000014690)
Elephant_shark (SINCAMP00000011
Spotted gar (ENSLOCP00000020139
Dnmt3aa
Tetraodon (ENSTNIP00000022331)
Fugu (ENSTRUP00000009049)
Medaka (ENSORLP00000022087)
Tilapia (ENSONIP00000006964)
Platyfish (XP_023203761.1)
Amazon_molly (ENSPFOP0000001500
Stickleback (ENSGACP00000009407
Cod (ENSGMOP00000019846)
Rainbow trout (XP_021470020.1)
Atlantic salmon (XM_014143600.1
Zebrafish (ENSDARP00000091093)
Golden line barbell (XM_0164978
Dnmt3ab
Tetraodon (ENSTNIP00000022492)
Fugu (ENSTRUP00000024460)
Stickleback (ENSGACP00000015064
Tilapia (ENSONIP00000001330)
Platyfish (ENSXMAP00000036171)
Amazon_molly (ENSPFOP0000001799
Cod (ENSGMOP00000020958)
Rainbow trout (XP_021429488.1)
Atlantic salmon (XM_014197582.1
Rainbow trout (XP_021440975.1)
Atlantic salmon (XM_014211429.1
Zebrafish (ENSDARP00000108609)
Golden line barbell (XM_0164754
DNMT3B
Human (ENSP00000328547)
Pig (ENSSSCP00000007722)
Mouse (ENSMUSP00000051830)
Rat (ENSRNOP00000015482)
Mouse (ENSMUSG00000082079)
Chinese hamster (ENSCGRP0000001
Rat (UCSC RGSC 5.0/rn5 chr3)
Chicken (ENSGALP00000052985)
Lizard (ENSACAP00000009682)
DNMT3C
Dnmt3ba
Elephant_shark (SINCAMP00000014
Coelacanth (ENSLACP00000009510)
Spotted gar (ENSLOCP00000006885
Tetraodon (ENSTNIP00000009615)
Fugu (ENSTRUP00000031152)
Stickleback (ENSGACP00000013579
Tilapia (ENSONIP00000018686)
Amazon_molly (ENSPFOP0000001143
Platyfish (ENSXMAP00000009160)
Medak (ENSORLP00000025108)
Cod (ENSGMOP00000016142)
Rainbow trout (XP_021471392.1)
Atlantic salmon (XM_014146292.1
Rainbow trout (XP_021422181.1)
Atlantic salmon (XM_014135215.1
Zebrafish (ENSDARP00000053417)
Golden line barbell (XM_0164651
Dnmt3bb
Spotted gar (ENSLOCP00000006834
Tetraodon (ENSTNIP00000003297)
Fugu (ENSTRUP00000012966)
Medaka (ENSORLP00000018521)
Amazon_molly (ENSPFOP0000000632
Platyfish (ENSXMAP00000011441)
Stickleback (ENSGACP00000015822
Tilapia (ENSONIP00000021143)
Cod (ENSGMOP00000021057)
Rainbow trout (XP_021472161.1)
Atlantic salmon (XM_014146676.
Rainbow trout (XP_021422885.1)
Atlantic salmon (XM_014136242.1
Zebrafish (ENSDARP00000046459)
Golden line barbell (XM_0164740
Dnmt3bba
Tetraodon (ENSTNIP00000009617)
Fugu (ENSTRUP00000030962)
Platyfish (ENSXMAP00000009128)
Stickleback (ENSGACP00000013567
Atlantic salmon (XP_014001845.1
Rainbow trout (XP_021422185.1)
Atlantic salmon (XM_014135212.1
Zebrafish (ENSDARP00000074994)
Golden line barbell (XM_0164450
Zebrafish (ENSDARP00000118597)
Golden line barbell (XM_0165118
Dnmt3bbb
DNM3A
Lamprey (ENSPMAT00000008525)
Lamprey (gi|1304463388|gb|PIZI0
Lamprey (ENSPMAT00000007628)
DNM3
Ciona_savignyi (ENSCSAVG0000000
Ciona_savignyi (ENSCSAVP0000002
European amphioxus (Sc0000092)

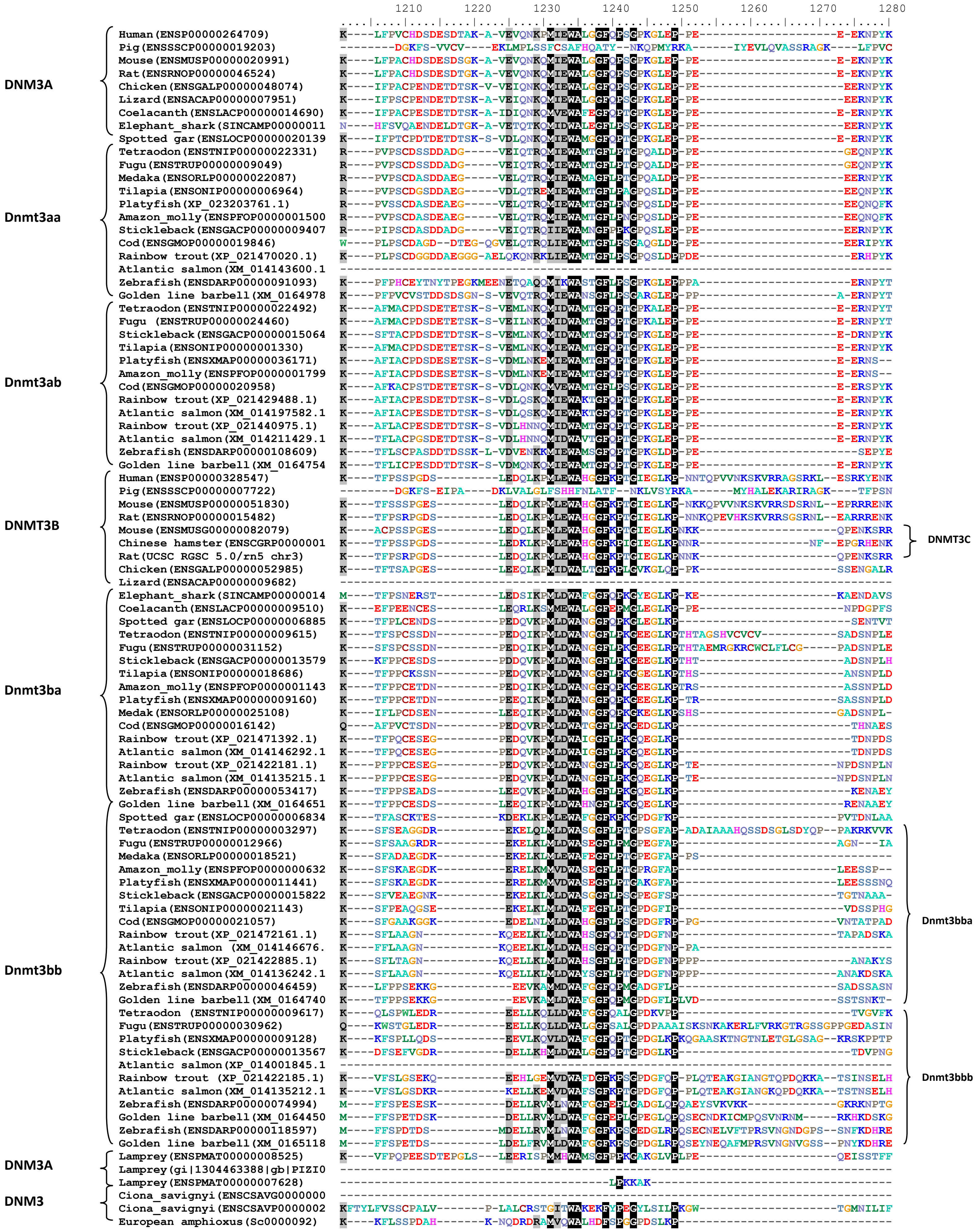

1210 1220 1230 1240 1250 1260 1270 1280
DNM3A
Human(ENSP00000264709)
Pig(ENSSSCP00000019203)
Mouse(ENSMUSP00000020991)
Rat(ENSRNOP00000046524)
Chicken(ENSGALP00000048074)
Lizard(ENSACAP00000007951)
Coelacanth(ENSLACP00000014690)
Elephant_shark(SINCAMP00000011
Spotted gar(ENSLOCP00000020139
Dnmt3aa
Tetraodon(ENSTNIP00000022331)
Fugu(ENSTRUP00000009049)
Medaka(ENSORLP00000022087)
Tilapia(ENSONIP00000006964)
Platyfish(XP_023203761.1)
Amazon_molly(ENSPFOP0000001500
Stickleback(ENSGACP00000009407
Cod(ENSGMOP00000019846)
Rainbow trout(XP_021470020.1)
Atlantic salmon(XM_014143600.1
Zebrafish(ENSDARP00000091093)
Golden line barbell(XM_0164978
Dnmt3ab
Tetraodon(ENSTNIP00000022492)
Fugu (ENSTRUP00000024460)
Stickleback(ENSGACP00000015064
Tilapia(ENSONIP00000001330)
Platyfish(ENSXMAP00000036171)
Amazon_molly(ENSPFOP0000001799
Cod(ENSGMOP00000020958)
Rainbow trout(XP_021429488.1)
Atlantic salmon(XM_014197582.1
Rainbow trout(XP_021440975.1)
Atlantic salmon(XM_014211429.1
Zebrafish(ENSDARP00000108609)
Golden line barbell(XM_0164754
DNMT3B
Human(ENSP00000328547)
Pig(ENSSSCP00000007722)
Mouse(ENSMUSP00000051830)
Rat(ENSRNOP00000015482)
Mouse(ENSMUSG00000082079)
Chinese hamster(ENSCGRP0000001
Rat(UCSC RGSC 5.0/rn5 chr3)
Chicken(ENSGALP00000052985)
Lizard(ENSACAP00000009682)
DNMT3C
Dnmt3ba
Elephant_shark(SINCAMP00000014
Coelacanth(ENSLACP00000009510)
Spotted gar(ENSLOCP00000006885
Tetraodon(ENSTNIP00000009615)
Fugu(ENSTRUP00000031152)
Stickleback(ENSGACP00000013579
Tilapia(ENSONIP00000018686)
Amazon_molly(ENSPFOP0000001143
Platyfish(ENSXMAP00000009160)
Medak(ENSORLP00000025108)
Cod(ENSGMOP00000016142)
Rainbow trout(XP_021471392.1)
Atlantic salmon(XM_014146292.1
Rainbow trout(XP_021422181.1)
Atlantic salmon(XM_014135215.1
Zebrafish(ENSDARP00000053417)
Golden line barbell(XM_0164651
Dnmt3bb
Spotted gar(ENSLOCP00000006834
Tetraodon(ENSTNIP00000003297)
Fugu(ENSTRUP00000012966)
Medaka(ENSORLP00000018521)
Amazon_molly(ENSPFOP0000000632
Platyfish(ENSXMAP00000011441)
Stickleback(ENSGACP00000015822
Tilapia(ENSONIP00000021143)
Cod(ENSGMOP00000021057)
Rainbow trout(XP_021472161.1)
Atlantic salmon (XM_014146676.
Rainbow trout(XP_021422885.1)
Atlantic salmon(XM_014136242.1
Zebrafish(ENSDARP00000046459)
Golden line barbell(XM_0164740
Dnmt3bba
Tetraodon (ENSTNIP00000009617)
Fugu(ENSTRUP00000030962)
Platyfish(ENSXMAP00000009128)
Stickleback(ENSGACP00000013567
Atlantic salmon(XP_014001845.1
Rainbow trout (XP_021422185.1)
Atlantic salmon(XM_014135212.1
Zebrafish(ENSDARP00000074994)
Golden line barbell(XM_0164450
Zebrafish(ENSDARP00000118597)
Golden line barbell(XM_0165118
Dnmt3bbb
DNM3A
Lamprey(ENSPMAT00000008525)
Lamprey(gi|1304463388|gb|PIZI0
Lamprey(ENSPMAT00000007628)
DNM3
Ciona_savignyi(ENSCSAVG0000000
Ciona_savignyi(ENSCSAVP0000002
European amphioxus(Sc0000092)

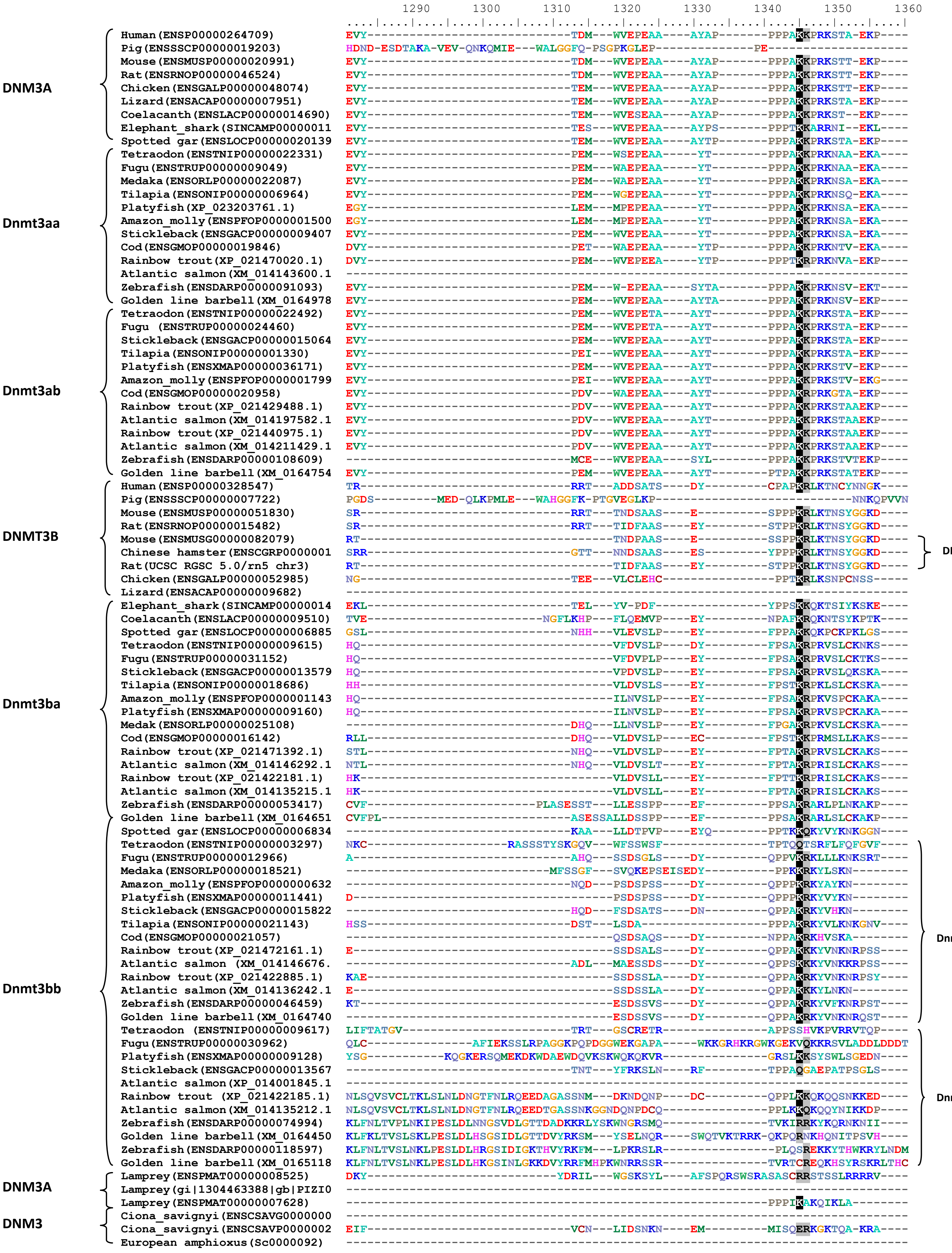

1290
1300
1310
1320
1330
1340
1350
1360
DNM3A
Human(ENSP00000264709)
Pig(ENSSSCP00000019203)
Mouse(ENSMUSP00000020991)
Rat(ENSRNOP00000046524)
Chicken(ENSGALP00000048074)
Lizard(ENSACAP00000007951)
Coelacanth(ENSLACP00000014690)
Elephant_shark(SINCAMP00000011
Spotted gar(ENSLOCP00000020139
Dnmt3aa
Tetraodon(ENSTNIP00000022331)
Fugu(ENSTRUP00000009049)
Medaka(ENSORLP00000022087)
Tilapia(ENSONIP00000006964)
Platyfish(XP_023203761.1)
Amazon_molly(ENSPFOP0000001500
Stickleback(ENSGACP00000009407
Cod(ENSGMOP00000019846)
Rainbow trout(XP_021470020.1)
Atlantic salmon(XM_014143600.1
Zebrafish(ENSDARP00000091093)
Golden line barbell(XM_0164978
Dnmt3ab
Tetraodon(ENSTNIP00000022492)
Fugu (ENSTRUP00000024460)
Stickleback(ENSGACP00000015064
Tilapia(ENSONIP00000001330)
Platyfish(ENSXMAP00000036171)
Amazon_molly(ENSPFOP0000001799
Cod(ENSGMOP00000020958)
Rainbow trout(XP_021429488.1)
Atlantic salmon(XM_014197582.1
Rainbow trout(XP_021440975.1)
Atlantic salmon(XM_014211429.1
Zebrafish(ENSDARP00000108609)
Golden line barbell(XM_0164754
DNMT3B
Human(ENSP00000328547)
Pig(ENSSSCP00000007722)
Mouse(ENSMUSP00000051830)
Rat(ENSRNOP00000015482)
Mouse(ENSMUSG00000082079)
Chinese hamster(ENSCGRP0000001
Rat(UCSC RGSC 5.0/rn5 chr3)
Chicken(ENSGALP00000052985)
Lizard(ENSACAP00000009682)
DNMT3C
Dnmt3ba
Elephant_shark(SINCAMP00000014
Coelacanth(ENSLACP00000009510)
Spotted gar(ENSLOCP00000006885
Tetraodon(ENSTNIP00000009615)
Fugu(ENSTRUP00000031152)
Stickleback(ENSGACP00000013579
Tilapia(ENSONIP00000018686)
Amazon_molly(ENSPFOP0000001143
Platyfish(ENSXMAP00000009160)
Medak(ENSORLP00000025108)
Cod(ENSGMOP00000016142)
Rainbow trout(XP_021471392.1)
Atlantic salmon(XM_014146292.1
Rainbow trout(XP_021422181.1)
Atlantic salmon(XM_014135215.1
Zebrafish(ENSDARP00000053417)
Golden line barbell(XM_0164651
Dnmt3bb
Spotted gar(ENSLOCP00000006834
Tetraodon(ENSTNIP00000003297)
Fugu(ENSTRUP00000012966)
Medaka(ENSORLP00000018521)
Amazon_molly(ENSPFOP0000000632
Platyfish(ENSXMAP00000011441)
Stickleback(ENSGACP00000015822
Tilapia(ENSONIP00000021143)
Cod(ENSGMOP00000021057)
Rainbow trout(XP_021472161.1)
Atlantic salmon (XM_014146676.
Rainbow trout(XP_021422885.1)
Atlantic salmon(XM_014136242.1
Zebrafish(ENSDARP00000046459)
Golden line barbell(XM_0164740
Dnmt3bba
Tetraodon (ENSTNIP00000009617)
Fugu(ENSTRUP00000030962)
Platyfish(ENSXMAP00000009128)
Stickleback(ENSGACP00000013567
Atlantic salmon(XP_014001845.1
Rainbow trout (XP_021422185.1)
Atlantic salmon(XM_014135212.1
Zebrafish(ENSDARP00000074994)
Golden line barbell(XM_0164450
Zebrafish(ENSDARP00000118597)
Golden line barbell(XM_0165118
Dnmt3bbb
DNM3A
Lamprey(ENSPMAT00000008525)
Lamprey(gi|1304463388|gb|PIZI0
Lamprey(ENSPMAT00000007628)
DNM3
Ciona_savignyi(ENSCSAVG0000000
Ciona_savignyi(ENSCSAVP0000002
European amphioxus(Sc0000092)

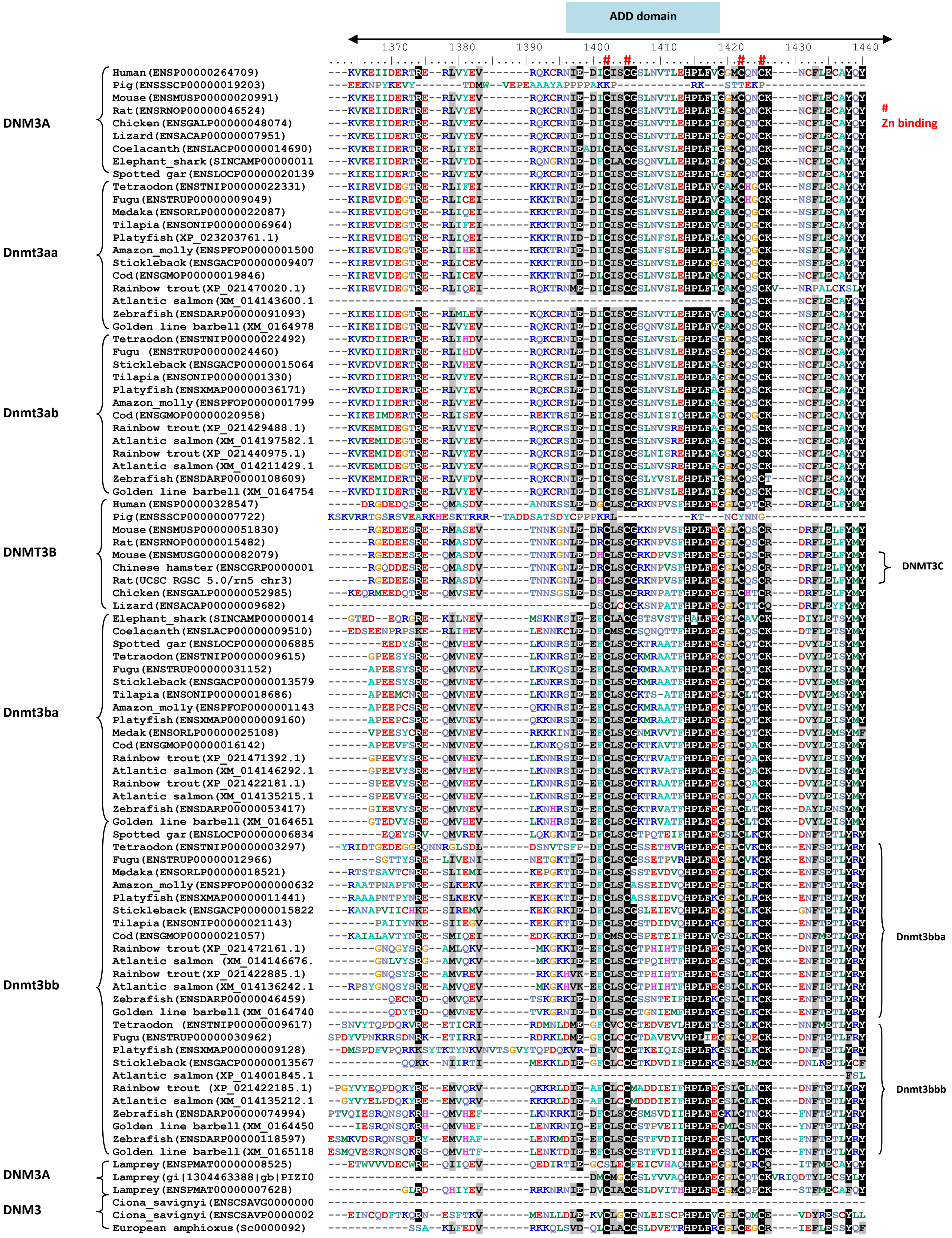

ADD domain
1370
1380
1390
1400
1410
1420
1430
1440
Zn binding
DNM3A
Human (ENSP00000264709)
Pig (ENSSSCP00000019203)
Mouse (ENSMUSP00000020991)
Rat (ENSRNOP00000046524)
Chicken (ENSGALP00000048074)
Lizard (ENSACAP00000007951)
Coelacanth (ENSLACP00000014690)
Elephant_shark (SINCAMP00000011
Spotted gar (ENSLOCP00000020139
Dnmt3aa
Tetraodon (ENSTNIP00000022331)
Fugu (ENSTRUP00000009049)
Medaka (ENSORLP00000022087)
Tilapia (ENSONIP00000006964)
Platyfish (XP_023203761.1)
Amazon_molly (ENSPFOP0000001500
Stickleback (ENSGACP00000009407
Cod (ENSGMOP00000019846)
Rainbow trout (XP_021470020.1)
Atlantic salmon (XM_014143600.1
Zebrafish (ENSDARP00000091093)
Golden line barbell (XM_0164978
Dnmt3ab
Tetraodon (ENSTNIP00000022492)
Fugu (ENSTRUP00000024460)
Stickleback (ENSGACP00000015064
Tilapia (ENSONIP00000001330)
Platyfish (ENSXMAP00000036171)
Amazon_molly (ENSPFOP0000001799
Cod (ENSGMOP00000020958)
Rainbow trout (XP_021429488.1)
Atlantic salmon (XM_014197582.1
Rainbow trout (XP_021440975.1)
Atlantic salmon (XM_014211429.1
Zebrafish (ENSDARP00000108609)
Golden line barbell (XM_0164754
DNMT3B
Human (ENSP00000328547)
Pig (ENSSSCP00000007722)
Mouse (ENSMUSP00000051830)
Rat (ENSRNOP00000015482)
Mouse (ENSMUSG00000082079)
Chinese hamster (ENSCGRP0000001
Rat (UCSC RGSC 5.0/rn5 chr3)
Chicken (ENSGALP00000052985)
Lizard (ENSACAP00000009682)
DNMT3C
Dnmt3ba
Elephant_shark (SINCAMP00000014
Coelacanth (ENSLACP00000009510)
Spotted gar (ENSLOCP00000006885
Tetraodon (ENSTNIP00000009615)
Fugu (ENSTRUP00000031152)
Stickleback (ENSGACP00000013579
Tilapia (ENSONIP00000018686)
Amazon_molly (ENSPFOP0000001143
Platyfish (ENSXMAP00000009160)
Medak (ENSORLP00000025108)
Cod (ENSGMOP00000016142)
Rainbow trout (XP_021471392.1)
Atlantic salmon (XM_014146292.1
Rainbow trout (XP_021422181.1)
Atlantic salmon (XM_014135215.1
Zebrafish (ENSDARP00000053417)
Golden line barbell (XM_0164651
Dnmt3bb
Spotted gar (ENSLOCP00000006834
Dnmt3bba
Tetraodon (ENSTNIP00000003297)
Fugu (ENSTRUP00000012966)
Medaka (ENSORLP00000018521)
Amazon_molly (ENSPFOP0000000632
Platyfish (ENSXMAP00000011441)
Stickleback (ENSGACP00000015822
Tilapia (ENSONIP00000021143)
Cod (ENSGMOP00000021057)
Rainbow trout (XP_021472161.1)
Atlantic salmon (XM_014146676.
Rainbow trout (XP_021422885.1)
Atlantic salmon (XM_014136242.1
Zebrafish (ENSDARP00000046459)
Golden line barbell (XM_0164740
Dnmt3bbb
Tetraodon (ENSTNIP00000009617)
Fugu (ENSTRUP00000030962)
Platyfish (ENSXMAP00000009128)
Stickleback (ENSGACP00000013567
Atlantic salmon (XP_014001845.1
Rainbow trout (XP_021422185.1)
Atlantic salmon (XM_014135212.1
Zebrafish (ENSDARP00000074994)
Golden line barbell (XM_0164450
Zebrafish (ENSDARP00000118597)
Golden line barbell (XM_0165118
DNM3A
Lamprey (ENSPMAT00000008525)
Lamprey (gi|1304463388|gb|PIZI0
Lamprey (ENSPMAT00000007628)
DNM3
Ciona_savignyi (ENSCSAVG0000000
Ciona_savignyi (ENSCSAVP0000002
European amphioxus (Sc0000092)

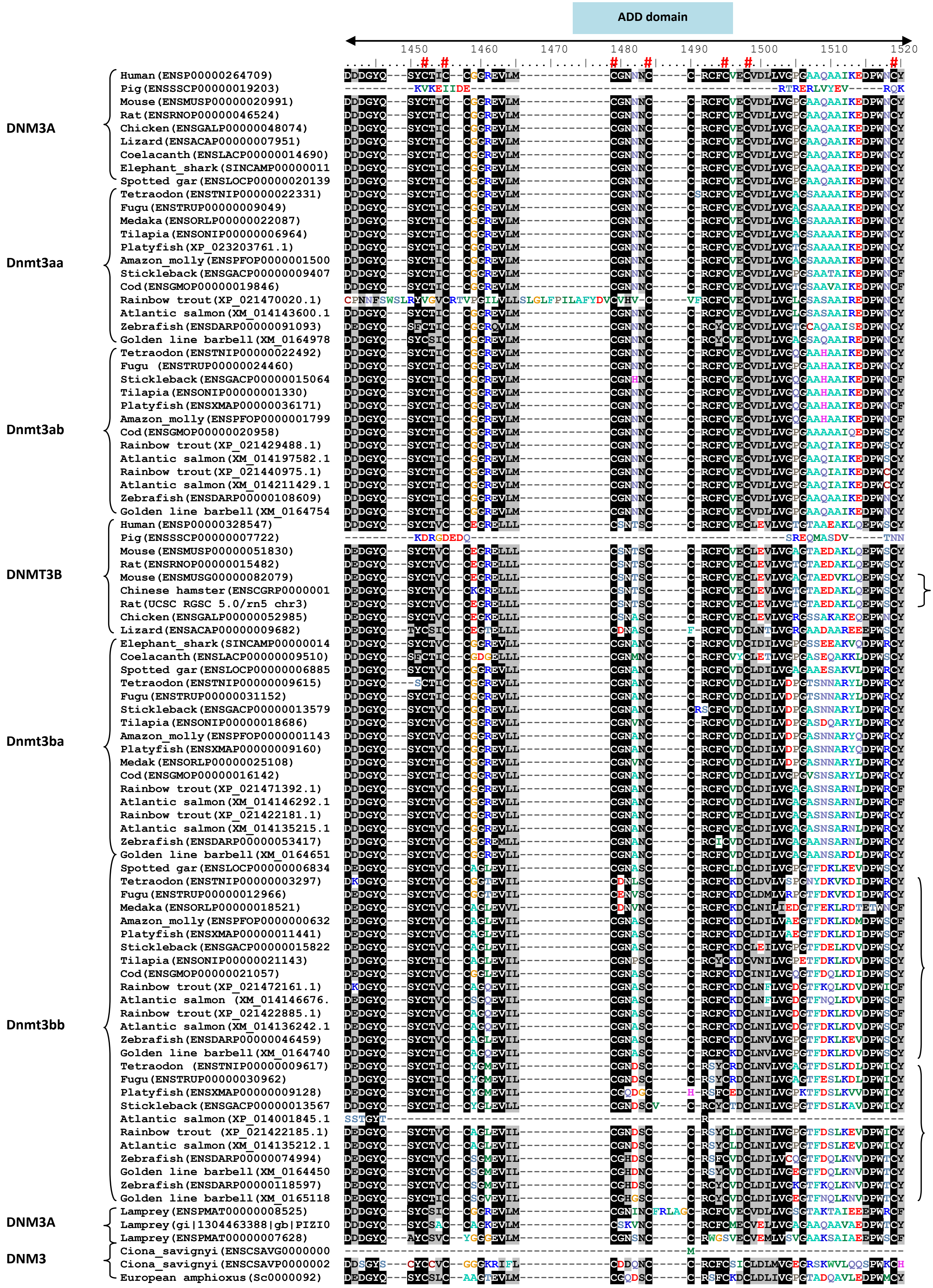

ADD domain
1450
1460
1470
1480
1490
1500
1510
1520
DNM3A
Dnmt3aa
Dnmt3ab
DNMT3B
Dnmt3ba
Dnmt3bb
DNM3A
DNM3
DNMT3C
Dnmt3bba
Dnmt3bbb
Human(ENSP00000264709)
Pig(ENSSSCP00000019203)
Mouse(ENSMUSP00000020991)
Rat(ENSRNOP00000046524)
Chicken(ENSGALP00000048074)
Lizard(ENSACAP00000007951)
Coelacanth(ENSLACP00000014690)
Elephant_shark(SINCAMP0000011
Spotted gar(ENSLOCP00000020139
Tetraodon(ENSTNIP00000022331)
Fugu(ENSTRUP00000009049)
Medaka(ENSORLP00000022087)
Tilapia(ENSONIP00000006964)
Platyfish(XP_023203761.1)
Amazon_molly(ENSPFOP0000001500
Stickleback(ENSGACP00000009407
Cod(ENSGMOP00000019846)
Rainbow trout(XP_021470020.1)
Atlantic salmon(XM_014143600.1
Zebrafish(ENSDARP00000091093)
Golden line barbell(XM_0164978
Tetraodon(ENSTNIP00000022492)
Fugu (ENSTRUP00000024460)
Stickleback(ENSGACP00000015064
Tilapia(ENSONIP00000001330)
Platyfish(ENSXMAP00000036171)
Amazon_molly(ENSPFOP0000001799
Cod(ENSGMOP00000020958)
Rainbow trout(XP_021429488.1)
Atlantic salmon(XM_014197582.1
Rainbow trout(XP_021440975.1)
Atlantic salmon(XM_014211429.1
Zebrafish(ENSDARP00000108609)
Golden line barbell(XM_0164754
Human(ENSP00000328547)
Pig(ENSSSCP00000007722)
Mouse(ENSMUSP00000051830)
Rat(ENSRNOP00000015482)
Mouse(ENSMUSG00000082079)
Chinese hamster(ENSCGRP0000001
Rat(UCSC RGSC 5.0/rn5 chr3)
Chicken(ENSGALP00000052985)
Lizard(ENSACAP00000009682)
Elephant_shark(SINCAMP00000014
Coelacanth(ENSLACP00000009510)
Spotted gar(ENSLOCP00000006885
Tetraodon(ENSTNIP00000009615)
Fugu(ENSTRUP00000031152)
Stickleback(ENSGACP00000013579
Tilapia(ENSONIP00000018686)
Amazon_molly(ENSPFOP0000001143
Platyfish(ENSXMAP00000009160)
Medak(ENSORLP00000025108)
Cod(ENSGMOP00000016142)
Rainbow trout(XP_021471392.1)
Atlantic salmon(XM_014146292.1
Rainbow trout(XP_021422181.1)
Atlantic salmon(XM_014135215.1
Zebrafish(ENSDARP00000053417)
Golden line barbell(XM_0164651
Spotted gar(ENSLOCP00000006834
Tetraodon(ENSTNIP00000003297)
Fugu(ENSTRUP00000012966)
Medaka(ENSORLP00000018521)
Amazon_molly(ENSPFOP0000000632
Platyfish(ENSXMAP00000011441)
Stickleback(ENSGACP00000015822
Tilapia(ENSONIP00000021143)
Cod(ENSGMOP00000021057)
Rainbow trout(XP_021472161.1)
Atlantic salmon (XM_014146676.
Rainbow trout(XP_021422885.1)
Atlantic salmon(XM_014136242.1
Zebrafish(ENSDARP00000046459)
Golden line barbell(XM_0164740
Tetraodon (ENSTNIP00000009617)
Fugu(ENSTRUP00000030962)
Platyfish(ENSXMAP00000009128)
Stickleback(ENSGACP00000013567
Atlantic salmon(XP_014001845.1
Rainbow trout (XP_021422185.1)
Atlantic salmon(XM_014135212.1
Zebrafish(ENSDARP00000074994)
Golden line barbell(XM_0164450
Zebrafish(ENSDARP00000118597)
Golden line barbell(XM_0165118
Lamprey(ENSPMAT00000008525)
Lamprey(gi|1304463388|gb|PIZI0
Lamprey(ENSPMAT00000007628)
Ciona_savignyi(ENSCSAVG0000000
Ciona_savignyi(ENSCSAVP0000002
European amphioxus(Sc0000092)

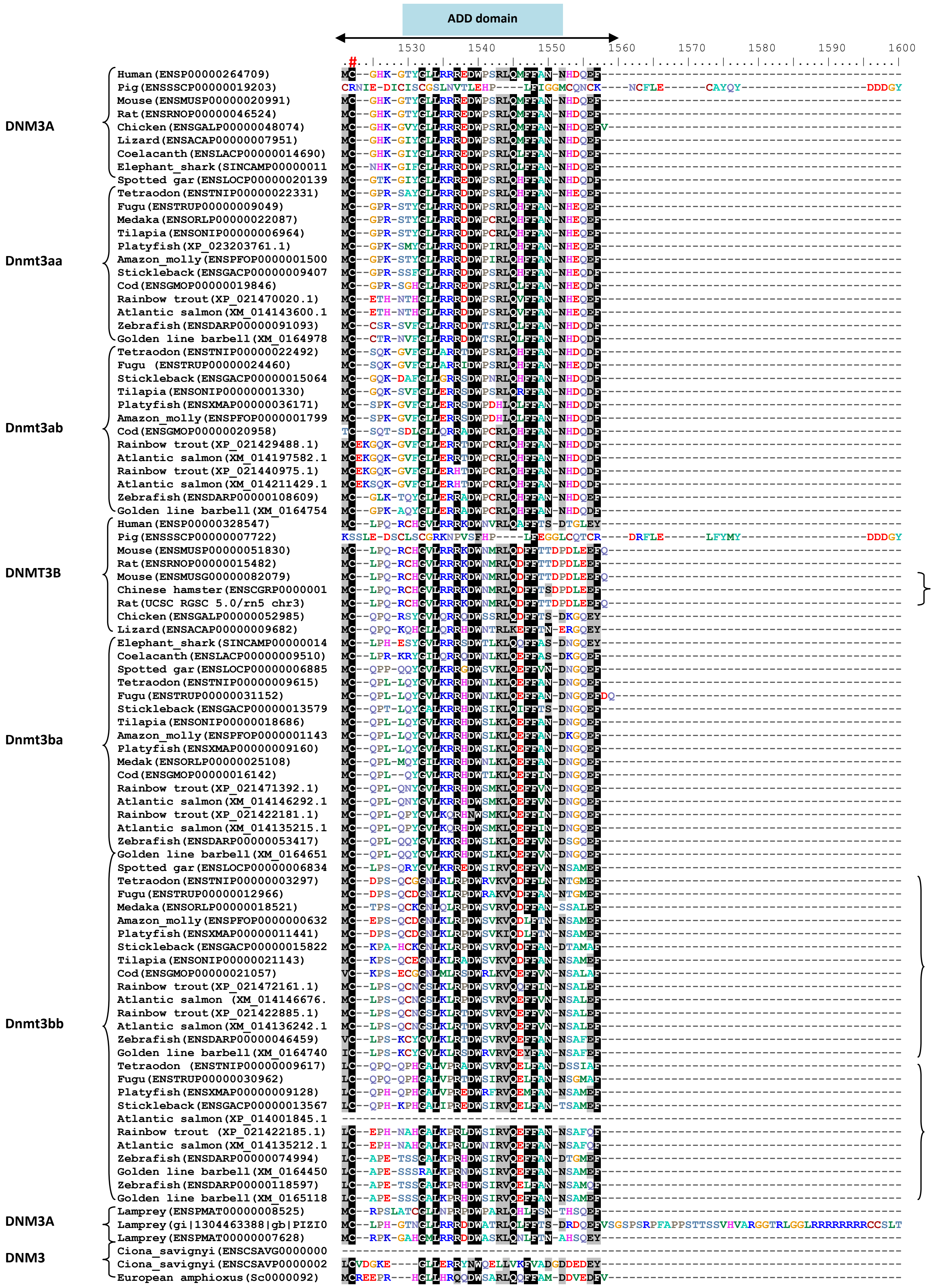

ADD domain
1530
1540
1550
1560
1570
1580
1590
1600
#
DNM3A
Human(ENSP00000264709)
Pig(ENSSSCP00000019203)
Mouse(ENSMUSP00000020991)
Rat(ENSRNOP00000046524)
Chicken(ENSGALP00000048074)
Lizard(ENSACAP00000007951)
Coelacanth(ENSLACP00000014690)
Elephant_shark(SINCAMP00000011
Spotted gar(ENSLOCP00000020139
Dnmt3aa
Tetraodon(ENSTNIP00000022331)
Fugu(ENSTRUP00000009049)
Medaka(ENSORLP00000022087)
Tilapia(ENSONIP00000006964)
Platyfish(XP_023203761.1)
Amazon_molly(ENSPFOP0000001500
Stickleback(ENSGACP00000009407
Cod(ENSGMOP00000019846)
Rainbow trout(XP_021470020.1)
Atlantic salmon(XM_014143600.1
Zebrafish(ENSDARP00000091093)
Golden line barbell(XM_0164978
Dnmt3ab
Tetraodon(ENSTNIP00000022492)
Fugu (ENSTRUP00000024460)
Stickleback(ENSGACP00000015064
Tilapia(ENSONIP00000001330)
Platyfish(ENSXMAP00000036171)
Amazon_molly(ENSPFOP0000001799
Cod(ENSGMOP00000020958)
Rainbow trout(XP_021429488.1)
Atlantic salmon(XM_014197582.1
Rainbow trout(XP_021440975.1)
Atlantic salmon(XM_014211429.1
Zebrafish(ENSDARP00000108609)
Golden line barbell(XM_0164754
DNMT3B
Human(ENSP00000328547)
Pig(ENSSSCP00000007722)
Mouse(ENSMUSP00000051830)
Rat(ENSRNOP00000015482)
Mouse(ENSMUSG00000082079)
Chinese hamster(ENSCGRP0000001
Rat(UCSC RGSC 5.0/rn5 chr3)
Chicken(ENSGALP00000052985)
Lizard(ENSACAP00000009682)
DNMT3C
Dnmt3ba
Elephant_shark(SINCAMP00000014
Coelacanth(ENSLACP00000009510)
Spotted gar(ENSLOCP00000006885
Tetraodon(ENSTNIP00000009615)
Fugu(ENSTRUP00000031152)
Stickleback(ENSGACP00000013579
Tilapia(ENSONIP00000018686)
Amazon_molly(ENSPFOP0000001143
Platyfish(ENSXMAP00000009160)
Medak(ENSORLP00000025108)
Cod(ENSGMOP00000016142)
Rainbow trout(XP_021471392.1)
Atlantic salmon(XM_014146292.1
Rainbow trout(XP_021422181.1)
Atlantic salmon(XM_014135215.1
Zebrafish(ENSDARP00000053417)
Golden line barbell(XM_0164651
Dnmt3bb
Spotted gar(ENSLOCP00000006834
Tetraodon(ENSTNIP00000003297)
Fugu(ENSTRUP00000012966)
Medaka(ENSORLP00000018521)
Amazon_molly(ENSPFOP0000000632
Platyfish(ENSXMAP00000011441)
Stickleback(ENSGACP00000015822
Tilapia(ENSONIP00000021143)
Cod(ENSGMOP00000021057)
Rainbow trout(XP_021472161.1)
Atlantic salmon (XM_014146676.
Rainbow trout(XP_021422885.1)
Atlantic salmon(XM_014136242.1
Zebrafish(ENSDARP00000046459)
Golden line barbell(XM_0164740
Dnmt3bba
Tetraodon (ENSTNIP00000009617)
Fugu(ENSTRUP00000030962)
Platyfish(ENSXMAP00000009128)
Stickleback(ENSGACP00000013567
Atlantic salmon(XP_014001845.1
Rainbow trout (XP_021422185.1)
Atlantic salmon(XM_014135212.1
Zebrafish(ENSDARP00000074994)
Golden line barbell(XM_0164450
Zebrafish(ENSDARP00000118597)
Golden line barbell(XM_0165118
Dnmt3bbb
DNM3A
Lamprey(ENSPMAT00000008525)
Lamprey(gi|1304463388|gb|PIZI0
Lamprey(ENSPMAT00000007628)
DNM3
Ciona_savignyi(ENSCSAVG0000000
Ciona_savignyi(ENSCSAVP0000002
European amphioxus(Sc0000092)

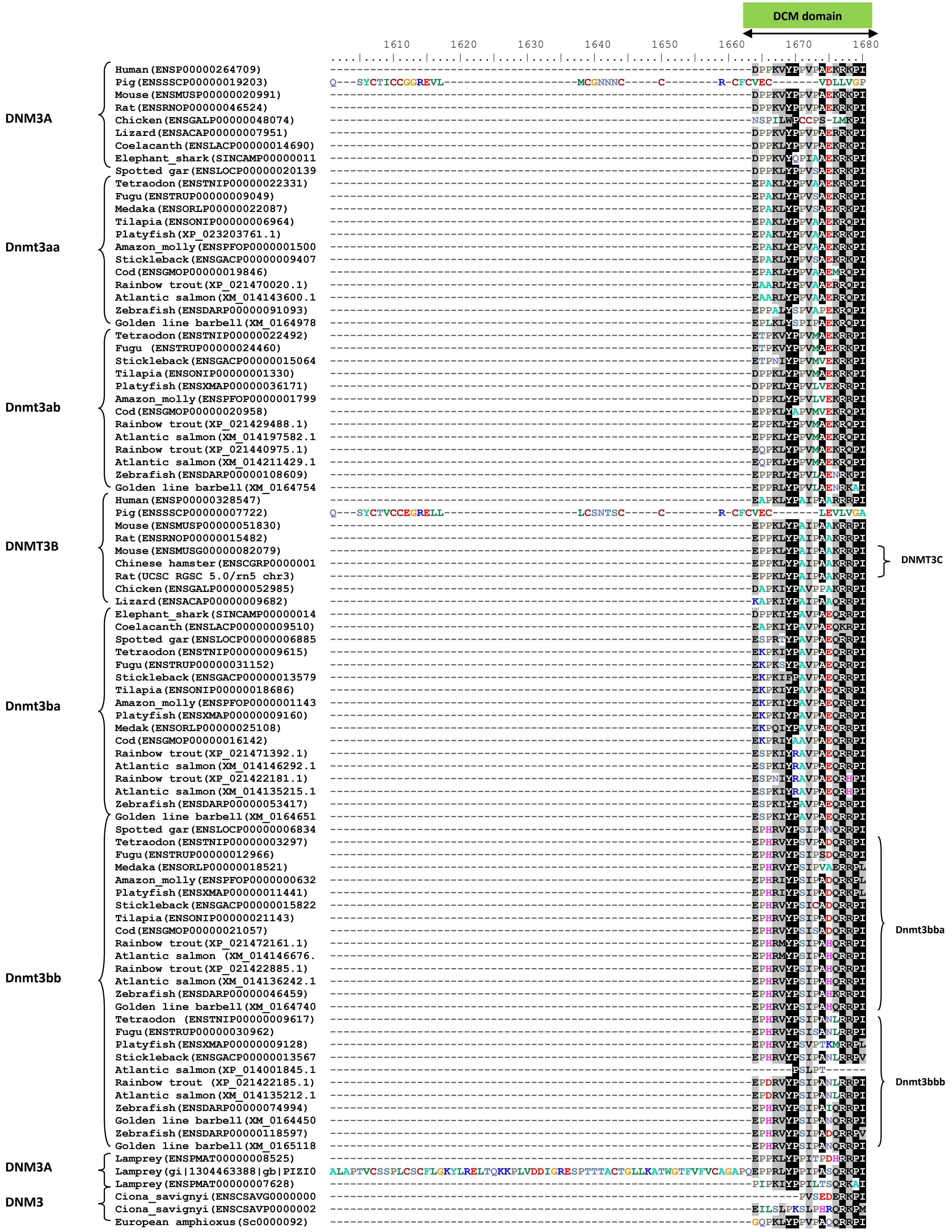

DCM domain
1610
1620
1630
1640
1650
1660
1670
1680
DNM3A
Human (ENSP00000264709)
Pig (ENSSSCP00000019203)
Mouse (ENSMUSP00000020991)
Rat (ENSRNOP00000046524)
Chicken (ENSGALP00000048074)
Lizard (ENSACAP00000007951)
Coelacanth (ENSLACP00000014690)
Elephant_shark (SINCAMP00000011
Spotted gar (ENSLOCP00000020139
Dnmt3aa
Tetraodon (ENSTNIP00000022331)
Fugu (ENSTRUP00000009049)
Medaka (ENSORLP00000022087)
Tilapia (ENSONIP00000006964)
Platyfish (XP_023203761.1)
Amazon_molly (ENSPFOP0000001500
Stickleback (ENSGACP00000009407
Cod (ENSGMOP00000019846)
Rainbow trout (XP_021470020.1)
Atlantic salmon (XM_014143600.1
Zebrafish (ENSDARP00000091093)
Golden line barbell (XM_0164978
Dnmt3ab
Tetraodon (ENSTNIP00000022492)
Fugu (ENSTRUP00000024460)
Stickleback (ENSGACP00000015064
Tilapia (ENSONIP00000001330)
Platyfish (ENSXMAP00000036171)
Amazon_molly (ENSPFOP0000001799
Cod (ENSGMOP00000020958)
Rainbow trout (XP_021429488.1)
Atlantic salmon (XM_014197582.1
Rainbow trout (XP_021440975.1)
Atlantic salmon (XM_014211429.1
Zebrafish (ENSDARP00000108609)
Golden line barbell (XM_0164754
DNMT3B
Human (ENSP00000328547)
Pig (ENSSSCP00000007722)
Mouse (ENSMUSP00000051830)
Rat (ENSRNOP00000015482)
Mouse (ENSMUSG00000082079)
Chinese hamster (ENSCGRP0000001
Rat (UCSC RGSC 5.0/rn5 chr3)
Chicken (ENSGALP00000052985)
Lizard (ENSACAP00000009682)
DNMT3C
Dnmt3ba
Elephant_shark (SINCAMP00000014
Coelacanth (ENSLACP00000009510)
Spotted gar (ENSLOCP00000006885
Tetraodon (ENSTNIP00000009615)
Fugu (ENSTRUP00000031152)
Stickleback (ENSGACP00000013579
Tilapia (ENSONIP00000018686)
Amazon_molly (ENSPFOP0000001143
Platyfish (ENSXMAP00000009160)
Medak (ENSORLP00000025108)
Cod (ENSGMOP00000016142)
Rainbow trout (XP_021471392.1)
Atlantic salmon (XM_014146292.1
Rainbow trout (XP_021422181.1)
Atlantic salmon (XM_014135215.1
Zebrafish (ENSDARP00000053417)
Golden line barbell (XM_0164651
Dnmt3bb
Spotted gar (ENSLOCP00000006834
Tetraodon (ENSTNIP00000003297)
Fugu (ENSTRUP00000012966)
Medaka (ENSORLP00000018521)
Amazon_molly (ENSPFOP0000000632
Platyfish (ENSXMAP00000011441)
Stickleback (ENSGACP00000015822
Tilapia (ENSONIP00000021143)
Cod (ENSGMOP00000021057)
Rainbow trout (XP_021472161.1)
Atlantic salmon (XM_014146676.
Rainbow trout (XP_021422885.1)
Atlantic salmon (XM_014136242.1
Zebrafish (ENSDARP00000046459)
Golden line barbell (XM_0164740
Dnmt3bba
Tetraodon (ENSTNIP00000009617)
Fugu (ENSTRUP00000030962)
Platyfish (ENSXMAP00000009128)
Stickleback (ENSGACP00000013567
Atlantic salmon (XP_014001845.1
Rainbow trout (XP_021422185.1)
Atlantic salmon (XM_014135212.1
Zebrafish (ENSDARP00000074994)
Golden line barbell (XM_0164450
Zebrafish (ENSDARP00000118597)
Golden line barbell (XM_0165118
Dnmt3bbb
DNM3A
Lamprey (ENSPMAT00000008525)
Lamprey (gi|1304463388|gb|PIZI0
Lamprey (ENSPMAT00000007628)
DNM3
Ciona_savignyi (ENSCSAVG0000000
Ciona_savignyi (ENSCSAVP0000002
European amphioxus (Sc0000092)

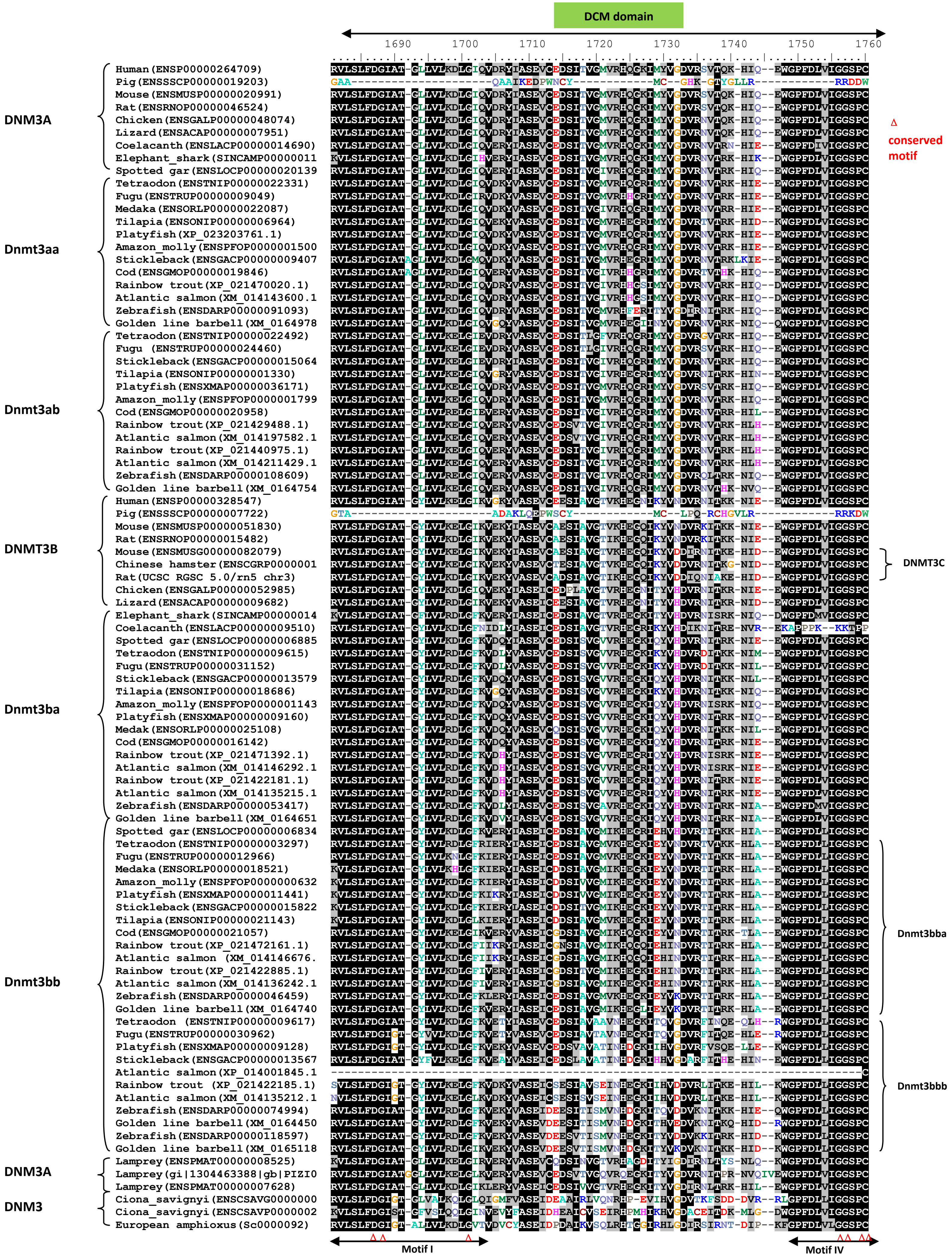

DCM domain
1690
1700
1710
1720
1730
1740
1750
1760
DNM3A
Human (ENSP00000264709)
Pig (ENSSSCP00000019203)
Mouse (ENSMUSP00000020991)
Rat (ENSRNOP00000046524)
Chicken (ENSGALP00000048074)
Lizard (ENSACAP00000007951)
Coelacanth (ENSLACP00000014690)
Elephant_shark (SINCAMP00000011
Spotted gar (ENSLOCP00000020139
Dnmt3aa
Tetraodon (ENSTNIP00000022331)
Fugu (ENSTRUP00000009049)
Medaka (ENSORLP00000022087)
Tilapia (ENSONIP00000006964)
Platyfish (XP_023203761.1)
Amazon_molly (ENSPFOP0000001500
Stickleback (ENSGACP00000009407
Cod (ENSGMOP00000019846)
Rainbow trout (XP_021470020.1)
Atlantic salmon (XM_014143600.1
Zebrafish (ENSDARP00000091093)
Golden line barbell (XM_0164978
Dnmt3ab
Tetraodon (ENSTNIP00000022492)
Fugu (ENSTRUP00000024460)
Stickleback (ENSGACP00000015064
Tilapia (ENSONIP00000001330)
Platyfish (ENSXMAP00000036171)
Amazon_molly (ENSPFOP0000001799
Cod (ENSGMOP00000020958)
Rainbow trout (XP_021429488.1)
Atlantic salmon (XM_014197582.1
Rainbow trout (XP_021440975.1)
Atlantic salmon (XM_014211429.1
Zebrafish (ENSDARP00000108609)
Golden line barbell (XM_0164754
DNMT3B
Human (ENSP00000328547)
Pig (ENSSSCP00000007722)
Mouse (ENSMUSP00000051830)
Rat (ENSRNOP00000015482)
Mouse (ENSMUSG00000082079)
Chinese hamster (ENSCGRP0000001
Rat (UCSC RGSC 5.0/rn5 chr3)
Chicken (ENSGALP00000052985)
Lizard (ENSACAP00000009682)
DNMT3C
Dnmt3ba
Elephant_shark (SINCAMP00000014
Coelacanth (ENSLACP00000009510)
Spotted gar (ENSLOCP00000006885
Tetraodon (ENSTNIP00000009615)
Fugu (ENSTRUP00000031152)
Stickleback (ENSGACP00000013579
Tilapia (ENSONIP00000018686)
Amazon_molly (ENSPFOP0000001143
Platyfish (ENSXMAP00000009160)
Medak (ENSORLP00000025108)
Cod (ENSGMOP00000016142)
Rainbow trout (XP_021471392.1)
Atlantic salmon (XM_014146292.1
Rainbow trout (XP_021422181.1)
Atlantic salmon (XM_014135215.1
Zebrafish (ENSDARP00000053417)
Golden line barbell (XM_0164651
Dnmt3bb
Spotted gar (ENSLOCP00000006834
Tetraodon (ENSTNIP00000003297)
Fugu (ENSTRUP00000012966)
Medaka (ENSORLP00000018521)
Amazon_molly (ENSPFOP0000000632
Platyfish (ENSXMAP00000011441)
Stickleback (ENSGACP00000015822
Tilapia (ENSONIP00000021143)
Cod (ENSGMOP00000021057)
Rainbow trout (XP_021472161.1)
Atlantic salmon (XM_014146676.
Rainbow trout (XP_021422885.1)
Atlantic salmon (XM_014136242.1
Zebrafish (ENSDARP00000046459)
Golden line barbell (XM_0164740
Dnmt3bba
Tetraodon (ENSTNIP00000009617)
Fugu (ENSTRUP00000030962)
Platyfish (ENSXMAP00000009128)
Stickleback (ENSGACP00000013567
Atlantic salmon (XP_014001845.1
Rainbow trout (XP_021422185.1)
Atlantic salmon (XM_014135212.1
Zebrafish (ENSDARP00000074994)
Golden line barbell (XM_0164450
Zebrafish (ENSDARP00000118597)
Golden line barbell (XM_0165118
Dnmt3bbb
DNM3A
Lamprey (ENSPMAT00000008525)
Lamprey (gi|1304463388|gb|PIZI0
Lamprey (ENSPMAT00000007628)
DNM3
Ciona_savignyi (ENSCSAVG0000000
Ciona_savignyi (ENSCSAVP0000002
European amphioxus (Sc0000092)
Δ conserved motif
Motif I
Motif IV

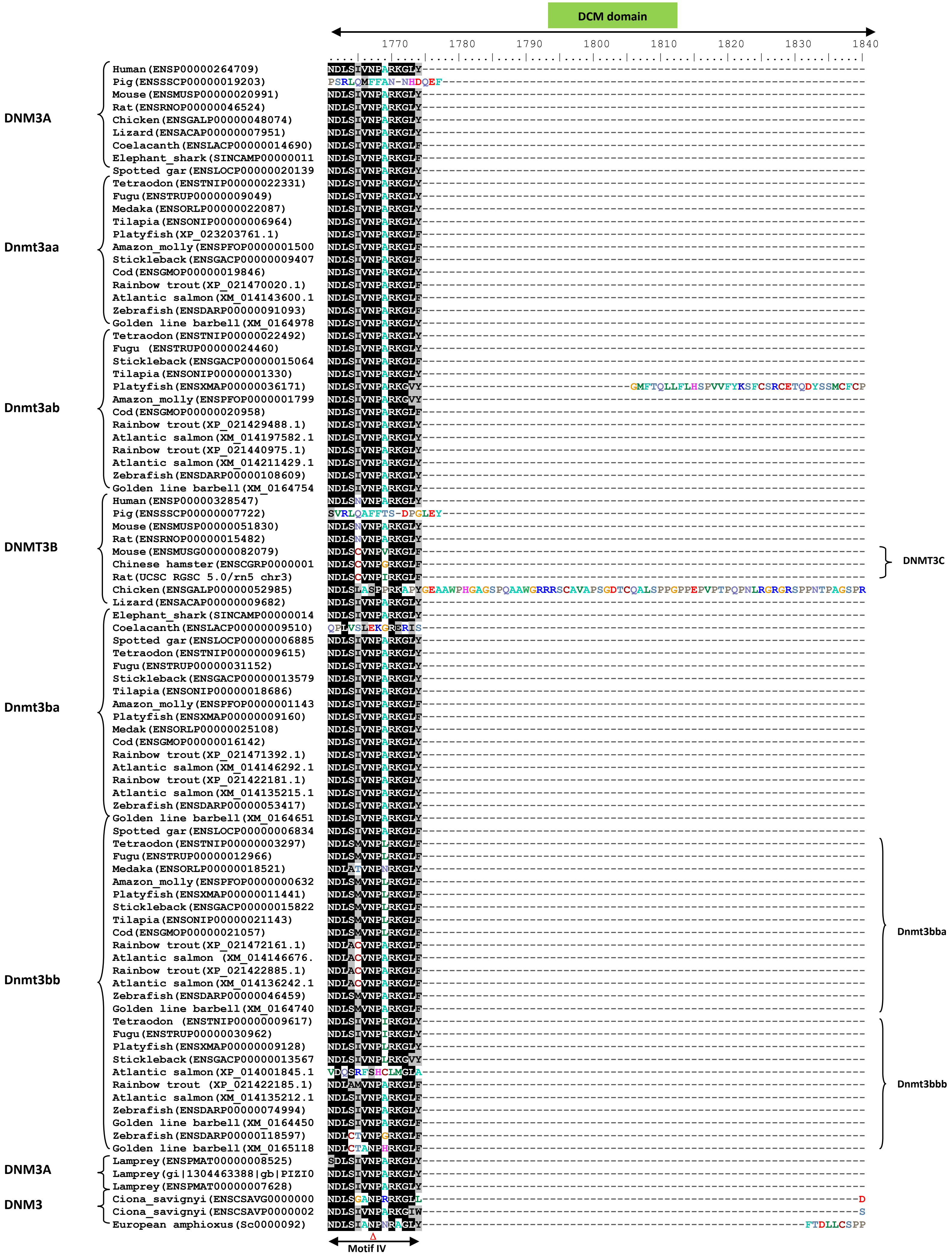

DCM domain
1770
1780
1790
1800
1810
1820
1830
1840
DNM3A
Human (ENSP00000264709) NDLSIVNPARKGLY
Pig (ENSSSCP00000019203) PSRLQMFFAN-NHDQEF
Mouse (ENSMUSP00000020991) NDLSIVNPARKGLY
Rat (ENSRNOP00000046524) NDLSIVNPARKGLY
Chicken (ENSGALP00000048074) NDLSIVNPARKGLY
Lizard (ENSACAP00000007951) NDLSIVNPARKGLY
Coelacanth (ENSLACP00000014690) NDLSIVNPARKGLF
Elephant_shark (SINCAMP00000011 NDLSIVNPARKGLF
Spotted gar (ENSLOCP00000020139 NDLSIVNPARKGLY
Dnmt3aa
Tetraodon (ENSTNIP00000022331) NDLSIVNPARKGLY
Fugu (ENSTRUP00000009049) NDLSIVNPARKGLY
Medaka (ENSORLP00000022087) NDLSIVNPARKGLY
Tilapia (ENSONIP00000006964) NDLSIVNPARKGLY
Platyfish (XP_023203761.1) NDLSIVNPARKGLF
Amazon_molly (ENSPFOP0000001500 NDLSIVNPARKGLF
Stickleback (ENSGACP00000009407 NDLSIVNPARKGLF
Cod (ENSGMOP00000019846) NDLSIVNPARKGLY
Rainbow trout (XP_021470020.1) NDLSIVNPARKGLF
Atlantic salmon (XM_014143600.1 NDLSIVNPARKGLF
Zebrafish (ENSDARP00000091093) NDLSIVNPARKGLF
Golden line barbell (XM_0164978 NDLSIVNPARKGLY
Dnmt3ab
Tetraodon (ENSTNIP00000022492) NDLSIVNPARKGLY
Fugu (ENSTRUP00000024460) NDLSIVNPARKGLY
Stickleback (ENSGACP00000015064 NDLSIVNPARKGLF
Tilapia (ENSONIP00000001330) NDLSIVNPARKGLY
Platyfish (ENSXMAP00000036171) NDLSIVNPARKGVY GMFTQLLFLHSPVVFYKSFCSRCETQDYSSMCFCP
Amazon_molly (ENSPFOP0000001799 NDLSIVNPARKGVY
Cod (ENSGMOP00000020958) NDLSIVNPARKGLF
Rainbow trout (XP_021429488.1) NDLSIVNPARKGLY
Atlantic salmon (XM_014197582.1 NDLSIVNPARKGLY
Rainbow trout (XP_021440975.1) NDLSIVNPARKGLY
Atlantic salmon (XM_014211429.1 NDLSIVNPARKGLY
Zebrafish (ENSDARP00000108609) NDLSIVNPARKGLY
Golden line barbell (XM_0164754 NDLSIVNPARKGLY
DNMT3B
Human (ENSP00000328547) NDLSNVNPARKGLY
Pig (ENSSSCP00000007722) SVRLQAFFTS-DPGLEY
Mouse (ENSMUSP00000051830) NDLSNVNPARKGLY
Rat (ENSRNOP00000015482) NDLSNVNPARKGLY
Mouse (ENSMUSG00000082079) NDLSCVNPVRKGLF
Chinese hamster (ENSCGRP0000001 NDLSCVNPGRKGLF
Rat (UCSC RGSC 5.0/rn5 chr3) NDLSCVNPIRKGLF
DNMT3C
Chicken (ENSGALP00000052985) NDLSLASPPRKAPYGEAAWPHGAGSPQAAWGRRRSCAVAPSGDTCQALSPPGPPEPVPTPQPNLRGRGRSPPNTPAGSPR
Lizard (ENSACAP00000009682) NDLSIVNPARKGLY
Dnmt3ba
Elephant_shark (SINCAMP00000014 NDLSIVNPARKGLY
Coelacanth (ENSLACP00000009510) QPLVSLEKGREERIS
Spotted gar (ENSLOCP00000006885 NDLSIVNPARKGLY
Tetraodon (ENSTNIP00000009615) NDLSIVNPARKGLY
Fugu (ENSTRUP00000031152) NDLSIVNPARKGLY
Stickleback (ENSGACP00000013579 NDLSIVNPARKGLY
Tilapia (ENSONIP00000018686) NDLSIVNPARKGLY
Amazon_molly (ENSPFOP0000001143 NDLSIVNPARKGLF
Platyfish (ENSXMAP00000009160) NDLSIVNPARKGLF
Medak (ENSORLP00000025108) NDLSIVNPARKGLY
Cod (ENSGMOP00000016142) NDLSIVNPARKGLY
Rainbow trout (XP_021471392.1) NDLSIVNPARKGLY
Atlantic salmon (XM_014146292.1 NDLSIVNPARKGLY
Rainbow trout (XP_021422181.1) NDLSIVNPARKGLY
Atlantic salmon (XM_014135215.1 NDLSIVNPARKGLY
Zebrafish (ENSDARP00000053417) NDLSIVNPARKGLY
Golden line barbell (XM_0164651 NDLSIVNPARKGLY
Dnmt3bb
Dnmt3bba
Spotted gar (ENSLOCP00000006834 NDLSIVNPARKGLF
Tetraodon (ENSTNIP00000003297) NDLSMVNPLRKGLF
Fugu (ENSTRUP00000012966) NDLSMVNPLRKGLF
Medaka (ENSORLP00000018521) NDIATVNPNRKGLY
Amazon_molly (ENSPFOP0000000632 NDLSMVNPLRKGLF
Platyfish (ENSXMAP00000011441) NDLSMVNPLRKGLF
Stickleback (ENSGACP00000015822 NDLSMVNPLRKGLF
Tilapia (ENSONIP00000021143) NDLSMVNPLRKGLF
Cod (ENSGMOP00000021057) NDLSMVNPLRKGLF
Rainbow trout (XP_021472161.1) NDIACVNPARKGLF
Atlantic salmon (XM_014146676. NDIACVNPARKGLF
Rainbow trout (XP_021422885.1) NDIACVNPARKGLF
Atlantic salmon (XM_014136242.1 NDIACVNPARKGLF
Zebrafish (ENSDARP00000046459) NDLSMVNPARKGLF
Golden line barbell (XM_0164740 NDLSMVNPARKGLF
Dnmt3bbb
Tetraodon (ENSTNIP00000009617) NDLSIVNPIRKGLY
Fugu (ENSTRUP00000030962) NDLSIVNPIRKGLY
Platyfish (ENSXMAP00000009128) NDLSIVNPLRKGLY
Stickleback (ENSGACP00000013567 NDLSIVNPLRKGVY
Atlantic salmon (XP_014001845.1 VDQSRFSHCLMGLA
Rainbow trout (XP_021422185.1) NDIAMVNPARKGLF
Atlantic salmon (XM_014135212.1 NDLSIVNPARKGLF
Zebrafish (ENSDARP00000074994) NDLSIVNPARKGLY
Golden line barbell (XM_0164450 NDLSIVNPARKGLF
Zebrafish (ENSDARP00000118597) NDLCTVNPGRKGLF
Golden line barbell (XM_0165118 NDLCTANPHRKGLF
DNM3A
Lamprey (ENSPMAT00000008525) SDLSIVNPARKGLY
Lamprey (gi|1304463388|gb|PIZI0 NDLSIVNPARKGLY
Lamprey (ENSPMAT00000007628) NDLSIVNPARKGLY
DNM3
Ciona_savignyi (ENSCSAVG0000000 NDLSGANPRRKGLL D
Ciona_savignyi (ENSCSAVP0000002 NDLSIVNPARKGIW S
European amphioxus (Sc0000092) NDLSIANPNRAGLY FTDLLCSPP
Motif IV

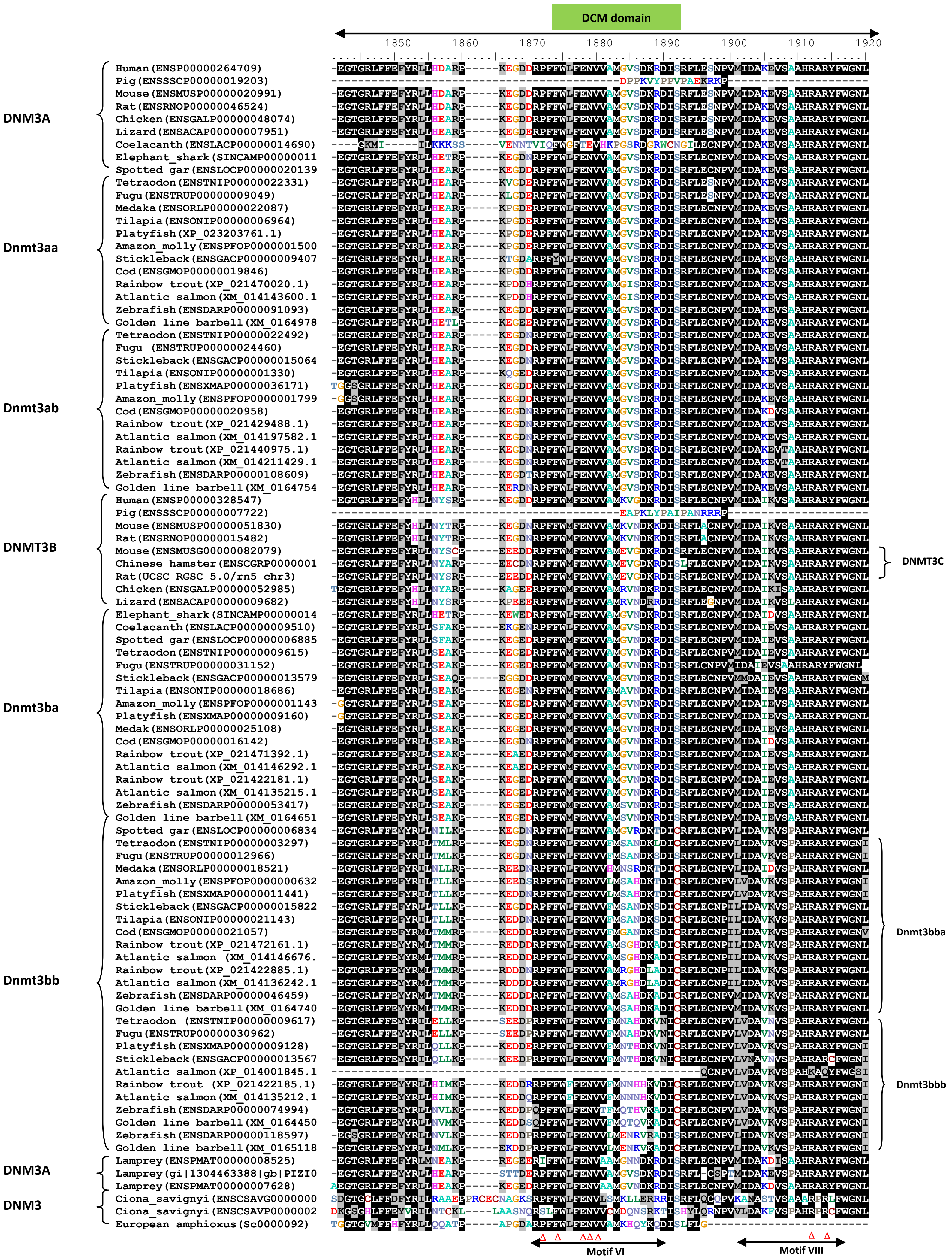
DCM domain
1850
1860
1870
1880
1890
1900
1910
1920
DNM3A
Human (ENSP00000264709)
Pig (ENSSSCP00000019203)
Mouse (ENSMUSP00000020991)
Rat (ENSRNOP00000046524)
Chicken (ENSGALP00000048074)
Lizard (ENSACAP00000007951)
Coelacanth (ENSLACP00000014690)
Elephant_shark (SINCAMP00000011
Spotted gar (ENSLOCP00000020139
Dnmt3aa
Tetraodon (ENSTNIP00000022331)
Fugu (ENSTRUP00000009049)
Medaka (ENSORLP00000022087)
Tilapia (ENSONIP00000006964)
Platyfish (XP_023203761.1)
Amazon_molly (ENSPFOP0000001500
Stickleback (ENSGACP00000009407
Cod (ENSGMOP00000019846)
Rainbow trout (XP_021470020.1)
Atlantic salmon (XM_014143600.1
Zebrafish (ENSDARP00000091093)
Golden line barbell (XM_0164978
Dnmt3ab
Tetraodon (ENSTNIP00000022492)
Fugu (ENSTRUP00000024460)
Stickleback (ENSGACP00000015064
Tilapia (ENSONIP00000001330)
Platyfish (ENSXMAP00000036171)
Amazon_molly (ENSPFOP0000001799
Cod (ENSGMOP00000020958)
Rainbow trout (XP_021429488.1)
Atlantic salmon (XM_014197582.1
Rainbow trout (XP_021440975.1)
Atlantic salmon (XM_014211429.1
Zebrafish (ENSDARP00000108609)
Golden line barbell (XM_0164754
DNMT3B
Human (ENSP00000328547)
Pig (ENSSSCP00000007722)
Mouse (ENSMUSP00000051830)
Rat (ENSRNOP00000015482)
Mouse (ENSMUSG00000082079)
Chinese hamster (ENSCGRP0000001
Rat (UCSC RGSC 5.0/rn5 chr3)
Chicken (ENSGALP00000052985)
Lizard (ENSACAP00000009682)
DNMT3C
Dnmt3ba
Elephant_shark (SINCAMP00000014
Coelacanth (ENSLACP00000009510)
Spotted gar (ENSLOCP00000006885
Tetraodon (ENSTNIP00000009615)
Fugu (ENSTRUP00000031152)
Stickleback (ENSGACP00000013579
Tilapia (ENSONIP00000018686)
Amazon_molly (ENSPFOP0000001143
Platyfish (ENSXMAP00000009160)
Medak (ENSORLP00000025108)
Cod (ENSGMOP00000016142)
Rainbow trout (XP_021471392.1)
Atlantic salmon (XM_014146292.1
Rainbow trout (XP_021422181.1)
Atlantic salmon (XM_014135215.1
Zebrafish (ENSDARP00000053417)
Golden line barbell (XM_0164651
Dnmt3bb
Spotted gar (ENSLOCP00000006834
Tetraodon (ENSTNIP00000003297)
Fugu (ENSTRUP00000012966)
Medaka (ENSORLP00000018521)
Amazon_molly (ENSPFOP0000000632
Platyfish (ENSXMAP00000011441)
Stickleback (ENSGACP00000015822
Tilapia (ENSONIP00000021143)
Cod (ENSGMOP00000021057)
Rainbow trout (XP_021472161.1)
Atlantic salmon (XM_014146676.
Rainbow trout (XP_021422885.1)
Atlantic salmon (XM_014136242.1
Zebrafish (ENSDARP00000046459)
Golden line barbell (XM_0164740
Dnmt3bba
Tetraodon (ENSTNIP00000009617)
Fugu (ENSTRUP00000030962)
Platyfish (ENSXMAP00000009128)
Stickleback (ENSGACP00000013567
Atlantic salmon (XP_014001845.1
Rainbow trout (XP_021422185.1)
Atlantic salmon (XM_014135212.1
Zebrafish (ENSDARP00000074994)
Golden line barbell (XM_0164450
Zebrafish (ENSDARP00000118597)
Golden line barbell (XM_0165118
Dnmt3bbb
DNM3A
Lamprey (ENSPMAT00000008525)
Lamprey (gi|1304463388|gb|PIZI0
Lamprey (ENSPMAT00000007628)
DNM3
Ciona_savignyi (ENSCSAVG0000000
Ciona_savignyi (ENSCSAVP0000002
European amphioxus (Sc0000092)
Motif VI
Motif VIII

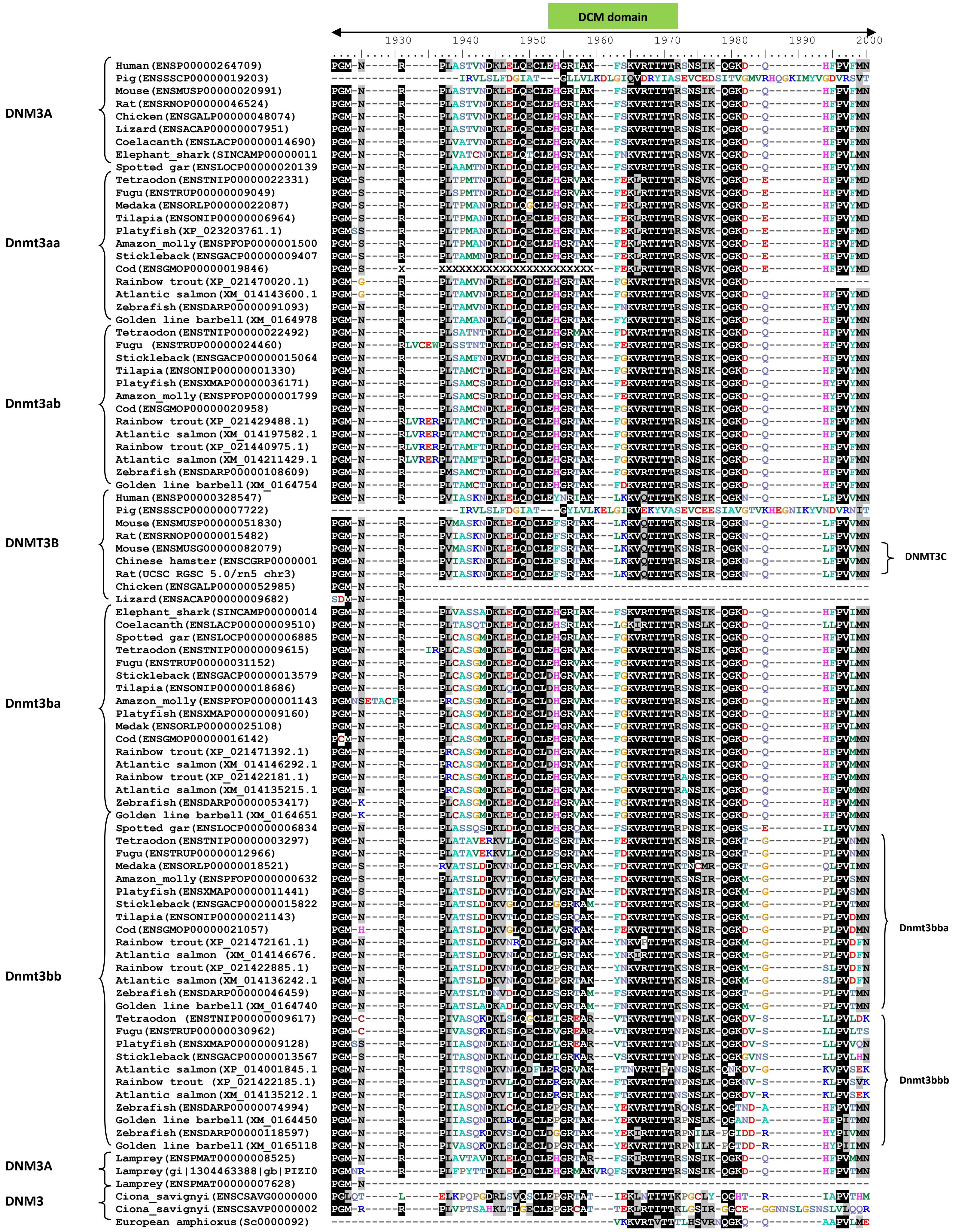
DCM domain
1930
1940
1950
1960
1970
1980
1990
2000
DNM3A
Human (ENSP00000264709)
Pig (ENSSSCP00000019203)
Mouse (ENSMUSP00000020991)
Rat (ENSRNOP00000046524)
Chicken (ENSGALP00000048074)
Lizard (ENSACAP00000007951)
Coelacanth (ENSLACP00000014690)
Elephant_shark (SINCAMP00000011
Spotted gar (ENSLOCP00000020139
Dnmt3aa
Tetraodon (ENSTNIP00000022331)
Fugu (ENSTRUP00000009049)
Medaka (ENSORLP00000022087)
Tilapia (ENSONIP00000006964)
Platyfish (XP_023203761.1)
Amazon_molly (ENSPFOP0000001500
Stickleback (ENSGACP00000009407
Cod (ENSGMOP00000019846)
Rainbow trout (XP_021470020.1)
Atlantic salmon (XM_014143600.1
Zebrafish (ENSDARP00000091093)
Golden line barbell (XM_0164978
Dnmt3ab
Tetraodon (ENSTNIP00000022492)
Fugu (ENSTRUP00000024460)
Stickleback (ENSGACP00000015064
Tilapia (ENSONIP00000001330)
Platyfish (ENSXMAP00000036171)
Amazon_molly (ENSPFOP0000001799
Cod (ENSGMOP00000020958)
Rainbow trout (XP_021429488.1)
Atlantic salmon (XM_014197582.1
Rainbow trout (XP_021440975.1)
Atlantic salmon (XM_014211429.1
Zebrafish (ENSDARP00000108609)
Golden line barbell (XM_0164754
DNMT3B
Human (ENSP00000328547)
Pig (ENSSSCP00000007722)
Mouse (ENSMUSP00000051830)
Rat (ENSRNOP00000015482)
Mouse (ENSMUSG00000082079)
Chinese hamster (ENSCGRP0000001
Rat (UCSC RGSC 5.0/rn5 chr3)
DNMT3C
Chicken (ENSGALP00000052985)
Lizard (ENSACAP00000009682)
Dnmt3ba
Elephant_shark (SINCAMP00000014
Coelacanth (ENSLACP00000009510)
Spotted gar (ENSLOCP00000006885
Tetraodon (ENSTNIP00000009615)
Fugu (ENSTRUP00000031152)
Stickleback (ENSGACP00000013579
Tilapia (ENSONIP00000018686)
Amazon_molly (ENSPFOP0000001143
Platyfish (ENSXMAP00000009160)
Medak (ENSORLP00000025108)
Cod (ENSGMOP00000016142)
Rainbow trout (XP_021471392.1)
Atlantic salmon (XM_014146292.1
Rainbow trout (XP_021422181.1)
Atlantic salmon (XM_014135215.1
Zebrafish (ENSDARP00000053417)
Golden line barbell (XM_0164651
Dnmt3bb
Spotted gar (ENSLOCP00000006834
Dnmt3bba
Tetraodon (ENSTNIP00000003297)
Fugu (ENSTRUP00000012966)
Medaka (ENSORLP00000018521)
Amazon_molly (ENSPFOP0000000632
Platyfish (ENSXMAP00000011441)
Stickleback (ENSGACP00000015822
Tilapia (ENSONIP00000021143)
Cod (ENSGMOP00000021057)
Rainbow trout (XP_021472161.1)
Atlantic salmon (XM_014146676.
Rainbow trout (XP_021422885.1)
Atlantic salmon (XM_014136242.1
Zebrafish (ENSDARP00000046459)
Golden line barbell (XM_0164740
Dnmt3bbb
Tetraodon (ENSTNIP00000009617)
Fugu (ENSTRUP00000030962)
Platyfish (ENSXMAP00000009128)
Stickleback (ENSGACP00000013567
Atlantic salmon (XP_014001845.1
Rainbow trout (XP_021422185.1)
Atlantic salmon (XM_014135212.1
Zebrafish (ENSDARP00000074994)
Golden line barbell (XM_0164450
Zebrafish (ENSDARP00000118597)
Golden line barbell (XM_0165118
DNM3A
Lamprey (ENSPMAT00000008525)
Lamprey (gi|1304463388|gb|PIZI0
Lamprey (ENSPMAT00000007628)
DNM3
Ciona_savignyi (ENSCSAVG0000000
Ciona_savignyi (ENSCSAVP0000002
European amphioxus (Sc0000092)

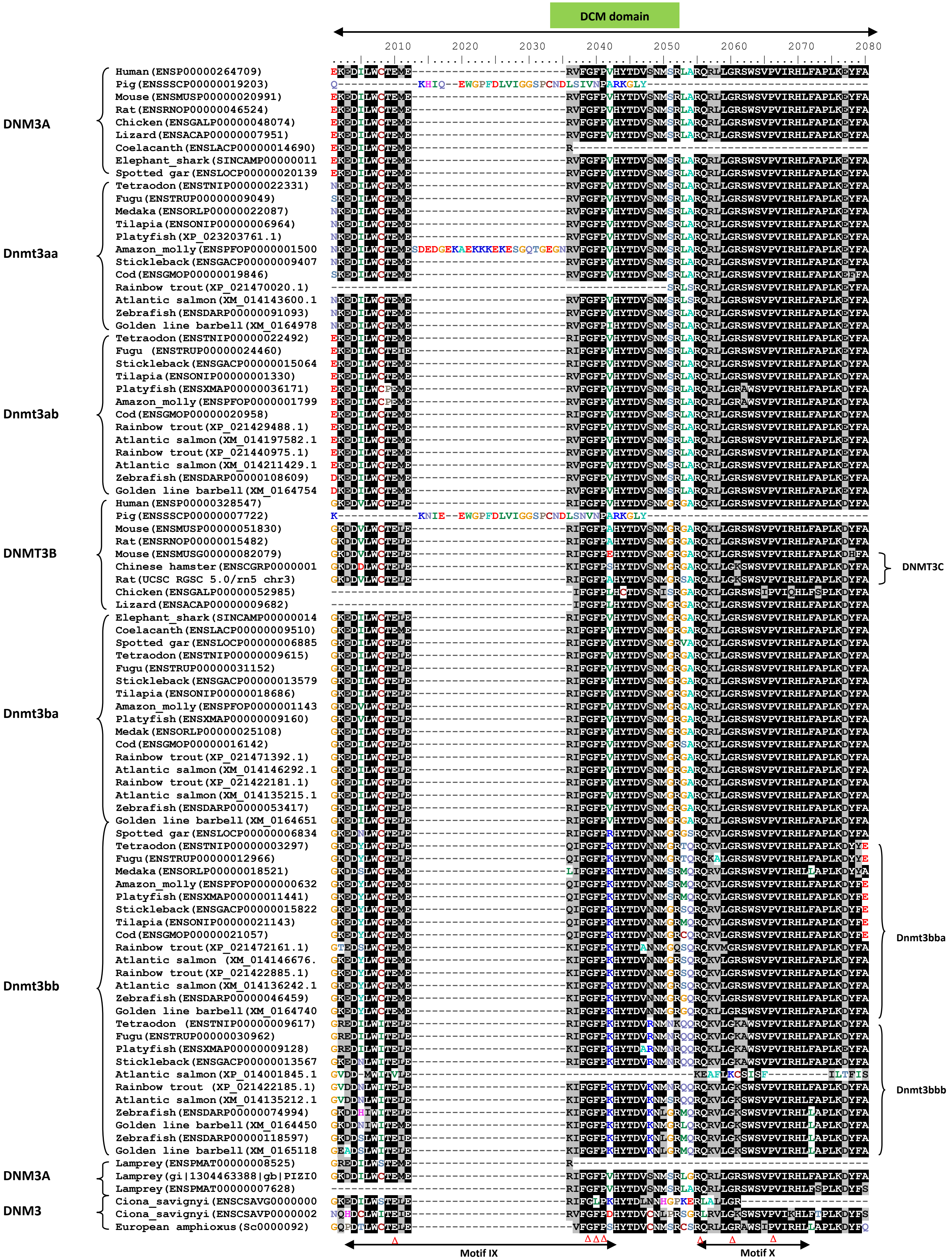
DCM domain
2010
2020
2030
2040
2050
2060
2070
2080
DNM3A
Dnmt3aa
Dnmt3ab
DNMT3B
DNMT3C
Dnmt3ba
Dnmt3bb
Dnmt3bba
Dnmt3bbb
DNM3A
DNM3
Motif IX
Motif X

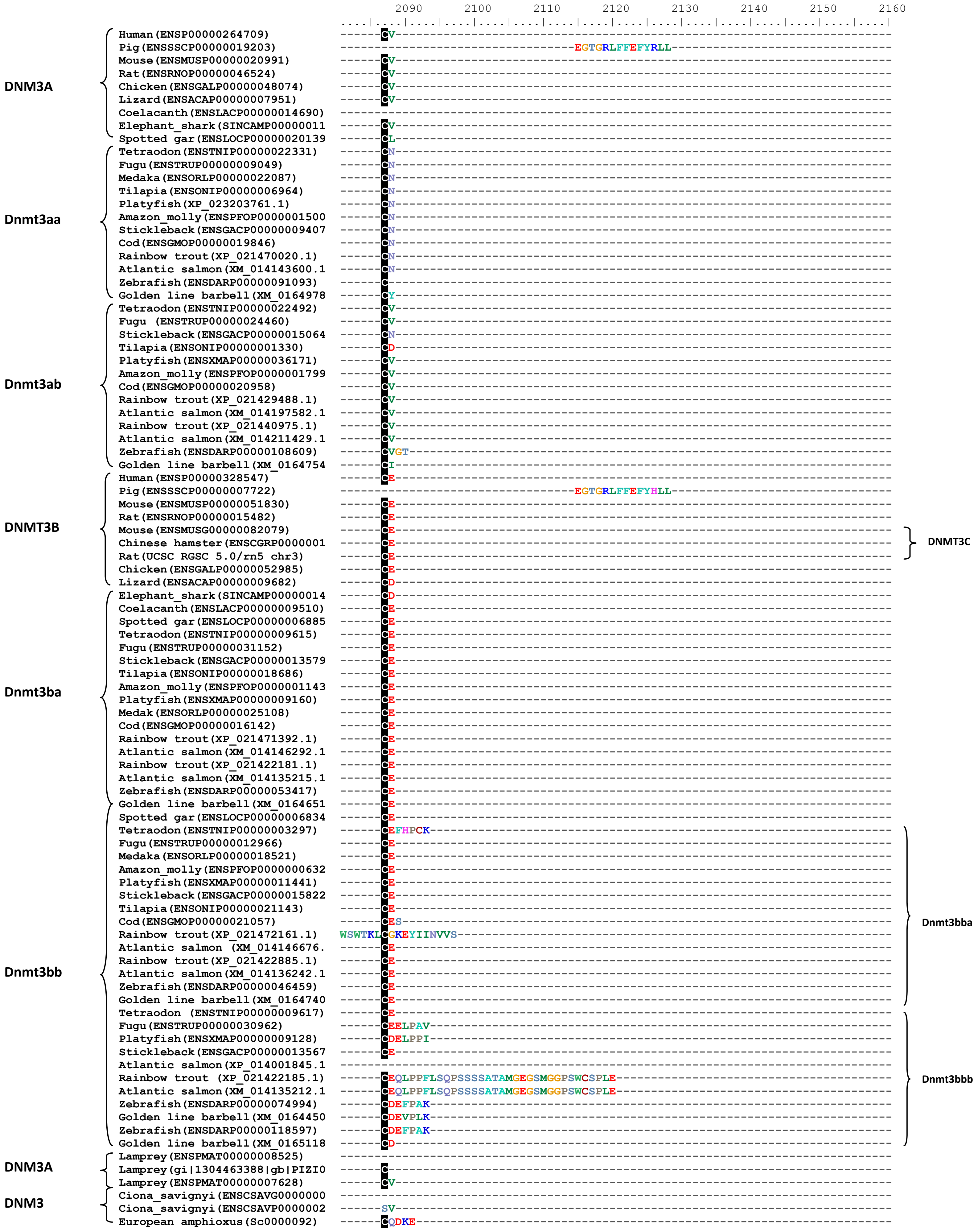
2090
2100
2110
2120
2130
2140
2150
2160
DNM3A
Human(ENSP00000264709)
Pig(ENSSSCP00000019203)
Mouse(ENSMUSP00000020991)
Rat(ENSRNOP00000046524)
Chicken(ENSGALP00000048074)
Lizard(ENSACAP00000007951)
Coelacanth(ENSLACP00000014690)
Elephant_shark(SINCAMP00000011
Spotted gar(ENSLOCP00000020139
Dnmt3aa
Tetraodon(ENSTNIP00000022331)
Fugu(ENSTRUP00000009049)
Medaka(ENSORLP00000022087)
Tilapia(ENSONIP00000006964)
Platyfish(XP_023203761.1)
Amazon_molly(ENSPFOP0000001500
Stickleback(ENSGACP00000009407
Cod(ENSGMOP00000019846)
Rainbow trout(XP_021470020.1)
Atlantic salmon(XM_014143600.1
Zebrafish(ENSDARP00000091093)
Golden line barbell(XM_0164978
Dnmt3ab
Tetraodon(ENSTNIP00000022492)
Fugu (ENSTRUP00000024460)
Stickleback(ENSGACP00000015064
Tilapia(ENSONIP00000001330)
Platyfish(ENSXMAP00000036171)
Amazon_molly(ENSPFOP0000001799
Cod(ENSGMOP00000020958)
Rainbow trout(XP_021429488.1)
Atlantic salmon(XM_014197582.1
Rainbow trout(XP_021440975.1)
Atlantic salmon(XM_014211429.1
Zebrafish(ENSDARP00000108609)
Golden line barbell(XM_0164754
DNMT3B
Human(ENSP00000328547)
Pig(ENSSSCP00000007722)
Mouse(ENSMUSP00000051830)
Rat(ENSRNOP00000015482)
Mouse(ENSMUSG00000082079)
Chinese hamster(ENSCGRP0000001
Rat(UCSC RGSC 5.0/rn5 chr3)
Chicken(ENSGALP00000052985)
Lizard(ENSACAP00000009682)
DNMT3C
Dnmt3ba
Elephant_shark(SINCAMP00000014
Coelacanth(ENSLACP00000009510)
Spotted gar(ENSLOCP00000006885
Tetraodon(ENSTNIP00000009615)
Fugu(ENSTRUP00000031152)
Stickleback(ENSGACP00000013579
Tilapia(ENSONIP00000018686)
Amazon_molly(ENSPFOP0000001143
Platyfish(ENSXMAP00000009160)
Medak(ENSORLP00000025108)
Cod(ENSGMOP00000016142)
Rainbow trout(XP_021471392.1)
Atlantic salmon(XM_014146292.1
Rainbow trout(XP_021422181.1)
Atlantic salmon(XM_014135215.1
Zebrafish(ENSDARP00000053417)
Golden line barbell(XM_0164651
Dnmt3bb
Spotted gar(ENSLOCP00000006834
Tetraodon(ENSTNIP00000003297)
Fugu(ENSTRUP00000012966)
Medaka(ENSORLP00000018521)
Amazon_molly(ENSPFOP0000000632
Platyfish(ENSXMAP00000011441)
Stickleback(ENSGACP00000015822
Tilapia(ENSONIP00000021143)
Cod(ENSGMOP00000021057)
Rainbow trout(XP_021472161.1)
Atlantic salmon (XM_014146676.
Rainbow trout(XP_021422885.1)
Atlantic salmon(XM_014136242.1
Zebrafish(ENSDARP00000046459)
Golden line barbell(XM_0164740
Dnmt3bba
Tetraodon (ENSTNIP00000009617)
Fugu(ENSTRUP00000030962)
Platyfish(ENSXMAP00000009128)
Stickleback(ENSGACP00000013567
Atlantic salmon(XP_014001845.1
Rainbow trout (XP_021422185.1)
Atlantic salmon(XM_014135212.1
Zebrafish(ENSDARP00000074994)
Golden line barbell(XM_0164450
Zebrafish(ENSDARP00000118597)
Golden line barbell(XM_0165118
Dnmt3bbb
DNM3A
Lamprey(ENSPMAT00000008525)
Lamprey(gi|1304463388|gb|PIZI0
Lamprey(ENSPMAT00000007628)
DNM3
Ciona_savignyi(ENSCSAVG0000000
Ciona_savignyi(ENSCSAVP0000002
European amphioxus(Sc0000092)
EGTGRLFFEFYRLL
EGTGRLFFEFYHLL
CEFHPCK
WSWTKLCGKEYIINVVS
CEELPAV
CDELPPI
CEQLPPFLSQPSSSSATAMGEGSMGGPSWCSPLE
CDEFPAK
CDEVPLK
CQDKE

Supplemental Fig.S3 Dnmt3 protein sequences alignment. All the protein sequences were aligned with the MUSCLE method in MEGA software. Conserved domains were noted above. Chromatin binding sites were marked with an asterisk (*); Conserved motifs were marked with a triangle (Δ); Zn binding sites were marked with a number sign (#).

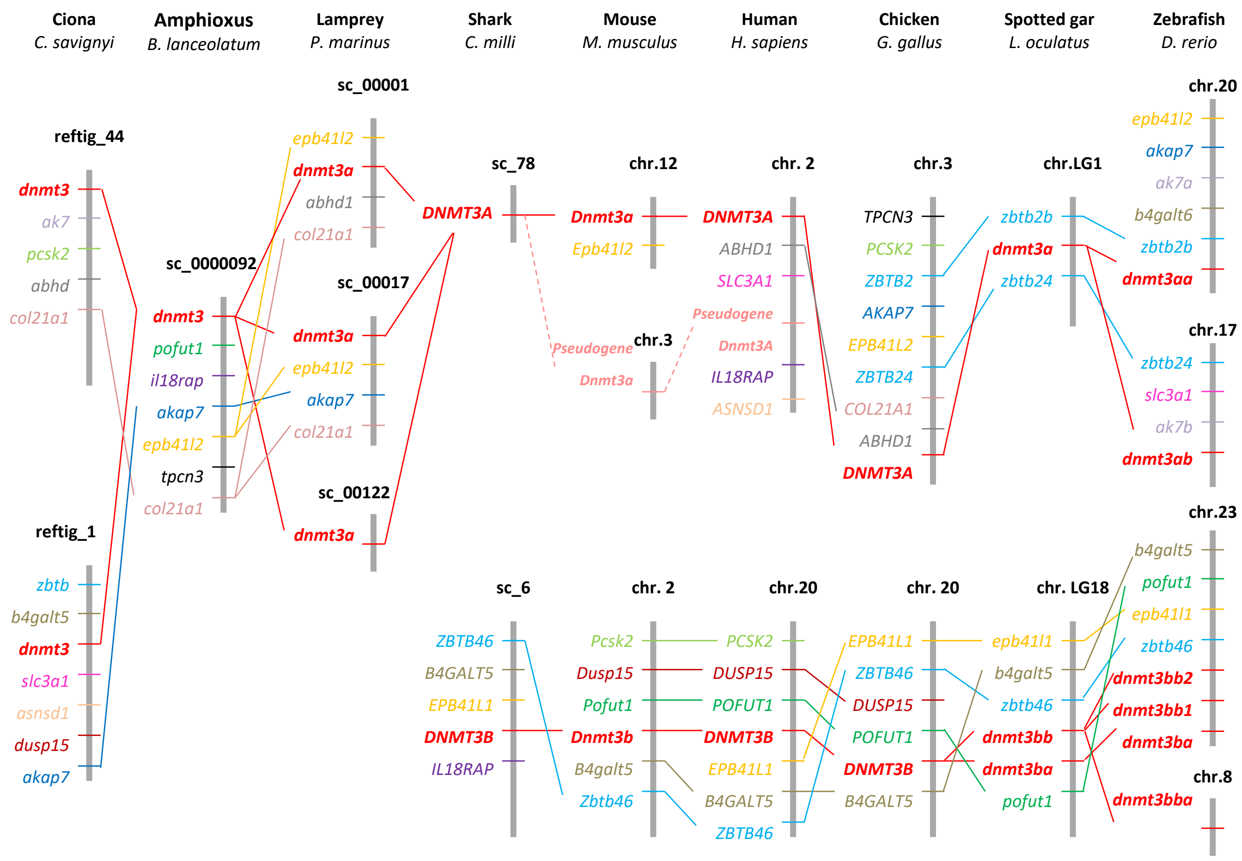

Supplementary Fig. S4 Conserved synteny around the *dnmt3* loci in chrodates. Data were collected with Genomicus software version 01.01. chr., chromosome; sc., scaffold.

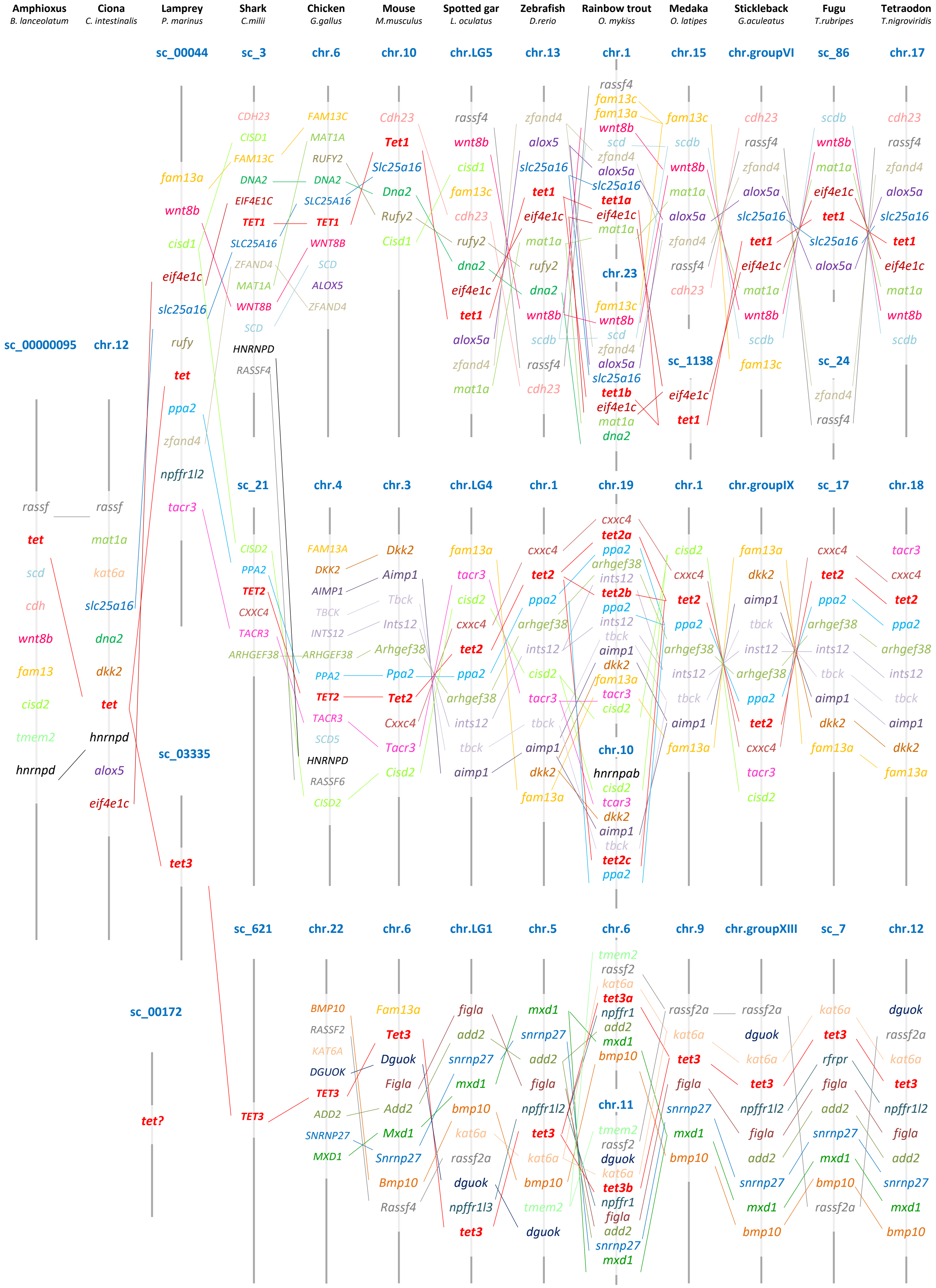

Supplementary Fig. S5 Conserved synteny around the *tet* loci in chrodates. Data were collected with Genomicus software version 01.01. chr., chromosome; sc., scaffold.

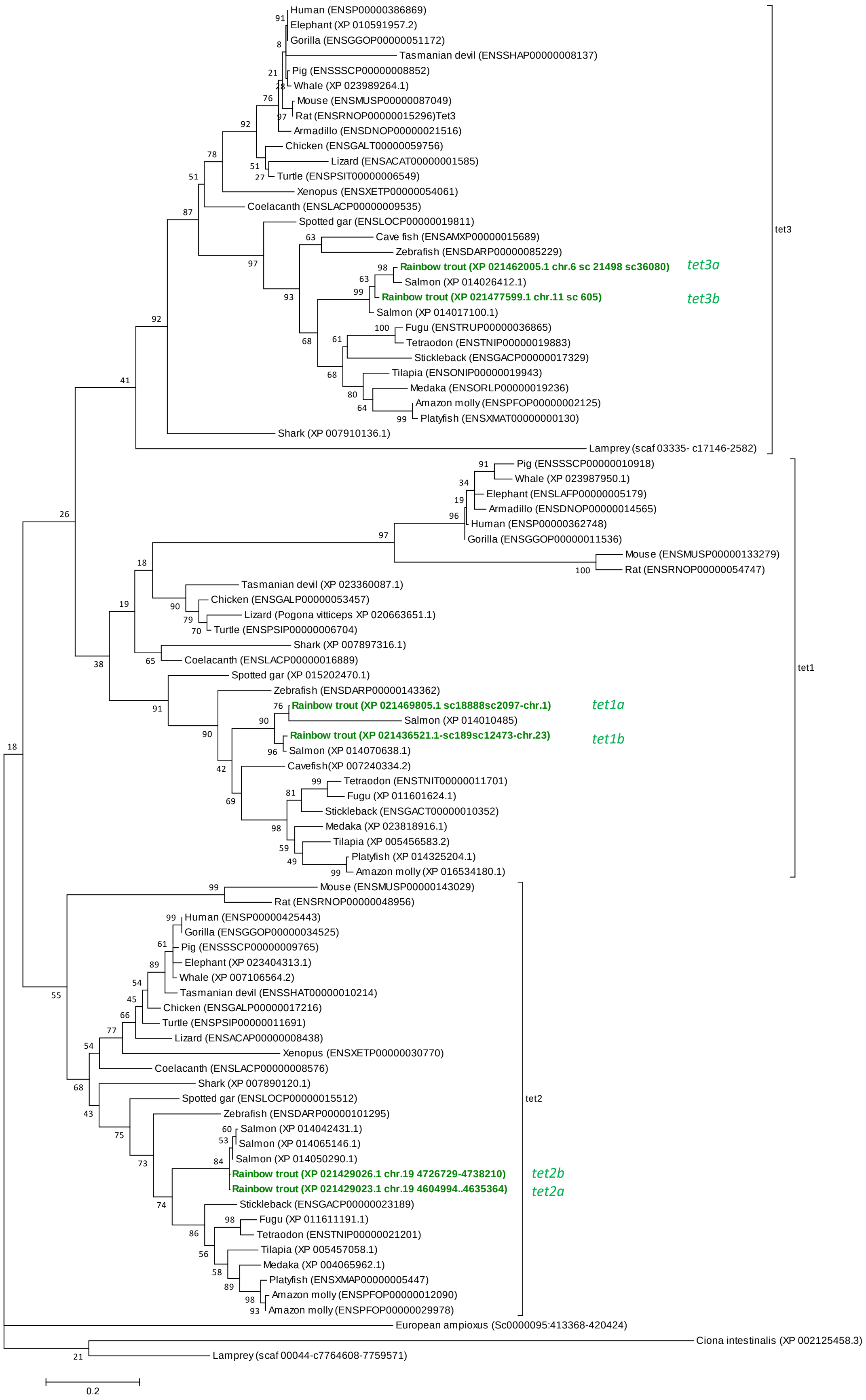

Supplementary Fig. S6: Pylogenetic of tet family genes. The phylogenetic trees were built using the Maximum Likelihood method based on the JTT matrix-based model in MEGAX software. The tree with the highest log likelihood (-14106.84) is shown. A discrete Gamma distribution was used to model evolutionary rate differences among sites (5 categories (+G, parameter = 0.5234)). The reliability of the inferred trees was estimated by the bootstrap method with 500 replications. All accession numbers are specified in parentheses.

```
                                              10        20        30        40        50        60        70        80        90
                                     ....|....|....|....|....|....|....|....|....|....|....|....|....|....|....|....|....|....|
European ampioxus (Sc0000095)        ------------------------------------------------------------------------------------------
Ciona intestinalis (XP_0021254       ------------------------------------------------------------------------------------------
Lamprey (scaf_00044)                 ------------------------------------------------------------------------------------------
Lamprey (scaf_03335)                 ------------------------------------------------------------------------------------------
Shark (XP_007897316.1)               ------------------------------------------------------------------------------------------
Lizard (XP_020663651.1)              -------------------------------------------MARDARSSRSVRKEEISKKKSSPVKKKSCQVTKDAQKTSPKAVCSAK
Chicken (ENSGALP00000053457)         -----------------------------------------ADMAHHARPSRLAKKEEIGKRKTNQGKKKSSQVRKKTTKTSTKAVSSGK
Turtle (ENSPSIP00000006704)          ------------------------------------------------------------------------------------------
Human (ENSP00000362748)              -----------------------------------------MSRSRHARPSRLVRKEDVNKKKKNSQLRKTTKG-ANKNVASVKTLSPGK
Gorilla (ENSGGOP00000011536)         -----------------------------------------MSRSRHARPSRLVRKEDVNKKKKNSQLRNTTKG-ANKNVASVKTLSPGK
Mouse (ENSMUSP00000133279)           -----------------------------------------MSRSRPAKPSKSVKTK-LQKKKDIQMKTKTSKQ-AVRHGASAKAVNPGK
Rat (ENSRNOP00000054747)             -----------------------------------------MSRSRPTKPSKSVKTK-LQKK-SSQMKKKISKQ-AVKHGASGKALTPGK
Pig (ENSSSCP00000010918)             -----------------------------------------MSRSRHARPSKLVRREDLNKKK-NSQLKKGSKP-ANKNVASVKTVNPGK
Whale (XP_023987950.1)               -----------------------------------------MSRSRHARPSKLVRREDLNKKK-NSQLKKGSKP-ANKNVATVKTVSPGK
Elephant (ENSLAFP00000005179)        -----------------------------------------MSRSRHARPSKLVRRDNLSKKK-SSQLKKTSKP-TNKNVASVKMMSPGK
Armadillo (ENSDNOP00000014565)       -----------------------------------------MSRSRHARPSRLVRREDLNKKK-NSQLKKTSKP-TNKNVALVKTVCPGK
Tasmanian_devil (XP_023360087.       ------------------------------------------------------------------------------------------
Coelacanth (ENSLACP00000016889       ------------------------------------------------------------------------------------------
Cavefish(XP_007240334.2)             ------------------------------------------------------------------MPRP----PKPQRKVHPPAKRNVR
Platyfish (XP_014325204.1)           --------------------------MPAPTKPTRRQTPAQRKKAQRGTS--------SRGQTCNTQKTP---AESTRSTSRS-------
Tilapia (XP_005456583.2)             --------------------------MPATTRPTRIQTSTQRKNTQKAIR--------TKRQTCSSNKTRGRAAQRTQKTSREPRSSKTR
Spotted_gar (XP_015202470.1)         --------------------------MPRSTKSTKKAPPRGQASNGKKKN-----IRAARRQTFNSNKTRGKPLKRKTEDAERSKNISRK
Stickleback (ENSGACT0000001035       ------------------------------------------------------------------------------------------
Tetraodon (ENSTNIT00000011701)       ------------------------------------------------------------------------------------------
Fugu (XP_011601624.1)                --------------------------MPATTKSTRRQKPAQRKPGPKGIS--------SKRQTCNSNKPKGRSAERRQKAPAKPKRSTTR
Zebrafish (ENSDARP00000143362)       -----------------------------------MSTLILLQTGSITLK-------------VQFLRRPQYCFSCRLSQTPPPSPSLTS
Amazon_molly (XP_016534180.1)        --------------------------MPAPTKPTRRHTPAQRKKAQRGTS--------TRGQTCNTQKTP---AESTRSTSRN-------
Medaka (XP_023818916.1)              --------------------------MPATTKSKKRKTPVRRKTQQIGTS--------TRGQICNSAKQGGRVAQKSKKHPAD-----SK
Rainbow_trout (XP_021469805.1)       --------------------------MPPTPKPPKRQPPPQTRNGCKVIPTTTTTTTTAKRQISNSSKFRGKAFESPQSTPKPPRRSTTR
Rainbow trout (XP_021436521.1)       --------------------------MPPIPKPSKRQSPPLTRNGCKVIP---TTT--AKRLNS--SKPRGKAFESPQPTSEPPRRSTTR
Salmon (XP_014010485)                --------------------------MPPTPKPPKRQPPPQTRNGCKVIP-TTTTTTTAKRQISNSSKSRGKAFESPQSTPKPPRRSTTR
Salmon (XP_014070638.1)              --------------------------MPPTPKPPKRQPPPLTRNGRKVIP---TTTSTAKRLNS--SKPRGKAFQSPQSTLEPPRRSTTR
Shark (XP_007890120.1)               -----------------------------------------MEQDRASHAEDKRLSPVLSIHYHPQEHHTECLSIKIENGEKVHQLPAVV
Human (ENSP00000425443)              --------------------MIQEEQIQLEGSLVDGPEASLMEQDRTNHVEGNRLSPFLIPSP--PICQTEPLATKLQNGSPLPERA---
Gorilla (ENSGGOP00000034525)         -------------------VDQEEAQIQLEGSLVDGPEASLMEQDRTNHVEGNRLSPLLIPSP--PICQTEPLATKLQNGSPLPERA---
Mouse (ENSMUSP00000143029)           -----------------------------------------MEQDRTTHAEGTRLSPFLIAPPS-PISHTEPLAVKLQNGSPLAERP---
Rat (ENSRNOP00000048956)             -----------------------------------------MEQDRTTHAEGTRQSPFLIASPS-PVSHTEPLAVKLQNGSPLAERP---
Pig (ENSSSCP00000009765)             -----------------------------------------MEQDRTNHVEGNRLSPFLIPSPS-PICQTEPLAIKLQNGSPLTERP---
Tasmanian_devil (ENSSHAT000000       -----------------------------------------MEQDRTNHIEGNRLSPFLIPPPS-HICQTESLAVKLQNGSPLTERP---
Whale (XP_007106564.2)               -----------------------------------------MEQDRRNHVEGNRLSPFLIPSSS-PICQTEPLAIKLQNGSPLTKRP---
Elephant (XP_023404313.1)            -----------------------------------------MEQDRTNHVEGNRLSPFLIPSLS-AICQTEPLAIKLQNGSPLTERP---
Lizard (ENSACAP00000008438)          ------------------------------------------------------------------------------------------
Chicken (ENSGALP00000017216)         -----------------------------------------MEQDRTIHVDGNRLSPFLISQPS-HICQADPSAVKLQNGSPATERP---
Turtle (ENSPSIP00000011691)          -----------------------------------------MEQDRTTHVDGNRLSPFLIPHPS-HVCHVEPLTVKLQNGSPLTERT---
Xenopus (ENSXETP00000030770)         -----------------------------------------MEEDRTNHDEGNRLSPTLLYSS----SACQNKNGLLQNGN-LPTDK---
Coelacanth (ENSLACP00000008576       --------------------LIFFFQISSEGSLVDGPGAGQMEQDRASHVEGKRLSPLLRPQQ------IGHLAEKLQNGSPLPETV---
Amazon_molly (ENSPFOP000000120       -----------------------------------------METEEAKHEAEESL--MLGQFG-----ASHHHSHKFQNGARCSQED---
Amazon_molly (ENSPFOP000000299       -----------------------------------------METEEAKHEAEESL--MLGQFG-----ASHHHSHKFQNGARCSQED---
Fugu (XP_011611191.1)                -----------------------------------------MERKQARHETEESL--ILAQFG-----SSH-ISHKFQNGGPSTEGE---
Medaka (XP_004065962.1)              -----------------------------------------METDQAKHEKEESS--MIAQFG-----IPHSISPRLQNGCHFSEG----
Platyfish (ENSXMAP00000005447)       -----------------------------------------METEEAKHEAEESL--MLGQFG-----TSHHHSHKFQNGARCSQED---
Spotted _gar (ENSLOCP000000155       -----------------------------------------MEKERASHVADERL--IFTQSSN--VHQSENLITKLHNGNQLQEVP---
Stickleback (ENSGACP0000002318       ------------------------------------------------------------------------------------------
Tilapia (XP_005457058.1)             -----------------------------------------METEEKRHEKEESL--ILEQFG-----TSHNIPHKLQNGGQFSEGN---
Tetraodon (ENSTNIP00000021201)       ------------------------------------------------------------------------------------------
Zebrafish (ENSDARP00000101295)       -----------------------------------------METEKTSNETEKSH--ILAKIS---ATQQTDFLTKHQNGDQSPEVL---
Salmon (XP_014042431.1)              ------------------------------------------------------------------------------------------
Salmon (XP_014065146.1)              -----------------------------------------METEKASQETEESL--IVAQLSA-ATQPDNLITAKLQNGTQSPEGA---
Salmon (XP_014050290.1)              -----------------------------------------METEKASQETEESL--IVAQLSA-ATQPDNLITAKLQNGTQSPEGA---
Rainbow trout (XP_021429026.1)       ------------------------------------------------------------------------------------------
Rainbow trout (XP_021429023.1)       -----------------------------------------METEKASQETEESL--ILAQLSA-ATQPDNLITAKLQNGTQSPEGA---
Shark (XP_007910136.1)               ------------------------------------------------------------------------------------------
Human (ENSP00000386869)              ----------------------------MSQFQVPLAVQPDLPGLYDFPQRQVMVGSFPGSGLSMAGSESQLRGGGDGRKKRKRCGTCEP
Gorilla (ENSGGOP00000051172)         ----------------------------MSQFQVPLAVQPDLPGLYDFPQGQVMVGGFPGSGLSMAGSESQLRGGGDGRKKRKRCGTCEP
Armadillo (ENSDNOP00000021516)       ----------------------------MSQFQVPLAVQPDLPGLYDFPQGQVMVGGYQGPVLPVAGSEPQLRGGGDGRKKRKRCGACEP
Mouse (ENSMUSP00000087049)           ------------------------------------------------------------------------------------------
Rat (ENSRNOP00000015296)Tet3         ----------------------------MSQFQVPLAVQPDLSGLYDFPQGQVMVGGFQGPGLPMAGSETQLRGGGDGRKKRKRCGTCDP
Pig (ENSSSCP00000008852)             ------------------------------------------------------------------------------------------
Elephant  (XP_010591957.2)           ----------------------------MSQFQVPLAVQPDLPGLYDFPQGQVMVGGFQGPGLPMTGSEPQLRGGGDGRKKRKRCGTCEP
Whale (XP_023989264.1)               ----------------------------MSQFQVPLAVQPDLPGLYDFPQGQVMVGGFQGPGLPMTGSEPQLRGGGDGRKKRKRCGTCEP
Tasmanian_devil (ENSSHAP000000       ------------------------------------------------------------------------------------------
Chicken (ENSGALT00000059756)         ------------------------------------------------------------------------------------------
Lizard (ENSACAT00000001585)          ------------------------------------------------------------------------------------------
Turtle (ENSPSIT00000006549)          ------------------------------------------------------------------------------------------
Xenopus (ENSXETP00000054061)         -----------------------------MDTQPAPVPHVLPQDVYEFPDDQESLGRLRVSEMPAELNGWNVYGSRTAHIIHTTECSVSP
Coelacanth (ENSLACP00000009535       ------------------------------------------------------------------------------------------
Spotted_gar (ENSLOCP0000001981       ------------------------------------------------------------------------------------------
Amazon_molly (ENSPFOP000000021       ------------------------------------------------------------------------------------------
Cave_fish (ENSAMXP00000015689)       ------------------------------------------------------------------------------------------
Fugu (ENSTRUP00000036865)            -------------------------------------------------------------------------------KRRKKCGACAP
Medaka (ENSORLP00000019236)          ------------------------------------------------------------------------------------------
Platyfish (ENSXMAT00000000130)       ------------------------------------------------------------------------------------------
Stickleback (ENSGACP0000001732       ------------------------------------------------------------------------------------------
Tetraodon (ENSTNIP00000019883)       ------------------------------------------------------------------------------------------
Tilapia (ENSONIP00000019943)         ------------------------------------------------------------------------------------------
Zebrafish (ENSDARP00000085229)       ------------------------------------------------------------------------------------------
```

```
Rainbow_trout (XP_021462005.1)  ------------------------------------------------------------------------------------------
Rainbow_trout (XP_021477599.1)  MHRAKGGPGPPHFQDIYEFSLDGDETTTQIFLRGPGKAMEGPGMPLSPHYKYIQTEHQQGGLFSLGPPTHGEALAAPQGKKRRRCGVCGP
Salmon (XP_014026412.1)         MHRAKGGPGPPPFQDIYEFSMDGDETGTQIFLRGPRTAMEGPGEPLSPHSEYIQTEHQQGGLFSLRPPAHGVPHTAPQGKKRRRCGVCAP
Salmon (XP_014017100.1)         MHRAKGGPGPPHIQDIYEFSLDGDETATQIFLRGPGKAMEGPGLPLSPHHKYIQTEHQQGVIFSLGPPTHGEALAAPQGKKRRRCGVCGP

                                        100       110       120       130       140       150       160       170       180
                                ....|....|....|....|....|....|....|....|....|....|....|....|....|....|....|....|....|....|
European ampioxus (Sc0000095)   ------------------------------------------------------------------------------------------
Ciona intestinalis (XP_0021254  ------------------------------------------------------------------------------------------
Lamprey (scaf_00044)            ------------------------------------------------------------------------------------------
Lamprey (scaf_03335)            ------------------------------------------------------------------------------------------
Shark (XP_007897316.1)          ------------------------------------------------------------------------------------------
Lizard (XP_020663651.1)         CKRLLEEKKNGKKKPQEQNSQLTSSCRFLRSSVTKSLSGTPWMNLELSDNVLFHNQPHLNGSGFTMTLRSKPFSHRRFQTATITRTKKAV
Chicken (ENSGALP00000053457)    GKKPAQEK-DVKKKQQEKKPIMSSSGRFLRSSVTRTLSGTPWVSLEKADNILFHSQEAFNINGFTMSLRNRSFSRRFSQAPTIAKPRKAA
Turtle (ENSPSIP00000006704)     ------------------------------------------------------------------------------------------
Human (ENSP00000362748)         LKQLIQERDVKKKTEPK-------PPVPVRSLLTR--AGAARMNLDRTE-VLFQNPESLTCNGFTMALRSTSLSRRLSQPPLVVAKSKKV
Gorilla (ENSGGOP00000011536)    LKQLIQERDVKKKTEPK-------PPMPVRSLLTR--AGAARMNLDRTE-VLFQNPESLTCNGFTMALRSTSLSRRLSQPPLVVAKSKKV
Mouse (ENSMUSP00000133279)      PKQLIKRRDGKKETEDK-------TPTPAPSFLTR--AGAARMNRDRNQ-VLFQNPDSLTCNGFTMALRRTSLSWRLSQRPVVTPKPKKV
Rat (ENSRNOP00000054747)        LKQLIRRRDGKKETEVK-------TPTLPQSLLTR--AGAARLNQDRNQ-VLFQNPESLTCNGFTMALRRTSLSWRLSQRPVVTPKPKKV
Pig (ENSSSCP00000010918)        LKQLIQERDVKKKTELK-------PPLPVRSLLTR--AGAARMNLDRTE-VLFQNPESLTCNGFTMALRSTSLSRRLSQPPVVIAKPKKV
Whale (XP_023987950.1)          LKQLIQERGVKKKTEPK-------LPMPVRSLLTR--AGAARMNLDRTE-VLFQNPESLTCNGFTMALRSTSLSRRLSQPPAVIAKPKKV
Elephant (ENSLAFP00000005179)   LKQLIQERDVKKKTEAK-------PPMPVRSLLTR--AAAARMNLERTE-VLFQNPESLTCNGFTMALRSTSLSRRLSQPPVVIAKPKKV
Armadillo (ENSDNOP00000014565)  LKPLIQERDVKKKPEPK-------PPMPVRSLLTR--AGAARMNLDRTE-VLFQNPESLTCNGFTMALRSTSLSRRLSQPPVVIAKTKKV
Tasmanian_devil (XP_023360087.  ------------------------------------------------------------------------------------------
Coelacanth (ENSLACP00000016889  ------------------------------------------------------------------------------------------
Cavefish(XP_007240334.2)        KLRASTDKKRVV---------------NSNKPKGPATRTRQAKSRKSTAK-KQATKKLQAGAADSKSAVKAS------------------
Platyfish (XP_014325204.1)      NQAKSGRSPCG---------SGKDLRGSPGRGNHA-GNVTGSTRLRRSSSVP------ENPLGPRKSQRGT-------QVSVAPCQSPKA
Tilapia (XP_005456583.2)        AQAQSGQSPHGREV---SRPAAQGPRRSPSRVNHS-LNVTGSARLRRASNPPDHCGKLQDPLGRRKSQRGT-------QVSVTPCQPPKP
Spotted_gar (XP_015202470.1)    RQQTAKELRSAKVS-----KSGSGGSRGSQNSIKNAFRVIGSMKLRQQGNSLPQQALSEDSPNRRKSLRGIP------------------
Stickleback (ENSGACT0000001035  ------------------------------------------------------------------------------------------
Tetraodon (ENSTNIT00000011701)  ------------------------------------------------------------------------------------------
Fugu (XP_011601624.1)           AQTKLAESPSRR-G---RRGAGQGPSQALGTANPA-LNVTGSMRLRWPSNLPERCGELKDLPGRGKSLQGA-------RVSISTTKSCKG
Zebrafish (ENSDARP00000143362)  AMPRTAKAPKKS---------------TRASTKRNVKRVKASMTRRQTINSLKASGEIKKGIDKKQAAKRAQ------------------
Amazon_molly (XP_016534180.1)   NQAKSGWRPRG---------SGKNPGRSPGRGSHA-GNVTGSTRLRRSSSVP------ENPLRPRKSQRGT-------QVSVAPCQSPKA
Medaka (XP_023818916.1)         KLTKRTQTLGRTLR---RRGSDDVCPNQDLRKSQA-INATVNTRLRRSSGLP--ETKLQAPSGRRRSLRET-------HLSVTPSLKQK-
Rainbow_trout (XP_021469805.1)  TQAQPARSPGRSGVGRLG---SQVPGGSPGRVNHAPVHVTGSVRLRSRGNPLLQHGVGEDPPGRRMSLRGMGCSKKFPPLTVKPSRGGKK
Rainbow trout (XP_021436521.1)  TQAQPARSPGRSGVGRLG---GQGPGASPGRVNHAPVGITGSVRLRSWGNPLLQHGVGEDPPGRRISLRGMGGAKQFPPLAIKPRTGGKT
Salmon (XP_014010485)           TQAQPARSPGRSGVGRLGTLGSQVPGGSPGRVNHAPVHVTGSVRLRSRGNPLLQHGVGEDPPGRRMSLRGMGCSKKFPPLTVKPCRGGKK
Salmon (XP_014070638.1)         TQAQPARSPGRSGVGRLG---GQGPGASPGRVNHAPVGITGSVRLRSWGNPLLQHGVGEDPPGRRISLRGMGGAKQFPPLAIKPRTGGKT
Shark (XP_007890120.1)          QQQVNGDFKRGTVKSNYVD----SHAQGNEEKFHNPEAKQEKTEFPKCLQ-----NGAIKHVLSESSSCGLQQPKKIKLDEESNGQDPDL
Human (ENSP00000425443)         HPEVNGDTKWHSFKSYYG----IPCMKGSQNSRVSPDFTQESRGYSKCLQ-----NGGIKRTVSEPSLSGLLQIKKLKQDQKANGERRNF
Gorilla (ENSGGOP00000034525)    HPEVNGDTKWQSFKSYYG----IPCMKGSQNSRVSPDFTQESRGYSKCLQ-----NGGIKRTVSEPSLSGLLQIKKLKQDQKANGERRNF
Mouse (ENSMUSP00000143029)      HPEVNGDTKWQSSQSCYG----ISHMKGSQSSHESP---HEDRGYSRCLQ-----NGGIKRTVSEPSLSGLHPNKILKLDQKAKGESNIF
Rat (ENSRNOP00000048956)        HPEVNGDTKWQSFQSYYG----IPHMKGSQSSHESPDFIHEGRGYSGCLQ-----NGGIKRTVSEPSLSGLHLNTKLTLDQKAKGESNNF
Pig (ENSSSCP00000009765)        YPEVNGDTKWQSFKSYSG----IPHMKGNQHSRVSPDFIQEGRGYSKCLQ-----NGGIKRTVSEPSLSGLHQNKKLKQDPKANGERTNF
Tasmanian_devil (ENSSHAT000000  HPEVNCENRWLSFKSSYG----MPPAKESQNNCVSPDFMHEDREYPKCMQ-----NGGIKRTSSEPSLSGIHQNKKVKQDQDINGERHDF
Whale (XP_007106564.2)          YPEVNGDTKWQSFKSYYG----IPHMKGSQNSRVSPDFIQESRGYSRCLQ-----NGGIKRTVSEPSLSGLHQNKKLKQDQKANGERKNF
Elephant (XP_023404313.1)       HSEVNGDTKWQSFRSYYG----IPSMKGSQNSRISPDFIQEGRGYSKCLQ-----NGGIKRTVSEPSLSGLHQNKKLKQDQTANGERKNF
Lizard (ENSACAP00000008438)     ------------------------------------------------------------------------------------------
Chicken (ENSGALP00000017216)    EVEVNGNHKRLFNKSNYR----EPHAKGSPNHRISPDLLQEKKACSIYMQ-----NGGIKRTFSEPSLFGLQQSKKVKQDKEVNGEKAEP
Turtle (ENSPSIP00000011691)     HAEVNGDHKWLLLKNNYG----IPHVKGNPNNRTSPDLLQEKRVYSKYMQ-----NGGIKRTFSEPSLYGLHQNKKIKQDEEVNGEQDEP
Xenopus (ENSXETP00000030770)    DLLVNGDICLPT-KNNYN----VAYMKGSQE-HGASALIHDNKAYPNHIQ-----NGGIKRTYSEPLFS-ESQLKKVRTD-EINGENKGD
Coelacanth (ENSLACP00000008576  QAQVNGDIKWFHFKGSYG----INHVKGNQENHASPDPVQNNIDYPKYIQ-----NGGIKRTSSESSLLGLQQSKKNKQNEELNGLDSEL
Amazon_molly (ENSPFOP000000120  SVQISDSESWITGVVHYEPSKAAGSMKRYRENCNIPTSEQALFQPGFFKMNGDLMNGELKHGFAEQ-SLAVHQSKKIKVDGEMKENGDS-
Amazon_molly (ENSPFOP000000299  SVQISDSESWITGVVHYEPSKAAGSMKRYRENCNIPTSEQALFQPGFFKMNGDLMNGELKHGFAEQ-SLAVHQSKKIKVDGEMKENGDS-
Fugu (XP_011611191.1)           SLQISGEGNWN----HYKPGTGANSMKRQRENSNGPASVEGLFDQASYMIKGELINGELKHALDDQ-AIRVHQHKKIKIN---SENDNV-
Medaka (XP_004065962.1)         SPQPTGDNNWITSPVQYNPNKVSNSLKRHRENCIIPASEESLFDQESYKMNGDLMNGELKHGFTG--DTLLHQPKRKKVDLEIKRNDGT-
Platyfish (ENSXMAP00000005447)  SVQISDSESWITGAVHYEPSKAAGSMKRYRENCNIPASEQALFQSGFFKMNGDLMNGELKHGFAEQ-SLAVHQTKKIKVDGDMKENGDS-
Spotted _gar (ENSLOCP000000155  PQQTNGDSNWN----HFKPNMGINQMKMYQENCSSPPVKQELLEQSQHIK-----NGGIKHAFSESSLLELCQSKKPRMDSEINGQGSQI
Stickleback (ENSGACP0000002318  ------------------------------------------------------------------------------------------
Tilapia (XP_005457058.1)        SLQITGDKNWN----HYKPSTDANTLKRHRENCNNPASEQGLLDQGSYIMNGELMNGELKHALTEQ-SVLVHQSKKTKIDSEIKGKDDI-
Tetraodon (ENSTNIP00000021201)  ------------------------------------------------------------------------------------------
Zebrafish (ENSDARP00000101295)  SQQK------------------------------VPANLQGIFDQSMFGMK-----GQMKHALSEQALLDFHQTKTLVNGNENPRIWDK-
Salmon (XP_014042431.1)         ------------------------------------------------------------------------------------------
Salmon (XP_014065146.1)         HQQINRDGNWN--LYNKAVAGVVNPMKRHRENCSSPARVQGLFNQGSYNM---INGGDLKHALCDQSFLGLHQAKKLRSDLEINGGDGEE
Salmon (XP_014050290.1)         HQQINRDGNWN--LYNKAVAGVVNPMKRHRENCSSPARVQGLFNQGSYNM---INGGDLKHALCDQSFLGLHQAKKLRSDLEINGGDGEE
Rainbow trout (XP_021429026.1)  ------------------------------------------------------------------------------------------
Rainbow trout (XP_021429023.1)  HQQINGDGNWN--LYNK--AGVINPMKRHRENCSSPAGVQGLFDQGSYNM---INGGDLKHALCDQSLLGLHQAKKLRSDLEINGGDGEE
Shark (XP_007910136.1)          ------------------------------------------------------------------------------------------
Human (ENSP00000386869)         CRRLENCGACTSCTN---------------------RRTHQICKLRKCEVLKKKVGLLKEVEIKAGEGAGPWGQGAAVKTGSELSPVDGP
Gorilla (ENSGGOP00000051172)    CRRLENCGACTSCTN---------------------RRTHQICKLRKCEVLKKKVGLLKEVEIKAGEGAGPWGQGAAVKTGSELSPVDGP
Armadillo (ENSDNOP00000021516)  CRRLENCGACTSCTN---------------------RRTHQICKLRKCEVLKKKAGLLKEVEIKAGEGGGPWGAGAAVKTCSELSPVNGP
Mouse (ENSMUSP00000087049)      ------------------------------------------------------------------------------------------
Rat (ENSRNOP00000015296)Tet3    CRRLENCGSCTSCTN---------------------RRTHQICKLRKCEVLKKKAGLLKEVEINAREGTGPWAQGATVKTGSELSPVDGP
Pig (ENSSSCP00000008852)        ------------------------------------------------------------------------------------------
Elephant  (XP_010591957.2)      CRRLENCGACTSCTN---------------------RRTHQICKLRKCEVLKKKAGLLKEVEIKAGEGAGPWGQRVAVKTGSELSPVDGP
Whale (XP_023989264.1)          CRRLENCGACTSCTN---------------------RRTHQICKLRKCEVLKKKVGLLKEVEIKAGEGAGPWGQGAAVKTGSELSPVDGP
Tasmanian_devil (ENSSHAP000000  ------------------------------------------------------------------------------------------
Chicken (ENSGALT00000059756)    -----------------------------------------------------------------------------------------M
Lizard (ENSACAT00000001585)     ---------------------------------------------------------------------------------MEEGPFHQA
Turtle (ENSPSIT00000006549)     -----------------------------------------------------------------------------------------M
Xenopus (ENSXETP00000054061)    RSTCSPCCPCFRDSL---------------------CMYVYVCMSRQQCHLNCRC-----TKFKLIKSREIITPKGILQNCSESILVDGP
Coelacanth (ENSLACP00000009535  -------------------------------------------------------------------------------TGSGGNSFDGP
Spotted_gar (ENSLOCP0000001981  -------GPNSFSFSLQTG-------------------------------------------SRVGSVDGPRRG-QMEVGSSDHGEENGL
Amazon_molly (ENSPFOP000000021  ------------------------------------------------------------------------------------------
Cave_fish (ENSAMXP00000015689)  ------------------------------------------------------------------------------------------
Fugu (ENSTRUP00000036865)       CLRRENCGSCANCLNRRTGKQICKLRKCEQLKKRQHE------------------WEEVETVQKVISVDGPRSGGQMEIGSHDRLQEGAL
Medaka (ENSORLP00000019236)     ------------------------------------------------------------------------------------------
```

```
Platyfish (ENSXMAT00000000130)      ------------------------------------------------------------------------------------------
Stickleback (ENSGACP0000001732      ------------------------------------------------------------------------------------------
Tetraodon (ENSTNIP00000019883)      ------------------------------------------------------------------------------------------
Tilapia (ENSONIP00000019943)        ------------------------------------------------------------------------------------------
Zebrafish (ENSDARP00000085229)      ------------------------------------------------------------------------------------------
Rainbow_trout (XP_021462005.1)      ----------------------------------------------------------------------------METGSHDRLEEGCS
Rainbow_trout (XP_021477599.1)      CMQKENCGTCSNCMNRKIGKQICKLRKCDQLKMKGQKDWET--------------------VSDARSVDGPRRGGQMETGSHDRLEEGCL
Salmon (XP_014026412.1)             CMQKENCGTCSNCMNRKIGKQICKLRKCDQLKKKGQKDCEVGHIEDYSKAWSVKPSLATETVSDTGSVDGPRRGGQMETGSHDRLEEGGS
Salmon (XP_014017100.1)             CMQKENCGTCSNCMNRKIGKQICKLRKCEQLKMKGQKDWEVGHIEDNSKAWFVKASLATETVSDARSVDGPRRGGQMETGSHDRIEEGGL

                                             190       200       210       220       230       240       250       260       270
                                    ....|....|....|....|....|....|....|....|....|....|....|....|....|....|....|....|....|....|
European ampioxus (Sc0000095)       ------------------------------------------------------------------------------------------
Ciona intestinalis (XP_0021254      ------------------------------------------------------------------------------------------
Lamprey (scaf_00044)                ------------------------------------------------------------------------------------------
Lamprey (scaf_03335)                ------------------------------------------------------------------------------------------
Shark (XP_007897316.1)              ------------------------------------------------------------------------------------------
Lizard (XP_020663651.1)             LGRREKSGTGTPHVPDRKEIEEIAQASQQFSNQKGESPSSKEDHTVCNKE----------------------------------------
Chicken (ENSGALP00000053457)        AQKKLET----------KEKHEQALAVDIPCSVAEEPAAT--------------------------------------------------
Turtle (ENSPSIP00000006704)         ------------------------------------------------------------------------------------------
Human (ENSP00000362748)             PLSKGLEKQHDCDYKILPALGVKHSENDS--VPMQDTQVLPDIETLIGVQ----------------------------------------
Gorilla (ENSGGOP00000011536)        PLSKGLEKQHDCDYKILPDLGVKHSENDS--VPMQDTQVLPDIETLIGVQ----------------------------------------
Mouse (ENSMUSP00000133279)          PPSK-----KQCTHNIQDEPGVKHSENDS--VPSQHATVSPGTE--NGEQ----------------------------------------
Rat (ENSRNOP00000054747)            PPSK-----KQCVHNVQDDPGVKHSGNDS--GPSQDAVMSPDTENLTGEQ----------------------------------------
Pig (ENSSSCP00000010918)            PPPKNLGKQHECDYKVLTDMGVKHSENDSKQVPKQDPPIPPDAENLIGKQ----------------------------------------
Whale (XP_023987950.1)              PPPKNLGKQHECDYKVLTDVGVKHSESDS--VPVQDPSIPPDIENLIGIQ----------------------------------------
Elephant (ENSLAFP00000005179)       PPSKNLEKQRECDCKVLTDVGVRHSENQS--DLVQDPPIPPDTENLIAVQ----------------------------------------
Armadillo (ENSDNOP00000014565)      PPSKNLQKQLECDYKVLTDVGVKTTENES--HPMQDPLIPLDIENLIGIQ----------------------------------------
Tasmanian_devil (XP_023360087.      ------------------------------------------------------------------------------------------
Coelacanth (ENSLACP00000016889      ------------------------------------------------------------------------------------------
Cavefish(XP_007240334.2)            --------------QERASR-SKNTSNSTESVRIS-------------------------------------------------------
Platyfish (XP_014325204.1)          FTGG--KNSRRGSSGRRAASRQQNTSNKPSSISINNSPPDGKNEKPDNK---EPPRMYHEAGVEAAASLEP-----NDATEGSSL-----
Tilapia (XP_005456583.2)            CSRG--KYSR-GSTRRRSAPQHGNKRNQRPSTRINDSTPDEEAEKLENC---DDEEVGPKTEVETPENVKDQGLGVNGAKEDSVF-----
Spotted_gar (XP_015202470.1)        ------------LSQSLPQAPQPNRNTQTS------------------------------------------------------------
Stickleback (ENSGACT0000001035      ------------------------------------------------------------------------------------------
Tetraodon (ENSTNIT00000011701)      ------------------------------------------------------------------------------------------
Fugu (XP_011601624.1)               -------RTGRGGAKRAAPPRRVKSRNQPSSAAINDSPPEEEDETPYRRGAEEKIPVSLKTDFEVSVSIKDQGLGVPNAQEGSPL-----
Zebrafish (ENSDARP00000143362)      --------------RTVVSKPNKKTVEDVLPVKIS-------------------------------------------------------
Amazon_molly (XP_016534180.1)       FTGG--KNSRRGSSQRRAASRQQNTSNKPPSISINNSPPDGKNEKPDNK---EPPRMHHEAGVEAAASLEP-----NDAIEGSSL-----
Medaka (XP_023818916.1)             -------RGRKTSSESGAAPQHQNTKRQTSSTSINESSFDEEKAKDSHT---NEELSLLCAEANVPAGIKDQSLGVGDTKESGAS-----
Rainbow_trout (XP_021469805.1)      ARGRGARGAR---GQRWSERGIEQGPEESDPGSIDPSMIDQEKEVVSHDS--EGQPLNLRTELEDPSCVDQQEPGVGHECEGKPLNLRTE
Rainbow trout (XP_021436521.1)      TRGRGVRGVRGVVGQRGSKSGIEQGSEESDTVSIDPSEINQGNNVVGHVG--KGKPPNLRTELDETTCVDQQELGVGHASEGKPR-----
Salmon (XP_014010485)               ARGRGARGAR---GQRWSERGIEQGPEESDPGSIDPSVIDQEKELVSHDS--EGQPLNLRTELEDPSCVDQQEPGVGHECEGKPLNLRTE
Salmon (XP_014070638.1)             ARGRGVRGGRGAVGQRGSKSGIEKGSEESDTVSVDPSEINQGNKVFGHVG--KGKLPNLRTELDETTCVDQQELGVGHASEGKPR-----
Shark (XP_007890120.1)              EEDQL------------------------WDFEGEKWSGAVKAPGYDCED----------------------------------------
Human (ENSP00000425443)             GVSQE-RNPGE-SSQPNVSDLSDKKESVSSVAQENAVKDFTSFSTHNCSG----------------------------------------
Gorilla (ENSGGOP00000034525)        GVSQG-RNPGE-SSQPNVSDLSDKKESVSSVAQENAVKDFTSFSTHNCSG----------------------------------------
Mouse (ENSMUSP00000143029)          EESQE-RNHGKSSRQPNVSGLSDNGEPVTSTTQESSGADAFPTRNYN-------------------------------------------
Rat (ENSRNOP00000048956)            GESQE-GNPGKSSSHPNVSGLSDDREPGTSTAQESTDIDAFPTRNYN-------------------------------------------
Pig (ENSSSCP00000009765)            GESQE-RNPGKGSSQPNVSDLSGKRESESFAAQENEVKDFTSFSTHNCIG----------------------------------------
Tasmanian_devil (ENSSHAT000000      RGNQEGNNQGENSNQPNVSNSCN-KVPLNFTVQEELISNFPCSARHNFSE----------------------------------------
Whale (XP_007106564.2)              GESQE-RNPGKGSSQPNVSNVSDKRESVSSAAQENEVKDFTGFSSHNCSG----------------------------------------
Elephant (XP_023404313.1)           GESQE-RNPGESSSQPNVSNLSDKREPES-IAQENAVKDLASFSTHNCSG----------------------------------------
Lizard (ENSACAP00000008438)         ------------------------------------------------------------------------------------------
Chicken (ENSGALP00000017216)        EDNYE---------TPSISNCYSEKKSE--MGQENEALELMPSTRYNSGG----------------------------------------
Turtle (ENSPSIP00000011691)         EEKNG---------KTTVSNSCSEKKSVSFTGQENAASDVTQSSRGNCDG----------------------------------------
Xenopus (ENSXETP00000030770)         QRQNN--------IQASVSEFCNEDQSVRIFQPVNSP-----------------------------------------------------
Coelacanth (ENSLACP00000008576      LEKNN---------ELHLPSLCNKKESVALEEEETVLSDLLKPSSFNFDS----------------------------------------
Amazon_molly (ENSPFOP000000120      -----------------------------SRSFVKNFSELTKKTEM--------------------------------------------
Amazon_molly (ENSPFOP000000299      -----------------------------SRSFVKNFSELTKKTEM--------------------------------------------
Fugu (XP_011611191.1)               -----------------------------SSNLADAFPDF-KATEF--------------------------------------------
Medaka (XP_004065962.1)             -----------------------------SSEMVDNLPELTNATNF--------------------------------------------
Platyfish (ENSXMAP00000005447)      -----------------------------SRNFVKNLSELTKKTEM--------------------------------------------
Spotted _gar (ENSLOCP000000155      AKNNV-------------ISSWSKNESAEMERKLGSFPEPLKPSECRDVP----------------------------------------
Stickleback (ENSGACP0000002318      ------------------------------------------------------------------------------------------
Tilapia (XP_005457058.1)            -----------------------------SSDLLDKFPELTTTNEF--------------------------------------------
Tetraodon (ENSTNIP00000021201)      ------------------------------------------------------------------------------------------
Zebrafish (ENSDARP00000101295)      ----------------------------------SEAGMENRLEVF--------------------------------------------
Salmon (XP_014042431.1)             ------------------------------------------------------------------------------------------
Salmon (XP_014065146.1)             MW-----------------------DKKDSGDIMDDFPELTKPGEF--------------------------------------------
Salmon (XP_014050290.1)             MW-----------------------DKKDGGDIMDDFPELTKPGEF--------------------------------------------
Rainbow trout (XP_021429026.1)      ------------------------------------------------------------------------------------------
Rainbow trout (XP_021429023.1)      MW-----------------------DKKDGGDIMDDFPELTKPGEF--------------------------------------------
Shark (XP_007910136.1)              ------------------------------------------------------------------------------------------
Human (ENSP00000386869)             VPGQMDSGPVYHGDSRQLSA--SGVPVNGAREPAGPSLLGTGGPWRVD------------------------------------------
Gorilla (ENSGGOP00000051172)        VPGQMDSGPVYHGDSRQLSA--SGVPVNGAREPAGPSLLGTGGPWRVD------------------------------------------
Armadillo (ENSDNOP00000021516)      VPGPMDSGPVYHGASRQLSA--SGAPVNGAREPAGPGLLGAGGPWRAD------------------------------------------
Mouse (ENSMUSP00000087049)          ----MDSGPVYHGDSRQLST--SGAPVNGAREPAGPGLLGAAGPWRVD------------------------------------------
Rat (ENSRNOP00000015296)Tet3        VPGQMDSGPVYHGDSRQLST--SGAPVNGAREPAGPSLLGAAGPWRVD------------------------------------------
Pig (ENSSSCP00000008852)            ----MDSGPVYHGDSRQLSA--PGAPVNGAREPAGPSLLGTGGPWRAD------------------------------------------
Elephant  (XP_010591957.2)          VPGQMDSGPVFHGDSRQLSS--SGVPVNGAREPAGPSLLGAGGPWRVD------------------------------------------
Whale (XP_023989264.1)              VPGQMDSGPVYDGDSRQLSA--SGAPVNGAREPAGPGLLGTGGPWRAE------------------------------------------
Tasmanian_devil (ENSSHAP000000      ----MDPGPVYHGEERRLKT--PRAPVNGAGEPAGPGLLGVGGVWQVN------------------------------------------
Chicken (ENSGALT00000059756)        LTPLLSPRSQTGPGGDPVDGPRRGPMEEGLIDYGGERTLSEGG-RLG-------------------------------------------
Lizard (ENSACAT00000001585)         EERRLSGGLTNGGGGGRMDG-RGGGREGG--GGGGSAMLSDGGSWLGGLC----------------------------------------
Turtle (ENSPSIT00000006549)         LNSDPPASLVSDP-------------------------MAELEQLLG-------------------------------------------
Xenopus (ENSXETP00000054061)        KTDQMEAGPVNHVQEGRLKQECDSTLPSKGCEDLANQLLMEANSWLSNTA----------------------------------------
Coelacanth (ENSLACP00000009535      KTGQMEEGPINHVEESRLRLESEPSVVNGARERDETSALMEESDWLE-------------------------------------------
```

```
Spotted_gar (ENSLOCP0000001981 N-------------------------------LGLVNGASRQPSEEEEGR----------------------------------------
Amazon_molly (ENSPFOP000000021 ------------------------------------------------------------------------------------------
Cave_fish (ENSAMXP00000015689) ----------------------------------------------IGAS----------------------------------------
Fugu (ENSTRUP00000036865)      SLKVAN--------------GVNNYPSGDGTSIETEQQRTGSVPPRPWVS----------------------------------------
Medaka (ENSORLP00000019236)    ------------------------------------------------------------------------------------------
Platyfish (ENSXMAT00000000130) ------------------------------------------------------------------------------------------
Stickleback (ENSGACP0000001732 ------------------------------------------------------------------------------------------
Tetraodon (ENSTNIP00000019883) ------------------------------------------------------------------------------------------
Tilapia (ENSONIP00000019943)   ------------------------------------------------------------------------------------------
Zebrafish (ENSDARP00000085229) ------------------------------------------------------------------------------------------
Rainbow_trout (XP_021462005.1) LSLEPTNGVIRQPSDTNEEGGAGLDTQREPHRSGSVSPGQGWVPGQGKAP----------------------------------------
Rainbow_trout (XP_021477599.1) LSLEPTNGVSRHPRDTHEEGGAGLEPQREPQRSSSVSPGQGCVPGHSKAT----------------------------------------
Salmon (XP_014026412.1)        LSLEPTNGVSRQPSDTHEEGGAGLDTQREPHRSGSVPPGQGWVPGQGKAP----------------------------------------
Salmon (XP_014017100.1)        LSLEPINGVSRHPRDTHEEGGAGLGPQREPQRSRSVSPGQGCVPGHFKAT----------------------------------------

                                        280       290       300       310       320       330       340       350       360
                               ....|....|....|....|....|....|....|....|....|....|....|....|....|....|....|....|....|....|
European ampioxus (Sc0000095)  ------------------------------------------------------------------------------------------
Ciona intestinalis (XP_0021254 ------------------------------------------------------------------------------------------
Lamprey (scaf_00044)           ------------------------------------------------------------------------------------------
Lamprey (scaf_03335)           ------------------------------------------------------------------------------------------
Shark (XP_007897316.1)         ------------------------------------------------------------------------------------------
Lizard (XP_020663651.1)        ----------------------------------------------------IVNPSECDRQAEKGESSP-------EKSISPSISDIPN
Chicken (ENSGALP00000053457)   ----------------------------------------------------------CAGQEEEGEIPE-------TKNSVPST-----
Turtle (ENSPSIP00000006704)    ------------------------------------------------------------------------------------------
Human (ENSP00000362748)        ----------------------------------------------------NPSLLK-GKSQETTQFWS---QRVEDSKINIPTHSGPA
Gorilla (ENSGGOP00000011536)   ----------------------------------------------------NPSLLK-GKSQETTQFWS---QRVEDSKINIPTHSGPA
Mouse (ENSMUSP00000133279)     ----------------------------------------------------NRCLVE-GESQEITQSCPVFEERIEDTQSCISASGNLE
Rat (ENSRNOP00000054747)       ----------------------------------------------------NMCLVE-GESQEITQSC---SQTVEDSQSCISEYGNLE
Pig (ENSSSCP00000010918)       ----------------------------------------------------NTSLIK-DEGQETTQSWP---QSVEESEVNHSTHSGPV
Whale (XP_023987950.1)         ----------------------------------------------------NASLIK-DESQETTQSWS---QSVENSKINRSIHSGPA
Elephant (ENSLAFP00000005179)  ----------------------------------------------------NSSLIIKGESQKTTQSWS---QRIEDAKINIPTHSDPA
Armadillo (ENSDNOP00000014565) ----------------------------------------------------NVSLIK-GEGQETTQSWS---QRVEDSRINIPTHHGPV
Tasmanian_devil (XP_023360087. ------------------------------------------------------------------------------------------
Coelacanth (ENSLACP00000016889 ------------------------------------------------------------------------------------------
Cavefish(XP_007240334.2)       -------------------------------------------------------------------RHTCNSLSQHDPAADLRSRTRSF
Platyfish (XP_014325204.1)     ----------------------------------------------------ETDTRGGD------VQHSCDGLITEDE-TSFSNNSEGD
Tilapia (XP_005456583.2)       ----------------------------------------------------KADSPLCNNTLEDCVQNSCDGPITQQEKITDSHKSIGD
Spotted_gar (XP_015202470.1)   -----------------------------------------------------------------------NSRCRLPARPSPEHRSTGA
Stickleback (ENSGACT0000001035 ------------------------------------------------------------------------------------------
Tetraodon (ENSTNIT00000011701) ------------------------------------------------------------------------------------------
Fugu (XP_011601624.1)          ----------------------------------------------------KADSPPCADTLDDCGQCSHDSATSQRDKNAVSRDASGD
Zebrafish (ENSDARP00000143362) -------------------------------------------------------------------RHTTSSLNQNGLLLECNGKGKSP
Amazon_molly (XP_016534180.1)  ----------------------------------------------------ETDTRRGD------LQHSCDSLITEDE-KSFSNNSEGD
Medaka (XP_023818916.1)        ----------------------------------------------------KVPSPEGTDMLDDSLPLGCDRHAAHQDQKTVISDGDSD
Rainbow_trout (XP_021469805.1) LDDTSCVDQQEPGVSHDSEGKPNQRTELDDHSCIDQQEPGVGHDGEGKPLNLRTDDSASDDKHEELVAHNGDAVGTDPHDKPFSHSGEGK
Rainbow trout (XP_021436521.1) --------------------------------------------------SLRTDDLASDDKQEELVAHNGETVGTDSYDKPVSHSGEGN
Salmon (XP_014010485)          LDDP---------------------------SCIDQLEPGVGHDGEGKPLNLRTDDSASDGKHEELVAHNGDVVGTDPHNKPFSHSGDGN
Salmon (XP_014070638.1)        --------------------------------------------------NLRTDDLASDDKQEELVAHNGETVGADSHDKTVSHSGEGN
Shark (XP_007890120.1)         ------------------------------------------------------------------------------------------
Human (ENSP00000425443)        ------------------------------------------------------------------------------------------
Gorilla (ENSGGOP00000034525)   ------------------------------------------------------------------------------------------
Mouse (ENSMUSP00000143029)     ------------------------------------------------------------------------------------------
Rat (ENSRNOP00000048956)       ------------------------------------------------------------------------------------------
Pig (ENSSSCP00000009765)       ------------------------------------------------------------------------------------------
Tasmanian_devil (ENSSHAT000000 ------------------------------------------------------------------------------------------
Whale (XP_007106564.2)         ------------------------------------------------------------------------------------------
Elephant (XP_023404313.1)      ------------------------------------------------------------------------------------------
Lizard (ENSACAP00000008438)    ------------------------------------------------------------------------------------------
Chicken (ENSGALP00000017216)   ------------------------------------------------------------------------------------------
Turtle (ENSPSIP00000011691)    ------------------------------------------------------------------------------------------
Xenopus (ENSXETP00000030770)   ------------------------------------------------------------------------------------------
Coelacanth (ENSLACP00000008576 ------------------------------------------------------------------------------------------
Amazon_molly (ENSPFOP000000120 ------------------------------------------------------------------------------------------
Amazon_molly (ENSPFOP000000299 ------------------------------------------------------------------------------------------
Fugu (XP_011611191.1)          ------------------------------------------------------------------------------------------
Medaka (XP_004065962.1)        ------------------------------------------------------------------------------------------
Platyfish (ENSXMAP00000005447) ------------------------------------------------------------------------------------------
Spotted _gar (ENSLOCP000000155 ------------------------------------------------------------------------------------------
Stickleback (ENSGACP0000002318 ------------------------------------------------------------------------------------------
Tilapia (XP_005457058.1)       ------------------------------------------------------------------------------------------
Tetraodon (ENSTNIP00000021201) ------------------------------------------------------------------------------------------
Zebrafish (ENSDARP00000101295) ------------------------------------------------------------------------------------------
Salmon (XP_014042431.1)        ------------------------------------------------------------------------------------------
Salmon (XP_014065146.1)        ------------------------------------------------------------------------------------------
Salmon (XP_014050290.1)        ------------------------------------------------------------------------------------------
Rainbow trout (XP_021429026.1) ------------------------------------------------------------------------------------------
Rainbow trout (XP_021429023.1) ------------------------------------------------------------------------------------------
Shark (XP_007910136.1)         ------------------------------------------------------------------------------------------
Human (ENSP00000386869)        ------------------------------------------------------------------------------------------
Gorilla (ENSGGOP00000051172)   ------------------------------------------------------------------------------------------
Armadillo (ENSDNOP00000021516) ------------------------------------------------------------------------------------------
Mouse (ENSMUSP00000087049)     ------------------------------------------------------------------------------------------
Rat (ENSRNOP00000015296)Tet3   ------------------------------------------------------------------------------------------
Pig (ENSSSCP00000008852)       ------------------------------------------------------------------------------------------
Elephant  (XP_010591957.2)     ------------------------------------------------------------------------------------------
Whale (XP_023989264.1)         ------------------------------------------------------------------------------------------
Tasmanian_devil (ENSSHAP000000 ------------------------------------------------------------------------------------------
```

```
Chicken (ENSGALT00000059756)           ------------------------------------------------------------------------------------------
Lizard (ENSACAT00000001585)            ------------------------------------------------------------------------------------------
Turtle (ENSPSIT00000006549)            ------------------------------------------------------------------------------------------
Xenopus (ENSXETP00000054061)           ------------------------------------------------------------------------------------------
Coelacanth (ENSLACP00000009535         ------------------------------------------------------------------------------------------
Spotted_gar (ENSLOCP0000001981         ------------------------------------------------------------------------------------------
Amazon_molly (ENSPFOP000000021         ------------------------------------------------------------------------------------------
Cave_fish (ENSAMXP00000015689)         ------------------------------------------------------------------------------------------
Fugu (ENSTRUP00000036865)              ------------------------------------------------------------------------------------------
Medaka (ENSORLP00000019236)            ------------------------------------------------------------------------------------------
Platyfish (ENSXMAT00000000130)         ------------------------------------------------------------------------------------------
Stickleback (ENSGACP0000001732         ------------------------------------------------------------------------------------------
Tetraodon (ENSTNIP00000019883)         ------------------------------------------------------------------------------------------
Tilapia (ENSONIP00000019943)           ------------------------------------------------------------------------------------------
Zebrafish (ENSDARP00000085229)         ------------------------------------------------------------------------------------------
Rainbow_trout (XP_021462005.1)         ------------------------------------------------------------------------------------------
Rainbow_trout (XP_021477599.1)         ------------------------------------------------------------------------------------------
Salmon (XP_014026412.1)                ------------------------------------------------------------------------------------------
Salmon (XP_014017100.1)                ------------------------------------------------------------------------------------------

                                               370       380       390       400       410       420       430       440       450
                                       ....|....|....|....|....|....|....|....|....|....|....|....|....|....|....|....|....|....|
European ampioxus (Sc0000095)          ------------------------------------------------------------------------------------------
Ciona intestinalis (XP_0021254         ------------------------------------MSNQMYQPGTSPNPSYSGQMYCSTNPSITPNGNSRFPYQQSNVQRPDYPNKPGM
Lamprey (scaf_00044)                   ------------------------------------------------------------------------------------------
Lamprey (scaf_03335)                   ------------------------------------------------------------------------------------------
Shark (XP_007897316.1)                 ------------------------------------------------------------------------------------------
Lizard (XP_020663651.1)                ILHSEVFQEGCKDQELVSGKQDLDNLFNTSNAAESVTSHSHLDSAVALKIPGNESGTSMKTPLKSSLDLPPASKGHSGSSCDSTVPLSSD
Chicken (ENSGALP00000053457)           -------------------------QVADDNTAVSEVEYEHED------LPCEPTLVDLNASSATPFDLVPNSKESDSGSID-----TSD
Turtle (ENSPSIP00000006704)            ------------------------------------------------------------------------------------------
Human (ENSP00000362748)                AEILPGPLEGTRCGEGLFSEETLNDTSGSPKMFAQDTVCAPFPQRATPKVTSQGNPSIQLEELGSRVESLKLSDSYLDPIKSEHDCYPTS
Gorilla (ENSGGOP00000011536)           AEILPGPLEGTRCGEGLFSEETLNDTSGSPKMFAQDTVCAPFPQRATPKVTSQGNPSNQLEELGSRVESLKLSDSYLDPIKSEHDCYPTS
Mouse (ENSMUSP00000133279)             AEIS-WPLEGTHC-EELLSHQTSDNECTSP------QECAPLPQRSTSEVTSQKNTSNQLADLSSQVESIKLSDPSPNPTGSDHNGFPDS
Rat (ENSRNOP00000054747)               AGIS-WSLEGTHS-EGLLSHQTSNNVSNSH------PGCAPSPQRSTSEVTSEKNASDQFVDLCSPVESPKLSDPSQNPMGSEHNCFPDS
Pig (ENSSSCP00000010918)               AEILSESLEGTCGGEGLFSKETLNDNSDSPGTFAQDTLCVPLLQRAALKVTSEGSPSIQLEDLGSRVESLKLSDSYLDPIKSEHDCCPTS
Whale (XP_023987950.1)                 AEVLSGSLEGTRGGKGLFSRETLNDISDSPRMFAQDTLCVPLLQKAALRVTSEGNPSIQLEDLGSRVESLKLSDSYLDPIKSEHDSCPTS
Elephant (ENSLAFP00000005179)          AEIHCKSLEGTHG-EGLFSEETLNDIEDYTRMFAQDTECAPLPQRAISNVTSERNSSVQLEDLGSRVESLKLSDSHLDPIKREHDCYPTS
Armadillo (ENSDNOP00000014565)         AEIHSGSLEGAHGDEGLFSGQTVNDTSD-------FAICTPLPQRTAPKVTSERTPSLQLEDLGSRVESLKLSDSYLDPIKSEHDCCPTS
Tasmanian_devil (XP_023360087.         ------------------------------------------------------------------------------------------
Coelacanth (ENSLACP00000016889         ------------------------------------------------------------------------------------------
Cavefish(XP_007240334.2)               RGSP-----------------------------LFQTFPQEVKPTRRAPSSRGRGQQAREQDNFKITECNDPNSDD-QQKKALVTNGEAQ
Platyfish (XP_014325204.1)             LKPATEVTACDDSDGRVG--------------DGSGDHTSGSSELTGSGSLGAQGSSEKNDTSSVHGNELDHLDSSGMQDEGGAVQQEDC
Tilapia (XP_005456583.2)               LKSVSGAPEGDDADGQIKL-----------CSERSKEAPPESSESTTAVSGEVQGSCEGNDTSAIRGKKFDHPASDGKEDNVCTAQQEQN
Spotted_gar (XP_015202470.1)           PEDVPDP-------------------------HHPIELGKEDATGQDVSSPSSGDCSVSRDPGQIETEVTERESVETLPSMVEETEPVSS
Stickleback (ENSGACT0000001035         ------------------------------------------------------------------------------------------
Tetraodon (ENSTNIT00000011701)         ------------------------------------------------------------------------------------------
Fugu (XP_011601624.1)                  LECGVGAP-----DRQLKP-----------CGDTDEASPSEPSELARMGPPGLQGVRQGTSEG------IESSDDPGRKDDDFASDGKER
Zebrafish (ENSDARP00000143362)         RKSV-----------------------------SCPSIQRDVQ-------KKGAQEIREQETETKRSEVNDPNGDAEQQKGAEIPTGEGQ
Amazon_molly (XP_016534180.1)          SKPATEVTACDDSDGRVG--------------DGSGDHTSGSSELTGSGSPGAQGSSEKNDTSSVHGNELDHLDSSGKQDDGGAVQQEEG
Medaka (XP_023818916.1)                SKHVSGHPVCVETDEQPTL-----------CSGGSKDCPSGTSELTS--SSGVQDS-NGNGNLSFPGNESDLLANDHKPDGAFSLQEEQG
Rainbow_trout (XP_021469805.1)         HSELNVPISVDQQQEKLVNNISEGNSAQFYQSHELGDSTSDERQEVSVVPKGEDKAKINLGAEQIEMEQRERDTHAEEQRGGERLEKDGK
Rainbow trout (XP_021436521.1)         HYELDEHTSVDQQQEKLINNINESNS--FNLNHELGDSTSEHRQEESVGPKCEDNANINLGAEQIEMEQRERNTHAGEQRGGESLEKEG-
Salmon (XP_014010485)                  HSELNDPISVDQQQEKLVNNISEGNSGQFNQSHELGDSTSDERQEVSVVPKGEDKAKINLGPEQIEMEQRERDTHAKEQRGGERLEKEGK
Salmon (XP_014070638.1)                HYDLDEPTSVDQQQEKLINNIAEGNS--LNLSHELGDSTSDDRQEESVGPKCEDNAKINLGAEQIEMEQRERDTHAGEQRGGERLEKEG-
Shark (XP_007890120.1)                 ------------------------------------PGYPDLQQQNEHSKVETKNASYKNGDIFSLLRSKQLSVSNGATGSSPFIEIQQD
Human (ENSP00000425443)                ------------------------------------PENP-ELQILNEQEG--KSANYHDKNIV-LLKNKAVLMPNGATVSASSVEHTHG
Gorilla (ENSGGOP00000034525)           ------------------------------------PENP-ELQILNEQEG--KSANYHDKNIV-LLKNKAVLMPNGATVSASSMEHTHG
Mouse (ENSMUSP00000143029)             --------------------------------------GVE-IQVLNEQEG--EKGRS-----VTLLKNKIVLMPNGATVSAHSEENTRG
Rat (ENSRNOP00000048956)               --------------------------------------GVENTEIPEEQEG--GNDRN-----ALLLKNKAVLMPNGATVSAPSVEDTHG
Pig (ENSSSCP00000009765)               ------------------------------------SENP-ELQILNKQEG--KNANYYDKNIR-ILKNQAVLMPNGATVSASSMENTRG
Tasmanian_devil (ENSSHAT000000         ------------------------------------SENS-QLKNQNEQER--KNASYSNKNIVLYLKNKAVPMPNGATVSASSMENTHG
Whale (XP_007106564.2)                 ------------------------------------SENP-ELQILNQQEG--KNANYHDKNIVLLLKNKAVLMPNGATVSASSMENTHG
Elephant (XP_023404313.1)              ------------------------------------SENP-GLQILNEQVE--KNVNYHDRNIVLLLKNKTVLMPNGATVSASSMENTHG
Lizard (ENSACAP00000008438)            ------------------------------------------------------------------------------------------
Chicken (ENSGALP00000017216)           ------------------------------------SEDPRELLIQDEQEG--ENINCHNRDIVLLLKNKAVPMPNGATVSASSMDSMHG
Turtle (ENSPSIP00000011691)            ------------------------------------SENPEDLQIQNEQER--ENMTCHNKDIVLLLKNKAVPMPNGATVSASSMENMSG
Xenopus (ENSXETP00000030770)           -----------------------------------------ALQVPNEQKK---DLNYSNRDIVSCLKDKTVPMCNGATASVSSMENVNN
Coelacanth (ENSLACP00000008576         ------------------------------------LESP-NLQNRNQHKLGKNNFSYHNGDLLLHFKNKKVPIPNGATVPTSSMDNMHG
Amazon_molly (ENSPFOP000000120         ------------------------------------EQTSVQTDINFDIRN----CHIPNRDIFSRPRNKSVP--NGAVSSSSTIESTPG
Amazon_molly (ENSPFOP000000299         ------------------------------------EQTSVQTDINFDIRN----CHIPNRDIFSRPRNKSVP--NGAVSSSSTIESTPG
Fugu (XP_011611191.1)                  ------------------------------------KCNTQQTDLLLDKRN----CHLPNGDIFRPPRSKQVSTPNNAGSPSSTSENTPG
Medaka (XP_004065962.1)                ------------------------------------ECSSSQREFSLDKRN----HSIQNGDIFGRPRNKQFP--NGAVSASSTLESTPG
Platyfish (ENSXMAP00000005447)          ------------------------------------EQTSAQTDINFDIRN----CRIPNRDIFSRPRNKSVP--NGAVSSSSTIESTPG
Spotted _gar (ENSLOCP000000155         ------------------------------------QCPDTQKELNLNKEN----CNYSNGDIFSLSRDKKVPISNGAMVTSSSMEDMPD
Stickleback (ENSGACP0000002318         ------------------------------------------------------------------------------------------
Tilapia (XP_005457058.1)               ------------------------------------GCNSQTSEIKFDSRN----CNFRNGDIFCVPRNKQVP--NGAVSSPSTIESTPG
Tetraodon (ENSTNIP00000021201)         ------------------------------------------------------------------------------------------
Zebrafish (ENSDARP00000101295)         ------------------------------------PKHNKAGDLTHAKIN----CNFTNGDLFSLSRNKQVQMPNGATTSPPSVKGTTG
Salmon (XP_014042431.1)                ------------------------------------------------------------------------------------------
Salmon (XP_014065146.1)                ------------------------------------DCDG--TRLDKRNCN----TFSNGGDIFSLSRNKQIA--NGATVSPNSIEGTPG
Salmon (XP_014050290.1)                ------------------------------------DCDG--TRLDKRNCN----TFSNGGDIFSLSRNKQIA--NGATVSPNSIKGTPG
Rainbow trout (XP_021429026.1)         ------------------------------------------------------------------------------------------
Rainbow trout (XP_021429023.1)         ------------------------------------DCDG--TRLDKRNCN----TFSNGGDIFSLSRNKQIA--NGATVSPNSIEGTPG
Shark (XP_007910136.1)                 ------------------------------------------------------------------------------------------
Human (ENSP00000386869)                ----------------------------------------QKPDWEAAPGPAHTARLEDAHDLVAFSAVAEAVSSYGALSTRLYETFN--
Gorilla (ENSGGOP00000051172)           ----------------------------------------QKPDWEAAPGPAHTARLEDAHDLVAFSAVAEAVSSYGALSTRLYETFN--
Armadillo (ENSDNOP00000021516)         ----------------------------------------PKPDWDAAPGPAHAARLEDAHDLVAFSAVAEAVSSYGALSTRLYETFN--
Mouse (ENSMUSP00000087049)             ----------------------------------------QKPDWEAASGPTHAARLEDAHDLVAFSAVAEAVSSYGALSTRLYETFN--
```

```
Rat (ENSRNOP00000015296)Tet3        ----------------------------------------QKPDWDAASGPAHTARLEDAHDLVAFSAVAEAVSSYGALSTRLYETFN--
Pig (ENSSSCP00000008852)            ----------------------------------------QKPDWDAAPGPAHTARLEDAHDLVAFSAVAEAVSSYGALSTRLYETFN--
Elephant  (XP_010591957.2)          ----------------------------------------QKSDWDAAPGPTHTARLEDAHDLVAFSAVAEAVSSYGALSTRLYETFN--
Whale (XP_023989264.1)              ----------------------------------------QKPDWDVTPGPAHTARLEDAHDLVAFSAVAEAVSSYGALSTRLYETFN--
Tasmanian_devil (ENSSHAP000000      ----------------------------------------QKPDWEPSPSPAHAAHPEDAHDLVAFSAVAEAVSSYGALSTRLYETFN--
Chicken (ENSGALT00000059756)        --------------------------------LAAGGRPQAGGSALMEPSGWLPGPPPDARNLVAFSAVAEAVSSYR-LPAPASPSLL--
Lizard (ENSACAT00000001585)         ------------------------------ADQVTGWESHGGPPASSSSSSSCNANLEDAQNLVAFSAAVEAAMSSQGLPPLASPSLLSV
Turtle (ENSPSIT00000006549)         -----------------------------------------------SASDYIKSVFKRPE---AVPNKVKAGKPKQELPAPP-------
Xenopus (ENSXETP00000054061)        ------------------------------APQDPCNKLNWDKPTIPNHAANNNSNLEDAKNLVAFSAVAEAMSTYG-MPASGTPSSVSL
Coelacanth (ENSLACP00000009535      ---------------------------------QNENPNWENTPVLQQTTATHNASLEDARNLAAFSAVVEAVSSYKTPASEPQPPQT-M
Spotted_gar (ENSLOCP0000001981      ------------------------------DGQEEVNNSTATTTSTFAGATHNDVDMEDARNLVAFSTTPVSMLPPYSTAGIGMAELQTM
Amazon_molly (ENSPFOP000000021      ------------------------------------------------------ADIEDAQNLVAFS-ATASSLPPSSSSSCLVLQPNTA
Cave_fish (ENSAMXP00000015689)      ------------------------------NPQQQQAG----WSDHAAGSSVNNVDMENARTLVAFS-ASAG-SLSTCN-VSKPSQSPTA
Fugu (ENSTRUP00000036865)           ------------------------------GQCKEQQPCWNGSHGANPVQPVHHAAMEDAHNLVAFS-AIAGSLPPSSSSPCNVQLN-TA
Medaka (ENSORLP00000019236)         ------------------------------------------------------------------------------------------
Platyfish (ENSXMAT00000000130)      ------------------------------------------------------------------------------------------
Stickleback (ENSGACP0000001732      ------------------------------------------------------------------------------------------
Tetraodon (ENSTNIP00000019883)      ------------------------------------------------------------------------------------------
Tilapia (ENSONIP00000019943)        --------------------------------------------------PAHHADMEDAHNLVAFS-AIAGSLPHSSSSSCHLLQATTA
Zebrafish (ENSDARP00000085229)      ------------------------------------------------------------------------------------------
Rainbow_trout (XP_021462005.1)      ------------------------------QPQKQLPCSGWSSSGRTPGKPVHEADMEDARNLVAFS-ASVN-VGSLAP--IPEAQSYTA
Rainbow_trout (XP_021477599.1)      ------------------------------QPQQQQHCNGWTSSGRTPGKPVHEADMEVARNLVAFS-ASVS-VGSLPPSGHLEPQSHAI
Salmon (XP_014026412.1)             ------------------------------QPQKQLPCSGWGSFGRTPGKPVHEADMEDARNLVAFS-ASVN-VGSLAP--IPEAQSYTA
Salmon (XP_014017100.1)             ------------------------------QPNQQQHCNGWSSSGRTPGKPVHEADMEVARNLVAFS-ASVS-VGSLPPSGHLEPQSHTA

                                           460       470       480       490       500       510       520       530       540
                                    ....|....|....|....|....|....|....|....|....|....|....|....|....|....|....|....|....|....|
European ampioxus (Sc0000095)       ------------------------------------------------------------------------------------------
Ciona intestinalis (XP_0021254      GQPGSQRYPSYPNQPHQSHPVYPNAQLNSRHPGLQPQAPYPG------------------------------------------------
Lamprey (scaf_00044)                ------------------------------------------------------------------------------------------
Lamprey (scaf_03335)                ------------------------------------------------------------------------------------------
Shark (XP_007897316.1)              ------------------------------------------------------------------------------------------
Lizard (XP_020663651.1)             TNSKVPAERSESPADPHSKNHCDVVLR--HCEPDAVISITSELISKLNLDDPCVLPES--------------------HHRQDPEPASTE
Chicken (ENSGALP00000053457)        PSSAVLLEELVPLAESHTEAESDLVLASEEHNSNSAPTVTSELNSQECLEDLSSFAES--------------------CLKGDGDLESEN
Turtle (ENSPSIP00000006704)         ------------------------------------------------------------------------------------------
Human (ENSP00000362748)             SLNKVIPDLNLRNCLALGGSTSPTSVIKFLLAGSKQATLGAKPDHQEAFEATANQQ-----EVSDTTS----------FLGQAFGAIPHQ
Gorilla (ENSGGOP00000011536)        SLNKVIPDLNLRNCLALGGSTSPTSVIKFLLAGSKQATLGAKPDHQEAFEATANQQ-----EVSDTTS----------FLGQAFGAIPHQ
Mouse (ENSMUSP00000133279)          SF-RIVPELDLKTCMPLDESVYPTALIRFILAGSQPDVFDTKPQ-EKTLITTPEQVGSHPNQVLDATS----------VLGQAFSTLPLQ
Rat (ENSRNOP00000054747)            SF-RIVPELDLKTCMSLDESVYPTALIKFILAGSQPDVLDTKSE-EETLKTTPEKVGSHPNQVVDATS----------ILGQAFSTLPHQ
Pig (ENSSSCP00000010918)            SFNKVVPELDLRNCLSIGGSIYPTSLIKLLLAGSEQETLCAKPDYQEVLKATPDQQ-----EIPDTTP----------VLGQVFSAIPHP
Whale (XP_023987950.1)              SFNKVVPELDLRNCLSIGGSIYPTSLIKLLLAGSEQVTLGAKPDHQEVLKSTPDEQ-----EVPDTTP----------VLGQAFSAVPHQ
Elephant (ENSLAFP00000005179)       SFNKVIPELDLRNCLSIGGSVYPTSLIKLLLAGSEQETLGANSDHQEALKATLDQQ-----EVSGATPNQQKIPDTSSVLGEVFSATPHQ
Armadillo (ENSDNOP00000014565)      SFNKVIPELDLRNCLSLGGSIYPTSLIKLLLAGSEQETLGANSDHQEALKATPFQQ-----EIPGATP----------VLREAFSAIPQQ
Tasmanian_devil (XP_023360087.      ------------------------------------------------------------------------------------------
Coelacanth (ENSLACP00000016889      ------------------------------------------------------------------------------------------
Cavefish(XP_007240334.2)            -------LVNRNSDETAEEEHREPLVHETPAVVDEEEVAVAAVTAETVQSENCLAFIDN-------------------------------
Platyfish (XP_014325204.1)          -------EELATKASRTDE--KALVVTER-------DVQSLEAKTEVERQDETMRVKEEIAVKLGSSESED-----------IWEKDTQT
Tilapia (XP_005456583.2)            -------NILILQDKTTDE--RVTPDDERREEINHERKESIEATKTVQRQKETVGEMEDITWKLGNCENKDGGKKQKENDVSIIPAHPQT
Spotted_gar (XP_015202470.1)        S------QSQETKLSHVDLEPPKSPNSDSTVIPHSNPTYLNEALNLGTDLYQMK------------------------------------
Stickleback (ENSGACT0000001035      ------------------------------------------------------------------------------------------
Tetraodon (ENSTNIT00000011701)      ------------------------------------------------------------------------------------------
Fugu (XP_011601624.1)               -------AELAEDDNKVKL--TENGPKERVIVCTGVKMNRREASLEPVETRKKTKEKQEDIS--------------------IYLTGSQP
Zebrafish (ENSDARP00000143362)      -------SVNISSKE-SGEEHRETVVNDTLVAEKKK----VKVNTETVQSESYSAVVE--------------------------------
Amazon_molly (XP_016534180.1)       -------EELAMKASRTDE--KPLVVIER-------DVESLEAETEVERQDETMIVKEEIAVKLGSSESED-----------IWEKDTQT
Medaka (XP_023818916.1)             -------NVLGTKGKPMDE--SVSVRQEIEEIN-YERGGSLQEETATETQQETVEEMEGTVERLGNCEGEAKRGEIIENRVSGGPTDSQI
Rainbow_trout (XP_021469805.1)      EAVE---EEAVEEGEKTDEEKKKAEGEERVEIVKVKETKEREEKDERLEKREFISRPPDTSSSISVSQPQKPPLQDDVLMMQSPVHPPSV
Rainbow trout (XP_021436521.1)      -------EETVKQEYERDEEKKEADGKQRVERVKVNETEERKEKNERQKEGEVISRPPDTSTIMSVAQPQKPIPQDDALMMQSPVHASPV
Salmon (XP_014010485)               EAVEKEGEEAMEEGEKTDEEKKKAEGEERVEIAKVKETKEREEKDERLEKREVISRPPDTSSSISVSQPQKPPLQDDVLMMQSPVHPSPV
Salmon (XP_014070638.1)             -------EETVKQEEERDEEKKEADGKQRVERVKVNETEERKEKNERQKEGEVISRPSDTSTIMSVAQPQKPTPQDDTLMMQSPVHASPV
Shark (XP_007890120.1)              GFRDKTLPLSYPEQVLIEQQNIASHEYTRRNQAIAELSHRM-------------------------------------------------
Human (ENSP00000425443)             ELLEKTLSQYYPDCVSIAVQK-TTSHINAINSQ--ATNELS-------------------------------------------------
Gorilla (ENSGGOP00000034525)        ELLEKTLSQYYPDCVSIAVQK-TTSHINAINSQ--ATNELS-------------------------------------------------
Mouse (ENSMUSP00000143029)          ELLEKT--QCYPDCVSIAVQS-TASHVNTPSSQ--AAIELS-------------------------------------------------
Rat (ENSRNOP00000048956)            EFLEKT--QCYPDCVSMAVQNNTTPHVNALSGQ--AAIELP-------------------------------------------------
Pig (ENSSSCP00000009765)            ELLEKTLSQYYPDCVSIAVQK-TTSHIHAINSQ--ATNELS-------------------------------------------------
Tasmanian_devil (ENSSHAT000000      ELLEKTLSPYYPDRVSIAMQK-TTSHINAINSQ--ATNELS-------------------------------------------------
Whale (XP_007106564.2)              ELLEKTLSQYYPDCVSIAVQK-TTSHIHAINSQ--ATNELS-------------------------------------------------
Elephant (XP_023404313.1)           ELLEKTLSQYYPDCVSIAVQK-TTSHINAINSL--ATNELL-------------------------------------------------
Lizard (ENSACAP00000008438)         ------------------------------------------------------------------------------------------
Chicken (ENSGALP00000017216)        ELLEKTLSQYYPEHVSIAMQK-NTSHINAITSQ--ATNELS-------------------------------------------------
Turtle (ENSPSIP00000011691)         ELLEKTLSQYYPDHVSIAMQK-NTSHINAINSQ--ATNELS-------------------------------------------------
Xenopus (ENSXETP00000030770)        ELLEKLPSQYYPEHVSTVVHN-NTSLANAINTLNDASNDLS-------------------------------------------------
Coelacanth (ENSLACP00000008576      DLLEKTLSQYYPDRVSIAMQK-NTSHKHAINSQ--AFGELS-------------------------------------------------
Amazon_molly (ENSPFOP000000120      DLLEKTLSQYYPEQVSIAPQA-SGSQLDTVNVSLATRSVGE-------------------------------------------------
Amazon_molly (ENSPFOP000000299      DLLEKTLSQYYPEQVSIAPQA-SGSQLDTVNVSLATRSVGE-------------------------------------------------
Fugu (XP_011611191.1)               DLLEKTLSQYYPDQVSIAQQA-SSPQLVVVNGSIANKFPSE-------------------------------------------------
Medaka (XP_004065962.1)             DLLEKTLSQYYPEQVSIAPQT-SGSQLDTVNGSLDNQLSSE-------------------------------------------------
Platyfish (ENSXMAP00000005447)       DLLEKTLSQYYPEQVSIATQA-SGSQLDTVNDSLATRSVGE-------------------------------------------------
Spotted _gar (ENSLOCP000000155      YLLEQTLSQYNPAHVSITSQN-NESQDNAIKSPAESNLEEQ-------------------------------------------------
Stickleback (ENSGACP0000002318      ------------------------------------------------------------------------------------------
Tilapia (XP_005457058.1)            DLLEKTLSQYYAEQVSIAPQT-SGSQLDAVNDSLPDKLSSE-------------------------------------------------
Tetraodon (ENSTNIP00000021201)      ------------------------------------------------------------------------------------------
Zebrafish (ENSDARP00000101295)      DLLEKTLSQYYPEHVSIAPQT-NSSQVDQVTSNLPDQATSS-------------------------------------------------
Salmon (XP_014042431.1)             ------------------------------------------------------------------------------------------
Salmon (XP_014065146.1)             DLLEKTLSQYYPEQVSIAAQT-GAQEFQPEVGLVDDISSLTNELPELPK-----------------------------------------
Salmon (XP_014050290.1)             DLLEKTLSQYYPEQVSIAAQT-GAQEFQPEVGLVDDISSLTNELPELPE-----------------------------------------
Rainbow trout (XP_021429026.1)      ------------------------------------------------------------------------------------------
Rainbow trout (XP_021429023.1)      DLLEKTLSQYYPEQVSIAAQT-GVQEFQPEVGLVDDISSLTNELPELPE-----------------------------------------
```

```
Shark (XP_007910136.1)              ------------------------------------------------------------------------------------------
Human (ENSP00000386869)             ---REMSREAGN-NSRGPRPGPEGCSAGS-----EDLDTLQT------------------------------------------------
Gorilla (ENSGGOP00000051172)        ---REMSREAGN-NSRGPRPGPEGCSAGS-----EDLDTLQT------------------------------------------------
Armadillo (ENSDNOP00000021516)      ---REMSGEAGD-ASRGPRPGPEGCPVGS-----EDLDTLQT------------------------------------------------
Mouse (ENSMUSP00000087049)          ---REMSREAGS-NGRGPR--PESCSEGS-----EDLDTLQT------------------------------------------------
Rat (ENSRNOP00000015296)Tet3        ---REMSREAGN-NGRAPR--PESCSEGG-----EDLDTLQT------------------------------------------------
Pig (ENSSSCP00000008852)            ---REMSREAGDDNSRGPQSGPEGCPAGS-----EDLDTLQT------------------------------------------------
Elephant  (XP_010591957.2)          ---REMSREAGN-NSREPRPGPESCSAGS-----EDLDTLQT------------------------------------------------
Whale (XP_023989264.1)              ---REMSREAGN-NSRGPRSGPESCSAGS-----EDLDTLQT------------------------------------------------
Tasmanian_devil (ENSSHAP000000      ---REMSREGGG-NLSGPQPGPESYSAGG-----EDLDTLQT------------------------------------------------
Chicken (ENSGALT00000059756)        --YKKFDTEMS---RGG--LAPRDGVPRG-----EDLHALKA------------------------------------------------
Lizard (ENSACAT00000001585)         RLYEKFNAEMK---AGESPSGPSAASDGP-----EDLSALQA------------------------------------------------
Turtle (ENSPSIT00000006549)         ---LKKEAGSS---HPKLASSQHLSQLLQ-----EADFHKKT------------------------------------------------
Xenopus (ENSXETP00000054061)        QLYEKFNYETN---RDNSGHLEGNAPSCP-----EDLNTLKA------------------------------------------------
Coelacanth (ENSLACP00000009535      QLYKKFELEMSRSPALDSSLPLPESSSGT-----EDLSTLRN------------------------------------------------
Spotted_gar (ENSLOCP0000001981      HLYEKFNQEMNTGGVSRGKTEAKGGSVGTGHGCTEDLNTLQM------------------------------------------------
Amazon_molly (ENSPFOP000000021      KLYEKFTQETCADGAH---SRVSAGAPETSCEPLEDLDILQT------------------------------------------------
Cave_fish (ENSAMXP00000015689)      QLYEKFIQEMG-GRGDDAKQKGWSGDGEKNCTP-EDLNTLQT------------------------------------------------
Fugu (ENSTRUP00000036865)           QLYERFSQEMGSNGAQ---ARLSAGTSEGSAQPPEDLKTLQT------------------------------------------------
Medaka (ENSORLP00000019236)         ------------------------------------------------------------------------------------------
Platyfish (ENSXMAT00000000130)      ------------------------------------------------------------------------------------------
Stickleback (ENSGACP0000001732      ------------------------------------------------------------------------------------------
Tetraodon (ENSTNIP00000019883)      ------------------------------------------------------------------------------------------
Tilapia (ENSONIP00000019943)        YLYEKFTQETGAGKAQ---ARISAGAPEGSCPPSEDLDTLQK------------------------------------------------
Zebrafish (ENSDARP00000085229)      ------------------------------------------------------------------------------------------
Rainbow_trout (XP_021462005.1)      QLYEKFNQEMGTAKGAAAQARLQAPEGGDQASPPEDLNTLQTTLKTALKT----------------------------------------
Rainbow_trout (XP_021477599.1)      QLYEKFNQEMGTGEGATAQDRLQAPEGENQASPPEDLNTLQN------------------------------------------------
Salmon (XP_014026412.1)             QLYEKFNQEMGTAKGAVAQARLQAPEGGDQASPREDLNNLQTTLKTALKT----------------------------------------
Salmon (XP_014017100.1)             QLYEKFNQEMGTGEGAAAQARLHAPEGGNQASPPEDLNTLQN------------------------------------------------

                                            550       560       570       580       590       600       610       620       630
                                    ....|....|....|....|....|....|....|....|....|....|....|....|....|....|....|....|....|....|
European ampioxus (Sc0000095)       ------------------------------------------------------------------------------------------
Ciona intestinalis (XP_0021254      ------------------------------------------------------------------------------------------
Lamprey (scaf_00044)                ------------------------------------------------------------------------------------------
Lamprey (scaf_03335)                ------------------------------------------------------------------------------------------
Shark (XP_007897316.1)              ------------------------------------------------------------------------------------------
Lizard (XP_020663651.1)             QPGLH----------------------------------------------------SLTESGNGLKDPVPCMESYLPKIVELEPGNKAL
Chicken (ENSGALP00000053457)        VHLAA----------------------------------------------------TGSNSLLHFESVSSLVESNM-KLVDFVPVHKEL
Turtle (ENSPSIP00000006704)         ------------------------------------------------------------------------------------------
Human (ENSP00000362748)             WELPG----------------------------------------------------ADPVHGEALGETPDLPEIPGAIPVQGEVFGTIL
Gorilla (ENSGGOP00000011536)        WELPG----------------------------------------------------ADPVHGEALGETPDLPEIPGAIPVQGEVFGTIL
Mouse (ENSMUSP00000133279)          WGFSG----------------------------------------------------ANLVQVEALGKGSDSPEDLGAI--------TML
Rat (ENSRNOP00000054747)            WGFPG----------------------------------------------------ANLVHSETLAKGSVSPEDLGAI--------TML
Pig (ENSSSCP00000010918)            WEVPG----------------------------------------------------ANPVHGEPLGETPNPPEIPGAIPVQGEVFGAIL
Whale (XP_023987950.1)              WEVPG----------------------------------------------------ANPVHGEPLGETPGPPEIPGAIPVQGEVFGALL
Elephant (ENSLAFP00000005179)       WKVPG----------------------------------------------------VNPVCGEALGETSSLPEVPGAIAVQGEVFGAIL
Armadillo (ENSDNOP00000014565)      WEVPG----------------------------------------------------ANPFYGEALGETPDPPEIPGTILAQQEVFGATQ
Tasmanian_devil (XP_023360087.      ------------------------------------------------------------------------------------------
Coelacanth (ENSLACP00000016889      ------------------------------------------------------------------------------------------
Cavefish(XP_007240334.2)            ------------------------------------------------------------TEQPSTTNESDDAFVKEHDPAVKVDYVCET
Platyfish (XP_014325204.1)          NTPTC---QIPDPTHWN-PLTAQSESP--VRVFSN-----------------------AATSYPTKALSNSDLCELEVVNQAKTDFQPTV
Tilapia (XP_005456583.2)            GTPAC---QTPDPPHSNKGLTAQSESPSCVLLVSN-----------------------TATSNPTKAPSTSESLGKVLS---KIDIQPAT
Spotted_gar (XP_015202470.1)        ----------------------------------------------------------SADSATSVSDPLKNAEDNNVVAESQREVSSSL
Stickleback (ENSGACT0000001035      ------------------------------------------------------------------------------------------
Tetraodon (ENSTNIT00000011701)      ------------------------------------------------------------------------------------------
Fugu (XP_011601624.1)               NTPGP---RTPDPSDANRRWTVQSEPPVRVPPVSN-----------------------TATSNTTKAPCTSEVLDPELLSQGKTDMQPPP
Zebrafish (ENSDARP00000143362)      --------------------------------------------------------------QPNTVFVDQQDPLK-HNPVNNVDFAYNN
Amazon_molly (XP_016534180.1)       NTPTC---QNPDPIHCN-PLTAQSESP--VRVFSN-----------------------AATSYPTKALSTSDLCELEVVNQAKTDLRPTV
Medaka (XP_023818916.1)             SLPAR---QTPHPLSSNNQLAAQSDLPAPALLFSN-----------------------QTTSSPTKVLCTS-----EDLSQHKTDVIPVT
Rainbow_trout (XP_021469805.1)      AEPATSDLVSPPLPHPHPTFTLHPTMSHDIKVLNQGAKLIQPTQTVEIHKPLKVPTTFHETSQTTSVHPVSLSLSVAFLSEPHKSLCVIP
Rainbow trout (XP_021436521.1)      SEPATSDLASPPLLHLP--SPPHPTVSHGMKVLKKGVKPIQPTQTVEIHKPLKGPTTCHETSQASPMHPVSLSFSVAFLSEPHKPLCVTP
Salmon (XP_014010485)               SEPATSDLASPPLPHPHPTSPPHPTMSHDIKVLNQGAKLIQPNQTVEIHKSLKVPTTFHETSQTTSVHPVSLSLSVAFLSEPHKSLCVIP
Salmon (XP_014070638.1)             SEPATSDLASPPLLRLP--SPPHPTVSHGMKVLNQGVKPIQPTKTVEIHKPLKDPTTCHETSQVSPMHPVSLSLSVAFLSEPHKPLCVTP
Shark (XP_007890120.1)              ------------------------------------------------------------------------------------------
Human (ENSP00000425443)             ------------------------------------------------------------------------------------------
Gorilla (ENSGGOP00000034525)        ------------------------------------------------------------------------------------------
Mouse (ENSMUSP00000143029)          ------------------------------------------------------------------------------------------
Rat (ENSRNOP00000048956)            ------------------------------------------------------------------------------------------
Pig (ENSSSCP00000009765)            ------------------------------------------------------------------------------------------
Tasmanian_devil (ENSSHAT000000      ------------------------------------------------------------------------------------------
Whale (XP_007106564.2)              ------------------------------------------------------------------------------------------
Elephant (XP_023404313.1)           ------------------------------------------------------------------------------------------
Lizard (ENSACAP00000008438)         ------------------------------------------------------------------------------------------
Chicken (ENSGALP00000017216)        ------------------------------------------------------------------------------------------
Turtle (ENSPSIP00000011691)         ------------------------------------------------------------------------------------------
Xenopus (ENSXETP00000030770)        ------------------------------------------------------------------------------------------
Coelacanth (ENSLACP00000008576      ------------------------------------------------------------------------------------------
Amazon_molly (ENSPFOP000000120      ------------------------------------------------------------------------------------------
Amazon_molly (ENSPFOP000000299      ------------------------------------------------------------------------------------------
Fugu (XP_011611191.1)               ------------------------------------------------------------------------------------------
Medaka (XP_004065962.1)             ------------------------------------------------------------------------------------------
Platyfish (ENSXMAP00000005447)      ------------------------------------------------------------------------------------------
Spotted _gar (ENSLOCP000000155      ------------------------------------------------------------------------------------------
Stickleback (ENSGACP0000002318      ------------------------------------------------------------------------------------------
Tilapia (XP_005457058.1)            ------------------------------------------------------------------------------------------
Tetraodon (ENSTNIP00000021201)      ------------------------------------------------------------------------------------------
Zebrafish (ENSDARP00000101295)      ------------------------------------------------------------------------------------------
```

```
Salmon (XP_014042431.1)          ------------------------------------------------------------------------------------------
Salmon (XP_014065146.1)          ------------------------------------------------------------------------------------------
Salmon (XP_014050290.1)          ------------------------------------------------------------------------------------------
Rainbow trout (XP_021429026.1)   ------------------------------------------------------------------------------------------
Rainbow trout (XP_021429023.1)   ------------------------------------------------------------------------------------------
Shark (XP_007910136.1)           ------------------------------------------------------------------------------------------
Human (ENSP00000386869)          ------------------------------------------------------------------------------------------
Gorilla (ENSGGOP00000051172)     ------------------------------------------------------------------------------------------
Armadillo (ENSDNOP00000021516)   ------------------------------------------------------------------------------------------
Mouse (ENSMUSP00000087049)       ------------------------------------------------------------------------------------------
Rat (ENSRNOP00000015296)Tet3     ------------------------------------------------------------------------------------------
Pig (ENSSSCP00000008852)         ------------------------------------------------------------------------------------------
Elephant  (XP_010591957.2)       ------------------------------------------------------------------------------------------
Whale (XP_023989264.1)           ------------------------------------------------------------------------------------------
Tasmanian_devil (ENSSHAP000000   ------------------------------------------------------------------------------------------
Chicken (ENSGALT00000059756)     ------------------------------------------------------------------------------------------
Lizard (ENSACAT00000001585)      ------------------------------------------------------------------------------------------
Turtle (ENSPSIT00000006549)      ------------------------------------------------------------------------------------------
Xenopus (ENSXETP00000054061)     ------------------------------------------------------------------------------------------
Coelacanth (ENSLACP00000009535   ------------------------------------------------------------------------------------------
Spotted_gar (ENSLOCP0000001981   ------------------------------------------------------------------------------------------
Amazon_molly (ENSPFOP000000021   ------------------------------------------------------------------------------------------
Cave_fish (ENSAMXP00000015689)   ------------------------------------------------------------------------------------------
Fugu (ENSTRUP00000036865)        ------------------------------------------------------------------------------------------
Medaka (ENSORLP00000019236)      ------------------------------------------------------------------------------------------
Platyfish (ENSXMAT00000000130)   ------------------------------------------------------------------------------------------
Stickleback (ENSGACP0000001732   ------------------------------------------------------------------------------------------
Tetraodon (ENSTNIP00000019883)   ------------------------------------------------------------------------------------------
Tilapia (ENSONIP00000019943)     ------------------------------------------------------------------------------------------
Zebrafish (ENSDARP00000085229)   ------------------------------------------------------------------------------------------
Rainbow_trout (XP_021462005.1)   ------------------------------------------------------------------------------------------
Rainbow_trout (XP_021477599.1)   ------------------------------------------------------------------------------------------
Salmon (XP_014026412.1)          ------------------------------------------------------------------------------------------
Salmon (XP_014017100.1)          ------------------------------------------------------------------------------------------

                                         640       650       660       670       680       690       700       710       720
                                 ....|....|....|....|....|....|....|....|....|....|....|....|....|....|....|....|....|....|
European ampioxus (Sc0000095)    ------------------------------------------------------------------------------------------
Ciona intestinalis (XP_0021254   ------------------------------------------------------------------------------------------
Lamprey (scaf_00044)             ------------------------------------------------------------------------------------------
Lamprey (scaf_03335)             ------------------------------------------------------------------------------------------
Shark (XP_007897316.1)           ------------------------------------------------------------------------------------------
Lizard (XP_020663651.1)          DSYFNSGQYYSG--CGEAKELSDHEHPSCTFLT---------------------------------------------------------
Chicken (ENSGALP00000053457)     DSCSDLSRTVENTESDSKNIFTASESVSDTSLT---------------------------------------------------------
Turtle (ENSPSIP00000006704)      ------------------------------------------------------------------------------------------
Human (ENSP00000362748)          DQQETLGMSGS--VVPDLPVFLPVPPNPIATFN---------------------------------------------------------
Gorilla (ENSGGOP00000011536)     DQQETLGMSGS--VVPDLPVFLPVPPNPIATFN---------------------------------------------------------
Mouse (ENSMUSP00000133279)       NQQETVAMDMDRNATPDLPIFLPKPPNTVATYS---------------------------------------------------------
Rat (ENSRNOP00000054747)         NQPETLGMDMD--APPDLPIFLPMPPNTVATYS---------------------------------------------------------
Pig (ENSSSCP00000010918)         NQQ-VLGMGGG--TASDPPIFLPAPPNQIATYY---------------------------------------------------------
Whale (XP_023987950.1)           NQQ-VLGMVGG--TASDPDIFPPAPLNPIATCN---------------------------------------------------------
Elephant (ENSLAFP00000005179)    DQQETLGVSGG--AVPDLPIFFAAPPDPIVTYH---------------------------------------------------------
Armadillo (ENSDNOP00000014565)   EQE-TLGMGGG--AAPDLPILLPGPPNSVAICN---------------------------------------------------------
Tasmanian_devil (XP_023360087.   ------------------------------------------------------------------------------------------
Coelacanth (ENSLACP00000016889   ------------------------------------------------------------------------------------------
Cavefish(XP_007240334.2)         DTSGPTDPPPSDLQSCDVAIRSTVDDSSQPD---------------------------------------VSVASPVCEIPKESPALLEA
Platyfish (XP_014325204.1)       HGAKPQLAGSSTNSDAALKVQTVIVTRSTP----------------------------------------VIICRDSLQPRSLGKSDLRE
Tilapia (XP_005456583.2)         HGAKPQSPGCSVVTEAALMVQAVVVKRNMP----------------------------------------VIIRTDSLKLSNMRDSCHGE
Spotted_gar (XP_015202470.1)     GPGLDMEPEPDAINPKSPDVSIPSWNVLKEH------------------------------------------PQGCVREVSTAHALPST
Stickleback (ENSGACT0000001035   ------------------------------------------------------------------------------------------
Tetraodon (ENSTNIT00000011701)   ------------------------------------------------------------------------------------------
Fugu (XP_011601624.1)            GAGPQFLVSS-----------AVSVKRNTP----------------------------------------VIVHSGSFRPRYASDPSRGE
Zebrafish (ENSDARP00000143362)   NTSGPPTPSPSDQ---VEAIQPTLPPVDLQD---------------------------------------ISLQTTRGNSTEEDANQIEE
Amazon_molly (XP_016534180.1)    HGAKPQLAGSSPNSDVALKVQTVIVTRSTP----------------------------------------VIICRDSLQPRSLGKSDLRE
Medaka (XP_023818916.1)          DGAKLLVPGSSAVPDTALTVQTVIVKRNMP----------------------------------------VIVHRDSLKPMSFGENSFGG
Rainbow_trout (XP_021469805.1)   ANPEPGQSPESPVSPEARQLLRPVDKETKESSNHLNVKLSSSS--ASSSSDLYTNAEVP-----------ITVSVPDLGPLSNNHNNYPE
Rainbow trout (XP_021436521.1)   AKPAPSQSLEIPISLEARQLLRPVDKETKESSNHSNTKYMSSSSSASSPSHLDINAEVPTTLSQSNTDVNITVSVSDLSPVPKNHNNYPE
Salmon (XP_014010485)            ANPAPGQSLESPVSPEARQLLRPVDEETKESSNHLNAKFSSSS--ASSSSDLYTNAEVP-----------ITVSVPDLGPLSNNHNNYPE
Salmon (XP_014070638.1)          AKPAPSQSLEIPISLEARQFLRPVDEETKESSNHSNTKYMSSSSSASSSSHLNTNAEVPTTLSQSNTDVNITVSVPDLSPVPKNHNNNPE
Shark (XP_007890120.1)           ------------------------------------------------------------------------------------------
Human (ENSP00000425443)          ------------------------------------------------------------------------------------------
Gorilla (ENSGGOP00000034525)     ------------------------------------------------------------------------------------------
Mouse (ENSMUSP00000143029)       ------------------------------------------------------------------------------------------
Rat (ENSRNOP00000048956)         ------------------------------------------------------------------------------------------
Pig (ENSSSCP00000009765)         ------------------------------------------------------------------------------------------
Tasmanian_devil (ENSSHAT000000   ------------------------------------------------------------------------------------------
Whale (XP_007106564.2)           ------------------------------------------------------------------------------------------
Elephant (XP_023404313.1)        ------------------------------------------------------------------------------------------
Lizard (ENSACAP00000008438)      ------------------------------------------------------------------------------------------
Chicken (ENSGALP00000017216)     ------------------------------------------------------------------------------------------
Turtle (ENSPSIP00000011691)      ------------------------------------------------------------------------------------------
Xenopus (ENSXETP00000030770)     ------------------------------------------------------------------------------------------
Coelacanth (ENSLACP00000008576   ------------------------------------------------------------------------------------------
Amazon_molly (ENSPFOP000000120   ------------------------------------------------------------------------------------------
Amazon_molly (ENSPFOP000000299   ------------------------------------------------------------------------------------------
Fugu (XP_011611191.1)            ------------------------------------------------------------------------------------------
Medaka (XP_004065962.1)          ------------------------------------------------------------------------------------------
Platyfish (ENSXMAP00000005447)   ------------------------------------------------------------------------------------------
```

```
Spotted _gar (ENSLOCP000000155 ------------------------------------------------------------------------------------------
Stickleback (ENSGACP0000002318 ------------------------------------------------------------------------------------------
Tilapia (XP_005457058.1)       ------------------------------------------------------------------------------------------
Tetraodon (ENSTNIP00000021201) ------------------------------------------------------------------------------------------
Zebrafish (ENSDARP00000101295) ------------------------------------------------------------------------------------------
Salmon (XP_014042431.1)        ------------------------------------------------------------------------------------------
Salmon (XP_014065146.1)        ------------------------------------------------------------------------------------------
Salmon (XP_014050290.1)        ------------------------------------------------------------------------------------------
Rainbow trout (XP_021429026.1) ------------------------------------------------------------------------------------------
Rainbow trout (XP_021429023.1) ------------------------------------------------------------------------------------------
Shark (XP_007910136.1)         ------------------------------------------------------------------------------------------
Human (ENSP00000386869)        ------------------------------------------------------------------------------------------
Gorilla (ENSGGOP00000051172)   ------------------------------------------------------------------------------------------
Armadillo (ENSDNOP00000021516) ------------------------------------------------------------------------------------------
Mouse (ENSMUSP00000087049)     ------------------------------------------------------------------------------------------
Rat (ENSRNOP00000015296)Tet3   ------------------------------------------------------------------------------------------
Pig (ENSSSCP00000008852)       ------------------------------------------------------------------------------------------
Elephant  (XP_010591957.2)     ------------------------------------------------------------------------------------------
Whale (XP_023989264.1)         ------------------------------------------------------------------------------------------
Tasmanian_devil (ENSSHAP000000 ------------------------------------------------------------------------------------------
Chicken (ENSGALT00000059756)   ------------------------------------------------------------------------------------------
Lizard (ENSACAT00000001585)    ------------------------------------------------------------------------------------------
Turtle (ENSPSIT00000006549)    ------------------------------------------------------------------------------------------
Xenopus (ENSXETP00000054061)   ------------------------------------------------------------------------------------------
Coelacanth (ENSLACP00000009535 ------------------------------------------------------------------------------------------
Spotted_gar (ENSLOCP0000001981 ------------------------------------------------------------------------------------------
Amazon_molly (ENSPFOP000000021 ------------------------------------------------------------------------------------------
Cave_fish (ENSAMXP00000015689) ------------------------------------------------------------------------------------------
Fugu (ENSTRUP00000036865)      ------------------------------------------------------------------------------------------
Medaka (ENSORLP00000019236)    ------------------------------------------------------------------------------------------
Platyfish (ENSXMAT00000000130) ------------------------------------------------------------------------------------------
Stickleback (ENSGACP0000001732 ------------------------------------------------------------------------------------------
Tetraodon (ENSTNIP00000019883) ------------------------------------------------------------------------------------------
Tilapia (ENSONIP00000019943)   ------------------------------------------------------------------------------------------
Zebrafish (ENSDARP00000085229) ------------------------------------------------------------------------------------------
Rainbow_trout (XP_021462005.1) ------------------------------------------------------------------------------------------
Rainbow_trout (XP_021477599.1) ------------------------------------------------------------------------------------------
Salmon (XP_014026412.1)        ------------------------------------------------------------------------------------------
Salmon (XP_014017100.1)        ------------------------------------------------------------------------------------------

                                        730       740       750       760       770       780       790       800       810
                               ....|....|....|....|....|....|....|....|....|....|....|....|....|....|....|....|....|....|
European ampioxus (Sc0000095)  ------------------------------------------------------------------------------------------
Ciona intestinalis (XP_0021254 ---------------------------------------------------------------------GHQQYNQYEHMSSFNGPNQPH
Lamprey (scaf_00044)           ------------------------------------------------------------------------------------------
Lamprey (scaf_03335)           ------------------------------------------------------------------------------------------
Shark (XP_007897316.1)         ---------------------------------------MEEGISNQDEEHGLEQSTRPFPGHRLHTEEEVTTAE-RDECTEG--QIQSL
Lizard (XP_020663651.1)        -----------SFALGDLERTLENIVPETFHPVA--VPVMPLNPEESTSDCVLVSSSQAIKES--WPSKGATLELPSDTAGGAEAG-ILT
Chicken (ENSGALP00000053457)   -----------NIEVEDIEQLVKCIDAETLILNSGYVPALSSDIEKRAVDNISSESSREFSEG--FVSELPSSTEI--SVLALEKA-INA
Turtle (ENSPSIP00000006704)    ---------------------------ETLVLDSDYVPISSSNTEKNTVALVSR-TTTVFQENSAHCSEGSTSSNIGSSTLISVSG-KNV
Human (ENSP00000362748)        -----------APSKWPEPQSTVSYGLAVQGAIQILPLG-SGHTPQSSSNSEKNSLPPVMAISNVENEKQVHISFLPANTQGFPLAPERG
Gorilla (ENSGGOP00000011536)   -----------APSKWPEPQSTVSYGLAVQGAIQILPLG-SGHTPQSSSNSEKNSLPPVMAISNVENEKQVHISFLPANTQGFPLASERG
Mouse (ENSMUSP00000133279)     -----------SPLLGPEPHSSTSCGLEVQGATPILTLD-SGHTPQLPPNPESSSVPLVIAANGTRAEKQFGTSLFPAVPQGFTVAAENE
Rat (ENSRNOP00000054747)       -----------CLLSGPEPHTSASCGLEVQGATPILTLD-SGHTPQPPQNSELSSVPLVIAANSTQAEKQFGANLFPANIQGFTIAPENE
Pig (ENSSSCP00000010918)       -----------PLPEWPEPLSTVSSGLQVQGAMQILPLG-SDHTPQPSSNSEISSVPPVMAINSIENEKQVHISFLPANTQGFTLAPESG
Whale (XP_023987950.1)         -----------AFPEWPEPQSTVSSGLEVQGTMQILPSG-LGHTPQPSSNSEISSVPPAMAINNIENKKKVHISFLPANTQGFTIAPERG
Elephant (ENSLAFP00000005179)  -----------VPPEWPEPQSTVSSGLEAQGAMRILPLGYSGHAPQSSSNSEKNLVPPAMAIRNKENEKQVHISFLPVYSQRFTLVPEKE
Armadillo (ENSDNOP00000014565) -----------ALPEWP-----VSYGLEAQGGTQVLPLG-SGHTPQSSN-----SVPPAKTISNKEKEKQAHVSFLPVSTQGFISTPERG
Tasmanian_devil (XP_023360087. ------------------------------------------------------------------------------------------
Coelacanth (ENSLACP00000016889 ---------------------ILQADLEKKAFNGSKSEHMEEGIPNGAETHSLKLNITPPLENGLHNQEETMTVLGETKWVDKGHQDFSI
Cavefish(XP_007240334.2)       TDVKY-------------AENGELDSTDDLKTKDENYDNDNDEFASKNEG--------------ALSLLSLSTGAYSGFLSTLDCGPSTT
Platyfish (XP_014325204.1)     TPVAG--------LEKQACTPGETKD-EGEIYKEAQKEKSFPSFMLLTEANTKP--PTG------KHEPNFVVDGTQATMPEDDPVPKIS
Tilapia (XP_005456583.2)       PHQPQSQEVLPNQGESLACTPAESQTKDSESIQEPLEHKSQKAVPLGSSALLSQ--LAAP-----STELELNLSEVQVAMPEQDSVPKIS
Spotted_gar (XP_015202470.1)   VLLVS-------------GPPCKEDTKECARSPEPRQELIAASKTP--------------------VPHCNIIADISESATALDSGFRTD
Stickleback (ENSGACT0000001035 ------------------------------------------------------------------------------------------
Tetraodon (ENSTNIT00000011701) ------------------------------------------------------------------------------------------
Fugu (XP_011601624.1)          TSLQP--ESLFSRGGRPVCTPAETQTKDTESGREPSKHLIKETLLMSSPPFSQLSVDVAA-----EFKPSPSKGGVLVVGPEQDPAPKIS
Zebrafish (ENSDARP00000143362) T---------------------ELRSTADVDTKDESID---AEMLMKKEG--------------ALSLLSLSAAIYNTTLCEMGHGPQAT
Amazon_molly (XP_016534180.1)  TPVAG--------VEKQACTPGKTKD-EGEIYKEPQKEKPFPSFMLLTEANTKP--STG------KHEPNFVVDGTRAAMPEDDPVPKIS
Medaka (XP_023818916.1)        SFQFQ----------RSVIPQPQGERGTRSTAETKDLDFQQHGFSCQKEAFPPP--FPQP-----GVAPSEPTDTFEAGMPEQDLVPKTS
Rainbow_trout (XP_021469805.1) EPPSS---SSLPYTKKDVFTTGSEPDKDIVSVSDCDLGSGPMTHNKYNQDEPQPSLPNTDTMSEPNTGSEPNTYIASVSVSDLGSVPKTS
Rainbow trout (XP_021436521.1) EPPSS---SSLPYTKIDLFTTGSAPDKDTVCVSVSDLGSVPKTHNKNNQDEPPPSFPYT------NTGSEPNTDIASVSVSDLGSVPKTS
Salmon (XP_014010485)          EPPSS---SSLPYTKKDVFTTGSEPDKDIVSVSDCDLGSGPMTHNKYNQDEPQPSLPNTDTRSEPNTGSEPNTDIASVSLSDLGSVPKTS
Salmon (XP_014070638.1)        APPSS---SSLPYTKIDLFTTGSAPDKDTVCVSVSDLGSVPKTHNTNNQDEPPPSLPYT------NTGSEPNTDIASVSVSDLGSVPKTS
Shark (XP_007890120.1)         --------------------------------------------------------------------------TQPPHTSGLTVSTWTS
Human (ENSP00000425443)         -----------------------------------------------------------------------CEITHPSHTSGQINSAQTS
Gorilla (ENSGGOP00000034525)   -----------------------------------------------------------------------CEITHPSHTSGQINSAQTS
Mouse (ENSMUSP00000143029)     -----------------------------------------------------------------------HEIPQPSLTSAQINFSQTS
Rat (ENSRNOP00000048956)       -----------------------------------------------------------------------REITQPSLTSAQINFSQTS
Pig (ENSSSCP00000009765)       -----------------------------------------------------------------------CEITHPSHTSGQINFPQTS
Tasmanian_devil (ENSSHAT000000 -----------------------------------------------------------------------YEVTHSSHTSGQTNSPLTS
Whale (XP_007106564.2)         -----------------------------------------------------------------------CEITHPSHTSGQINFPQTS
Elephant (XP_023404313.1)      -----------------------------------------------------------------------CEITHPSHTSGQINSPQTS
Lizard (ENSACAP00000008438)    ------------------------------------------------------------------------------------------
Chicken (ENSGALP00000017216)   -----------------------------------------------------------------------HETTHSSHTSGQITSPQTS
Turtle (ENSPSIP00000011691)    -----------------------------------------------------------------------YMTTHSSHTSGQIISPQTS
Xenopus (ENSXETP00000030770)   -----------------------------------------------------------------------TLATHPSHTSGQMTPQQTS
Coelacanth (ENSLACP00000008576 -----------------------------------------------------------------------HGTTHSSHTSGLTNSPQTS
```

```
Amazon_molly (ENSPFOP000000120   ------------------------------------------------------------------------GAQPPAVTSGLP------
Amazon_molly (ENSPFOP000000299   ------------------------------------------------------------------------GAQPPAVTSGLP------
Fugu (XP_011611191.1)            ------------------------------------------------------------------------NAKSHSSTSGSPGSAQIP
Medaka (XP_004065962.1)          ------------------------------------------------------------------------GAQPPALASGLLPSGQMP
Platyfish (ENSXMAP00000005447)   ------------------------------------------------------------------------GAQPPAVTSGLS------
Spotted _gar (ENSLOCP000000155   ------------------------------------------------------------------------ARNSPSHVSGLPISPQIP
Stickleback (ENSGACP0000002318   ------------------------------------------------------------------------------------------
Tilapia (XP_005457058.1)         ------------------------------------------------------------------------GAQLPPMTSGLPNSAQMP
Tetraodon (ENSTNIP00000021201)   ------------------------------------------------------------------------------------------
Zebrafish (ENSDARP00000101295)   ----------------------------------------------------------------------------PSFTSGFPISPQIS
Salmon (XP_014042431.1)          ------------------------------------------------------------------------------------------
Salmon (XP_014065146.1)          -----Q----------------------------------------------------------------ATTQSPTSLTSGFPPISAQM
Salmon (XP_014050290.1)          -----Q----------------------------------------------------------------ATTQSPTSLTSGFPPISAQM
Rainbow trout (XP_021429026.1)   ------------------------------------------------------------------------------------------
Rainbow trout (XP_021429023.1)   -----Q----------------------------------------------------------------VTTQSPTSLTSGFPPISAQM
Shark (XP_007910136.1)           ------------------------------------------------------------------------------------------
Human (ENSP00000386869)          ---------------------------------------------------------------------ALALARHGMKPPNCNCDGPEC
Gorilla (ENSGGOP00000051172)     ---------------------------------------------------------------------ALALARHGMKPPNCNCDGPEC
Armadillo (ENSDNOP00000021516)   ---------------------------------------------------------------------ALALARHGMKPPNCTCDGPEC
Mouse (ENSMUSP00000087049)       ---------------------------------------------------------------------ALALARHGMKPPNCTCDGPEC
Rat (ENSRNOP00000015296)Tet3     ---------------------------------------------------------------------ALALARHGMKPPNCNCDGPEC
Pig (ENSSSCP00000008852)         ---------------------------------------------------------------------ALALARHGMKPPNCNCDGPEC
Elephant  (XP_010591957.2)       ---------------------------------------------------------------------ALALARHGMKPPNCNCDGPEC
Whale (XP_023989264.1)           ---------------------------------------------------------------------ALALARHGMKPPNCNCDGPEC
Tasmanian_devil (ENSSHAP000000   ---------------------------------------------------------------------ALALARHGMKPPNCNCDGPEC
Chicken (ENSGALT00000059756)     ---------------------------------------------------------------------ALALAKHGMKPPNCNCDGPEC
Lizard (ENSACAT00000001585)      ---------------------------------------------------------------------ALALARHGVKPPNCNCDGPEC
Turtle (ENSPSIT00000006549)      ---------------------------------------------------------------------QMVLQQHLHHKRNLFLDQSLA
Xenopus (ENSXETP00000054061)     ---------------------------------------------------------------------ALALAKHGVKPPNCNCDGPEC
Coelacanth (ENSLACP00000009535   ---------------------------------------------------------------------ALTLARQGKKPPNCDCDGPEC
Spotted_gar (ENSLOCP0000001981   ---------------------------------------------------------------------ALKLAKHGTKPPNCNCDGPEC
Amazon_molly (ENSPFOP000000021   ---------------------------------------------------------------------ALRQAKHGHKPPNCNCDGHGC
Cave_fish (ENSAMXP00000015689)   ---------------------------------------------------------------------ALNQARHGHKPPNCDCEGPDC
Fugu (ENSTRUP00000036865)        ---------------------------------------------------------------------ALSQAKHGHKPPNCDCDGPDC
Medaka (ENSORLP00000019236)      ------------------------------------------------------------------------------------------
Platyfish (ENSXMAT00000000130)   ------------------------------------------------------------------------------------------
Stickleback (ENSGACP0000001732   ------------------------------------------------------------------------------------------
Tetraodon (ENSTNIP00000019883)   ------------------------------------------------------------------------------------------
Tilapia (ENSONIP00000019943)     ---------------------------------------------------------------------ALSQAKHGHKPPNCDCDGPDC
Zebrafish (ENSDARP00000085229)   ------------------------------------------------------------------------------------------
Rainbow_trout (XP_021462005.1)   ---------------------------------------------------------------------ALSQARHGHKPPNCDCDGPDC
Rainbow_trout (XP_021477599.1)   ---------------------------------------------------------------------ALSQARHGHKPPNCDCDGPDC
Salmon (XP_014026412.1)          ---------------------------------------------------------------------ALSQARHGHKPPNCDCDGPDC
Salmon (XP_014017100.1)          ---------------------------------------------------------------------ALSQARHGHKPPNCDCDGPDC
```

```
                                         820       830       840       850       860       870       880       890       900
                                 ....|....|....|....|....|....|....|....|....|....|....|....|....|....|....|....|....|....|
European ampioxus (Sc0000095)    ------------------------------------------------------------------------------------------
Ciona intestinalis (XP_0021254   INQPDKISPRVPQSRGHSSDKQWNFQAPPLIATNHSSVSNLTSPHELQQRS-------------AYPHPGRRPSNTPGECFPAVPPRHLS
Lamprey (scaf_00044)             ------------------------------------------------------------------------------------------
Lamprey (scaf_03335)             ------------------------------------------------------------------------------------------
Shark (XP_007897316.1)           HVNGDICKTFT-----AELDNLTKNEDVDFSIIDSDSSPKAQTLFAHPFSNVAKHIINSLNESSLP-------LKKFDLGESLDLVTNKT
Lizard (XP_020663651.1)          EEPIPKAQDPL-----PQSGLEDGKGIHL--VQDTRTYDAGLVVASEPTSCGNSVGSLLVSFTSLQPVAEKKKRRRCGVCEPCLRKTNCE
Chicken (ENSGALP00000053457)     DLPADLRLQQN-----DYSSQLESVGISLNLVQDNVSNSTQTVEASGPSSLTNPAGGYPPAYSSVLPMLEKKKRRRCGVCEPCLRKTNCE
Turtle (ENSPSIP00000006704)      HSPSDSKLQHK-----DSSSQSGSIGINLNSDQDSRSNSIGPIE---PSSITNSVYSYPLSYSSLLPMLEKKKRRRCGVCEPCLRKTNCE
Human (ENSP00000362748)          LFHASLGIAQL-----SQAGPSKSDRGSSQVSVTSTVHVVNTTVVTMPVPMVSTSSS---SYTTLLPTLEKKKRKRCGVCEPCQQKTNCG
Gorilla (ENSGGOP00000011536)     LFHASLGVAQL-----SQAGPSKSDRGSSQVSVTSTVHVVNTTVVTMPVPMVSTSSS---SYTTLLPTLEKKKRKRCGVCEPCQQKTNCG
Mouse (ENSMUSP00000133279)       VQHAPLDLTQG-----SQAAPSKLEGEISRVSITGSAD-VKATAMSMPVTQASTSSP---PCNSTPPMVERRKRKACGVCEPCQQKANCG
Rat (ENSRNOP00000054747)         LQHAPLDLTQG-----SHAAPSKLEGGISHVSMTSSADDVKATMMSMPVTRASTSSL---PCKSMPPTVERRKRKACGVCEPCQQKANCG
Pig (ENSSSCP00000010918)         HYHAPQGIAQL-----SQVSPS-RLEGGSSQISTTSTLDANTTVVSRPVSFASTSSSP--SYTTLLPTLEKKKRKRCGVCEPCQQKTNCG
Whale (XP_023987950.1)           LHHASQGIVHL-----SQASPS-KLEGGSSQISATSMVDINTTVVSRPVSLVSTSSSP--SYTTLLPTLEKKKRKRCGVCEPCQQKTNCG
Elephant (ENSLAFP00000005179)    LYQASLSVVPL-----SQPDPRKSVGGNSQVHTTSTVDVANPTLVSMSVSLASTSSS----YTTLLPTLEKKKRKRCGVCGPCQLKTNCG
Armadillo (ENSDNOP00000014565)   LYQASLGVAQL-----TQAGDSNKSEGGSSLLSTSPVDLANPTVGSLSMTLATTSSS---SYTTLLPTLEKKKRKRCGVCEPCQQKTNCG
Tasmanian_devil (XP_023360087.   -----------------------------------------------------MSDLFFIVPFRLSPFAET------GFENSFRLQVQGG
Coelacanth (ENSLACP00000016889   HLNGDINRTVA-----ETKNLKVTGEDNKVSFFHDSVEP--QKLFAQTFMNGFKPEVDPLTDSAVP-------LKKVKLEESTDSFKSNQ
Cavefish(XP_007240334.2)         ASLDSSK----------VSSSTDSTRFSFDTESEAGSLELALEHRAA-GASPLLDVDIRQRLLQAQVRKERKKRSRCGACGPCLLKYSCG
Platyfish (XP_014325204.1)       TSSVDSSSTLS------CCSESTRSSFSFDTESETGCGNASFSALP--GSWGTEGTGSRACPTLTSERKGRKKRSRCGACEPCLRKINCG
Tilapia (XP_005456583.2)         TPSLDSSSTFS------CSSESTRSSFSFETESEAGYGEPSLSVQP--GSWAQEGACSLAWTASKLQRKEGKKRRRCGKCEPCLRKINCG
Spotted_gar (XP_015202470.1)     VSTDDKS------------LYSSESNPPFDMGQELQCLDQTTPGVS---TSRPSSEVGSQYVSSDQEKRDKKKRRRCGVCEPCLRKINCG
Stickleback (ENSGACT0000001035   ------------------------------------------------------------------------------------------
Tetraodon (ENSTNIT00000011701)   ------------------------------------------------------------------------------------------
Fugu (XP_011601624.1)            IPSLDSSSTFS------FSSESARSSFSLDADSDVGYAEPGLSAPS--GSCSPEAASFSSRDAQKPQKKERKKRSRCGTCEPCLRKTNCG
Zebrafish (ENSDARP00000143362)   ALLSS---------------STESTQSSFDSESE-----LVLEHRTS-GKSIPQVEDIQLRLLQDQQRREQNKRGHCGVCAPCQCKVNCG
Amazon_molly (XP_016534180.1)    TSSVDSSSTLS------CCSESTRSSFSFDTESETGCGDASFSALP--GSWGPEGTGSLTCPTLTSERKERKKRSRCGACEPCLRKINCG
Medaka (XP_023818916.1)          AP-LDSSSTFS------CSSESTRSSFSFDTESESGYGEPTPSTLS--ERWGQEEAGLLFSSSPKLKRKERKKRNRCGTCEPCLRKINCG
Rainbow_trout (XP_021469805.1)   CIPNQESSSSKFSFSFSCSSESTQSSYSFDTESETGYGELSPPGHPSLDLGHLDGATSLPRTPRIIQRRERKKRSRCGGCEPCLRKINCG
Rainbow trout (XP_021436521.1)   SIPNQESSTSK----FSCSSESTQSSYSFDTESEQGYGAPSPPGHPSLGSRPLEEGTSLPRTPQVNQRRERKKRSSCGGCEPCLRKINCG
Salmon (XP_014010485)            SIPNQESSSSK--FSFSCSSESTQSSYSFDTESETGYGEPSPPGHHSLDLGHLDGGISLPQTPRIIQRRERKKRSRCGRCEPCLRNINCG
Salmon (XP_014070638.1)          SIPNQESCTSK----FSCSSESTQSSYSFDTESEQGYGAPSPPGHPSLGPRPLEEGTSLPRPPRVNQRRERKKRSSCGGCEPCLRKINCG
Shark (XP_007890120.1)           TSAQPQVPTEMTVERLVTQ---NYSVSPAFKTIHTFKTEGQLQQPQHNLHSAPVNKKQEPQSYAGQVPSQRYSAVSMNCLQIPNSIPTTM
Human (ENSP00000425443)          NSELPPKPAAVVSEACDADDADNASKLAAMLN--TCSFQKP-EQLQQQKSVFEICPS-PAEN-NIQGTTKLASGEEFCSGSSSNLQAPGG
Gorilla (ENSGGOP00000034525)     NSELPPKPAAVVTEACDADDADNASKPAAMLN--TCSFQKP-EQLQQQKSVFEICPS-PAEN-NIQGTTKLASGEEFCSGSSSNLQAPGG
Mouse (ENSMUSP00000143029)       SLQLPPEPAAMVTKACDAD---NASKPAIVPG--TCPFQKAEHQQ---KSALDIGPS-RAENKTIQGSMELFA-EEYYPSSDRNLQASHG
Rat (ENSRNOP00000048956)         SAQLPPEPAAMVTKACDLD---SASKPAGVLG--TCAFQKAEHQQ---KSVLGIGPSSCAENINIQGNTKPFA-EEYYAGSDSDLQTSLG
```

```
Pig (ENSSSCP00000009765)               NSELPPEPAAVVTEACDAD---SASKPAAILG--TCPFQKP-EQQ---KAVFEMCPS-PAQNSHIQGTIKLVSGEEFCSGSGSNVQAPGG
Tasmanian_devil (ENSSHAT000000         NSELPQVPAAVVPEACNAD---NSSVPATMPD--SYPLQKPEKQVQQQKSIFEISPS-PVESRNHQGTPELVPGEEFCLGSDNNLQIPGD
Whale (XP_007106564.2)                 NSELPPEPAAVVTEACDAG---SASKPAAMLG--TCPFQKP-EQR---KSVFEICPS-PAENSNIQGTTKLVSGEEFCSGSSSNLQAPGG
Elephant (XP_023404313.1)              NSEQPPAPVAVVTEACDAD---NASKPPAMLG--TCPFQKP-EQLRPQKSVFEICPS-PAENSNNRGTTKLESGEEFCSGSSSNLQAPGG
Lizard (ENSACAP00000008438)            ------------------------------------------------------------------------------------------
Chicken (ENSGALP00000017216)           NSELPQVPAVVVTEVYGAD---DSSKPPVLPG--SCSLQKPELQLQQQIPGYDTHRL-PLGNSAVHGSVGQVPNQDLSLSSSSNLQAQNA
Turtle (ENSPSIP00000011691)            NSELPQVPAVVATEVYNMG---DSSKPPVLPG--SYSLQKS--ELQQQNTGYDTHQL-AIGNRSVQRHMGQLPNQDLALSSSSNLQARNN
Xenopus (ENSXETP00000030770)           --VLPQVSGAIVDGAPNSD---HGSEPLVLPG--SYSLNRQ-------------------EQKPLSQETLNYNSHNLVGSMGDACKSMGQ
Coelacanth (ENSLACP00000008576         TSTLPQVPAAMAIDIHSSQ----NSRTSAVTGGCSYQFQECHQQQNHEQQVCNLQKLPVENNLHLQSSLGQVPNENILITSQSSLQKQN-
Amazon_molly (ENSPFOP000000120         -ESQ-QQPPAASA-----EGVNSYNSVNYLVN--GYSSNFGVNQQQQPS------------------SCSGQEI---VLG----AQQHPS
Amazon_molly (ENSPFOP000000299         -ESQ-QQPPAASA-----EGVNSYNSVNYLVN--GYSSNFGVNQQQQPS------------------SCSGQEI---VLG----AQQHPS
Fugu (XP_011611191.1)                  NSQKLQLRASGNN-----EGGNNYDSANYIMN--GYSKEFQADRQHQEQQFQQQLSSYS-EQKMSTGQLPGMFP---SRNVGNGSPQHQN
Medaka (XP_004065962.1)                NSQQQQPRAPENS-----ERGNSCN--SYLVN--GFSSSFGADHQQQQQPHAYS--------ALEMRKLSGEIT---PTNDTNCSQEHPT
Platyfish (ENSXMAP00000005447)         -ESQHQQPPATSA-----EGVNSYNSGNYLVN--GYSSNFGVNQQQQLS------------------SCSGQEI---VLG----AQQHPS
Spotted _gar (ENSLOCP000000155         VSVQLDTISPEIH-------TKSIYSPRFGTD--GYSGAFQGKHQKQPQQESYSLQDVPGKDEQHLQSQEGQVPSIVLPARPDGSLQDQI
Stickleback (ENSGACP0000002318         ------------------------------------------------------------------------------------------
Tilapia (XP_005457058.1)               GSQQQQAQASGNT-----EGGNNYNSVNYVLN--GYSNNFGADHQQTQQHQQSTPS-YS-GEELALGQLSGMIL---STNNAKIPQQHPN
Tetraodon (ENSTNIP00000021201)         ------------------------------------------------------------------------------------------
Zebrafish (ENSDARP00000101295)         ASETLVKAPPEVQ-------NSNGYNPELAVN--GYS-SFAAEQKKPAYSLSDLP----------ELSQKDSESNVPSQTVVNGSSQIQE
Salmon (XP_014042431.1)                ------------------------------------------------------------------------------------------
Salmon (XP_014065146.1)                PASEKQQQTGVAAPKGQGSNGYNNSAAPYMVN--GYSNSYRGE--QQQPADGSQHQNNT-EGIYRKPNQEFNHQNSFLTPLEQSTPLQTE
Salmon (XP_014050290.1)                PASEKQQQTGVAAPKGQGSNGYNNSAAPYMVN--GYSNSYRGEHQQQQPADGSQHQNNT-EGIYRKPNQEFNHQNSFLTPLEQSTPLQTE
Rainbow trout (XP_021429026.1)         ------------------------------------------------------------------------------------------
Rainbow trout (XP_021429023.1)         PASEKHQQTGVADPKGQGSNGYNNSAAPYMVN--GYSNSYRGEHPQQQPADGSQHQNNP-EGIYRKPNQEFNHQNSFPSPLEQSTPLQTK
Shark (XP_007910136.1)                 ------------------------------------------------------------------------------------------
Human (ENSP00000386869)                PDYLEWLEGKIKS--VVMEGGEERPRLPGPLPPGEAGLPAPSTR-PLLSSE-----------------VPQISPQ-EGLPLSQSALSIAK
Gorilla (ENSGGOP00000051172)           PDYLEWLEGKIKS--VVMEGGEERPRLPGPLPPGEAGLPAPSTR-PLLSSE-----------------VPQISPQ-EGLPLSQSALSIAK
Armadillo (ENSDNOP00000021516)         PDYLEWLEGKIKSAAVVVEGGEEPPRPPGTLPPGEAGLPTPSAR-PLLGGE-----------------VPRVPPP-EGLPLSQSALSIAK
Mouse (ENSMUSP00000087049)             PDFLEWLEGKIKS--MAMEGGQGRPRLPGALPPSEAGLPAPSTRPPLLSSE-----------------VPQVPPL-EGLPLSQSALSIAK
Rat (ENSRNOP00000015296)Tet3           PDYLEWLEGKIKS--AAMEGGQGRPRLPGTLPPSEAGLPAPSTR-PLLSSE-----------------VPQVPPL-EGLPLSQSALSIAK
Pig (ENSSSCP00000008852)               PDYLEWLEGKIKS--VVVEGGEERPRLPGTLPSGEAGLPAPISR-PLLSSE-----------------VPQIPPP-EGLPLSQSALSIAK
Elephant  (XP_010591957.2)              PDYLEWLEGKIKS--VVVEGGEERPRLPGTLPSGEAGLPAPSTR-PLLNAE-----------------VPQIPPP-EGLPLSQSALSIAK
Whale (XP_023989264.1)                 PDYLEWLEGKIKS--VVVEGGEERPRLPGTLPSGEAGLLAPITR-PLLSSE-----------------VPHIPPPPEGLPLSQSALSIAK
Tasmanian_devil (ENSSHAP000000         PDYLEWLEGKIKS--VAVGGGEDRSRPPESLPPTEVGLPALGPG-TLLSPD-----------------APQLHSP-NGLPLSESALNIAK
Chicken (ENSGALT00000059756)           PDYLEWLEQKIQAAL-GEGPLP----------PAPPPPDKGAI-APPAVLE-----------------AAEP-CPSDGLPFSQSALNIAK
Lizard (ENSACAT00000001585)            PDYLEWLERKIRLAG-GRQPLPQPQPLCPAPLTTPAPFSNGAT-NLEAAPE-----------------EKPPRCPLEALPLSQSALSIAK
Turtle (ENSPSIT00000006549)            PPGPQDQLPKWWAP--GSQALP------------PKPSEKQAK-EKKKKIQ-----------------PDKP---PQVKPLRK-QVQIKK
Xenopus (ENSXETP00000054061)           PDYLEWLENKIKSTVKGSQESPFPNLG----QVSKELVQKQYPKEQVLNLE-----------------NKNSTCPSGNLPFSQNALSLAK
Coelacanth (ENSLACP00000009535         PDYLEWLEKKIRSAA-GKEQKRIPHQP---TKMNSQPLVNGAFPNQVPAVE-----------------ETQTLNSFDALPYSQSVLSIAK
Spotted_gar (ENSLOCP0000001981         PDYLEWLEKKIKLAT-----GDEQQCSAPNTSPQPQPLNRASQCPLP-------LSASTPVPPDTVNAEDQAVHPEAPLPYSPSVLSIAK
Amazon_molly (ENSPFOP000000021         PDYLEWLEKKIKLA------TRRDQGSHKMGDEAPHLEQPHLPHQH--------QMYQQQQEVNQYSHQDQVPSSKPPIPCSPQVLSIAK
Cave_fish (ENSAMXP00000015689)         PDYLEWLEKKIKTA---ANKQDEGLCKMSGAPSVAESQLQN--------------------PVNQPPGPRPYGPRP-PIQCTPSVLSIAK
Fugu (ENSTRUP00000036865)              PDYLDWLKKKIKLN------HPQQYPQLNGGQRLPTSYPQQQQGSH--------LKQAT--------------CSKSPIPCSPQVLSIAK
Medaka (ENSORLP00000019236)            ------------------------------------------------------------------------------------------
Platyfish (ENSXMAT00000000130)         ------------------------------------------------------------------------------------------
Stickleback (ENSGACP0000001732         ------------------------------------------------------------------------------------------
Tetraodon (ENSTNIP00000019883)         ------------------------------------------------------------------------------------------
Tilapia (ENSONIP00000019943)           PDYLEWLEKKIKLA------TNEDQGSRKLVDTPSHLQP-HIEQPH--------LLHHSQTYQ------------QPPIPCSPQVLSIAK
Zebrafish (ENSDARP00000085229)         ------------------------------------------------------------------------------------------
Rainbow_trout (XP_021462005.1)         PDYLEWLEKRLKQAGAGEEQDNNTPCSKMAADTPPHQQPPHSQQPPHPHHHPHPHVNGGNPPSCPPLHPHQQGQRPGSVPCSPQVLSIAK
Rainbow_trout (XP_021477599.1)         PDYLEWLEKRIKQTWAGEERDNTTPCSKMTTDTPPHQQPPHSQQPP----HPQPHVNGGNPPSGPPLHPHQQGQHPAPIPCSPQVLFIAK
Salmon (XP_014026412.1)                PDYLEWLEKRIKQA--GEEQDNNTPCSKMAADTPPHQQPPHSQQPPHPHHHPHPHVNGGNPPSCPPLHPHQQGQRPGSVPCSPQVLSIAK
Salmon (XP_014017100.1)                PDYLEWLEKRIKQTGAGEGRDNNMPCSKMTADTPPHQQPPHSQQPP----HPQPHVNGGNPPSCPPLRPHQQGQHLAPIPCSPQVLSIAK
```

```
                                                910       920       930       940       950       960       970       980       990
                                       ....|....|....|....|....|....|....|....|....|....|....|....|....|....|....|....|....|....|
European ampioxus (Sc0000095)          ------------------------------------------------------------------------------------------
Ciona intestinalis (XP_0021254         GQSIGSGNNRSPMMQSSPAGVVSHSPFAG--------------------------------SAGSSLRSPMSNLMGNSPANMPQGQTTSP
Lamprey (scaf_00044)                   ------------------------------------------------------------------------------------------
Lamprey (scaf_03335)                   ------------------------------------------------------------------------------------------
Shark (XP_007897316.1)                 EYFCNSTHPLNNTIKLNNDLSTLVSVVS----------------YAQSESGRSLSVEKESTSCNCEGLDCPDYQNVVDESAKTMCEEGRT
Lizard (XP_020663651.1)                ECSCCRKRKTSHRICKKRKCEELKKPPPPLGLMLPSEVLTENKRPQR-RKQRVLKGDVENKPVNGYKPDLMQCVVYSHGEKHKVKP---L
Chicken (ENSGALP00000053457)           ECSCCRKRKTSHRICKKRKCEELKKPPPP--ITLPFEVLTENKRPQR-RKQRVFKANLENKPVNGRRPELMECSICGHGEKYRVKTNQTQ
Turtle (ENSPSIP00000006704)            ECSCCRKRKTSHRICKKRKCEELKKPPPP--IMLPFEVLTENKRPQR-RKQRVLKADLENKPVNGLRPELMECSICGHGEKYRVKTNQTL
Human (ENSP00000362748)                ECTYCKNRKNSHQICKKRKCEELKKKPS---VVVPLEVIKENKRPQREKKPKVLKADFDNKPVNGPKSESMDYSRCGHGEE-QKLELNPH
Gorilla (ENSGGOP00000011536)           ECTYCKNRKNSHQICKKRKCEELKKKPS---VAVPLEVIKENKRPQREKKPKVLKADFDNKPVNGPKSESMDYSRCGHGEE-QKLELNPH
Mouse (ENSMUSP00000133279)             ECTYCKNRKNSHQICKKRKCEVLKKKPE---ATSQAQVTKENKRPQREKKPKVLKTDFNNKPVNGPKSESMDCSRRGHGEEEQRLDLITH
Rat (ENSRNOP00000054747)               ECTYCKNRKNSHQICKKRKCEVLKKKPE---ATSRVQVTKENKRPQREKKPKVLKTDFNNKPVNGPKSESMDYSRRGHGEEEQRLGLFTH
Pig (ENSSSCP00000010918)               ECTYCKNRKNSHQICKKRKCEELKKKPS---IIVP------------------LEADFDNKPVNGPKSESMEYSSCGHEEE-QRLELSVF
Whale (XP_023987950.1)                 ECTYCKNRKNSHQICKKRKCEELKKKPS---IIVPLEVIKENKRPQREKKPKVLKADFDNKPVNGPKSESMEYSSCGHEEE-KRLELNSC
Elephant (ENSLAFP00000005179)          ECTYCKNRKNSHQICKKRKCEELKKKPS---IVVPLEVIKENKRPQREKKPKVLKAGFDNKPVNGPKSESMEYSRCGHGEE-QRLELNPH
Armadillo (ENSDNOP00000014565)         ECTYCKNRKNSHQICKKRKCEELKKKPS---VIVPLEVIKENKRPQREKKPKVLKADFGNKPVNGPKSESMEYRRCGHGEE-QRLESNPH
Tasmanian_devil (XP_023360087.         ACSQNADRQVGS-----------------------DKVIKENKRPQREKKSKVFKIHLDNNLVNGPRSESMENSKCSNGEKQRLKLN-QN
Coelacanth (ENSLACP00000016889         DESS-STHLTN---TDNLDAVNTLTTVC----------------YAPSEKQKSHNVVVNSTDCSHQGLQHPDYS-YLHNNAENLTQQS-N
Cavefish(XP_007240334.2)               QCSCCLNRKTGHQICKLRKCVMLKKR----PWQVTAGEVVCKNIPKSRKRNRVSKVDSEGILVNGTNSEQMEETCVVPEEEARDVSQAHS
Platyfish (XP_014325204.1)             QCSCCLNRRTGHQICKLRKCVELKRRRSSSVISQRDAQV-----------------APEIGSVNGKGKEQMDNTYMAAEEDEEEDGHISR
Tilapia (XP_005456583.2)               QCSCCLNRRTGHQICKLRKCVELKRRRPSSPHTHSAAQV-----------------VPESGLVNGTGKAQMDDTHMAAEEDEGEEGHIPP
Spotted_gar (XP_015202470.1)           ECSCCRNRKTGHQICKLRKCMELRKK----PSLLSSSQVGSKDTTKPQRKTRVSKVESYGHSVNGTKSSQMEESFSMQEEDLALQIYPIS
Stickleback (ENSGACT0000001035         ------------------------------------------------------------------------------------------
Tetraodon (ENSTNIT00000011701)         ------------------------------------------------------------------------------------------
Fugu (XP_011601624.1)                  QCSCCLKRSTGHQICKLRKCVQLKRRPLSSPFALPAAQV-----------------APESGSVDGKGKEQMDDTHAAAEEDEGEEGHIYR
Zebrafish (ENSDARP00000143362)         ECRSCLNQKTDHQICRLRRCIKLRKK----TLIICTG-----------------KVESEANSVNGTSSEQMEETCAAPEEEAHYIPVTHN
Amazon_molly (XP_016534180.1)          QCSCCLNRRTGHQICKLRKCVELKRRRSSSVISQRDAQV-----------------APEIGSINGKGKEQMDDTYMAAEEDEEEDDHIPH
```

```
Medaka (XP_023818916.1)              LCSCCLNRRTCHQICKLRKCVELKRRKPSSSPGFSAKEV-----------------IPESGSVNGKSKAQMEDTHMAAEEDEGEEGHIPH
Rainbow_trout (XP_021469805.1)       QCSCCLNRRTGHQICKLRKCMELRKRRPMSLLTLTSTQVGCKEVAKPALKKPVSTVVPEDSSVNGRGPVQMAEDTYTAAED-DEEGHIPH
Rainbow trout (XP_021436521.1)       QCSCCLNRRTGHQICKLRKCMELRKRRPMSLLTLTSAQVACKDVAKGALKKAVSTVVPEGVAINGRSPVQMVKETYTAEEECHIPPTHPQ
Salmon (XP_014010485)                QCSCCMNRRTGHQICKLRKCMELRKRRPMSPLTLTSTQVGCKDVAKPALKKPVSTVVPEDSSVNGRGPVQMAEDTYTAAED-DEEGHIPH
Salmon (XP_014070638.1)              QCSCCLNRRTGHQICKLRKCMELRKRRPMSLLALTSAQVACKDVAKGALKKPVSTVVPEGVAINGRSPVQMVEETYTAEEEGHIPPTHPQ
Shark (XP_007890120.1)               CSTQMPETTDLFYSRNKTNYINDLGAISRSESPAQS-----------AATVTLNSNQTNASTTEHCKRINNITREPDLSDVRRSSDQTNH
Human (ENSP00000425443)              SSERYLKQNEMNGAYFKQSSVFTKDSFSATTTPPPP--------SQLLLSPPPPLPQVPQLPSEGKSTLNGGVLEEHHHYPNQSNTTLLR
Gorilla (ENSGGOP00000034525)         SSERYLKQNEMNGAYFKQSSVFTKDSFSATTTPPPP--------SQLLLSPPPPLPQVPQLPSEGKSTLNGGVLEEHHHYPNQSNTTLLR
Mouse (ENSMUSP00000143029)           SSEQYSKQKETNGAYFRQSSKFPKDSISPTTVTPPS-------------QSLLAPRLVLQPPLEGKGALNDVALEEHHDYPNRSNRTLLR
Rat (ENSRNOP00000048956)             SSEQCIKQKETNGAYFRQSSVFPEDSISATTMTPPS-------------PSPLAPRPVLQLPSEGKGTLNDVALEEHHRYPNQSDLTLSR
Pig (ENSSSCP00000009765)             SSERYLKQNEMNGAYFKQSSVFTKDSFSATTTPPP---------SQLLLSPPPPLPQVPQLPSEGKSTLSDGGLEGHHHYPNQSNTVLLK
Tasmanian_devil (ENSSHAT000000       NPDGYTKQTEMSGAYLRQNSVFAKDSFIATTPPPPPQSSPLPFLLPLLPPPPVSPPPVPQPPSEMESTLHDEVTEKPHHYPSQSEESLLR
Whale (XP_007106564.2)               SSERYLKQNEMNGAYFKQSSVFTKDSFSATTTPPP---------SQLLLSPPPPLPQVPQLPSEGKSTLNDGVLEEHHHYPNQSNTALLR
Elephant (XP_023404313.1)            SSEQYLKQNEINGAYFKQCSVFTKDSFSATTTPPP---------SQLLLSTRPPLPRISQLPSEGKSTLDDGVLEEHRHYPNQSNTALLG
Lizard (ENSACAP00000008438)          ------------------------------------------------------------------------------------------
Chicken (ENSGALP00000017216)         ASERFSEQAEKNGAFFTQNSMFHKDSS-----------------------TPP--------APEMNSALSVMVREGCHSYDNRCNETLPG
Turtle (ENSPSIP00000011691)          SLE--TEQAEKNGAFFKHNSMFSKDSF-----------------------TPPPASAPPPPDPEMNSTLSIGVTEGHHPCDHRNVENLPR
Xenopus (ENSXETP00000030770)         MSAQCLDVSG-NGSFHIQNSSSNEIAS----------------------------------ELEAIKKDPAPSLQLHHIMPNKQN-----
Coelacanth (ENSLACP00000008576       STECFSNQPEANNVFFKKNSNFNEGSVVS--------------------------------TLESPRTLHIGVRDRLTPYQNINSEALSR
Amazon_molly (ENSPFOP000000120       GAQCFPDNTNPQGTYTKTQQEYNPRSFLN---------------------------------HNNPSLAAEAG--GYGSLLNSGANENSQ
Amazon_molly (ENSPFOP000000299       GAQCFPDNTNPQGTYTKTQQEYNPRSFLN---------------------------------HNNPSQAAEAG--GYGSLLNSGANENSQ
Fugu (XP_011611191.1)                GPQHYPDDTNPQLIYPEMNQEFLEG-------------------------------------NAKLQPPVTGGYKSYDSSKLQKMGQKEE
Medaka (XP_004065962.1)              SIPHFTDQSDPQSAYKNTQQEFDHSPFLR---------------------------------S-SNAQHAIDENGRYRCFANSGMAQNEK
Platyfish (ENSXMAP00000005447)        GAQCFPDNTNPQGKYTKTQQEYNPRSFLN---------------------------------HNNPSQAAEAG--GYGSLLITGANENSQ
Spotted _gar (ENSLOCP000000155       HVECFSDNVETSDTYVKSNKDFSQEPYLS---------------------------------RMEPKFPLEEGETDRLIHFNDKAMERTL
Stickleback (ENSGACP0000002318       ------------------------------------------------------------------------------------------
Tilapia (XP_005457058.1)             GAQCYQDGTRHQGIYVKAHQEFNGSSFPN---------------------------------CNSSLQSAEAEYESFPSSGTQKQGQSEE
Tetraodon (ENSTNIP00000021201)       ------------------------------------------------------------------------------------------
Zebrafish (ENSDARP00000101295)       DREALPNDSECVNMFSKSNQNLSHGSYTL---------------------------------PPVTAGSPSQTSQTGQYNPQIKVPQKKT
Salmon (XP_014042431.1)              ------------------------------------------------------------------------------------------
Salmon (XP_014065146.1)              EDIVFSPGPFPSPGMGQSEGRRRQQQYGM---------------------------------QQQQQQTGNPCGADSTGPQGPMGPASLP
Salmon (XP_014050290.1)              EDIVFSPGPFPSPGMGQSEGRRRQQQYGM---------------------------------QQQQ--TGNPCGADSTGPQGSMGPASLP
Rainbow trout (XP_021429026.1)       ------------------------------------------------------------------------------------------
Rainbow trout (XP_021429023.1)       EAIGFGPGPFPSTGMGQSEGRRRQQQYGM---------------------------------QQQQQQTGNPCGADSTGPQGSMGPASLP
Shark (XP_007910136.1)               ------------------------------------------------------------------------------------------
Human (ENSP00000386869)              EKNISLQTAIAIEALTQLSSALPQPSHSTPQASCPLPEALS---------------------------PPAPFRSPQSYLRAPSWPVVPP
Gorilla (ENSGGOP00000051172)         EKNISLQTAIAIEALTQLSSALPQPSHSTPQASCPLPEALS---------------------------PPAPFRSPQSYLRAPSWPVVPP
Armadillo (ENSDNOP00000021516)       EKNISLQTAIAIEALTQLSSALPQPAHPASQAPCPLPEALS---------------------------PPAPFRSAQSYLRTPPWPAVPA
Mouse (ENSMUSP00000087049)           EKNISLQTAIAIEALTQLSSALPQPSHSTSQASCPLPEALS---------------------------PSAPFRSPQSYLRAPSWPVVPP
Rat (ENSRNOP00000015296)Tet3         EKNISLQTAIAIEALTQLSSALPQPSHSTSQASCPLPEALS---------------------------PSAPFRSPQSYLRAPSWPVVPP
Pig (ENSSSCP00000008852)             EKNISLQTAIAIEALTQLSSALPQPSHATSQAPCPLPEALS---------------------------PPAPFRSPQSYLRAPSWPVVPP
Elephant  (XP_010591957.2)           EKNISLQTAIAIEALTQLSSALPQPSHSTSQAAGPLPEALS---------------------------PPAPFRSPQSYLRAPSWPVAPP
Whale (XP_023989264.1)               EKNISLQTAIAIEALTQLSSALPQPSHATSQASCPLPEALS---------------------------PPAPFRSPQSYLRAPSWPVVPP
Tasmanian_devil (ENSSHAP000000       EKNISLQTAIAIEALTQLSSTLPQP------SPCPQSKALS---------------------------PNPAFRPPQSYLRTSPWPVGPP
Chicken (ENSGALT00000059756)         EKNISLQTAIAIEALTQLSAALPQPGADGP-AVPP----SG-------------------------------------------------
Lizard (ENSACAT00000001585)          EKNISLQTAIAIEALTQLSAVLPEAAPKTP-PFPAPRPNSGP---------------------PLSSPPSSWSPGAKATQDSGRYRLDPC
Turtle (ENSPSIT00000006549)          SKQKDSQPLFLPLRQISLEGFHSAPAPELPT-----------------------------------------------------------
Xenopus (ENSXETP00000054061)         EKNISLQTAIAIEALTQLSSALPQTNNECPNAPSQPLINPHD---------------------QLTHFPSAKGNQLPMLPVARNELFQNQ
Coelacanth (ENSLACP00000009535       EKNISLQTAIAIEALTQLSSTFPQPLADVSATQNG-----------------------------------------TLQVELFRSKDQMV
Spotted_gar (ENSLOCP0000001981       EKNISLQTAIAIEALTQLSSTVPQTVVP-------------------------------PGESSSANPSNIQPHQTMETPTCFPIRPSSL
Amazon_molly (ENSPFOP000000021       EKNISLQTALAIDALTQLSSPQTVTAPGQGFRNNFHNHQNTP--------------SLIPSSLNDNSSSRSQSVPPGLHTN--QQGAASY
Cave_fish (ENSAMXP00000015689)       ERNVSLQTAIAIEALTQLSGTTPGPLS------------------------------------SASPSPMAQSGPPGHFPQ--QDPASWD
Fugu (ENSTRUP00000036865)            EKNISLQAAIAIEALTQLSGTGPQAPGSPGHAPFNSNFHHQHRTQNLLSCPPNGNSSILSPPGSQSSSSRSQSVPPGSHSS--HQTPVSS
Medaka (ENSORLP00000019236)          ------------------------------------------------------------------------------------------
Platyfish (ENSXMAT00000000130)       ------------------------------------------------------------------------------------------
Stickleback (ENSGACP0000001732       ------------------------------------------------------------------------------------------
Tetraodon (ENSTNIP00000019883)       ------------------------------------------------------------------------------------------
Tilapia (ENSONIP00000019943)         EKNISLQTAIAIDALTQLSG--------------------------------------TSPQSTYSSSSRSQSVPPGLHTS--QQALESC
Zebrafish (ENSDARP00000085229)       ------------------------------------------------------------------------------------------
Rainbow_trout (XP_021462005.1)       EKNVSLQTAIAIEALTQLSATAPQTVVPPAQASSTSHQ-----QPPLHPQHGNHLLPSSPGPMSSLSSSRSQSVPPGLYPQ--QSSGSWE
Rainbow_trout (XP_021477599.1)       EKNVSLQTAIAIEALTQLSATVPQTVVPPAQASSTSQP----QHPPLHLQHGTRLNPSSSNPISSLSSSRSQSVPPGLYPQPQTSPVSRE
Salmon (XP_014026412.1)              EKNVSLQTAIAIEALTQLSATTPQTVVPPAQASSTSHQ-----QPPLQPQHGNRLLPPSPGPMSSLSSSRSQSVPPGLYPQ--QSSSSWE
Salmon (XP_014017100.1)              EKNVSLQTAIAIEALTQLSATVPQTVVPPAQASSTSQPQHHPQHPPPHLQHGTRLVPSSPNPLSSLSSSRSQSVPPGLYPQPQQSPVSWE

                                              1000      1010      1020      1030      1040      1050      1060      1070      1080
                                     ....|....|....|....|....|....|....|....|....|....|....|....|....|....|....|....|....|....|
European ampioxus (Sc0000095)        ------------------------------------------------------------------------------------------
Ciona intestinalis (XP_0021254       SQVSMPHLASSPITSNVAAPQIPHPSGSNPSGGSS-------------------------------------------------------
Lamprey (scaf_00044)                 ------------------------------------------------------------------------------------------
Lamprey (scaf_03335)                 ------------------------------------------------------------------------------------------
Shark (XP_007897316.1)               ASPFLRKDT-------CVENGALHKSS-----TLYEYSPSILSMVKKKN-----------------------------------------
Lizard (XP_020663651.1)              PWENVQSNEKGSMTGVETDKWAHSEKPSLSI-HVNGDLCKAGTRDENSKHLEKNAKAIHSSS----------------------------
Chicken (ENSGALP00000053457)         PFENAQSKEKERMTGLEAEKWAHNEKPSFGV-HVNGDTHGTVTGHEHLRNAEESERAVSPSQ----------------------------
Turtle (ENSPSIP00000006704)          PFENVQCNEKESMTGLEAEKWTDKEKPSFNV-YVNGNIHETVTDNEHLKNSED-ERAVSLSE----------------------------
Human (ENSP00000362748)              TVENVTKNE-DSMTGIEVEKWTQNKKSQLTD-HVKGDFSANVPEAEKSKNSEVDKKRT--------------------------------
Gorilla (ENSGGOP00000011536)         TVENVTKNE-DSMTGIEVEKWTQNKKSQLTD-HVKGDFSANVPEAEKSKNSEVDKKRT--------------------------------
Mouse (ENSMUSP00000133279)           PLENVRKNA-GGMTGIEVEKWAPNKKSHLAEGQVKGSCDANLTGVENPQPSEDDKQQT--------------------------------
Rat (ENSRNOP00000054747)             PLENVRKNA-GGMTGIEVEKWTPNKKSHLAAGQVKGSRGANLAGVENSQPSEDDKQQT--------------------------------
Pig (ENSSSCP00000010918)             PFEHVTKNE-ESMTGIEVEKWTQNKKSNLSD-HVKGEFNAIITEAENRKNSEDDRKKVLLT-----------------------------
Whale (XP_023987950.1)               PLENVTKNE-ESMTGIEVEKWTQNKKSHLTD-HVKGEFNTIKTEAENLKSSEDDRKKILLT-----------------------------
Elephant (ENSLAFP00000005179)        PLENVTRNE-ESMTGIEIEKWTQNKKSHLTD-HVKGDFSATVPEAEKLKNSEDDRKKVPLT-----------------------------
Armadillo (ENSDNOP00000014565)       PLENVTKNE-ESGTGIEVEKWTQNKKSHLTD-YVRGDSGAIVTEAEKSKNSEDDRKKVLLT-----------------------------
Tasmanian_devil (XP_023360087.       ILENVQTNEEDTMTGVEVEKWAENKKSHLTI-HVQGDISGPGPETENLKKSEDHEKKTLLSD----------------------------
Coelacanth (ENSLACP00000016889       SICFSKKDT-------GSEESAINVPKSDGCISSLLFQPSLLSFMKNRN-----------------------------------------
Cavefish(XP_007240334.2)             APPDSPTP-------LGHDTEPAPAERALQPAVSHHALLTHQSSLSS---HNGANH----------------------------MEPNTE
Platyfish (XP_014325204.1)           TQFPVIVQSFTN-----HGGQPALIKRESEMDLTNSA-LHRHICSSV-THQNGSDGSFTKPN-----------GANMTNGSQSDLKSACK
Tilapia (XP_005456583.2)             TQSPPITHSLTHDLALDQGVRPTLIKGEPGLELTSTAPLHQQVCSSV-TFQNGSAANLIKPKP----------GANMTT------VSALK
Spotted_gar (XP_015202470.1)         PVVK-----------EEQDAHPILEKEQVG----DSAQQPSHLCDSGNNPENWNHN------------------------------GERK
```

```
Stickleback (ENSGACT0000001035   ------------------------------------------------------------------------------------------
Tetraodon (ENSTNIT00000011701)   ------------------------------------------------------------------------------------------
Fugu (XP_011601624.1)            ARSPSITNSLTHNLAPEQGGLPALVKGEPGLELTNVA-LHQHVCSSL-TLENGSTESQTKPN-----------GANMTTGSHADPKSVYK
Zebrafish (ENSDARP00000143362)   VPPDIITP-------LQHGLLPEPHGKDPG---TTDVLLPHSFSSTS---HNGAEH----------------------------IKSNAQ
Amazon_molly (XP_016534180.1)    TQSPVIVQPFTN-----HSGQPALIKRESEMDLTNSA-LHRHICSSV-THQNGSDGSLTKPN-----------GANMTNGSQSDLKSACK
Medaka (XP_023818916.1)          TQTTLVSQSLVHNLALDLGGRAALIKSEAGLERTSFA-LHRCLSSFS-NVPNGSVENPLRAN-----------GANLTRGP--DLKSTCK
Rainbow_trout (XP_021469805.1)   THHFTHPPHVHTLAPIRDGRPAALIKREPGLEDTNDGLLLQHHCSSSGTFHNGANENMAKTNGANENMAKTNGAQLIAGPESHQLSPESQ
Rainbow trout (XP_021436521.1)   THPHTHPPHAHTLTPILDGRPAVLIKREPGLEDTNDALLLQHHYSSS--FHNGADENLSKTN-------------------GAPVSPDSQ
Salmon (XP_014010485)            THHFTHPPHVHTLAPIRDGRPAALIKREPGLEDTNDGLLLQHHCSSSGTFHNGANENMAKTN----------GAQLIAGPDSHPVSPESQ
Salmon (XP_014070638.1)          TDPHTHPPHAHTLTPILDGRPAVLIKREPGLEDTNDALLLQHHYSSSGTFHNGADENLSKAN-------------------SAPVSPESQ
Shark (XP_007890120.1)           TESVKPENTQVEIPCKQEPEQPDMTRNCMIASSPNITQQHCAEAAVRHPIQQSIEPPAHNQTLI--------------------------
Human (ENSP00000425443)          EVKIEGKP-EAPPSQSPNPSTHVCSPSPMLSERPQ--NNCVNRNDIQT-AGTMTVPLCSEKTRP--------------------------
Gorilla (ENSGGOP00000034525)     EVKIEGKP-EAPPSQSPNPSTHVCSPSPMLSERPQ--NNCVNRKDIQT-AGTMTVPLCSEKTRP--------------------------
Mouse (ENSMUSP00000143029)       EGKIDHQP-KTSSSQSLNPSVHTPNPPLMLPEQHQ--NDCG--------------SPSPEKSRK--------------------------
Rat (ENSRNOP00000048956)         EGKTEHQPKKTTSSQSLNTSVHTPNPPLMLPEQHQ--NDCV--------------PPCSEKTRK--------------------------
Pig (ENSSSCP00000009765)         EMKIESQP-EAPPSQTPNPSTHVSNPPLMLPEKPQ--NNYVNKSDIRT-PGTMTVPLSSEKTRQ--------------------------
Tasmanian_devil (ENSSHAT000000   EMKNEGMS-EGPMSQNPDLSTCATK-SLMFPEKPQ--NNCVHQNEISASIMTLAVPLCSEGKKP--------------------------
Whale (XP_007106564.2)           EVKIEGQH-EAPPSQSPTLSTQVSNPSPMLPERPQ--NYCVNKNDIRT-PGTVTVPLCSEKTRQ--------------------------
Elephant (XP_023404313.1)        EVKIERPP-EAPPSQSPNPSTHASSPSLMLPDRPQ--NNCVNKNDIRT-PGTTGIPLCSEKTRQ--------------------------
Lizard (ENSACAP00000008438)      ------------------------------------------------------------------------------------------
Chicken (ENSGALP00000017216)     EIKNEGQH-QGPMPESPGLSQQQLHPQQRLPQQVQTSQHKVSHSDPRAAAAASIQQHPEEMPPP--------------------------
Turtle (ENSPSIP00000011691)      EVKNEGQQ-EGRVTKSPGLAQQQPKPQPKLPQKPEVSQQDGGESDLPTATMTSIQQCTEETKP---------------------------
Xenopus (ENSXETP00000030770)      ESSISQSHDGHPIASQTNESCHSHDPRVSPHPNDQ----QLQNERTAMFPLTRLPALHSSVNEI--------------------------
Coelacanth (ENSLACP00000008576   AVASETSS-NQTMPQSPSIVAQPENHQHKLSHPHEFQKQNLTENYGVN-STNAALELSPEEMKE--------------------------
Amazon_molly (ENSPFOP000000120   ND-------------NGAGLYHDTNMGIQYGTQAQ-------------------------------------------------------
Amazon_molly (ENSPFOP000000299   ND-------------NGAGLYHDTNMGIQYGTQAQ-------------------------------------------------------
Fugu (XP_011611191.1)            PS-------------NGQ--HHSMGRGQQYGIQTQ-------------------------------------------------------
Medaka (XP_004065962.1)          SG-------------SDQFLHLSTERSQ-CGIQPQ-------------------------------------------------------
Platyfish (ENSXMAP00000005447)   ND-------------NGAGLYHDTSMGLQYGTQGQ-------------------------------------------------------
Spotted _gar (ENSLOCP000000155   KSKESETRQQELLFESQKHFDHPTAGGTSQSPAPE-------------------------------------------------------
Stickleback (ENSGACP0000002318   ------------------------------------------------------------------------------------------
Tilapia (XP_005457058.1)         NT-------------SSQPMQHGTDRDRHYGIQPQ-------------------------------------------------------
Tetraodon (ENSTNIP00000021201)   ------------------------------------------------------------------------------------------
Zebrafish (ENSDARP00000101295)   TD-------------ENLHGNRDFSLLQQQTHEPQ-------------------------------------------------------
Salmon (XP_014042431.1)          ------------------------------------------------------------------------------------------
Salmon (XP_014065146.1)          GG-------------SEPGPQRSHQNGMMKDTTQQ-------------------------------------------------------
Salmon (XP_014050290.1)          GG-------------SEPGPQRSHQNGMMKDTTQQ-------------------------------------------------------
Rainbow trout (XP_021429026.1)   ------------------------------------------------------------------------------------------
Rainbow trout (XP_021429023.1)   SS-------------SEPGPQRSHQNGMMKDTTQQ-------------------------------------------------------
Shark (XP_007910136.1)           ------------------------------------------------------------------------------------------
Human (ENSP00000386869)          EEHSSFAPDSSAFPPATPRTEFPEAWGTDTPP----------------------------------------------------------
Gorilla (ENSGGOP00000051172)     EEHSSFAPDSSAFPPATPRTEFPEAWGTDTPP----------------------------------------------------------
Armadillo (ENSDNOP00000021516)   EEPPSFAPDSPAFPPPTPRTEFPDAWVTDTPP----------------------------------------------------------
Mouse (ENSMUSP00000087049)       EEHPSFAPDSPAFPPATPRPEFSEAWGTDTPP----------------------------------------------------------
Rat (ENSRNOP00000015296)Tet3     EEHPSFAPDSPAFPPATPRTEFSEAWSTDTPP----------------------------------------------------------
Pig (ENSSSCP00000008852)         EEHPSFAPESPAFPPATPRTEFPEAWGTDTPP----------------------------------------------------------
Elephant  (XP_010591957.2)       EEHPSFAPDSPAFPPATPRTEFSEAWGTDTPP----------------------------------------------------------
Whale (XP_023989264.1)           EEHPSFAPDSPAFPTATPRTEFPEAWGTDTPP----------------------------------------------------------
Tasmanian_devil (ENSSHAP000000   EEPQSCAPESSAFPPVAPRTEFPEVWGPENPP----------------------------------------------------------
Chicken (ENSGALT00000059756)     ------------------------------------------------------------------------------------------
Lizard (ENSACAT00000001585)      NGHPETLGLFPNVASTPQKADFPEMWGEEEVA-----------------------------RAR--------------------------
Turtle (ENSPSIT00000006549)      ------------------------------------------------------------------------------------------
Xenopus (ENSXETP00000054061)     QSQLYTGKNALPVPQSPRQTSWEQNKKSSYQEGQYIPENLSHSSS-----VLPSDASTPQKPEF--------------------------
Coelacanth (ENSLACP00000009535   NSFPFDPNGAHALQMPTTNQALQRQSPQAYTA-----------------------------QNV--------------------------
Spotted_gar (ENSLOCP0000001981   QALQPCKHPSVSPFAHSGPSSQPLPGNTQVQWQSSCEKPRKPVNN-------------WMMLNS--------------------------
Amazon_molly (ENSPFOP000000021   EHHRPQSQGQPARASPLPSSISPFPGQGKVPGFSPNPQQWRQGSD------------------R--------------------------
Cave_fish (ENSAMXP00000015689)    -QHRPSSQGPAPHPSQYGSSSPFPNSHPSIAN--PFPQQWQQDAGGSCVKSSPRNPWMMMNSEP--------------------------
Fugu (ENSTRUP00000036865)         ELHRPQSQGQPPHVTPLPSSTSPFPGQGKHPG--PNSQHWQQGSG------------------K--------------------------
Medaka (ENSORLP00000019236)       ------------------------------------------------------------------------------------------
Platyfish (ENSXMAT00000000130)   ------------------------------------------------------------------------------------------
Stickleback (ENSGACP0000001732   ------------------------------------------------------------------------------------------
Tetraodon (ENSTNIP00000019883)   ------------------------------------------------------------------------------------------
Tilapia (ENSONIP00000019943)     EHHRPQSQGHPPHVTPIQSSTSPFPSQGKPPGFNPNPQQWQQPSG------------------R--------------------------
Zebrafish (ENSDARP00000085229)   ---------------------------------------------------------MEIGS----------------------------
Rainbow_trout (XP_021462005.1)   QQHRPQSQGHPAHASPLPSSTLLFPEQTQAGS--PHPKQWQQQQA-PGGIQEQRNRWMMMNSDS--------------------------
Rainbow_trout (XP_021477599.1)   QQHRPQSHGHPAHASHLPSSTSPFPLQPQAGSLLPHHQQWQQQQG-PGGSQEQRN--QWMNSDS--------------------------
Salmon (XP_014026412.1)          QQHRPQSQGHPAHASPLPSSTLLFPGQTQAGSPHPHPQQWQQQQA-PGGIQEQRNQWMMMNSDS--------------------------
Salmon (XP_014017100.1)          QQHRPQSHGHPAHTSPLPSSTSPFPGQPQAGSRLPHHQQWQQQQG-PGGSQEQRN--LWMNSDS--------------------------

                                         1090      1100      1110      1120      1130      1140      1150      1160      1170
                                 ....|....|....|....|....|....|....|....|....|....|....|....|....|....|....|....|....|....|
European ampioxus (Sc0000095)    ------------------------------------------------------------------------------------------
Ciona intestinalis (XP_0021254   --------DPAFQRNMQTYRPWHEQQDPNARTLGSITYQAHVTQTPAPSDAYVQKLDPMGQSSISPASNHFLAPPGSSPSLPAHKHTPNT
Lamprey (scaf_00044)             ------------------------------------------------------------------------------------------
Lamprey (scaf_03335)             ------------------------------------------------------------------------------------------
Shark (XP_007897316.1)           ----------ISIQQAIAIEALTQLSVTPPQILAEVSKPTIEIRERFVS---------SSPSVCEQKCFSSSLGQSLPMEANEPHQQQQL
Lizard (XP_020663651.1)          -----VSDESKKLFAQTVRNGIKSISYSPAECDALLKKTDLSADSSNS--CKERHCDASRGDAAPLNN-VSTLSAGTCGALLEKDHVPTQ
Chicken (ENSGALP00000053457)     -----LAE-PKKSFAQTIKNGIKTLHYSSPEAVASLKKASVEERTDLT-----SNSHAQWMKATNLNDIVSTLTTSVSCVHPDKQGNLFM
Turtle (ENSPSIP00000006704)      -----LVE-PKKLFAQTIRNGIKNINYPPSESDVSLTKVIVEERTEVTNNCRESSSPSHQSNITNLNN-VSILTTGVCCVHSEKQGNVSL
Human (ENSP00000362748)          --------KSPKLFVQTVRNGIKHVHCLPAETNVSFKKFNIEEFGKTLE----NNSYKFLKDTANHKNAMSSVATDMSCDHLKGRSNVLV
Gorilla (ENSGGOP00000011536)     --------KSPKLFVQTIRNGIKHVHCLPAETNVSFKKFNIEEFGKTLE----NNSYKFLKDTANHNNAMSSVATDMSCDHLKGRSNVLV
Mouse (ENSMUSP00000133279)       --------NPSPTFAQTIRNGMKNVHCLPTDTHLPLNKLNHEEFSKALG----NNSSKLLTDPSNCKDAMSVTTSGGECDHLKGPRNTLL
Rat (ENSRNOP00000054747)         --------NPSPTFAQTVRNGMKNVHCLPTDTDLPLNKLSHEEFSKVLG----NNSSKLLTDPSDCKDAMSDTTSREGCDHLKGPRNIVL
Pig (ENSSSCP00000010918)         -----DLLEPQKLFAQTVRNGIKNVHYLPTETNLSFKKINIEELGKAFE----NNPYKFLKDTASHNNAMSSIATSASCDHLKGRNNILV
Whale (XP_023987950.1)           -----DLLEPQKLFAQTVRNGIKNVHYLPTETNLSFKKINIEELGKAFE----NNPYKFLKDTANHNNAMSAIATSASCDHLKGRSNVLV
Elephant (ENSLAFP00000005179)    -----DLLEPQK-FAQTVRNGIKNVHYLPTEN------FNIEEFGKALE----NNSYKFLKDTANHNNAMSSIATSTSCDHLKEGSNVFV
Armadillo (ENSDNOP00000014565)   -----DFLEPQKLFAQTVRNGITNIHHLPTDTNTSFKKINIEEFGKALE----NNSYKFLKDTANFNNAMSSIVNSESCDHLKGASDAFV
Tasmanian_devil (XP_023360087.   -----LIE-PPKSFAQTVKNGIKNVYYSPAEADVSLKKVKLEKNPDLIDHDTESNPTPSLGNTTSSDNRAGALVPGVYNTHLKREENILV
```

```
Coelacanth (ENSLACP00000016889  ----------LSIEQAIAIEALTQLSATP------LENP------------------SETKASDSQVITSSLLQSGSTQN---EVQVIC
Cavefish(XP_007240334.2)        DHRDPTDLQSKSQRTVNMPTKSQTPEAIPAQT-VPLKKIKLEEPSTVSDQHSTSYLS--SSDFYQDALSTLANVVCSTISDLKGLEEKTV
Platyfish (XP_014325204.1)      KTSMASELQRTIIAAMPKDSSENGPKGSSDDVSLPLKKIKLEEPWLWITEQTTTQLTRQDDDTCEDPLSMLAAVVCLSVTERKGLEEKLL
Tilapia (XP_005456583.2)        RTTMALEPQRNIIAAVPKD-SENGPMTPPDDLSVPLKKIKQEEPWVWITEQTTTQLS-DEDEVCEDPLATLAAVVCLSVTERKGLEEKLF
Spotted_gar (XP_015202470.1)    TSCLESPPNFQESFPWHPRNSPKSKAESLLEDTVPLKKIKLEENCVGIHHQIS-LAQLESDGDYEDGLTLAAVVCYSISKRQAVSDGIT
Stickleback (ENSGACT0000001035  ------------------------------------------------------------------------------------------
Tetraodon (ENSTNIT00000011701)  ------------------------------------------------------------------------------------------
Fugu (XP_011601624.1)           RTMIASEPQRTIITKTPKDHLENGPT-SPPNMTVPLKRIKVEQPWMWITEQAPKQLC-DEDEVCEDPLSMLAAVVCLSVTERKGLEEKAF
Zebrafish (ENSDARP00000143362)  RYVDPTETPANFHSST--INNRHAPEVVQEQM-VPLKKIKLEEQQLEIDDQHSKHLE--TNGCYEDALSTLAAVVCFSSTDRKSLEEKLF
Amazon_molly (XP_016534180.1)   RTSMASELQRTIIATIPKDNSENGPKASPDDVSLPLKKIKLEEPWLWITEQTTTQLTRQDDDTCEDPLSMLAAVVCLSVTERKGLEEKLL
Medaka (XP_023818916.1)         RT---LEPER---STITKRTSENGPKTPADDMLAPLKRIKQEEPWAWITEQSAPVLR-DDDHVCEDPLSTLAAVVCLSVTERRGLEEKLF
Rainbow_trout (XP_021469805.1)  NTTIQLESQSTTKSTYPKGSSELKDALLDDSIAVPLKKIKVEEPWVWSMDQSSAPLGGDDDDVYEDALSTLAAVVCFSITDRKGLEEKLC
Rainbow trout (XP_021436521.1)  KTTRQLESQRTTKTTYPKGSSEPNDALLDDTMTVPLKKIKLEESWVWSTEQSSTLLGGNDDDVYVDALSTLAAVVCFSITDRKGLEDKLF
Salmon (XP_014010485)           NTTIQLESQSTTKSTYPKGSSELKDALLDDTIAVPLKKIKVEEPWVWSMDQSSAPLGGDDDDVYEDALSTLAAVVCFSITDRKGLEEKLC
Salmon (XP_014070638.1)         KTTRQFESQRTTKTTYPKGSSEPNDALPDDTMAVPLKKIKLEEPWVWSTDQSSTLLGGNDDDVYVDALSTLAAVVCFSITDRKGLEDKLC
Shark (XP_007890120.1)          -------HEAWLQLHRQRQIQQLRQYKQVQDAQDKCSSWGESQPQEIASLSETTEVFSHEWRGSLPLNLQHQLQWPMQISPKQDSPAQLQ
Human (ENSP00000425443)         -------MSEHLKHNPPIFGSSGELQDNCQQLMRNKEQEILKGRDKEQTRDLVPPTQHYLKPGWIELKAPRFHQAESHLKRNEASLPSIL
Gorilla (ENSGGOP00000034525)    -------MSEHLKHNPPIFGSSGELQDNCQQLMRNKEQEILKARDKEQTRDLVPPTQHYLKPGWIELKAPHFHQAESHLKRNEASLPSVL
Mouse (ENSMUSP00000143029)      -------MSEYLMYYLPNHGHSGGLQEHSQYLMGHREQEIPKDANGKQTQGSVQA-----APGWIELKAPNLHEALHQTKRKDISLHSVL
Rat (ENSRNOP00000048956)        -------VSDYRMYYLPNHGHGGDLQENSQYLMGHGEHEILKDANKGQTQGSVLA-----APGWIELKASHFHKALHQTECKATSLHSGL
Pig (ENSSSCP00000009765)        -------ITEHLKHNPPILGSSGDPQDHSQQLMGHKEQETVKNQDKEQTRDLVLQTQPYLKSGWIELKAPPYHQGESHPKCNEATLRSIL
Tasmanian_devil (ENSSHAT000000  -------ISEQPNQQPEVIESGRDLQQHYQKFIRQKDQEVSSEKERQEAKEHVLQTQSYLKPGWIDLNSSHFHQGESHPKSKEELLRMML
Whale (XP_007106564.2)          -------LSEHLKHNPPILGSSGDAQDHCQQLMGHKEQEIFKSQDKEQTRDPVLPTQPYLKPGWIELKAPHYHQAESHLKCNEATLRSIL
Elephant (XP_023404313.1)       -------ISEYLKHNSPILGSSANPQGHCQQLMGHKEQEILKGQHKEHAREHELSTQSYLKPGWIELKAPHFHQAESNPKYKEASLRSIL
Lizard (ENSACAP00000008438)     --------------------------QHFQHLYGQMNQENLADNEKDTSRSHLQS-------SWTGMTSPHFHPNESPQKSNEVLLRTML
Chicken (ENSGALP00000017216)    -------PSEPPLQNLHACGSDGELPQLCQRFPGQREPEIPPDKEKDQVKESVQQPQRYSKPAWIELVSTPFRQGELPHKPNEALLRSIL
Turtle (ENSPSIP00000011691)     -------TSEPHIQDLQVAGRHGELLRNSQLLLGQKNQATLPDKGKDQVKQPTQH---YPTPGWIELISTHFRHRDPPQKPNEALLRSIL
Xenopus (ENSXETP00000030770)    -------LAKHKFHN-----SLEMQNEHLRQIKVMQNCNGTSKDDSFIKSTKEFQSNPFLKSGWLELRPNQRLKEGSSETSCENLLNLLL
Coelacanth (ENSLACP00000008576  -------SSNQLMQALQASVSN-ELQQHYEQFQMQTQHQPLDEKQLSEANDLLQQSPHSHKQGWIELSSSRP-QGDHSKTWKNTVLKSLL
Amazon_molly (ENSPFOP000000120  ----------NFQDNSGRQ--HQPDGQIGNSLQQQNHSVSVNGVEDAHQQRVNISCSTPSQTNWIERNLSHSQQQPTQCTASQAKEQDMW
Amazon_molly (ENSPFOP000000299  ----------NFQDNSGRQ--HQPDGQIGNSLQQQNHSVSVNGVEDAHQQRVNISCSTPSQTNWIERNLSHSQQQPTQCTASQAKEQDMW
Fugu (XP_011611191.1)           ----------TLKQNSRIP-------EDRAGYIQPSPTDLENGMESNAQQRVNLSPSTPHQRGWPDLNSSRSHQQPASDQLSQVQEQDRW
Medaka (XP_004065962.1)         ----------NFQENGRTQNNDDEMG------QHQHHIGKGNGLENTGWMEENPSNSTQQQ----------------APPSQTQDQDMW
Platyfish (ENSXMAP00000005447)   ----------NFQDNSGRQ--HQPDGQMGNSLQQSSNSVSVNGVEDTHQQRVNISCSTPSQTNWIERNLSHSQQQPTQCTASQAKEQELW
Spotted _gar (ENSLOCP000000155  ----------LINIHHVSANSPQPQPSYKPQQDQKALRHLLAHQAPSSNLEHKRAFKEEAGEMLQQTNSYAETTWIDLNSSQPPQQGDLS
Stickleback (ENSGACP0000002318  ------------------------------------------------------------------------------------------
Tilapia (XP_005457058.1)        ----------DFQENAVNPLRAESHGPMGSRLQQLHHTGMENGMENMSQQRANLSCSTPNQRGWRELKSSHSQQPPTSGPSSQAQEQDMW
Tetraodon (ENSTNIP00000021201)  ------------------------------------------------------------------------------------------
Zebrafish (ENSDARP00000101295)  ----------AVESKLIPQACTSPSQNRNNDQDNKSKLGLEKTLSKGTDPSSQMEWIDLNSTSASTPSTEHSQMWDLFLPQTNIQQHTVP
Salmon (XP_014042431.1)         ------------------------------------------------------------------------------------------
Salmon (XP_014065146.1)         ----------QQRGGADPGCPNSQMGWIDLNSRGQMLEPDSARRFQTGGQSFDVG-PTGGAYQQQQPRPAQSPHQAPPAQHNPAPEWQQQ
Salmon (XP_014050290.1)         ----------QQRGGADPSCPNSQMGWIDLNSRGQRLEPDSARRFQTGGQSFDVG-PTGGAYQQQQPRPAQSPHQAPPAQHNPAPEWQQQ
Rainbow trout (XP_021429026.1)  ------------------------------------------------------------------------------------------
Rainbow trout (XP_021429023.1)  ----------QQRGGADPGCPNSQMGWIDLNSRGQMLEPDSARRFQTGGQSFDVG-PTGGAYQQQQPRPAQSPHQASPAQHNTAPEWQQQ
Shark (XP_007910136.1)          ------------------------------------------------------------------------------------------
Human (ENSP00000386869)         ---------ATPRSSWPMPRPSPDPMAELEQLLGSA-SDYIQSVFKRPE----------------------ALPTKPKVKVEAPSSS--
Gorilla (ENSGGOP00000051172)    ---------ATPRSSWPMPRPSPDPMAELEQLLGSA-SDYIQSVFKRPE----------------------ALPTKPKVKVEARSSSPA
Armadillo (ENSDNOP00000021516)  ---------ATPRGSWPLPRPSPDPMAELEQLLGSA-SDYIQSVFKRPE----------------------ALPTKPKVKVEPPP----
Mouse (ENSMUSP00000087049)      ---------ATPRNSWPVPRPSPDPMAELEQLLGSA-SDYIQSVFKRPE----------------------ALPTKPKVKVEAPSSS--
Rat (ENSRNOP00000015296)Tet3    ---------ATPRNSWPVPRPSPDPMAELEQLLGSA-SDYIQSVFKRPE----------------------ALPTKPKVKVEAPPSS--
Pig (ENSSSCP00000008852)        ---------ATPRSPWPMPRPSPDPMAELEQLLGSA-SDYIQSVFKRPE----------------------ALPSKPKVKVEVPSSSSA
Elephant  (XP_010591957.2)      ---------ATPRSPWPMPRPSPDPMAELEQLLGSA-SDYIQS-----------------------------------------------
Whale (XP_023989264.1)          ---------ATPRSSWPMPRPSPDPMAELEQLLGSA-SDYIQSVFKRPE----------------------ALPSKPKVKVEVPSSSPS
Tasmanian_devil (ENSSHAP000000  ---------VAPCASWPVPRTGPDPMAELEQLLGGS-NDYIPSSFKRPE----------------------ALPTKPKIKVEPRS----
Chicken (ENSGALT00000059756)    --------------------TADPMAELEQLLGST--EYIATAFKRP----------------------EAGRAPPAAAPKPPD----
Lizard (ENSACAT00000001585)     --------SGPWGLNAVASPLSIDPMANLEQLLDSAGMEGLHADFKRPP----------------------EKSRVTKVKEEQAAQNDEN
Turtle (ENSPSIT00000006549)     --------------------------------------EEMQTDHPLPS----------------------TPVRHPPLALITPCS----
Xenopus (ENSXETP00000054061)    --------LQQWVQNADLLKSPSDPMTGLKQLLGNT-DEYIKSVFKGPE--------------------ALPNKKNVKPKHTIKSIKKE
Coelacanth (ENSLACP00000009535  --------LQGPQAQSQIPPAANDPMAELQQLLDNASNNYMKPVFKTPDMIPS-----------------KEKAMRSKQGQPVKPSSVEE
Spotted_gar (ENSLOCP0000001981  --------QQPSFPG-------CDPMSELQQLLGDTSGKYIGKSSNKIPEMLQQQHP----------------------QLHNEQVKDK
Amazon_molly (ENSPFOP000000021  --------DPEHS----------DPMSELKQLLSDT--KLKNASFKLPLTQQLSLNQ----------------------NGGIQ--VQG
Cave_fish (ENSAMXP00000015689)   --------Q-AHFPQPS--QSGSDPMSELKQLLGDTSGKYANAAFRFPGLPQNLKDGQKGGHPVMPYVKQEVDNGEYPT----------
Fugu (ENSTRUP00000036865)       --------SSEQS---------SNPMSELKQLLGDTTGKFSGAPFKLPVTQQPRLSQ----------------------NGSLQTQDNP
Medaka (ENSORLP00000019236)     ------------------------------------------------------------------------------------------
Platyfish (ENSXMAT00000000130)  ------------------------------------------------------------------------------------------
Stickleback (ENSGACP0000001732  ------------------------------------------------------------------------------------------
Tetraodon (ENSTNIP00000019883)  ------------------------------------------------------------------------------------------
Tilapia (ENSONIP00000019943)    --------GSEHRNPWMAPHSSSDPMSELKQLLGDTSSKFSNGPFKLPASQQLSLNQ----------------------NGTLQ--AQD
Zebrafish (ENSDARP00000085229)  ------------------------------------------------HERLEEG---------LLNQELSNG--------------
Rainbow_trout (XP_021462005.1)  --------QQSHFAPLSGGHSVGDPMSELKQLLGDSSGKYINDPFKLPVPHHHIKQE-----PGMPQVKQELNSGEYQSSACAYMGGRYG
Rainbow_trout (XP_021477599.1)  --------QQPRFAPQSGGHSTGDPMSELKQLLGDSSGKYISGPFKLPVPHHHIKQE-----PGMPRVKQEVDSGEYQSSACASMGGCYS
Salmon (XP_014026412.1)         --------QQSHFAPPSGGHSVGDPMSKLKQLLGDSSGKYINDPFKLPIPHHHIKQE-----PGMPQVKQEVNSGEYQSSACAYMGGRYG
Salmon (XP_014017100.1)         --------QQPRFAPPSGGHSTGDPMSELKQLLGDSSGKYINGPFKLPVPHHHIKQE-----PGMPRDKQEVDSGEYQSSACTSMGGCYS

                                        1180      1190      1200      1210      1220      1230      1240      1250      1260
                                ....|....|....|....|....|....|....|....|....|....|....|....|....|....|....|....|....|....|
European ampioxus (Sc0000095)   ------------------------------------------------------------------------------------------
Ciona intestinalis (XP_0021254  QNGSVSHGNQRFHG-------------------------------DLAFAAARSQRMVAEADQVGRADERGRFDAQKTFSETMNSVLYGQ
Lamprey (scaf_00044)            ------------------------------------------------------------------------------------------
Lamprey (scaf_03335)            ------------------------------------------------------------------------------------------
Shark (XP_007897316.1)          QQQNEQQQKSNGTLPPESRLTYQLSPL---CQPN-SIDNPVESEHEPRETHPSKPNISPLEILLEAISYPGISVNHTEMVNSAHLEEKKV
Lizard (XP_020663651.1)         QAENSNVLQESENSLLETSSLLALQDKSSCAPVFQKDGCPNDGASAIPSKDSHTEDLSLQSTFLSLIKTRNLTVEQVVAIEALTRLSEVP
Chicken (ENSGALP00000053457)    KEEN----------LLQTSSVLDLQES-TCCPPLVKDEVHGEDGFKVPNTETQHENPSLQPTFLSLIKNRNLTVEQVVAIEALTQLSEVP
Turtle (ENSPSIP00000006704)     SQKSNCTCK-----TFQGSDSSVLQTN-SIVD-IQERSCHTLVPEKIPNKETQLENPSLQPTFLSLIKKRNLTVEQVVAIEALTKLSEAP
Human (ENSP00000362748)         FQQPGFNCSS-----IPHSSHSIINHH----ASIHNEGDQPKTPENIPSKE-PKDGSPVQPSLLSLMKDRRLTLEQVVAIEALTQLSEAP
Gorilla (ENSGGOP00000011536)    FQQPGFNCSS-----IPHSSHSIINHH----ASIHNEGDQPKTPENIPSKE-PKDGSPVQPSLLSLMKDRRLTLEQVVAIEALTQLSEAP
Mouse (ENSMUSP00000133279)      FQKPGLNCRS-----GAEPT--IFNNH----PNTHSAGSRPHPPEKVPNKE-PKDGSPVQPSLLSLMKDRRLTLEQVVAIEALTQLSEAP
Rat (ENSRNOP00000054747)        FQKPGLNCRS-----GAEST--IFNNH----PDTHSTGSQHQPPEKVPNKE-PKDGSPVQPSLLSLMKDRRLTLEQVVAIEALTQLSEAP
```

```
Pig (ENSSSCP00000010918)       FQKPGFNCKS-----FPDPANCNLNSR----TSVHNESDQPKSLENITNRE-PKEGATLQPSLLSLMKDRRLTLEQVVAIEALTQLSEAP
Whale (XP_023987950.1)         IQKPGFNCKS-----FPDPANCNLNSH----PSIHNESDQPKSLEIITNRE-LKEGSTLQPSLLSLMKDRRLTLEQVVAIEALTQLSEAP
Elephant (ENSLAFP00000005179)  FHKPGFNSKS-----VPDPTNFNLNNH----TGTHNESNQPKTPENAPIKE-PKDGSPVQRSLFSLVKDRRLTLEQMVAIEALTQLSEAP
Armadillo (ENSDNOP00000014565) FQKPGFHCKS-----IPDSAKLSLNSH----TSPHNESEQPKTPETIPSKE-PKDGSPVQPSLLSLMKDRRLTLEQVVAIEALTQLSEAP
Tasmanian_devil (XP_023360087. IQKTDPICHS---AQDPTDSVLKSNIDAQENPPHIITWGKGEAPESKHISELKQYNSPVQPCLLSLMRDRRLTLEQIVAIEALTQLSQAP
Coelacanth (ENSLACP00000016889 SQQSEQQPESQ-----WSLLQHQLPSQ---PVPSGSLTNTSTSWENSPETS---------NVLLHACSSAALVSDH---VNSHHTVENSA
Cavefish(XP_007240334.2)       GPQTSNACNIKTER--------EEEPYQSHSHQER-TSNSPAGADNSHPK---DPVPLTNNSVQSLVEHRNISIDQAIAIEALTQLAATP
Platyfish (XP_014325204.1)     GTRSSILCSIKTEPPDLHFVKTEK-----DELKKS-TPANLQRTPQPIKS--EPPQSILLPSVQSLVERRNLSFDQAIAIEALTQLAVIP
Tilapia (XP_005456583.2)       SSRASILHSIKAEAPDLHFVRKEKE----DLYLKS-TPVNLQSTPPPIKS--EPLPSVLLPSVQSLAERRNLSVDQAIAIEALTQLAAIP
Spotted_gar (XP_015202470.1)   KPISPFSRNETCK-------LEETEVTTDDACSTSGATNGATSLENIDASFCRNSLVLSHPSLQSLVEKRNISIEQAIAIEALTELSEIP
Stickleback (ENSGACT0000001035 ------------------------------------------------------------------------------------------
Tetraodon (ENSTNIT00000011701) ------------------------------------------------------------------------------------------
Fugu (XP_011601624.1)          SSRASILRSIKTEPPDLHLVKEEPEEFKNNSCQKS-TPLGPQRPSHGVKS--EPPPSLLLPSVQSLAEKRNLSFDQAIAIEALTQLAAIP
Zebrafish (ENSDARP00000143362) GSQTSDVC-LKTEP--------KEEPYQCQLNQEE-PPQN-------------DYIALSLPTVQSLVQHRNISVDQAIAIEALTQLAAIP
Amazon_molly (XP_016534180.1)  GTRSSILCSIKTEPPDLHFVKTEK-----DELKKN-TPANLQRTPQPIKS--EPPQSVLLPSVQSLAERRNLSFDQAIAIEALTQLAVIP
Medaka (XP_023818916.1)        SRRSSILRSIKTEPPDLHFVKKEENYLNDDWCPKS-TPLNTKRTPHTIKC--EPSPSVLLPSVQYLAERRNLSFDQAVAIEALTQLAAIP
Rainbow_trout (XP_021469805.1) SSRSPVLRSFK----------TEPEDFKVDICLKS-APISPRKTFDIIVKREQPSPDLSQPSIQSLVEERNISNDQAIAIEALTLLAAIP
Rainbow trout (XP_021436521.1) SSRSPVLHSLK----------TEPEDFKVDLLLRS-TPISPRKALDIIVKREQPSPDLSLPSIQSLVQERNISNDQAIAIEALTLLASIP
Salmon (XP_014010485)          SSRSPVLRSFK----------TEPEDLKVDICLKS-APISPRKALDIIVKQEQPSPGLSQPSIQSLVEERNISNDQAIAIEALTLLAAIP
Salmon (XP_014070638.1)        SSHSPVLHSLKTE------LKTEPEDFKVDLLLKS-TPISPRKGLDIIVKREQPSPDLSLPSIQSLVQERNISNDQAIAIEALTLLASIP
Shark (XP_007890120.1)         YLTKHEEISKLCSN------------------------------SQTTDAEPRGVQLQQLQKQSSQTQQEEAQSLTQPKSQMQMVHQYRP
Human (ENSP00000425443)        QYQPNLSNQMTSK--------------------------------QYTGNSNMPGGLPRQAYTQKTTQLEHKSQMYQVEMNQGQSQGTVD
Gorilla (ENSGGOP00000034525)   QYQPNLSNQMTSK--------------------------------QYTGNSNMPGGLPRQAYTQKTTQLEHKSQMYQVEMNQGQSQGTVD
Mouse (ENSMUSP00000143029)     HSQTGPVNQMSSK--------------------------------QSTGNVNMPGGFQRLPYLQKTAQPEQKAQMYQVQVNQGPSPGMGD
Rat (ENSRNOP00000048956)       RYQTSPTNQIGSI--------------------------------QSTGSVNMTGEFQRPPYIQKTAQPEHKSQMYQVEMNQGPSPGMGD
Pig (ENSSSCP00000009765)       QYQSNPSNHMTSK--------------------------------QYTGNSNMPGGLPGQAYIQKTMQPDQRPQRYHVEMNQGQSQGTVD
Tasmanian_devil (ENSSHAT000000 QYQSNKIDQMTSK--------------------------------QYSRGPNISKGTQGQTYIQKMVQQDQMPQKYQMENAQVQFQALMD
Whale (XP_007106564.2)         QYQSKPSHQVTSK--------------------------------QYTGNSNMPGGLPGQAYTQKIMQPEQRPPRYQAEMNQGQSRGTVD
Elephant (XP_023404313.1)      LYESHPSNQMTSR--------------------------------RYTGYSNVRGGLPRQAYVQKLMQLEQRSQMYQAELNQGQSQGTVD
Lizard (ENSACAP00000008438)    QFQSNSSEQTHPE--------------------------------QYSENPDRSKGHTEQACYQNITQCEQIPALYKNEVTQLPPHLTAN
Chicken (ENSGALP00000017216)   QYQANTSKAAYMK--------------------------------QYAGSPDALKGPSGQPQSQKIMQQEQIPLQYKSESSQMQQHPTAD
Turtle (ENSPSIP00000011691)    QFQANPMEQVHSD--------------------------------QYPGNPDALKGPSGQPHSEKTIQQEPMPQLYKSEAPQRPLHPIAE
Xenopus (ENSXETP00000030770)   QSQTN--NKKKLR--------------------------------QYPGKQGLAN----------------------RKCNSQDSEEHLQ
Coelacanth (ENSLACP00000008576 QLQQHQNEQIQCQK------------------------------QEYIDSPNVSNGFQLQNFNQ-----DQLCLLYKNELAHGQQQSRAE
Amazon_molly (ENSPFOP000000120 R---AKPQSEQQTT------------------------------SPQVQNQILEPN-----QAQRFRETQTQGLFTDAGRGSNGSLQKQP
Amazon_molly (ENSPFOP000000299 R---AKPQSEQQTT------------------------------SPQVQNQILEPN-----QAQRFRETETQGLFTDSGRGSNGSLQKQP
Fugu (XP_011611191.1)          REFPSKLQSDQQIV------------------------------NPQVHNQISEPK-----PTQRF---QTQRSFTDSNQQSNSFQQQQQ
Medaka (XP_004065962.1)        RALPPK-PNVERTA------------------------------SPQRDTQIMEPG-----PVQRF---QIQRSLADMSPGSNTSQQKLP
Platyfish (ENSXMAP00000005447) R---AKPQSEQQTT------------------------------SPPVQNQILEPN-----QVQRYSETQTQGLFTDTSRGSNSSLQKQP
Spotted _gar (ENSLOCP000000155 RLWKDFNLRQQQQM------------------------------SNQSQEKILDSDNMQGFQIQGFSHSSMQQQQKQLSDVYKPNVEQDQ
Stickleback (ENSGACP0000002318 ------------------------------------------------------------------------------------------
Tilapia (XP_005457058.1)       RNFSIKSMSEQQTA------------------------------NPPVHGQMFEPN-----PTQRF---QTQGVFSDGSQGSNF-QKKQQ
Tetraodon (ENSTNIP00000021201) ------------------------------------------------------------------------------------------
Zebrafish (ENSDARP00000101295) Q-SQSQCINSIPPN------------------------------NFQSHNFSNSSF-----PSHEIEAQENKNMLPPTRFPHSAAPESQQ
Salmon (XP_014042431.1)        ------------------------------------------------------------------------------------------
Salmon (XP_014065146.1)        ANSKGPSHMHQQQC------------------------------NLPPQQQPHTQH-----MHQQQCNLPPPQQQQQGENCFRAPMQPEH
Salmon (XP_014050290.1)        ADSKGPSHMHQQQC------------------------------NLLPQQQPHTQH-----MHQQQCNLPPPQQQQQGENCFRAPMQPEH
Rainbow trout (XP_021429026.1) ------------------------------------------------------------------------------------------
Rainbow trout (XP_021429023.1) ADSKGPSHMHQQQC------------------------------NLPPQQQPHTQH-----MHQQQCNLPPPQQQQQGENCFRAPMQPEH
Shark (XP_007910136.1)         ------------------------------------------------------------------------------------------
Human (ENSP00000386869)        PAPAPSPVLQREAP--------------------------------TPSSEPDTHQKAQTALQQHLHHKRSLFLEQVHDTSFP-APSEPS
Gorilla (ENSGGOP00000051172)   PAPAPSPVLQREAP--------------------------------TPSSEPDTHQKAQTALQQHLHHKRSLFLEQVHDTSFP-APSEPS
Armadillo (ENSDNOP00000021516) PAPAPSPAPQREAP------------------------------------EPDAHQKAQTALQQHLHHKRGLFLEQARDAAFS-APLEPP
Mouse (ENSMUSP00000087049)     PAPVPSPISQREAP--------------------------------LLSSEPDTHQKAQTALQQHLHHKRNLFLEQAQDASFP-TSTEPQ
Rat (ENSRNOP00000015296)Tet3   PAPAPSPISQREAP--------------------------------LPSSEPDTHQKAQTALQQHLHHKRNLFLEQAHDASFP-TSTEPQ
Pig (ENSSSCP00000008852)       PSPSPT--LQREAP--------------------------------TPSSEPSTHQKAQTALQQHLHHKRSLFLEQARDAAFP-HPSEPP
Elephant  (XP_010591957.2)     ------------------------------------------------------HDAILPLLLQSLL-----------------------
Whale (XP_023989264.1)         PSPSPSPILQREAP--------------------------------TPSSEPDSHQKAQTALQQHLHHKRSLFLEQAHDASLP-PPSEPP
Tasmanian_devil (ENSSHAP000000 -PPAPTPSPRKEAP--------------------------------TPSSDPDTHRKVQSTLQQHLHHKRSLFLEQNQNGGCPRASSVPP
Chicken (ENSGALT00000059756)   ---RTPGKEAASSP--------------------------------RVLQEPDLHRKTQLVLQQHLHHKRSLFLEQNLS---APPSERP-
Lizard (ENSACAT00000001585)    AGYRRPSACSPHLS--------------------------------QLLQEPNFHKKTRTALQQHLHHKRSLFREQKATPTATPPPQGPP
Turtle (ENSPSIT00000006549)    --------------------------------------------------------AAQSPLQKQLDDRSQAPNTQEMC-----------
Xenopus (ENSXETP00000054061)   STEFLKMSPDQQLS--------------------------------QLLQTNEFHRNTQAALQQHLHHKRNLFVDPNAMEACTQEQQN--
Coelacanth (ENSLACP00000009535 GARYYGTTCRQQLS--------------------------------QPTEQTGLQQTTQKALQQHLHHKRNLFQEQGQTQASQPMQPN--
Spotted_gar (ENSLOCP0000001981 GASVARIKQEPFSE--------------------------------GDFPGSAIHHKTQVALQQHLNHKRSLFQDQSDQ----TSALPSS
Amazon_molly (ENSPFOP000000021 ---RIKQEPDSGNP--------------------------------MSPGQAAIRHSTQAALQHHLHHRRNLFSTHPPGFGVPSPRAPMT
Cave_fish (ENSAMXP00000015689)  -----------------------------------------------------IRHSTQAALQQHLHHKRNLFSSPPP----FSPQSPMA
Fugu (ENSTRUP00000036865)       APARIKQEPDLANC--------------------------------QQQGQTSISHSTQAALQQHLHYKRNLFSNHSPSFGAPGPRASLA
Medaka (ENSORLP00000019236)    ------------------------------------------------------------------------------------------
Platyfish (ENSXMAT00000000130) ------------------------------------------------------------------------------------------
Stickleback (ENSGACP0000001732 ------------------------------------------------------------------------------------------
Tetraodon (ENSTNIP00000019883) ------------------------------------------------------------------------------------------
Tilapia (ENSONIP00000019943)   NPALHQGQHYPGTP--------------------------------LSPGQAAISHSTQAALQQHLHYKRNLFSNHPSGFGGVGPRGPLA
Zebrafish (ENSDARP00000085229) -------------------------------------------------------------LSKHASNEENVCDNED-------------
Rainbow_trout (XP_021462005.1) LMNGQQQPGYPGPP--------------------------------LSAGSTAIHYSTQTALQQHLHHKRNLFSNPPGPPGLCPPRPTQA
Rainbow_trout (XP_021477599.1) LMNGQQQPRYPGPP--------------------------------LSLGSASIRHSTQTALQQHLHHKRNLFSNSPGLPGLCPLRPAKA
Salmon (XP_014026412.1)        RMNGQQQPGYPGPP--------------------------------LSAGSTAIHYSTQTALQQHLHHKRNLFSNPPGPPGLCPPRPTQA
Salmon (XP_014017100.1)        LMNGQQQPRYPGPP--------------------------------LSPGSATIRHSTQTALQQHLHHKRNLFSNSPGLPGLCPLRPAKA

                                        1270      1280      1290      1300      1310      1320      1330      1340      1350
                               ....|....|....|....|....|....|....|....|....|....|....|....|....|....|....|....|....|....|
European ampioxus (Sc0000095)  ------------------------------------------------------------------------------------------
Ciona intestinalis (XP_0021254 SDPAKPNPLLGEIKTASSPTDPQS--------------------------------------------TISPGESGYDSADLISTVDSQR
Lamprey (scaf_00044)           ------------------------------------------------------------------------------------------
Lamprey (scaf_03335)           ------------------------------------------------------------------------------------------
Shark (XP_007897316.1)         NGNCQFFPNLSSDLPNNINYSTLTTQSL------EFSDRENCDVENGKVSKCLITG------------VRNKAEEEAAAQLTQLAAIIES
Lizard (XP_020663651.1)        AGASSPAKTERVQCTDEQISHSLNSYKKDNACSLTSSALKAIKGTCPQKDQQLFSHVPSPRRILP-KSLLYNGQGAISEFPSKSSGPSSL
Chicken (ENSGALP00000053457)   LGTSSPAKADNTECTEERTSNLLHSYKRDISSSFTSSTLKEIKDTYLEKEEQLLTHCAHSQKQLPNKPVLYNGQSSISELRDKPPNLASE
```

```
Turtle (ENSPSIP00000006704)       LQASVPDETENIEDAEQTTSHVLRNYKRDISGSLTSSTLKEIKETYLQNEQHLLSRCAHSQKQFLNKAVLYNGQSAVFEPLDKSVNTTSG
Human (ENSP00000362748)           SENSSPSKSEKDEESEQRTASLLNSCKA-----ILYTVRKDLQDPNLQGEPPKLNHCPSLEKQSSCNTVVFNGQTTTLSNSHINSATNQA
Gorilla (ENSGGOP00000011536)      SENSSPSKSEKDEESEQRTASLLNSCKA-----ILYTVRKDLQDPNLQGEPPKLNHCPSLEKQSSCNTVVFNGQTTTLSNSHINSATNQA
Mouse (ENSMUSP00000133279)        SESSSPSKPEKDEEAHQKTASLLNSCKA-----ILHSVRKDLQDPNVQG--KGLHH----------DTVVFNGQNR-TFKSPDSFATNQA
Rat (ENSRNOP00000054747)          SENSSPSKTEKDEETDQRTASLLNSCKD-----VLHSVRKDFQDPNVQG--KSLHH----------DTVVFNGQNR-AFKSPDSFATNQA
Pig (ENSSSCP00000010918)          SENSSPSKSEKDEETEQRTASLLNSCKA-----ILYSVRKDLQDPNLQGEPQSLHHCPSLEKQNSCNTIVFNGQNN-ISKSHSSSPSDQA
Whale (XP_023987950.1)            SENSSPSKSEKDEETQQRTASLLNSCKA-----ILYSVRKDLQDPNLQGGPQSLHHCPSLEKQSSCNTLMFNGQN--ISKSHNSSSSDQA
Elephant (ENSLAFP00000005179)     SENSSPSKSEKDEETDQRTAALFNSCKV-----FLCSVR---EDPNSQGEPQSLHHFPSLEKQSSCNMVIFNGQHT-VCKSHSSTVTNQA
Armadillo (ENSDNOP00000014565)    SENSSPSKAE-DEETEQRTASLLNSCKA-----ILYSVRKDLQDPSLQVEPQSLHHCSSLEKQNSCSTLVFNGQST-IAKTHNIPATNPA
Tasmanian_devil (XP_023360087.    SENSSPSKSEKYEETAQLTTNLLHNCKT-ILCSVGSPAGKDFQDPNLKIDSLVLHPCPSLEKQTPRKPVLYNGQN--NSKWHNSLMTNHS
Coelacanth (ENSLACP00000016889    SK----SPNHLHFLQQRINDSVETSQSE------PSNSNTTLDLNS---SAVFSKR------------VRNKDEVDVAAQLAQLALIIES
Cavefish(XP_007240334.2)          KSAP--VKADSKVQDPPQESCNKL--------------ANVSSSRQSPVQEAKP-----ISEVISNKVSVINS--HQTSVICSPLNRQEN
Platyfish (XP_014325204.1)        QSPSGPIKAESNGEHPISSSSP----------------GFNTNPALQAAKR--------ASAIHYNKVSVISSPLHQTSVIRPPVARQGN
Tilapia (XP_005456583.2)          QSTPGSIKTESKCEHPISTAVPSS--------------ASSTNTVLQEAKP--------AVAFGYNKVSVISSPLHQMSVIRPPVARQGN
Spotted_gar (XP_015202470.1)      --LQNLAQTAETCHSPEVSTPPPG--------------KAELPNDKNS-----------ASGSCLRDTLTVSSLQHENATLPALLHNQGS
Stickleback (ENSGACT0000001035    ------------------------------------------------------------------------------------------
Tetraodon (ENSTNIT00000011701)    ------------------------------------------------------------------------------------------
Fugu (XP_011601624.1)             QRTQRSIKAEN----PISNAAPFV--------------SATTN-TPLVVRA--------SAGVHCNKVSVISSLRHQMSVICPPVARQGN
Zebrafish (ENSDARP00000143362)    QTVP--FKTENQIQDAQQETS-----------------TYSAPSNHIPIREAKP-----ATDVVSNKVSVISSSLHQTSVIRSSLNTPTN
Amazon_molly (XP_016534180.1)     QSPSGPIKAESNGEHPISSSSP----------------GFHTNPAQQAAKR--------ASAIHYNKVSVISSPLHQTSVIRPPVARQGN
Medaka (XP_023818916.1)           QSAQGAIKPENNHEHHMSTASTCS--------------NSKTNTALQEAKP--------MSAICYNKVSVISSPLHQMSVIRPPVARQGN
Rainbow_trout (XP_021469805.1)    QNSPEPFRTNAQGENPTTSSMHLS--------------NRNTPLQGS--------NKVLVISSPLHQTSVIRSPLHQTSVIRSPVARQAH
Rainbow trout (XP_021436521.1)    QNPPEPFRMDAHGQNPTTSSMHLS--------------SRNTSLQDVKPPTGFLPNKVLVISSPLHQTSVIRSPLHQTSVIRSPVARQSL
Salmon (XP_014010485)             QNSPEPFRTDAQGENPTTPSMHLS--------------NRNTPLQGS--------NKVLVISSPLHQTSVIHSPLHQTSVIRSPVARQAH
Salmon (XP_014070638.1)           QNPPEPLRMDAHGQNPTTPSMHLS--------------SRNTSLQDVKPPTGFLPNKVLVISSPLHQTSVIRSPLHQTSVIRSPVARQSL
Shark (XP_007890120.1)            QSAPQQLLQLNQDKTDNMIENKLVLQKMVQCRPHSVPQQQDQHNLVLLKP--------QTKQWHLQQNALTQEQMAQIQAGQNVRYQYGP
Human (ENSP00000425443)            QHLQFQKPSHQVHFSKTDHLPKAHVQSLCGTRFHFQQRADSQTEKLMSPVLK-QHLNQQASETEPFSNSHLLQHKPHKQAAQTQPSQSS-
Gorilla (ENSGGOP00000034525)      QHLQFQKPSHQVHFSKTDHLPKAHVQSLCGTRFHFQQRTDSQTEKLMSPVLK-QHLNQQASETEPFSNSHLLQHKPHKQAAQTQPSQNS-
Mouse (ENSMUSP00000143029)        QHLQFQKALYQECIPRTDPSSEAHPQAPSVPQYHFQQRVNPSSDKHLS---------QQATETQRLSG--FLQHTPQTQASQTPASQNSN
Rat (ENSRNOP00000048956)           QHLQFQKTSYQECISRTDLPPEAHG-----PQYHFHQRVDPSTDK------------QQATEAELFSS--FLQHTPHKQAAQTQASQNSN
Pig (ENSSSCP00000009765)           QHLQFQKPSLQVHFSKTDPSPEAHRQSLCAPRFHFQQRPDPQTEKLIPLTLK-QHLNQQASETEPFSNSNLLQQKPHKEAAQTLPSQNS-
Tasmanian_devil (ENSSHAT000000    Q---LQKHSPQMPLPKIEPPLQIHMQAQCMPKFHFQARPDYQVEKPLDPSLKQQHLNLQAAETEPFLPSHLSQPSFQKQAAQAKLPLNL-
Whale (XP_007106564.2)            QHLQFQKPSLQVHFSKTDPSPEAHRQSVCALRCHFQQRPDPQTEKLTPPTLK-RHLNQQASETDPFSNSHLSQHKPHKQAAQTQPSQTS-
Elephant (XP_023404313.1)         QNLQFQKPSPQVHFSKTDPSPEAHKQSLCAPRLHFQSRPDPQTQKLIPMSLK-QHLNQQASEAEPFSNSHLLQFKPHKQATQTQPSQNS-
Lizard (ENSACAP00000008438)       QQMHFQKHSP-PQPTKMNTMLKSHEQQQNAKHFHFEARAEHHNEESL-----------EAQENEQFLRSHILQQMLQ--TQPVQLPQNPQ
Chicken (ENSGALP00000017216)      LQLLFQKHSPQPQLTKMDSLLKSRVQQHPPQQLHFQQQLEQQTEQPLGAPLKQQHLNPQPGESEQFLHSHILQQMLQKQTQQTQMLCSP-
Turtle (ENSPSIP00000011691)       QQLQIQKHSPQPQLTEMKSLVKAHVQQHHVQQLHFQARQEQQTEQSLGTLLKQQHLNLQATEKEQFVHSNILQQMLQKPAQQTQVSRSPQ
Xenopus (ENSXETP00000030770)      PNISEQQPNFPKHLSQDS----------------------------------------QYQPYSQKTNNGLMWPDLEQASEGTQLGDVQ-
Coelacanth (ENSLACP00000008576    QQVQFQKHSPQVQHPKPEPQLQPYMQQHKAQQSHSQAGPTQHSQHAFTQQLQQQHLSQNTQETQQALQSQFVHQPLDKQLQETQLQQSQT
Amazon_molly (ENSPFOP000000120    DCLPAHMQCAPVQHNTAPEWQQSN----------------------------------SKAP--QMQQHLLQKMPETKNFPQNQQSNCY-
Amazon_molly (ENSPFOP000000299    DCLPAHMQCAPVQHNTAPEWQQSN----------------------------------SKAP--QMQQHLLQKMPETKNFPQNQQSNCY-
Fugu (XP_011611191.1)             DCIPTQRHCAPTQRNTAPEWQHTD----------------------------------SKAL--EQHTVPPNEHT---------------
Medaka (XP_004065962.1)           ELLSNQMQFAPSQQNTATRWQHQS----------------------------------PKAF--QMQQTPVTEMSDQQNFPQNQQGNN--
Platyfish (ENSXMAP00000005447)     DCLPAHMQCASVQHNTAPEWQQSN----------------------------------SKAP--QMQQHLLQKMPETKNFPQNQQSNCY-
Spotted _gar (ENSLOCP000000155    AQRTDTAEWQQMNSNTPPGVQQVE----------------------------------PQMQPNKLQHQSSQSSLSQQQVQHPFQPKMLQ
Stickleback (ENSGACP0000002318    ------------------------------------------------------------------------------------------
Tilapia (XP_005457058.1)          DYLPMQTHCASAQHNTASEWQQSN----------------------------------SEGA--QIQQPLAQKMPEQQKLTQNQQVDSRC
Tetraodon (ENSTNIP00000021201)    ------------------------------------------------------------------------------------------
Zebrafish (ENSDARP00000101295)    RNSGMHNHIN-MQFNNPQHMSQKL----------------------------------CKND--QDQILSPSFLSQLPPTETHQQATS--
Salmon (XP_014042431.1)           ------------------------------------------------------------------------------------------
Salmon (XP_014065146.1)           LCEGDPDLEEILSPSFLQPPQHPQ----------------------------------HQQQ--QQRPLSHPSQYDGQQSVNAPQSQDPN
Salmon (XP_014050290.1)           LCEGDPDLEEILSPSFLQPPQHPQ----------------------------------HQQQ--QQRPLSHPSQYDGQQSVNAPQSQDPN
Rainbow trout (XP_021429026.1)    ------------------------------------------------------------------------------------------
Rainbow trout (XP_021429023.1)    LCEGDTDLEEILSPSFLQPPQRPQ----------------------------------HQQQ--QQRPLSHPSQYDGQQSVNAPQSQDPN
Shark (XP_007910136.1)            ------------------------------------------------------------------------------------------
Human (ENSP00000386869)           -----APGWWPPPSSPVP--------------------------------------------------RLPDRPPKEKKKKLPTPAGGPV
Gorilla (ENSGGOP00000051172)      -----APGWWPPPSSPAT--------------------------------------------------RLPDRPPKEKKKKLPTPAGGPV
Armadillo (ENSDNOP00000021516)    -----APGWWAPPSSPAP--------------------------------------------------RPPDRPPKEKKKKPPAPAGGPV
Mouse (ENSMUSP00000087049)        -----APGWWAPPGSPAP--------------------------------------------------RPPDKPPKEKKKKPPTPAGGPV
Rat (ENSRNOP00000015296)Tet3      -----APGWWAPPGSPAP--------------------------------------------------RPPDKPPKEKKKKPPTPAGGPV
Pig (ENSSSCP00000008852)          -----APGWWAPPSS-----------------------------------------------------RPSDRPPKEKKKKSLAPVGGPT
Elephant  (XP_010591957.2)        -----LPAWWAPASSPAP--------------------------------------------------RPPDKPPKEKKKKPLAPSGGPM
Whale (XP_023989264.1)            -----ASGWWAPPSSPAS--------------------------------------------------RPSDRPPKEKKRKPLAPAGGLV
Tasmanian_devil (ENSSHAP000000    -----TRGWWAPPIPGTP--------------------------------------------------RPLDKPAKEKKKKQLTAVGGLV
Chicken (ENSGALT00000059756)      ------PGWWTPSTP-----------------------------------------------------KPFEKQAKEKKKRMQ-------
Lizard (ENSACAT00000001585)       TRQEQLPAWWSPSPPPQQQQQSPK--------------------------------------------AAAEKPVKERRRKTPQ------
Turtle (ENSPSIT00000006549)       ---------------------------------------------------------------------AFSQGSPEENRQHPL------
Xenopus (ENSXETP00000054061)       --------WWVPSSQQAP----VS--------------------------------------------KTTEKPVKERKKRRQSP-----
Coelacanth (ENSLACP00000009535    --------WWTPASPLLP----PR--------------------------------------------ETEKQPVKEKKKKLQSPLL---
Spotted_gar (ENSLOCP0000001981    F--QDLRKWWPHPGGD------LS--------------------------------------------IKQELKEIKKKKTSQTSQYLKQ
Amazon_molly (ENSPFOP000000021    C--QDLKKWWPQMDAEGLSILPI-------------------------------------------------KQEPKRRKNTQGSPVMKS
Cave_fish (ENSAMXP00000015689)     C--QTLRKWWPQTTQQE---STLA--------------------------------------------IKQEPKEPKKKKNTQSSPMLKQ
Fugu (ENSTRUP00000036865)          C--QNLKKWWPQG--EGFPHLPI-------------------------------------------------KQEPKKKKSNQGSPVIKS
Medaka (ENSORLP00000019236)        ------------------------------------------------------------------------------------------
Platyfish (ENSXMAT00000000130)    ------------------------------------------------------------------------------------------
Stickleback (ENSGACP0000001732    ------------------------------------------------------------------------------------------
Tetraodon (ENSTNIP00000019883)    ------------------------------------------------------------------------------------------
Tilapia (ENSONIP00000019943)      C--QNLKKWWPQMDAEALSHLAI-------------------------------------------------KQEPKRKKISQGSPGFKA
Zebrafish (ENSDARP00000085229)    ---------------------------------------------------------------------RDATKEQHQHNFHEGAGSISQ
Rainbow_trout (XP_021462005.1)    C--QNLRKWWPQQMGPEGHLIPVA--------------------------------------------IKQEPKEPKRRKSTVGTSPLLK
Rainbow_trout (XP_021477599.1)    C--QNLRKWWPQQMGPEGHPVPVA--------------------------------------------IKQ---ETKRRKSTAGTSPLLK
Salmon (XP_014026412.1)           C--QNLRKWWPQQMGPEGHLIPVV--------------------------------------------IKQEPKEPKRRKSTAGTSPLLK
Salmon (XP_014017100.1)           C--QNLRKWWPQQMGPEGHPVPVA--------------------------------------------IKQEPKETKRRKSTAGTSPLLK

                                           1360      1370      1380      1390      1400      1410      1420      1430      1440
                                  ....|....|....|....|....|....|....|....|....|....|....|....|....|....|....|....|....|....|
European ampioxus (Sc0000095)     ------------------------------------------------------------------------------------------
Ciona intestinalis (XP_0021254    NMYDGTIQQRPSANTDLQQAKNLHAASAGLTHQQGSEFEQPSSSAAPTNVHCTKTEGCN------------------TNSNLLPAEAAVT
```

```
Lamprey (scaf_00044)                   ------------------------------------------------------------------------------------------
Lamprey (scaf_03335)                   ------------------------------------------------------------------------------------------
Shark (XP_007897316.1)                 QQYCQNWLKHKECKDVLHDDVHQKGWPKQKKMFLQHQQRVPVLPKQLTLALQRKNKAPSH-KHNQT-----PQATSRMQPYKQRQQKQPK
Lizard (XP_020663651.1)                LRRLHGSPRDTKSFSALASGG--SSAHPTKKYPLHPKAAIGPCAKNSSAVKQRRNKVDMLGKWEGK---AIHSLNSKSNSVIR-GSIHSQ
Chicken (ENSGALP00000053457)            MYKLQLSSRDTKTLSELRSNAKSFSGLNTKKN--YDPVTLSRCCN----VQHTSNNLDTLGQLDQKP--TCNDSSLEAGSLIKEGGIHSQ
Turtle (ENSPSIP00000006704)            SYRSHTSRRDTKSLSVSVPNAKLSSGYSAKKNSLYDKVTIAACSNNSHNAHQTSGNLEILSQFDKNI--AYNDLNLKNNFIMKEGGIHSQ
Human (ENSP00000362748)                STKSHEYSKVTNSLSLFIPKSN-SSKIDTNKSIAQGIITLDNCSNDLHQLPPRNNEVEYCNQLLDS----SKKLDSDDL--SCQDATHTQ
Gorilla (ENSGGOP00000011536)           STKSHEYSKVTNSLSLFIPKSN-SSKIDTNKSIAQGIITLDNCSNDLHQLPPRNNEVEYCNQLLDS----SKKLDSDDL--SCQDATHTQ
Mouse (ENSMUSP00000133279)             LIKSQGYP---------------SSPTAEKKGAAGGRAPFD-GFENSHPLPIESHNLENCSQVLSC----DQNLSSHDP--SCQDAPYSQ
Rat (ENSRNOP00000054747)               LIKSHEYS---------------RSPTAG-KSIASGKAPLD-SSKNSCPLPEESQNLENFSQVLIC----DQNLNSHDP--SCQDAPYSQ
Pig (ENSSSCP00000010918)               STKAQEYSTVTNSISLFIPNSN-SSKTDTNKNVAQGKVTLN-SSKNLHQLTVTN-KLENCNQSLDS----SNKLDSKDN-PSCQDTVHSK
Whale (XP_023987950.1)                 STKAQEYSIVTNSVSVFIPNSN-SSKTDTNKNVAQGRTALD-SSKNLHQLTTTTSKSEYCNQSLDS----SIKLDSKDD-PSCQDTVHSK
Elephant (ENSLAFP00000005179)          PTKSQEYSKVTNSISLFVPNSN-SSKADAGKNVAQGKITLDSYSKNVHQLPPRSNQSEYSNQLLDS----SKKLDSKDD-PSCQDTAHSQ
Armadillo (ENSDNOP00000014565)         STKSQEYSKVTNSISLFIPNSH-SSKIDTNKNADQGRITLDNCSKDLHQLPPTSNKLGHYNQLLDS----SKKLDTKDDDPSCQDAAHSQ
Tasmanian_devil (XP_023360087.         TSKTHASSKVIKSGSITEPGSNSPRNHSNKEIVLQGRITVGRCSKSTHGLQTSS-KLEFCRQLQES---TSKNFDSKND-LSHRTTPHSQ
Coelacanth (ENSLACP00000016889         SQYCQNGPQHKQPINTTETNLAS---EETKQTYSQEQN---VLQEKTDLLKKPSYQTLSAGKQKPS-----PQKKAKSTPLKQSFKKKPQ
Cavefish(XP_007240334.2)               PTHP---------KLSLQDLLIAS-SYDKLLHSPENGRVSQALCKT----------------------EKLDGTFKTFKHVERTQTSRRR
Platyfish (XP_014325204.1)             VIQC-SRRLSSDTKPS---VAEMRSN-----CRGTGQ-GQAPYVINSQCS----YKDS-----S----RGHERLV-EDRERCVIKMRRNR
Tilapia (XP_005456583.2)               VIQR-SNGLSINKLQS---LLEASSDSDKTISRRTEQ-DCGAYVIKSEFS----YKTPTDVKFS----KDHERLS-MEG-GVVAKVSRNR
Spotted_gar (XP_015202470.1)           INNS--QTLLSTGKLSLQDLLEASSLTDKIQTHVSPEKQCSYIEQSLNFHRDHTIFRTQGLDGRGAPFQGHKQSPCSDGSLLPNKRVRNK
Stickleback (ENSGACT0000001035         ------------------------------------------------------------------------------------------
Tetraodon (ENSTNIT00000011701)         ------------------------------------------------------------------------------------------
Fugu (XP_011601624.1)                  VIKC-SQGFSSNAKLAHLDPSKSGSDSNKTPCRRAEQGYLGSPVIKSECS----YKDLNDATFR----KDCDRVFREEKYRVPGKE-RNK
Zebrafish (ENSDARP00000143362)         FTQSQRCAPPPR-KLSLKDLLMASSECEKLSYTTENRRNGQALCKS----------------------DRLEGTFKMYRHGEATPIYRKK
Amazon_molly (XP_016534180.1)          VIQC-SRRLSSDTKPS---VEEMPSN-----CRGTNQ-SQSPYVIHSQCS----YEDS-----S----RGHERLG-EDRERCVIKMRRNR
Medaka (XP_023818916.1)                VIQC-SRGLNPDTKVL---VPDRLEKIS---RDRTAF-RLGERGNASHSI----RLDSSQMKFS----SAHDQLSQVDGARSQSKVVRNS
Rainbow_trout (XP_021469805.1)         VIQCHSRSPAATGNLSLQDLLEASSDCDRLPYDSTERAGQHLYRRADQGLGSQHNRGEGTFRQR----KDMERNSKETIERTVGKSGRNR
Rainbow trout (XP_021436521.1)         VIQCHSRSPAATGKLSLQDLLEASSDCGRLPYDSTERAGQHLYRRADQDLGSHHNRSEGTFRER----KDMERNSKETTERTVGKSGRNR
Salmon (XP_014010485)                  VIQCHSRSPAATGNLSLQDLLEASSDCDRLPYDSTERAGQHLYRRADQGLGSQHNRGEGTFRQR----KDMERNSKETIERTVGRSGRNR
Salmon (XP_014070638.1)                VIQCHSRSPAATGKQSLQDLLEASSDCGRLPYDSTERAGQHLYRRADQDLGSHHNRSEGTFRER----KDMERNSKETTERTVGKSGRNR
Shark (XP_007890120.1)                 NQHEKLPTIHQQQVQDPNPNAAQTCEYREPQYQCGTEQQYSQIIKPVKFIKTEKQGEIS-----------------NYCHQSHILQQQQD
Human (ENSP00000425443)                -HLPQNQQQQQKLQIKNKEEILQTFPHPQSNNDQQREGSFFGQTKVEECFHGENQYSKS-----------------SEFETHNVQMG-LE
Gorilla (ENSGGOP00000034525)           -HLPQNQQQQQKLQIKNKEEILQTFPHPQSNNDQQREGSFFGQTKVEECFHGENQYSKS-----------------SEFDTHNVQMG-LE
Mouse (ENSMUSP00000143029)             FPQICQQQQQQQLQRKNKEQMPQTFSHLQGSNDKQREGSCFGQIKVEESFCVGNQYSKS-----------------SNFQTHNNTQGGLE
Rat (ENSRNOP00000048956)               FPQVCQQQH--QLQRKNKEQMPQTFSHLHGTNHEQSQGQCFGTIKAEESFCGENQYSKP-----------------SNFQTHS-AQGGLE
Pig (ENSSSCP00000009765)               -HLSQSQQQQQKLQMKNKEQMPQTFSHPQSNSDQQREGSFFSQIKVEECFHGENQFSKS-----------------SEYQTHNTQMG-LE
Tasmanian_devil (ENSSHAT000000         -PLTLN-HQQQKINGKNKEAGLQIFPHSHGSNEQQKERPSFGQMKREECLHSENQYSKS-----------------DEFPSHNTHVG-VE
Whale (XP_007106564.2)                 -HLSQNQQQQQKLQMKNKEQMPQTFSHPQGNNDQQREGSFFSQIKVEECFRGEGQHSKS-----------------SEFQTHNPQVG-LE
Elephant (XP_023404313.1)              -HLPQNQQQQQTLQMKNKEQMPQTFSHPQSNSDQRREGSFFGQIKVEECIHGENQHLKS-----------------SEFQTHNTQIG-LE
Lizard (ENSACAP00000008438)            -ITMSSQ--QQALQMKNRD-LPQTFSHSHSSQEQQLEGTSFSQFKLEE-FETANNNFKS-----------------SEFPMQNPQTR-LD
Chicken (ENSGALP00000017216)           -QLTPNQ--QQALQMKSKE-PPQTIPHSQSNAEQQPDRTSFSQPKADECFQTGNKYMKP-----------------TAFPLHSPQQG-LE
Turtle (ENSPSIP00000011691)            -QLTPNHHPQPALQMKSKE-LSQPFSQLQSNNEQRLDGVSYAQFKPEERFQTETTCIKT-----------------TEFPP-TPQLG-LE
Xenopus (ENSXETP00000030770)           ------HFLQAQPSEYIEELLKQHFDQSLRNCNQNRD-SPLNHFKLEEGAQAANQYQKH-----------------HPYPQQFEMEP---
Coelacanth (ENSLACP00000008576         QQQPQKSHQQALLQAINSIKLPQNLKQTQGNFELQCDGKSFNQLKLEEFVRLEKQYKMS-----------------NQFSSHTPQLQ-QE
Amazon_molly (ENSPFOP000000120         LSPMQSEHLHKDKDLQEILSVEFLTTQQQ-HCHLQRPLSHPPQFEAQQLKSPTYRPR------------------------SQPPPGQHQ
Amazon_molly (ENSPFOP000000299         LSPMQSEHLHKDKDLQEILSVEFLTTQQQ-HCHLQRPLSHPPQFEAQQLKSPTYRPR------------------------SQPPPGQHQ
Fugu (XP_011611191.1)                  ---MQSEHLCDDPDLRDILSAGFSATPQQQHCHLQRPLSHPPQFDDQQLKSPDYRPR------------------------SQPQPGHHQ
Medaka (XP_004065962.1)                YFQKDSQHLSDDTDLQDILSAG-LLTQQQQHCKLQRPLSHPPQFEEQQLKSPHYRPH------------------------SQPPPEQHL
Platyfish (ENSXMAP00000005447)          LSPMESEHFHEDKDLQEILSVEFLTTQQQ-HCHLQRPLSHPPQFEAQQLKSPTYRPR------------------------SQPPPGQHQ
Spotted _gar (ENSLOCP000000155         EGECNSTQEIKQKYLSNFLQQQQQQPPQDRTEEHQVLNTLQQHPSQDTSSIGTGQPKDSWKLEEFIR---LEKGLKSSDCKSQPPQVQQQ
Stickleback (ENSGACP0000002318         ------------------------------------------------------------------------------------------
Tilapia (XP_005457058.1)               HSQMQSERLCEDNDLQEILSPGFLATQQQQHCQLQRPMSQPPQFEAQQLKSPGYRPH------------------------SQPQPGQQL
Tetraodon (ENSTNIP00000021201)         ------------------------------------------------------------------------------------------
Zebrafish (ENSDARP00000101295)         --TFSNQLPQDRQNMQASPLSRDASRSQEVEPLLKKLKTEDYLHSEFQMLPACIKTP------------------------AQVQISSKA
Salmon (XP_014042431.1)                ------------------------------------------------------------------------------------------
Salmon (XP_014065146.1)                QGTMDGQQLN-KLKLEDYIRLEKKLKSPGGGYRGQGGQPQPGHQQQIQSMMRNNPGY------------------------NPVDPSQPN
Salmon (XP_014050290.1)                QGTMDGQQLN-KLKLEDYIRLEKKLKSPAGGYRGQGGQPQPGHQQQIQSMMRNNPGY------------------------NPVDPSQPN
Rainbow trout (XP_021429026.1)         ------------------------------------------------------------------------------------------
Rainbow trout (XP_021429023.1)         QGTMDGQQLLNKLKLEDYIRLEKKLKSPGGGYRGQGGQPQPGHQQQIQSMMRNNPGY------------------------NPVDPSQPN
Shark (XP_007910136.1)                 ------------------------------------------------------------------------------------------
Human (ENSP00000386869)                GTEKAAPGIKPSV----RKPIQIKKSRPREAQPLFPPVRQIVLEGLRSPAS---QEVQAH-----------------PPAPLPASQGS--
Gorilla (ENSGGOP00000051172)           GTEKAAPGIKPSV----RKPIQIKKSRPREAQPLFPPVRQIVLEGLRSPAS---QEVQAH-----------------PPAPLPASQGS--
Armadillo (ENSDNOP00000021516)         GTEKATSGVKSGV----RKPVQIKKSKPREAQPLFPPLRQIVLEGLRSPAA---EEAQAQ-----------------PPAPPPALQGS--
Mouse (ENSMUSP00000087049)             GAEKTTPGIKTSV----RKPIQIKKSRSRDMQPLFLPVRQIVLEGLKPQASEGQAPLPAQ-----------------LSVPPPASQGAAS
Rat (ENSRNOP00000015296)Tet3           GAEKTTPGIKASV----RKPIQIKKSRSRDMQPLFLPVRQIILEGLKPQASEGQAPTPAQ-----------------LPVPPPASQGAAS
Pig (ENSSSCP00000008852)               GTDKAGPGIKPGV----RKPIQIKKSRPREAQPLFLPVRQIVLEGLRSPAS---EDAQAQ-----------------TPTPVPASQGS--
Elephant  (XP_010591957.2)              GTEKAAPGIKPTV----RKPVQIKKSKLRDTQPLFPPLRQIVLEGLRSPAP---EEVQAH-----------------PPAPLPTSQGS--
Whale (XP_023989264.1)                 GTDKAGPGIKPSV----RKSIHIKKSRPREAQPLFPPLRQIILEGLRSPAS---EDVQAH-----------------PPAPVPASQGS--
Tasmanian_devil (ENSSHAP000000         GGEKRALGLKTVT----RKIVQIKKPKVRDSHPRFLPVRQINLEVLRSPTP---GAPKAG-----------------VTPPLPACRGA--
Chicken (ENSGALT00000059756)           --------PDKPA----RKQVQIKKPKQKDSQPLFLPFWQISLEGLRAPAE---PPASAP-----------------QSEPPPPTPLP--
Lizard (ENSACAT00000001585)            ----EKNSPAKPL----RKAVQIRRPKQKDVQPLFLPLAQISLEGYRSAVA---AAAIPE-----------------AASTPSATPST--
Turtle (ENSPSIT00000006549)            ----RQNFPGHPA----APSTPISG---------YSPACSAVPSGDSQDQA---PHKPLN-----------------PSDPLPAQNSV--
Xenopus (ENSXETP00000054061)           -SQKQVEPKPKPQ----RKQVQIKKPKVKEGSAVFMPVSQISLDTFRRVEK---EENQGKEMDAENSLPNNVQTELLESQSLQLTGSQ--
Coelacanth (ENSLACP00000009535         -KSQMQNTPQKLV----KKQVHIRKSKQKDCQAKFLPLRQISLAEYHLLEK---LKNSKE-----------------MQLPPLQTPRP--
Spotted_gar (ENSLOCP0000001981         QQ-ALLGLQPQMPQLTKPKQIIIKKPKQKASIPPFLPQFQITVQKPRKLELSSCIPESTST---------------PPAPTSQPSLSSDG
Amazon_molly (ENSPFOP000000021         VGGMAIGPALPKP-----KQIIIKKNKQKASMPTFLPQAQIAIQKPPVHMMDTSPSLTNSQ---------------ICPLNPLALLSNSA
Cave_fish (ENSAMXP00000015689)          QV--GGQFSPLGPPLPKPKPIIIKKPKQKASQPVFLPQNQITIQK-SLPSMPASMPSPSQL---------------TPSLPI--------
Fugu (ENSTRUP00000036865)              MS-----PPLPKP-----KQIIIKKTKQKASMPTFLPHAQISIQKPAL-LIDRAPGLTNLQ---------------PGFLPPQPLHCNST
Medaka (ENSORLP00000019236)            ------------------------------------------------------------------------------------------
Platyfish (ENSXMAT00000000130)         ------------------------------------------------------------------------------------------
Stickleback (ENSGACP0000001732         ------------------------------------------------------------------------------------------
Tetraodon (ENSTNIP00000019883)         ------------------------------------------------------------------------------------------
Tilapia (ENSONIP00000019943)           MTETLLGPPLPKP-----KQIIIKKPKQKASMPTFLPQTQITIQKPPVHMMDRAAPLAN-----------------------------LQ
Zebrafish (ENSDARP00000085229)         TP--GWVLEHTG-----------KLSNPESQQGVWSGQN--------------------------------------ASTTV--------
Rainbow_trout (XP_021462005.1)         Q---PGMLHYPGP---KPKTIVIKKTKQKASLPTFLPHSQISIQKPSPLTIIGALPLASAH---------------SGSLPFPTFPLLSN
Rainbow_trout (XP_021477599.1)         QHAGGGMLLQPGP---KPKTIVIKKTKQKVSLPTFLPHRQISIQKPSPLTITGALPLASAH---------------PGSLPLPTFPLLSN
Salmon (XP_014026412.1)                QQ--PGMLLYPGP---KPKTIVIKKTKRKASLPTFLPHSQISIQKPSPLTMIGALPLASAH---------------SGSLPFPTFPLLSN
Salmon (XP_014017100.1)                QHPGSGMLFQPGP---KPKTIVIKKTKQKVSLPIFLPHRQISIQKPSPLTITGALPLASAH---------------PGSLPLPTFPLLSN
```

```
                                         1450      1460      1470      1480      1490      1500      1510      1520      1530
                                    ....|....|....|....|....|....|....|....|....|....|....|....|....|....|....|....|....|....|
European ampioxus (Sc0000095)       ------------------------------------------------------------------------------------------
Ciona intestinalis (XP_0021254      SRCSTSSIISNEARSHNLPQLQATTCNGGPIHTSLRRAFTAPNFSSRRSPVSRSHSADCCTPDSDTGLDSTLETTKKKVLLRRYSSQGIG
Lamprey (scaf_00044)                ------------------------------------------------------------------------------------------
Lamprey (scaf_03335)                ------------------------------------------------------------------------------------------
Shark (XP_007897316.1)              QGHQLTQQPNQATSFRQQEQQCLGHLHQ--------QNQNLEKFRKYSDVQCQQQIQDANLNQEIHQQIQPESSEMQSYSNPPQMSQEKG
Lizard (XP_020663651.1)             DEEEVATQLTQLAAIIESSKTSPDQK----ASLFSRIPSDMQSKHKQDQCLLQKKQSVFIRNNYSSLLVKQRQPSRKNDKAVPRAKRLKK
Chicken (ENSGALP00000053457)        DEEDVATQLTQLAALIESNRANPAQKNDIKTSLLNLISHERQAKHNQD--LLQRKESVFFRHNCSSLLLKQKQSTNKKGNAVPWKPRHKK
Turtle (ENSPSIP00000006704)         DEEDVATQLTQLAAIIESNQTSPEQKNDVKTSLLSLLSHEIQPKHNQDQCLLKKKQSVFIRNNYSSLLVKQNQPTHKKGKTIPWKQRHKK
Human (ENSP00000362748)             IEEDVATQLTQLASIIKINYIKPEDKKV-ESTPTSLVTCNVQQKYNQEKGTIQQKPPSSVHNNHGSSLTKQKNPTQKKTKSTPSRDRRKK
Gorilla (ENSGGOP00000011536)        IEEDVATQLTQLASIIKINYIKPEDKKV-ENTPTSLVTCNVQQKYNQEKGTIQQKPPSSVHNNHGSSLTKQKNPTQKKTKSTPSRDRRKK
Mouse (ENSMUSP00000133279)          IEEDVAAQLTQLASTI--NHINAEVRNA-ESTPESLVAKNTKQKHSQEKRMVHQKPPSSTQTKPSVPSAKPKK-AQKKARATPHANKRKK
Rat (ENSRNOP00000054747)            IEEDVAAQLTQLASTI--NHINPEFRNA-ESTPESLVAKNTQQKHSQEKRMVQQKPPSSIQNKPIVPSTKPKK-TQKKARPTPYTSKRKK
Pig (ENSSSCP00000010918)            IEEDVATQLTQLASIIKFNYTKAEDKKV-ESTPN-LTAYNVQQNYCQK--TIQQKPPSSVQNNHGSSVTKQKNPTQKKTKSTPSRDRRKK
Whale (XP_023987950.1)              IEEDVAAQLTQLASIIKFNCIKPEDKKV-ESTPTSLVAYNVQQNYSQEKGTIEHKPPSSVQNNQGPSVTKQKNTIQKKTKSTPSRDRRKK
Elephant (ENSLAFP00000005179)       IEEDVATQLTQLASIIKFNYIKAENRRG-ESTPASLPEHNAQEK-----SIIRQKPPPSVQNNHGSSLTKQKNTSQKKTKPTPPRDRRKK
Armadillo (ENSDNOP00000014565)      IEEDVATQLTQLASIIKFNYIKPEDKKC-ENTPTSLVAHALQQKYRQEKGMIPQKPPSSVQNNYSSSLTKQKNTSQKKIKSTPSKERRKK
Tasmanian_devil (XP_023360087.      IEEDVAAQLTQLASIIESNNAKREEKEG-AKMFPSLECHNAQQKSKQEQSSFQEKPPVPIQNNHSSLLAKQKSSSHKRGKSASWKQRLKK
Coelacanth (ENSLACP00000016889      VAFQELSNQKKKPQYKLQN-IWIQQLNK--------FSQFLQQDLKSTKIKKLPKTKVQKVLPRKKPHLFLPQTQIQFHKYLPKPTQENI
Cavefish(XP_007240334.2)            DEEEVAAQLVQLAFIIQSRTNPMSS-------------ENSPPKGMPVQTIKYN------HHVVGQNVKKQRKPRTTPSSPRISKKKASE
Platyfish (XP_014325204.1)          DEEEVAAQLADLAFIIQARHNQQ--------------SDNDPPKGTPVSAINYN---YKSQQSPSQKKPPLKKIKTTPSKPRKRK-SDGL
Tilapia (XP_005456583.2)            DEEEVAAQLANLAFIIQSQHNQQ--------------SENNPLKGAPVSAIKYN---YKSQQPPSQKTPVRKKTKATPSKPRKKK-TDGI
Spotted_gar (XP_015202470.1)        DEEDVAAQLAQLAFIIESSHKKGQVNSSGVDETSTAQLAPHTPQGFPLQPIKNN----HHSLLQGLSTSPLKKTRTPPSKQRTKKAQLQK
Stickleback (ENSGACT0000001035      ------------------------------------------------------------------------------------------
Tetraodon (ENSTNIT00000011701)      ------------------------------------------------------------------------------------------
Fugu (XP_011601624.1)               DEVEVAAQLADLAFIIQSRHSQH--------------SENSPPKGTPVSAIKYN---YNSQLTASQKKNNAKKTKATPSKPRKKK-SDGL
Zebrafish (ENSDARP00000143362)      DEEEVAAQLVQLAFMIESRQAQVS--------------ENSPPNGMATQSLKYN------QNYLGQHLKKQRKPKMTPSNPRMSKKKVAE
Amazon_molly (XP_016534180.1)       DEEEVAAQLAELAFIIQARHNQQ--------------SDNGPPKGTPVSAIKYN---YKSQQSPCQKKPPLKKTKTTPSKPRKKK-SDGL
Medaka (XP_023818916.1)             DEEEVAAQLADLAFIIQSRHNQQ--------------SENNPPKGTPVSAIKYNNNHKLQLQSLSQKNPLARKQKSTPSKPRKRK-SDGL
Rainbow_trout (XP_021469805.1)      DEEEVAAQLAELAFIIQSRHKQQ-GFDP------SQHSENNPPRGTPVSAIKYN--YNSEQAPPNHKKTPVKKPRTTLSKPRKKKGTEGL
Rainbow trout (XP_021436521.1)      DEEEVAAQLAELAFIIQSRHKQQ-GFQP------SQHSENNPPRGTPVSAIKYN--YNSQQAPPNNKKIPVRKPSSTPSKPRKKKGMEGV
Salmon (XP_014010485)               DEEEVAAQLAELAFIIQSRHKQQQGFHP------SQHSENNPPRGTPVSAIKYN--YNSEQAPPNHKKTPVKKPRTTLSKPRKKKGTEGL
Salmon (XP_014070638.1)             DEEEVAAQLAELAFIIQSRHKQQ-GFQP------SQHSENNPPRGTPVSAIKYN--YNSQHAPPNNKKIPVRKPRSTPSKPRKKKGMEGV
Shark (XP_007890120.1)              QPYGIFANSSIHRKRIETQFSKLVPAYCHNIETIPEKADTFYRSENTITQDLHKPNNDLHQVPNDQQDSVQSQSQEQVQQLQQQAHHMSV
Human (ENSP00000425443)              EVQNINRRNSPYSQTMKSSACKIQVSCSNNTHLVSENKEQTTHPELFAGNKTQNLHHMQYFPNNVIP--KQDLLHRCFQEQEQKSQQAS-
Gorilla (ENSGGOP00000034525)        EVQNINSRNSPYSQTMKSSACKIQVSCSNNTHLVSENKEQTIHPELFAGNKTQNLHHMQYFPNNVIP--KQDLLHRCFQEQEQKSQQAS-
Mouse (ENSMUSP00000143029)          QVQNINK-NFPYSKILTPNSSNLQILPSNDTHPACE-REQALHP---VGSKTSNLQNMQYFPNNVTP--NQD-VHRCFQEQAQKPQQAS-
Rat (ENSRNOP00000048956)            QVQNVNR-SFPYAKIETPNSSKVQTLCSNYTHPASESREQNLHLDLFIGNRTPNQQNTQCFPNNVTP--SQDVAHRCFQEQEQKPHQAS-
Pig (ENSSSCP00000009765)            QVQNMNSRKSPYGQILKSNGSKVQISCSNSTHIVPENKEQTVNSELVAGNKTPNLHHMQYFPNNVIP--KQDILHRCFQEQEQKPQQAS-
Tasmanian_devil (ENSSHAT000000      QVHNISSKNSSYGQMLKSN-INTQLSCQNNIYLASERKENTNNSEFFIGNKTQDLQHIQYFPNNTTS--KPGVSHRCFQEQAHNLQQASP
Whale (XP_007106564.2)              QVQNMNSINSPYDQILKSNASKVQISCSNNIHVVPENKEQTVNSELFAGNKIPNLHHMQYFPNNVTP--KQDVLHRCFQQLEQKPQQAS-
Elephant (XP_023404313.1)           QRQNMNSRNALYGQILKSNTSNVQLSCGNNTHLVPEKKEQTINTELFAGNKTQNLHHMQYFPNNVTP--KQDVSHRCFQEQEQKPQQAS-
Lizard (ENSACAP00000008438)         HGSGINN----HAFKVNTI---EKHSYPNNMHLIPEKREKLMNTELFAENMTHDLQHMQYYSNSMTP--KQDTSQ-CFQDQKPQSPLGSA
Chicken (ENSGALP00000017216)        QVQSMNNKTPLYSQKTSTG---LQHPCPNNVHLMSEKTENAANLEHFGANKARDLQHVQYFSNNLPP--KQDVNH-CFQEQEQQTQQASV
Turtle (ENSPSIP00000011691)         QEQSINNKTSLYNQILKTN---THLQRPGHVHLVSEKKENTISAELLGLKKRQDLQYMQYYPNSMAP--KQDVQH-RFQEQEHQTQQASA
Xenopus (ENSXETP00000030770)        -KQSLSNMNSPFSQIQENNTVNPNHSYPDKQIYLSQKTEN---SCGFAENKPDPLQYMQYYQANSTE-------------QQEIS-----
Coelacanth (ENSLACP00000008576      ELHNPYNKNSPYKETSETT----AMTSYNNMHFASEKTESTMNHELHTFNK-QELQHMQYHPHSSAPKPQQKTSHMALIQGKQQLQQAP-
Amazon_molly (ENSPFOP000000120      PQPDQPFSNNH----QHTDQSAFTYNN-------TEMQQPQHQRQCLENSGSQNLKQFQPQQPKNHCHEPNQVNL--AQTSSQSQPHLP-
Amazon_molly (ENSPFOP000000299      PQPDQPFSNNH----QHTDQSAFTYNN-------TEMQQPQHQRQCLENSGSQNLKQFQPQQPKNHCHEPNQVDL--AQTSSQSQPHLP-
Fugu (XP_011611191.1)               VQPNQLLQNSS----GQSNNPLFSYNTS------PEIQQQQRLYSPIPNSGTNNLKQFQPQRPNNQCHQPDHADI--SQTSTQSQPHLP-
Medaka (XP_004065962.1)             PQLSQSLGNNL----AQANNS--QHSNHATFMYSNKAEMQQQQRQYLPN-----LKQFQPQLPNNHCHKHNKMDF--TQTSTQSQPHLP-
Platyfish (ENSXMAP00000005447)      LQPDQPFSNDH----QHTDQSAFAYNN-------TEMQQLQHQRQCLENSGSQNLKQFQPQQPNNHCHEPNQVDL--AQTSSQSQPHLP-
Spotted _gar (ENSLOCP000000155      TATLSPSTNTPGFLDSSSPSAQPQQNDSSHFTYGKTNNTVMHRHCKFSNAMEIQQSQYCPNPAAPKQYVEQQRIREQSNHRNVSQLPPP-
Stickleback (ENSGACP0000002318      ------------------------------------------------------------------------------------------
Tilapia (XP_005457058.1)            LQSNQPFGNGP----AQSSNQHVPQNDHATFSNINTVEMPQHQRQYPPNSGSTNLKQFQPQQPKSHCHQPNHVDF--PPNSTQSQPHLP-
Tetraodon (ENSTNIP00000021201)      ------------------------------------------------------------------------------------------
Zebrafish (ENSDARP00000101295)      IRENFGFQDSS----KSNTPSLFKEVEA------LTERKLQQNTECSQNISTGQQIYQQPLQHRIYN------------HSEFSQSLIS-
Salmon (XP_014042431.1)             ------------------------------------------------------------------------------------------
Salmon (XP_014065146.1)             DAQQSQQASWR----HSSSNTMEMEMELEMKQQQQQQHQQQQQRQYTPSPGPKQYSHQQQPPPHLRQPHPNHMDFPLTQTPPQPLPHLP-
Salmon (XP_014050290.1)             DAQQSQQASWR----QSSSNTMEMEMELEMKQQQQQQHQQQQQRQYTPSPGPKQYSHQQPPPPHLRQPHPNHMDFPLTQTPPQPLPHLP-
Rainbow trout (XP_021429026.1)      ------------------------------------------------------------------------------------------
Rainbow trout (XP_021429023.1)      DAQQSQQASWR----HSSSNTMEMEMELEMKQQQQQQHQQQQQRQYTPSPGPKQYSHQQQQPPHLRQPHPNHMDFPLTQTPPQPLPHLP-
Shark (XP_007910136.1)              ------------------------------------------------------------------------------------------
Human (ENSP00000386869)             ---AVPLPPEPSLALFAPSPSRDS-----------------------LLPPTQEMRSPSPMTALQPGSTG-PLPPAD-------------
Gorilla (ENSGGOP00000051172)        ---AVPLPPEPSLALFAPSPSRDS-----------------------LLPPTQEMRSPSPMTTLQPGSTG-PLPPAD-------------
Armadillo (ENSDNOP00000021516)      ---AVSLPPEPSLALFAPSPSGDS-----------------------LLPPTQEMRSPSPTATLQPGSAG-PLPPAD-------------
Mouse (ENSMUSP00000087049)          QSCATPLTPEPSLALFAPSPSGDS-----------------------LLPPTQEMRSPSPMVALQSGSTGGPLPPAD-------------
Rat (ENSRNOP00000015296)Tet3        QSCATPLPPEPSLALFAPSPSGDS-----------------------LLPPTQEMRSPSPMVALQSGSTGGPLPPAD-------------
Pig (ENSSSCP00000008852)            ---AVPQPPEPSLALFAPSPSGDS-----------------------LLPPTQEMRSPSPMATLQPGSAG-PLPPAD-------------
Elephant  (XP_010591957.2)          ---AVPQPPEPSLALFAPSPSGDS-----------------------LLPPTQETRSPSPMATLQAVSTG-PLPPAD-------------
Whale (XP_023989264.1)              ---AVPLPPEPSLALFAPSPSGDS-----------------------LLAPTQEMRSPSPMATLQPGSAG-PLPPAD-------------
Tasmanian_devil (ENSSHAP000000      ---SVPAAPAPPHALFAPGPSGGS-----------------------LLPPTQEMACPSLAAALQS-SPTVPLGPAD-------------
Chicken (ENSGALT00000059756)        ---PTAPDSQERGTPGGDTHNHPPAPGPEG-----------------AAPAVVDDKLEELIRQFEAEFGDTFSLPP--------------
Lizard (ENSACAT00000001585)         ---PTPADRSPLQKLLENRPNTQETPDRGGQGLCP------------ENPACPSAVAPATLGPSDPSPFSQNPLPV--------------
Turtle (ENSPSIT00000006549)         ---IVDDKLEELIRQFEAEFGENFSLQNTD----L------------PAPPLADGLPPQALSHQVSSLSHLPELP---------------
Xenopus (ENSXETP00000054061)        ---ANPDDRKTVNTQEMCNENQSNIGKANNFALCVNRANSFVAKDQCPTPSTHDTSSSSGQGDSANQHTNVSDVPGQNDLSCL-------
Coelacanth (ENSLACP00000009535      ---DLSDNAIGQPSPLLPAPHSTP-----------------------QHLPQTQLTSHQALSTQEAFSLN-TPCPQ--------------
Spotted_gar (ENSLOCP0000001981      LSLNSEGRTAQASSGLNIPEEPLNSTSLS----------------------------------------------------------ASG
Amazon_molly (ENSPFOP000000021      QASAAG-LPAPEQSQVCISNTSPTPSSNVTT--------------ENVPAVTDPSSTSVVLSAISKSEEAGISLTSTNTSNATPVTSTAP
Cave_fish (ENSAMXP00000015689)      -MEVAMSQAAPAQTQESIFNSSSTP--------------------MSGNTPPISTPASVPAP--------------------------HA
Fugu (ENSTRUP00000036865)           QAAAAG-LPAPAQSQVSISNSPTTSSILSAE--------------GTPTLVGPPPSPVVTGNAQSAGAGGQSSSNTSTTTSLT-STSSSS
Medaka (ENSORLP00000019236)         ------------------------------------------------------------------------------------------
Platyfish (ENSXMAT00000000130)      ------------------------------------------------------------------------------------------
Stickleback (ENSGACP0000001732      ------------------------------------------------------------------------------------------
Tetraodon (ENSTNIP00000019883)      ------------------------------------------------------------------------------------------
Tilapia (ENSONIP00000019943)        AAAAAG-LPAPAQSQLG------------------------------------PLLPPVGLAAHNKSEEMGS-LASSNTSTMTPMTSTSS
```

```
Zebrafish (ENSDARP00000085229)     -NDVDMEDAQTLVAFS------------------------------VGSLPPYTNHQPSPTP--------------------------TA
Rainbow_trout (XP_021462005.1)     PTQAAAGLPTPAQSQVSILNSSIARSVTAPA--------------FSVNSPAVSTPLPLPAAPDAPASSGEAGAT------------QTS
Rainbow_trout (XP_021477599.1)     PTQAAAGLPAPAQSQVSILNSSIAPSVAA----------------LSVNTPDVSTPQPLPVALDIPASSAEASAT------------QTS
Salmon (XP_014026412.1)            PTQAAAGLPTPAQSQVSILNSSIARSVTAPA--------------FSVNSPAVSTPLPLPAAPDAPASSGEAGAT------------QTS
Salmon (XP_014017100.1)            PTQAAAGLPAPAQSQVSILNSSIAPPVAAST--------------LSVNTPDVLTPQPLTVALNIPASSAEAIATQA-------STPQTS

                                            1540      1550      1560      1570      1580      1590      1600      1610      1620
                                   ....|....|....|....|....|....|....|....|....|....|....|....|....|....|....|....|....|....|
European ampioxus (Sc0000095)      ------------------------------------------------------------------------------------------
Ciona intestinalis (XP_0021254     ALENIVRAVTPQPSSTNTLPTQRLPLPPNHISRILPNFIPATKCEPFPLPTQFPTP----------------------------------
Lamprey (scaf_00044)               ------------------------------------------------------------------------------------------
Lamprey (scaf_03335)               ------------------------------------------------------------------------------------------
Shark (XP_007897316.1)             EVMQS-----VSQEKQCFPTDSLPSNSFRTHSPHSP-SMSSSQCTAKETDTPKWTCAETVLPCSCPNEVNSYNQSILTK----VKSLKQS
Lizard (XP_020663651.1)            KPQPI-----PDQQ-NQLQQE---CQHSEVQEMQKKPSKLRKLGRIVSQDSKLTSLGKKSSKTKVQKTRKWSTLPSQQKPTALFLPKTQI
Chicken (ENSGALP00000053457)       KPQEA-----RYQQINRNQLELLSCQHSELQDIWIS-SKLHKLGQAMPQDSRIALAEKKSPRTKVQKALNQNALTLQEK-PRLFLPQTQI
Turtle (ENSPSIP00000006704)        KSQVP-----DYQQNNQKQQEQLAYQHSELQDIWIS-SKP--LGRTMPRDLKIAASEKKSSRTKVQKALNQNTSSSQQK-TRLFLPQTQI
Human (ENSP00000362748)            KPTVV-----SYQENDRQKWEKLSYMYGTICDIWIA-SKFQNFGQFCPHDFPTVFG-KISSSTKIWKPLAQTRSIMQPK--TVFPPLTQI
Gorilla (ENSGGOP00000011536)       KPTVV-----SYQENDRQKWEKLSYMYGTICDIWIA-SKFQNFGQFCPHDFPTVFG-KISSSTKIWKPLAQTRSIMQPK--TVFPPLTQI
Mouse (ENSMUSP00000133279)         KPPAR-----SSQENDQKKQEQLAIEYSKMHDIWMS-SKFQRFGQSSPRSFPVLLR-NIPVFNQILKPVTQSKTPSQHN--ELFPPINQI
Rat (ENSRNOP00000054747)           KAPAH-----SSQESDQKKQERLAIEYSKMHDIWIS-SQFQRFGQSGPRSLPVLLR-NIPIFDQILKPVTQSKTSSHPN--KLFPPINQI
Pig (ENSSSCP00000010918)           KPAVI-----SCQENDQKKQEQLSYEYSKLHDIWIA-SKFQRFGQFGPHNFPILLG-KIPPLTKVWKPLTQKRSALEHK--TLFPPLNQI
Whale (XP_023987950.1)             KPVII-----SCQENDQKKQEQLAYEYSKLHDIWIA-SKFQRFGQFGPHDFPILLG-KIPPLTKVWKPLTQRSSALEHK--KLFPPLTQI
Elephant (ENSLAFP00000005179)      KPAIL-----SCEEIDRKKQEQLAEEYRKLYEIWVA-SKFQRYGQFVPRCFPILLG-KIPPVTKVRKPLTQKSSAFQHK--KLFPPLAQI
Armadillo (ENSDNOP00000014565)     KPAVI-----SCQENDQK-QEQLSYEYSKLHDIWIA-SKFQRFGQFGPHDFPILLG-KIPPLTKVWKPLTQTSSALQHK--KLFPPLAQI
Tasmanian_devil (XP_023360087.     KPPAK-----SIQQNDQSERERLAWQYSKFHDLWVG-SKLRRLGYVSSREFHATMIERSPSLTKVPKPLTQSPLSSQHK--KRFLPLTQI
Coelacanth (ENSLACP00000016889     KDLQP-----ELDKPDCLSPNHLKS----------------------KTDFP---------------EHSRLQGSIVTN----TIPLQPL
Cavefish(XP_007240334.2)           VEG----------GNHRIPLAKRTPNSKPPFKTKAQHVALQEK-PKPQPKWSPFLPQTQIDLKKYLAQAHHEKHQLFHFSS-----KRQD
Platyfish (XP_014325204.1)         QEG----------ASRRT-----AQGGETPHRSRGQNGLPHGK-SSRQPKNNFFLPLAQIDLKKYLAEAQEGRRQLIHQSN----IHSAS
Tilapia (XP_005456583.2)           NQR----------TPNR----------ETAYRSRNQKNPFQGK-SGLHHKKNLFLPQAQIDLKRYLAEAQEERKQFIHQINTQNIFHLGH
Spotted_gar (XP_015202470.1)       LEDTNGHLWGQSPANHVGHFPKRGANTKAPQRTKVQKVFPQRQ-MVQNHKSPLFPPQTQIDLKSYLAEVSQDKQALIYQNNPQDGVKSEL
Stickleback (ENSGACT0000001035     ------------------------------------------------------------------------------------------
Tetraodon (ENSTNIT00000011701)     ------------------------------------------------------------------------------------------
Fugu (XP_011601624.1)              QEG----------LNCRMSLSKRMPNGEVAHKGRGKKNLTQGR-SGVHQKKNLFLPLTQIDLKRYRTEAQEERRHLIQPTP-----FMGQ
Zebrafish (ENSDARP00000143362)     IDD----------NHCRILLAKKTPNKKTPLKATIQKAISQHK-MRFQHKRNPFLPQAQIDLKKYIAEAQHENRQLLYYNT-----KKEH
Amazon_molly (XP_016534180.1)      QEG----------ASRRT-----GQGGETPHRSRGQNGLPHGK-SSRQPKNNFFLPLAQIDLKKYLAEAQEGRRQLIHQSN----IHNTS
Medaka (XP_023818916.1)            QEP----------HNRRTPLSKRMPNGEIPHRGKGHKSLQQGK-SGPHLKNNLFLPQAQIDLKKYLAAAQEERSNVHGAS------LFGQ
Rainbow_trout (XP_021469805.1)     EEEGC-------KPNNRTPLYKRIPNGKTPHRARVQKVLPQPKTSSLSHKRSLFLPQAQMDLKRYLTEAQEERRQLFYYSN----AHSGV
Rainbow trout (XP_021436521.1)     EEESY-------NPNNKIPMSKRTPNGKTPHRARVQKVLPQPKTLSLSHKRSLFLPQTQMDLKRYLAEAQEEQRQLFYYSN----SHSGV
Salmon (XP_014010485)              EEEGY-------KLNNRTPLYKRIPNGKTPHRARVQKVLPQPKTSSLSHKRSLFLPQAQMDLKRYLTEAQEERRQLFYYSN----AHSGV
Salmon (XP_014070638.1)            EEEGY-------NPNNKTPLSKRTPNGKTPHRARVQKVLPQPKTLSLSHKRSLFLPQTQMDLKRYLAEAQEEQRQLFYYSN----SHSGV
Shark (XP_007890120.1)             IQQTQQRLMHQQHSQIQHGLSQQDTSHKMEQRNAAPHNRHAVPMKGTFLNPRYRQYPPSIQNEEEFKKHAALNSHLLQQQERKMYQQRMQ
Human (ENSP00000425443)            -------VLQGYKNRNQDMSGQQAAQLAQQRYLIHNHANVFPVPDQGGSHTQTPPQ-------KDTQKHAALRWHLLQKQEQQQTQQPQT
Gorilla (ENSGGOP00000034525)       -------VLQGYKNRNQDMSGQQAAQLAQQRYLIHNHANVFPVPDQGGSHTQTPPQ-------KDIQKHAALRWHLLQKQEQQQTQQPQT
Mouse (ENSMUSP00000143029)         -------SLQGLKDRSQGESPAPPAEAAQQRYLVHNEAKALPVPEQGGSQTQTPPQ-------KDTQKHAALRWLLLQKQEQQQTQQSQP
Rat (ENSRNOP00000048956)           -------SLQGLKGRSRDEFSVPPAEATQARYLVHIQAKALPVPKQGGSHTQISPQ-------KDTQKHAALRRLLLQKQEQQQIQQSQS
Pig (ENSSSCP00000009765)           -------VLQGYKSRNQDMSGQQAAQIAQQRHVMQNQANTFPVPDQAGSHIQTLPQ-------KDIHKHAALRWHLLQKQEQQQTQQLQA
Tasmanian_devil (ENSSHAT000000     HQQLPPQQPQGYHNKNQDLPSKQITQDLQQRYSLHHQPAALALPEQRANHIQSLPQ-------KDFQKHAALRWHLLQRQEQLQAQQAKT
Whale (XP_007106564.2)             -------VLQGYKNRNQDMSSQQAAQLAQQRYLMQNQANAFPLPSQAGSHIQTLPQ-------KDIQKHAALRWHLLQKQEQQQTQQLQA
Elephant (XP_023404313.1)          -------VLQGYKSRNQDMSSQQAAQLAQQRYLMQNQANAFPVPDQGGSHIQTPPQ-------KDIQKHAALRWHLLQRQEQQHT-QLQT
Lizard (ENSACAP00000008438)        PQRPFQQTQEYP-NFTSNMCSQQATQIQPS-YLPYSQQTVSNAIDQRECQITSQPH-------RDFQKHAALRWHILNKQEQQLNQNKSD
Chicken (ENSGALP00000017216)       IQLPQG----YGGSLSQDPPCQQAAPMPQR-YLPHSQQTPAHSQDQRGCHLQSQAP-------KDFHKHAALRWHLLQKQEQQAYQQPKT
Turtle (ENSPSIP00000011691)        MQLPLQQPERCGGNLKQDLPGQQATPIQQR-YLPHSQH--ALPQDQRGCHPLSQTH-------KDFQNHAALRWHLLQKQEQHMHQQPKP
Xenopus (ENSXETP00000030770)       -------------NYEHDLS---ARIAKQTHCLPESFSSSPHLHDKMGYYSQFENQ------VDCIPKHAALRNHLLQRSR---------
Coelacanth (ENSLACP00000008576     ----------VLGGLNFKNSSPQATQMQQQRLVAASQQPPPVQQESQVSNQQTPLQN-----QKDFQKHAALRWHLLQKQEHQTYQQKVN
Amazon_molly (ENSPFOP000000120     -----------QGLFSQQS---GTRMYPKAEEQARASCAQFQTGPRLPLGPASPH--------ADFQRHAALRMRLLQRQERHGPSQLPQ
Amazon_molly (ENSPFOP000000299     -----------QGLFSQQP---GTRMYPKAEEQARASCAQFQTGPRLPLGPASPH--------ADFQRHAALRMRLLQRQERHGPSQLPQ
Fugu (XP_011611191.1)              -----------QNMQNQQL---STQIFPKAEQKL--SCTQFQRGPQLPLRPTGPH--------GSFQRHTALRTHLLQKQERQGPPHHLQ
Medaka (XP_004065962.1)            -----------QGALNQQV---RNQMHPKAEPQLKSSCTQFQRGPQLPLGSMGPQ--------TNFQRNAAIRMHLLQKQELQN-PLHPL
Platyfish (ENSXMAP00000005447)      -----------QGLFSQQS---GTRMYPKAEEQTRASCTQFQTGPRLPLGPAGPH--------VDFQRHAALRMRLLQRQERHGPSQLPR
Spotted _gar (ENSLOCP000000155     -----------STQRQPQQGALNQHILGLMTAQMYPKTESQDSCAQAQQGQNQNHP-------GDLQKHAILRMHLLQKQEKQAYQQN--
Stickleback (ENSGACP0000002318     ------------------------------------------------------------------------------------------
Tilapia (XP_005457058.1)           -----------QGALSQQV---TKQMYPKAEQQLKTSCTQFQRGPQLPLGPAGPH--------VDFQRHAALRMHLLQKQERQGSPQTPQ
Tetraodon (ENSTNIP00000021201)     ------------------------------------------------------------------------------------------
Zebrafish (ENSDARP00000101295)     -----------QSQHSQASDGLQIPVRTLTKAEFHESFAQIQRE---SLSSNGAQ--------MDVQRHAALRMHLLQRQERQS------
Salmon (XP_014042431.1)            ------------------------------------------------------------------------------------------
Salmon (XP_014065146.1)            -----------HGALNQQVSAQQQQMYPKMEQNQQESCAQFQRGGPPPLGHGGPQ--------GDFQRHAALRMHLLQRQSERGSLGPPH
Salmon (XP_014050290.1)            -----------HGALNQQVSAQQQQMYPKMEQNQQESCAQFQRGGPPPLGHGGPQ--------GDFQRHAALRMHLLQRQSERGSLGHTH
Rainbow trout (XP_021429026.1)     ------------------------------------------------------------------------------------------
Rainbow trout (XP_021429023.1)     -----------HGALNQQVSAQQQQIYPKMEQNQQESCAQFQRGGPPPLGHGGPQ--------GDFQRHAALRMHLLQRQSERESLGPPH
Shark (XP_007910136.1)             ------------------------------------------------------------------------------------------
Human (ENSP00000386869)            -------------DKLEELIRQFEAEFGDSFGLPGPPSVPIQDPENQQT-----------------------------------------
Gorilla (ENSGGOP00000051172)       -------------DKLEELIRQFEAEFGDSFGLPGPPSVPIQDPENQQT-----------------------------------------
Armadillo (ENSDNOP00000021516)     -------------DKLEELIRQFEAEFGDSFGLPGPPSVPIQDPENQPP-----------------------------------------
Mouse (ENSMUSP00000087049)         -------------DKLEELIRQFEAEFGDSFGLPGPPSVPIQEPENQST-----------------------------------------
Rat (ENSRNOP00000015296)Tet3       -------------DKLEELIRQFEAEFGDSFGLPGPPSVPIQDPENQST-----------------------------------------
Pig (ENSSSCP00000008852)           -------------DKLEELIRQFEAEFGDSFGLPGPPSVPIQDPENQPT-----------------------------------------
Elephant  (XP_010591957.2)         -------------DKLEELIRQFEAEFGESFGLPGPPSVPIQDSENQPT-----------------------------------------
Whale (XP_023989264.1)             -------------DKLEELIRQFEAEFGDNFGLPGPQSVPIQDPENQPT-----------------------------------------
Tasmanian_devil (ENSSHAP000000     -------------DKLEELFQQFEAEFGDNFGLAAAPPAPACSPEQLAS-----------------------------------------
Chicken (ENSGALT00000059756)       -------------SGATALPEGPPPAPQVTAAVAPSSAAPGGSALSPG------------------------------------------
Lizard (ENSACAT00000001585)        -------------ENLDELIQQFEAQFGDSFGFAGPDGKDRGSPQGPRDP----------------------------------------
Turtle (ENSPSIT00000006549)        -------------RGSDSTLQVPAAEDGGPPLADAPEVGPKGFSPSPK------------------------------------------
Xenopus (ENSXETP00000054061)       ------------DDKLEDLIRQFEAEFGEDFSLPGSAVPSQNGEGPPKQTPSGDPQFKLPFPSQLLPPENSTKPATHSNPALSNNPVSRE
Coelacanth (ENSLACP00000009535     -------------DKFEELIREFEAKFGGSPSPQ-HSQFPAQSNIGPQE-----------------------------------------
Spotted_gar (ENSLOCP0000001981     ISSSAPDGLNSIDDKFEELIRQFEAELEDRVAVPS-------------------------QTQTQIQTKAQAPVTIRAPVQEPQ------
Amazon_molly (ENSPFOP000000021     SNTSPLQKLINIDPKYEELIRQFEAEFGNLAPDTSTSPRKEETTPAPT------------LANQFLISPQDLSLASSNTTQSVSSVPTPQ
Cave_fish (ENSAMXP00000015689)     QPTPPLAGLGSLDPKFEDLIRQFEEEFGDPLPSASV----------ADAPGQGPQQQHRILADPFTMS----------------------
Fugu (ENSTRUP00000036865)          TSNSQLPSLVNVDPKYEELIRQFEAEFGESHTPHSKSLKSSPTCPNPTPTPNPDPNDQMEVGKDESGIQCETAPVGGRNGGTQEGSLQVS
```

```
Medaka (ENSORLP00000019236)          ------------------------------------------------------------------------------------------
Platyfish (ENSXMAT00000000130)       -----------------------------------------------------------------------MCVCKPYPVLSLS------
Stickleback (ENSGACP0000001732       ------------------------------------------------------------------------------------------
Tetraodon (ENSTNIP00000019883)       ------------------------------------------------------------------------------------------
Tilapia (ENSONIP00000019943)         PSTSNVPSVINLDPKYEELIRQFEAEFGDSVPDISASQPVEETATAPN------------MPASPLSKRMKIESSGDITVVSTT------
Zebrafish (ENSDARP00000085229)       Q-----------------------------------------------------------------------------------------
Rainbow_trout (XP_021462005.1)       TTSQSIPGLSSLDPKFEELILQFEAEFGDSSSSAPVPCPPQIQ--PQDQSPSASAQPVVIESQPNITSGQARTASPSS-TATQSCSPTPL
Rainbow_trout (XP_021477599.1)       TTSQSIPGLSSLDPKFEELICQFEAEFGDSSSSAPEPCPSQIQSQPQDQAPSASAQPVVTESQPKVNSGQARPPSPSSATLTQSSSAAPL
Salmon (XP_014026412.1)              TTSQSIPGLSSLDPKFEELILQFEAEFGDSSSSAPVPCPPQIQ--PQDQSPSASAQPVVIESQPNITSGQARPTSPSSTTNTQSCSPTPL
Salmon (XP_014017100.1)              TISQSIPGLSSLDPKFEELICQFEAEFGDSSSSAPEPCPSQIQSQPQDQAPSASAQPVVTEPHPKVNSGQARPPSPSSATITQSSSAAPL

                                              1630      1640      1650      1660      1670      1680      1690      1700      1710
                                     ....|....|....|....|....|....|....|....|....|....|....|....|....|....|....|....|....|....|
European ampioxus (Sc0000095)        ------------------------------------------------------------------------------------------
Ciona intestinalis (XP_0021254       ------------------------------------------------------------------------------------------
Lamprey (scaf_00044)                 ------------------------------------------------------------------------------------------
Lamprey (scaf_03335)                 ------------------------------------------------------------------------------------------
Shark (XP_007897316.1)               KSNSDNISELQAQYNCISQVR---------------------------------------------PSGETTHREDVLENPNKQDAEKGQ
Lizard (XP_020663651.1)              IFRRPLPVLVQDR-MKDKLFGREMVPEPIRTMGSREAQGTSPPSGAPVALPPSGLLSHDLLTVCHLEAKSCLNQGSENVQMFTEKDKNSQ
Chicken (ENSGALP00000053457)         KFHR-LSEVPQEK-KKDRLFV------------------------------------------SQLKTASLLTRPSENTQMFTEKCN-SQ
Turtle (ENSPSIP00000006704)          KIHRYLPEIPWEK-TKDRLFG------------------------------------------CEVVELCS-NRPSENVQMFTEKYNNSQ
Human (ENSP00000362748)              KLQR-YPESAEEK-VKVEPLD---------------------------------------------SLSLFHLKTESNGKAFTDKAYNSQ
Gorilla (ENSGGOP00000011536)         KLQR-YPESAEEK-VKVEPLD---------------------------------------------SLSLFHLKTESNGKAFTDKAYNSQ
Mouse (ENSMUSP00000133279)           KFTR-NPELAKEK-VKVEPSD---------------------------------------------SLPTCQFKTESGGQTFAEPADNSQ
Rat (ENSRNOP00000054747)             KFTR-NPELAKET-VKVEPSD---------------------------------------------SLPTCQFKMESSGQAFSEPADNSQ
Pig (ENSSSCP00000010918)             KVDRYYPELAQDK-MKVEPLD---------------------------------------------SFPIIQLKTESSGQAFTENAY-PQ
Whale (XP_023987950.1)               KFERYYPELAQEK-MKVEPLD---------------------------------------------SLPIIQLKAESNGQAFTENGY-AQ
Elephant (ENSLAFP00000005179)        KFERYYPELVQEKVVKVEPLD---------------------------------------------SFSLSQFKTESNGQAFTDKAYSSQ
Armadillo (ENSDNOP00000014565)       KFERYYPELAQEKIIKVEPLD---------------------------------------------SFSVSPFKTEFNGQVFTEKAYNSQ
Tasmanian_devil (XP_023360087.       KFHRHLPESAQEK-MKEKRFGFEEMADHTKSEEVDPPTGRVVPEG----LQQSGGSSTDPFSLFHLHTKPPLSRPSDNVQVFTEKGDNSQ
Coelacanth (ENSLACP00000016889       EAN------------------------------------------------------------------MAVNGSDQNEIPS--------
Cavefish(XP_007240334.2)             LDMQTLKSSGNQKAFTYKTDHAALSHSHVNQFRS---------------------------------NGHCHSLTNGHLGATHGQKYECE
Platyfish (XP_014325204.1)           LSQNPN--NLLTTNHTGARENHQWSLPNG-----------------------------------------LPLQHNQCNGFIAGP---TE
Tilapia (XP_005456583.2)             QTQNYS--TLATINHISGQENQAWSLSKG-----------------------------------------PLHQHNPCNGYAAGPE-EYE
Spotted_gar (XP_015202470.1)         LEHNSLLQSVSCKNSGLPPSNDSSELHPDWN--------------------------------------TDGHLIQKSEHLEGSLRQKTE
Stickleback (ENSGACT0000001035       ------------------------------------------------------------------------------------------
Tetraodon (ENSTNIT00000011701)       ------------------------------------------------------------------------------------------
Fugu (XP_011601624.1)                QIQNHS--IIRTN------HTPSWCLSNG-----------------------------------------PLNRLDPCTSPAAELGRECE
Zebrafish (ENSDARP00000143362)       L--ETFIGSGRQNGFHVKYEHGGHSLPPQ--------------------------------------NGHFYNHTNDHLGASQCPRHECE
Amazon_molly (XP_016534180.1)        LSQNPN--NLFTTNHTGAGENHQWSLPNG-----------------------------------------LPLPHNQCNGFVAGP---AE
Medaka (XP_023818916.1)              QTLNLS--TLTKSNHDSGQENHPWSFSNG-----------------------------------------PLHKHNTCNGYAAGP-----
Rainbow_trout (XP_021469805.1)       KYHKTSGAVDRGRSQGYGHDNQQWSHFN--------------------------------------------GHLDPLQGQGVGQGHDCE
Rainbow trout (XP_021436521.1)       KYHKTSGAVSRG--QGYGHDNQQWSHLNGHH------------------------------------NGPLNGHLDPLQGQGVGQGHECE
Salmon (XP_014010485)                KYHKTSGAVDRGRSQGYGHDNQQWSHFN--------------------------------------------GHLDPLQGQGVGQGHDCE
Salmon (XP_014070638.1)              KYHKTSGAVDRGQCQGYGHDNQQWSHLNGDH------------------------------------SGPLNGHLDPLQGQGVGQGHECE
Shark (XP_007890120.1)               A-----FKDLQKPIKVEEGLPIPS-------------------------------------------------------SMLHLQSELEV
Human (ENSP00000425443)               E---SCHSQMHRPIKVEPGCKPHA---------------------------------------------------CMH--TAPPENKTWK
Gorilla (ENSGGOP00000034525)         E---SCHSQMHRPIKVEPGCKPHA---------------------------------------------------CMH--TAPPENKTWK
Mouse (ENSMUSP00000143029)           -----GHNQMLRPIKTEPVSKPSS---------------------------------------------------YRYPLSPPQEN--MS
Rat (ENSRNOP00000048956)             E---SGHHQMPRPIKIEPGSKLSS---------------------------------------------------CKHPTSGPQEN--MP
Pig (ENSSSCP00000009765)             G---SCHEQMHRSIKVEPGSRPHA---------------------------------------------------CMHPMSAQPENKMWK
Tasmanian_devil (ENSSHAT000000       E---TCHRQMLKPIKVEAGAKPNA---------------------------------------------------CMSSLAGPSEIKPWK
Whale (XP_007106564.2)               E---SCHSQMQRPIKVEPGSKPHA---------------------------------------------------CMRLMSAQPENKMWK
Elephant (XP_023404313.1)            E---TCHNQMQRPIKVEPGSKPHA---------------------------------------------------CTRPMSAQPENKMCK
Lizard (ENSACAP00000008438)          T---CHIMGHNKPIKPEAGSKLNA---------------------------------------------------CLPQLAGQLENKTWK
Chicken (ENSGALP00000017216)         E---TGAGAARKPIKIEAGAKSNF---------------------------------------------------CMRLSAGQLENKMWK
Turtle (ENSPSIP00000011691)          E---ICASVMRKPIKVEAGSKSNV---------------------------------------------------CMHLSTGQLENKTWK
Xenopus (ENSXETP00000030770)         ------SVKADRLVKAEKSSHSNA---------------------------------------------------CMHTPMESKTKKNTD
Coelacanth (ENSLACP00000008576       -----SFNNLLQFIKSEESSQLQT---------------------------------------------------SMPQQCALQKNKIWK
Amazon_molly (ENSPFOP000000120       --SPVDSKQNLRAVKMENGPRFELPTSR-----------------------------------------------QQEQPFMTRDAGMGG
Amazon_molly (ENSPFOP000000299       --SPVDSKQNLGAVKMENGPRFELPTSR-----------------------------------------------QQEQPFMTRDAGMGG
Fugu (XP_011611191.1)                --SINDPRHNFGTMRMENKPRFDLPGAS-----------------------------------------------QQRQNLQMQESAMGG
Medaka (XP_004065962.1)              --QSSELNQSQRAIKMEP--RFELPTSQ-----------------------------------------------QQGQLLHMREPDKGG
Platyfish (ENSXMAP00000005447)       --SPVDSKQNLGAVKLENSPRFELPTSW-----------------------------------------------QQEQPFMARDAGMGG
Spotted _gar (ENSLOCP000000155       ---LEDFRHLLQSIKKENHPMSEP---------------------------------------------------PVPQTARDLTNSAVN
Stickleback (ENSGACP0000002318       ------------------------------------------------------------------------------------------
Tilapia (XP_005457058.1)             --NSSDPKHIPRAVKMENGPRFEISGFQ-----------------------------------------------NQEHQIHMREAGR--
Tetraodon (ENSTNIP00000021201)       ------------------------------------------------------------------------------------------
Zebrafish (ENSDARP00000101295)       ---PNNIRQMMPAIKSENCSGFEISAQP-----------------------------------------------MEVQRQHEDHTKVLK
Salmon (XP_014042431.1)              ------------------------------------------------------------------------------------------
Salmon (XP_014065146.1)              PQTPADFKRGVRPIKMVNGPRFEHPHHQGPGPPP---------------------------------------LMQHQQQGREMGMVAGG
Salmon (XP_014050290.1)              PQTPVDYKRGVRPIKMVNGPRFEHPHHQGPGPPP---------------------------------------LMQHQQQGREMGMVAGG
Rainbow trout (XP_021429026.1)       ------------------------------------------------------------------------------------------
Rainbow trout (XP_021429023.1)       PQTPVDFKHGVRPIKMVNGPRFEHPHHQGPGPPP---------------------------------------LMQHQQQGREMGMVAGG
Shark (XP_007910136.1)               ------------------------------------------------------------------------------------------
Human (ENSP00000386869)              CLPAPESPFA--------------------------------------------------------------------------------
Gorilla (ENSGGOP00000051172)         CLPAPESPFA--------------------------------------------------------------------------------
Armadillo (ENSDNOP00000021516)       CLPAPESLFA--------------------------------------------------------------------------------
Mouse (ENSMUSP00000087049)           CLPAPESPFA--------------------------------------------------------------------------------
Rat (ENSRNOP00000015296)Tet3         CLPTPESPFA--------------------------------------------------------------------------------
Pig (ENSSSCP00000008852)             CLPAPESPFA--------------------------------------------------------------------------------
Elephant  (XP_010591957.2)           CLPAPESPFA--------------------------------------------------------------------------------
Whale (XP_023989264.1)               CLPAPESPFA--------------------------------------------------------------------------------
Tasmanian_devil (ENSSHAP000000       CVLRPDSPFS--------------------------------------------------------------------------------
Chicken (ENSGALT00000059756)         KGPPPEHLFS--------------------------------------------------------------------------------
Lizard (ENSACAT00000001585)          RPFPFESPFG--------------------------------------------------------------------------------
Turtle (ENSPSIT00000006549)          MGQHFENPFG--------------------------------------------------------------------------------
Xenopus (ENSXETP00000054061)         VSNNLDSLFS--------------------------------------------------------------------------------
```

```
Coelacanth (ENSLACP00000009535   IPKEDEPSLS--------------------------------------------------------------------------------
Spotted_gar (ENSLOCP0000001981   ------------------------------------------------------------------------------------------
Amazon_molly (ENSPFOP000000021   ANTTPQSDDQKMEEALGMQD----------------------------------------------------------------------
Cave_fish (ENSAMXP00000015689)   ------------------------------------------------------------------------------------------
Fugu (ENSTRUP00000036865)        VVPEKQQQPGTLESMFSTPG----------------------------------------------------------------------
Medaka (ENSORLP00000019236)      ------------------------------------------------------------------------------------------
Platyfish (ENSXMAT00000000130)   ------------------------------------------------------------------------------------------
Stickleback (ENSGACP0000001732   ------------------------------------------------------------------------------------------
Tetraodon (ENSTNIP00000019883)   ------------------------------------------------------------------------------------------
Tilapia (ENSONIP00000019943)     ------------------------------------------------------------------------------------------
Zebrafish (ENSDARP00000085229)   ------------------------------------------------------------------------------------------
Rainbow_trout (XP_021462005.1)   ASTTAPPADQDMEVDKENVTRGVSEASYPSA-----------------------------------------------------------
Rainbow_trout (XP_021477599.1)   APTTAPPAD------KEESG---SMSKVTST-----------------------------------------------------------
Salmon (XP_014026412.1)          ASTTAPPADQDMEVDKENVTGGVSEASYTSA-----------------------------------------------------------
Salmon (XP_014017100.1)          APTTAPPADQDMEVDKEESG---GMSKVTST-----------------------------------------------------------

                                         1720      1730      1740      1750      1760      1770      1780      1790      1800
                                 ....|....|....|....|....|....|....|....|....|....|....|....|....|....|....|....|....|....|
European ampioxus (Sc0000095)    ------------------------------------------------------------------------------------------
Ciona intestinalis (XP_0021254   --------------------------------------------------SYPTPTFMPSFETPPRTPLGCTPRSTSYESTS--------
Lamprey (scaf_00044)             ------------------------------------------------------------------------------------------
Lamprey (scaf_03335)             ------------------------------------------------------------------------------------------
Shark (XP_007897316.1)           VDLE-------GDLPLNRPSTQQGQSNLRATDQQSASVSIEAQIAPTIHQ---SSSSSEGLSVFTSKSPRCIKVETSGCVTVLSTSNLNT
Lizard (XP_020663651.1)          VQQNKNITQ-MHPLPQIFS---KRTNETCKDSQAKFQQDVGEKLKGQNLL-AIPNSCDEAPQADNMQALGN--VECVSETKFPTS---RN
Chicken (ENSGALP00000053457)     VQQAVNVGQ-THPLPQTFSQSNLRAGETCGEDKAYVQQGAVDQRVDPKSQ-MLPVSCSAGQVPGTVEALRN--MECAGEVTILTS---RS
Turtle (ENSPSIP00000006704)      VQQTMNVIQ-MHPLPQTLNQSNQRAHEKSDEDQANFQQNATEQQLGQKLQ-TLPVSCCEAHLPHTVETLRN--IDCAGEITVVTS---TS
Human (ENSP00000362748)          VQLTVNANQKAHPLTQPSSPPNQCANVMAGDDQIRFQQVVKEQLMHQRLP-TLPGISHETPLPESALTLRNVNVVCSGGITVVSTK--SE
Gorilla (ENSGGOP00000011536)     VQLTVNANQKAHPLTQPSSPPNQCANVMAGDDQIRFQQVVKEQLMHQRLP-TLPGISHETPLPESALTLRNVNVVCSGGITVVSTK--SE
Mouse (ENSMUSP00000133279)       GQPMVSVNQEAHPLPQ-SPPSNQCANIMAGAAQTQFHLGAQENLVHQIPPPTLPGTSPDTLLPDPASILRKGKVLHFDGITVVTEK---R
Rat (ENSRNOP00000054747)         GQPMVNVDQKAHPLPQ-SPPSNQCA--IYGCAAQPXHL-ALERTVHQIHPPTWC-ISPDPLLPDPASALRKGNVLSSNGITVVTTK--HE
Pig (ENSSSCP00000010918)         VPPTVNVNQKVHPLLQPSSPTNQCANVMAGNDQTQFQQDVKDQHMHQRLP-TLPGISHETPLPDPAQILRNLNVVCSGGITVVSTK--SE
Whale (XP_023987950.1)           VQPTVNVNQKAHPLLRPTSPTNQCANGMAGNDQTQFQQDVKDQVTHQRLP-TLPGISHETPLPDPAQILRNLNVVCSGGITVVSTK--SE
Elephant (ENSLAFP00000005179)    VQPTGNVNEKAHPLSRPSSPTSQCVNLMAGDDQTRIQQDVNEQLMHQRLP-TLPGISHERPLSDPAQILRNVNVVCSGGITVVSTK--SE
Armadillo (ENSDNOP00000014565)   VQPTVNVDQKAHPLPQPSSPSNQCANVVAGDDQTQFPQDFKEQLIHQRLP-TLPGISHERSLPDPAQILRNVNVVCSGGITVVSTK--SE
Tasmanian_devil (XP_023360087.   VQHSVNVN-------QSLNQYNQRAGATAGVDQTQFQQDAEEQLRSQRQH-MLPAPSCATPVPGTVQTLRNESTACPGGASVAAAN--NS
Coelacanth (ENSLACP00000016889   ----------------------PGPVLVSEADRTSK------------------RNSFETPSSCTTRDPRHVKVETSGAITILSTSNLGA
Cavefish(XP_007240334.2)         EHLISQVSKTYSVLNHGANPQAQYAMPSVPGADP-----LPGSPGRCHKV----NSLSCEPRQTSFGQNGRYKVDTAGAVTVLSVSAGNM
Platyfish (XP_014325204.1)       SHVLSQVAQPVGELQHGTHPNTSPAKPAFISN----------VNGFHGLS----NGFSGPPDSPPPSQQNYYKLETSGSVTVLST----A
Tilapia (XP_005456583.2)         KHLLSQVVQPCGGLQHGADPNTSPAKSAFLSP----------TSGHHGLA----NGFTGARQSPPPSQQSYYKLESSGPVTVLST----A
Spotted_gar (XP_015202470.1)     CDSNTQVAQSLDGQQHGANLDTGSAKVQFYVQASQDRNEPPSSKIYSNES----KISSLGQLPPASTAEGCIKIETSGSVTVLSTSNLNS
Stickleback (ENSGACT0000001035   ------------------------------------------------------------------------------------------
Tetraodon (ENSTNIT00000011701)   ------------------------------------------------------------------------------------------
Fugu (XP_011601624.1)            KHLLSQVAQPCSRLHHSADLDTGPATAASLSH----------STGHHVLA----NGFSGAQLSPPPNQQSYYKLEKSGAVTILST----A
Zebrafish (ENSDARP00000143362)   QK-VSQVSKTYDVLNPGAKQQLLQTMANEPFKD------LPQIPEMYHKA----NGFNG-HQHLHTNQNGFYKVEKSGGVTVLSASSVHS
Amazon_molly (XP_016534180.1)    SHVLSQVAPPAGELQHGTHPIASPAKPAFTSN----------VNGFRVLS----NGFSGPPDSPPPSQQNYYKLETSGSVTVLST----A
Medaka (XP_023818916.1)          DDVSSQVGKPCGGLQN-----TSIEKTAFPSH----------STGHHSWA----NGFSRTPQSPPPSQQSYYKLERSGPVAVLST----A
Rainbow_trout (XP_021469805.1)   RGLYSQVSQPYVGQQHGANLKASSTIPSFTSID---------SNNHRTGL----NHGLSNGYSSGGQQQGYYKVERSGPVTVLST----S
Rainbow trout (XP_021436521.1)   RRLYSQVSQPYVWQQHGANLKASSTNPSFTSLD---------SNNHRTGL----NHSLPNGYSSGGQRQGYYKVERSGPVTILST----S
Salmon (XP_014010485)            RGLYSQVSQPYVGQQHGANLKSSSTIPSFTSID---------SNNHRTGL----NHGLSNGYSPGGQQQGYYKVERSGPVTVLST----S
Salmon (XP_014070638.1)          RQLYSQVSQPFVGQQHGANLKASSTNPSFTSLD---------SNNHRTGL----NNSLPNGYSSGGQRQGYYKVERSGPVTILST----S
Shark (XP_007890120.1)           NPIEHQSQHFNFEH-------QQQERSIIEALEQRFKQYQLSSPFERQTCVIKSPKQVKVETSGPVTVLSTTAHFESDMGGDCTSAQQTS
Human (ENSP00000425443)          KVTKQENPPASCD--------NVQQKSIIETMEQHLKQFHAKSLFDHKALTLKSQKQVKVEMSGPVTVLTRQTTAAELDSHTPALEQQTT
Gorilla (ENSGGOP00000034525)     KVTKQENPPASCD--------NVQQKSIIETMEQHLKQFQAKSLFDHKALTLKSQKQVKVEMSGPVTVLTRQTTAAELDSHTPALEQQTT
Mouse (ENSMUSP00000143029)       SRIKQEISSPSRD--------NGQPKSIIETMEQHLKQFQLKSLCDYKALTLKSQK--HVKVP-------TDIQAAESENHARAAEPQA-
Rat (ENSRNOP00000048956)         KRIKQESPLLSCD--------GGRTESIIETMERHLKQFQLKALCDYKILTLNSQRQGDVETA-------TNARSAGSENHTPAAEPQAS
Pig (ENSSSCP00000009765)         KIPKQEIPPPNCD--------NVQQKSILETMEQHLKQFQVKSLFDHKALTLKSQKQVKVEMSGPVTVLTRQTTAAELESHTPALEQQAM
Tasmanian_devil (ENSSHAT000000   KAAKQEVQHLGCD--------QSQQKSIIETMEQQLKQIQVKSLFDHKALTIKSQKHVKVEMAGPVTVLTRNTSASELDGHNPTSEQQAT
Whale (XP_007106564.2)           KIPKQEIPPPSCD--------NMQQRSILETMEQHLKQFQVKSLFDHKALTLKSQKQVKVEMSGPVTVLTRQTTAAELDSHTPALEQQAM
Elephant (XP_023404313.1)        KVTKQEIAPQSCD--------NVQQKSILETMEQHLKQFQVKSLFDHKAPPLRSQKQVKVEMSGPVTVLTRHTTAAELDSYTPALEQQAT
Lizard (ENSACAP00000008438)      KTIKQEVQNFSCE--------NMQQRSIIETMEQQLRQIQVKTPFDHKSLTIKSPKHIKMPFP-----LQPEQAQKYGTGDDPQLTHDSS
Chicken (ENSGALP00000017216)     KTIKQENQHFGCE--------NTQQKSIIETMEQQLKQIQVKSLFDHKTFTVKSPKHVKVETAGPITILSRNTSAAEFDTHTPILEQQAN
Turtle (ENSPSIP00000011691)      KIIKQENQHFGCE--------NLQQKSIIETMEQQLKQIQVKSLFDHKALTIKSPKHVKVETAGPITILSRNSTAAEFESHASALDHHAI
Xenopus (ENSXETP00000030770)     KKIKQETRHSSTE--------AGQQKSIIETMEQQMRHYPGKSSFHRQVSSIKSPKHVKVESSGPVTVLTTHTNFSTLDQQTVPPHEKT-
Coelacanth (ENSLACP00000008576   TL-KQESQKLSCE--------NLQQKSIIEAMEQQLKQFQVSSLFEHQAFALKSPKNVKVETSGPVTILSTNIAGK--DSSVSAPKSIPS
Amazon_molly (ENSPFOP000000120   VKIKTENQQSLCV-------QDRKPGNIIASMEQSLRQYELSNLFEKKPLVVNSSNKVKVESSGPVTILSTNMDMSG------ADS--TA
Amazon_molly (ENSPFOP000000299   VKIKTENQQSLCV-------QDRKPGNIIASMEQSLRQYELSNLFEKKPLVVNSSNKVKVESSGPVTILSTNMDMSG------ADS--AA
Fugu (XP_011611191.1)            IQIKQENQQFLCE-------QNKRQGSILASMEQNLRQYQLSPVFEKKSLVINSSNKVKVESSGPVTVLSTNTELGG------MEVS---
Medaka (XP_004065962.1)          LQVKQENHPSPCN-------HKKIPRSILASMEQSLRQYQLSTVFEQKSLLIHSSNKVKVESSGPVTILSTNTEVRD------VQSSIPA
Platyfish (ENSXMAP00000005447)    VKIKTESQQSLCV-------QDKKPGNILASMEQSLRQYKLSNLFEKKPLVN-SSNKVKVESSGAVTILSTNMDMSG------TDS--TP
Spotted _gar (ENSLOCP000000155   NMIKQEYSQMDCD--------QSRQKSIIATMEHQLKQYHPSPLFERKSLVIKSPKQVKVETSGPVTILSTNADLGN----------GDI
Stickleback (ENSGACP0000002318   ------------------------------------------------------------------------------------------
Tilapia (XP_005457058.1)         IQIKQENQQSLCE-------QSKKQGSIIASMEQSLRQYQLSPVFEKKSIVINALNKVKVESSGPVTILSTKTDVNG------VESSALA
Tetraodon (ENSTNIP00000021201)   ------------------------------------------------------------------------------------------
Zebrafish (ENSDARP00000101295)   AVVKQEHPPSSCD--------ENQQRSILATMEQQLKQYQLSPVFEKKSLVIKSPNKVKVEMAGAVTVISTNAEGNA-------------
Salmon (XP_014042431.1)          ------------------------------------------------------------------------------------------
Salmon (XP_014065146.1)          IQVKQEYNPQPQTSCCREQSQSSSQRSILATMEQTLRQYQLSPVFDRKSLVIRSPNKVKVEQSGAVTVLSTHADLDGGSGGMEDMGMGRP
Salmon (XP_014050290.1)          IQVKQEYNPQPQTSCCGEQSQSSSQRSILATMEQTLRQYQLSPVFDRKSLVIRSPNKVKVEQSGAVTVLSTHADLDGGLGGMEDMGMGRP
Rainbow trout (XP_021429026.1)   ------------------------------------------------------------------------------------------
Rainbow trout (XP_021429023.1)   IQVKQEYNPQPQTSCCGERSQSSSQRSILATMEQTLRQYQLSPVFDRKSLVIRSPNKVKVEQSGAVTVLSTHADLDGGSGGMEDMGMGRP
Shark (XP_007910136.1)           ------------------------------------------------------------------------------------------
Human (ENSP00000386869)          ---------------------------------------------------TRSPKQIKIESSGAVTVLSTTCFHSEEGGQ---------
Gorilla (ENSGGOP00000051172)     ---------------------------------------------------TRSPKQIKIESSGAVTVLSTTCFHSEEGGQ---------
Armadillo (ENSDNOP00000021516)   ---------------------------------------------------PRSPKQIKIESSGAVTVLSTTCFPSEEGSQ---------
Mouse (ENSMUSP00000087049)       ---------------------------------------------------TRSPKKIKIESSGAVTVLSTTCFHSEEGGQ---------
Rat (ENSRNOP00000015296)Tet3     ---------------------------------------------------TRSPKKIKIESSGAVTVLSTTCFHSEEGGQ---------
Pig (ENSSSCP00000008852)         ---------------------------------------------------TRSPKQIKIESSGAVTVLSTTCFHSEEGAQ---------
Elephant  (XP_010591957.2)       ---------------------------------------------------TRSPKQIKIESSGAVTVLSTTCFHPEEGGQ---------
Whale (XP_023989264.1)           ---------------------------------------------------TRSPKQIKIESSGAVTVLSTTCFHSEEGGQ---------
```

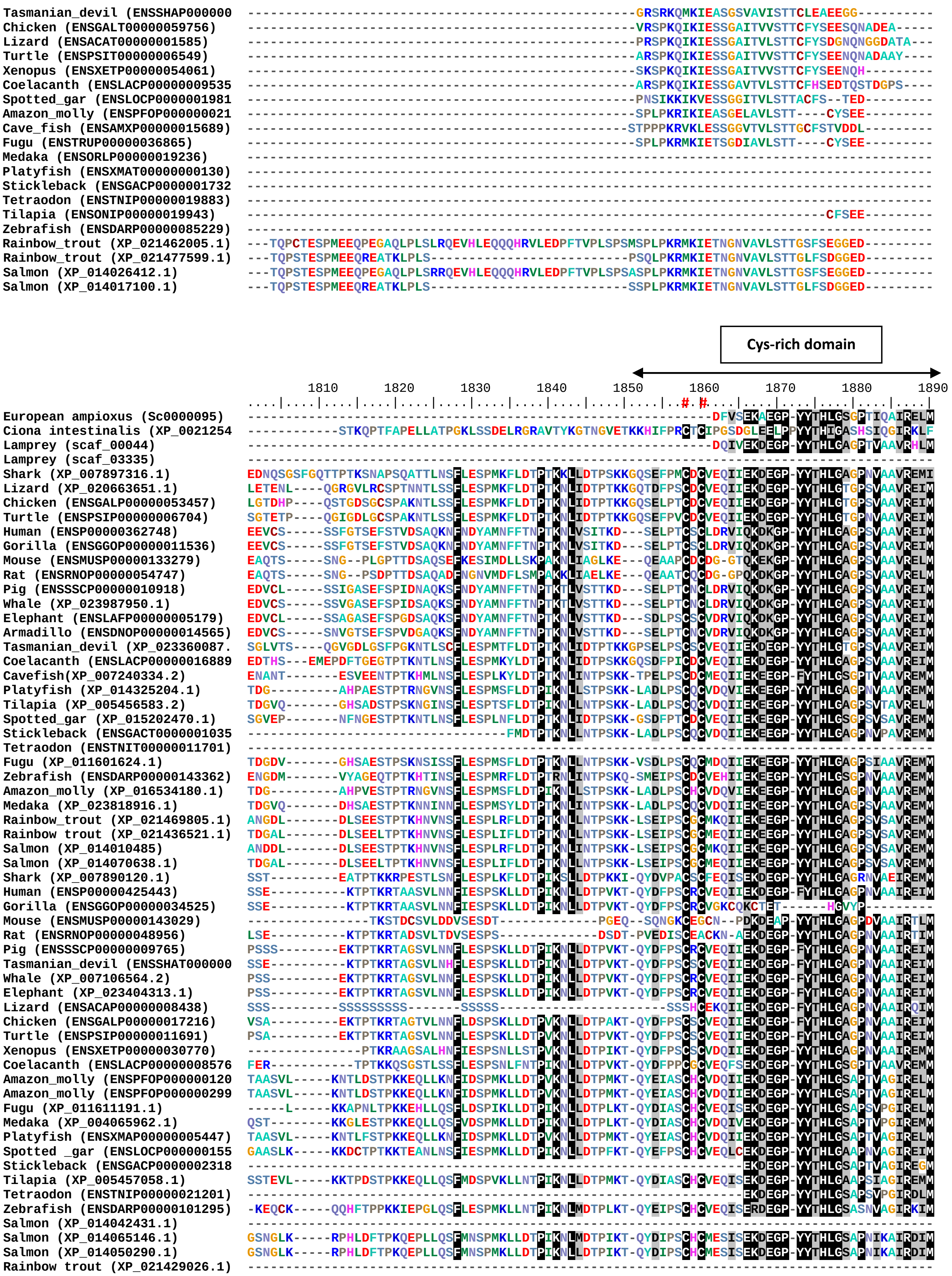
Tasmanian_devil (ENSSHAP000000
Chicken (ENSGALT00000059756)
Lizard (ENSACAT00000001585)
Turtle (ENSPSIT00000006549)
Xenopus (ENSXETP00000054061)
Coelacanth (ENSLACP00000009535
Spotted_gar (ENSLOCP0000001981
Amazon_molly (ENSPFOP000000021
Cave_fish (ENSAMXP00000015689)
Fugu (ENSTRUP00000036865)
Medaka (ENSORLP00000019236)
Platyfish (ENSXMAT00000000130)
Stickleback (ENSGACP0000001732
Tetraodon (ENSTNIP00000019883)
Tilapia (ENSONIP00000019943)
Zebrafish (ENSDARP00000085229)
Rainbow_trout (XP_021462005.1)
Rainbow_trout (XP_021477599.1)
Salmon (XP_014026412.1)
Salmon (XP_014017100.1)
Cys-rich domain
1810
1820
1830
1840
1850
1860
1870
1880
1890
European ampioxus (Sc0000095)
Ciona intestinalis (XP_0021254
Lamprey (scaf_00044)
Lamprey (scaf_03335)
Shark (XP_007897316.1)
Lizard (XP_020663651.1)
Chicken (ENSGALP00000053457)
Turtle (ENSPSIP00000006704)
Human (ENSP00000362748)
Gorilla (ENSGGOP00000011536)
Mouse (ENSMUSP00000133279)
Rat (ENSRNOP00000054747)
Pig (ENSSSCP00000010918)
Whale (XP_023987950.1)
Elephant (ENSLAFP00000005179)
Armadillo (ENSDNOP00000014565)
Tasmanian_devil (XP_023360087.
Coelacanth (ENSLACP00000016889
Cavefish(XP_007240334.2)
Platyfish (XP_014325204.1)
Tilapia (XP_005456583.2)
Spotted_gar (XP_015202470.1)
Stickleback (ENSGACT0000001035
Tetraodon (ENSTNIT00000011701)
Fugu (XP_011601624.1)
Zebrafish (ENSDARP00000143362)
Amazon_molly (XP_016534180.1)
Medaka (XP_023818916.1)
Rainbow_trout (XP_021469805.1)
Rainbow trout (XP_021436521.1)
Salmon (XP_014010485)
Salmon (XP_014070638.1)
Shark (XP_007890120.1)
Human (ENSP00000425443)
Gorilla (ENSGGOP00000034525)
Mouse (ENSMUSP00000143029)
Rat (ENSRNOP00000048956)
Pig (ENSSSCP00000009765)
Tasmanian_devil (ENSSHAT000000
Whale (XP_007106564.2)
Elephant (XP_023404313.1)
Lizard (ENSACAP00000008438)
Chicken (ENSGALP00000017216)
Turtle (ENSPSIP00000011691)
Xenopus (ENSXETP00000030770)
Coelacanth (ENSLACP00000008576
Amazon_molly (ENSPFOP000000120
Amazon_molly (ENSPFOP000000299
Fugu (XP_011611191.1)
Medaka (XP_004065962.1)
Platyfish (ENSXMAP00000005447)
Spotted _gar (ENSLOCP000000155
Stickleback (ENSGACP0000002318
Tilapia (XP_005457058.1)
Tetraodon (ENSTNIP00000021201)
Zebrafish (ENSDARP00000101295)
Salmon (XP_014042331.1)
Salmon (XP_014065146.1)
Salmon (XP_014050290.1)
Rainbow trout (XP_021429026.1)

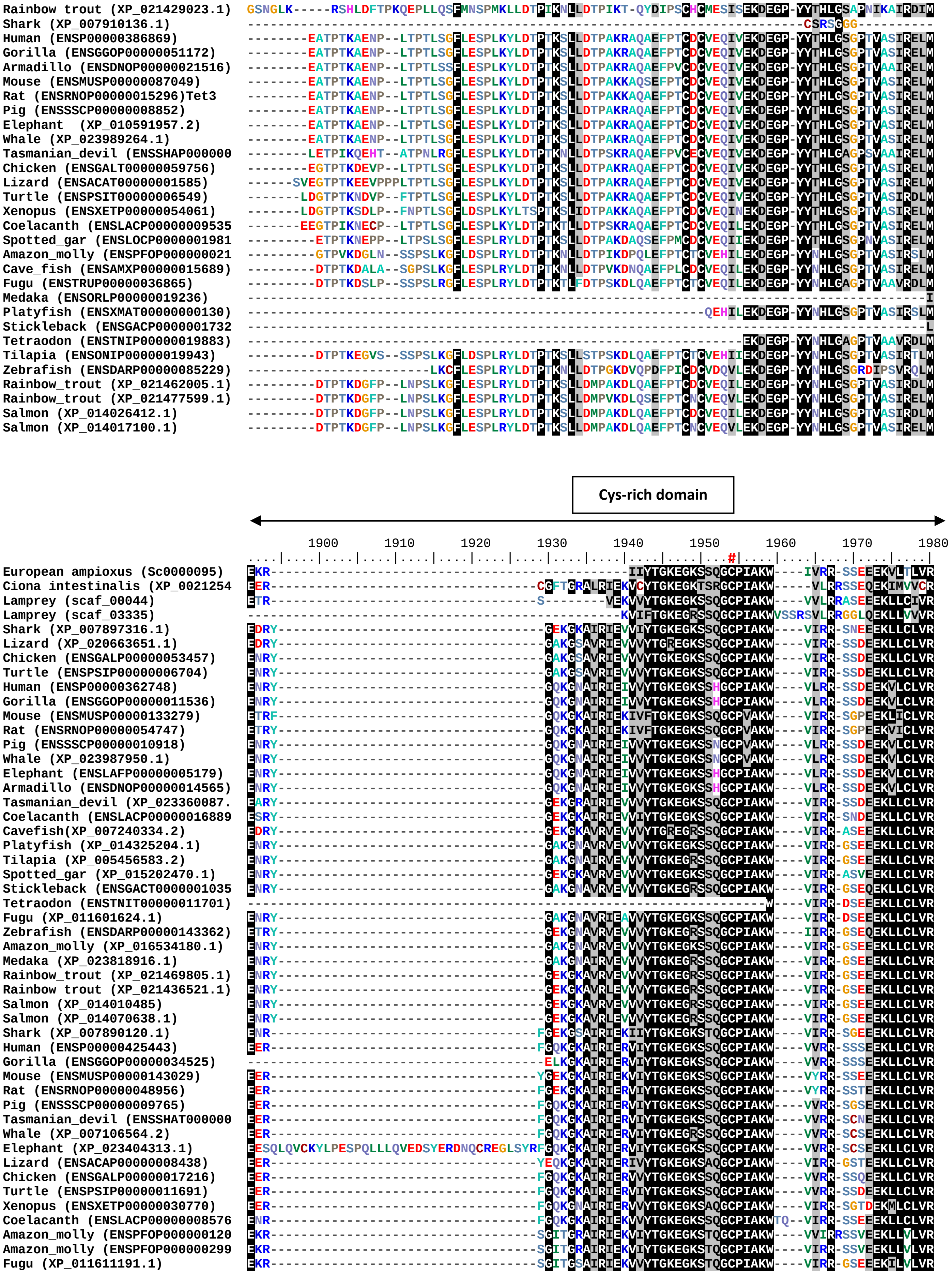
Rainbow trout (XP_021429023.1)
Shark (XP_007910136.1)
Human (ENSP00000386869)
Gorilla (ENSGGOP00000051172)
Armadillo (ENSDNOP00000021516)
Mouse (ENSMUSP00000087049)
Rat (ENSRNOP00000015296)Tet3
Pig (ENSSSCP00000008852)
Elephant (XP_010591957.2)
Whale (XP_023989264.1)
Tasmanian_devil (ENSSHAP000000
Chicken (ENSGALT00000059756)
Lizard (ENSACAT00000001585)
Turtle (ENSPSIT00000006549)
Xenopus (ENSXETP00000054061)
Coelacanth (ENSLACP00000009535
Spotted_gar (ENSLOCP0000001981
Amazon_molly (ENSPFOP000000021
Cave_fish (ENSAMXP00000015689)
Fugu (ENSTRUP00000036865)
Medaka (ENSORLP00000019236)
Platyfish (ENSXMAT00000000130)
Stickleback (ENSGACP0000001732
Tetraodon (ENSTNIP00000019883)
Tilapia (ENSONIP00000019943)
Zebrafish (ENSDARP00000085229)
Rainbow_trout (XP_021462005.1)
Rainbow_trout (XP_021477599.1)
Salmon (XP_014026412.1)
Salmon (XP_014017100.1)
Cys-rich domain
1900
1910
1920
1930
1940
1950
1960
1970
1980
European ampioxus (Sc0000095)
Ciona intestinalis (XP_0021254
Lamprey (scaf_00044)
Lamprey (scaf_03335)
Shark (XP_007897316.1)
Lizard (XP_020663651.1)
Chicken (ENSGALP00000053457)
Turtle (ENSPSIP00000006704)
Human (ENSP00000362748)
Gorilla (ENSGGOP00000011536)
Mouse (ENSMUSP00000133279)
Rat (ENSRNOP00000054747)
Pig (ENSSSCP00000010918)
Whale (XP_023987950.1)
Elephant (ENSLAFP00000005179)
Armadillo (ENSDNOP00000014565)
Tasmanian_devil (XP_023360087.
Coelacanth (ENSLACP00000016889
Cavefish(XP_007240334.2)
Platyfish (XP_014325204.1)
Tilapia (XP_005456583.2)
Spotted_gar (XP_015202470.1)
Stickleback (ENSGACT0000001035
Tetraodon (ENSTNIT00000011701)
Fugu (XP_011601624.1)
Zebrafish (ENSDARP00000143362)
Amazon_molly (XP_016534180.1)
Medaka (XP_023818916.1)
Rainbow_trout (XP_021469805.1)
Rainbow trout (XP_021436521.1)
Salmon (XP_014010485)
Salmon (XP_014070638.1)
Shark (XP_007890120.1)
Human (ENSP00000425443)
Gorilla (ENSGGOP00000034525)
Mouse (ENSMUSP00000143029)
Rat (ENSRNOP00000048956)
Pig (ENSSSCP00000009765)
Tasmanian_devil (ENSSHAT000000
Whale (XP_007106564.2)
Elephant (XP_023404313.1)
Lizard (ENSACAP00000008438)
Chicken (ENSGALP00000017216)
Turtle (ENSPSIP00000011691)
Xenopus (ENSXETP00000030770)
Coelacanth (ENSLACP00000008576
Amazon_molly (ENSPFOP000000120
Amazon_molly (ENSPFOP000000299
Fugu (XP_011611191.1)

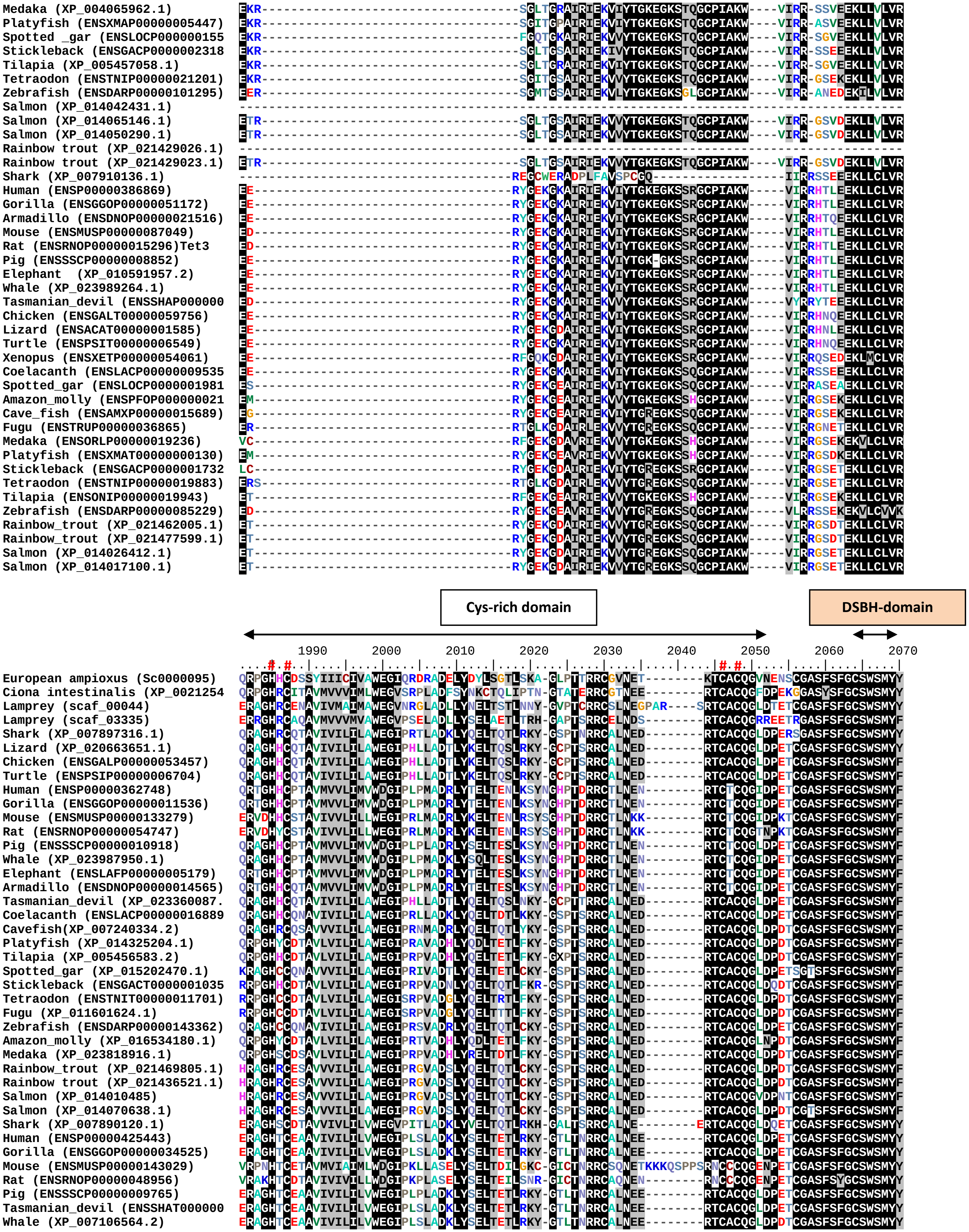
Medaka (XP_004065962.1)
Platyfish (ENSXMAP00000005447)
Spotted _gar (ENSLOCP000000155
Stickleback (ENSGACP0000002318
Tilapia (XP_005457058.1)
Tetraodon (ENSTNIP00000021201)
Zebrafish (ENSDARP00000101295)
Salmon (XP_014042431.1)
Salmon (XP_014065146.1)
Salmon (XP_014050290.1)
Rainbow trout (XP_021429026.1)
Rainbow trout (XP_021429023.1)
Shark (XP_007910136.1)
Human (ENSP00000386869)
Gorilla (ENSGGOP00000051172)
Armadillo (ENSDNOP00000021516)
Mouse (ENSMUSP00000087049)
Rat (ENSRNOP00000015296)Tet3
Pig (ENSSSCP00000008852)
Elephant (XP_010591957.2)
Whale (XP_023989264.1)
Tasmanian_devil (ENSSHAP000000
Chicken (ENSGALT00000059756)
Lizard (ENSACAT00000001585)
Turtle (ENSPSIT00000006549)
Xenopus (ENSXETP00000054061)
Coelacanth (ENSLACP00000009535
Spotted_gar (ENSLOCP0000001981
Amazon_molly (ENSPFOP000000021
Cave_fish (ENSAMXP00000015689)
Fugu (ENSTRUP00000036865)
Medaka (ENSORLP00000019236)
Platyfish (ENSXMAT00000000130)
Stickleback (ENSGACP0000001732
Tetraodon (ENSTNIP00000019883)
Tilapia (ENSONIP00000019943)
Zebrafish (ENSDARP00000085229)
Rainbow_trout (XP_021462005.1)
Rainbow_trout (XP_021477599.1)
Salmon (XP_014026412.1)
Salmon (XP_014017100.1)
Cys-rich domain
DSBH-domain
1990
2000
2010
2020
2030
2040
2050
2060
2070
European ampioxus (Sc0000095)
Ciona intestinalis (XP_0021254
Lamprey (scaf_00044)
Lamprey (scaf_03335)
Shark (XP_007897316.1)
Lizard (XP_020663651.1)
Chicken (ENSGALP00000053457)
Turtle (ENSPSIP00000006704)
Human (ENSP00000362748)
Gorilla (ENSGGOP00000011536)
Mouse (ENSMUSP00000133279)
Rat (ENSRNOP00000054747)
Pig (ENSSSCP00000010918)
Whale (XP_023987950.1)
Elephant (ENSLAFP00000005179)
Armadillo (ENSDNOP00000014565)
Tasmanian_devil (XP_023360087.
Coelacanth (ENSLACP00000016889
Cavefish(XP_007240334.2)
Platyfish (XP_014325204.1)
Tilapia (XP_005456583.2)
Spotted_gar (XP_015202470.1)
Stickleback (ENSGACT0000001035
Tetraodon (ENSTNIT00000011701)
Fugu (XP_011601624.1)
Zebrafish (ENSDARP00000143362)
Amazon_molly (XP_016534180.1)
Medaka (XP_023818916.1)
Rainbow_trout (XP_021469805.1)
Rainbow trout (XP_021436521.1)
Salmon (XP_014010485)
Salmon (XP_014070638.1)
Shark (XP_007890120.1)
Human (ENSP00000425443)
Gorilla (ENSGGOP00000034525)
Mouse (ENSMUSP00000143029)
Rat (ENSRNOP00000048956)
Pig (ENSSSCP00000009765)
Tasmanian_devil (ENSSHAT000000
Whale (XP_007106564.2)

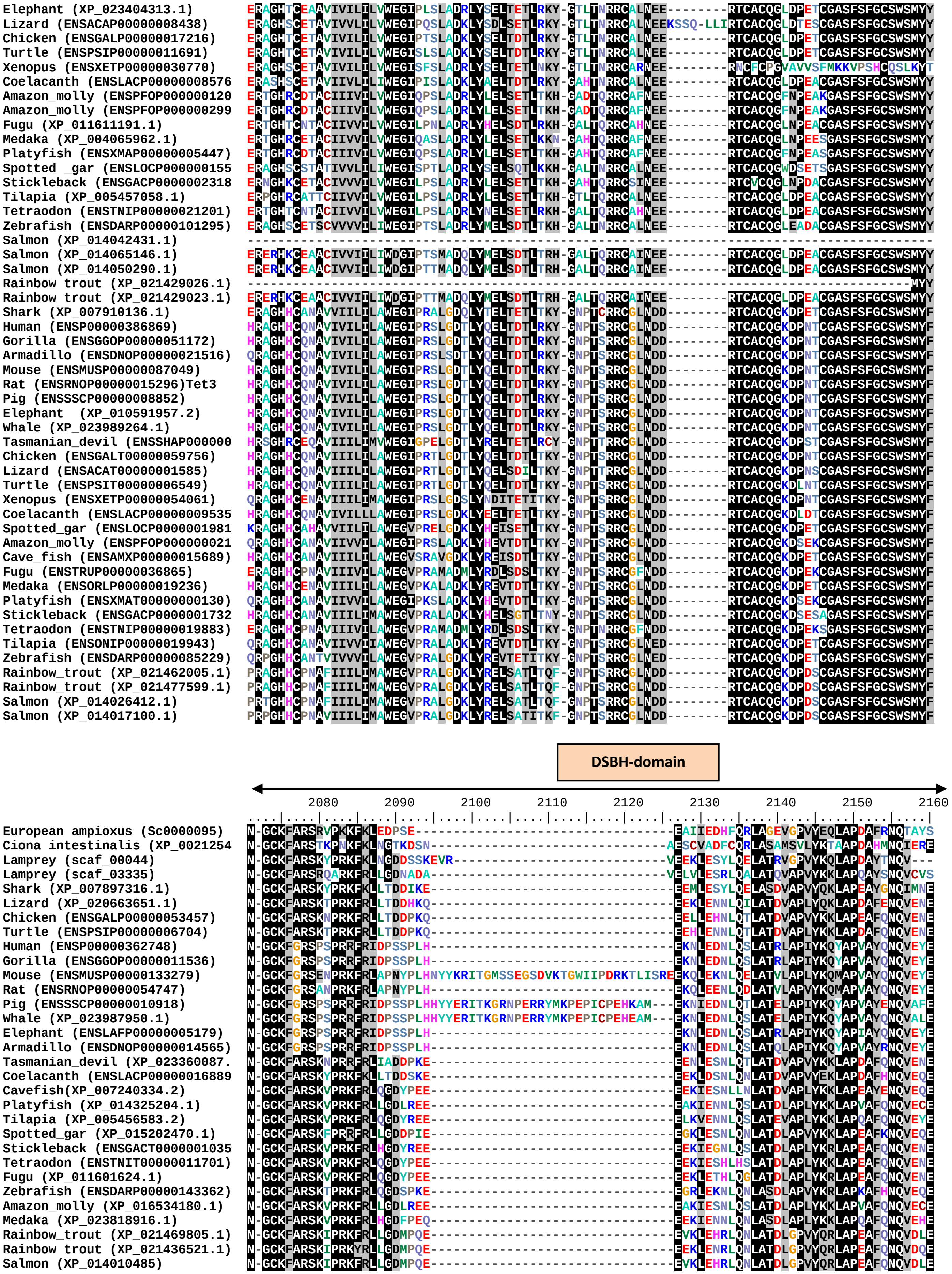
Elephant (XP_023404313.1)
Lizard (ENSACAP00000008438)
Chicken (ENSGALP00000017216)
Turtle (ENSPSIP00000011691)
Xenopus (ENSXETP00000030770)
Coelacanth (ENSLACP00000008576
Amazon_molly (ENSPFOP000000120
Amazon_molly (ENSPFOP000000299
Fugu (XP_011611191.1)
Medaka (XP_004065962.1)
Platyfish (ENSXMAP00000005447)
Spotted _gar (ENSLOCP000000155
Stickleback (ENSGACP0000002318
Tilapia (XP_005457058.1)
Tetraodon (ENSTNIP00000021201)
Zebrafish (ENSDARP00000101295)
Salmon (XP_014042431.1)
Salmon (XP_014065146.1)
Salmon (XP_014050290.1)
Rainbow trout (XP_021429026.1)
Rainbow trout (XP_021429023.1)
Shark (XP_007910136.1)
Human (ENSP00000386869)
Gorilla (ENSGGOP00000051172)
Armadillo (ENSDNOP00000021516)
Mouse (ENSMUSP00000087049)
Rat (ENSRNOP00000015296)Tet3
Pig (ENSSSCP00000008852)
Elephant (XP_010591957.2)
Whale (XP_023989264.1)
Tasmanian_devil (ENSSHAP000000
Chicken (ENSGALT00000059756)
Lizard (ENSACAT00000001585)
Turtle (ENSPSIT00000006549)
Xenopus (ENSXETP00000054061)
Coelacanth (ENSLACP00000009535
Spotted_gar (ENSLOCP0000001981
Amazon_molly (ENSPFOP000000021
Cave_fish (ENSAMXP00000015689)
Fugu (ENSTRUP00000036865)
Medaka (ENSORLP00000019236)
Platyfish (ENSXMAT00000000130)
Stickleback (ENSGACP0000001732
Tetraodon (ENSTNIP00000019883)
Tilapia (ENSONIP00000019943)
Zebrafish (ENSDARP00000085229)
Rainbow_trout (XP_021462005.1)
Rainbow_trout (XP_021477599.1)
Salmon (XP_014026412.1)
Salmon (XP_014017100.1)
DSBH-domain
2080
2090
2100
2110
2120
2130
2140
2150
2160
European ampioxus (Sc0000095)
Ciona intestinalis (XP_0021254
Lamprey (scaf_00044)
Lamprey (scaf_03335)
Shark (XP_007897316.1)
Lizard (XP_020663651.1)
Chicken (ENSGALP00000053457)
Turtle (ENSPSIP00000006704)
Human (ENSP00000362748)
Gorilla (ENSGGOP00000011536)
Mouse (ENSMUSP00000133279)
Rat (ENSRNOP00000054747)
Pig (ENSSSCP00000010918)
Whale (XP_023987950.1)
Elephant (ENSLAFP00000005179)
Armadillo (ENSDNOP00000014565)
Tasmanian_devil (XP_023360087.
Coelacanth (ENSLACP00000016889
Cavefish(XP_007240334.2)
Platyfish (XP_014325204.1)
Tilapia (XP_005456583.2)
Spotted_gar (XP_015202470.1)
Stickleback (ENSGACT0000001035
Tetraodon (ENSTNIT00000011701)
Fugu (XP_011601624.1)
Zebrafish (ENSDARP00000143362)
Amazon_molly (XP_016534180.1)
Medaka (XP_023818916.1)
Rainbow_trout (XP_021469805.1)
Rainbow trout (XP_021436521.1)
Salmon (XP_014010485)

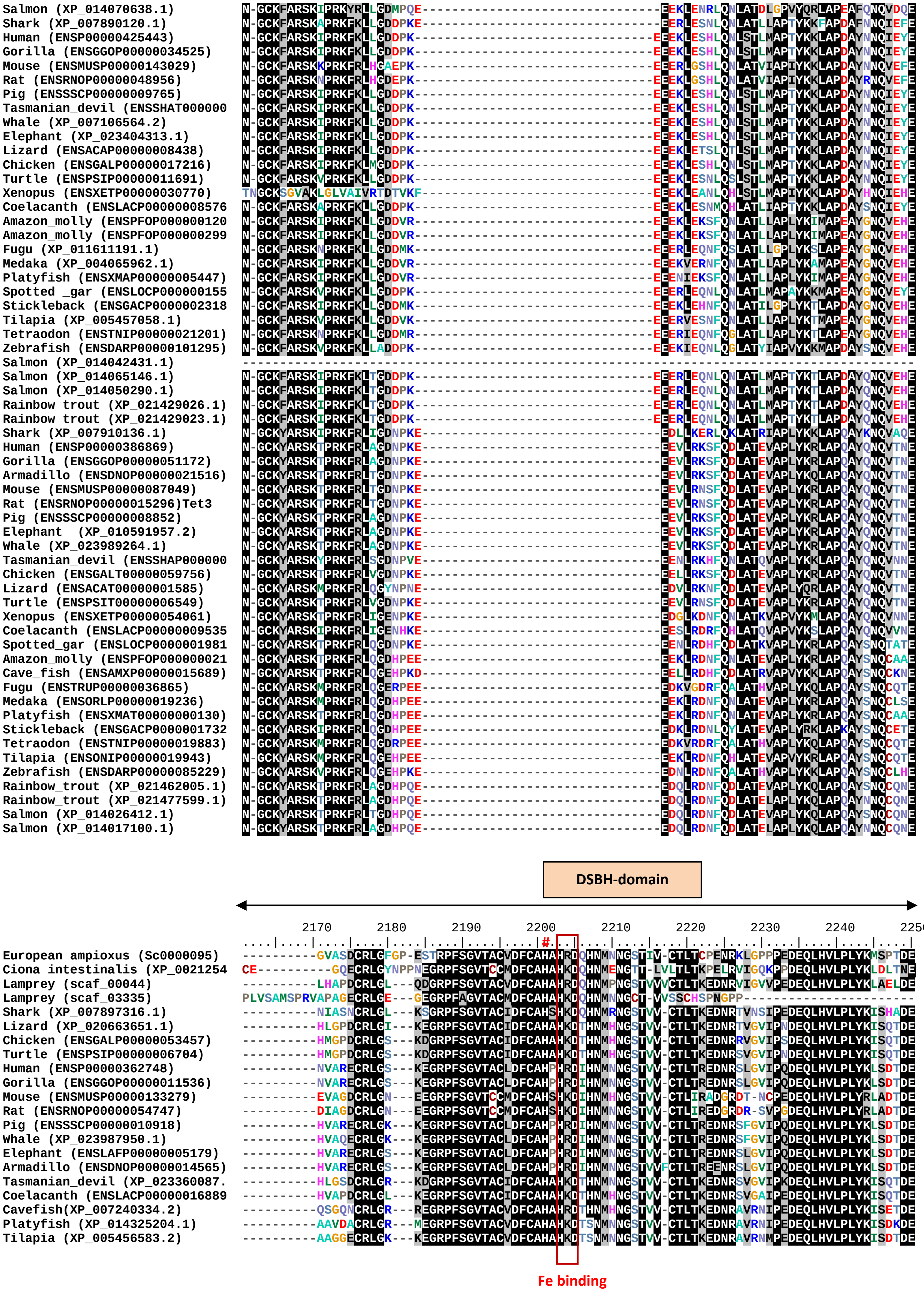
DSBH-domain
2170
2180
2190
2200
2210
2220
2230
2240
2250
Fe binding

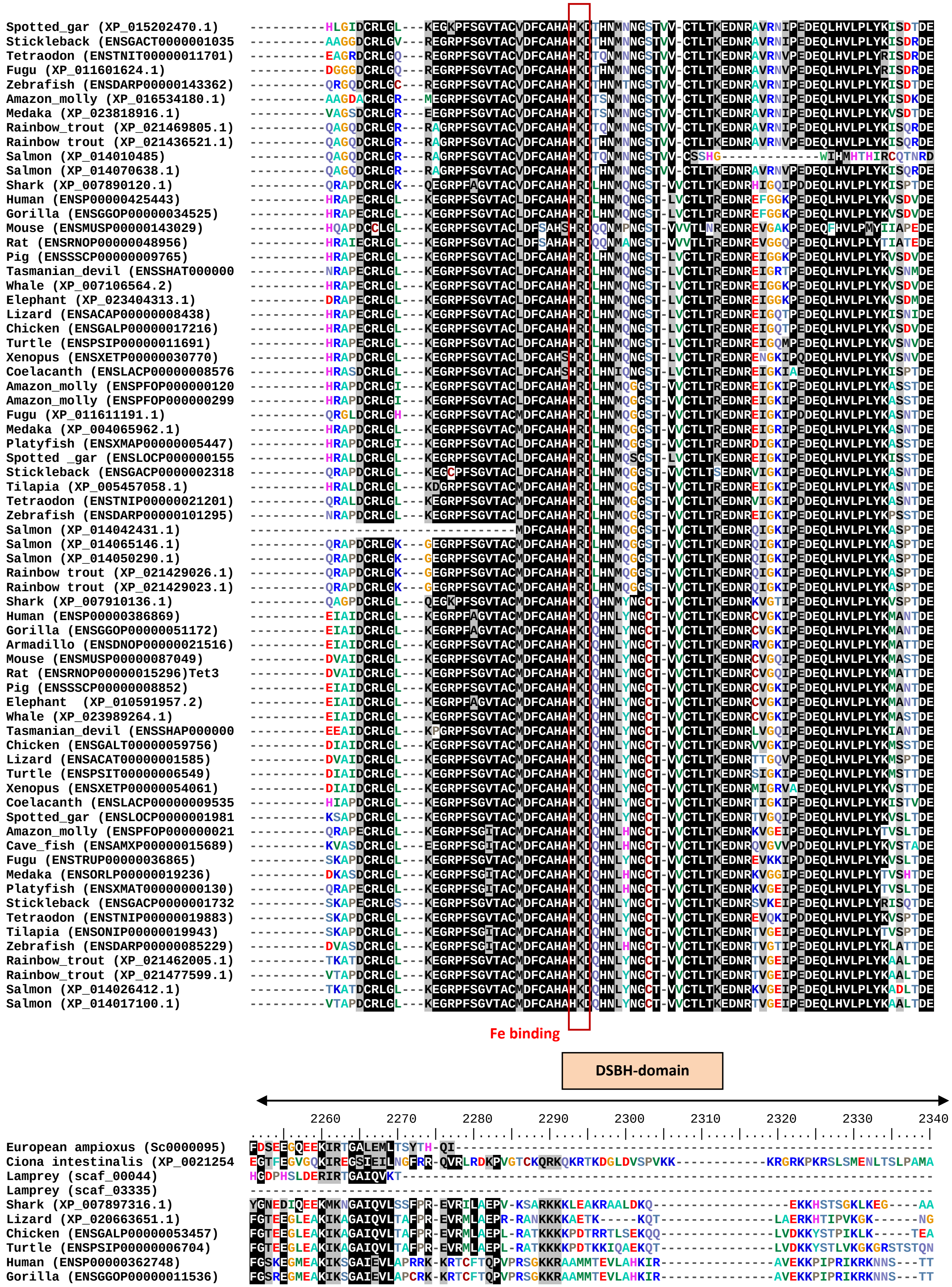
Fe binding
DSBH-domain
2260
2270
2280
2290
2300
2310
2320
2330
2340

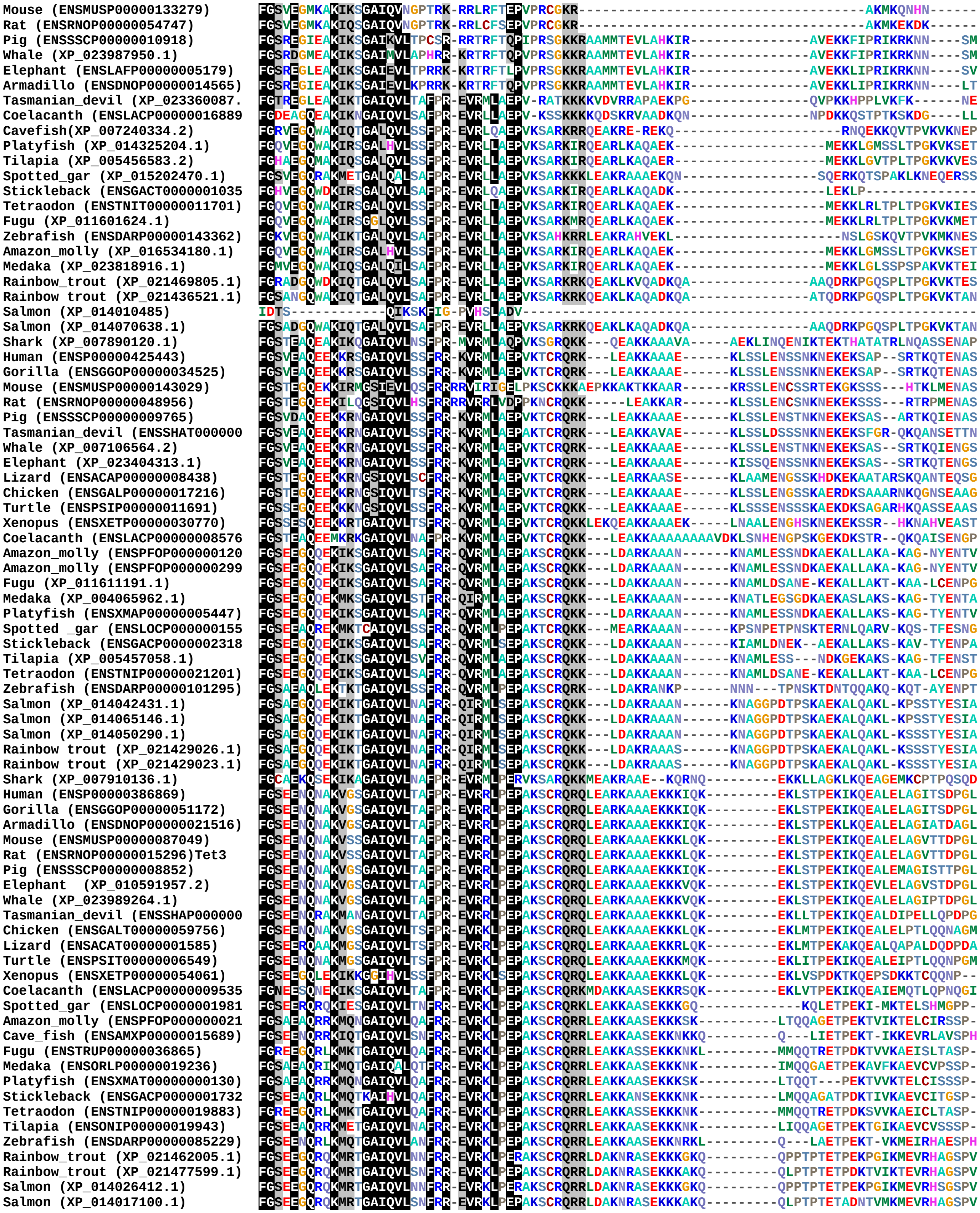
Mouse (ENSMUSP00000133279)
Rat (ENSRNOP00000054747)
Pig (ENSSSCP00000010918)
Whale (XP_023987950.1)
Elephant (ENSLAFP00000005179)
Armadillo (ENSDNOP00000014565)
Tasmanian_devil (XP_023360087.
Coelacanth (ENSLACP00000016889
Cavefish(XP_007240334.2)
Platyfish (XP_014325204.1)
Tilapia (XP_005456583.2)
Spotted_gar (XP_015202470.1)
Stickleback (ENSGACT0000001035
Tetraodon (ENSTNIT00000011701)
Fugu (XP_011601624.1)
Zebrafish (ENSDARP00000143362)
Amazon_molly (XP_016534180.1)
Medaka (XP_023818916.1)
Rainbow_trout (XP_021469805.1)
Rainbow trout (XP_021436521.1)
Salmon (XP_014010485)
Salmon (XP_014070638.1)
Shark (XP_007890120.1)
Human (ENSP00000425443)
Gorilla (ENSGGOP00000034525)
Mouse (ENSMUSP00000143029)
Rat (ENSRNOP00000048956)
Pig (ENSSSCP00000009765)
Tasmanian_devil (ENSSHAT000000
Whale (XP_007106564.2)
Elephant (XP_023404313.1)
Lizard (ENSACAP00000008438)
Chicken (ENSGALP00000017216)
Turtle (ENSPSIP00000011691)
Xenopus (ENSXETP00000030770)
Coelacanth (ENSLACP00000008576
Amazon_molly (ENSPFOP000000120
Amazon_molly (ENSPFOP000000299
Fugu (XP_011611191.1)
Medaka (XP_004065962.1)
Platyfish (ENSXMAP00000005447)
Spotted _gar (ENSLOCP000000155
Stickleback (ENSGACP0000002318
Tilapia (XP_005457058.1)
Tetraodon (ENSTNIP00000021201)
Zebrafish (ENSDARP00000101295)
Salmon (XP_014042431.1)
Salmon (XP_014065146.1)
Salmon (XP_014050290.1)
Rainbow trout (XP_021429026.1)
Rainbow trout (XP_021429023.1)
Shark (XP_007910136.1)
Human (ENSP00000386869)
Gorilla (ENSGGOP00000051172)
Armadillo (ENSDNOP00000021516)
Mouse (ENSMUSP00000087049)
Rat (ENSRNOP00000015296)Tet3
Pig (ENSSSCP00000008852)
Elephant (XP_010591957.2)
Whale (XP_023989264.1)
Tasmanian_devil (ENSSHAP000000
Chicken (ENSGALT00000059756)
Lizard (ENSACAT00000001585)
Turtle (ENSPSIT00000006549)
Xenopus (ENSXETP00000054061)
Coelacanth (ENSLACP00000009535
Spotted_gar (ENSLOCP0000001981
Amazon_molly (ENSPFOP000000021
Cave_fish (ENSAMXP00000015689)
Fugu (ENSTRUP00000036865)
Medaka (ENSORLP00000019236)
Platyfish (ENSXMAT00000000130)
Stickleback (ENSGACP0000001732
Tetraodon (ENSTNIP00000019883)
Tilapia (ENSONIP00000019943)
Zebrafish (ENSDARP00000085229)
Rainbow_trout (XP_021462005.1)
Rainbow_trout (XP_021477599.1)
Salmon (XP_014026412.1)
Salmon (XP_014017100.1)

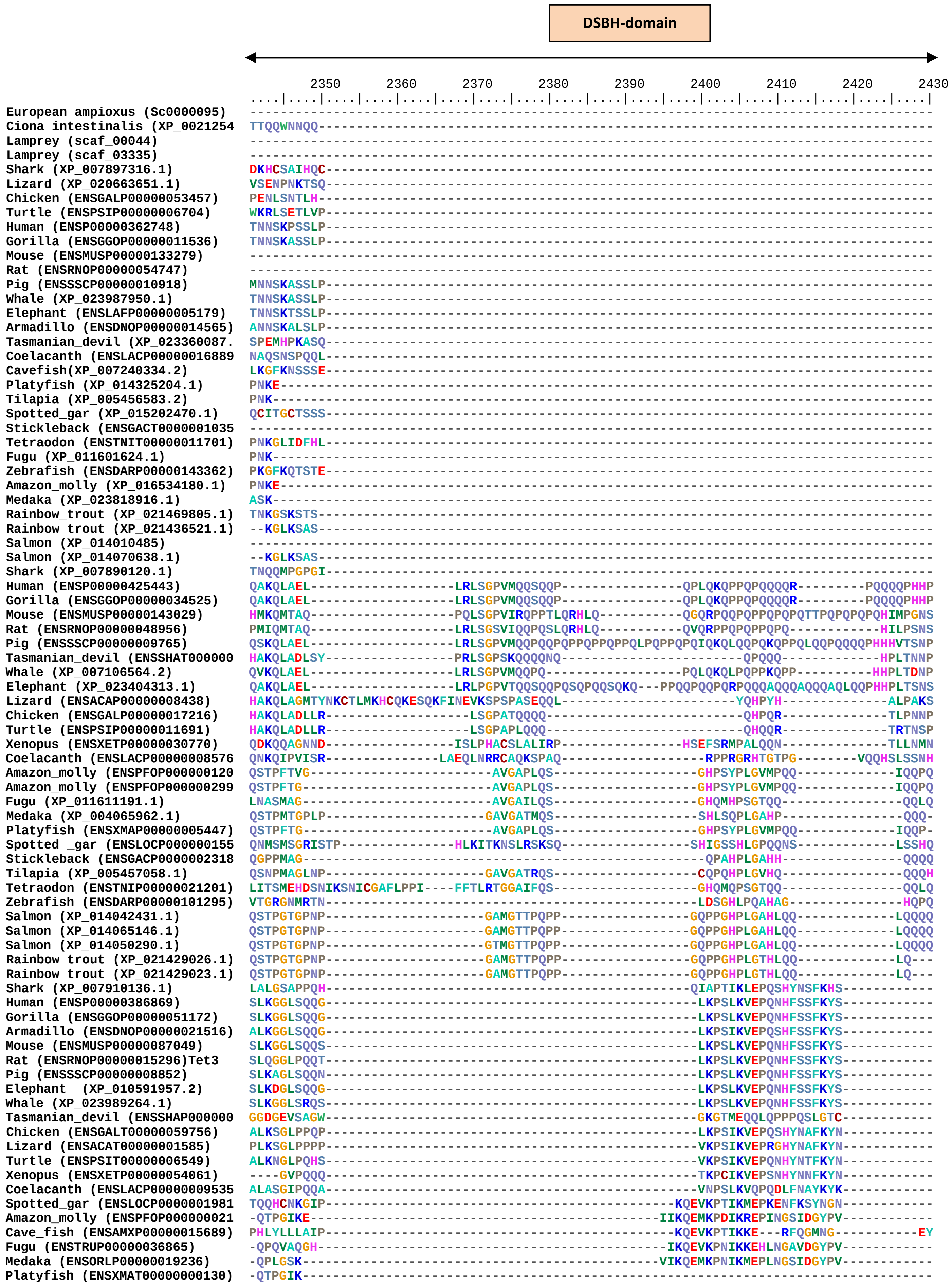
DSBH-domain
2350
2360
2370
2380
2390
2400
2410
2420
2430
European ampioxus (Sc0000095)
Ciona intestinalis (XP_0021254
Lamprey (scaf_00044)
Lamprey (scaf_03335)
Shark (XP_007897316.1)
Lizard (XP_020663651.1)
Chicken (ENSGALP00000053457)
Turtle (ENSPSIP00000006704)
Human (ENSP00000362748)
Gorilla (ENSGGOP00000011536)
Mouse (ENSMUSP00000133279)
Rat (ENSRNOP00000054747)
Pig (ENSSSCP00000010918)
Whale (XP_023987950.1)
Elephant (ENSLAFP00000005179)
Armadillo (ENSDNOP00000014565)
Tasmanian_devil (XP_023360087.
Coelacanth (ENSLACP00000016889
Cavefish(XP_007240334.2)
Platyfish (XP_014325204.1)
Tilapia (XP_005456583.2)
Spotted_gar (XP_015202470.1)
Stickleback (ENSGACT0000001035
Tetraodon (ENSTNIT00000011701)
Fugu (XP_011601624.1)
Zebrafish (ENSDARP00000143362)
Amazon_molly (XP_016534180.1)
Medaka (XP_023818916.1)
Rainbow_trout (XP_021469805.1)
Rainbow trout (XP_021436521.1)
Salmon (XP_014010485)
Salmon (XP_014070638.1)
Shark (XP_007890120.1)
Human (ENSP00000425443)
Gorilla (ENSGGOP00000034525)
Mouse (ENSMUSP00000143029)
Rat (ENSRNOP00000048956)
Pig (ENSSSCP00000009765)
Tasmanian_devil (ENSSHAT000000
Whale (XP_007106564.2)
Elephant (XP_023404313.1)
Lizard (ENSACAP00000008438)
Chicken (ENSGALP00000017216)
Turtle (ENSPSIP00000011691)
Xenopus (ENSXETP00000030770)
Coelacanth (ENSLACP00000008576
Amazon_molly (ENSPFOP000000120
Amazon_molly (ENSPFOP000000299
Fugu (XP_011611191.1)
Medaka (XP_004065962.1)
Platyfish (ENSXMAP00000005447)
Spotted _gar (ENSLOCP000000155
Stickleback (ENSGACP0000002318
Tilapia (XP_005457058.1)
Tetraodon (ENSTNIP00000021201)
Zebrafish (ENSDARP00000101295)
Salmon (XP_014042431.1)
Salmon (XP_014065146.1)
Salmon (XP_014050290.1)
Rainbow trout (XP_021429026.1)
Rainbow trout (XP_021429023.1)
Shark (XP_007910136.1)
Human (ENSP00000386869)
Gorilla (ENSGGOP00000051172)
Armadillo (ENSDNOP00000021516)
Mouse (ENSMUSP00000087049)
Rat (ENSRNOP00000015296)Tet3
Pig (ENSSSCP00000008852)
Elephant (XP_010591957.2)
Whale (XP_023989264.1)
Tasmanian_devil (ENSSHAP000000
Chicken (ENSGALT00000059756)
Lizard (ENSACAT00000001585)
Turtle (ENSPSIT00000006549)
Xenopus (ENSXETP00000054061)
Coelacanth (ENSLACP00000009535
Spotted_gar (ENSLOCP0000001981
Amazon_molly (ENSPFOP000000021
Cave_fish (ENSAMXP00000015689)
Fugu (ENSTRUP00000036865)
Medaka (ENSORLP00000019236)
Platyfish (ENSXMAT00000000130)

```
Stickleback (ENSGACP0000001732  -QLQDNK----------------------------------------------AIIKQEVKPNIKKEPFNGSMDGYRV------------
Tetraodon (ENSTNIP00000019883)  -QAQVNQG-------------------------------------------------RSMRDTLRRQPTCSTTSAH--------------
Tilapia (ENSONIP00000019943)    -QPQGNKVKQT------------------------------------------AIIKQEVKPNIKKDPLNGSIDGYSV------------
Zebrafish (ENSDARP00000085229)  IQQ-GNKAIP----------------------------------------------KQEVKPTIKKEPVDHFQPFNGAI--------HGY
Rainbow_trout (XP_021462005.1)  RQN-GNKAIP----------------------------------------------KQEVKPTIKKEPVDQRFQPFHG------------
Rainbow_trout (XP_021477599.1)  RQH-GNKVIP----------------------------------------------KQEVKPTIKKEAVDQ-------------------
Salmon (XP_014026412.1)         RQN-GNKAIP----------------------------------------------KQEVKPTIKKEPVDQRFQPFHG------------
Salmon (XP_014017100.1)         RQH-GNKAIP----------------------------------------------KQEVKPTIKKEPMDQ-------------------
```

DSBH-domain

```
                                        2440      2450      2460      2470      2480      2490      2500      2510      2520
                                ....|....|....|....|....|....|....|....|....|....|....|....|....|....|....|....|....|....|
European ampioxus (Sc0000095)   ------------------------------------------------------------------------------------------
Ciona intestinalis (XP_0021254  --WSISPSGLKIPPSPQKFDPSTFKDPILSPPPRWPYRRHVTQSDDSNLPSIGSFFSPNAHGAKTPQNQSVSPAIFGTPP----------
Lamprey (scaf_00044)            ------------------------------------------------------------------------------------------
Lamprey (scaf_03335)            ------------------------------------------------------------------------------------------
Shark (XP_007897316.1)          ---SAGCKMETASSSMKTRPQDHYNSFKYTGHTGVGNYSPLGGFTSPAPYNLGGVYPYSTLIPRPNLPTLPS------------------
Lizard (XP_020663651.1)         ---NLGSKSNTLQLGTKMETGDRLP-TIKQNSDTTTNCSVLKQYAPSSPLKLDSLLSCSPLTQKASGAMPVTN-----------------
Chicken (ENSGALP00000053457)    ---CLGNKADALKPGIKTETSDHLC-AMKHTSNTTKNCSLLKQYTASPPFKVDSLHPYSSLAHKPG-ITAVTN-----------------
Turtle (ENSPSIP00000006704)     ---TMTNKTDALQPGIKMETSDHPY-NVKHTSNATTSCSVLKQYTASSPVKVDSLHPYSSLTHKAS-ITAVTD-----------------
Human (ENSP00000362748)         ---TLGSNTETVQPEVKSETEPHF---ILKSSDNTKTYSLMPSAP--HPVKEA--SPGFSWSPKTASATPAP------------------
Gorilla (ENSGGOP00000011536)    ---TLGSKTETVQPEVKSETEPHF---ILKSSDNTKTYSLMPSAP--HPVKEA--SPGFSWSPKTASATPAP------------------
Mouse (ENSMUSP00000133279)      ----------------KS------------GSHNTKSFSSASSTS--HLVKDE--STDFCP-LQASSAETST------------------
Rat (ENSRNOP00000054747)        ----------------KS------------GSHNTKIYSSASSTP--HPVKDE--STDFCP-LQASSTETST------------------
Pig (ENSSSCP00000010918)        ---LLGNKIETMQPEIKSETEPHL---IFKGSENTKTYSPTPSIP--HPVKEANLAPGFSWSPKSASVTTPP------------------
Whale (XP_023987950.1)          ---LLGNKTETVQPEIKSETEPHL---IFKGSDNTKTYSPTPSIP--HPVKEAHLAPGFSWSPKTASVPTVP------------------
Elephant (ENSLAFP00000005179)   ---LLGCKTETLQPEIKSETEPHF---IFEGSDNTKTYSLIPSIP--HPVKEANLPPSFSWSPKSTSATTAP------------------
Armadillo (ENSDNOP00000014565)  ---LLGSKTETLQPEIKNEPEPHF---IFKGSDNTKTYSLIPSIP--HPLKEANLPPGFSWSPKSASATTAP------------------
Tasmanian_devil (XP_023360087.  ---LLGNKTETLQPDVKNELSDSHF-NLHSVAN-TTNYSWIKSIH--HPLKGTNLPLNSSLTPLSG--LAATS-----------------
Coelacanth (ENSLACP00000016889  ---GNRTSSLQKLTAMKMESQEHQNAFKYAGPTSVGSYSLLGNYTPSSSYNLCSVYPFPS-IPQPGTPTVSN------------------
Cavefish(XP_007240334.2)        ---LSPCFKTEACSPTPYCSPMRLPRTPDKGSHLLDSASPY-RSTSC-TPPLSSTRDTAALSSSPHTVLPVPQYQNVGELE---------
Platyfish (XP_014325204.1)      ----------PQGFYSSYGVPPRPTSVGRSTPERNQATAYSHSISSCSSPGAGITTQREVLPSNQHSVSNLQF-----------------
Tilapia (XP_005456583.2)        -----------AGYNSSY----RPASVERYLPEKNQACTYN-QISSYPIVGPGVNPQREVISPNTHSLPGLQF-----------------
Spotted_gar (XP_015202470.1)    ---QSPSLKLEPQDYYNSFKLPRQTGIGNYLLENYNPSAPYDISKMYPLPSVMPNAGMEALSKFHPSFSVPYGCLGLPANQ---------
Stickleback (ENSGACT0000001035  ---------------------PRPASTGRYPAEGNPTSTYNQSTSSYPTLGAEVTPQKKLTTPNHHGLPGLQS-----------------
Tetraodon (ENSTNIT00000011701)  ---NVSSKPEPQGYYSTQKLQPRPASVGRCLPDRVEPSTYSQSTSNYPSVSSGATTQKEVISLS-HGPAGLQF-----------------
Fugu (XP_011601624.1)           ---------EPQGYYSTQRLQPRPASVGRYLPDRNEPSTYNQSTSNYPSVNTGVTTQEDVFSLS-HGPAGLQF-----------------
Zebrafish (ENSDARP00000143362)  ---NS--SFSEKNSPN-LSSTMRVPDIGGQYSPLHSGGSYN-QSNSCRTPPVGSSFGTSTLPKFPHDPS--------GGTT---------
Amazon_molly (XP_016534180.1)   ----------PQGFYSAYGVPPRPTSVGRATPERNQAAAYSHSVSSCSSPGAGIASQREVLPSSHHSVSNLQF-----------------
Medaka (XP_023818916.1)         ------------DYYSAYRPPPRPSTAEKSTPERSQTSAYSQGTSSCST--SGVPPQRGVISSNHHGLPDLQF-----------------
Rainbow_trout (XP_021469805.1)  ---SVDQSSSFKAEPQSFYSSFTPGSVGAYAPESNSPSPYHHSTPTYPTPPGRSTPGMEALSPHRPGMPSTQYGYRGPPGA---------
Rainbow trout (XP_021436521.1)  ---SADQSPLFKREPQSFSS-YRPGNVGAYVPDSNSPSTYNHSTPTYPTPPGRSTPGMESLSPHRLGLPAPQY--RGPPGT---------
Salmon (XP_014010485)           ------------------------------------------------------------------------------------------
Salmon (XP_014070638.1)         ---SADQSPLFKREPQSFSS-YRPGSVGLYVPDSNSPSAYNHSTPTYPTPPGRSTPGMEALSPHRPGLPAPQY--RGPPGA---------
Shark (XP_007890120.1)          ------------------------------------------PPAIQQHAMPRHPVAGFYQSAPPS-----------AYP----------
Human (ENSP00000425443)         QTESVNSYSASGSTN-PYMRRPNPVSPYPNSSHTSDIY--GSTSPMNFYSTSSQAAGSYLNSSNPMNPYPGLLNQNTQYP----------
Gorilla (ENSGGOP00000034525)     QTESVNSYSASGSTN-PYMRRPNPVSPYPSSSHTSDIY--GSTSPMNFYSTSSQAAGSYLNSSNPMNPYPGLLNQNTQYP----------
Mouse (ENSMUSP00000143029)      QS--VGSHC-SGSTS-VYTRQPTPHSPYPSSAHTSDIY--GDTNHVNFYPTSSHASGSYLNPSNYMNPYLGLLNQNNQYA----------
Rat (ENSRNOP00000048956)        QP--VGSHS-SGSTN-VYMRQPAPLSPYPSSSHAADMY--GDANHVNFHPTSSHAAGLYLSPSNSMNPYPGLFNQNNQYA----------
Pig (ENSSSCP00000009765)        QSESVSSYSSSGSTN-LYMKRPNPVSPYPSSSHTSDIY--GGANPVNLYATSSQAAGSYLNSSNPMSPYSGLLNQNNQYP----------
Tasmanian_devil (ENSSHAT000000  QSSSANAYATSGPSN-LYMRRPNSINPYSSPSHPSDLY--SGTNPMNLYTTSSQAAGSYLNSSNPMNPYSGSLNQNNQYP----------
Whale (XP_007106564.2)          QSESVSSYASSGSTS-LYMRRPNPVSPYPSSSHTSDIY--GGANPVNLYSTSSQAAGSYLNSSNPMSPYPGLLNQSNQYPS---------
Elephant (XP_023404313.1)       QSEAVNSYSSSGPTN-LYMRRPNPVSPYASSSHTSDIY--GGANPMNLYSTSSQAAGSYFNSSNSMNSYPGLLNQNNQYP----------
Lizard (ENSACAP00000008438)     QTNHINAYSASGSTN-LYMGLPNPANPYSNSSHAPESY--RGSNPMNIYPTSSQPTGAYLNSSSLVNPYSVHLGQNSQYP----------
Chicken (ENSGALP00000017216)    QSNAINTYSGSGSAN-LYVRLPNPASAYPSSSYTSDPY--GGSGAMNLYTTSSQPAGSYLNSSSPMNPYSGSLSQNNQYP----------
Turtle (ENSPSIP00000011691)     QSNPINSYTGSSPAN-LYMRLPNTANAYPSSSYTSDLY--GGSNTMNLYTTSSQPAGSYLNSSNPMNPYSGSLSQNNQYP----------
Xenopus (ENSXETP00000030770)    HRNSLNHYPSSAPTN-PYTRIPNLPDPYQNSFSP---Y--PGSSHMNIFQTAASSSVSCANS--PMNSFQGSFNQGNSCP----------
Coelacanth (ENSLACP00000008576  QLNPMNCYPSSPHPASPYLRFSNPTSLYQSPSYSTNSCP-GSSNPLTTYPNSVHLGSPYLNSSNPVNPYTGPLTQTNPYS----------
Amazon_molly (ENSPFOP000000120  HQGMYPPYPSSPN--AALPRFSNSPGSFPGSPKPGSMYTAQPSTPASHFLSSL--PNSYMNGPNRP------------QP----------
Amazon_molly (ENSPFOP000000299  HQGMYPPYPSSPN--AALPRFSNSPGSFPGSPKPGSMYTAQPSTPASHFLSSL--PNSYMNGPNRP------------QP----------
Fugu (XP_011611191.1)           HENIRSPFPGSMNA-AGYPRFPDHQMLFPSTSKPGSMYSPQSQTPSSPFTSPLRVPNSYMNGSNRP------------YA----------
Medaka (XP_004065962.1)         HQSMIPPYSRSTH--PGFMRFPNSPGSFPSTSKPGSMCPTQAQAPASPYPSPLNVTNSYMNGPNRP------------YP----------
Platyfish (ENSXMAP00000005447)  -QAMCPPYPSSPN--TALPRFSNSPGSFPGSPKPGSMYLAQPSTPANHFLSSL--PNSYMNGPNRP------------QP----------
Spotted _gar (ENSLOCP000000155  QKNNLNHHPNSLHP-HSFQRFPNPTDQFPSTSQPANLYQ-ESTTPTNPYPTSLRAADSYLNASTPMNSYPASLNPSNLHT----------
Stickleback (ENSGACP0000002318  HQSALPPYPGSTNV-ASYPRFPNHPGSFPSTSKPGTMYPPQPPTPASPYTSPLHAPNSYINGTNRP------------YP----------
Tilapia (XP_005457058.1)        HQSILPPYPGSTN--AAFSRFPNHNGSFPSTSKPGSIYPHQP-SSSSPYPSPLHATNSYMNASNRP------------YL----------
Tetraodon (ENSTNIP00000021201)  HENIRSPFPGSANA-ASYPQVPEH------------------------------------TGSNRP------------YP----------
Zebrafish (ENSDARP00000101295)  QQFPQQTHPNPSYASPPFTRFPNAS-------KPSTPHP--QTPSVDPYASPLHAPNSYINASNAPSPYSRSLAPSPLYN----------
Salmon (XP_014042431.1)         HQNALHAFPGSPHPASPYARFPNHPGPFQSTSKPGSMYP-QPPASASPYPSPLHVPTSYINGSNPPSSYPSPMTPGSPYP----------
Salmon (XP_014065146.1)         HQNALHAFPGSPHPASPYARFPNHPGPFQSTSKPGSMYP-QPPASASPYPSPLHVPTSYINGSNPPSSYPSPMTPGSPYP----------
Salmon (XP_014050290.1)         HQNALHAFPGSPHPASPYVRFPNHPGPFQSTSKPGSMYP-QPPASASSYPSPLHLPTSYMNGSNPPSSYPSPMTPGSPYP----------
Rainbow trout (XP_021429026.1)  HQNALHAFPGSPHPASPYAGFPNHP--FQSTSKPGSMYP-QPPASASPYPSPLHIPTSYMNGSNPPSSYPSPMTPGSPYP----------
Rainbow trout (XP_021429023.1)  HQNALHAFPGSPHPASPYAGFPNHP--FQSTSKPGSMYP-QPPASASPYPSPLHIPTSYMNGSNPPSSYPSPMTPGSPYP----------
Shark (XP_007910136.1)          -GNSVVESYTVLGNCRPSDPYSLGSVYPYHSYYARPG-----LPALSAYRHPGYS-FSYGYYGYASN-PLFPPPFLKYGS----------
Human (ENSP00000386869)         -GNAVVESYSVLGNCRPSDPYSMNSVYSYHSYYAQPS-----LTSVNGFHSKYAL-PSFSYYGFPSSNPVFPSQFLGPGA---WGHSGSS
Gorilla (ENSGGOP00000051172)    -GNAVVESYSVLGNCRPSDPYSMNSVYSYHSYYAQPS-----LTSVNGFHSKYAL-PSFSYYGFPSSNPVFPSQFLGPGA---WGHSGSS
Armadillo (ENSDNOP00000021516)  -GNAVVESYSVLGSCRPSDPYSLSSVYSYHSYYAQPS-----LPAVNGFHSKYTL-PSFGYYGFPSSSPVFSTQFLGPGT---WGHGGSG
Mouse (ENSMUSP00000087049)      -GNAVVESYSVLGSCRPSDPYSMSSVYSYHSRYAQPG-----LASVNGFHSKYTL-PSFGYYGFPSSNPVFPSQFLGPSA---WGHGGSG
Rat (ENSRNOP00000015296)Tet3    -GNAVVESYSVLGSCRPSDPYSMSSVYSYHSRYAQPG-----LASVNGFHSKYTL-PSFGYYGFPSSNPVFPSQFLGPSA---WGHGGGG
Pig (ENSSSCP00000008852)        -GNAVVESYSVLGNCRPSDPYSVNSVYSYHSYYAQPG-----LASVNGFHSKYAL-PSFSYYSFPSSNPVFPSQFLGPSA---WGHSGSS
Elephant  (XP_010591957.2)      -GNTVVESYSVLGNCRPSDPYSMNSVYSYHSYYAQPS-----LTSVNGFHSKYAL-PSFGYYGFPSSNPVFPSQFLGPGA---WGHGGSS
Whale (XP_023989264.1)          -GNTVVESYSVLGSCRPSDPYSVNSVYSYHSYYAQPG-----LASVNGFHSKYAL-PSFGYYGFPSSNPVFPSQFLGPST---WGHSGSN
Tasmanian_devil (ENSSHAP000000  -LWSLLKQN-FPTDAFLKERTSATRFVHPVPVFVELW-----LVSLPADEWPERG-AGLGLGGRGGRVMQWAPGTGGGEE---WGKG---
```

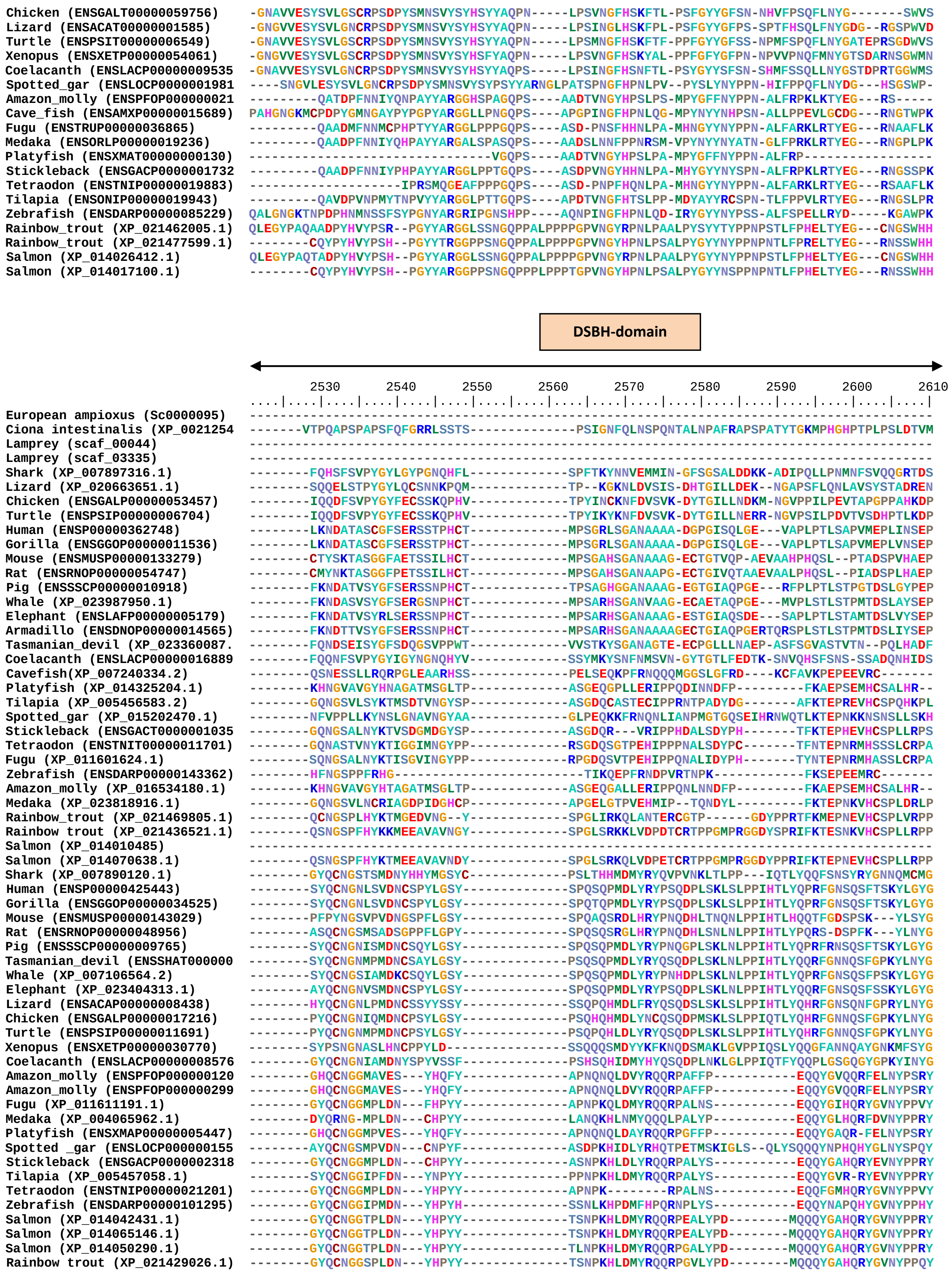
DSBH-domain
2530
2540
2550
2560
2570
2580
2590
2600
2610

```
Rainbow trout (XP_021429023.1)      --------GYQCNGGSPLDN---YHPYY--------------TSNPKHLDMYRQQRPGVLYPD--------MQQQYGAHQRYGVNYPPQY
Shark (XP_007910136.1)              --------SFDQKPDVQELCSSYSLALRP---------GRTDCAEPGLGPTPPPPARPGPGLSLTTS----PPAPRAPGGPARAS-----
Human (ENSP00000386869)             G-------SFEKKPDLHALHNSLSPAYGG-AEFAELPSQAVPTDAHHPTPHHQQPAYPGPKEYLLPKAPLLHSVSRDPSPFAQSS-NCYN
Gorilla (ENSGGOP00000051172)        G-------SFEKKPDLHALHNSLSPAYGG-AEFAELPSQAVPTDAHHPTPHHQQPAYPGPKEYLLPKAPPIHSVSRDPSPFAQSS-NCYN
Armadillo (ENSDNOP00000021516)      GG-----SSFEKKPDLHALHSSLSPAY---------------------------------------------------------------
Mouse (ENSMUSP00000087049)          G-------SFEKKPDLHALHNSLNPAYGG-AEFAELPGQAVATDNHHPIPHHQQPAYPGPKEYLLPKVPQLHPASRDPSPFAQSS-SCYN
Rat (ENSRNOP00000015296)Tet3        G-------SFEKKPDLHALHNSLNPAYGG-AEFAELPGQAVSTDNHHPAPHHQQPAYPGPKEYLLPKVPQLHPASRDPSPFAQSS-GCYN
Pig (ENSSSCP00000008852)            S-------NFEKKPDLHALHNSLSPAYGG-AEFAELPGQAVPTDTHHPSPHHQQPAYPGPKEYLLPKAPQIHPVSRDPSPFAQSS-NCYN
Elephant  (XP_010591957.2)          G-------SFEKKPDLHALHNSLSPAYGG-AEFAELPGQAVATDTLHPTPHHQQPAYPGPKEYLLPKVPQIHSASRDPSPFAQSS-NCYT
Whale (XP_023989264.1)              G-------SFEKKPDLHALHNSLSPAYGG-AEFAELPGQAVPTDTHHPAPHHQQPAYPGPKEYLLPKAPQIHPVSRDPSPFVQSS-SCYN
Tasmanian_devil (ENSSHAP000000      ---------LDYEPGKGRGDGPGSGRRGR-VGEGASGSEASKSDGHHTP--HHQPAYPGPKEYLLPKGHRTHPAATAPSPFAQSG-PCFG
Chicken (ENSGALT00000059756)        N-------SYEKKPNIQVLQENLNHTYRN-TDFPEPIPHSVRSKNHHQRTYERASRYASQQKAAAAGVHRTSTGSEEASPFAQN--CFGS
Lizard (ENSACAT00000001585)         GNG----GGYEKKPDVQVLQDNLSRVYRS-PDPLDDVQPTVRSKSHHQWTYERANRGVSKTPPATS-PRCPSAAPSDALPFAQNTHCFNS
Turtle (ENSPSIT00000006549)         S-------NYEKKPDLHALQDNLNHTYRN-SEFSEPLPHTVRNKNHHQRTYERANRYASQKSTAMS--HRTNSVPVESPPFAQNTNCFNN
Xenopus (ENSXETP00000054061)        N-------CFEKKPELQSLADGMNQSYGS--ELSEQSFRRSSEVPHHYSLQNPS----SQKSVNVP--HRTTPAPVETTPYSNLP--CYN
Coelacanth (ENSLACP00000009535      S------HNFEKKPNLSALDS-LNSSYRSKSEFSEQASHAAPKVTHQLPTLQGSREFANNKPSHLP--HRPSQITMETKPCSQT-VNFLG
Spotted_gar (ENSLOCP0000001981      -----KATIYEKKPDVQTLQASLGKSSFP-----DYPEQSQ-GVTPSKTGYPLHSLVPASPEFAQPLVPP-HRNHQAPEGARRSFPSPLT
Amazon_molly (ENSPFOP000000021      -----KLVQAERKSDAQSPQAHLPS-----------------NSHPEQNNQPNNSAYAQPANYSLS-----RPSSVSSEASNRGTP----
Cave_fish (ENSAMXP00000015689)      S------AALDQKRDP---QARLAQAYSN-----RPDQQRQQVADLSNP------GYLHQPELSQSPATPNRTPSATPDQ-HRGTP----
Fugu (ENSTRUP00000036865)           ADV--KTEEVDKKPDVQSLQARLAEAF---------------PGNAS-----SHGGYSQPASYSQS-----RPSSVSSEASNRGTP----
Medaka (ENSORLP00000019236)         TGG--KPVEVDKKPDIQSLQVRMAQS----------------SSHPEQQNQLNHTVYSQPADYNQS-----RPSSNSSEPSIRGSP----
Platyfish (ENSXMAT00000000130)      --------KAERKSDAQSPQGHLPS-----------------NSHPEQNNQPNNSAYAQPANYSLS-----RPSSVSSEPSNRGTP----
Stickleback (ENSGACP0000001732      AGG--KVAQADLKPDIQSLQARLTQSF---------------PSHPEQANQPNLSAYAQHADHNQS-----RSSSVCSEQSNRGTP----
Tetraodon (ENSTNIP00000019883)      AEV--KSEEVEKKPDVQGLQARLAEAF---------------PG-----------GYSQPANFSQS-----RPSLASSEASNRGTP----
Tilapia (ENSONIP00000019943)        AGC--KAVQVDQKPDIQNFQSRVALSC---------------SSQAEQANQPNHTTYTQAPVYSQS-----RPSSASSESSNRGTP----
Zebrafish (ENSDARP00000085229)      NGE-PSAQAFEQKPDVQCLQANLAQAYPT-----HHGQSQAQMADASIQ------GYPQPSDISQC-----RTPSMSLEQTHRSKP----
Rainbow_trout (XP_021462005.1)      MGPKFSPSPLDKKPDVQSLQARLAHSYSS-----PGHLEQQQHGDPANQHQRSAYPHPHQPDYNQS-----RPSSASSEPSHRGTP----
Rainbow_trout (XP_021477599.1)      AGHNASPGPVDKKPDVQSLQAKLAHSYSG-----PGLPEQQ-HGDPANQHQCNAYPHPHQPDYNQS-----RPSSVSSEPSHRGTP----
Salmon (XP_014026412.1)             MGHKFSPSPVDKKPDVQSLQARLAHSYSS-----PGHLEQQ-HGDPANQHQRSAYPHPHQTDYNQS-----RPSSVSSEPSHRGTP----
Salmon (XP_014017100.1)             AGHNASPGPVDKKPDIQSLQARLAQSYSG-----PGLPEQQ-HGDPANQHQCNAYPHPHQPDYNQS-----RPSSVSSEPSHRGTP----
```

DSBH-domain

```
                                            2620      2630      2640      2650      2660      2670      2680      2690      2700
                                    ....|....|....|....|....|....|....|....|....|....|....|....|....|....|....|....|....|....|
European ampioxus (Sc0000095)       ------------------------------------------------------------------------------------------
Ciona intestinalis (XP_0021254      HHPFHQTFATVKSEPSTD--------QVAYYPQQENKINLKS-------YAAVKTEAGSAFPTQSYPQHPQQPPNLQFQQPQPPYP----
Lamprey (scaf_00044)                ------------------------------------------------------------------------------------------
Lamprey (scaf_03335)                ------------------------------------------------------------------------------------------
Shark (XP_007897316.1)              LDQIYKNATQFTQQRKP----------QAIKEQTSHSQLPGSCD-----NMSLSLETKTTDTSKPNGLVKLQLGGNFREFQQSFTQDESL
Lizard (XP_020663651.1)             LPYTLEQEEVSKQNCEI----------KLENSLASQLASS------------CESSLRLNAPKEVARNEISSSQECPVQKDGFMQISNCD
Chicken (ENSGALP00000053457)        LPSILEHQP-DKQNCQP----------QSDSSPSSQMISS------------CDLSVPLSSP---AKDAQNSALGQPTDSE---------
Turtle (ENSPSIP00000006704)         LHSILEHQPNDMQNCQL----------QLENAPS-QSINP------------CDMPIPLNSP---TKDVTRN--DEPMDSE---------
Human (ENSP00000362748)             STGVTEPLTPHQPNHQP----------SFLTSPQ-DLASS-----------PMEEDEQHSEADEPPSDEPLSDDPLSPAEE---------
Gorilla (ENSGGOP00000011536)        STGVTEPLTPHQPNHQP----------SFLTSPQ-DLASS-----------PMEEDEQHSEADEPPSDEPLSDDLLSPAEE---------
Mouse (ENSMUSP00000133279)          LTSPSEQLTSNQSNQQL----------PLLSNSQ-KLASC-----------QVE-DERHPEADEPQ----------HPEDD---------
Rat (ENSRNOP00000054747)            LTSPPDHLTSNQSNQQL----------PFLSSPQ-KLASC-----------QVE-DERHSETDEPP----------QPEDG---------
Pig (ENSSSCP00000010918)            PTGPCEHLPSHQPNQQS----------PFVTFPH-DPASS-----------LVEEDEQHSEADEPLSDDPLSDDPLSPVEE---------
Whale (XP_023987950.1)              PTGPSEQLTSDQPNQQP----------PFIPSPH-ELASS-----------LAEEDEQHSEADEPPSDDPLSDDPLSPAEE---------
Elephant (ENSLAFP00000005179)       PIGATEQLTPNQPNQQP----------LFITSPH-ELAPS-----------VAEEDEQHSEADEPLSDEPLSDDPLSPAEE---------
Armadillo (ENSDNOP00000014565)      PTGPTEQLTSIQPNQQS----------PFITSPH-ELAPS-----------LVEEDEQHSEADELLSDDPLSDEPLSPAEE---------
Tasmanian_devil (XP_023360087.      SPGPVEHLISDPTKFQP----------PSLLPPVTQIAGPGDST-----PGKSDSDEHMTGPREAAALASSLVYPRRQEPE---PAAHFP
Coelacanth (ENSLACP00000016889      LNRMFEN--QVVKEQKI----------PNQLPNSQFSQLSNACN---------SLEKIFANSKATN-------GSDSNSY----------
Cavefish(XP_007240334.2)            -FQGGAAPRLLSPSRA-----------EGLHSRLNFRQA----------------------QPSLDDHHGPPVTPRPLT-----------
Platyfish (XP_014325204.1)          -----LSPSSFSARPTS----------EGLFSRLNGLLR----------AAESVAGEVRGHDFPPGQ--SLPLQAPPLE-----------
Tilapia (XP_005456583.2)            PSGSAPPPSPFSPSPTS----------EGLFSRLNGLHG----------SVGDVIAEARGHGLPPISPLSLPVQTPPFE-----------
Spotted_gar (XP_015202470.1)        PHG--STPELFCSKPP-----------EALSNRPNGFHR-------------VQFAEKDQHGLLAEHIQGTHNVCRTPESP---------
Stickleback (ENSGACT0000001035      PSGSAPPHSSFSPRPTS----------EGLFSRLNGMHR----------PAEEATPEVGGHGFPPLSSLHLPPHSPSLE-----------
Tetraodon (ENSTNIT00000011701)      PSRSVPTPSSFSPRPTS----------EGLFSRINGLHR----------AAEDVAPEVKGQGLPHLSSLPPHTQTPLLK-----------
Fugu (XP_011601624.1)               PSRSVPPPSSFSPRPTS----------EGLFSRLNGLHT----------AAEDIATEVKGHGLPHLSSLPLHAPTHMLE-----------
Zebrafish (ENSDARP00000143362)      -FQGGAAPTPLSPPMA-----------EGLHSRLN-------------------------------------ITAQPVT-----------
Amazon_molly (XP_016534180.1)       -----LSPSSFSARPTS----------EGLFGRLNGLLR----------AAEDVAGEVRGHDFPPGQ--SLPLQTPPLEE----------
Medaka (XP_023818916.1)             PSG--PPPSSFSPRPTS----------EGLFSRLNGLHG----------AVG----EVRGHGFPPGLP---PPQPTPPTE----------
Rainbow_trout (XP_021469805.1)      HSHSTPPPSSSSFSAPLPQP-------EGLHSRLNGLPGVAEEI-----G--DGVKGHGGHDLPHCATHLPLQAPPPLFPN---------
Rainbow trout (XP_021436521.1)      HSHSTPPPSSSTHTHP-----------EGLHSRLNGLLRAKAEL-----GVKDGVRGHVGHVIPHSAPHPPHQAPSPLFPN---------
Salmon (XP_014010485)               ------------------------------------------------------------------------------------------
Salmon (XP_014070638.1)             HSHSTPPPSS-THTHP-----------EGLHSRLNGLLRAEGEV-----GVKDVVRGHVGHVIPHSAPHPPHQAPPPLFPN---------
Shark (XP_007890120.1)              RFWNYGDYGMPVNGYSNCQI-------DNRIPTIPQIRAYPH-------YESSPQIEPQLMDTASKPVSSLGHPSLDHAAVAHSSQYP--
Human (ENSP00000425443)              NQN------MQGDGFSS----------CTIRPNVHHVGKLPP-------YPT-HEMDGHFMGATSRLPPNLSNPNMDY----KNGEH---
Gorilla (ENSGGOP00000034525)        NQN------MQGDGFSS----------CTIRPNVHHVGKLPP-------YPT-HEMDGHFMGATSRLPSNLSNPNMDY----KNGEH---
Mouse (ENSMUSP00000143029)          NQN------MQRDAFTTN---------STLKPNVHHLATFSP-------YPT-PKMDSHFMGAASRSP--YSHPHTDY----KTSEH---
Rat (ENSRNOP00000048956)            NQNNV---QRDRDAFTN----------CPLKPNVHHLPTFSS-------YAT-PKMDGHFMGAASRLP--YSHPNMEY----KSSDH---
Pig (ENSSSCP00000009765)            NQN------VQGDAFSS----------CTVRPNVHHVGAFPP-------YSA-HEMDGHFMGAASRLPPNLSNPNIDY----KNGDH---
Tasmanian_devil (ENSSHAT000000      NQN------MQVDPYSS----------YTIRPNVHHVGSYPP-------YSM-HDMDGYFMESASRLPPNLNNPTLDYTSMNKNGEY---
Whale (XP_007106564.2)              NQN------MQGDAFSS----------CTLRPNVHHVGTFPP-------YSA-HEMDGHFMGAASRLPSNLSNPNTDY----KNGEH---
Elephant (XP_023404313.1)           NQN------MQGDAFSS----------CTIRPNVHHVGTFSP-------YST-HEMDSHFMGAASRLPPHLSNPDIDY----KNGEH---
Lizard (ENSACAP00000008438)         NQN------VQVDAFNS----------CTIRPNVQHAGSFPP-------YST-HEAEDPFMDIASRLKSSLNNPGMDYINKN----SDH-
Chicken (ENSGALP00000017216)        NQN------MQVDSFSN----------CTIRPNVHHVGSFSS-------YST-HEADGHFMEVASRLKSNLSNPSMDYASMSKTTEHHH-
Turtle (ENSPSIP00000011691)         NQN------MQVDSFNN----------CTSRPNVHHTGSFSP-------YST-HEADSHFMDVASRLKSNFTNPSVNYTSINKNGEP-H-
Xenopus (ENSXETP00000030770)        NQR------VQADDFSG----------CNAYP----------------------APQADIMNPATSSRPITSNQ--DFSSATKNGEY---
Coelacanth (ENSLACP00000008576      NHN------VQVSGYSN----------CSMRPNTLQMGSYPA-------YGTNHQVEAHFMEAASRSASSLSHPSLDFASLSKSNQYHP-
Amazon_molly (ENSPFOP000000120      SQPG-----FQVNGYSA-------------MRPPQPMRHYGP-------FGNNRTSDARFIDTLTGAPS-VHG-GMDYALAVSKANQFG-
Amazon_molly (ENSPFOP000000299      SQPG-----FQVNGYSA-------------MRPPQPMRHYGP-------FGNNRTSDARFIDTLTGAPS-VHG-GMDYALAVSKANQFG-
Fugu (XP_011611191.1)               GEAG-----LQVNGYNPC---------G--IRPLTPVRPFSP-------YGANGASDPRLVDPLSRTPS-THE-GLDYTAAASKGNQFG-
Medaka (XP_004065962.1)             SESG-----LQVNGYNG-------------MRPAQIMQPYNP-------YGPSRPLDAQFIDPLSRMPS-GHG-GMDYSPVVSKSNQLG-
Platyfish (ENSXMAP00000005447)      SQPG-----FQVNGYSA-------------MRPPQPMRHYGP-------FGNNRTSDARFIDTLTGAPS-VHG-GMDYTLTVSKGNQFG-
```

```
Spotted _gar (ENSLOCP000000155   GDHN-----IQVNGYGNC---------N-MRPNVHSMGPYPS-------FGPNAGTEAQFFEAISRPPS-TRP-NLDY-AAVNKGNQYN-
Stickleback (ENSGACP0000002318   AEPG-----LQVNGYNAC---------S--MRPGHPARPYGP-------YGPNGASDPQFMDPLSRAPS-AHG-GLDYAAAVSKGNQFG-
Tilapia (XP_005457058.1)         GEPG-----LQVNGYNAS---------S--IRPVPPMRPYGP-------YTPNSVSDPRFMDPLSRASS-AHA-GVDYTTAMSKGNRFG-
Tetraodon (ENSTNIP00000021201)   GEAG-----LQVNGYNTC---------G--MRPVSPMRPFPP-------YGANGASDPRFIDPLSRAPS-THE-GLDYTAAANKSNQLG-
Zebrafish (ENSDARP00000101295)   GEA------MATNGYGNC---------N-MRPGIHSMGHYPG-------FDSNMSTN-----AFARPPS-AHL-HLDYAAAGN-------
Salmon (XP_014042431.1)          GESG-----LQINGYSNC---------SSMRSSIHPMSPYGPPS-----YGPSGAPDAQYLDALSRPPS-AHHPGLDYAAAVSKGNQFG-
Salmon (XP_014065146.1)          GESG-----LQINGYSNC---------SSMRSSIHPMSPYGPPS-----YGPSGAPDAQYLDALSRPPS-AHHPGLDYAAAVSKGNQFG-
Salmon (XP_014050290.1)          GEPG-----LQINGYSNC---------SSMRSGIHPMSPYGPPS-----YGPSGAPDAQYLDALSRPPS-AHHPGLDYAAAVSKGNQFG-
Rainbow trout (XP_021429026.1)   GEPG-----LQINGYSNC---------SSMRSGIHPMSPYGPPS-----YGPNGAPDAQYLDALSRPPS-AHHPGLDYAAAVSKGNQFG-
Rainbow trout (XP_021429023.1)   GEPG-----LQINGYSNC---------SSMRSGIHPMSPYGPPS-----YGPNGAPDAQYLDALSRPPS-AHHPGLDYAAAVSKGNQFG-
Shark (XP_007910136.1)           -ASAQPRRQALGRLQ-----------SQREPAVATAARGVRP-----------------REAVEPVRGVGRLRARAVGVTEGQPEAPAFR
Human (ENSP00000386869)          RSIKQEPVDPLTQAEPVPRD----AGKMGKTPLSEVSQNGGP-------SHLWG-QYSGGPSMSPKRTNGVGGSWGVFSSGESPA----I
Gorilla (ENSGGOP00000051172)     RSIKQEPVDPLTQAEPVPRD----AGKMGKTPLSEVSQNGGP-------SHLWG-QYSGGPSMSPKRTNGVGGSWGVFSSGESPA----I
Armadillo (ENSDNOP00000021516)   ------------EVEPVPRD----PGKVGKTPVPEVSQNGGP-------SHLWG-QYSGGSSVSPKRTNRVGGAWGVLPPAESPA----V
Mouse (ENSMUSP00000087049)        RSIKQEPIDPLTQAESIPRD----SAKMSRTPLPEASQNGGP-------SHLWG-QYSGGPSMSPKRTNSVGGNWGVFPPGESPT----I
Rat (ENSRNOP00000015296)Tet3     RSIKQEPVDPLTQAEAVPRD----SGKMSRTPLPEASQNGGP-------GHLWG-QYSGGPSMSPKRTNSVGGNWGVFPSGESPT----I
Pig (ENSSSCP00000008852)         RSIKQEPVDPLVHVESVSRE----PGKIGKTPLPEASQNGGP-------NHLWG-QYSGGPSMSPKRTTSVGGSWGVFPPAESPA----L
Elephant  (XP_010591957.2)       RSIKQEPVDPLAQAEPVPRD----PSKMGKTPLPEASQNGGP-------NHLWG-QYAGGPSISPKRTNSVGGSWSVFPSGESPA----I
Whale (XP_023989264.1)           RSIKQEPVDPLVHAESVPRE----PGKMGKMPLPEASQNGGP-------NHLWG-QYSGGPSVSPKRTNSVGGSWGVFPPAESPA----V
Tasmanian_devil (ENSSHAP000000   QVIKQEPCDRLPQAEPLPGV----PGNMGKPPIPEASQNGTK-------EHLWGGAHPGSQSASPKRTNGVTGAWGLFAPGESP-----V
Chicken (ENSGALT00000059756)     RTIKQEPPDPPPSIEPLNNP----AAAVPGTGLALPA-VPVP------EQQWSPYKASSRGSSSPEQTGAADSSWSSLVPG-------AG
Lizard (ENSACAT00000001585)      RTIKQEPADPEAVQRPAELS----CQNLN-PEMSQNG-GPME------PPAWSPYKP-HRNESPCERTSHAENPWSAFPSGDSM--LGAN
Turtle (ENSPSIT00000006549)      RAIKQEPVDALSHIESIQRA----AVNVQNSSLNLSA-VPVASQNGEQEQQWSPYKV-NRNGSPPDRTSNAERSWNTFTPNDNTNLPTTS
Xenopus (ENSXETP00000054061)     KVIKKEP-GSDPLVDSFQR-----ANSVHSHSPGVNHSLQAS-------DLPISYKA--NGALSSSGRTNAESPCSMFMPND------KN
Coelacanth (ENSLACP00000009535   RVIKQEPEGSFPYLDSVHGS----ALSTRNGNCSSAATLPASSQTCEQERLWHSHKM--NGTLPSSERTVPGASWNVFGSNESTKLSNMN
Spotted_gar (ENSLOCP0000001981   KMIKQEPIDSPQYTDCGGAAGGDANPPARDDITHGSIQGPSPGP----QHEQWPGYKMNGNLGSESWDSNRKVSTPHDGATTPLPSP---
Amazon_molly (ENSPFOP000000021   -LIKQEPMDVPVYEG----------ALPSQACANTSRSTPQP--------AQWPGYKINGSIISNNWDGHLNRRQSPDVSSLNSDKLQLH
Cave_fish (ENSAMXP00000015689)   -VIKQEPTEVPLYDNR--------TDGQVRSCPNTPSVTPQP--------DAWSGHKPNGSLASKGWDGNLR-PGVAHSPFTPDKQRQLH
Fugu (ENSTRUP00000036865)        -VIKQEPMDVPVYEG----------TTPGRATPNTPSTTPQP--------APWPGHRINGSIGPQSWERHLSPKPRPEASIVNPDQPQFL
Medaka (ENSORLP00000019236)      -LIKQEPMDVPVYEG----------ALPNQAGANTSRTTPQP--------AAWPGYKPN--IIPTTWDDHLKRKQSSEGSSMNLDKQ---
Platyfish (ENSXMAT00000000130)   -LIKQEPMDVPVYEG----------ALPSQACANTSRSTPQS--------AQWPGYKINGSIISNNWDGHLNRRQSPDVSSLNSDKLQLH
Stickleback (ENSGACP0000001732   -VIKQEPMDVPVYEG----------TTHGQAGAKTPSNTPQP--------AAWQGHKFNVPPSSS-PSPHLGTAFPGNMHQGSTRPS-TP
Tetraodon (ENSTNIP00000019883)   -VIKQEPLDVPVYEG----------STPGRATPHTPGTTPQP--------APWSAHRVNG-----SCDSPLNLRAGPDGPAVNPEQ--FH
Tilapia (ENSONIP00000019943)     -VIKQEPMDVPVYEG----------TLPNQVGANTSRSTPQP--------VTWSGHKLNQWSSFLGLNTMRASPAPSPSLQVPPSPSPSP
Zebrafish (ENSDARP00000085229)   -IVKQEPMDVPLYEAR--------SDGQAQSCPTTPSTTPTP--------EGWHGHKPNGSLVPKGWDGNIR-AASNNSPFTPDKQR-LH
Rainbow_trout (XP_021462005.1)   -VIKQEPMDMPVYKE-----------VNAQSCPSMPSTTLQPGA----AGGPWHGHKPNGIRVPSSWEGNLQPPGPPEAPFTSDKQQ-FH
Rainbow_trout (XP_021477599.1)   -VIKQEPMDVPVCEG-----------GNTQSCPDTPIPTPQP-------GGPWSGHKPNGSMVPGSWEGNPQPPCPPGAPFTSDKQQ-VH
Salmon (XP_014026412.1)          -VIKQEPIDMPVYKG-----------VNAQSCPSTPSTTPQPGA----AGGPWHGHKPNGIRVPSSWEGNLQPPGPPEAPFTSDKQQ-FH
Salmon (XP_014017100.1)          -VIKQEPMDVPVYEG-----------GNTQSCPDTPIPAPQP-------EGPWPGHKPNGSMVPGSWEGNLQPPCPPEAPFTSDKQQ-LH
```

DSBH-domain

```
                                       2710      2720      2730      2740      2750      2760      2770      2780      2790
                                 ....|....|....|....|....|....|....|....|....|....|....|....|....|....|....|....|....|....|
European ampioxus (Sc0000095)    ------------------------------------------------------------------------------------------
Ciona intestinalis (XP_0021254   ------------------------------------------------------------------------------------------
Lamprey (scaf_00044)             ------------------------------------------------------------------------------------------
Lamprey (scaf_03335)             ------------------------------------------------------------------------------------------
Shark (XP_007897316.1)           IPPKAENNEEP-------------------------------------------------------------------------------
Lizard (XP_020663651.1)          SANVSKLSGSE-------------------------------------------------------------------------------
Chicken (ENSGALP00000053457)     ------------------------------------------------------------------------------------------
Turtle (ENSPSIP00000006704)      ------------------------------------------------------------------------------------------
Human (ENSP00000362748)          ------------------------------------------------------------------------------------------
Gorilla (ENSGGOP00000011536)     ------------------------------------------------------------------------------------------
Mouse (ENSMUSP00000133279)       ------------------------------------------------------------------------------------------
Rat (ENSRNOP00000054747)         ------------------------------------------------------------------------------------------
Pig (ENSSSCP00000010918)         ------------------------------------------------------------------------------------------
Whale (XP_023987950.1)           ------------------------------------------------------------------------------------------
Elephant (ENSLAFP00000005179)    ------------------------------------------------------------------------------------------
Armadillo (ENSDNOP00000014565)   ------------------------------------------------------------------------------------------
Tasmanian_devil (XP_023360087.   TVTEQVPSDEL-------------------------------------------------------------------------------
Coelacanth (ENSLACP00000016889   ---KMQSSEIP-------------------------------------------------------------------------------
Cavefish(XP_007240334.2)         ------------------------------------------------------------------------------------------
Platyfish (XP_014325204.1)       ------------------------------------------------------------------------------------------
Tilapia (XP_005456583.2)         ------------------------------------------------------------------------------------------
Spotted_gar (XP_015202470.1)     ------------------------------------------------------------------------------------------
Stickleback (ENSGACT0000001035   ------------------------------------------------------------------------------------------
Tetraodon (ENSTNIT00000011701)   ------------------------------------------------------------------------------------------
Fugu (XP_011601624.1)            ------------------------------------------------------------------------------------------
Zebrafish (ENSDARP00000143362)   ------------------------------------------------------------------------------------------
Amazon_molly (XP_016534180.1)    ------------------------------------------------------------------------------------------
Medaka (XP_023818916.1)          ------------------------------------------------------------------------------------------
Rainbow_trout (XP_021469805.1)   ------------------------------------------------------------------------------------------
Rainbow trout (XP_021436521.1)   ------------------------------------------------------------------------------------------
Salmon (XP_014010485)            ------------------------------------------------------------------------------------------
Salmon (XP_014070638.1)          ------------------------------------------------------------------------------------------
Shark (XP_007890120.1)           -----------AYTSPYLAHDHR-------------------------------------------------------------------
Human (ENSP00000425443)          ------------HSPSHIIHNY--------------------------------------------------------------------
Gorilla (ENSGGOP00000034525)     ------------HSPSHIMHNY--------------------------------------------------------------------
Mouse (ENSMUSP00000143029)       ------------HLPSHTIYSYT-------------------------------------------------------------------
Rat (ENSRNOP00000048956)         ------------HLPPHMIHGYP-------------------------------------------------------------------
Pig (ENSSSCP00000009765)         ------------HPSSHLIHNY--------------------------------------------------------------------
Tasmanian_devil (ENSSHAT000000   ------------HPPPHLVHDY--------------------------------------------------------------------
Whale (XP_007106564.2)           ------------HPSSHLIHNY--------------------------------------------------------------------
Elephant (XP_023404313.1)        ------------HPSSHIIHNY--------------------------------------------------------------------
Lizard (ENSACAP00000008438)      -----------LYPPPHLTHDY--------------------------------------------------------------------
```

```
Chicken (ENSGALP00000017216)        -----------VQPPPHLARDY--------------------------------------------------------------------
Turtle (ENSPSIP00000011691)         -----------LHPPSHLAHDY--------------------------------------------------------------------
Xenopus (ENSXETP00000030770)        -------------PLPHSFQGHES------------------------------------------------------------------
Coelacanth (ENSLACP00000008576      ------------SQNPYVVQEH--------------------------------------------------------------------
Amazon_molly (ENSPFOP000000120      -----------EYPSSYLPQSP--------------------------------------------------------------------
Amazon_molly (ENSPFOP000000299      -----------EYPSSYLPQSP--------------------------------------------------------------------
Fugu (XP_011611191.1)               -----------GYPNSYLSRSP--------------------------------------------------------------------
Medaka (XP_004065962.1)             -----------EYPNTYLHQNP--------------------------------------------------------------------
Platyfish (ENSXMAP00000005447)      -----------EYPSSYLPQSP--------------------------------------------------------------------
Spotted _gar (ENSLOCP000000155      -----------RYPNPYLVQNS--------------------------------------------------------------------
Stickleback (ENSGACP0000002318      -----------GYPNPYLSRSP--------------------------------------------------------------------
Tilapia (XP_005457058.1)            -----------ECPNPYLPQNP--------------------------------------------------------------------
Tetraodon (ENSTNIP00000021201)      -----------GYPSSYLSRSP--------------------------------------------------------------------
Zebrafish (ENSDARP00000101295)      -----------AYPKPHISQNP--------------------------------------------------------------------
Salmon (XP_014042431.1)             -----------GYPNPYLSQST--------------------------------------------------------------------
Salmon (XP_014065146.1)             -----------GYPNPYLSQST--------------------------------------------------------------------
Salmon (XP_014050290.1)             -----------GYPNPYLSQST--------------------------------------------------------------------
Rainbow trout (XP_021429026.1)      -----------GYPNPYLSQST--------------------------------------------------------------------
Rainbow trout (XP_021429023.1)      -----------GYPNPYLSQST--------------------------------------------------------------------
Shark (XP_007910136.1)              PPHPGAG---------------------------------------HQDTQPRRRLPAQTLG----------FLQSRRRRRWGG------
Human (ENSP00000386869)             VPDKLSSFGASCLAPSHFTDG-------------------------QWGLFPGEGQQAASHS------GGRLRGKPWSPCKFGN------
Gorilla (ENSGGOP00000051172)        VPDKLSSFGASCLAPSHFTDG-------------------------QWGLFPGEGQQAASHS------GGRLRGKPWSPCKFGN------
Armadillo (ENSDNOP00000021516)      VSDKLGSFGAGCLTPSHFPDS-------------------------QWGLFPGEGPQPAPQP------GGRLRGKPWSPCKFGN------
Mouse (ENSMUSP00000087049)          VPDKLNSFGASCLTPSHFPES-------------------------QWGLFTGEGQQSAPHA------GARLRGKPWSPCKFGN------
Rat (ENSRNOP00000015296)Tet3        VPDKLNSFGASCLTPSHFPDS-------------------------QWGLFTGEGQQSAPHP------GGRLRGKPWSPCKFGN------
Pig (ENSSSCP00000008852)            VPDKLSSLGAACLTPSRFADGQH-----------------------QWGLFPSEGQQPAPQP------GGRLRSKPWSPCKFGN------
Elephant  (XP_010591957.2)          VPDKLGSFGAGCLTPSHFSDG-------------------------QWGLFPGEGQQPAPQP------GGRLRGKPWSPCKFGN------
Whale (XP_023989264.1)              VPDKLGSFGAGCLTPSHFPDGQH-----------------------QWGLFPGEGQQPAPQP------GGRLRGKPWSHCKFGN------
Tasmanian_devil (ENSSHAP000000      LSDKLGSFGASCLPASRLSDG-------------------------QWGLFPAEGGQPPQQPPLGPPNGASLLSKAWSPCKFRD------
Chicken (ENSGALT00000059756)        GREKLSAFDAAVRLLLP---------------------------------------------------------KPWSPCKLG-------
Lizard (ENSACAT00000001585)         GQNKPGLFGSHSASRLPPQNHLRE---------------------KPWSLFPREGQPMEE--------------KPWSPCKGDDGSSA--
Turtle (ENSPSIT00000006549)         MQDKLNSFTGHSNATRLPQSHLQE---------------------KQWNVFPGEGQATQQSS--------SLLGKSWSPCKLNE------
Xenopus (ENSXETP00000054061)        GLEKKDYFGVHSNAPGLKDKQWPP---------------------YGTDVSVRQHDSLDSQS--------P--GKVWSSCKLSD------
Coelacanth (ENSLACP00000009535      VQEACSSFKVNLNASRLSNAHVQE---------------------TQWNSFTNGSQAAQHTG--------NLQGKPWSTYKLSK------
Spotted_gar (ENSLOCP0000001981      -------------------------------------------SPSPSPSLPLTP----SPAPSC----SPHVPNTSWGTFGS-------
Amazon_molly (ENSPFOP000000021      QLHQHTQ------QRQSSPY------------------------PQQWTSHPCS----NPQIAST----NPALQTPGQLTP---------
Cave_fish (ENSAMXP00000015689)      QQHHNQP-------QHPQYAHQQ----------------------QHWGSY--PGTPIASPAPSP----SPALKGGPSPAPSPHP-----
Fugu (ENSTRUP00000036865)           QQPSPFP------HQWSSFP--NAAMPPAPASPSLQ------VPPSPSHPGPGLP--------GN----LHQSSTPRPSTP---------
Medaka (ENSORLP00000019236)         QFHQHSQ------QQQSSPF------------------------PQQWTSFPGS----NLPIASS----SPSLQVTPSPPPSPHLGTVIQ
Platyfish (ENSXMAT00000000130)      KLHQHAQ------QRQSSPY------------------------PQQWTSHPCS----NPQIAST----NPAHQVPASPPPSPHLGTGHQ
Stickleback (ENSGACP0000001732      RSNTPQT------GSVTTH-------------------------PGPGTPGPGTPGPGTPGPGT------PGPGTPGPGTPGP-------
Tetraodon (ENSTNIP00000019883)      QQHSPYP------PQWASYPAPNAAMPPAAASPSLQGNLHQSSTPRPSTPRSGTPQPGSCRSGAA----TPQPGTPGPGTPGP-------
Tilapia (ENSONIP00000019943)        QLGTVVQ------GNIHQAS------------------------PRPGTPRPSTPHSVTPQPGVS----HSGPLTPRPSTPRP-------
Zebrafish (ENSDARP00000085229)      QQHQH---------EHPQYP-QQ----------------------QQWNSYSCPNTPMASPAPSP----SPSLKAGLSPAPSPHP-----
Rainbow_trout (XP_021462005.1)      QQGPRQTFQSPYLQEQHQHPPQQP-----------------SPYSQQWNSYHGPNTPTASPAPSP----SPSIKIPPSPFPSSHP-----
Rainbow_trout (XP_021477599.1)      QQGPHQTSQSPYSQQQHQYPPQQP-----------------SPYPQQWNSYPGPNTPKASPVPS------------PSPSPSPHP-----
Salmon (XP_014026412.1)             QQGPHQTFQSPYPQEQHQHPPQQP-----------------SPYSQQWNSYHGPNTPTASPAPSL----SPSIKIPPSPFPSSHP-----
Salmon (XP_014017100.1)             QQGPHQTSQSPYS--QHQHPPQQP-----------------SPYTQQWNSYPGPNTPKASPVPS------------PSPSPSPHP-----
```



```
                                             2800      2810      2820      2830      2840      2850      2860      2870      2880
                                    ....|....|....|....|....|....|....|....|....|....|....|....|....|....|....|....|....|....|
European ampioxus (Sc0000095)       ------------------------------------------------------------------------------------------
Ciona intestinalis (XP_0021254      ------------------------------------------------------------------------------------------
Lamprey (scaf_00044)                ------------------------------------------------------------------------------------------
Lamprey (scaf_03335)                ------------------------------------------------------------------------------------------
Shark (XP_007897316.1)              ------------------------------------------------------------------------------------------
Lizard (XP_020663651.1)             ------------------------------------------------------------------------------------------
Chicken (ENSGALP00000053457)        ------------------------------------------------------------------------------------------
Turtle (ENSPSIP00000006704)         ------------------------------------------------------------------------------------------
Human (ENSP00000362748)             ------------------------------------------------------------------------------------------
Gorilla (ENSGGOP00000011536)        ------------------------------------------------------------------------------------------
Mouse (ENSMUSP00000133279)          ------------------------------------------------------------------------------------------
Rat (ENSRNOP00000054747)            ------------------------------------------------------------------------------------------
Pig (ENSSSCP00000010918)            ------------------------------------------------------------------------------------------
Whale (XP_023987950.1)              ------------------------------------------------------------------------------------------
Elephant (ENSLAFP00000005179)       ------------------------------------------------------------------------------------------
Armadillo (ENSDNOP00000014565)      ------------------------------------------------------------------------------------------
Tasmanian_devil (XP_023360087.      ------------------------------------------------------------------------------------------
Coelacanth (ENSLACP00000016889      ------------------------------------------------------------------------------------------
Cavefish(XP_007240334.2)            ------------------------------------------------------------------------------------------
Platyfish (XP_014325204.1)          ------------------------------------------------------------------------------------------
Tilapia (XP_005456583.2)            ------------------------------------------------------------------------------------------
Spotted_gar (XP_015202470.1)        ------------------------------------------------------------------------------------------
Stickleback (ENSGACT0000001035      ------------------------------------------------------------------------------------------
Tetraodon (ENSTNIT00000011701)      ------------------------------------------------------------------------------------------
Fugu (XP_011601624.1)               ------------------------------------------------------------------------------------------
Zebrafish (ENSDARP00000143362)      ------------------------------------------------------------------------------------------
Amazon_molly (XP_016534180.1)       ------------------------------------------------------------------------------------------
Medaka (XP_023818916.1)             ------------------------------------------------------------------------------------------
Rainbow_trout (XP_021469805.1)      ------------------------------------------------------------------------------------------
Rainbow trout (XP_021436521.1)      ------------------------------------------------------------------------------------------
Salmon (XP_014010485)               ------------------------------------------------------------------------------------------
Salmon (XP_014070638.1)             ------------------------------------------------------------------------------------------
```

```
Shark (XP_007890120.1) --------------------------------------------------PANSFQIENKENELNMLRANG------LSRLLPSINFDMA
Human (ENSP00000425443) -----------------------------------------SAAPGMFNSSLHALHLQNKENDMLSHTANG------LSKMLPALNHDRT
Gorilla (ENSGGOP00000034525) -----------------------------------------SAAPGMFNSSLHALHLQNKENDMLSHTANG------LSKMLPALNHDRT
Mouse (ENSMUSP00000143029) -----------------------------------------AAASG--SSSSHAFHNKEND-----NIANG------LSRVLPGFNHDRT
Rat (ENSRNOP00000048956) -----------------------------------------TAASGSVSSSSHAFHNKENDN-MVSHTANG------LSRVLPGFNHDRT
Pig (ENSSSCP00000009765) -----------------------------------------SAAPSMFNSSLHALHLQNKENDMLSHTTNG------FSKMLPGLNHDRT
Tasmanian_devil (ENSSHAT000000 -----------------------------------------HAVSGIFTSPSHSFHLPNKEREMLSHTTNE------LSKMLPGFNHDRT
Whale (XP_007106564.2) -----------------------------------------SAAPGMFNSSLHALHLPNKENDVLSHTANG------LSKTLPGLNHDGT
Elephant (XP_023404313.1) -----------------------------------------RAAPGMFNSSLHALPLQNKENDVLSHTANG------LLKMLPGLNHDGT
Lizard (ENSACAP00000008438) -----------------------------------------QSTAGMFSGPPHSLHLQNKD----SQTTNG------LSNMLPDLNPNRT
Chicken (ENSGALP00000017216) -----------------------------------------HSASSMFSGPPNSLHLQNKDSEMISHAVNG------LSNTIPGQNHDRT
Turtle (ENSPSIP00000011691) -----------------------------------------HSASNIFSGPPHSLHLQNKDSEMTSHIANG------LSNMLPGLNHDRT
Xenopus (ENSXETP00000030770) -----------------------------------------NMSSSILGGRAGSLPFPGKQSEMHSRTANG------TSEMLPGLNHDRT
Coelacanth (ENSLACP00000008576 -----------------------------------------QSASGLFSNVPNTSHLQNQEADLNLLTING------ISRMLPSLNHNRA
Amazon_molly (ENSPFOP000000120 -------------------------------------------HIP----GRDSFPMQLNTDVAMPNP-----QMLPAQISSGCLNPETQ
Amazon_molly (ENSPFOP000000299 -------------------------------------------HIP----GRDSFPMQLNTDVAMPNP-----QMLPAQISSGCLNPETQ
Fugu (XP_011611191.1) -------------------------------------------QVLPP--GQDPFHMQIKTEIGIPHPNS---HPLSSQIGGGCLNSDTK
Medaka (XP_004065962.1) -------------------------------------------HILPS--GLDPLHTQVKTEMGIPDP-----QMLSAQLRGRCLNPDTQ
Platyfish (ENSXMAP00000005447) -------------------------------------------HIP----GRDSFPMQLNTDVAIPNP-----QMLPAQISSGCLNPETQ
Spotted _gar (ENSLOCP000000155 -------------------------------------------SVFSS--VHDSFHMQHKTEVNLHGPNG--IARVLPTLGNECPNLSQL
Stickleback (ENSGACP0000002318 -------------------------------------------QIFAP--GQDPFHMQIKTEAGTPRP-----QVLSAPLGGGCYNPEAQ
Tilapia (XP_005457058.1) -------------------------------------------QMLHP--GQDPFHLQIKAEMGMPGP-----QMLSAQSGSMCLNPETQ
Tetraodon (ENSTNIP00000021201) -------------------------------------------QILPP--GQDSFHMQIKTEMGVPHP-------LSAQIGGGCLTSDTK
Zebrafish (ENSDARP00000101295) -------------------------------------------HMFSP--NLNTLSMQSHKDLGISMHEANGISQGFPPLGKECFNFNQP
Salmon (XP_014042431.1) -------------------------------------------KMFPPGSPQDPFCMQVKTEMGLQS---------------PCMTPTQP
Salmon (XP_014065146.1) -------------------------------------------KMFPPGSPQDPFCMQVKTEMGLQS---------------PCMTPTQP
Salmon (XP_014050290.1) -------------------------------------------KMFPPGGPQDPFCMQVKTEMGLQS---------------PCMTPTQP
Rainbow trout (XP_021429026.1) -------------------------------------------KMFPPGGPQDPFCMQVKTEMGLQS---------------PCMTPTQP
Rainbow trout (XP_021429023.1) -------------------------------------------KMFPPGGPQDPFCMQVKTEMGLQSP-------ISCQSNFPCMTPTQP
Shark (XP_007910136.1) ---------------------------------------ARHVVHTSPPPAAAAAAP--PPAAHRP------------QEKPWDLFASGK
Human (ENSP00000386869) ---------------------------------------STS-ALAGPSLTEKPWAL--GAGDFNSALKGS----PGFQDKLWNPMKGEE
Gorilla (ENSGGOP00000051172) ---------------------------------------STS-ALAGPSLTEKPWAL--GAGDFNSALKGS----PGFQDKLWNPMKGEE
Armadillo (ENSDNOP00000021516) ---------------------------------------ATATSLTAPGLTEKPWGV--VAGDFNSALKGG----PGFQEKLWSPMKGEE
Mouse (ENSMUSP00000087049) ---------------------------------------GTS-ALTGPSLTEKPWGM--GTGDFNPALKGG----PGFQDKLWNPVKVEE
Rat (ENSRNOP00000015296)Tet3 ---------------------------------------GTS-ALSGPSLPEKPWGM--GAGDFNSSLKGG----PGFQDKLWNPVKVEE
Pig (ENSSSCP00000008852) ---------------------------------------TPT-ALSGPSLTEKSWGV--GAGDLTSALKGG----PGFQDKLWSPLKGEG
Elephant  (XP_010591957.2) ---------------------------------------SAS-ALAGPGLTEKPWSI--GAGDFNSALKGS----AGYQEKLWSPMKGEE
Whale (XP_023989264.1) ---------------------------------------NTS-ALAGPSLTEKPWAV--GAGDFNSALKGG----PRFQDKLWSPLKGEE
Tasmanian_devil (ENSSHAP000000 ---------------------------------------STS-TLNGPSLAEKPWGV--AP-DFGPALKGA----PGYPEKLWGPVKGDE
Chicken (ENSGALT00000059756) --------------------------------------ETVLGGAGTPTLRDKGWEL--GPLGFGSALPEL----PVFSEEPWGSGKAEE
Lizard (ENSACAT00000001585) -------------------------------------PNSVFAAPGS-DLQEKRWGF--GQLDLSSIKGSG----RTLG-DLWGPVKADS
Turtle (ENSPSIT00000006549) -------------------------------------SPSALPSSANSNLQEKPWHL--PQLNYSSTLKGN----SGFQDELWNSVKVDE
Xenopus (ENSXETP00000054061) -------------------------------------SSAALPSSAS--TQDKNWNG--RQVSLNQGMKES----ALFQEKLWNSVAASD
Coelacanth (ENSLACP00000009535 -------------------------------------TPSPLPGSANGNLGAPSWNL--ASMNLTAAIKTN----SELQDKLWHSVKINE
Spotted_gar (ENSLOCP0000001981 ---------------------------------------PGEGCCPSPNPQGKSWNS----NGYNANLKRA-TPVGGFPDKLWC--KDGE
Amazon_molly (ENSPFOP000000021 ----------------------------------------RHWASPAPSPQPNPWGF--SPAAYSPGLKHS-NPAGAYPDKIWS--KTGE
Cave_fish (ENSAMXP00000015689) -------------------------------------STPHHWESPVPSPQPKAWGP-IGSAGFGPGGLRQGTPVGAFPDKMLS--QTVE
Fugu (ENSTRUP00000036865) ------------------------------------------------RPQPNAWAM--GPVAYSPALKHS-NPAGACPDKIWS--KTGE
Medaka (ENSORLP00000019236) ASLQGSTRHVSPHPGTPLQPGTSHSRSLTPQPGLQGPGLPRHLASPASSPQPNAWAM--GPAPCSPGLKHS-NPAGAYPDKMWS--KTGE
Platyfish (ENSXMAT00000000130) GNLY--------------------------------RGAIRHSASYPGTPQP-------VTSHSGPGLKHS-NPAGAYPDKIWS--KTGE
Stickleback (ENSGACP0000001732 -------------------------------------GTPRHWASPAPSPQPNAWAM--GPAAYSPGLKHS-NPAGAYADKIWS--KTGE
Tetraodon (ENSTNIP00000019883) -------------------------------------GTLMHWASPAPSPQPNAWGV--GPLAYSPALKHS-NPAGACPDKIWS--KTGE
Tilapia (ENSONIP00000019943) -------------------------------------GTPRHWVSPVASPQPQTWGM--GP--YSPGMKHS-NPAGAYPDKIWS--KTGE
Zebrafish (ENSDARP00000085229) -------------------------------------STPRQWGSPAPSPQPKAWGP-YGPMGYGAGVIRQGNPAGAYPDNMLS--QAGV
Rainbow_trout (XP_021462005.1) -------------------------------------GTPHHWDSPDSSPQPKAWPMSMVPAGYSP------SPVGGFPDKMWS--KAGE
Rainbow_trout (XP_021477599.1) -------------------------------------GTPRHWDSPAPSPQPKAWPMDMVPAGYCP------SPARGFPDKICS--KAGE
Salmon (XP_014026412.1) -------------------------------------GTPRHWDSPDSSPQPKAWPMGMVPAGYSP------SPAGGFPDKMWS--KAGE
Salmon (XP_014017100.1) -------------------------------------GTPRHWDSPAPSPQPKAWPMGMVPAGYCP------SPARGFPDKICS--KAGE
```

DSBH-domain

```
          2890      2900      2910      2920      2930      2940      2950      2960      2970
      ....|....|....|....|....|....|....|....|....|....|....|....|....|....|....|....|....|....|
European ampioxus (Sc0000095) ------------------------------------------------------------------------------------------
Ciona intestinalis (XP_0021254 ------------------------------------------------------------------------------------------
Lamprey (scaf_00044) ------------------------------------------------------------------------------------------
Lamprey (scaf_03335) ------------------------------------------------------------------------------------------
Shark (XP_007897316.1) ------------------------------------------------------------------------------------------
Lizard (XP_020663651.1) ------------------------------------------------------------------------------------------
Chicken (ENSGALP00000053457) ------------------------------------------------------------------------------------------
Turtle (ENSPSIP00000006704) ------------------------------------------------------------------------------------------
Human (ENSP00000362748) ------------------------------------------------------------------------------------------
Gorilla (ENSGGOP00000011536) ------------------------------------------------------------------------------------------
Mouse (ENSMUSP00000133279) ------------------------------------------------------------------------------------------
Rat (ENSRNOP00000054747) ------------------------------------------------------------------------------------------
Pig (ENSSSCP00000010918) ------------------------------------------------------------------------------------------
Whale (XP_023987950.1) ------------------------------------------------------------------------------------------
Elephant (ENSLAFP00000005179) ------------------------------------------------------------------------------------------
Armadillo (ENSDNOP00000014565) ------------------------------------------------------------------------------------------
Tasmanian_devil (XP_023360087. ------------------------------------------------------------------------------------------
Coelacanth (ENSLACP00000016889 ------------------------------------------------------------------------------------------
Cavefish(XP_007240334.2) ------------------------------------------------------------------------------------------
Platyfish (XP_014325204.1) ------------------------------------------------------------------------------------------
Tilapia (XP_005456583.2) ------------------------------------------------------------------------------------------
Spotted_gar (XP_015202470.1) ------------------------------------------------------------------------------------------
Stickleback (ENSGACT0000001035 ------------------------------------------------------------------------------------------
```

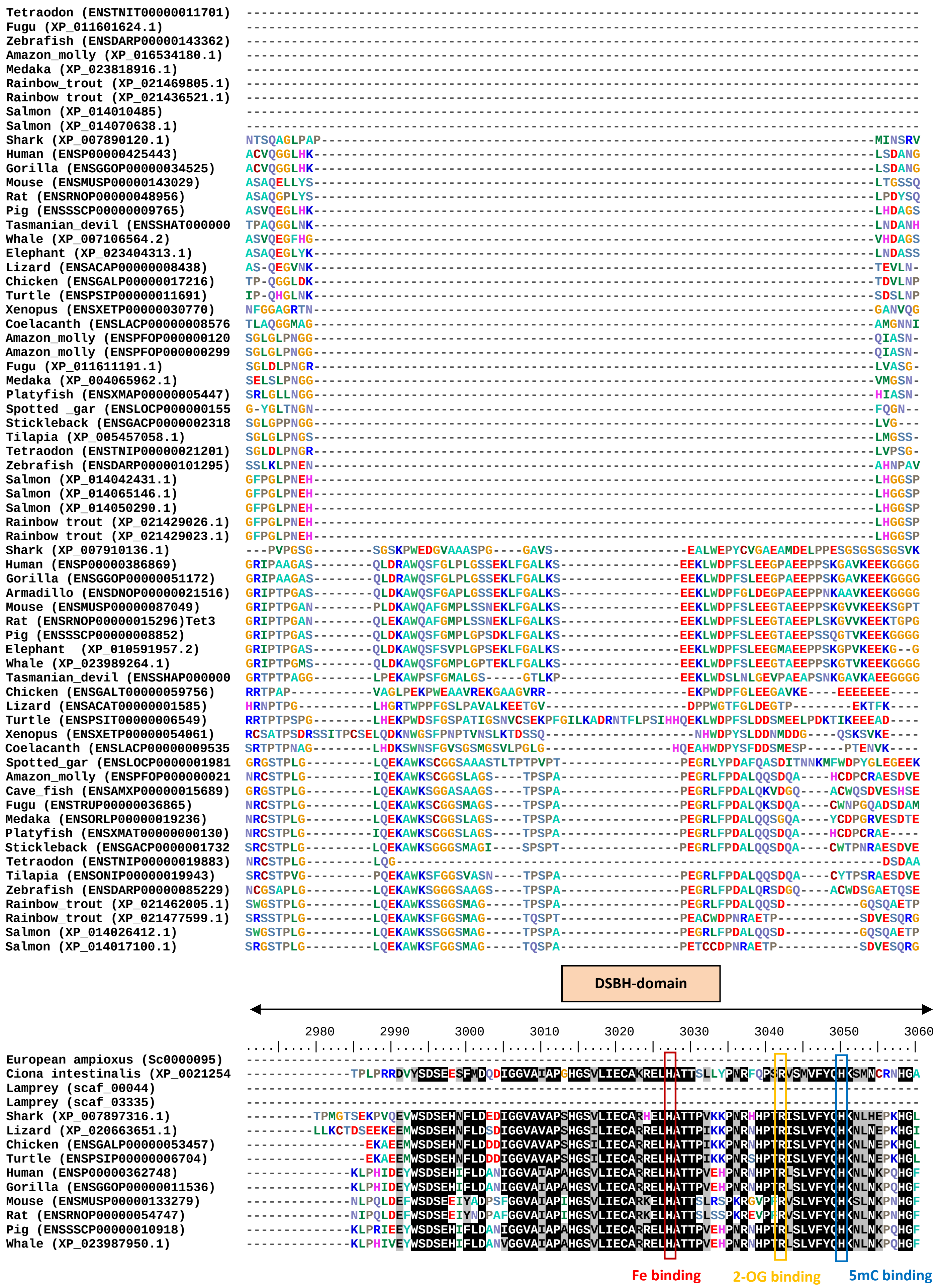

Tetraodon (ENSTNIT00000011701)
Fugu (XP_011601624.1)
Zebrafish (ENSDARP00000143362)
Amazon_molly (XP_016534180.1)
Medaka (XP_023818916.1)
Rainbow_trout (XP_021469805.1)
Rainbow trout (XP_021436521.1)
Salmon (XP_014010485)
Salmon (XP_014070638.1)
Shark (XP_007890120.1)
Human (ENSP00000425443)
Gorilla (ENSGGOP00000034525)
Mouse (ENSMUSP00000143029)
Rat (ENSRNOP00000048956)
Pig (ENSSSCP00000009765)
Tasmanian_devil (ENSSHAT000000
Whale (XP_007106564.2)
Elephant (XP_023404313.1)
Lizard (ENSACAP00000008438)
Chicken (ENSGALP00000017216)
Turtle (ENSPSIP00000011691)
Xenopus (ENSXETP00000030770)
Coelacanth (ENSLACP00000008576
Amazon_molly (ENSPFOP000000120
Amazon_molly (ENSPFOP000000299
Fugu (XP_011611191.1)
Medaka (XP_004065962.1)
Platyfish (ENSXMAP00000005447)
Spotted _gar (ENSLOCP000000155
Stickleback (ENSGACP0000002318
Tilapia (XP_005457058.1)
Tetraodon (ENSTNIP00000021201)
Zebrafish (ENSDARP00000101295)
Salmon (XP_014042431.1)
Salmon (XP_014065146.1)
Salmon (XP_014050290.1)
Rainbow trout (XP_021429026.1)
Rainbow trout (XP_021429023.1)
Shark (XP_007910136.1)
Human (ENSP00000386869)
Gorilla (ENSGGOP00000051172)
Armadillo (ENSDNOP00000021516)
Mouse (ENSMUSP00000087049)
Rat (ENSRNOP00000015296)Tet3
Pig (ENSSSCP00000008852)
Elephant (XP_010591957.2)
Whale (XP_023989264.1)
Tasmanian_devil (ENSSHAP000000
Chicken (ENSGALT00000059756)
Lizard (ENSACAT00000001585)
Turtle (ENSPSIT00000006549)
Xenopus (ENSXETP00000054061)
Coelacanth (ENSLACP00000009535
Spotted_gar (ENSLOCP0000001981
Amazon_molly (ENSPFOP000000021
Cave_fish (ENSAMXP00000015689)
Fugu (ENSTRUP00000036865)
Medaka (ENSORLP00000019236)
Platyfish (ENSXMAT00000000130)
Stickleback (ENSGACP0000001732
Tetraodon (ENSTNIP00000019883)
Tilapia (ENSONIP00000019943)
Zebrafish (ENSDARP00000085229)
Rainbow_trout (XP_021462005.1)
Rainbow_trout (XP_021477599.1)
Salmon (XP_014026412.1)
Salmon (XP_014017100.1)
DSBH-domain
2980 2990 3000 3010 3020 3030 3040 3050 3060
European ampioxus (Sc0000095)
Ciona intestinalis (XP_0021254
Lamprey (scaf_00044)
Lamprey (scaf_03335)
Shark (XP_007897316.1)
Lizard (XP_020663651.1)
Chicken (ENSGALP00000053457)
Turtle (ENSPSIP00000006704)
Human (ENSP00000362748)
Gorilla (ENSGGOP00000011536)
Mouse (ENSMUSP00000133279)
Rat (ENSRNOP00000054747)
Pig (ENSSSCP00000010918)
Whale (XP_023987950.1)
Fe binding
2-OG binding
5mC binding

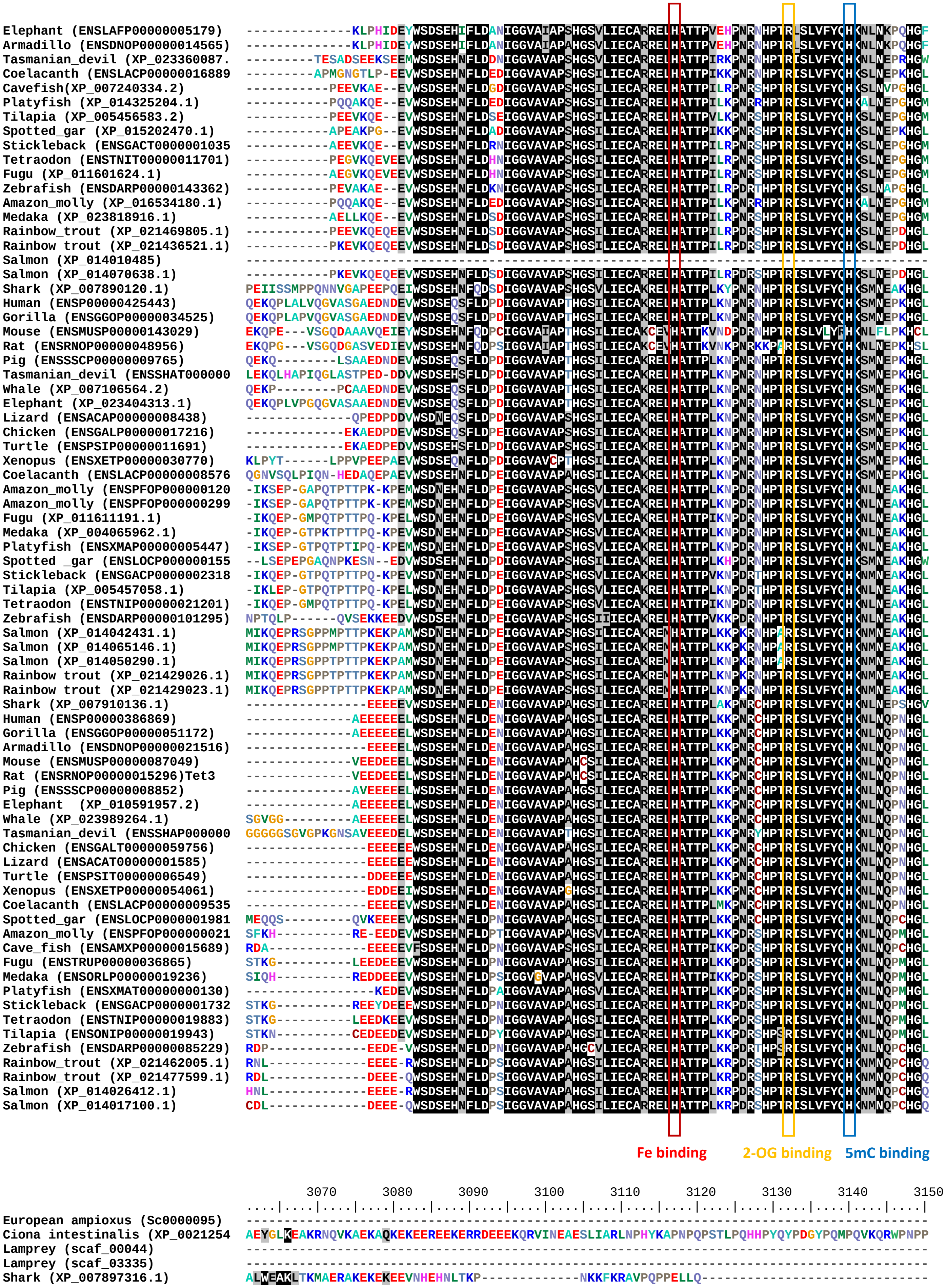
Fe binding
2-OG binding
5mC binding
3070
3080
3090
3100
3110
3120
3130
3140
3150
European ampioxus (Sc0000095)
Ciona intestinalis (XP_0021254
Lamprey (scaf_00044)
Lamprey (scaf_03335)
Shark (XP_007897316.1)

```
Lizard (XP_020663651.1)          ALWEAKMAERAKEK--EAEKLNTEND--SQQ------------PSDRNAHQASATRENVY------------------------------
Chicken (ENSGALP00000053457)     AMWEAKMAERAKEKEKEAERLGTENT--ELN------------SSSRKTKQTSENREIFY------------------------------
Turtle (ENSPSIP00000006704)      AMWEAKMAERAKEKEKEAE--------------------------SRKTKQSNEAREIFY------------------------------
Human (ENSP00000362748)          ELNKIKFEAKEAKNKKMKA-------------------------SEQKDQAANEGPEQSS------------------------------
Gorilla (ENSGGOP00000011536)     ELNKIKFEAKEAKNKKMKA-------------------------SEQKDQAANEGPEQSS------------------------------
Mouse (ENSMUSP00000133279)       DINKIKCKCKKVTKK---------------------------------KPADRECPDVSP------------------------------
Rat (ENSRNOP00000054747)         DINRIKCKCKKVTKK---------------------------------PPADREDPDLSP------------------------------
Pig (ENSSSCP00000010918)         ELNKIKFEAKEAKNKKIKA-------------------------SEQKDQAANEGPELSP------------------------------
Whale (XP_023987950.1)           ELNKIKFEAKEAKNKKTKA-------------------------SEQKDQAANEGPELST------------------------------
Elephant (ENSLAFP00000005179)    ELNKIKFEAKEAKNKKTKA-------------------------SEQKDQAANEGPELSP------------------------------
Armadillo (ENSDNOP00000014565)   ELNKIKFEAKEAKNKKTKA-------------------------LEQKDQAANEGSELSP------------------------------
Tasmanian_devil (XP_023360087.   AVWEAKMAERAREKEREAEKLATEN------------------AMNQKTKQPPESPEIFC------------------------------
Coelacanth (ENSLACP00000016889   ALWEAKMAERAK-EREEEAERLGYESGVTKS------------GNKKGKQAASDNRDELQ------------------------------
Cavefish(XP_007240334.2)         LRWEAKMAEKAREKEEEAMRLG----LETEGLGS---AKTRKSQPAAGSDTESGEEDVWQ------------------------------
Platyfish (XP_014325204.1)       AMWDAKMAKRERDREEEAERL----RMEESG------GMGKNRKTSGAGEGEEDEDKETA------------------------------
Tilapia (XP_005456583.2)         AMWDAKMAKRERDREEEAERL----KLEGSL------SKG-----AGGVEGEEETGEETE------------------------------
Spotted_gar (XP_015202470.1)     ALWEAKMAERAKEREEEAERLG-GAEGGP----------ARPRNKRAKPGGGAGKEEVRQ------------------------------
Stickleback (ENSGACT0000001035   AMWDAKMAKREREREEEAERL----RMEECI------GKS----------GEEAEG----------------------------------
Tetraodon (ENSTNIT00000011701)   AMWDAKMAKREREREEEAERL----RLEEMP------GGSAVMKGGRAGRSQEESREGEK------------------------------
Fugu (XP_011601624.1)            AMWDAKMAKREREREEEAERL----RLEEMP------GGSALKKGGKAGGLQEETKEEEE------------------------------
Zebrafish (ENSDARP00000143362)   QQWEAKRAEKAREKEQ-AERLG----LNNQ------------SEPVD-SDTDS-ENETCL------------------------------
Amazon_molly (XP_016534180.1)    AMWDAKMAKREREREEEAERL----RMEGSG------GAGKNRKTSGGREGEEDEDKETA------------------------------
Medaka (XP_023818916.1)          AMWDAKMAKRERDREEEAERS----RIEAGL------GKD------GKDGKERREEDAGE------------------------------
Rainbow_trout (XP_021469805.1)   HMWEAKMARREREREEEAERIGMGLDLEEVSPVKGKGKRGKGAGAESVEPEEEVEEEGPE------------------------------
Rainbow trout (XP_021436521.1)   HMWEAKTARREREREEEAERLGMGLDLEEVSPVKG--KRGNGAGSESVEPEEEVEEEGPE------------------------------
Salmon (XP_014010485)            ------------------------------------------------------------------------------------------
Salmon (XP_014070638.1)          HMWEAKKARREREREEEAERLGMGLDLEEVSLVKG--KRGNGAGGESVEPEEEVEEEGPE------------------------------
Shark (XP_007890120.1)           AMWEAKVAEKAREREEEAERLGVDY----------VPTKSYSKKSKREVTD-SHELAEAA------------------------------
Human (ENSP00000425443)          ALWEAKMAEKAREKEEECEKYGPDY----------VPQKSHGKKVKREPAE-PHETSEPT------------------------------
Gorilla (ENSGGOP00000034525)     ALWEAKMAEKAREKEEECEKYGPDY----------VPQKSHGKKVKREPAE-PHETSEPT------------------------------
Mouse (ENSMUSP00000143029)       ALWEAKMAEKARKEEE-CGKNGSDH----------VSQKNHGKQEKREPTG-PQE---PS------------------------------
Rat (ENSRNOP00000048956)         AVWEAKMAGKARKEEEECEKYGSDH----------VSQKNHGKRVKREPTG-PQETPKPS------------------------------
Pig (ENSSSCP00000009765)         ALWEAKMAEKAREKEEECEKYGPDY----------VPQKTHGKKVKREPTE-PHEPSEPT------------------------------
Tasmanian_devil (ENSSHAT000000   ALWEAKMAEKAREKEEECEKYGPDY----------VPQKTYGKK-KREPVE-PHEPSEPT------------------------------
Whale (XP_007106564.2)           ALWEAKMAEKAREKEEECEKYGPDY----------VPQKTHGKKVKREPTE-PHEPAEPT------------------------------
Elephant (XP_023404313.1)        ALWEAKMAEKAREKEEECEKYGPDY----------VPQKIHGKKVKREPPE-PHEPSEPT------------------------------
Lizard (ENSACAP00000008438)      ALWEAKMAEKAREKEEDCERYGPDY----------VPQKVHGKKVKREPVE-LNENLEPT------------------------------
Chicken (ENSGALP00000017216)     ALWEAKMAEKAREKEEECEKYGPDY----------VPQKSYGKKAKREPAE-PHEPSEPT------------------------------
Turtle (ENSPSIP00000011691)      ALWEAKMAEKAREKEEECEKYGPDY----------VPQKSYGKKSKREPIE-SHEPSEPT------------------------------
Xenopus (ENSXETP00000030770)     ALWEAKMAGKAREKEEDCERYGPDY----------VAPKSYNRKAKREPVEPIHEPAEPT------------------------------
Coelacanth (ENSLACP00000008576   ALWEAKMAEKAREKEEESEKHGSDY----------VPNKSHNKKAKREPSE-SQDTSEPP------------------------------
Amazon_molly (ENSPFOP000000120   AMWEAKMAEKAREKEEEAERNGGEG----------TPSKSSKKGGKREHSESSEATGEPP------------------------------
Amazon_molly (ENSPFOP000000299   AMWEAKMAEKAREKEEEAERNGGEG----------TPSKSSKKGGKREHSESSEATGEPP------------------------------
Fugu (XP_011611191.1)            AMWEAKMAEKAREKEEDAERNGGEG----------TPSKG-KKGVKREHPESSESTAEPP------------------------------
Medaka (XP_004065962.1)          ALWEAKMAEKAREKEEEAERNGGEG----------TPTKGNKKGVKREHPES-ETTGEPP------------------------------
Platyfish (ENSXMAP00000005447)   AMWEAKMAEKAREKEEDAERNGGEG----------TPSKSSKKGGKREHSESSETTGEPP------------------------------
Spotted _gar (ENSLOCP000000155   ALWEAKMAEKAREKEEESEKQGSDN----------GPIRSGSKKIKREHSEVLDQS-EPP------------------------------
Stickleback (ENSGACP0000002318   ALWEAKMAEKAREKEEDAERNGGEG----------TPSKG-KKGAKREHIET-ESTGEPP------------------------------
Tilapia (XP_005457058.1)         ALWEAKMAEKAREKEEEAERNGGEG----------TPSKSSKKGPKREHLESSETTGEPP------------------------------
Tetraodon (ENSTNIP00000021201)   AMWEARMAEKAREKEEDAEKNGGEG----------TPSKG-KKGVKREHSESSESTVEPP------------------------------
Zebrafish (ENSDARP00000101295)   SLWEAKMAEKAREKEEDAEKHGAEN----------TSSKSGGKKAKREHSEHSEPS-EPP------------------------------
Salmon (XP_014042431.1)          ALWEAKMAEKQREKDEDAERNGAEGAT-------VTPSKSSKKGAKREHPELSEQQGEPP------------------------------
Salmon (XP_014065146.1)          ALWEAKMAEKQREKDEDAERNGAEGAT-------VTPSKSSKKGAKREHPELSEQQGEPP------------------------------
Salmon (XP_014050290.1)          ALWEAKMAEKQREKDEDAERNGAEGTT-------VTPSKSTKKGAKREHPELSEQQGEPP------------------------------
Rainbow trout (XP_021429026.1)   ALWEAKMAEKQREKDEDAERTGAEGAT-------VTPSKSTKKGAKREHPELSEQQGEPP------------------------------
Rainbow trout (XP_021429023.1)   ALWEAKMAEKQREKDEDAERTGAEGAT-------VTPSKSTKKGAKREHPELSEQQGEPP------------------------------
Shark (XP_007910136.1)           ALWEAKMKLLAERARQRQEE-------AARLGLPLPGTAKLLDSAKIWAIKGRIASAPTSN-----------------------------
Human (ENSP00000386869)          ALWEAKMKQLAERARARQEE-------AARLGLGQQE-AKLYGKKRKWGG--TVVAEPQQ------------------------------
Gorilla (ENSGGOP00000051172)     ALWEAKMKQLAERARARQEE-------AARLGLGQQE-AKLYGKKRKWGG--TVVAEPQQ------------------------------
Armadillo (ENSDNOP00000021516)   ALWEAKVKQLAERARARQEE-------AARLGLGQQE-AKLYGKKRKWGG---AAAEPQH------------------------------
Mouse (ENSMUSP00000087049)       ALWEAKMKQLAERARQRQEE-------AARLGLGQQE-AKLYGKKRKWGG--AMVAEPQH------------------------------
Rat (ENSRNOP00000015296)Tet3     ALWEAKMKQLAERARQRQEE-------AARLGLGQQE-AKLYGKKRKWGG--AMVAEPQH------------------------------
Pig (ENSSSCP00000008852)         ALWEAKMKQLAERARARQEE-------AARLGLGQQE-AKLYGKKRKWGG--AMVTEPQH------------------------------
Elephant  (XP_010591957.2)       ALWEAKMKQLAERARARQEE-------AARLGLGQQE-AKLYGKKRKWGG--ALVAEPQH------------------------------
Whale (XP_023989264.1)           ALWEAKMKQLAERARARQEE-------AARLGLGQQE-AKLYGKKRKWGG--AVVPEPQH------------------------------
Tasmanian_devil (ENSSHAP000000   ALWEAKMKQLAERARAR-EE-------AARLGLSHPE-VKPYGKKRKWGG--AAAPEPQQ------------------------------
Chicken (ENSGALT00000059756)     ALWEAKMKQLAERARARQEE-------AARLGLQQD--AKAFAKKRKWGG--ALAAEAAT------------------------------
Lizard (ENSACAT00000001585)      ALWEAKVKQLAERARARQEEAEAAAAAAASLGLQVEE-GKGFGKKRKWGSNSGPAPESQP------------------------------
Turtle (ENSPSIT00000006549)      ALWEAKMKQLAERARARQEE-------AARLGLQQD--SKPFSKKRKWGG--AVAPEPQH------------------------------
Xenopus (ENSXETP00000054061)     ALWEAKMKQLAERARAREEE-------AAKLGIKQE--VKSLGKKRKWGG--AATTETPP------------------------------
Coelacanth (ENSLACP00000009535   ALWEAKMRLLAERARARQEE-------AERLGLQPD--IKPFGKKRKWGG--APAAEPEH------------------------------
Spotted_gar (ENSLOCP0000001981   ALWEAKMKLLAERARQRQEE-------AARLGLPQED-IKAYGKKRKWGAAGSNSSAASGN-----------------------------
Amazon_molly (ENSPFOP000000021   ALWEAKMKLLAERALQRQQE-------AALLGLSQED-IKALGKKRKWGSLLTGASPGPG------------------------------
Cave_fish (ENSAMXP00000015689)   ALWEAKMKLLAERARQRQQE-------AALLGLSQEE-IKAYAKKRKWADGAS--SPSSG------------------------------
Fugu (ENSTRUP00000036865)        GLWEAKMKLLAERALQRQQE-------AALLGLSQED-IKALGKKRKWGAALAGASPGPG------------------------------
Medaka (ENSORLP00000019236)      ALWEAKM-----------------------------------------------------------------------------------
Platyfish (ENSXMAT00000000130)   ALWEAKMKLLAERALQRQQE-------AALLGLSQED-IKALGKKRKWGSLLTGASPGPG------------------------------
Stickleback (ENSGACP0000001732   AVWEAKMKLLAERALERQQE-------AALLGLSQEE-IKALGKKRKWGATAAGASPGPG------------------------------
Tetraodon (ENSTNIP00000019883)   GLWEAKMKLLAERALQRQQE-------AALLGLSQED-IKALGKKRKWGAAAAGASPGPG------------------------------
Tilapia (ENSONIP00000019943)     ALWEAKMKLLAERALQRQQE-------AALLGLSQED-IKTLGKKRKWGATAAGASPGPG------------------------------
Zebrafish (ENSDARP00000085229)   ALWESKMKILAERARQRQQE-------AALLGLSQEE-IKAYGKKRKWAAGSA--SPSPG------------------------------
Rainbow_trout (XP_021462005.1)   WIWEAKMKMLAERARERQQE-------AALLGLPYDD-IKP-GKKRKLGATAAGASPGPG------------------------------
Rainbow_trout (XP_021477599.1)   WLWEAKMKMLAERARERQQE-------AALLGLPYED-IKP-GKKRKLGATAGGASPGPG------------------------------
Salmon (XP_014026412.1)          WIWEAKMKMLAERARERQQE-------AALLGLPYDD-IKP-GKKRKLGATAAGASPGPG------------------------------
Salmon (XP_014017100.1)          WLWEAKMKMLAERARERQQE-------AALLGLPYED-IKP-GKKRKLGATAGGASPGPG------------------------------

                                         3160      3170      3180      3190      3200
                                 ....|....|....|....|....|....|....|....|....|....|....|....
```

```
European ampioxus (Sc0000095)       -----------------------------------------------------------
Ciona intestinalis (XP_0021254      PVSRTFPIEGELPRVMTSPRNMPRLMTSPTNQAPHPTGGIPTSSPYPIPQYCHPTPQRT
Lamprey (scaf_00044)                -----------------------------------------------------------
Lamprey (scaf_03335)                -----------------------------------------------------------
Shark (XP_007897316.1)              -------------------EDNELVRVPTRKALSTPRDSVITVSSYALTQVTGPYSRWI
Lizard (XP_020663651.1)             -------------------EDNEFNQIPSRRALTVTHDNIITVSTYALTRVAGPYNHWA
Chicken (ENSGALP00000053457)        -------------------EDNEFNQIPSRRALTVTKDNVITVSSYALTRVAGPYNHWA
Turtle (ENSPSIP00000006704)         -------------------EENEFNQIPSRRALTVTQDNVITVSSYALTRVAGPYNHWA
Human (ENSP00000362748)             -------------------EVNELNQIPSHKALTLTHDNVVTVSPYALTHVAGPYNHWV
Gorilla (ENSGGOP00000011536)        -------------------EVNELNQIPSHKALTLTHDNVVTVSPYALTHVAGPYNHWV
Mouse (ENSMUSP00000133279)          -------------------EANLSHQIPSRVASTLTRDNVVTVSPYSLTHVAGPYNRWV
Rat (ENSRNOP00000054747)            -------------------ETNLSYQIPSRVASTLTSDNVVTVSPYALTHVAGPYNRWV
Pig (ENSSSCP00000010918)            -------------------EINEFSQIPSHKALTLTHDNVVTVSPYALTHVAGPYNHWV
Whale (XP_023987950.1)              -------------------EVNEFNQIPSHKALTLTHDNVVTVSPYALTHVAGPYNHWV
Elephant (ENSLAFP00000005179)       -------------------EVNELNQIPSHKALTLTHDNVVTVSPYALTHVAGPYNRWV
Armadillo (ENSDNOP00000014565)      -------------------AVNELNQIPSHKALTLTHDNVVTVSPYALTHVAGPYNHWV
Tasmanian_devil (XP_023360087.      -------------------EQNELNQIPSRKALTVTHDNVITVSSYALTQVAGPYNHWV
Coelacanth (ENSLACP00000016889      -------------------KDNELIKIPTRKSLTATRDRVITVSSYALTQVTGPYNRWV
Cavefish(XP_007240334.2)            -------------------DEREKLQVPTRQSLTATRDGLVTSAPYSLTRVTGPYNRWM
Platyfish (XP_014325204.1)          -------------------ETRRVMNVPTRQAFTAPTGGAITVSPYALTHVTGPYNRWS
Tilapia (XP_005456583.2)            -------------------EMR-IMNVPTRKAWTLPRDEVITASPYALTQVTGPYNRWS
Spotted_gar (XP_015202470.1)        -------------------EERELLQIPTRRALAITRDGVITVSSYALTQVTGPYNRWV
Stickleback (ENSGACT0000001035      --------------------TRRIMNVPTRQARTLQRDGVVTASPYALTQVTGPYNRWA
Tetraodon (ENSTNIT00000011701)      -------------------DE-RTTHVPTRQAWTFPRDGVITVSPYALTQVTGPYNRWT
Fugu (XP_011601624.1)               -------------------EEERTMHVPTRQAWTFPRDGVITVSPYALTQVTGPYNRWT
Zebrafish (ENSDARP00000143362)      -------------------DERHKFQVPTRQSLTVSRDSLVMSAPYALTHVTGPYNRWT
Amazon_molly (XP_016534180.1)       -------------------EARRVMNVPTRQAFTAPTGGVITVSPYALTHVTGPYNRWS
Medaka (XP_023818916.1)             -------------------ATRRIMNVPTRQAWTLPRDGVITVSPYALTQVTGPYNRWT
Rainbow_trout (XP_021469805.1)      -------------------EKKGVLKVPTRQAVTVARDGVVTVSPYALTQVTGPYNRWT
Rainbow trout (XP_021436521.1)      -------------------EKKGVLTVPTRKAVTVPRDGVVTVSPYALTQVTGPYNRWT
Salmon (XP_014010485)               -----------------------------------------------------------
Salmon (XP_014070638.1)             -------------------EKKGVLTVPTRKAVTVPRDGVVTVSPYALTQVTGPYNRWT
Shark (XP_007890120.1)              -------------------QLRFIKTLTQRTMT-FTTNSVTTASPYAFTRVTGPYNRWI
Human (ENSP00000425443)             -------------------YLRFIKSLAERTMS-VTTDSTVTTSPYAFTRVTGPYNRYI
Gorilla (ENSGGOP00000034525)        -------------------YLRFIKSLAERTMS-VTTDSTVTTSPYAFTRVTGPYNRYI
Mouse (ENSMUSP00000143029)          -------------------YLRFIQSLAENTGS-VTTDSTVTTSPYAFTQVTGPYNTFV
Rat (ENSRNOP00000048956)            -------------------YLRFIHSLAENTVS-MTTDSTVTTRPYAFTQVTGPYNTYV
Pig (ENSSSCP00000009765)            -------------------YLRFIRSLAERTMS-VTTDSTVTTSPYAFTRVTGPYNRYV
Tasmanian_devil (ENSSHAT000000      -------------------YLRFIKSLTQRTMS-ITTNSTVTTSPYAFTRVTGPYNRYV
Whale (XP_007106564.2)              -------------------YLRFIKSLAERTMS-VTTDSTVTTSPYAFTRVTGPYNRYI
Elephant (XP_023404313.1)           -------------------YLRFIKSLTERTMS-VTTDSTVTTSPYAFTRVTGPYNRYI
Lizard (ENSACAP00000008438)         -------------------YMRFIRSLAQRTMS-LTTNSTVTTSPYAFTRVTGPYNRYI
Chicken (ENSGALP00000017216)        -------------------YLRFIKSLAQRTLS-VTTDSTVTTSPYAFTRVTGPYNRYI
Turtle (ENSPSIP00000011691)         -------------------YMRFVKSLAQRTMS-VTTNSTVTTSPYAFTRVTGPYNRYI
Xenopus (ENSXETP00000030770)        -------------------YLRFIKSLTQRTMS-ITTDSQVTTSPYALTRVTGPYNRYI
Coelacanth (ENSLACP00000008576      -------------------YLRFVKSLAQRTMS-ITTNTSVTTSPYAFTRVTGPYNRYI
Amazon_molly (ENSPFOP000000120      -------------------YKRFIRALMEGSSSSFTTNTHVMTTPYAFTKVTGPYSQFV
Amazon_molly (ENSPFOP000000299      -------------------YKRFIRALMEGSSSSFTTNTHVMTTPYAFTKVTGPYSQFV
Fugu (XP_011611191.1)               -------------------YKRFIKALAEGSAS-CTTNTWVNTAPYAFTKVTGPYSHFV
Medaka (XP_004065962.1)             -------------------YKRFIKALMEGSLS-STTNTYVNTSPYAFTKVTGPYSRFA
Platyfish (ENSXMAP00000005447)      -------------------YKRFIKALMEGSSSSFTTNTHVITTPYAFTKVTGPYSQFV
Spotted _gar (ENSLOCP000000155      -------------------YKRFIQTLTQRTVS-CTTNSIVNTSPYAFTKVTGPYNRYI
Stickleback (ENSGACP0000002318      -------------------YKRFIQALMEGSSS-CTTNSHVNTTPYAFTKVTGPYSQFA
Tilapia (XP_005457058.1)            -------------------YKRYIKALMEGSLS-CTTNTYVSTSPYAFTKVTGPYSQLA
Tetraodon (ENSTNIP00000021201)      -------------------YKRFIKALAEGSAS-CTTNTWVNTAPYAFTKVTGPYSHFV
Zebrafish (ENSDARP00000101295)      -------------------YKQFLLMLTERSMS-CTTNTYVSTSPYAFTKVTGPYNNFM
Salmon (XP_014042431.1)             -------------------YKRFIQTLMERSMS-CTTNTYVNTSPYAFTKVTGPYSRFI
Salmon (XP_014065146.1)             -------------------YKRFIQTLMERSMS-CTTNTYVNTSPYAFTKVTGPYSRFI
Salmon (XP_014050290.1)             -------------------YKRFIQTLMERSMS-CTTNTYVNTSPYAFTKVTGPYSRFI
Rainbow trout (XP_021429026.1)      -------------------YKRFIQTLMERSMS-CTTNTYVNTSPYAFTKVTGPYSRFI
Rainbow trout (XP_021429023.1)      -------------------YKRFIQTLMERSMS-CTTNTYVNTSPYAFTKVTGPYSRFI
Shark (XP_007910136.1)              ----------REWLRKILLQKNKPPWSSTGTEEKRLTNTWQESKALLFQPTPGAQGVKS
Human (ENSP00000386869)             -------------------KE-KKGVVPTRQALAVPTDSAVTVSSYAYTKVTGPYSRWI
Gorilla (ENSGGOP00000051172)        -------------------KE-KKGVVPTRQALAVPTDSAVTVSSYAYTKVTGPYSRWI
Armadillo (ENSDNOP00000021516)      -------------------KE-RRGAVPTRQALAVPTDSAVTVSSYAYTKVTGPYSRWI
Mouse (ENSMUSP00000087049)          -------------------KE-KKGAIPTRQALAMPTDSAVTVSSYAYTKVTGPYSRWI
Rat (ENSRNOP00000015296)Tet3        -------------------KE-KKGAVPTRQALAMPTDSAVTVSSYAYTKVTGPYSRWI
Pig (ENSSSCP00000008852)            -------------------KE-KKGLVPTRQALAMPTDSAVTVSSYAYTKVTGPYSRWI
Elephant  (XP_010591957.2)          -------------------KE-KKGAVPTRQALAMPTDSAVTVSSYAYTKVTGPYSRWI
Whale (XP_023989264.1)              -------------------KE-KKGLVPTRQALAVPTDSAVTVSSYAYTKVTGPYSRWI
Tasmanian_devil (ENSSHAP000000      -------------------EE-QKSPIPTR-----------------HTKVTGPYSRWI
Chicken (ENSGALT00000059756)        -------------------KE-RRNAVPTRQAVAIPTNSAITVSSYAYTKVTGPYSRWV
Lizard (ENSACAT00000001585)         -------------------KE-RRGAVPTREALTVPTHSAITAASYAFTKVTGPYSRWI
Turtle (ENSPSIT00000006549)         -------------------KE-KKDSVPTRQAVAIPTNSAITVSSYAYTKTN--CWEWL
Xenopus (ENSXETP00000054061)        -------------------VE-KKDYTPTRQAATILTDSATTSFSYAYTKVTGPYSRFI
Coelacanth (ENSLACP00000009535      -------------------KE-KKDIIPIRQALALTTNSVITVSSYAYTKVTGPYSHWI
Spotted_gar (ENSLOCP0000001981      ----------S-ASGQSQSKD-KKEGIHTRHAPTPHTNSLVTVSPYAHTQLTGPYSRWV
Amazon_molly (ENSPFOP000000021      -----------------QLKD-KKDGPVTRLSHTFYTTSMVTVSPYAFTRVTGPYSHFV
Cave_fish (ENSAMXP00000015689)      -----------------PTKD-RREGVVTRMAPTQHTTTMVTLSPYAFTQLTGPYSRFM
Fugu (ENSTRUP00000036865)           -----------------QSKD-KREGPVTRLAPTVHTTSVVTVSPYAFTRLTGPYSHFV
Medaka (ENSORLP00000019236)         -----------------------------------------------------------
Platyfish (ENSXMAT00000000130)      -----------------QSKD-KKDGPVTRLSHTFYTTSMVTVSPYAFTRVTGPYSHF-
Stickleback (ENSGACP0000001732      -----------------QSKD-KREGPVTRLAPTLHTSTTVTASPYAFTRLTGPYSHFV
Tetraodon (ENSTNIP00000019883)      -----------------QSKD-KREGPVTRLAPTAHTTSVVTASPYAFTRLTGPYSHFV
Tilapia (ENSONIP00000019943)        -----------------QSKD-KKEGPVTRLAPTFYTTSTVTVSPYASTVVTGPYSHFV
Zebrafish (ENSDARP00000085229)      -----------------QTKD-KREGVVTRMAPTQHSTTIVTVSPYPSTHLTGPYSRFV
Rainbow_trout (XP_021462005.1)      -----------------QTTD-KREGPVTRLASTRHTTSTITVSPYAFTTLTGPYSHFV
Rainbow_trout (XP_021477599.1)      -----------------QTTEGPVMGMVTRLASTHHTTSTVTVSPYAFTTLTGPYSHFV
```

```
Salmon (XP_014026412.1)         -----------------QTTD-KREGPVTRLAPTRHTTSTITVSPYAFTTLTGPYSHFV
Salmon (XP_014017100.1)         -----------------QTTEGPVMGTVTRLAPTHHTTSTVTVSPYAFTTLTGPYSHFV
```

Supplemental Fig.S7 Tet protein sequences alignment. All the protein sequences were aligned with the MUSCLE method in MEGA software. Conserved domains were noted above. # Zn binding sites; red box, Fe binding sides; yellow box, 2-OG binding sites; blue box, 5mC binding sites.

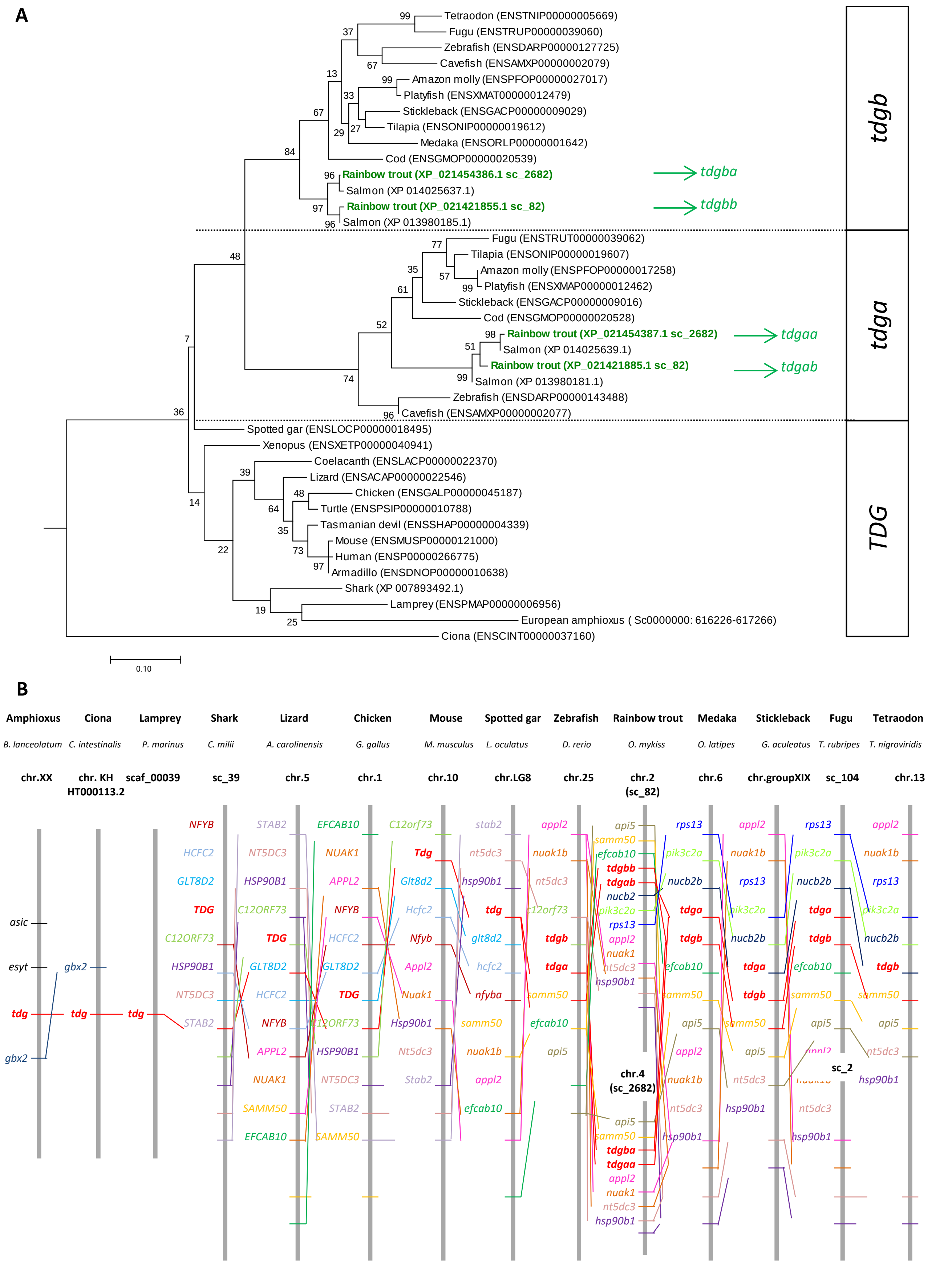

Supplementary Fig. S8 ***In silico*** **analysis of tdg in chrodates.** (A) Pylogenetic of tdg genes. The phylogenetic trees were built using the Maximum Likelihood method based on the WAG model in MEGA7. The tree with the highest log likelihood (-5287.94) is shown. A discrete Gamma distribution was used to model evolutionary rate differences among sites (5 categories (+G, parameter = 1.1866)). The reliability of the inferred trees was estimated by the bootstrap method with 500 replications. All accession numbers are specified in parentheses. (B) Syntenic analysis of dnmt1 genes. Conserved synteny around the tdgloci in chrodates. Data were collected with Genomicus software version 01.01, and tdg genes in rainbow trout were annotated by ourselves according to our phylogenetic analysis following ZFIN nomenclature guidelines. chr., chromosome; sc., scaffold.

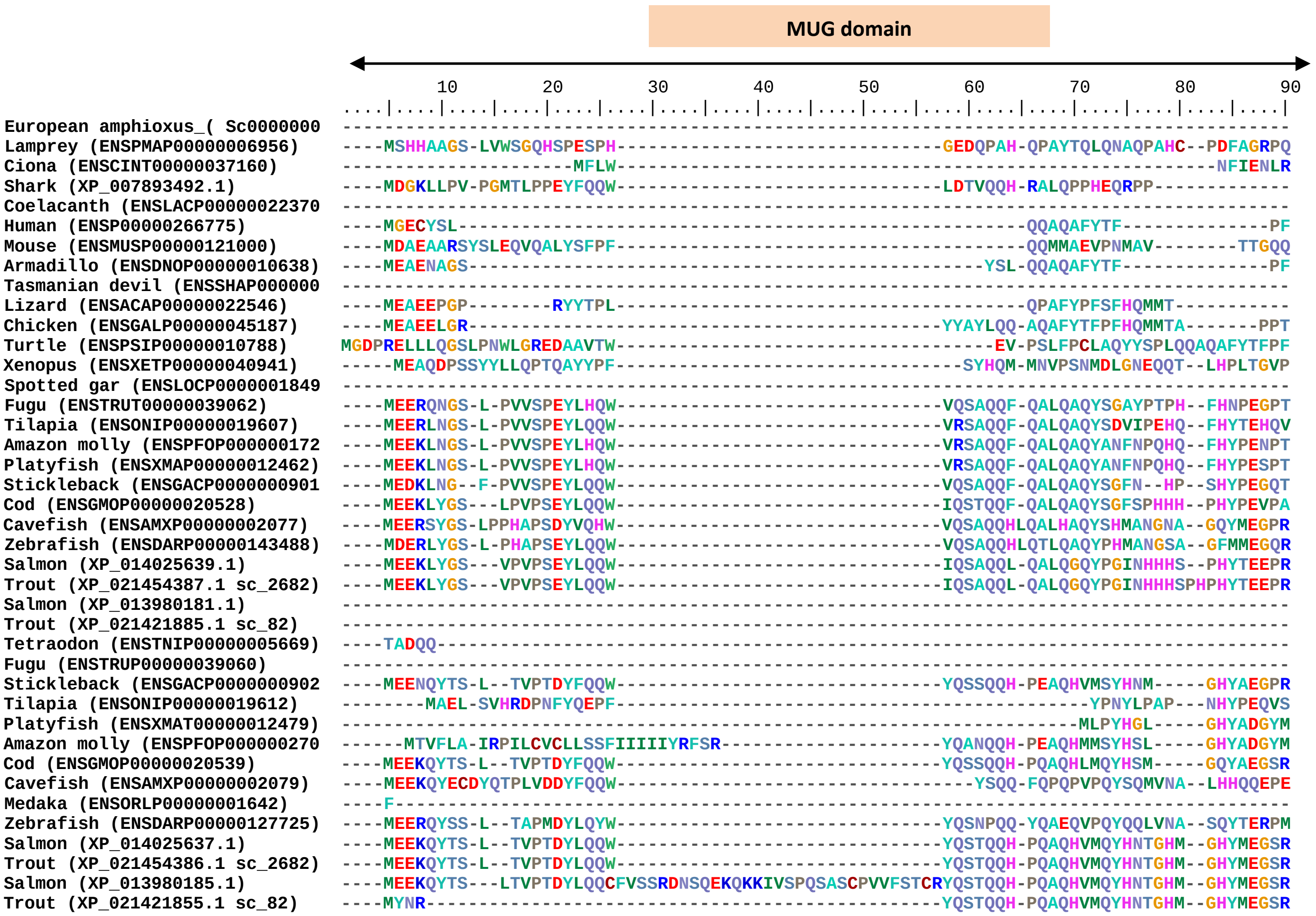
MUG domain
10
20
30
40
50
60
70
80
90
European amphioxus_(Sc0000000
Lamprey (ENSPMAP00000006956)
Ciona (ENSCINT00000037160)
Shark (XP_007893492.1)
Coelacanth (ENSLACP00000022370
Human (ENSP00000266775)
Mouse (ENSMUSP00000121000)
Armadillo (ENSDNOP00000010638)
Tasmanian devil (ENSSHAP000000
Lizard (ENSACAP00000022546)
Chicken (ENSGALP00000045187)
Turtle (ENSPSIP00000010788)
Xenopus (ENSXETP00000040941)
Spotted gar (ENSLOCP0000001849
Fugu (ENSTRUT00000039062)
Tilapia (ENSONIP00000019607)
Amazon molly (ENSPFOP000000172
Platyfish (ENSXMAP00000012462)
Stickleback (ENSGACP0000000901
Cod (ENSGMOP00000020528)
Cavefish (ENSAMXP00000002077)
Zebrafish (ENSDARP00000143488)
Salmon (XP_014025639.1)
Trout (XP_021454387.1 sc_2682)
Salmon (XP_013980181.1)
Trout (XP_021421885.1 sc_82)
Tetraodon (ENSTNIP00000005669)
Fugu (ENSTRUP00000039060)
Stickleback (ENSGACP0000000902
Tilapia (ENSONIP00000019612)
Platyfish (ENSXMAT00000012479)
Amazon molly (ENSPFOP000000270
Cod (ENSGMOP00000020539)
Cavefish (ENSAMXP00000002079)
Medaka (ENSORLP00000001642)
Zebrafish (ENSDARP00000127725)
Salmon (XP_014025637.1)
Trout (XP_021454386.1 sc_2682)
Salmon (XP_013980185.1)
Trout (XP_021421855.1 sc_82)

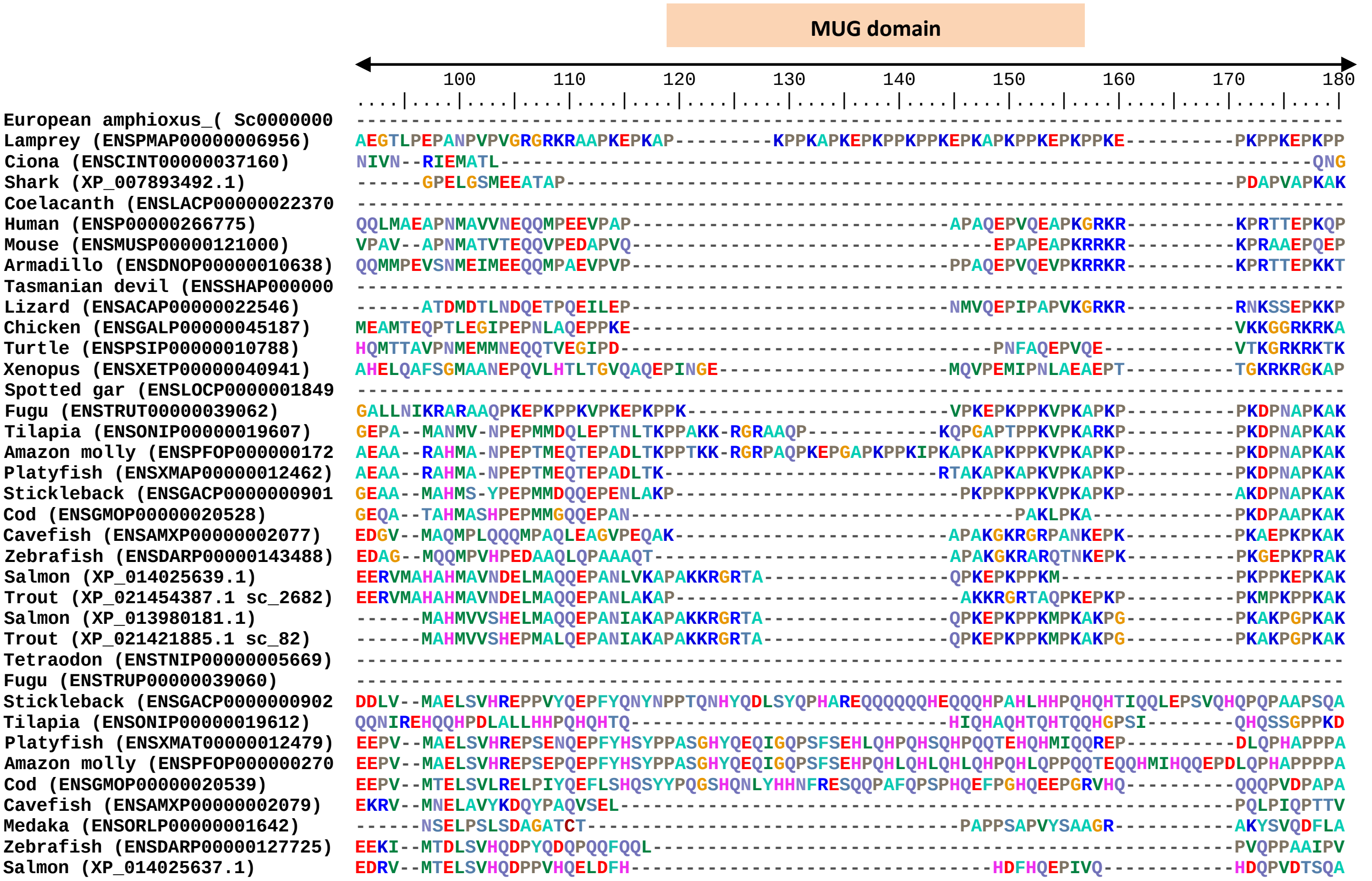
MUG domain
100
110
120
130
140
150
160
170
180
European amphioxus_(Sc0000000
Lamprey (ENSPMAP00000006956)
Ciona (ENSCINT00000037160)
Shark (XP_007893492.1)
Coelacanth (ENSLACP00000022370
Human (ENSP00000266775)
Mouse (ENSMUSP00000121000)
Armadillo (ENSDNOP00000010638)
Tasmanian devil (ENSSHAP000000
Lizard (ENSACAP00000022546)
Chicken (ENSGALP00000045187)
Turtle (ENSPSIP00000010788)
Xenopus (ENSXETP00000040941)
Spotted gar (ENSLOCP0000001849
Fugu (ENSTRUT00000039062)
Tilapia (ENSONIP00000019607)
Amazon molly (ENSPFOP000000172
Platyfish (ENSXMAP00000012462)
Stickleback (ENSGACP0000000901
Cod (ENSGMOP00000020528)
Cavefish (ENSAMXP00000002077)
Zebrafish (ENSDARP00000143488)
Salmon (XP_014025639.1)
Trout (XP_021454387.1 sc_2682)
Salmon (XP_013980181.1)
Trout (XP_021421885.1 sc_82)
Tetraodon (ENSTNIP00000005669)
Fugu (ENSTRUP00000039060)
Stickleback (ENSGACP0000000902
Tilapia (ENSONIP00000019612)
Platyfish (ENSXMAT00000012479)
Amazon molly (ENSPFOP000000270
Cod (ENSGMOP00000020539)
Cavefish (ENSAMXP00000002079)
Medaka (ENSORLP00000001642)
Zebrafish (ENSDARP00000127725)
Salmon (XP_014025637.1)

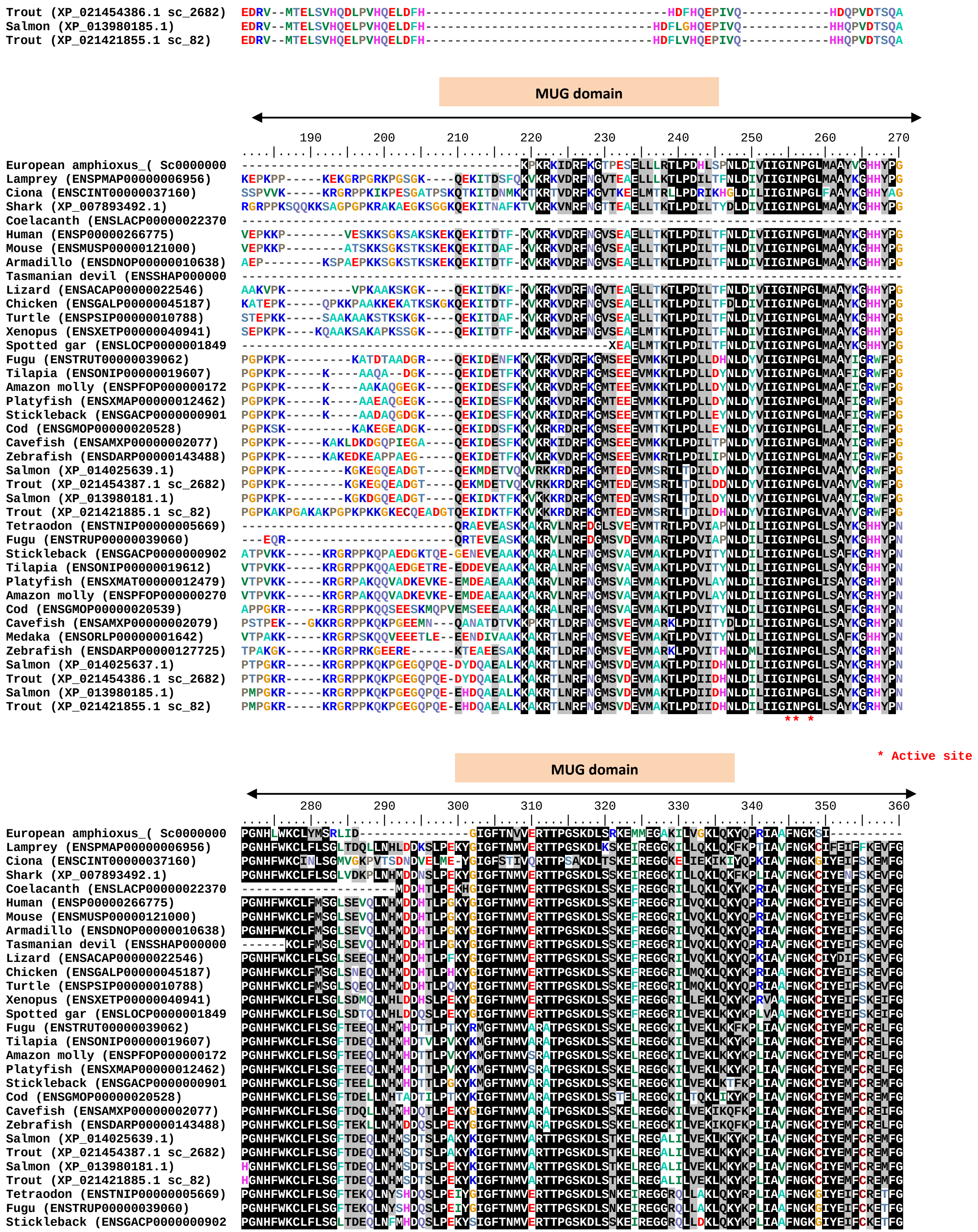

Trout (XP_021454386.1 sc_2682)
Salmon (XP_013980185.1)
Trout (XP_021421855.1 sc_82)
MUG domain
190
200
210
220
230
240
250
260
270
European amphioxus_(Sc0000000
Lamprey (ENSPMAP00000006956)
Ciona (ENSCINT00000037160)
Shark (XP_007893492.1)
Coelacanth (ENSLACP00000022370
Human (ENSP00000266775)
Mouse (ENSMUSP00000121000)
Armadillo (ENSDNOP00000010638)
Tasmanian devil (ENSSHAP000000
Lizard (ENSACAP00000022546)
Chicken (ENSGALP00000045187)
Turtle (ENSPSIP00000010788)
Xenopus (ENSXETP00000040941)
Spotted gar (ENSLOCP0000001849
Fugu (ENSTRUT00000039062)
Tilapia (ENSONIP00000019607)
Amazon molly (ENSPFOP000000172
Platyfish (ENSXMAP00000012462)
Stickleback (ENSGACP0000000901
Cod (ENSGMOP00000020528)
Cavefish (ENSAMXP00000002077)
Zebrafish (ENSDARP00000143488)
Salmon (XP_014025639.1)
Trout (XP_021454387.1 sc_2682)
Salmon (XP_013980181.1)
Trout (XP_021421885.1 sc_82)
Tetraodon (ENSTNIP00000005669)
Fugu (ENSTRUP00000039060)
Stickleback (ENSGACP0000000902
Tilapia (ENSONIP00000019612)
Platyfish (ENSXMAT00000012479)
Amazon molly (ENSPFOP000000270
Cod (ENSGMOP00000020539)
Cavefish (ENSAMXP00000002079)
Medaka (ENSORLP00000001642)
Zebrafish (ENSDARP00000127725)
Salmon (XP_014025637.1)
Trout (XP_021454386.1 sc_2682)
Salmon (XP_013980185.1)
Trout (XP_021421855.1 sc_82)
* Active site
MUG domain
280
290
300
310
320
330
340
350
360

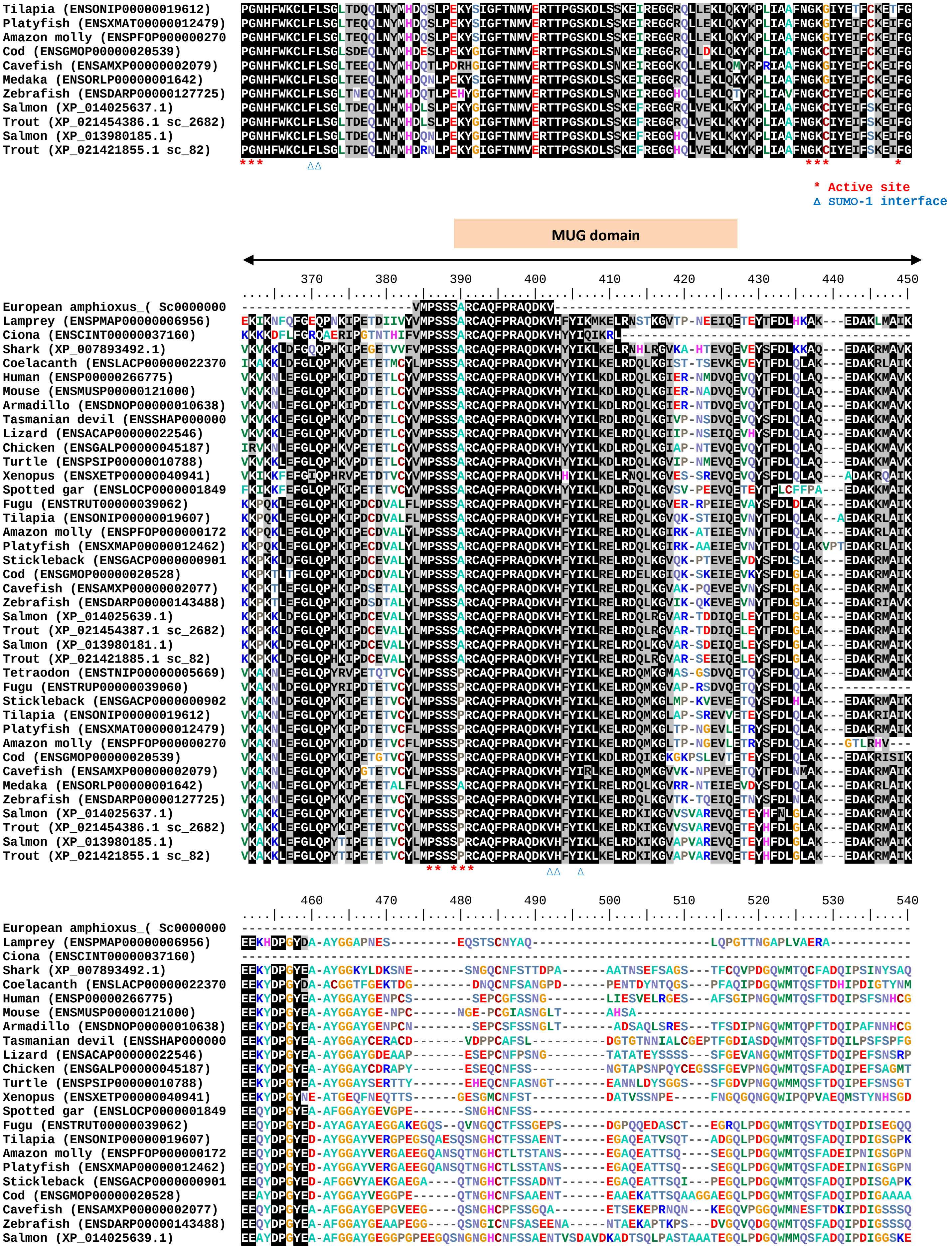
Tilapia (ENSONIP00000019612)
Platyfish (ENSXMAT00000012479)
Amazon molly (ENSPFOP000000270
Cod (ENSGMOP00000020539)
Cavefish (ENSAMXP00000002079)
Medaka (ENSORLP00000001642)
Zebrafish (ENSDARP00000127725)
Salmon (XP_014025637.1)
Trout (XP_021454386.1 sc_2682)
Salmon (XP_013980185.1)
Trout (XP_021421855.1 sc_82)
* Active site
Δ SUMO-1 interface
MUG domain
370 380 390 400 410 420 430 440 450
European amphioxus_(Sc0000000
Lamprey (ENSPMAP00000006956)
Ciona (ENSCINT00000037160)
Shark (XP_007893492.1)
Coelacanth (ENSLACP00000022370
Human (ENSP00000266775)
Mouse (ENSMUSP00000121000)
Armadillo (ENSDNOP00000010638)
Tasmanian devil (ENSSHAP000000
Lizard (ENSACAP00000022546)
Chicken (ENSGALP00000045187)
Turtle (ENSPSIP00000010788)
Xenopus (ENSXETP00000040941)
Spotted gar (ENSLOCP0000001849
Fugu (ENSTRUT00000039062)
Tilapia (ENSONIP00000019607)
Amazon molly (ENSPFOP000000172
Platyfish (ENSXMAP00000012462)
Stickleback (ENSGACP0000000901
Cod (ENSGMOP00000020528)
Cavefish (ENSAMXP00000002077)
Zebrafish (ENSDARP00000143488)
Salmon (XP_014025639.1)
Trout (XP_021454387.1 sc_2682)
Salmon (XP_013980181.1)
Trout (XP_021421885.1 sc_82)
Tetraodon (ENSTNIP00000005669)
Fugu (ENSTRUP00000039060)
Stickleback (ENSGACP0000000902
Tilapia (ENSONIP00000019612)
Platyfish (ENSXMAT00000012479)
Amazon molly (ENSPFOP000000270
Cod (ENSGMOP00000020539)
Cavefish (ENSAMXP00000002079)
Medaka (ENSORLP00000001642)
Zebrafish (ENSDARP00000127725)
Salmon (XP_014025637.1)
Trout (XP_021454386.1 sc_2682)
Salmon (XP_013980185.1)
Trout (XP_021421855.1 sc_82)
460 470 480 490 500 510 520 530 540
European amphioxus_(Sc0000000
Lamprey (ENSPMAP00000006956)
Ciona (ENSCINT00000037160)
Shark (XP_007893492.1)
Coelacanth (ENSLACP00000022370
Human (ENSP00000266775)
Mouse (ENSMUSP00000121000)
Armadillo (ENSDNOP00000010638)
Tasmanian devil (ENSSHAP000000
Lizard (ENSACAP00000022546)
Chicken (ENSGALP00000045187)
Turtle (ENSPSIP00000010788)
Xenopus (ENSXETP00000040941)
Spotted gar (ENSLOCP0000001849
Fugu (ENSTRUT00000039062)
Tilapia (ENSONIP00000019607)
Amazon molly (ENSPFOP000000172
Platyfish (ENSXMAP00000012462)
Stickleback (ENSGACP0000000901
Cod (ENSGMOP00000020528)
Cavefish (ENSAMXP00000002077)
Zebrafish (ENSDARP00000143488)
Salmon (XP_014025639.1)

```
Trout (XP_021454387.1 sc_2682)  EEAYDPGYEA-AFGGAYGEGGPGPEEGQSNGNGHCNFSSAENTVSDAVEKADTSQLPAATAAATEGQLPDGQWMMQSFADQIPDIGGSKE
Salmon (XP_013980181.1)         EEAYDPGYEA-AFGGAYGEGGPG-----------------------------------------PEEGQSNGH------------------
Trout (XP_021421885.1 sc_82)    EEAYDPGYEA-ALGGAYGEGGPG-----------------------------------------PEEGQSNGH------------------
Tetraodon (ENSTNIP00000005669)  EEQYDPGYED-EGGAREGQS----------QVNGHCTFSS----------DAPREKDASPA---AEGGVQDGRWMTQSFADQIPDIS----
Fugu (ENSTRUP00000039060)       --GKQPSPSPVPVGLTFSRL-------------QCCF--------------------------CLQKMARG-WPSKRSRKILSMRAAAGS
Stickleback (ENSGACP0000000902  EEQVDPEYES-CAEVRGDAR----------QSSNSTN------------------------------------------------------
Tilapia (ENSONIP00000019612)    EEQLDPEYES-CSALHEETR----------HSNNSSN------------------------------------------------------
Platyfish (ENSXMAT00000012479)  EEQVDPEYES-CSGLHDGVRQSS--------------------------------------------------------------------
Amazon molly (ENSPFOP000000270  -----FGHRKKCSVLQFIY-----------------------------------------------------FLINS-------------
Cod (ENSGMOP00000020539)        EEQVDPEYEN-CDRGREEAR----------QGPSHCSI-----------------------------------------------------
Cavefish (ENSAMXP00000002079)   EEQPDPGYEESSTSGSQQA---------------CSFYPANER------DAANSEHSS-----LMDQVRSSQWTVQPFEDQIPDIRCPNT
Medaka (ENSORLP00000001642)     EEQYDPGYED-AYGGAYVEKTAEEGQAEGQSNGHCSFSSAEN---------------------KEGQLPDGQWMTQSFADHIPDIS----
Zebrafish (ENSDARP00000127725)  EEQNDPGYEETCAHGSWGEP-------------QQAFGVHPST--------------------NTDQVMDDRWTMQPFAEQIPDIRCSNN
Salmon (XP_014025637.1)         EEQVDPEYET-CDGEREEAR----------QSTSYCNISSTKET------AMPSKDQAQAT---PLNHMPYDQWMTQSFADQIPDISCNSS
Trout (XP_021454386.1 sc_2682)  EEQVDPEYET-CDGEREEAR----------QSTSYCNISSTKET------AMPSKDQAQAT---PLNHMPYDQWMTQSFADQIPDISCNSS
Salmon (XP_013980185.1)         EEQVDPEYEN-CGGARQDAK----------QSTSYCNISSTKET------AMPSKDQAT-----AMSHMPYDQWMTQSFADQIPDISCNSS
Trout (XP_021421855.1 sc_82)    EEQVDPEYEN-CGGASQDAK----------QSTSYCNISSTKET------GMPSKDQAT-----AMSHMPYDQWMTQSFADQIPDISCNSS

                                        550       560       570
                                ....|....|....|....|....|....|....|..
European amphioxus_( Sc0000000  -------------------------------------
Lamprey (ENSPMAP00000006956)    -------------------------------------
Ciona (ENSCINT00000037160)      -------------------------------------
Shark (XP_007893492.1)          PQAENST------------------------------
Coelacanth (ENSLACP00000022370  QQQG---------------------------------
Human (ENSP00000266775)         TQEQEEESHA---------------------------
Mouse (ENSMUSP00000121000)      -------------------------------------
Armadillo (ENSDNOP00000010638)  TPEQEEGSHA---------------------------
Tasmanian devil (ENSSHAP000000  TLEQE--------------------------------
Lizard (ENSACAP00000022546)     QEEGNDA------------------------------
Chicken (ENSGALP00000045187)    QEREGSSA-----------------------------
Turtle (ENSPSIP00000010788)     QEQEESSV-----------------------------
Xenopus (ENSXETP00000040941)    QQQGGSNA-----------------------------
Spotted gar (ENSLOCP0000001849  -------------------------------------
Fugu (ENSTRUT00000039062)       DGGV---------------------------------
Tilapia (ENSONIP00000019607)    DGSI---------------------------------
Amazon molly (ENSPFOP000000172  ASST---------------------------------
Platyfish (ENSXMAP00000012462)  ASSVLSLSSTSLEGRIEPGLLLHSRCYELIRCTVTTR
Stickleback (ENSGACP0000000901  EVFEWNI------------------------------
Cod (ENSGMOP00000020528)        AVTQI--------------------------------
Cavefish (ENSAMXP00000002077)   APAGAV-------------------------------
Zebrafish (ENSDARP00000143488)  AQNWGV-------------------------------
Salmon (XP_014025639.1)         PQAWAGNV-----------------------------
Trout (XP_021454387.1 sc_2682)  PQAWAGNV-----------------------------
Salmon (XP_013980181.1)         -------------------------------------
Trout (XP_021421885.1 sc_82)    -------------------------------------
Tetraodon (ENSTNIP00000005669)  -------------------------------------
Fugu (ENSTRUP00000039060)       SASPPPE------------------------------
Stickleback (ENSGACP0000000902  -------------------------------------
Tilapia (ENSONIP00000019612)    -------------------------------------
Platyfish (ENSXMAT00000012479)  -------------------------------------
Amazon molly (ENSPFOP000000270  -------------------------------------
Cod (ENSGMOP00000020539)        -------------------------------------
Cavefish (ENSAMXP00000002079)   NRIS---------------------------------
Medaka (ENSORLP00000001642)     -------------------------------------
Zebrafish (ENSDARP00000127725)  TRTS---------------------------------
Salmon (XP_014025637.1)         NVAQPQLGAETF-------------------------
Trout (XP_021454386.1 sc_2682)  NVAQPHLGAETF-------------------------
Salmon (XP_013980185.1)         NVPQPQLGAETF-------------------------
Trout (XP_021421855.1 sc_82)    NVPQPQLGAETF-------------------------
```

Supplemental Fig.S9 Tdg protein sequences alignment. All the protein sequences were aligned with the MUSCLE method in MEGA software. Conserved domains were noted above. Active sites were marked with an asterisk (*); SUMO-1 interface (Δ **)**.

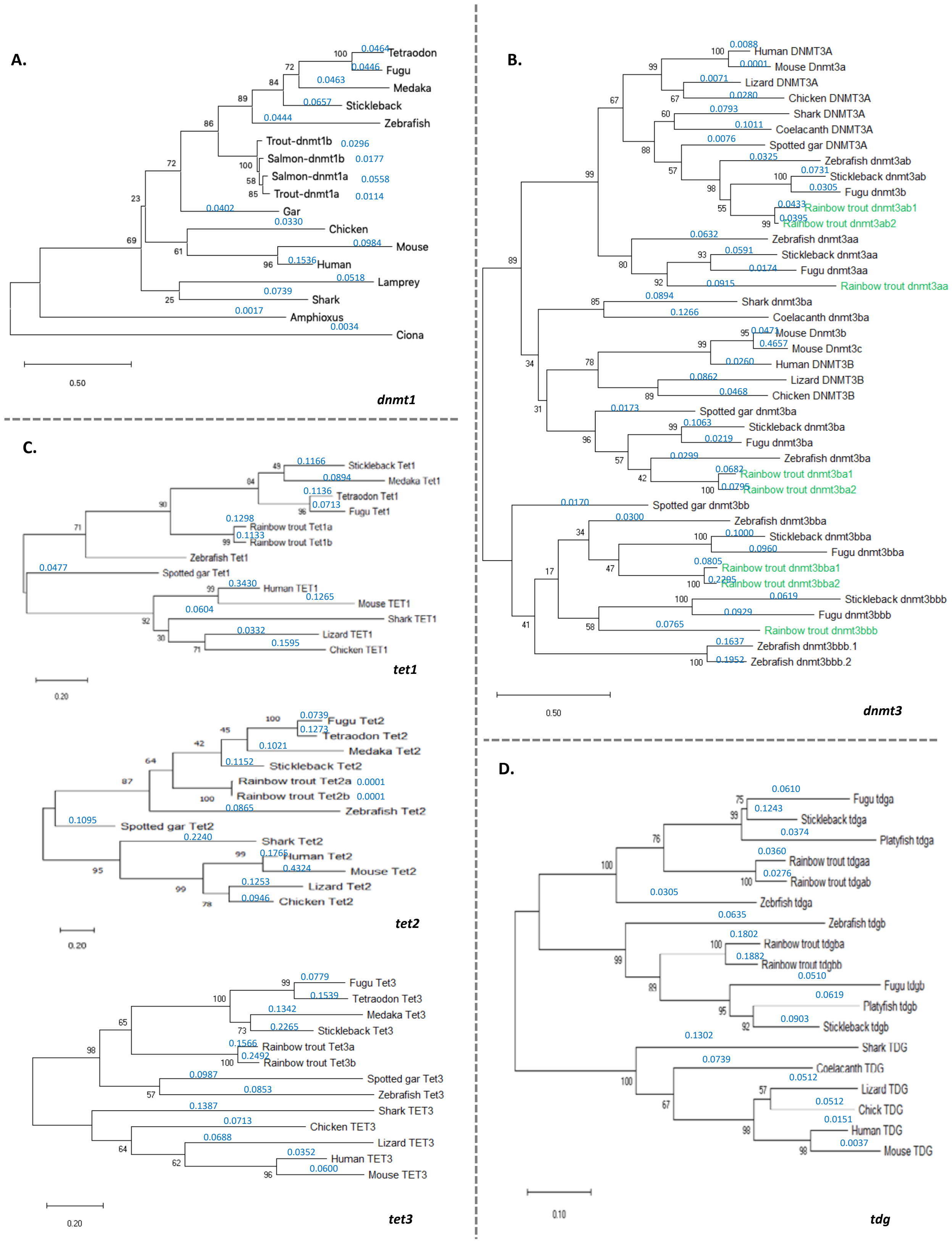

Supplemental Fig. S10 Branch test of dN/ds ratio for dnmt, tet and tdg genes. The ratios between non-synonymous and synonymous differences (dN/dS) were also calculated with the codeml program in the PAML package version 4.9i. The aligned amino acids sequences of *dnmt1*, *dnmt3*, *tet1*, *tet2*, *tet3* and *tdg* genes in selected representative species were used to guide the alignment of the nucleotides sequences by PAL2NAL to get codon alignments of these genes. We further built phylogenetic trees with their respective codon alignment of these genes using Maximum likelihood method. The codon alignments and phylogenetic trees were used as the input file for codeml program. Branch tests with the free-ratios model (Alternative hypothesis: heterogeneity of dN/dS among branches) and model 0 (Null hypothesis: homogeneous dN/dS across all sites and branches) were performed to calculate the dN/dS for each of the branches of the gene tree. The comparison between two models was conducted using a likelihood ratio test (LRT) followed by a chi-squared test of significance.
